# Supplementary material for: Circumpolar spread of avian influenza H5N1 to southern Indian Ocean islands
Source: Nat Commun. 2025 Sep 29;16:8463. doi: 10.1038/s41467-025-64297-y (PMC12479825; doi:10.1038/s41467-025-64297-y)
Supplement: Supplementary file 1 — Supplementary Information [file 41467_2025_64297_MOESM1_ESM.pdf]

Extended Data and Figures – Clessin et al. 2025. ***Nature Communications***

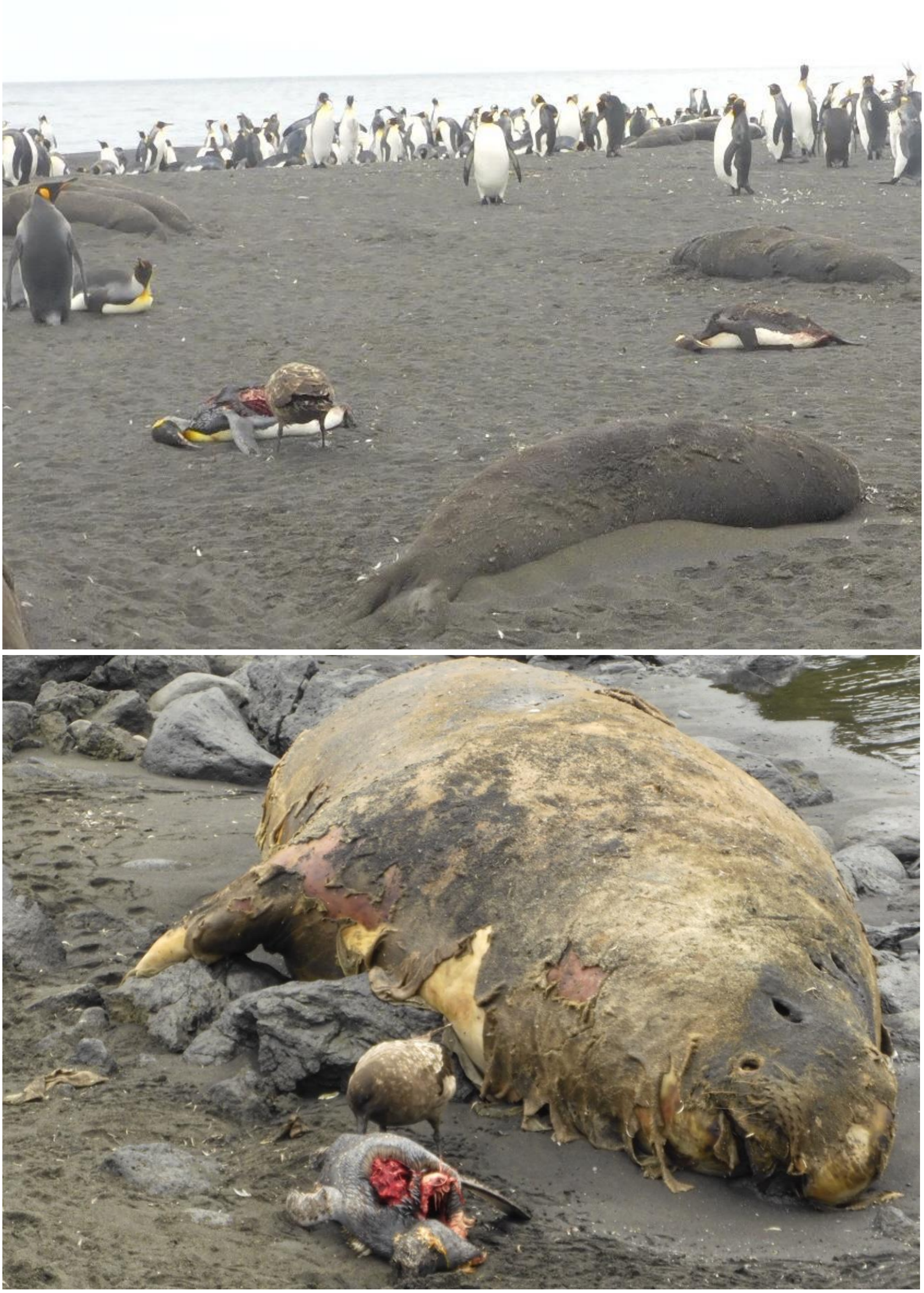

Extended Data Fig. 1 | Detailed pictures of brown skuas scavenging on king penguins on Petite Manchotière, Possession Island, Crozet archipelago. Picture credit: Jérémy Tornos/Mathilde Lejeune, CNRS/IPEV.

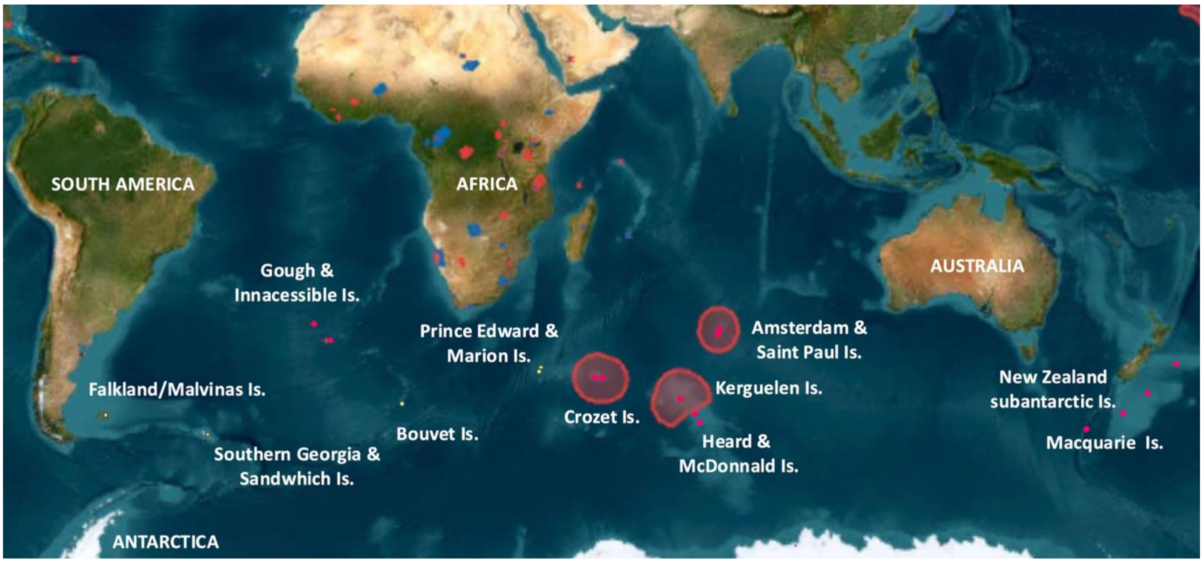

Extended Data Fig. 2 | Map showing sub-Antarctic islands of high relevance for southern marine mammal and seabird populations. Islands and areas of UNESCO World Heritage Sites in pink (modified from Earthstar Geographics, UNESCO World Heritage Sites, https://whc.unesco.org/en/list/).

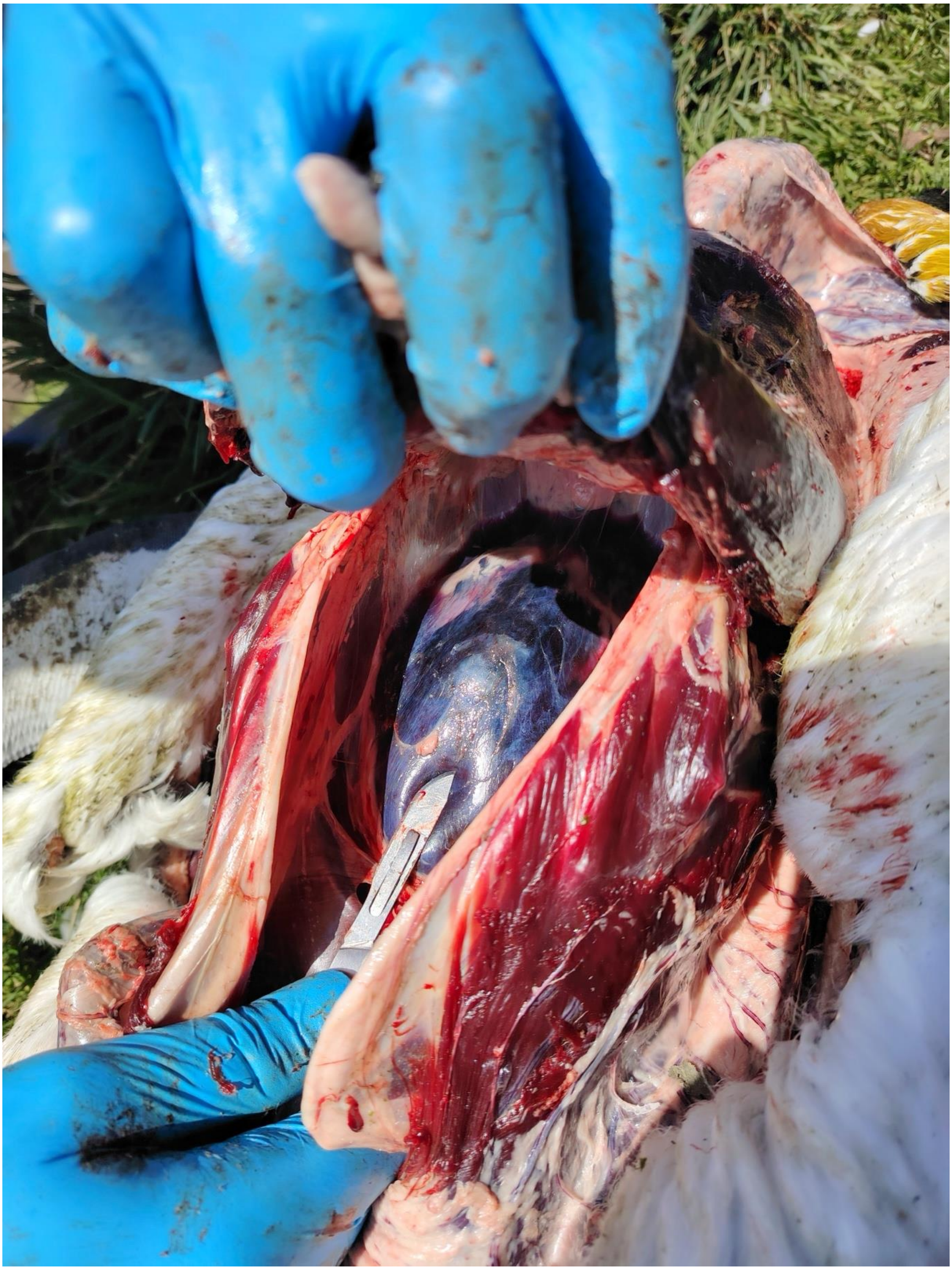

Extended Data Fig. 3 | Picture of a necropsied king penguin, positive by qRT-PCR **for HPAI H5N1 clade 2.3.4.4b virus.** The necropsy shows hemorrhage into the pericardial space (haemopericardium) as a macroscopic lesion. Picture credit: Jérémy Tornos/Mathilde Lejeune, CNRS/IPEV.

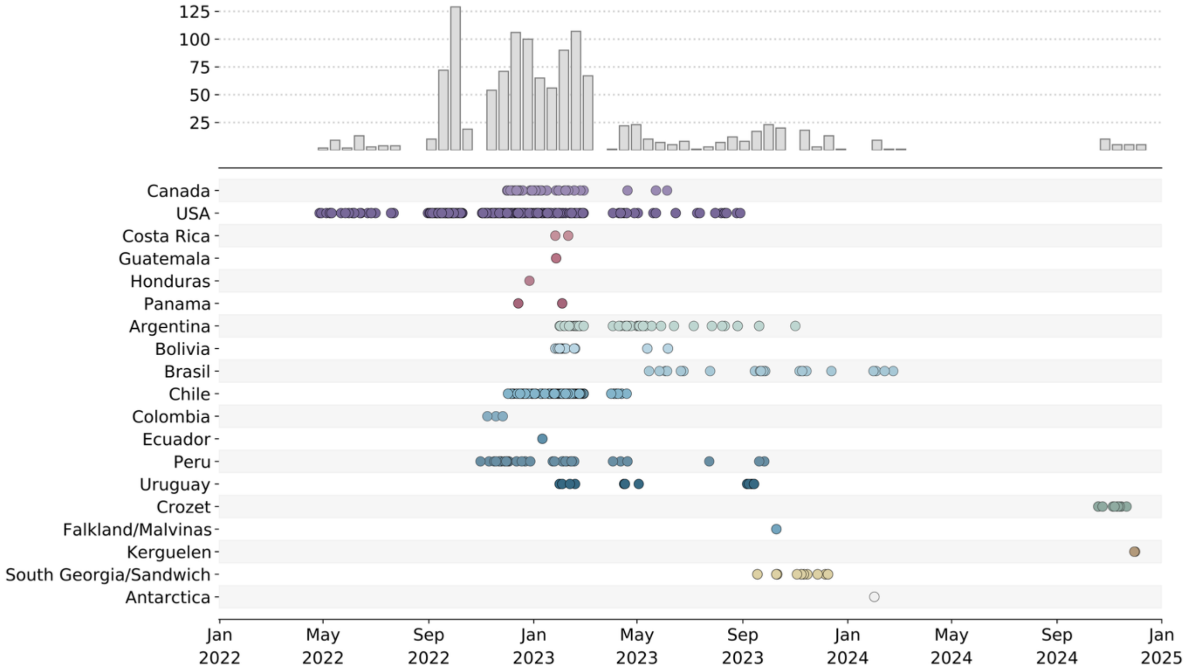

Extended Data Fig. 4 | Known locations and dates in our well-supported phylogeny of genomes that contains the samples from Crozet and Kerguelen islands. Rows show the collection dates of genomes on the bottom, as well as the frequency of genomes as a bar plot at the top. Histogram bars use 2-week bins.

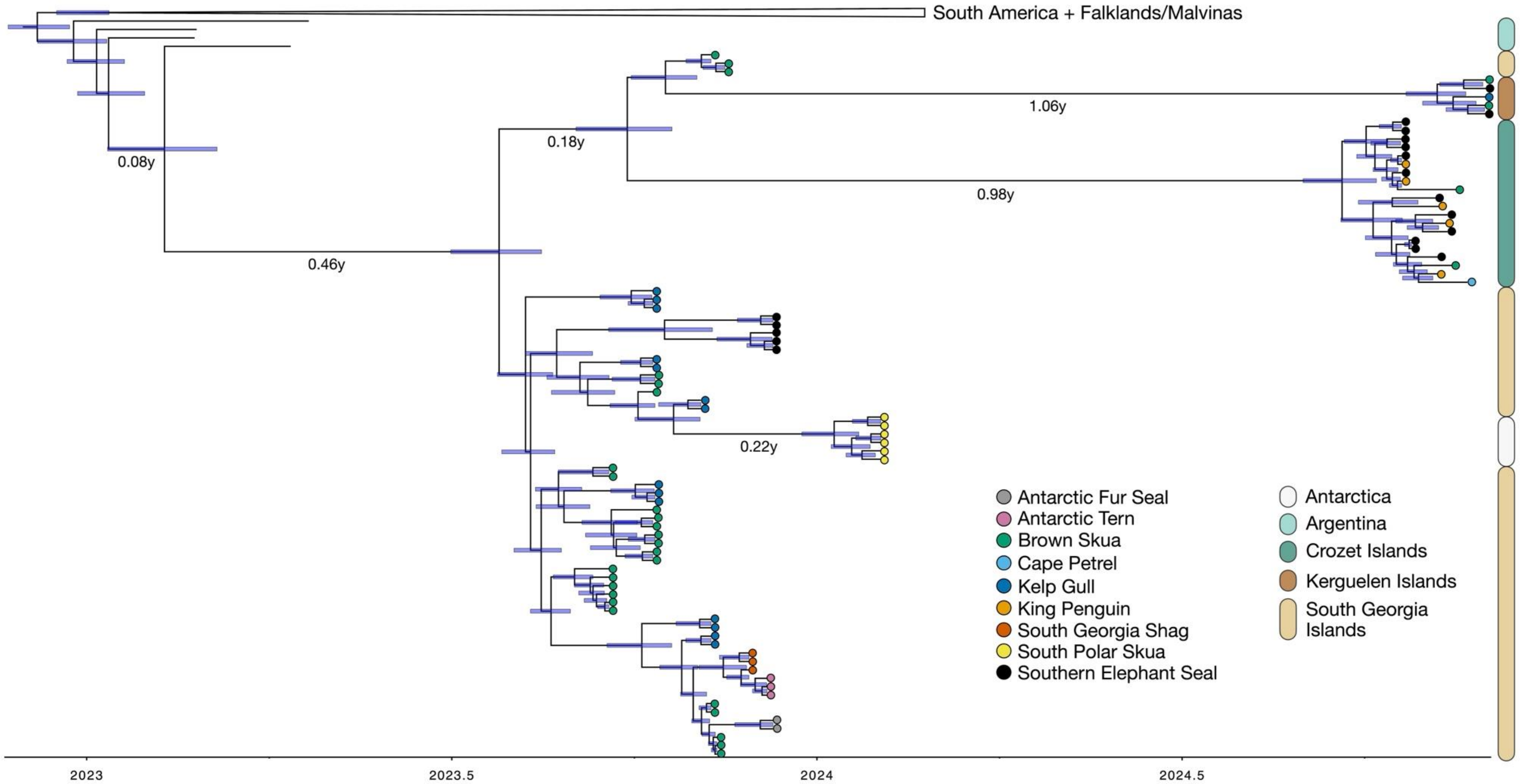

Extended Data Fig. 5 | The main cluster of interest in our consensus tree, containing our novel sequences from the Crozet and Kerguelen islands, in October and November of 2024. These sequences cluster among a larger number of sequences from the South Georgia islands. This implies two separate introductions - one into the Crozet islands and another into the Kerguelen islands - from the South Georgia islands, based on the currently available genetic data. Importantly, the very long branches of approximately 1 year, on top of a branch of nearly six months for the first introduction into the South Georgia islands, illustrate significant gaps in (animal / pathogen) surveillance in the Antarctic and sub-Antarctic regions. Horizontal blue bars represent the 95% HPD intervals for the ancestral node date estimates. Color scheme according to ColorBrewer 2.0 [75].

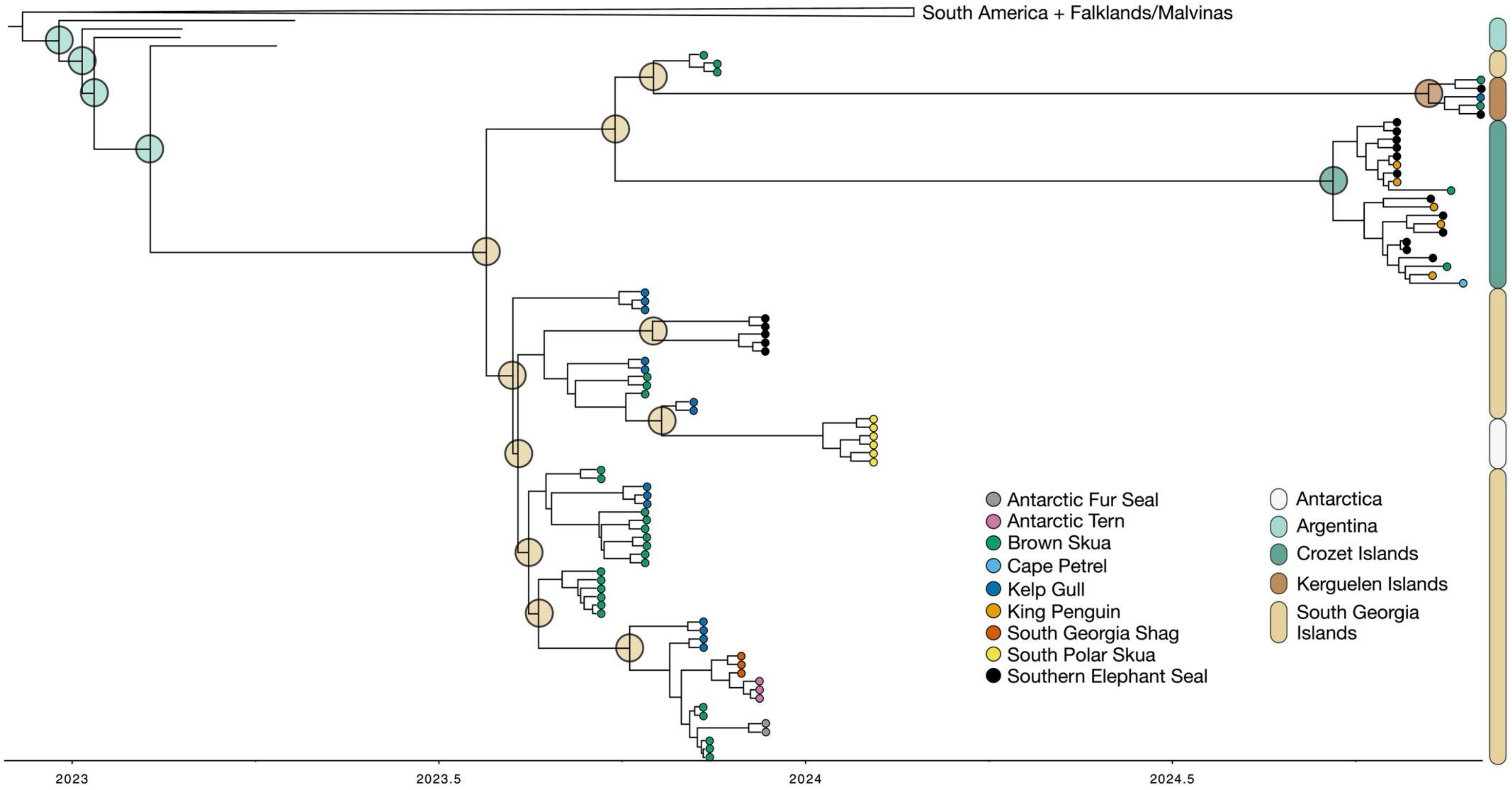

Extended Data Fig. 6 | Discrete phylogeographic reconstruction reveals South Georgia Island as the source for the (independent) introductions of H5N1 into the Crozet islands, the Kerguelen islands, and Antarctica. All inferred ancestral locations in this phylogeny - the most relevant ones indicated by semi-transparent colored circles - have 100% posterior probability.

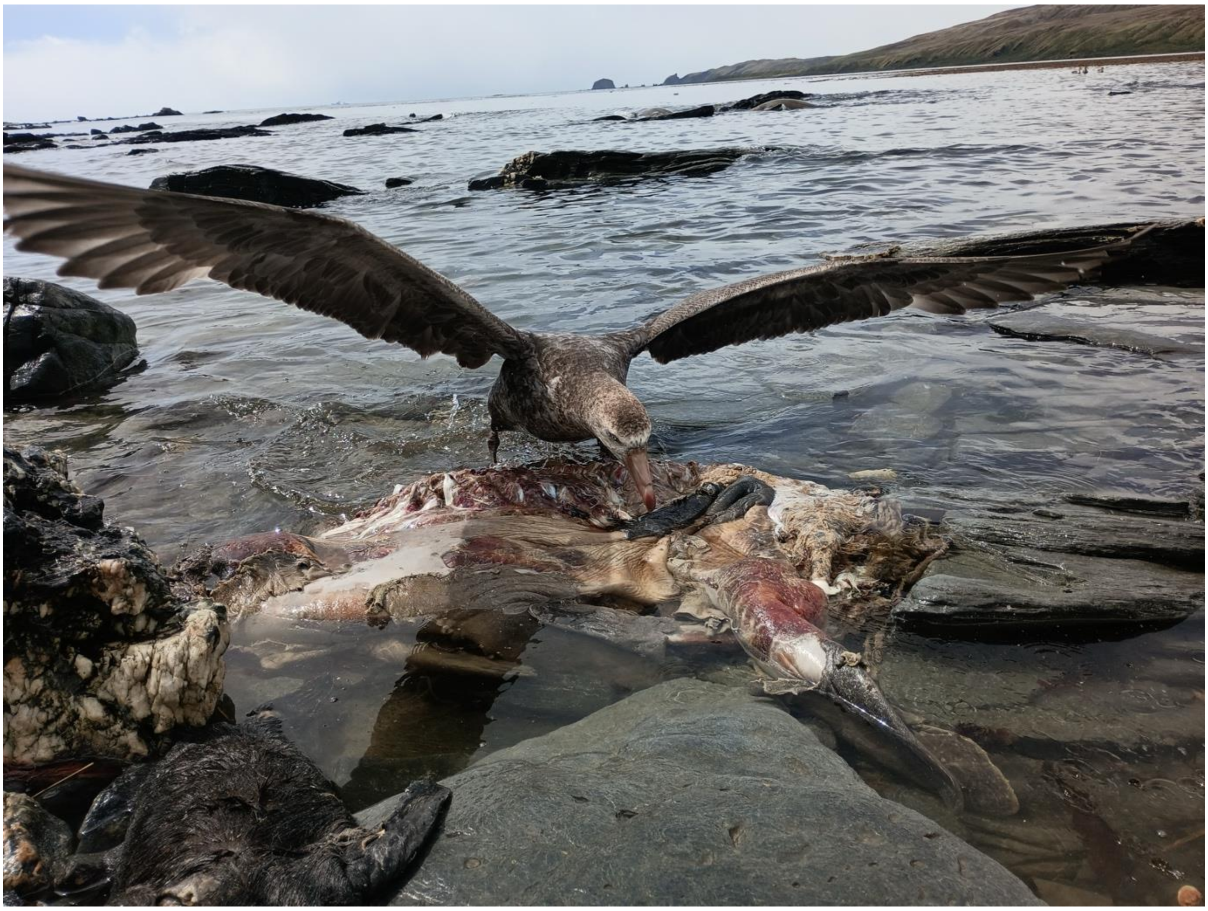

Extended Data Fig. 7 | A northern giant petrel scavenging on a southern elephant seal pup carcass. Picture credit: Augustin Clessin.

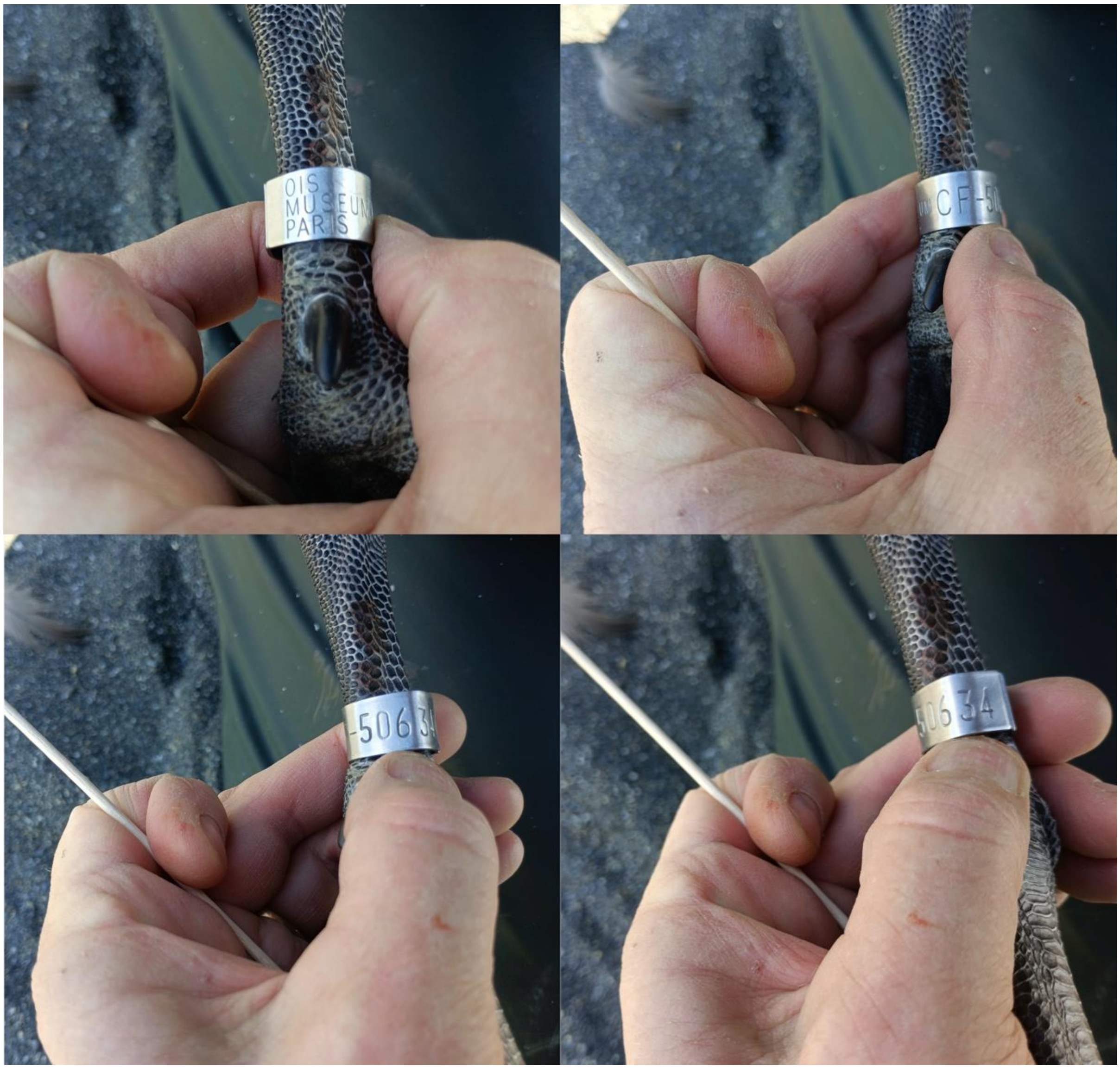

Extended Data Fig. 8 | Ringed northern giant petrel found on South Georgia Island. Captured on the 17th of January, 2025, in South Georgia by one of the authors of this study (AC), a northern giant petrel was found to have been ringed on Crozet islands as a chick, at Pointe Basse, on the 14th of January, 2024. Picture credit: Augustin Clessin.

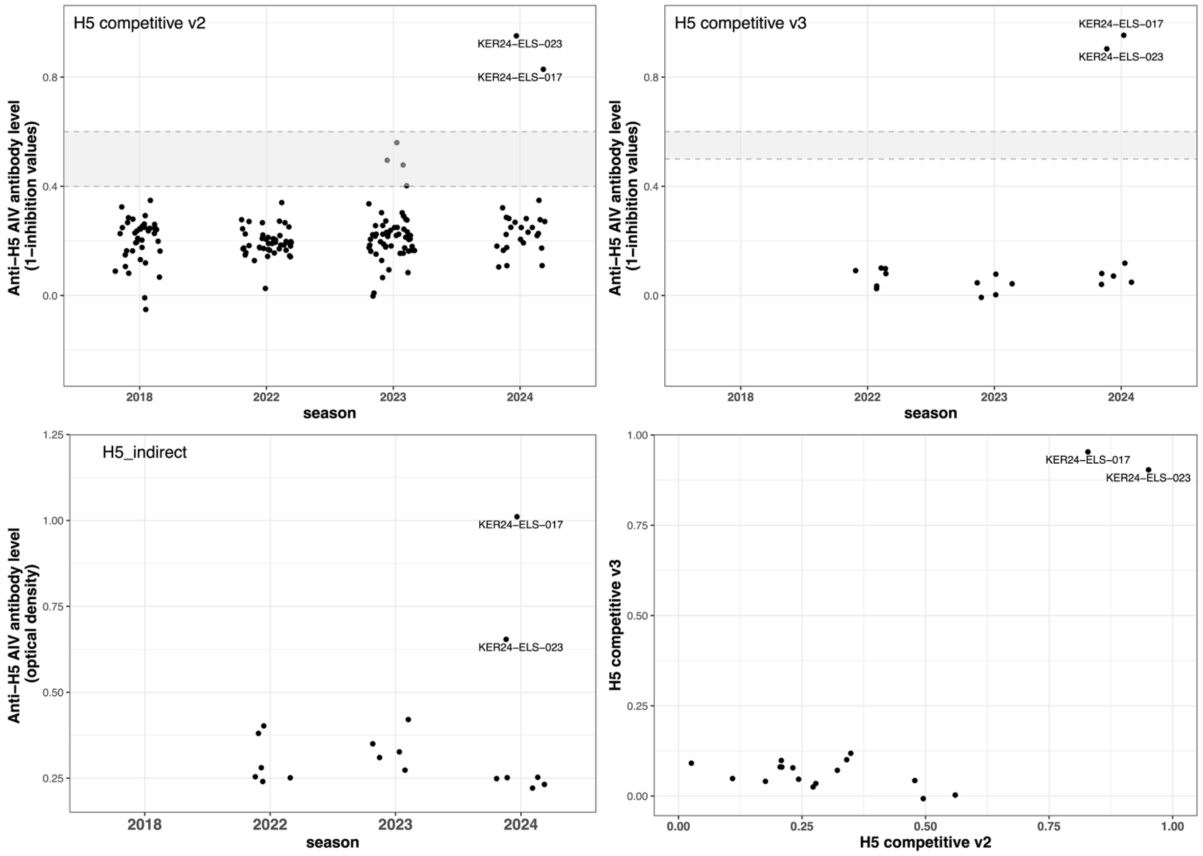

Extended Data Fig. 9 | Competitive and indirect ELISA for H5 proteins performed on southern elephant seal plasmas. Top left plot is a reproduction of Figure 5 for comparison purposes. The grey area displays the range for inconclusive H5-seropositivity results, according to the kit manufacturer. A jitter was introduced to displace the data points horizontally to improve their visibility. Below right plot shows the correlation between the v2 and v3 multispecies H5 ELISA. The results confirm that two of the pup samples were seropositive against H5 AIV protein in 2024, with no evidence of exposure to H5 AIV prior to that season.

Host	Site	Location	Age	Collection date	Influenza A antigenic test	Ct value, target 1	Ct value, target 2	GISAID accession no. Genbank accession no.	Sequence name
Mirounga leonina	Crozet	Baie du Marin	Pup	20 Oct. 2024	n.t.	18.5	20.4	EPI_ISL_19747197 PV294985 – PV294992	A/southern_elephant_seal/Crozet/24P021402/2024
Mirounga leonina	Crozet	Baie du Marin	Pup	20 Oct. 2024	n.t.	23.5	25.5	EPI_ISL_19747198 PV294993 – PV295000	A/southern_elephant_seal/Crozet/24P021403/2024
Mirounga leonina	Crozet	Baie du Marin	Pup	20 Oct. 2024	n.t.	15.1	17.7	EPI_ISL_19747199 PV295137 – PV295144	A/southern_elephant_seal/Crozet/24P021404/2024
Mirounga leonina	Crozet	Baie du Marin	Pup	20 Oct. 2024	n.t.	18.5	20.5		
Mirounga leonina	Crozet	Baie du Marin	Pup	20 Oct. 2024	n.t.	18.1	20.6		
Mirounga leonina	Crozet	Baie du Marin	Pup	20 Oct. 2024	n.t.	16.9	19.4	EPI_ISL_19747200 PV295145 – PV295152	A/southern_elephant_seal/Crozet/24P021407/2024
Mirounga leonina	Crozet	Baie du Marin	Pup	20 Oct. 2024	n.t.	24.4	25.9		
Mirounga leonina	Crozet	Baie du Marin	Pup	20 Oct. 2024	n.t.	15.8	17.8		
Mirounga leonina	Crozet	Baie du Marin	Pup	20 Oct. 2024	n.t.	16.8	18.8		
Mirounga leonina	Crozet	Baie du Marin	Pup	20 Oct. 2024	n.t.	15.7	17.8		
Aptenodytes patagonicus	Crozet	Baie du Marin	Adult	20 Oct. 2024	n.t.	27	29	EPI_ISL_19747201 PV295121 – PV295128	A/king_penguin/Crozet/24P021412/2024
Aptenodytes patagonicus	Crozet	Baie du Marin	Adult	20 Oct. 2024	n.t.	34.5	undet		
Aptenodytes patagonicus	Crozet	Baie du Marin	Adult	20 Oct. 2024	n.t.	25.3	34.3	EPI_ISL_19747202 PV295129 – PV295136	A/king_penguin/Crozet/24P021416/2024
Mirounga leonina	Crozet	Baie du Marin	Pup	20 Oct. 2024	n.t.	18.7	20.6		
Mirounga leonina	Crozet	Baie du Marin	Pup	20 Oct. 2024	n.t.	17.6	18.8	EPI_ISL_19747210 PV295153 – PV295160	A/southern_elephant_seal/Crozet/24P021415/2024
Mirounga leonina	Crozet	Baie du Marin	Pup	20 Oct. 2024	n.t.	15.5	17.5	EPI_ISL_19747211 PV295161 – PV295168	A/southern_elephant_seal/Crozet/24P021417/2024
Mirounga leonina	Crozet	Baie du Marin	Pup	20 Oct. 2024	n.t.	25.7	28		
Mirounga leonina	Crozet	Jardin Japonais	Adult	27 Oct. 2024	-	undet	undet		

Mirounga leonina	Crozet	Jardin Japonais	Pup	27 Oct. 2024	+	16.9	18.9	EPI_ISL_19747204 PV295169 – PV295176	A/southern_elephant_seal/Crozet/24P021420/2024
Mirounga leonina	Crozet	Jardin Japonais	Pup	27 Oct. 2024	+	17.1	19		
Mirounga leonina	Crozet	Jardin Japonais	Pup	27 Oct. 2024	+	18.1	20.5	EPI_ISL_19747205 PV295177 – PV295184	A/southern_elephant_seal/Crozet/24P021422/2024
Mirounga leonina	Crozet	Mare aux éléphants	Adult	06 Nov. 2024	+	29.5	32		
Mirounga leonina	Crozet	Mare aux éléphants	Pup	06 Nov. 2024	+	undet	undet		
Mirounga leonina	Crozet	Mare aux éléphants	Pup	06 Nov. 2024		undet	32.3		
Mirounga leonina	Crozet	Mare aux éléphants	Pup	06 Nov. 2024		undet	38.4		
Mirounga leonina	Crozet	Mare aux éléphants	Pup	06 Nov. 2024	n.t.	20.6	23	EPI_ISL_19747207 PV295017 – PV295024	A/southern_elephant_seal/Crozet/24P023403/2024
Mirounga leonina	Crozet	Jardin Japonais	Pup	07 Nov. 2024	n.t.	18.3	20.7	EPI_ISL_19747206 PV295009 – PV295016	A/southern_elephant_seal/Crozet/24P023401/2024
Aptenodytes patagonicus	Crozet	Jardin Japonais	Adult	07 Nov. 2024	+	22.4	25.1	EPI_ISL_19747213 PV295001 – PV295008	A/king_penguin/Crozet/24P023398/2024
Aptenodytes patagonicus	Crozet	Jardin Japonais	Adult	07 Nov. 2024		26.9	30		
Aptenodytes patagonicus	Crozet	Jardin Japonais	Adult	08 Nov. 2024	n.t.	22	24.7		
Aptenodytes patagonicus	Crozet	Jardin Japonais	Adult	08 Nov. 2024	n.t.	17	19.9	EPI_ISL_19747212 PV295025 – PV295032	A/king_penguin/Crozet/24P023407/2024
Aptenodytes patagonicus	Crozet	Jardin Japonais	Adult	08 Nov. 2024	n.t.	25.2	28		
Pygoscelis papua	Crozet	Baie du Marin	Adult	10 Nov. 2024	-	31.6	34.6		
Mirounga leonina	Crozet	Petite Manchotière	Adult	11 Nov. 2024	n.t.	undet	undet		
Aptenodytes patagonicus	Crozet	Petite Manchotière	Adult	11 Nov. 2024	n.t.	22	24.3	EPI_ISL_19747214 PV295041 – PV295048	A/king_penguin/Crozet/24P023414/2024
Mirounga leonina	Crozet	Chaloupe	Pup	12 Nov. 2024	+	23.7	26.9		
Mirounga leonina	Crozet	Chaloupe	Pup	12 Nov. 2024		27	29.1		
Mirounga leonina	Crozet	Chaloupe	Pup	12 Nov. 2024		18.5	20.4	EPI_ISL_19747208 PV295049 – PV295056	A/southern_elephant_seal/Crozet/24P023417/2024

Mirounga leonina	Crozet	Chaloupe	Pup	12 Nov. 2024		27.7	37.3		
Mirounga leonina	Crozet	Chaloupe	Pup	12 Nov. 2024	+	18.4	21	EPI_ISL_19747209 PV295057 – PV295064	A/southern_elephant_seal/Crozet/24P023419/2024
Mirounga leonina	Crozet	Chaloupe	Pup	12 Nov. 2024		22.5	25.1		
Aptenodytes patagonicus	Crozet	Chaloupe	Adult	12 Nov. 2024	-	29.6	undet		
Stercorarius antarcticus	Crozet	Baie Américaine	Adult	14 Nov. 2024	+	32.2	undet		
Stercorarius antarcticus	Crozet	Baie Américaine	Adult	14 Nov. 2024		22.8	25.8	EPI_ISL_19747215 PV295033 – PV295040	A/brown_skua/Crozet/24P023412/2024
Aptenodytes patagonicus	Crozet	Baie Américaine	Adult	14 Nov. 2024	n.t.	29.2	32.4		
Mirounga leonina	Crozet	Baie du Marin	Pup	16 Nov. 2024		17.1	19.8	EPI_ISL_19747203 PV295065 – PV295072	A/southern_elephant_seal/Crozet/24P023422/2024
Mirounga leonina	Crozet	Baie du Marin	Pup	16 Nov. 2024	+	17.2	19.9		
Mirounga leonina	Crozet	Baie du Marin	Pup	16 Nov. 2024		22.3	27.7		
Pygoscelis papua	Crozet	Baie du Marin	Chick	16 Nov. 2024	-	undet	undet		
Aptenodytes patagonicus	Crozet	La Pérouse	Adult	19 Nov. 2024	-	undet	undet		
Daption capense	Crozet	Sphinx	Adult	22 Nov. 2024	+	19.2	21.4	EPI_ISL_19747216 PV295073 – PV295080	A/cape_petrel/Crozet/24P023427/2024
Mirounga leonina	Crozet	Baie du Marin	Pup	24 Nov. 2024	+	23	24.7		
Mirounga leonina	Crozet	Baie du Marin	Pup	24 Nov. 2024		undet	undet		
Stercorarius antarcticus	Kerguelen	Ratmanoff	Adult	01 Dec. 2024		21.5	22.7		
Stercorarius antarcticus	Kerguelen	Ratmanoff	Adult	01 Dec. 2024	+	28	29		
Stercorarius antarcticus	Kerguelen	Ratmanoff	Adult	01 Dec. 2024		18	18.9	EPI_ISL_19747220 PV295113 – PV295120	A/brown_skua/Kerguelen/24P023446/2024
Stercorarius antarcticus	Kerguelen	Ratmanoff	Adult	01 Dec. 2024	n.t.	24.7	26		
Mirounga leonina	Kerguelen	Ratmanoff	Pup	01 Dec. 2024	+	21	22.1		
Mirounga leonina	Kerguelen	Ratmanoff	Pup	01 Dec. 2024		16.1	17.3	EPI_ISL_19747217 PV295089 – PV295096	A/southern_elephant_seal/Kerguelen/24P023439/2024

Mirounga leonina	Kerguelen	Ratmanoff	Pup	01 Dec. 2024		18.6	19.5	EPI_ISL_19747218 PV295081 – PV295088	A/southern_elephant_seal/Kerguelen/24P023433/2024
Mirounga leonina	Kerguelen	Ratmanoff	Pup	01 Dec. 2024	n.t.	21.3	22		
Mirounga leonina	Kerguelen	Ratmanoff	Pup	01 Dec. 2024	n.t.	19	20		
Mirounga leonina	Kerguelen	Ratmanoff	Pup	01 Dec. 2024	n.t.	22.4	23.3		
Mirounga leonina	Kerguelen	Ratmanoff	Pup	01 Dec. 2024	n.t.	24.6	25.7		
Mirounga leonina	Kerguelen	Ratmanoff	Pup	01 Dec. 2024	n.t.	23	23.7		
Mirounga leonina	Kerguelen	Ratmanoff	Pup	01 Dec. 2024	n.t.	18.8	19.9		
Mirounga leonina	Kerguelen	Ratmanoff	Pup	01 Dec. 2024	n.t.	18.6	19.8		
Larus dominicanus	Kerguelen	Ratmanoff	Immature	01 Dec. 2024		25	25.4		
Larus dominicanus	Kerguelen	Ratmanoff	Immature	01 Dec. 2024	+	21.25	22		
Larus dominicanus	Kerguelen	Ratmanoff	Immature	01 Dec. 2024		20.1	21.1	EPI_ISL_19747219 PV295097 – PV295104	A/kelp_gull/Kerguelen/24P023440/2024
Stercorarius antarcticus	Kerguelen	Port aux Français	Adult	02 Dec. 2024	+	17.6	19.1	EPI_ISL_19747221 PV295105 – PV295112	A/brown_skua/Kerguelen/24P023443/2024

Extended Data Table 1 | List of the samples we collected as part of our study. The table indicates the species name, the location, the age of the individual sampled, the sampling date, the influenza A antigenic test result (against avian influenza NP protein) and the qRT-PCR results - negative samples are indicated as "undet", and positive ones are reported with the associated Ct value. We consider a sample to be positive when at least one of the two targets of the qRT-PCR is positive. All samples are brain swabs collected on carcasses. They were stored dry and frozen at -80°C. Influenza A antigenic tests were conducted directly on the field ("**+**" and "-" indicates respectively a positive and a negative antigenic test; "*n.t.*" indicates that the sample has not been tested). Pooled antigenic tests are indicated when a result spans multiple rows. qRT-PCR H5 HP clade 2.3.4.4b were conducted on all collected samples and results are presented as "Detected", "Not detected" or "*Inconclusive*" for each sample. We provide GISAID and Genbank accession numbers and the sequence names of the samples that have been sequenced.

Sample	HA	M	NP	NA	NS	PA	PB1	PB2	genome coverage (%)
24P021402	Complete	Complete	Complete	Complete	Complete	Partial	Partial	Partial	62
24P021403	Complete	Complete	Complete	Complete	Complete	Partial	Complete	Partial	76
24P021404	Complete	Complete	Complete	Complete	Complete	Complete	Complete	Complete	100
24P021407	Complete	Complete	Complete	Complete	Complete	Complete	Complete	Complete	100
24P021412	Complete	Complete	Complete	Complete	Complete	Complete	Complete	Complete	100
24P021415	Complete	Complete	Complete	Complete	Complete	Complete	Complete	Complete	100
24P021416	Complete	Complete	Complete	Complete	Complete	Complete	Complete	Complete	99
24P021417	Complete	Complete	Complete	Complete	Complete	Complete	Complete	Complete	100
24P021420	Complete	Complete	Complete	Complete	Complete	Complete	Complete	Complete	100
24P021422	Complete	Complete	Complete	Complete	Complete	Complete	Complete	Complete	100
24P023398	Complete	Complete	Complete	Complete	Complete	Partial	Partial	Partial	71
24P023401	Complete	Complete	Complete	Complete	Complete	Partial	Partial	Partial	66
24P023403	Complete	Complete	Complete	Complete	Complete	Partial	Partial	Partial	64
24P023407	Complete	Complete	Complete	Complete	Complete	Partial	Partial	Partial	63
24P023412	Complete	Complete	Complete	Complete	Complete	Partial	Partial	Partial	66
24P023414	Complete	Complete	Complete	Complete	Complete	Partial	Partial	Partial	70
24P023417	Complete	Complete	Complete	Complete	Complete	Partial	Partial	Partial	61
24P023419	Complete	Complete	Complete	Complete	Complete	Partial	Partial	Partial	61
24P023422	Complete	Complete	Complete	Complete	Complete	Partial	Partial	Partial	71
24P023427	Complete	Complete	Complete	Complete	Complete	Partial	Partial	Partial	70
24P023433	Complete	Complete	Complete	Complete	Complete	Partial	Partial	Partial	64
24P023439	Complete	Complete	Complete	Complete	Complete	Partial	Partial	Partial	67
24P023440	Complete	Complete	Complete	Complete	Complete	Partial	Partial	Partial	66
24P023443	Complete	Complete	Complete	Complete	Complete	Partial	Partial	Partial	63
24P023446	Complete	Complete	Complete	Complete	Complete	Complete	Partial	Partial	78

Extended Data Table 2 | Sequencing summary. The completeness of each segment is reported for all samples, together with the overall genome coverage.
