# Supplementary material for: Circumpolar spread of avian influenza H5N1 to southern Indian Ocean islands
Source: Nat Commun. 2025 Sep 29;16:8463. doi: 10.1038/s41467-025-64297-y (PMC12479825; doi:10.1038/s41467-025-64297-y)
Supplement: Supplementary file 3 — Description of Additional Supplementary Files [file 41467_2025_64297_MOESM3_ESM.pdf]

Description of Additional Supplementary Data of Clessin et al. 2025 *Nature Communications*

Supplementary Data 1 the detail of the mutation analysis

Supplementary Data 2 the serology analyses results

Supplementary Data 3 the file to reproduce the BEAST analysis (with the alignment removed to comply with GISAID terms of use)

Supplementary Data 4 the complete phylogenetic tree of Figure 3 in Nexus format

Supplementary Data 5 the GISAID acknowledgement link and the DOI associated with our dataset

Supplementary Data 6 the FASTA file with our newly obtained sequences

Supplementary Data 7 the metadata associated with those sequences

Supplementary Data 8 a table that maps the GISAID and Genbank accession numbers to the individual gene sequences

Supplementary Data 9 a table with the strain names, GISAID accession numbers and Genbank accession numbers.
