# Supplementary material for: Circumpolar spread of avian influenza H5N1 to southern Indian Ocean islands
Source: Nat Commun. 2025 Sep 29;16:8463. doi: 10.1038/s41467-025-64297-y (PMC12479825; doi:10.1038/s41467-025-64297-y)
Supplement: Supplementary file 6 — Supplementary Data 3 [file 41467_2025_64297_MOESM6_ESM.pdf]

```
<!--  Generated by BEAUTi v10.5.0-beta5 Prerelease #691e5ff44
-->
<!--        by Alexei J. Drummond, Andrew Rambaut and Marc A. Suchard
-->
<!--        Department of Computer Science, University of Auckland and
-->
<!--        Institute of Evolutionary Biology, University of Edinburgh
-->
<!--        David Geffen School of Medicine, University of California, Los
Angeles -->
<!--        http://beast.community/
-->
<beast version="10.5.0-beta5">
<!--  The list of taxa to be analysed (can also include dates/ages).
-->
<!--  ntax=1227
-->
<taxa id="taxa">
<taxon id="24P021402_H5N1_D-24-09077_HP_B3.2_CRO_2024-10-20">
<date value="2024.8005464480875" direction="forwards" units="years"/>
</taxon>
<taxon id="24P021403_H5N1_D-24-09077_HP_B3.2_CRO_2024-10-20">
<date value="2024.8005464480875" direction="forwards" units="years"/>
</taxon>
<taxon id="24P021404_H5N1_D-24-09077_HP_B3.2_CRO_2024-10-20">
<date value="2024.8005464480875" direction="forwards" units="years"/>
</taxon>
<taxon id="24P021407_H5N1_D-24-09077_HP_B3.2_CRO_2024-10-20">
<date value="2024.8005464480875" direction="forwards" units="years"/>
</taxon>
<taxon id="24P021412_H5N1_D-24-09077_HP_B3.2_CRO_2024-10-20">
<date value="2024.8005464480875" direction="forwards" units="years"/>
</taxon>
<taxon id="24P021415_H5N1_D-24-09077_HP_B3.2_CRO_2024-10-20">
<date value="2024.8005464480875" direction="forwards" units="years"/>
</taxon>
<taxon id="24P021416_H5N1_D-24-09077_HP_B3.2_CRO_2024-10-20">
<date value="2024.8005464480875" direction="forwards" units="years"/>
</taxon>
<taxon id="24P021417_H5N1_D-24-09077_HP_B3.2_CRO_2024-10-20">
<date value="2024.8005464480875" direction="forwards" units="years"/>
</taxon>
<taxon id="24P021420_H5N1_D-24-09077_HP_B3.2_CRO_2024-10-25">
<date value="2024.8142076502731" direction="forwards" units="years"/>
</taxon>
<taxon id="24P021422_H5N1_D-24-09077_HP_B3.2_CRO_2024-10-25">
<date value="2024.8142076502731" direction="forwards" units="years"/>
</taxon>
<taxon id="24P023398_H5N1_D-24-10045_HP_B3.2_CRO_2024-11-07">
<date value="2024.8497267759562" direction="forwards" units="years"/>
</taxon>
<taxon id="24P023401_H5N1_D-24-10045_HP_B3.2_CRO_2024-11-07">
<date value="2024.8497267759562" direction="forwards" units="years"/>
</taxon>
<taxon id="24P023403_H5N1_D-24-10045_HP_B3.2_CRO_2024-11-06">
<date value="2024.8469945355191" direction="forwards" units="years"/>
</taxon>
<taxon id="24P023407_H5N1_D-24-10045_HP_B3.2_CRO_2024-11-08">
<date value="2024.8524590163934" direction="forwards" units="years"/>
</taxon>
<taxon id="24P023412_H5N1_D-24-10045_HP_B3.2_CRO_2024-11-14">
```

<date value="2024.8688524590164" direction="forwards" units="years"/>
</taxon>
<taxon id="24P023414_H5N1_D-24-10045_HP_B3.2_CRO_2024-11-11">
<date value="2024.860655737705" direction="forwards" units="years"/>
</taxon>
<taxon id="24P023417_H5N1_D-24-10045_HP_B3.2_CRO_2024-11-12">
<date value="2024.863387978142" direction="forwards" units="years"/>
</taxon>
<taxon id="24P023419_H5N1_D-24-10045_HP_B3.2_CRO_2024-11-12">
<date value="2024.863387978142" direction="forwards" units="years"/>
</taxon>
<taxon id="24P023422_H5N1_D-24-10045_HP_B3.2_CRO_2024-11-16">
<date value="2024.8743169398906" direction="forwards" units="years"/>
</taxon>
<taxon id="24P023427_H5N1_D-24-10045_HP_B3.2_CRO_2024-11-22">
<date value="2024.8907103825136" direction="forwards" units="years"/>
</taxon>
<taxon id="24P023433_H5N1_D-24-10045_HP_B3.2_KER_2024-12-01">
<date value="2024.9153005464482" direction="forwards" units="years"/>
</taxon>
<taxon id="24P023439_H5N1_D-24-10045_HP_B3.2_KER_2024-12-01">
<date value="2024.9153005464482" direction="forwards" units="years"/>
</taxon>
<taxon id="24P023440_H5N1_D-24-10045_HP_B3.2_KER_2024-12-01">
<date value="2024.9153005464482" direction="forwards" units="years"/>
</taxon>
<taxon id="24P023443_H5N1_D-24-10045_HP_B3.2_KER_2024-12-02">
<date value="2024.9180327868853" direction="forwards" units="years"/>
</taxon>
<taxon id="24P023446_H5N1_D-24-10045_HP_B3.2_KER_2024-12-01">
<date value="2024.9153005464482" direction="forwards" units="years"/>
</taxon>
<taxon id="A/American_Crow/BC/AIVPHL-
861/2022|EPI_ISL_18665555|A_/_H5N1|Original||2.3.4.4b|30.12.2022|Caleta_Jes
sica_Marija|||21.12.2023|B.C._Centre_for_Disease_Control|Public_Health_Agen
cy_of_Canada|HA|4|A/American_Crow/BC/AIVPHL-
861/2022_HA|EPI2856376|DNA_INS">
<date value="2022.9972602739726" direction="forwards" units="years"/>
</taxon>
<taxon id="A/American_Crow/BC/AIVPHL-
864/2022|EPI_ISL_18665557|A_/_H5N1|Original||2.3.4.4b|30.12.2022|Caleta_Jes
sica_Marija|||21.12.2023|B.C._Centre_for_Disease_Control|Public_Health_Agen
cy_of_Canada|HA|4|A/American_Crow/BC/AIVPHL-
864/2022_HA|EPI2856394|DNA_INS">
<date value="2022.9972602739726" direction="forwards" units="years"/>
</taxon>
<taxon id="A/American_Crow/BC/AIVPHL-
874/2023|EPI_ISL_18665565|A_/_H5N1|Original||2.3.4.4b|09.01.2023|Caleta_Jes
sica_Marija|||21.12.2023|B.C._Centre_for_Disease_Control|Public_Health_Agen
cy_of_Canada|HA|4|A/American_Crow/BC/AIVPHL-
874/2023_HA|EPI2856448|DNA_INS">
<date value="2023.0246575342467" direction="forwards" units="years"/>
</taxon>
<taxon id="A/American_Crow/BC/AIVPHL-
900/2023|EPI_ISL_18665492|A_/_H5N1|Original||2.3.4.4b|16.01.2023|Caleta_Jes
sica_Marija|||21.12.2023|B.C._Centre_for_Disease_Control|Public_Health_Agen
cy_of_Canada|HA|4|A/American_Crow/BC/AIVPHL-
900/2023_HA|EPI2855906|DNA_INS">
<date value="2023.0438356164384" direction="forwards" units="years"/>
</taxon>

<taxon id="A/American_Crow/BC/AIVPHL-921/2023|EPI_ISL_18665496|A_/_H5N1|Original||2.3.4.4b|30.01.2023|Caleta_Jessica_Marija|||21.12.2023|B.C._Centre_for_Disease_Control|Public_Health_Agency_of_Canada|HA|4|A/American_Crow/BC/AIVPHL-921/2023_HA|EPI2855933|DNA_INS">
<date value="2023.0821917808219" direction="forwards" units="years"/>
</taxon>
<taxon id="A/American_Crow/BC/AIVPHL-922/2023|EPI_ISL_18665498|A_/_H5N1|Original||2.3.4.4b|30.01.2023|Caleta_Jessica_Marija|||21.12.2023|B.C._Centre_for_Disease_Control|Public_Health_Agency_of_Canada|HA|4|A/American_Crow/BC/AIVPHL-922/2023_HA|EPI2855949|DNA_INS">
<date value="2023.0821917808219" direction="forwards" units="years"/>
</taxon>
<taxon id="A/American_Crow/BC/AIVPHL-929/2023|EPI_ISL_18665506|A_/_H5N1|Original||2.3.4.4b|30.01.2023|Caleta_Jessica_Marija|||21.12.2023|B.C._Centre_for_Disease_Control|Public_Health_Agency_of_Canada|HA|4|A/American_Crow/BC/AIVPHL-929/2023_HA|EPI2856013|DNA_INS">
<date value="2023.0821917808219" direction="forwards" units="years"/>
</taxon>
<taxon id="A/American_Crow/BC/FAV-0053-11/2022|EPI_ISL_19155031|A_/_H5N1|||2.3.4.4b|02.12.2022|Signore_Anthony|||21.05.2024|Canadian_Food_Inspection_Agency|Canadian_Food_Inspection_Agency|HA|4|A/American_Crow/BC/FAV-0053-11/2022_HA|EPI3314928|DNA_IN">
<date value="2022.9205479452055" direction="forwards" units="years"/>
</taxon>
<taxon id="A/American_Crow/BC/FAV-0053-19/2022|EPI_ISL_19155032|A_/_H5N1|||2.3.4.4b|02.12.2022|Signore_Anthony|||21.05.2024|Canadian_Food_Inspection_Agency|Canadian_Food_Inspection_Agency|HA|4|A/American_Crow/BC/FAV-0053-19/2022_HA|EPI3314936|DNA_IN">
<date value="2022.9205479452055" direction="forwards" units="years"/>
</taxon>
<taxon id="A/American_Crow/BC/FAV-0053-21/2022|EPI_ISL_19155079|A_/_H5N1|||2.3.4.4b|11.12.2022|Signore_Anthony|||21.05.2024|Canadian_Food_Inspection_Agency|Canadian_Food_Inspection_Agency|HA|4|A/American_Crow/BC/FAV-0053-21/2022_HA|EPI3315312|DNA_IN">
<date value="2022.945205479452" direction="forwards" units="years"/>
</taxon>
<taxon id="A/American_Crow/BC/FAV-0053-22/2022|EPI_ISL_19155033|A_/_H5N1|||2.3.4.4b|02.12.2022|Signore_Anthony|||21.05.2024|Canadian_Food_Inspection_Agency|Canadian_Food_Inspection_Agency|HA|4|A/American_Crow/BC/FAV-0053-22/2022_HA|EPI3314944|DNA_IN">
<date value="2022.9205479452055" direction="forwards" units="years"/>
</taxon>
<taxon id="A/American_Crow/BC/FAV-0053-24/2022|EPI_ISL_19155111|A_/_H5N1|||2.3.4.4b|16.12.2022|Signore_Anthony|||21.05.2024|Canadian_Food_Inspection_Agency|Canadian_Food_Inspection_Agency|HA|4|A/American_Crow/BC/FAV-0053-24/2022_HA|EPI3315568|DNA_IN">
<date value="2022.958904109589" direction="forwards" units="years"/>
</taxon>
<taxon id="A/American_Crow/BC/FAV-0053-42/2023|EPI_ISL_19155140|A_/_H5N1|||2.3.4.4b|02.01.2023|Signore_Anthony|||21.05.2024|B.C._Centre_for_Disease_Control|Canadian_Food_Inspection_Agency|HA|4|A/American_Crow/BC/FAV-0053-42/2023_HA|EPI3315800|DNA_IN">
<date value="2023.0054794520547" direction="forwards" units="years"/>
</taxon>
<taxon id="A/American_crow/Iowa/23-007468-001/2023|EPI_ISL_17964890|A_/_H5N1|Original||2.3.4.4b|02.03.2023|Killian_Mary_Lea||23-007468-

001|06.07.2023|National_Veterinary_Services_Laboratories_-
_USDA|National_Veterinary_Services_Laboratories_-_USDA|HA|4|HA_A/Amer">
<date value="2023.0821917808219" direction="forwards" units="years"/>
</taxon>
<taxon id="A/American_crow/Iowa/23-007781-
002/2023|EPI_ISL_17964893|A_/_H5N1|Original||2.3.4.4b|05.03.2023|Killian_Ma
ry_Lea||23-007781-
002|06.07.2023|National_Veterinary_Services_Laboratories_-
_USDA|National_Veterinary_Services_Laboratories_-_USDA|HA|4|HA_A/Amer">
<date value="2023.0904109589042" direction="forwards" units="years"/>
</taxon>
<taxon id="A/American_crow/Utah/23-007867-
006/2023|EPI_ISL_17964896|A_/_H5N1|Original||2.3.4.4b|03.03.2023|Killian_Ma
ry_Lea||23-007867-
006|06.07.2023|National_Veterinary_Services_Laboratories_-
_USDA|National_Veterinary_Services_Laboratories_-_USDA|HA|4|HA_A/Amer">
<date value="2023.0849315068492" direction="forwards" units="years"/>
</taxon>
<taxon id="A/Antarctic_Fur_Seal/Jason_Harbour/141037/2023|EPI_ISL_18742212|
A_/_H5N1|Clinical_Sample||2.3.4.4b|09.12.2023|Mollett_Benjamin|||25.06.2024
|Animal_and_Plant_Health_Agency__APHA_|Animal_and_Plant_Health_Agency__APHA
_|HA|4|A/Antarctic_Fur_Seal/Jason_Har">
<date value="2023.9397260273972" direction="forwards" units="years"/>
</taxon>
<taxon id="A/Antarctic_Fur_Seal/South_Georgia_and_the_South_Sandwich_Island
s/128/2023|EPI_ISL_19313608|A_/_H5N1|Original||2.3.4.4b|09.12.2023||||06.08
.2024|Animal_and_Plant_Health_Agency__APHA_||HA|4|A/Antarctic_Fur_Seal/Sout
h_Georgia_and_the_South_Sandwich_Islan">
<date value="2023.9397260273972" direction="forwards" units="years"/>
</taxon>
<taxon id="A/Antarctic_Tern/South_Georgia_and_the_South_Sandwich_Islands/40
/2023|EPI_ISL_19313616|A_/_H5N1|Original||2.3.4.4b|06.12.2023||||06.08.2024
|Animal_and_Plant_Health_Agency__APHA_||HA|4|A/Antarctic_Tern/South_Georgia
_and_the_South_Sandwich_Islands/40/202">
<date value="2023.9315068493152" direction="forwards" units="years"/>
</taxon>
<taxon id="A/Antarctic_tern/King_Edward_Point/141271/2023|EPI_ISL_18742220|
A_/_H5N1|Clinical_Sample||2.3.4.4b|06.12.2023|Mollett_Benjamin|||25.06.2024
|Animal_and_Plant_Health_Agency__APHA_|Animal_and_Plant_Health_Agency__APHA
_|HA|4|A/Antarctic_tern/King_Edward_P">
<date value="2023.9315068493152" direction="forwards" units="years"/>
</taxon>
<taxon id="A/Antofagasta/25945/2023|EPI_ISL_19391463|A_/_H5N1|Original||2.3
.4.4b|24.03.2023||||09.09.2024|Instituto_de_Salud_Publica_de_Chile||HA|4|A/
Antofagasta/25945/2023_HA|EPI3549652|DNA_IN">
<date value="2023.1424657534246" direction="forwards" units="years"/>
</taxon>
<taxon id="A/Avian/Argentina/1762-
2/2023|EPI_ISL_18698516|A_/_H5N1|||2.3.4.4b|21.04.2023|Benedetti_Estefania|
||28.12.2023|Direccion_del_Laboratorio_Animal_Direccion_General_de_Laborato
rios_y_Control_Tecnico_SENASA.|Instituto_Nacional_de_Enfermedades_Infeccios
a">
<date value="2023.304109589041" direction="forwards" units="years"/>
</taxon>
<taxon id="A/Avian/Argentina/1790-
5/2023|EPI_ISL_18698517|A_/_H5N1|||2.3.4.4b|24.04.2023|Benedetti_Estefania|
||28.12.2023|Direccion_del_Laboratorio_Animal_Direccion_General_de_Laborato
rios_y_Control_Tecnico_SENASA.|Instituto_Nacional_de_Enfermedades_Infeccios
a">
<date value="2023.3123287671233" direction="forwards" units="years"/>
</taxon>

<taxon id="A/Avian/Argentina/579-
5/2023|EPI_ISL_18698485|A_/_H5N1|||2.3.4.4b|24.02.2023|Benedetti_Estefania|
||28.12.2023|Direccion_del_Laboratorio_Animal_Direccion_General_de_Laborato
rios_y_Control_Tecnico_SENASA.|Instituto_Nacional_de_Enfermedades_Infeccios
as">
<date value="2023.150684931507" direction="forwards" units="years"/>
</taxon>
<taxon id="A/Avian/Argentina/586-
4/2023|EPI_ISL_18698487|A_/_H5N1|||2.3.4.4b|24.02.2023|Benedetti_Estefania|
||28.12.2023|Direccion_del_Laboratorio_Animal_Direccion_General_de_Laborato
rios_y_Control_Tecnico_SENASA.|Instituto_Nacional_de_Enfermedades_Infeccios
as">
<date value="2023.150684931507" direction="forwards" units="years"/>
</taxon>
<taxon id="A/Band-tailed_gull/Antofagasta/228525-
1/2022|EPI_ISL_19131166|A_/_H5N1|Original||2.3.4.4b|19.12.2022||||10.05.202
4|Emory_University__CEIRR_|Emory_University__CEIRR_|HA|4|A/Band-
tailed_gull/Antofagasta/228525-1/2022_HA|EPI3276855|DNA_IN">
<date value="2022.9671232876713" direction="forwards" units="years"/>
</taxon>
<taxon id="A/Band-tailed_gull/Arica_y_Parinacota/232200-
1/2023|EPI_ISL_19131168|A_/_H5N1|Original||2.3.4.4b|11.01.2023||||10.05.202
4|Emory_University__CEIRR_|Emory_University__CEIRR_|HA|4|A/Band-
tailed_gull/Arica_y_Parinacota/232200-1/2023_HA|EPI3276870|DNA_IN">
<date value="2023.0301369863014" direction="forwards" units="years"/>
</taxon>
<taxon id="A/Barn_Owl/BC/AIVPHL-
1011/2023|EPI_ISL_18665563|A_/_H5N1|Original||2.3.4.4b|20.03.2023|Caleta_Je
ssica_Marija|||21.12.2023|B.C._Centre_for_Disease_Control|Public_Health_Age
ncy_of_Canada|HA|4|A/Barn_Owl/BC/AIVPHL-1011/2023_HA|EPI2856439|DNA_INS">
<date value="2023.131506849315" direction="forwards" units="years"/>
</taxon>
<taxon id="A/Barn_Owl/BC/AIVPHL-
926/2023|EPI_ISL_18665504|A_/_H5N1|Original||2.3.4.4b|30.01.2023|Caleta_Jes
sica_Marija|||21.12.2023|B.C._Centre_for_Disease_Control|Public_Health_Agen
cy_of_Canada|HA|4|A/Barn_Owl/BC/AIVPHL-926/2023_HA|EPI2855997|DNA_INS">
<date value="2023.0821917808219" direction="forwards" units="years"/>
</taxon>
<taxon id="A/Barn_Owl/BC/AIVPHL-
995/2023|EPI_ISL_18665547|A_/_H5N1|Original||2.3.4.4b|13.03.2023|Caleta_Jes
sica_Marija|||21.12.2023|B.C._Centre_for_Disease_Control|Public_Health_Agen
cy_of_Canada|HA|4|A/Barn_Owl/BC/AIVPHL-995/2023_HA|EPI2856323|DNA_INS">
<date value="2023.1123287671232" direction="forwards" units="years"/>
</taxon>
<taxon id="A/Barred_Owl/BC/AIVPHL-
1008/2023|EPI_ISL_18665556|A_/_H5N1|Original||2.3.4.4b|20.03.2023|Caleta_Je
ssica_Marija|||21.12.2023|B.C._Centre_for_Disease_Control|Public_Health_Age
ncy_of_Canada|HA|4|A/Barred_Owl/BC/AIVPHL-1008/2023_HA|EPI2856390|DNA_INS">
<date value="2023.131506849315" direction="forwards" units="years"/>
</taxon>
<taxon id="A/Barred_Owl/BC/AIVPHL-
982/2023|EPI_ISL_18665541|A_/_H5N1|Original||2.3.4.4b|10.03.2023|Caleta_Jes
sica_Marija|||21.12.2023|B.C._Centre_for_Disease_Control|Public_Health_Agen
cy_of_Canada|HA|4|A/Barred_Owl/BC/AIVPHL-982/2023_HA|EPI2856275|DNA_INS">
<date value="2023.1041095890412" direction="forwards" units="years"/>
</taxon>
<taxon id="A/Barred_Owl/BC/FAV-0053-
23/2022|EPI_ISL_19155098|A_/_H5N1|||2.3.4.4b|13.12.2022|Signore_Anthony|||2
1.05.2024|Canadian_Food_Inspection_Agency|Canadian_Food_Inspection_Agency|H
A|4|A/Barred_Owl/BC/FAV-0053-23/2022_HA|EPI3315464|DNA_IN">
<date value="2022.9506849315069" direction="forwards" units="years"/>

</taxon>
<taxon id="A/Black-crowned_night-heron/Antofagasta/228705-2/2022|EPI_ISL_18005786|A_/_H5N1|Original||2.3.4.4b|20.12.2022|GISAID_EpiFlu_Data_Curator|||19.07.2023|CEIRS_Data_Processing_and_Coordinating_Center_Center_for_Research_on_Influenza_Pathogenesis__CRIP_|C">
<date value="2022.9698630136986" direction="forwards" units="years"/>
</taxon>
<taxon id="A/Black_Skimmer/Maule/240379/2023|EPI_ISL_17885849|A_/_H5N1|Original||2.3.4.4b|06.03.2023|GISAID_EpiFlu_Data_Curator|||19.07.2023|CEIRS_Data_Processing_and_Coordinating_Center_Center_for_Research_on_Influenza_Pathogenesis__CRIP_|CEIRS_Data_Processing">
<date value="2023.0931506849315" direction="forwards" units="years"/>
</taxon>
<taxon id="A/Blackish_oystercatcher/OHiggins/240628/2023|EPI_ISL_17885967|A_/_H5N1|Original||2.3.4.4b|06.03.2023|GISAID_EpiFlu_Data_Curator|||24.07.2023|CEIRS_Data_Processing_and_Coordinating_Center_Center_for_Research_on_Influenza_Pathogenesis__CRIP_|CEIRS_Dat">
<date value="2023.0931506849315" direction="forwards" units="years"/>
</taxon>
<taxon id="A/Broiler_Breeders_Chicken/BC/FAV-1616-1/2022|EPI_ISL_19155042|A_/_H5N1|||2.3.4.4b|05.12.2022|Signore_Anthony|||21.05.2024|Canadian_Food_Inspection_Agency|Canadian_Food_Inspection_Agency|HA|4|A/Broiler_Breeders_Chicken/BC/FAV-1616-1/2022_HA|EPI3315016">
<date value="2022.9287671232876" direction="forwards" units="years"/>
</taxon>
<taxon id="A/Broiler_Breeders_Chicken/BC/FAV-1616-2/2022|EPI_ISL_19155043|A_/_H5N1|||2.3.4.4b|05.12.2022|Signore_Anthony|||21.05.2024|Canadian_Food_Inspection_Agency|Canadian_Food_Inspection_Agency|HA|4|A/Broiler_Breeders_Chicken/BC/FAV-1616-2/2022_HA|EPI3315024">
<date value="2022.9287671232876" direction="forwards" units="years"/>
</taxon>
<taxon id="A/Brown_Skua/Bird_Island/141232/2023|EPI_ISL_18742214|A_/_H5N1|Clinical_Sample||2.3.4.4b|11.11.2023|Mollett_Benjamin|||25.06.2024|Animal_and_Plant_Health_Agency__APHA_|Animal_and_Plant_Health_Agency__APHA_|HA|4|A/Brown_Skua/Bird_Island/141232/2023|HA|">
<date value="2023.86301369863" direction="forwards" units="years"/>
</taxon>
<taxon id="A/Brown_Skua/Hound_Bay/133949/2023|EPI_ISL_18592429|A_/_H5N1|Egg_Passage_1||2.3.4.4b|30.10.2023|Mollett_Benjamin|||06.12.2023|Animal_and_Plant_Health_Agency__APHA_|Animal_and_Plant_Health_Agency__APHA_|HA|4|A/Brown_Skua/Hound_Bay/133949/2023|HA|EPI281">
<date value="2023.7753424657535" direction="forwards" units="years"/>
</taxon>
<taxon id="A/Brown_Skua/Moltke_Harbour/133752/2023|EPI_ISL_18592424|A_/_H5N1|Clinical_Sample||2.3.4.4b|31.10.2023|Mollett_Benjamin|||06.12.2023|Animal_and_Plant_Health_Agency__APHA_|Animal_and_Plant_Health_Agency__APHA_|HA|4|A/Brown_Skua/Moltke_Harbour/133752/20">
<date value="2023.7780821917809" direction="forwards" units="years"/>
</taxon>
<taxon id="A/Brown_Skua/Moltke_Harbour/133755/2023|EPI_ISL_18592426|A_/_H5N1|Clinical_Sample||2.3.4.4b|31.10.2023|Mollett_Benjamin|||06.12.2023|Animal_and_Plant_Health_Agency__APHA_|Animal_and_Plant_Health_Agency__APHA_|HA|4|A/Brown_Skua/Moltke_Harbour/133755/20">
<date value="2023.7780821917809" direction="forwards" units="years"/>
</taxon>
<taxon id="A/Brown_Skua/Penguin_River/141236/2023|EPI_ISL_18742216|A_/_H5N1|Clinical_Sample||2.3.4.4b|08.11.2023|Mollett_Benjamin|||25.06.2024|Animal_and_Plant_Health_Agency__APHA_|Animal_and_Plant_Health_Agency__APHA_|HA|4|A/Brown_Skua/Penguin_River/141236/2023">
<date value="2023.854794520548" direction="forwards" units="years"/>
</taxon>

<taxon id="A/Brown_Skua/Penguin_River/141240/2023|EPI_ISL_18742218|A_/_H5N1
|Clinical_Sample||2.3.4.4b|08.11.2023|Mollett_Benjamin|||25.06.2024|Animal_
and_Plant_Health_Agency__APHA_|Animal_and_Plant_Health_Agency__APHA_|HA|4|A
/Brown_Skua/Penguin_River/141240/2023">
<date value="2023.854794520548" direction="forwards" units="years"/>
</taxon>
<taxon id="A/Brown_Skua/South_Georgia_and_the_South_Sandwich_Islands/104/20
23|EPI_ISL_19313621|A_/_H5N1|Original||2.3.4.4b|15.11.2023||||06.08.2024|An
imal_and_Plant_Health_Agency__APHA_||HA|4|A/Brown_Skua/South_Georgia_and_th
e_South_Sandwich_Islands/104/2023_HA|E">
<date value="2023.8739726027397" direction="forwards" units="years"/>
</taxon>
<taxon id="A/Brown_Skua/South_Georgia_and_the_South_Sandwich_Islands/112/20
23|EPI_ISL_19313575|A_/_H5N1|Original||2.3.4.4b|30.10.2023||||06.08.2024|An
imal_and_Plant_Health_Agency__APHA_||HA|4|A/Brown_Skua/South_Georgia_and_th
e_South_Sandwich_Islands/112/2023_HA|E">
<date value="2023.7753424657535" direction="forwards" units="years"/>
</taxon>
<taxon id="A/Brown_Skua/South_Georgia_and_the_South_Sandwich_Islands/144/20
23|EPI_ISL_19313583|A_/_H5N1|Original||2.3.4.4b|11.11.2023||||06.08.2024|An
imal_and_Plant_Health_Agency__APHA_||HA|4|A/Brown_Skua/South_Georgia_and_th
e_South_Sandwich_Islands/144/2023_HA|E">
<date value="2023.86301369863" direction="forwards" units="years"/>
</taxon>
<taxon id="A/Brown_Skua/South_Georgia_and_the_South_Sandwich_Islands/16/202
3|EPI_ISL_19321280|A_/_H5N1|Original||2.3.4.4b|08.10.2023||||08.08.2024|Ani
mal_and_Plant_Health_Agency__APHA_||HA|4|A/Brown_Skua/South_Georgia_and_the
_South_Sandwich_Islands/16/2023_HA|EPI">
<date value="2023.7150684931507" direction="forwards" units="years"/>
</taxon>
<taxon id="A/Brown_Skua/South_Georgia_and_the_South_Sandwich_Islands/24/202
3|EPI_ISL_19321271|A_/_H5N1|Original||2.3.4.4b|08.10.2023||||08.08.2024|Ani
mal_and_Plant_Health_Agency__APHA_||HA|4|A/Brown_Skua/South_Georgia_and_the
_South_Sandwich_Islands/24/2023_HA|EPI">
<date value="2023.7150684931507" direction="forwards" units="years"/>
</taxon>
<taxon id="A/Brown_Skua/South_Georgia_and_the_South_Sandwich_Islands/48/202
3|EPI_ISL_19313591|A_/_H5N1|Original||2.3.4.4b|31.10.2023||||06.08.2024|Ani
mal_and_Plant_Health_Agency__APHA_||HA|4|A/Brown_Skua/South_Georgia_and_the
_South_Sandwich_Islands/48/2023_HA|EPI">
<date value="2023.7780821917809" direction="forwards" units="years"/>
</taxon>
<taxon id="A/Brown_Skua/South_Georgia_and_the_South_Sandwich_Islands/64/202
3|EPI_ISL_19313626|A_/_H5N1|Original||2.3.4.4b|31.10.2023||||06.08.2024|Ani
mal_and_Plant_Health_Agency__APHA_||HA|4|A/Brown_Skua/South_Georgia_and_the
_South_Sandwich_Islands/64/2023_HA|EPI">
<date value="2023.7780821917809" direction="forwards" units="years"/>
</taxon>
<taxon id="A/Brown_Skua/South_Georgia_and_the_South_Sandwich_Islands/8/2023
|EPI_ISL_19321287|A_/_H5N1|Original||2.3.4.4b|08.10.2023||||08.08.2024|Anim
al_and_Plant_Health_Agency__APHA_||HA|4|A/Brown_Skua/South_Georgia_and_the_
South_Sandwich_Islands/8/2023_HA|EPI34">
<date value="2023.7150684931507" direction="forwards" units="years"/>
</taxon>
<taxon id="A/Brown_Skua/South_Georgia_and_the_South_Sandwich_Islands/80/202
3|EPI_ISL_19313660|A_/_H5N1|Original||2.3.4.4b|08.11.2023||||06.08.2024|Ani
mal_and_Plant_Health_Agency__APHA_||HA|4|A/Brown_Skua/South_Georgia_and_the
_South_Sandwich_Islands/80/2023_HA|EPI">
<date value="2023.854794520548" direction="forwards" units="years"/>
</taxon>

<taxon id="A/Brown_Skua/South_Georgia_and_the_South_Sandwich_Islands/96/202
3|EPI_ISL_19313668|A_/_H5N1|Original||2.3.4.4b|30.10.2023||||06.08.2024|Ani
mal_and_Plant_Health_Agency__APHA_||HA|4|A/Brown_Skua/South_Georgia_and_the
_South_Sandwich_Islands/96/2023_HA|EPI">
<date value="2023.7753424657535" direction="forwards" units="years"/>
</taxon>
<taxon id="A/Brown_skua/Bird_Island/128287/2023|EPI_ISL_18439562|A_/_H5N1|C
linical_Sample||2.3.4.4b|08.10.2023|Byrne_Alex|||27.10.2023|Animal_and_Plan
t_Health_Agency__APHA_|Animal_and_Plant_Health_Agency__APHA_|HA|4|A/Brown_s
kua/Bird_Island/128287/2023|HA|EPI278">
<date value="2023.7150684931507" direction="forwards" units="years"/>
</taxon>
<taxon id="A/Brown_skua/Bird_Island/128288/2023|EPI_ISL_18439563|A_/_H5N1|C
linical_Sample||2.3.4.4b|08.10.2023|Byrne_Alex|||27.10.2023|Animal_and_Plan
t_Health_Agency__APHA_|Animal_and_Plant_Health_Agency__APHA_|HA|4|A/Brown_s
kua/Bird_Island/128288/2023|HA|EPI278">
<date value="2023.7150684931507" direction="forwards" units="years"/>
</taxon>
<taxon id="A/Brown_skua/Bird_Island/128289/2023|EPI_ISL_18439564|A_/_H5N1|C
linical_Sample||2.3.4.4b|08.10.2023|Byrne_Alex|||27.10.2023|Animal_and_Plan
t_Health_Agency__APHA_|Animal_and_Plant_Health_Agency__APHA_|HA|4|A/Brown_s
kua/Bird_Island/128289/2023|HA|EPI278">
<date value="2023.7150684931507" direction="forwards" units="years"/>
</taxon>
<taxon id="A/Cackling_Goose/BC/AIVPHL-
875/2023|EPI_ISL_18665568|A_/_H5N1|Original||2.3.4.4b|09.01.2023|Caleta_Jes
sica_Marija|||21.12.2023|B.C._Centre_for_Disease_Control|Public_Health_Agen
cy_of_Canada|HA|4|A/Cackling_Goose/BC/AIVPHL-
875/2023_HA|EPI2856470|DNA_IN">
<date value="2023.0246575342467" direction="forwards" units="years"/>
</taxon>
<taxon id="A/Cackling_Goose/BC/AIVPHL-
876/2023|EPI_ISL_18665571|A_/_H5N1|Original||2.3.4.4b|09.01.2023|Caleta_Jes
sica_Marija|||21.12.2023|B.C._Centre_for_Disease_Control|Public_Health_Agen
cy_of_Canada|HA|4|A/Cackling_Goose/BC/AIVPHL-
876/2023_HA|EPI2856496|DNA_IN">
<date value="2023.0246575342467" direction="forwards" units="years"/>
</taxon>
<taxon id="A/Calidris_alba/Lima/HA_4/2023|EPI_ISL_19158339|A_/_H5N1|Origina
l||2.3.4.4b|20.04.2023||||22.05.2024|National_University_of_San_Marcos_Biol
ogy_Sciences||HA|4|A/Calidris_alba/Lima/HA_4/2023_HA|EPI3324598|DNA_IN">
<date value="2023.3013698630136" direction="forwards" units="years"/>
</taxon>
<taxon id="A/Calidris_alba/Lima/Villa01/2023___this_|EPI_ISL_19070288|A_/_H
5N1|||2.3.4.4b|20.04.2023|Landazabal_Castillo_Sandra_Liliana|||24.04.2024|M
olecular_and_Clinical_Virology_Laboratory_National_University_of_San_Marcos
|National_University_of_San_Marcos|HA">
<date value="2023.3013698630136" direction="forwards" units="years"/>
</taxon>
<taxon id="A/Canada_Goose/BC/AIVPHL-
844/2022|EPI_ISL_18665543|A_/_H5N1|Original||2.3.4.4b|16.12.2022|Caleta_Jes
sica_Marija|||21.12.2023|B.C._Centre_for_Disease_Control|Public_Health_Agen
cy_of_Canada|HA|4|A/Canada_Goose/BC/AIVPHL-844/2022_HA|EPI2856282|DNA_INS">
<date value="2022.958904109589" direction="forwards" units="years"/>
</taxon>
<taxon id="A/Canada_goose/Iowa/23-028688-001-
original/2023|EPI_ISL_19371749|A_/_H5N1|original||2.3.4.4b|13.01.2023|Killi
an_Mary_Lea||23-028688-
001|31.08.2024|National_Veterinary_Services_Laboratories_-
_USDA|National_Veterinary_Services_Laboratories_-_USDA|HA|4|H">
<date value="2023.035616438356" direction="forwards" units="years"/>

</taxon>
<taxon id="A/Canada_goose/Iowa/23-030290-001/2022|EPI_ISL_19131437|A_/_H5N1|Original||2.3.4.4b|07.12.2022|Killian_Mary_Lea||23-030290-001|12.12.2024|National_Veterinary_Services_Laboratories_-_USDA|National_Veterinary_Services_Laboratories_-_USDA|HA|4|HA_A/Canad">
<date value="2022.9342465753425" direction="forwards" units="years"/>
</taxon>
<taxon id="A/Canada_goose/Washington/23-007799-001/2023|EPI_ISL_17964894|A_/_H5N1|Original||2.3.4.4b|27.02.2023|Killian_Mary_Lea||23-007799-001|07.07.2023|National_Veterinary_Services_Laboratories_-_USDA|National_Veterinary_Services_Laboratories_-_USDA|HA|4|HA_A">
<date value="2023.158904109589" direction="forwards" units="years"/>
</taxon>
<taxon id="A/Caspian_tern/Oregon/23-018836-001/2023|EPI_ISL_18311014|A_/_H5N1|Original||2.3.4.4b|15.06.2023|Killian_Mary_Lea||23-018836-001|26.09.2023|National_Veterinary_Services_Laboratories_-_USDA|National_Veterinary_Services_Laboratories_-_USDA|HA|4|HA_A/Cas">
<date value="2023.454794520548" direction="forwards" units="years"/>
</taxon>
<taxon id="A/Caspian_tern/Oregon/23-018836-002/2023|EPI_ISL_18311015|A_/_H5N1|Original||2.3.4.4b|15.06.2023|Killian_Mary_Lea||23-018836-002|26.09.2023|National_Veterinary_Services_Laboratories_-_USDA|National_Veterinary_Services_Laboratories_-_USDA|HA|4|HA_A/Cas">
<date value="2023.454794520548" direction="forwards" units="years"/>
</taxon>
<taxon id="A/Caspian_tern/Oregon/23-018836-003/2023|EPI_ISL_18311016|A_/_H5N1|Original||2.3.4.4b|15.06.2023|Killian_Mary_Lea||23-018836-003|26.09.2023|National_Veterinary_Services_Laboratories_-_USDA|National_Veterinary_Services_Laboratories_-_USDA|HA|4|HA_A/Cas">
<date value="2023.454794520548" direction="forwards" units="years"/>
</taxon>
<taxon id="A/Caspian_tern/Washington/23-021119-003/2023|EPI_ISL_18311017|A_/_H5N1|Original||2.3.4.4b|11.07.2023|Killian_Mary_Lea||23-021119-003|26.09.2023|National_Veterinary_Services_Laboratories_-_USDA|National_Veterinary_Services_Laboratories_-_USDA|HA|4|HA_A">
<date value="2023.5260273972603" direction="forwards" units="years"/>
</taxon>
<taxon id="A/Caspian_tern/Washington/23-023703-001/2023|EPI_ISL_18311018|A_/_H5N1|Original||2.3.4.4b|31.07.2023|Killian_Mary_Lea||23-023703-001|26.09.2023|National_Veterinary_Services_Laboratories_-_USDA|National_Veterinary_Services_Laboratories_-_USDA|HA|4|HA_A">
<date value="2023.5808219178082" direction="forwards" units="years"/>
</taxon>
<taxon id="A/Caspian_tern/Washington/23-023703-002/2023|EPI_ISL_18311019|A_/_H5N1|Original||2.3.4.4b|31.07.2023|Killian_Mary_Lea||23-023703-002|26.09.2023|National_Veterinary_Services_Laboratories_-_USDA|National_Veterinary_Services_Laboratories_-_USDA|HA|4|HA_A">
<date value="2023.5808219178082" direction="forwards" units="years"/>
</taxon>
<taxon id="A/Caspian_tern/Washington/W231930045-3/2023|EPI_ISL_19592591|A_/_H5N1|Original||2.3.4.4b|10.07.2023||||06.12.2024|Washington_State_University||HA|4|A/Caspian_tern/Washington/W231930045-3/2023_HA|EPI3675293|DNA_IN">

<date value="2023.5232876712328" direction="forwards" units="years"/>
</taxon>
<taxon id="A/Caspian_tern/Washington/W232140074-1/2023|EPI_ISL_19592592|A_/_H5N1|Original||2.3.4.4b|31.07.2023||||06.12.2024|Washington_State_University||HA|4|A/Caspian_tern/Washington/W232140074-1/2023_HA|EPI3675301|DNA_IN">
<date value="2023.5808219178082" direction="forwards" units="years"/>
</taxon>
<taxon id="A/Caspian_tern/Washington/W232140074-2/2023|EPI_ISL_19592593|A_/_H5N1|Original||2.3.4.4b|31.07.2023||||06.12.2024|Washington_State_University||HA|4|A/Caspian_tern/Washington/W232140074-2/2023_HA|EPI3675309|DNA_IN">
<date value="2023.5808219178082" direction="forwards" units="years"/>
</taxon>
<taxon id="A/Caspian_tern/Washington/W232270041-1/2023|EPI_ISL_19592594|A_/_H5N1|Original||2.3.4.4b|11.08.2023||||06.12.2024|Washington_State_University||HA|4|A/Caspian_tern/Washington/W232270041-1/2023_HA|EPI3675317|DNA_IN">
<date value="2023.6109589041096" direction="forwards" units="years"/>
</taxon>
<taxon id="A/Chicken/Argentina/1035-1/2023|EPI_ISL_18698504|A_/_H5N1|||2.3.4.4b|16.03.2023|Benedetti_Estefania|||28.12.2023|Direccion_del_Laboratorio_Animal_Direccion_General_de_Laboratorios_y_Control_Tecnico_SENASA.|Instituto_Nacional_de_Enfermedades_Infeccio">
<date value="2023.1205479452055" direction="forwards" units="years"/>
</taxon>
<taxon id="A/Chicken/Argentina/1147-2/2023|EPI_ISL_18698505|A_/_H5N1|||2.3.4.4b|21.03.2023|Benedetti_Estefania|||28.12.2023|Direccion_del_Laboratorio_Animal_Direccion_General_de_Laboratorios_y_Control_Tecnico_SENASA.|Instituto_Nacional_de_Enfermedades_Infeccio">
<date value="2023.1342465753426" direction="forwards" units="years"/>
</taxon>
<taxon id="A/Chicken/Argentina/1200-1/2023|EPI_ISL_18698506|A_/_H5N1|||2.3.4.4b|27.03.2023|Benedetti_Estefania|||28.12.2023|Direccion_del_Laboratorio_Animal_Direccion_General_de_Laboratorios_y_Control_Tecnico_SENASA.|Instituto_Nacional_de_Enfermedades_Infeccio">
<date value="2023.150684931507" direction="forwards" units="years"/>
</taxon>
<taxon id="A/Chicken/Argentina/1340-2/2023|EPI_ISL_18698507|A_/_H5N1|||2.3.4.4b|31.03.2023|Benedetti_Estefania|||28.12.2023|Direccion_del_Laboratorio_Animal_Direccion_General_de_Laboratorios_y_Control_Tecnico_SENASA.|Instituto_Nacional_de_Enfermedades_Infeccio">
<date value="2023.1616438356164" direction="forwards" units="years"/>
</taxon>
<taxon id="A/Chicken/Argentina/1375-6/2023|EPI_ISL_18698509|A_/_H5N1|||2.3.4.4b|03.04.2023|Benedetti_Estefania|||28.12.2023|Direccion_del_Laboratorio_Animal_Direccion_General_de_Laboratorios_y_Control_Tecnico_SENASA.|Instituto_Nacional_de_Enfermedades_Infeccio">
<date value="2023.2547945205479" direction="forwards" units="years"/>
</taxon>
<taxon id="A/Chicken/Argentina/1416-3/2023|EPI_ISL_18698510|A_/_H5N1|||2.3.4.4b|03.04.2023|Benedetti_Estefania|||28.12.2023|Direccion_del_Laboratorio_Animal_Direccion_General_de_Laboratorios_y_Control_Tecnico_SENASA.|Instituto_Nacional_de_Enfermedades_Infeccio">
<date value="2023.2547945205479" direction="forwards" units="years"/>

</taxon>
<taxon id="A/Chicken/Argentina/1495-4/2023|EPI_ISL_18698719|A_/_H5N1|||2.3.4.4b|10.04.2023|Benedetti_Estefania|||28.12.2023|Direccion_del_Laboratorio_Animal_Direccion_General_de_Laboratorios_y_Control_Tecnico_SENASA.|Instituto_Nacional_de_Enfermedades_Infeccio">
<date value="2023.2739726027398" direction="forwards" units="years"/>
</taxon>
<taxon id="A/Chicken/Argentina/1530-3/2023|EPI_ISL_18698511|A_/_H5N1|||2.3.4.4b|12.04.2023|Benedetti_Estefania|||28.12.2023|Direccion_del_Laboratorio_Animal_Direccion_General_de_Laboratorios_y_Control_Tecnico_SENASA.|Instituto_Nacional_de_Enfermedades_Infeccio">
<date value="2023.2794520547945" direction="forwards" units="years"/>
</taxon>
<taxon id="A/Chicken/Argentina/1708-1/2023|EPI_ISL_18698512|A_/_H5N1|||2.3.4.4b|19.04.2023|Benedetti_Estefania|||28.12.2023|Direccion_del_Laboratorio_Animal_Direccion_General_de_Laboratorios_y_Control_Tecnico_SENASA.|Instituto_Nacional_de_Enfermedades_Infeccio">
<date value="2023.2986301369863" direction="forwards" units="years"/>
</taxon>
<taxon id="A/Chicken/Argentina/1976-2/2023|EPI_ISL_18698518|A_/_H5N1|||2.3.4.4b|01.05.2023|Benedetti_Estefania|||28.12.2023|Direccion_del_Laboratorio_Animal_Direccion_General_de_Laboratorios_y_Control_Tecnico_SENASA.|Instituto_Nacional_de_Enfermedades_Infeccio">
<date value="2023.331506849315" direction="forwards" units="years"/>
</taxon>
<taxon id="A/Chicken/Argentina/1984-4/2023|EPI_ISL_18698519|A_/_H5N1|||2.3.4.4b|04.05.2023|Benedetti_Estefania|||28.12.2023|Direccion_del_Laboratorio_Animal_Direccion_General_de_Laboratorios_y_Control_Tecnico_SENASA.|Instituto_Nacional_de_Enfermedades_Infeccio">
<date value="2023.3397260273973" direction="forwards" units="years"/>
</taxon>
<taxon id="A/Chicken/Argentina/2016-2/2023|EPI_ISL_18698732|A_/_H5N1|||2.3.4.4b|03.05.2023|Benedetti_Estefania|||28.12.2023|Direccion_del_Laboratorio_Animal_Direccion_General_de_Laboratorios_y_Control_Tecnico_SENASA.|Instituto_Nacional_de_Enfermedades_Infeccio">
<date value="2023.33698630137" direction="forwards" units="years"/>
</taxon>
<taxon id="A/Chicken/Argentina/2034-5/2023|EPI_ISL_18698520|A_/_H5N1|||2.3.4.4b|05.05.2023|Benedetti_Estefania|||28.12.2023|Direccion_del_Laboratorio_Animal_Direccion_General_de_Laboratorios_y_Control_Tecnico_SENASA.|Instituto_Nacional_de_Enfermedades_Infeccio">
<date value="2023.3424657534247" direction="forwards" units="years"/>
</taxon>
<taxon id="A/Chicken/Argentina/2049-3/2023|EPI_ISL_18698521|A_/_H5N1|||2.3.4.4b|08.05.2023|Benedetti_Estefania|||28.12.2023|Direccion_del_Laboratorio_Animal_Direccion_General_de_Laboratorios_y_Control_Tecnico_SENASA.|Instituto_Nacional_de_Enfermedades_Infeccio">
<date value="2023.3506849315067" direction="forwards" units="years"/>
</taxon>
<taxon id="A/Chicken/Argentina/2064-3/2023|EPI_ISL_18698522|A_/_H5N1|||2.3.4.4b|08.05.2023|Benedetti_Estefania|||28.12.2023|Direccion_del_Laboratorio_Animal_Direccion_General_de_Laborato

rios_y_Control_Tecnico_SENASA.|Instituto_Nacional_de_Enfermedades_Infeccio"
>
<date value="2023.3506849315067" direction="forwards" units="years"/>
</taxon>
<taxon id="A/Chicken/Argentina/2305-
1/2023|EPI_ISL_18698524|A_/_H5N1|||2.3.4.4b|18.05.2023|Benedetti_Estefania|
||28.12.2023|Direccion_del_Laboratorio_Animal_Direccion_General_de_Laborato
rios_y_Control_Tecnico_SENASA.|Instituto_Nacional_de_Enfermedades_Infeccio"
>
<date value="2023.3780821917808" direction="forwards" units="years"/>
</taxon>
<taxon id="A/Chicken/Argentina/2305-
6/2023|EPI_ISL_18698525|A_/_H5N1|||2.3.4.4b|18.05.2023|Benedetti_Estefania|
||28.12.2023|Direccion_del_Laboratorio_Animal_Direccion_General_de_Laborato
rios_y_Control_Tecnico_SENASA.|Instituto_Nacional_de_Enfermedades_Infeccio"
>
<date value="2023.3780821917808" direction="forwards" units="years"/>
</taxon>
<taxon id="A/Chicken/Argentina/2483-
3/2023|EPI_ISL_18698526|A_/_H5N1|||2.3.4.4b|29.05.2023|Benedetti_Estefania|
||28.12.2023|Direccion_del_Laboratorio_Animal_Direccion_General_de_Laborato
rios_y_Control_Tecnico_SENASA.|Instituto_Nacional_de_Enfermedades_Infeccio"
>
<date value="2023.4082191780822" direction="forwards" units="years"/>
</taxon>
<taxon id="A/Chicken/Argentina/2796-
2/2023|EPI_ISL_18698527|A_/_H5N1|||2.3.4.4b|13.06.2023|Benedetti_Estefania|
||28.12.2023|Direccion_del_Laboratorio_Animal_Direccion_General_de_Laborato
rios_y_Control_Tecnico_SENASA.|Instituto_Nacional_de_Enfermedades_Infeccio"
>
<date value="2023.4493150684932" direction="forwards" units="years"/>
</taxon>
<taxon id="A/Chicken/Argentina/3346-
1/2023|EPI_ISL_18698528|A_/_H5N1|||2.3.4.4b|06.07.2023|Benedetti_Estefania|
||28.12.2023|Direccion_del_Laboratorio_Animal_Direccion_General_de_Laborato
rios_y_Control_Tecnico_SENASA.|Instituto_Nacional_de_Enfermedades_Infeccio"
>
<date value="2023.5123287671233" direction="forwards" units="years"/>
</taxon>
<taxon id="A/Chicken/Argentina/3695-
3/2023|EPI_ISL_18698530|A_/_H5N1|||2.3.4.4b|27.07.2023|Benedetti_Estefania|
||28.12.2023|Direccion_del_Laboratorio_Animal_Direccion_General_de_Laborato
rios_y_Control_Tecnico_SENASA.|Instituto_Nacional_de_Enfermedades_Infeccio"
>
<date value="2023.5698630136985" direction="forwards" units="years"/>
</taxon>
<taxon id="A/Chicken/Argentina/464-
4/2023|EPI_ISL_18698460|A_/_H5N1|||2.3.4.4b|16.02.2023|Benedetti_Estefania|
||28.12.2023|Direccion_del_Laboratorio_Animal_Direccion_General_de_Laborato
rios_y_Control_Tecnico_SENASA.|Instituto_Nacional_de_Enfermedades_Infeccios
">
<date value="2023.1287671232876" direction="forwards" units="years"/>
</taxon>
<taxon id="A/Chicken/Argentina/477-
5/2023|EPI_ISL_18698462|A_/_H5N1|||2.3.4.4b|18.02.2023|Benedetti_Estefania|
||28.12.2023|Direccion_del_Laboratorio_Animal_Direccion_General_de_Laborato
rios_y_Control_Tecnico_SENASA.|Instituto_Nacional_de_Enfermedades_Infeccios
">
<date value="2023.1342465753426" direction="forwards" units="years"/>
</taxon>

<taxon id="A/Chicken/Argentina/481-
2/2023|EPI_ISL_18698464|A_/_H5N1|||2.3.4.4b|18.02.2023|Benedetti_Estefania|
||28.12.2023|Direccion_del_Laboratorio_Animal_Direccion_General_de_Laborato
rios_y_Control_Tecnico_SENASA.|Instituto_Nacional_de_Enfermedades_Infeccios
">
<date value="2023.1342465753426" direction="forwards" units="years"/>
</taxon>
<taxon id="A/Chicken/Argentina/485-
6/2023|EPI_ISL_18698466|A_/_H5N1|||2.3.4.4b|19.02.2023|Benedetti_Estefania|
||28.12.2023|Direccion_del_Laboratorio_Animal_Direccion_General_de_Laborato
rios_y_Control_Tecnico_SENASA.|Instituto_Nacional_de_Enfermedades_Infeccios
">
<date value="2023.13698630137" direction="forwards" units="years"/>
</taxon>
<taxon id="A/Chicken/Argentina/491-
2/2023|EPI_ISL_18698469|A_/_H5N1|||2.3.4.4b|20.02.2023|Benedetti_Estefania|
||28.12.2023|Direccion_del_Laboratorio_Animal_Direccion_General_de_Laborato
rios_y_Control_Tecnico_SENASA.|Instituto_Nacional_de_Enfermedades_Infeccios
">
<date value="2023.1397260273973" direction="forwards" units="years"/>
</taxon>
<taxon id="A/Chicken/Argentina/501-
1/2023|EPI_ISL_18698471|A_/_H5N1|||2.3.4.4b|20.02.2023|Benedetti_Estefania|
||28.12.2023|Direccion_del_Laboratorio_Animal_Direccion_General_de_Laborato
rios_y_Control_Tecnico_SENASA.|Instituto_Nacional_de_Enfermedades_Infeccios
">
<date value="2023.1397260273973" direction="forwards" units="years"/>
</taxon>
<taxon id="A/Chicken/Argentina/506-
2/2023|EPI_ISL_18698474|A_/_H5N1|||2.3.4.4b|20.02.2023|Benedetti_Estefania|
||28.12.2023|Direccion_del_Laboratorio_Animal_Direccion_General_de_Laborato
rios_y_Control_Tecnico_SENASA.|Instituto_Nacional_de_Enfermedades_Infeccios
">
<date value="2023.1397260273973" direction="forwards" units="years"/>
</taxon>
<taxon id="A/Chicken/Argentina/509-
2/2023|EPI_ISL_18698476|A_/_H5N1|||2.3.4.4b|21.02.2023|Benedetti_Estefania|
||28.12.2023|Direccion_del_Laboratorio_Animal_Direccion_General_de_Laborato
rios_y_Control_Tecnico_SENASA.|Instituto_Nacional_de_Enfermedades_Infeccios
">
<date value="2023.1424657534246" direction="forwards" units="years"/>
</taxon>
<taxon id="A/Chicken/Argentina/556-
6/2023|EPI_ISL_18698478|A_/_H5N1|||2.3.4.4b|23.02.2023|Benedetti_Estefania|
||28.12.2023|Direccion_del_Laboratorio_Animal_Direccion_General_de_Laborato
rios_y_Control_Tecnico_SENASA.|Instituto_Nacional_de_Enfermedades_Infeccios
">
<date value="2023.1479452054793" direction="forwards" units="years"/>
</taxon>
<taxon id="A/Chicken/Argentina/559-
8/2023|EPI_ISL_18698480|A_/_H5N1|||2.3.4.4b|22.02.2023|Benedetti_Estefania|
||28.12.2023|Direccion_del_Laboratorio_Animal_Direccion_General_de_Laborato
rios_y_Control_Tecnico_SENASA.|Instituto_Nacional_de_Enfermedades_Infeccios
">
<date value="2023.145205479452" direction="forwards" units="years"/>
</taxon>
<taxon id="A/Chicken/Argentina/578-
2/2023|EPI_ISL_18698482|A_/_H5N1|||2.3.4.4b|23.02.2023|Benedetti_Estefania|
||28.12.2023|Direccion_del_Laboratorio_Animal_Direccion_General_de_Laborato
rios_y_Control_Tecnico_SENASA.|Instituto_Nacional_de_Enfermedades_Infeccios
">

<date value="2023.1479452054793" direction="forwards" units="years"/>
</taxon>
<taxon id="A/Chicken/Argentina/588-
4/2023|EPI_ISL_18698490|A_/_H5N1|||2.3.4.4b|24.02.2023|Benedetti_Estefania|
||28.12.2023|Direccion_del_Laboratorio_Animal_Direccion_General_de_Laborato
rios_y_Control_Tecnico_SENASA.|Instituto_Nacional_de_Enfermedades_Infeccios
">
<date value="2023.150684931507" direction="forwards" units="years"/>
</taxon>
<taxon id="A/Chicken/Argentina/606-
1/2023|EPI_ISL_18698492|A_/_H5N1|||2.3.4.4b|27.02.2023|Benedetti_Estefania|
||28.12.2023|Direccion_del_Laboratorio_Animal_Direccion_General_de_Laborato
rios_y_Control_Tecnico_SENASA.|Instituto_Nacional_de_Enfermedades_Infeccios
">
<date value="2023.158904109589" direction="forwards" units="years"/>
</taxon>
<taxon id="A/Chicken/Argentina/736-
1/2023|EPI_ISL_18698494|A_/_H5N1|||2.3.4.4b|03.03.2023|Benedetti_Estefania|
||28.12.2023|Direccion_del_Laboratorio_Animal_Direccion_General_de_Laborato
rios_y_Control_Tecnico_SENASA.|Instituto_Nacional_de_Enfermedades_Infeccios
">
<date value="2023.0849315068492" direction="forwards" units="years"/>
</taxon>
<taxon id="A/Chicken/Argentina/747-
1/2023|EPI_ISL_18698730|A_/_H5N1|||2.3.4.4b|04.03.2023|Benedetti_Estefania|
||28.12.2023|Direccion_del_Laboratorio_Animal_Direccion_General_de_Laborato
rios_y_Control_Tecnico_SENASA.|Instituto_Nacional_de_Enfermedades_Infeccios
">
<date value="2023.0876712328768" direction="forwards" units="years"/>
</taxon>
<taxon id="A/Chicken/Argentina/858-
1/2023|EPI_ISL_18698499|A_/_H5N1|||2.3.4.4b|08.03.2023|Benedetti_Estefania|
||28.12.2023|Direccion_del_Laboratorio_Animal_Direccion_General_de_Laborato
rios_y_Control_Tecnico_SENASA.|Instituto_Nacional_de_Enfermedades_Infeccios
">
<date value="2023.0986301369862" direction="forwards" units="years"/>
</taxon>
<taxon id="A/Chicken/Argentina/895-
1/2023|EPI_ISL_18698731|A_/_H5N1|||2.3.4.4b|09.03.2023|Benedetti_Estefania|
||28.12.2023|Direccion_del_Laboratorio_Animal_Direccion_General_de_Laborato
rios_y_Control_Tecnico_SENASA.|Instituto_Nacional_de_Enfermedades_Infeccios
">
<date value="2023.1013698630136" direction="forwards" units="years"/>
</taxon>
<taxon id="A/Chicken/Argentina/919-
3/2023|EPI_ISL_18698502|A_/_H5N1|||2.3.4.4b|13.03.2023|Benedetti_Estefania|
||28.12.2023|Direccion_del_Laboratorio_Animal_Direccion_General_de_Laborato
rios_y_Control_Tecnico_SENASA.|Instituto_Nacional_de_Enfermedades_Infeccios
">
<date value="2023.1123287671232" direction="forwards" units="years"/>
</taxon>
<taxon id="A/Chicken/BC/FAV-0005-
1/2023|EPI_ISL_19155141|A_/_H5N1|||2.3.4.4b|03.01.2023|Signore_Anthony|||21
.05.2024|Canadian_Food_Inspection_Agency|Canadian_Food_Inspection_Agency|HA
|4|A/Chicken/BC/FAV-0005-1/2023_HA|EPI3315808|DNA_IN">
<date value="2023.0082191780823" direction="forwards" units="years"/>
</taxon>
<taxon id="A/Chicken/BC/FAV-0005-
2/2023|EPI_ISL_19155142|A_/_H5N1|||2.3.4.4b|03.01.2023|Signore_Anthony|||21
.05.2024|Canadian_Food_Inspection_Agency|Canadian_Food_Inspection_Agency|HA
|4|A/Chicken/BC/FAV-0005-2/2023_HA|EPI3315816|DNA_IN">

<date value="2023.0082191780823" direction="forwards" units="years"/>
</taxon>
<taxon id="A/Chicken/BC/FAV-0010-
1/2023|EPI_ISL_19155147|A_/_H5N1|||2.3.4.4b|09.01.2023|Signore_Anthony|||21
.05.2024|Canadian_Food_Inspection_Agency|Canadian_Food_Inspection_Agency|HA
|4|A/Chicken/BC/FAV-0010-1/2023_HA|EPI3315856|DNA_IN">
<date value="2023.0246575342467" direction="forwards" units="years"/>
</taxon>
<taxon id="A/Chicken/BC/FAV-0010-
2/2023|EPI_ISL_19155148|A_/_H5N1|||2.3.4.4b|09.01.2023|Signore_Anthony|||21
.05.2024|Canadian_Food_Inspection_Agency|Canadian_Food_Inspection_Agency|HA
|4|A/Chicken/BC/FAV-0010-2/2023_HA|EPI3315864|DNA_IN">
<date value="2023.0246575342467" direction="forwards" units="years"/>
</taxon>
<taxon id="A/Chicken/BC/FAV-0011-
1/2023|EPI_ISL_19155149|A_/_H5N1|||2.3.4.4b|09.01.2023|Signore_Anthony|||21
.05.2024|Canadian_Food_Inspection_Agency|Canadian_Food_Inspection_Agency|HA
|4|A/Chicken/BC/FAV-0011-1/2023_HA|EPI3315872|DNA_IN">
<date value="2023.0246575342467" direction="forwards" units="years"/>
</taxon>
<taxon id="A/Chicken/BC/FAV-0011-
2/2023|EPI_ISL_19155150|A_/_H5N1|||2.3.4.4b|09.01.2023|Signore_Anthony|||21
.05.2024|Canadian_Food_Inspection_Agency|Canadian_Food_Inspection_Agency|HA
|4|A/Chicken/BC/FAV-0011-2/2023_HA|EPI3315880|DNA_IN">
<date value="2023.0246575342467" direction="forwards" units="years"/>
</taxon>
<taxon id="A/Chicken/BC/FAV-1617-
1/2022|EPI_ISL_19155051|A_/_H5N1|||2.3.4.4b|05.12.2022|Signore_Anthony|||21
.05.2024|Canadian_Food_Inspection_Agency|Canadian_Food_Inspection_Agency|HA
|4|A/Chicken/BC/FAV-1617-1/2022_HA|EPI3315088|DNA_IN">
<date value="2022.9287671232876" direction="forwards" units="years"/>
</taxon>
<taxon id="A/Chicken/BC/FAV-1617-
2/2022|EPI_ISL_19155052|A_/_H5N1|||2.3.4.4b|05.12.2022|Signore_Anthony|||21
.05.2024|Canadian_Food_Inspection_Agency|Canadian_Food_Inspection_Agency|HA
|4|A/Chicken/BC/FAV-1617-2/2022_HA|EPI3315096|DNA_IN">
<date value="2022.9287671232876" direction="forwards" units="years"/>
</taxon>
<taxon id="A/Chicken/BC/FAV-1630-
1/2022|EPI_ISL_19155066|A_/_H5N1|||2.3.4.4b|07.12.2022|Signore_Anthony|||21
.05.2024|Canadian_Food_Inspection_Agency|Canadian_Food_Inspection_Agency|HA
|4|A/Chicken/BC/FAV-1630-1/2022_HA|EPI3315208|DNA_IN">
<date value="2022.9342465753425" direction="forwards" units="years"/>
</taxon>
<taxon id="A/Chicken/BC/FAV-1630-
2/2022|EPI_ISL_19155067|A_/_H5N1|||2.3.4.4b|07.12.2022|Signore_Anthony|||21
.05.2024|Canadian_Food_Inspection_Agency|Canadian_Food_Inspection_Agency|HA
|4|A/Chicken/BC/FAV-1630-2/2022_HA|EPI3315216|DNA_IN">
<date value="2022.9342465753425" direction="forwards" units="years"/>
</taxon>
<taxon id="A/Chicken/BC/FAV-1647-
1/2022|EPI_ISL_19155091|A_/_H5N1|||2.3.4.4b|12.12.2022|Signore_Anthony|||21
.05.2024|Canadian_Food_Inspection_Agency|Canadian_Food_Inspection_Agency|HA
|4|A/Chicken/BC/FAV-1647-1/2022_HA|EPI3315408|DNA_IN">
<date value="2022.9479452054795" direction="forwards" units="years"/>
</taxon>
<taxon id="A/Chicken/BC/FAV-1647-
2/2022|EPI_ISL_19155092|A_/_H5N1|||2.3.4.4b|12.12.2022|Signore_Anthony|||21
.05.2024|Canadian_Food_Inspection_Agency|Canadian_Food_Inspection_Agency|HA
|4|A/Chicken/BC/FAV-1647-2/2022_HA|EPI3315416|DNA_IN">
<date value="2022.9479452054795" direction="forwards" units="years"/>

</taxon>
<taxon id="A/Chicken/BC/FAV-1685-1/2022|EPI_ISL_19155130|A_/_H5N1|||2.3.4.4b|28.12.2022|Signore_Anthony|||21.05.2024|Canadian_Food_Inspection_Agency|Canadian_Food_Inspection_Agency|HA|4|A/Chicken/BC/FAV-1685-1/2022_HA|EPI3315720|DNA_IN">
<date value="2022.9917808219177" direction="forwards" units="years"/>
</taxon>
<taxon id="A/Chicken/BC/FAV-1685-2/2022|EPI_ISL_19155131|A_/_H5N1|||2.3.4.4b|28.12.2022|Signore_Anthony|||21.05.2024|Canadian_Food_Inspection_Agency|Canadian_Food_Inspection_Agency|HA|4|A/Chicken/BC/FAV-1685-2/2022_HA|EPI3315728|DNA_INS">
<date value="2022.9917808219177" direction="forwards" units="years"/>
</taxon>
<taxon id="A/Chicken/BC/FAV-1689-1/2022|EPI_ISL_19155136|A_/_H5N1|||2.3.4.4b|28.12.2022|Signore_Anthony|||21.05.2024|Canadian_Food_Inspection_Agency|Canadian_Food_Inspection_Agency|HA|4|A/Chicken/BC/FAV-1689-1/2022_HA|EPI3315768|DNA_IN">
<date value="2022.9917808219177" direction="forwards" units="years"/>
</taxon>
<taxon id="A/Chicken/BC/FAV-1689-2/2022|EPI_ISL_19155137|A_/_H5N1|||2.3.4.4b|28.12.2022|Signore_Anthony|||21.05.2024|Canadian_Food_Inspection_Agency|Canadian_Food_Inspection_Agency|HA|4|A/Chicken/BC/FAV-1689-2/2022_HA|EPI3315776|DNA_IN">
<date value="2022.9917808219177" direction="forwards" units="years"/>
</taxon>
<taxon id="A/Chicken/NB/FAV-0013/2023|EPI_ISL_19155156|A_/_H5N1|||2.3.4.4b|10.01.2023|Signore_Anthony|||21.05.2024|Canadian_Food_Inspection_Agency|Canadian_Food_Inspection_Agency|HA|4|A/Chicken/NB/FAV-0013/2023_HA|EPI3315927|DNA_IN">
<date value="2023.027397260274" direction="forwards" units="years"/>
</taxon>
<taxon id="A/Chile/25945/2023|EPI_ISL_17468386|A_/_H5N1|||2.3.4.4b|24.03.2023|Campano_Constanza|||13.04.2023|Instituto_de_Salud_Publica_de_Chile|Instituto_de_Salud_Publica_de_Chile|HA|4|A/Chile/25945/2023|EPI2510183|DNA_IN">
<date value="2023.1424657534246" direction="forwards" units="years"/>
</taxon>
<taxon id="A/Chiloe_wigeon/OHiggins/240893-2/2023|EPI_ISL_17885965|A_/_H5N1|Original||2.3.4.4b|09.03.2023|GISAID_EpiFlu_Data_Curator|||19.07.2023|CEIRS_Data_Processing_and_Coordinating_Center_Center_for_Research_on_Influenza_Pathogenesis__CRIP_|CEIRS_Data_Proce">
<date value="2023.1013698630136" direction="forwards" units="years"/>
</taxon>
<taxon id="A/Cooper_s_Hawk/BC/AIVPHL-1125/2023|EPI_ISL_18665595|A_/_H5N1|Original||2.3.4.4b|23.05.2023|Caleta_Jessica_Marija|||21.12.2023|B.C._Centre_for_Disease_Control|Public_Health_Agency_of_Canada|HA|4|A/Cooper_s_Hawk/BC/AIVPHL-1125/2023_HA|EPI2856671|DNA_IN">
<date value="2023.3917808219178" direction="forwards" units="years"/>
</taxon>
<taxon id="A/Cooper_s_Hawk/BC/AIVPHL-924/2023|EPI_ISL_18665500|A_/_H5N1|Original||2.3.4.4b|30.01.2023|Caleta_Jessica_Marija|||21.12.2023|B.C._Centre_for_Disease_Control|Public_Health_Agency_of_Canada|HA|4|A/Cooper_s_Hawk/BC/AIVPHL-924/2023_HA|EPI2855965|DNA_INS">
<date value="2023.0821917808219" direction="forwards" units="years"/>
</taxon>
<taxon id="A/Cooper_s_hawk/Montana/23-016105-001/2023|EPI_ISL_17964974|A_/_H5N1|Original||2.3.4.4b|16.04.2023|Killian_Mary_Lea||23-016105-001|06.07.2023|National_Veterinary_Services_Laboratories_-_USDA|National_Veterinary_Services_Laboratories_-_USDA|HA|4|HA_A/C">

<date value="2023.2904109589042" direction="forwards" units="years"/>
</taxon>
<taxon id="A/Cooper_s_s_hawk/Colorado/23-005617-001/2023|EPI_ISL_17964858|A_/_H5N1|Original||2.3.4.4b|14.02.2023|Killian_Mary_Lea||23-005617-001|06.07.2023|National_Veterinary_Services_Laboratories_-_USDA|National_Veterinary_Services_Laboratories_-_USDA|HA|4|HA_">
<date value="2023.123287671233" direction="forwards" units="years"/>
</taxon>
<taxon id="A/Duck/Argentina/1712-5/2023|EPI_ISL_18698515|A_/_H5N1|||2.3.4.4b|19.04.2023|Benedetti_Estefania|||28.12.2023|Direccion_del_Laboratorio_Animal_Direccion_General_de_Laboratorios_y_Control_Tecnico_SENASA.|Instituto_Nacional_de_Enfermedades_Infecciosas">
<date value="2023.2986301369863" direction="forwards" units="years"/>
</taxon>
<taxon id="A/Duck/Argentina/2197-1/2023|EPI_ISL_18698523|A_/_H5N1|||2.3.4.4b|11.05.2023|Benedetti_Estefania|||28.12.2023|Direccion_del_Laboratorio_Animal_Direccion_General_de_Laboratorios_y_Control_Tecnico_SENASA.|Instituto_Nacional_de_Enfermedades_Infecciosas">
<date value="2023.358904109589" direction="forwards" units="years"/>
</taxon>
<taxon id="A/Duck/BC/FAV-1593-3/2022|EPI_ISL_19155028|A_/_H5N1|||2.3.4.4b|01.12.2022|Signore_Anthony|||21.05.2024|Canadian_Food_Inspection_Agency|Canadian_Food_Inspection_Agency|HA|4|A/Duck/BC/FAV-1593-3/2022_HA|EPI3314904|DNA_IN">
<date value="2022.9178082191781" direction="forwards" units="years"/>
</taxon>
<taxon id="A/Duck/BC/FAV-1593-4/2022|EPI_ISL_19155029|A_/_H5N1|||2.3.4.4b|01.12.2022|Signore_Anthony|||21.05.2024|Canadian_Food_Inspection_Agency|Canadian_Food_Inspection_Agency|HA|4|A/Duck/BC/FAV-1593-4/2022_HA|EPI3314912|DNA_IN">
<date value="2022.9178082191781" direction="forwards" units="years"/>
</taxon>
<taxon id="A/Dunlin/BC/AIVPHL-940/2023|EPI_ISL_18665510|A_/_H5N1|Original||2.3.4.4b|06.02.2023|Caleta_Jessica_Marija|||21.12.2023|B.C._Centre_for_Disease_Control|Public_Health_Agency_of_Canada|HA|4|A/Dunlin/BC/AIVPHL-940/2023_HA|EPI2856045|DNA_INS">
<date value="2023.1013698630136" direction="forwards" units="years"/>
</taxon>
<taxon id="A/Dunlin/BC/FAV-0053-16/2022|EPI_ISL_19155099|A_/_H5N1|||2.3.4.4b|13.12.2022|Signore_Anthony|||21.05.2024|Canadian_Food_Inspection_Agency|Canadian_Food_Inspection_Agency|HA|4|A/Dunlin/BC/FAV-0053-16/2022_HA|EPI3315472|DNA_IN">
<date value="2022.9506849315069" direction="forwards" units="years"/>
</taxon>
<taxon id="A/Elegant_tern/Arica_y_Parinacota/229476-1/2022|EPI_ISL_17885955|A_/_H5N1|Original||2.3.4.4b|24.12.2022|GISAID_EpiFlu_Data_Curator|||19.07.2023|CEIRS_Data_Processing_and_Coordinating_Center_Center_for_Research_on_Influenza_Pathogenesis__CRIP_|CEIRS_D">
<date value="2022.9808219178083" direction="forwards" units="years"/>
</taxon>
<taxon id="A/Elegant_tern/Tarapaca/229133-1/2022|EPI_ISL_17885953|A_/_H5N1|Original||2.3.4.4b|23.12.2022|GISAID_EpiFlu_Data_Curator|||19.07.2023|CEIRS_Data_Processing_and_Coordinating_Center_Center_for_Research_on_Influenza_Pathogenesis__CRIP_|CEIRS_Data_Proces">
<date value="2022.978082191781" direction="forwards" units="years"/>
</taxon>
<taxon id="A/Franklin_gull/OHiggins/236195-1/2023|EPI_ISL_19162729|A_/_H5N1|Original||2.3.4.4b|12.02.2023||||24.05.202

4|Emory_University__CEIRR_|Emory_University__CEIRR_|HA|4|A/Franklin_gull/OHiggins/236195-1/2023_HA|EPI3333780|DNA_IN">
<date value="2023.1178082191782" direction="forwards" units="years"/>
</taxon>
<taxon id="A/Gallus_gallus/Mato_Grosso_do_Sul/2108-SN52/2023|EPI_ISL_19215176|A_/_H5N1|Original||2.3.4.4b|22.09.2023||||25.06.2024|Ministerio_da_Agricultura_e_Pecuaria||HA|4|A/Gallus_gallus/Bonito/2108-SN52/2023_HA|EPI3395036|DNA_IN">
<date value="2023.7260273972602" direction="forwards" units="years"/>
</taxon>
<taxon id="A/Glaucous-winged_gull/Washington/W231990001-1/2023|EPI_ISL_19592595|A_/_H5N1|Original||2.3.4.4b|13.07.2023||||06.12.2024|Washington_State_University||HA|4|A/Glaucous-winged_gull/Washington/W231990001-1/2023_HA|EPI3675325|DNA_IN">
<date value="2023.531506849315" direction="forwards" units="years"/>
</taxon>
<taxon id="A/Glaucous-winged_gull/Washington/W232270041-2/2023|EPI_ISL_19592596|A_/_H5N1|Original||2.3.4.4b|11.08.2023||||06.12.2024|Washington_State_University||HA|4|A/Glaucous-winged_gull/Washington/W232270041-2/2023_HA|EPI3675333|DNA_IN">
<date value="2023.6109589041096" direction="forwards" units="years"/>
</taxon>
<taxon id="A/Glaucous-winged_gull/Washington/W232270041-4/2023|EPI_ISL_19592597|A_/_H5N1|Original||2.3.4.4b|11.08.2023||||06.12.2024|Washington_State_University||HA|4|A/Glaucous-winged_gull/Washington/W232270041-4/2023_HA|EPI3675341|DNA_IN">
<date value="2023.6109589041096" direction="forwards" units="years"/>
</taxon>
<taxon id="A/Goose/Argentina/389-1/2023|EPI_ISL_18698459|A_/_H5N1|||2.3.4.4b|11.02.2023|Benedetti_Estefania|||28.12.2023|Direccion_del_Laboratorio_Animal_Direccion_General_de_Laboratorios_y_Control_Tecnico_SENASA.|Instituto_Nacional_de_Enfermedades_Infecciosas">
<date value="2023.1150684931506" direction="forwards" units="years"/>
</taxon>
<taxon id="A/Gray_gull/Tarapaca/232825-1/2023|EPI_ISL_17885951|A_/_H5N1|Original||2.3.4.4b|18.01.2023|GISAID_EpiFlu_Data_Curator|||19.07.2023|CEIRS_Data_Processing_and_Coordinating_Center_Center_for_Research_on_Influenza_Pathogenesis__CRIP_|CEIRS_Data_Processin">
<date value="2023.0493150684931" direction="forwards" units="years"/>
</taxon>
<taxon id="A/Great_Blue_Heron/BC/AIVPHL-835/2022|EPI_ISL_18665537|A_/_H5N1|Original||2.3.4.4b|14.12.2022|Caleta_Jessica_Marija|||21.12.2023|B.C._Centre_for_Disease_Control|Public_Health_Agency_of_Canada|HA|4|A/Great_Blue_Heron/BC/AIVPHL-835/2022_HA|EPI2856234|DN">
<date value="2022.9534246575342" direction="forwards" units="years"/>
</taxon>
<taxon id="A/Great_Horned_Owl/ON/FAV-0002-2/2022|EPI_ISL_19155078|A_/_H5N1|||2.3.4.4b|10.12.2022|Signore_Anthony|||21.05.2024|Canadian_Food_Inspection_Agency|Canadian_Food_Inspection_Agency|HA|4|A/Great_Horned_Owl/ON/FAV-0002-2/2022_HA|EPI3315304|DNA_IN">
<date value="2022.9424657534246" direction="forwards" units="years"/>
</taxon>
<taxon id="A/Great_egret/Araucania/240518/2023|EPI_ISL_17885950|A_/_H5N1|Original||2.3.4.4b|07.03.2023|GISAID_EpiFlu_Data_Curator|||24.07.2023|CEIRS_Data_Processing_and_Coordinating_Center_Center_for_Research_on_Influenza_Pathogenesis__CRIP_|CEIRS_Data_Processi">
<date value="2023.0958904109589" direction="forwards" units="years"/>
</taxon>
<taxon id="A/Great_grabe/Atacama/231482-1/2023|EPI_ISL_19162730|A_/_H5N1|Original||2.3.4.4b|09.01.2023||||24.05.202

4|Emory_University__CEIRR_|Emory_University__CEIRR_|HA|4|A/Great_grabe/Atacama/231482-1/2023_HA|EPI3333788|DNA_IN">
<date value="2023.0246575342467" direction="forwards" units="years"/>
</taxon>
<taxon id="A/Great_grabe/Atacama/231482-2/2023|EPI_ISL_19162731|A_/_H5N1|Original||2.3.4.4b|09.01.2023||||24.05.2024|Emory_University__CEIRR_|Emory_University__CEIRR_|HA|4|A/Great_grabe/Atacama/231482-2/2023_HA|EPI3333795|DNA_IN">
<date value="2023.0246575342467" direction="forwards" units="years"/>
</taxon>
<taxon id="A/Guanay_cormorant/Chile/239584/2023|EPI_ISL_19391460|A_/_H5N1|Original||2.3.4.4b|01.03.2023||||09.09.2024|CEIRS_Data_Processing_and_Coordinating_Center_Center_for_Research_on_Influenza_Pathogenesis__CRIP_|CEIRS_Data_Processing_and_Coordinating_Center">
<date value="2023.0794520547945" direction="forwards" units="years"/>
</taxon>
<taxon id="A/Gull/Chile/227023-2/2022|EPI_ISL_17011964|A_/_H5N1|||2.3.4.4b|07.12.2022||||22.02.2023|CEIRS_Data_Processing_and_Coordinating_Center_Center_for_Research_on_Influenza_Pathogenesis__CRIP_|CEIRS_Data_Processing_and_Coordinating_Center_Center_for_Resea">
<date value="2022.9342465753425" direction="forwards" units="years"/>
</taxon>
<taxon id="A/Gull/Chile/227023-3/2022|EPI_ISL_17011958|A_/_H5N1|||2.3.4.4b|07.12.2022||||22.02.2023|CEIRS_Data_Processing_and_Coordinating_Center_Center_for_Research_on_Influenza_Pathogenesis__CRIP_|CEIRS_Data_Processing_and_Coordinating_Center_Center_for_Resea">
<date value="2022.9342465753425" direction="forwards" units="years"/>
</taxon>
<taxon id="A/Harbor_seal/Washington/W232490069-3/2023|EPI_ISL_19592610|A_/_H5N1|Original||2.3.4.4b|13.08.2023||||06.12.2024|Washington_State_University||HA|4|A/Harbor_seal/Washington/W232490069-3/2023_HA|EPI3675357|DNA_IN">
<date value="2023.6164383561643" direction="forwards" units="years"/>
</taxon>
<taxon id="A/Humboldt_penguin/Antofagasta/236063-2/2023|EPI_ISL_18760066|A_/_H5N1|Original||2.3.4.4b|05.02.2023||||14.01.2024|Emory_University__CEIRR_|Emory_University__CEIRR_|HA|4|A/Humboldt_penguin/Antofagasta/236063-2/2023_HA|EPI2913222|DNA_IN">
<date value="2023.0986301369862" direction="forwards" units="years"/>
</taxon>
<taxon id="A/Humboldt_penguin/Coquimbo/239590/2023|EPI_ISL_17885948|A_/_H5N1|Original||2.3.4.4b|28.02.2023|GISAID_EpiFlu_Data_Curator|||19.07.2023|CEIRS_Data_Processing_and_Coordinating_Center_Center_for_Research_on_Influenza_Pathogenesis__CRIP_|CEIRS_Data_Proc">
<date value="2023.1616438356164" direction="forwards" units="years"/>
</taxon>
<taxon id="A/Inca_tern/Antofagasta/238083/2023|EPI_ISL_19131156|A_/_H5N1|Original||2.3.4.4b|22.02.2023||||10.05.2024|CEIRS_Data_Processing_and_Coordinating_Center_Center_for_Research_on_Influenza_Pathogenesis__CRIP_||HA|4|A/Inca_tern/Antofagasta/238083/2023_HA|E">
<date value="2023.145205479452" direction="forwards" units="years"/>
</taxon>
<taxon id="A/Inca_tern/Arica_y_Parinacota/227519-1/2022|EPI_ISL_19131159|A_/_H5N1|Original||2.3.4.4b|12.12.2022||||10.05.2024|CEIRS_Data_Processing_and_Coordinating_Center_Center_for_Research_on_Influenza_Pathogenesis__CRIP_||HA|4|A/Inca_tern/Arica_y_Parinacota/">
<date value="2022.9479452054795" direction="forwards" units="years"/>
</taxon>

<taxon id="A/Inca_tern/Coquimbo/239619/2023|EPI_ISL_19131162|A_/_H5N1|Origi
nal||2.3.4.4b|01.03.2023||||10.05.2024|CEIRS_Data_Processing_and_Coordinati
ng_Center_Center_for_Research_on_Influenza_Pathogenesis__CRIP_||HA|4|A/Inca
_tern/Coquimbo/239619/2023_HA|EPI3276">
<date value="2023.0794520547945" direction="forwards" units="years"/>
</taxon>
<taxon id="A/Kelp_Gull/Hound_Bay/133744/2023|EPI_ISL_18592422|A_/_H5N1|Clin
ical_Sample||2.3.4.4b|30.10.2023|Mollett_Benjamin|||06.12.2023|Animal_and_P
lant_Health_Agency__APHA_|Animal_and_Plant_Health_Agency__APHA_|HA|4|A/Kelp
_Gull/Hound_Bay/133744/2023|HA|EPI281">
<date value="2023.7753424657535" direction="forwards" units="years"/>
</taxon>
<taxon id="A/Kelp_Gull/Hound_Bay/133747/2023|EPI_ISL_18592423|A_/_H5N1|Clin
ical_Sample||2.3.4.4b|30.10.2023|Mollett_Benjamin|||06.12.2023|Animal_and_P
lant_Health_Agency__APHA_|Animal_and_Plant_Health_Agency__APHA_|HA|4|A/Kelp
_Gull/Hound_Bay/133747/2023|HA|EPI281">
<date value="2023.7753424657535" direction="forwards" units="years"/>
</taxon>
<taxon id="A/Kelp_Gull/Moltke_Harbour/133754/2023|EPI_ISL_18592425|A_/_H5N1
|Clinical_Sample||2.3.4.4b|31.10.2023|Mollett_Benjamin|||06.12.2023|Animal_
and_Plant_Health_Agency__APHA_|Animal_and_Plant_Health_Agency__APHA_|HA|4|A
/Kelp_Gull/Moltke_Harbour/133754/2023">
<date value="2023.7780821917809" direction="forwards" units="years"/>
</taxon>
<taxon id="A/Kelp_Gull/Penguin_River/141234/2023|EPI_ISL_18742215|A_/_H5N1|
Clinical_Sample||2.3.4.4b|08.11.2023|Mollett_Benjamin|||25.06.2024|Animal_a
nd_Plant_Health_Agency__APHA_|Animal_and_Plant_Health_Agency__APHA_|HA|4|A/
Kelp_Gull/Penguin_River/141234/2023|H">
<date value="2023.854794520548" direction="forwards" units="years"/>
</taxon>
<taxon id="A/Kelp_Gull/Penguin_River/141239/2023|EPI_ISL_18742217|A_/_H5N1|
Clinical_Sample||2.3.4.4b|08.11.2023|Mollett_Benjamin|||25.06.2024|Animal_a
nd_Plant_Health_Agency__APHA_|Animal_and_Plant_Health_Agency__APHA_|HA|4|A/
Kelp_Gull/Penguin_River/141239/2023|H">
<date value="2023.854794520548" direction="forwards" units="years"/>
</taxon>
<taxon id="A/Kelp_Gull/South_Georgia_and_the_South_Sandwich_Islands/136/202
3|EPI_ISL_19313644|A_/_H5N1|Original||2.3.4.4b|08.11.2023||||06.08.2024|Ani
mal_and_Plant_Health_Agency__APHA_||HA|4|A/Kelp_Gull/South_Georgia_and_the_
South_Sandwich_Islands/136/2023_HA|EPI">
<date value="2023.854794520548" direction="forwards" units="years"/>
</taxon>
<taxon id="A/Kelp_Gull/South_Georgia_and_the_South_Sandwich_Islands/152/202
3|EPI_ISL_19313573|A_/_H5N1|Original||2.3.4.4b|30.10.2023||||06.08.2024|Ani
mal_and_Plant_Health_Agency__APHA_||HA|4|A/Kelp_Gull/South_Georgia_and_the_
South_Sandwich_Islands/152/2023_HA|EPI">
<date value="2023.7753424657535" direction="forwards" units="years"/>
</taxon>
<taxon id="A/Kelp_Gull/South_Georgia_and_the_South_Sandwich_Islands/160/202
3|EPI_ISL_19313506|A_/_H5N1|Original||2.3.4.4b|30.10.2023||||06.08.2024|Ani
mal_and_Plant_Health_Agency__APHA_||HA|4|A/Kelp_Gull/South_Georgia_and_the_
South_Sandwich_Islands/160/2023_HA|EPI">
<date value="2023.7753424657535" direction="forwards" units="years"/>
</taxon>
<taxon id="A/Kelp_Gull/South_Georgia_and_the_South_Sandwich_Islands/32/2023
|EPI_ISL_19313514|A_/_H5N1|Original||2.3.4.4b|03.11.2023||||06.08.2024|Anim
al_and_Plant_Health_Agency__APHA_||HA|4|A/Kelp_Gull/South_Georgia_and_the_S
outh_Sandwich_Islands/32/2023_HA|EPI34">
<date value="2023.841095890411" direction="forwards" units="years"/>
</taxon>

<taxon id="A/Kelp_Gull/South_Georgia_and_the_South_Sandwich_Islands/56/2023|EPI_ISL_19313522|A_/_H5N1|Original||2.3.4.4b|31.10.2023||||06.08.2024|Animal_and_Plant_Health_Agency__APHA_||HA|4|A/Kelp_Gull/South_Georgia_and_the_South_Sandwich_Islands/56/2023_HA|EPI34">
<date value="2023.7780821917809" direction="forwards" units="years"/>
</taxon>
<taxon id="A/Kelp_Gull/South_Georgia_and_the_South_Sandwich_Islands/72/2023|EPI_ISL_19313477|A_/_H5N1|Original||2.3.4.4b|08.11.2023||||06.08.2024|Animal_and_Plant_Health_Agency__APHA_||HA|4|A/Kelp_Gull/South_Georgia_and_the_South_Sandwich_Islands/72/2023_HA|EPI34">
<date value="2023.854794520548" direction="forwards" units="years"/>
</taxon>
<taxon id="A/Kelp_gull/Maule/239349/2023|EPI_ISL_17885972|A_/_H5N1|Original||2.3.4.4b|01.03.2023|GISAID_EpiFlu_Data_Curator|||19.07.2023|CEIRS_Data_Processing_and_Coordinating_Center_Center_for_Research_on_Influenza_Pathogenesis__CRIP_|CEIRS_Data_Processing_and">
<date value="2023.0794520547945" direction="forwards" units="years"/>
</taxon>
<taxon id="A/Layer_Chicken/BC/FAV-1623-1/2022|EPI_ISL_19155058|A_/_H5N1|||2.3.4.4b|06.12.2022|Signore_Anthony|||21.05.2024|Canadian_Food_Inspection_Agency|Canadian_Food_Inspection_Agency|HA|4|A/Layer_Chicken/BC/FAV-1623-1/2022_HA|EPI3315144|DNA_IN">
<date value="2022.9315068493152" direction="forwards" units="years"/>
</taxon>
<taxon id="A/Layer_Chicken/BC/FAV-1623-2/2022|EPI_ISL_19155059|A_/_H5N1|||2.3.4.4b|06.12.2022|Signore_Anthony|||21.05.2024|Canadian_Food_Inspection_Agency|Canadian_Food_Inspection_Agency|HA|4|A/Layer_Chicken/BC/FAV-1623-2/2022_HA|EPI3315152|DNA_IN">
<date value="2022.9315068493152" direction="forwards" units="years"/>
</taxon>
<taxon id="A/Long_Eared_Owl/BC/AIVPHL-925/2023|EPI_ISL_18665502|A_/_H5N1|Original||2.3.4.4b|30.01.2023|Caleta_Jessica_Marija|||21.12.2023|B.C._Centre_for_Disease_Control|Public_Health_Agency_of_Canada|HA|4|A/Long_Eared_Owl/BC/AIVPHL-925/2023_HA|EPI2855981|DNA_IN">
<date value="2023.0821917808219" direction="forwards" units="years"/>
</taxon>
<taxon id="A/Mixed/AB/FAV-1654/2022|EPI_ISL_19155100|A_/_H5N1|||2.3.4.4b|14.12.2022|Signore_Anthony|||21.05.2024|Canadian_Food_Inspection_Agency|Canadian_Food_Inspection_Agency|HA|4|A/Mixed/AB/FAV-1654/2022_HA|EPI3315480|DNA_IN">
<date value="2022.9534246575342" direction="forwards" units="years"/>
</taxon>
<taxon id="A/Northern_Pintail/BC/FAV-0053-43/2023|EPI_ISL_19155143|A_/_H5N1|||2.3.4.4b|06.01.2023|Signore_Anthony|||21.05.2024|B.C._Centre_for_Disease_Control|Canadian_Food_Inspection_Agency|HA|4|A/Northern_Pintail/BC/FAV-0053-43/2023_HA|EPI3315824|DNA_IN">
<date value="2023.0164383561644" direction="forwards" units="years"/>
</taxon>
<taxon id="A/Numida_meleagris/Santa_Catarina/1843-N3/2023|EPI_ISL_19215178|A_/_H5N1|Original||2.3.4.4b|15.09.2023||||25.11.2024|Ministerio_da_Agricultura_e_Pecuaria||HA|4|A/Numida_meleagris/Maracaja/1843-N3/2023_HA|EPI3395052|DNA_IN">
<date value="2023.7068493150684" direction="forwards" units="years"/>
</taxon>
<taxon id="A/Otaria_flavescens/Rio_Grande_do_Sul/2165-SO/2023|EPI_ISL_19215180|A_/_H5N1|Original||2.3.4.4b|11.10.2023||||25.06.2024|Ministerio_da_Agricultura_e_Pecuaria||HA|4|A/Otaria_flavescens/Torres/2165-SO/2023_HA|EPI3395068|DNA_IN">
<date value="2023.7232876712328" direction="forwards" units="years"/>
</taxon>

<taxon id="A/Pelecanus/Peru/VFAR-140/2022|EPI_ISL_17099964|A_/_H5N1|SPF_Eggs||2.3.4.4b|01.12.2022|Tataje_Luis|VFAR-140|VFAR-140|06.03.2023|Farmacologicos_Veterinarios_S.A.C.__FARVET_SAC_|Universidad_Privada_San_Juan_Bautista|HA|4|A/Pelecanus/Peru/VFAR-140/2022|E">
<date value="2022.9178082191781" direction="forwards" units="years"/>
</taxon>
<taxon id="A/Pelican/Antofagasta/228244-2/2022|EPI_ISL_17885944|A_/_H5N1|Original||2.3.4.4b|16.12.2022|GISAID_EpiFlu_Data_Curator|||29.06.2023|CEIRS_Data_Processing_and_Coordinating_Center_Center_for_Research_on_Influenza_Pathogenesis__CRIP_|CEIRS_Data_Processi">
<date value="2022.958904109589" direction="forwards" units="years"/>
</taxon>
<taxon id="A/Pelican/Antofagasta/228246-2/2022|EPI_ISL_17885934|A_/_H5N1|Original||2.3.4.4b|15.12.2022|GISAID_EpiFlu_Data_Curator|||19.07.2023|CEIRS_Data_Processing_and_Coordinating_Center_Center_for_Research_on_Influenza_Pathogenesis__CRIP_|CEIRS_Data_Processi">
<date value="2022.9561643835616" direction="forwards" units="years"/>
</taxon>
<taxon id="A/Pelican/Antofagasta/228246-3/2022|EPI_ISL_17885931|A_/_H5N1|Original||2.3.4.4b|15.12.2022|GISAID_EpiFlu_Data_Curator|||19.07.2023|CEIRS_Data_Processing_and_Coordinating_Center_Center_for_Research_on_Influenza_Pathogenesis__CRIP_|CEIRS_Data_Processi">
<date value="2022.9561643835616" direction="forwards" units="years"/>
</taxon>
<taxon id="A/Pelican/Antofagasta/228272-1/2022|EPI_ISL_17885929|A_/_H5N1|Original||2.3.4.4b|18.12.2022|GISAID_EpiFlu_Data_Curator|||19.07.2023|CEIRS_Data_Processing_and_Coordinating_Center_Center_for_Research_on_Influenza_Pathogenesis__CRIP_|CEIRS_Data_Processi">
<date value="2022.964383561644" direction="forwards" units="years"/>
</taxon>
<taxon id="A/Pelican/Atacama/229424-2/2022|EPI_ISL_17885927|A_/_H5N1|Original||2.3.4.4b|22.12.2022|GISAID_EpiFlu_Data_Curator|||19.07.2023|CEIRS_Data_Processing_and_Coordinating_Center_Center_for_Research_on_Influenza_Pathogenesis__CRIP_|CEIRS_Data_Processing_a">
<date value="2022.9753424657533" direction="forwards" units="years"/>
</taxon>
<taxon id="A/Pelican/Atacama/229450-2/2022|EPI_ISL_17885926|A_/_H5N1|Original||2.3.4.4b|26.12.2022|GISAID_EpiFlu_Data_Curator|||19.07.2023|CEIRS_Data_Processing_and_Coordinating_Center_Center_for_Research_on_Influenza_Pathogenesis__CRIP_|CEIRS_Data_Processing_a">
<date value="2022.986301369863" direction="forwards" units="years"/>
</taxon>
<taxon id="A/Pelican/Atacama/230158-1/2022|EPI_ISL_17885925|A_/_H5N1|Original||2.3.4.4b|30.12.2022|GISAID_EpiFlu_Data_Curator|||19.07.2023|CEIRS_Data_Processing_and_Coordinating_Center_Center_for_Research_on_Influenza_Pathogenesis__CRIP_|CEIRS_Data_Processing_a">
<date value="2022.9972602739726" direction="forwards" units="years"/>
</taxon>
<taxon id="A/Pelican/Atacama/230158-2/2022|EPI_ISL_17885924|A_/_H5N1|Original||2.3.4.4b|30.12.2022|GISAID_EpiFlu_Data_Curator|||29.06.2023|CEIRS_Data_Processing_and_Coordinating_Center_Center_for_Research_on_Influenza_Pathogenesis__CRIP_|CEIRS_Data_Processing_a">
<date value="2022.9972602739726" direction="forwards" units="years"/>
</taxon>

<taxon id="A/Pelican/Chile/227023-1/2022|EPI_ISL_17050893|A_/_H5N1|||2.3.4.4b|07.12.2022||||26.02.2023|||HA|4|A/Pelican/Chile/227023-1/2022|EPI2427757|DNA_IN">
<date value="2022.9342465753425" direction="forwards" units="years"/>
</taxon>
<taxon id="A/Pelican/Chile/6618-1/2022|EPI_ISL_17011962|A_/_H5N1|||2.3.4.4b|05.12.2022||||22.02.2023|||HA|4|A/Pelican/Chile/6618-1/2022|EPI2416961|DNA_IN">
<date value="2022.9287671232876" direction="forwards" units="years"/>
</taxon>
<taxon id="A/Pelican/Chile/6618-2/2022|EPI_ISL_17011961|A_/_H5N1|||2.3.4.4b|05.12.2022||||22.02.2023|||HA|4|A/Pelican/Chile/6618-2/2022|EPI2416955|DNA_IN">
<date value="2022.9287671232876" direction="forwards" units="years"/>
</taxon>
<taxon id="A/Pelican/Chile/6924-1/2022|EPI_ISL_17011963|A_/_H5N1|||2.3.4.4b|06.12.2022||||22.02.2023|||HA|4|A/Pelican/Chile/6924-1/2022|EPI2417011|DNA_IN">
<date value="2022.9315068493152" direction="forwards" units="years"/>
</taxon>
<taxon id="A/Pelican/Chile/6955-1/2022|EPI_ISL_17011956|A_/_H5N1|||2.3.4.4b|06.12.2022||||22.02.2023|||HA|4|A/Pelican/Chile/6955-1/2022|EPI2416941|DNA_IN">
<date value="2022.9315068493152" direction="forwards" units="years"/>
</taxon>
<taxon id="A/Pelican/Chile/6955-3/2022|EPI_ISL_17011957|A_/_H5N1|||2.3.4.4b|06.12.2022||||22.02.2023|||HA|4|A/Pelican/Chile/6955-3/2022|EPI2416946|DNA_IN">
<date value="2022.9315068493152" direction="forwards" units="years"/>
</taxon>
<taxon id="A/Pelican/Chile/6958-1/2022|EPI_ISL_17011959|A_/_H5N1|||2.3.4.4b|06.12.2022||||22.02.2023|||HA|4|A/Pelican/Chile/6958-1/2022|EPI2416967|DNA_IN">
<date value="2022.9315068493152" direction="forwards" units="years"/>
</taxon>
<taxon id="A/Pelican/Chile/7087-1/2022|EPI_ISL_17012018|A_/_H5N1|||2.3.4.4b|07.12.2022||||22.02.2023|||HA|4|A/Pelican/Chile/7087-1/2022|EPI2417003|DNA_IN">
<date value="2022.9342465753425" direction="forwards" units="years"/>
</taxon>
<taxon id="A/Pelican/Coquimbo/230310-1/2023|EPI_ISL_17885923|A_/_H5N1|Original||2.3.4.4b|02.01.2023|GISAID_EpiFlu_Data_Curator|||19.07.2023|CEIRS_Data_Processing_and_Coordinating_Center_Center_for_Research_on_Influenza_Pathogenesis__CRIP_|CEIRS_Data_Processing_">
<date value="2023.0054794520547" direction="forwards" units="years"/>
</taxon>
<taxon id="A/Pelican/Coquimbo/231946-1/2023|EPI_ISL_17885922|A_/_H5N1|Original||2.3.4.4b|14.01.2023|GISAID_EpiFlu_Data_Curator|||24.07.2023|CEIRS_Data_Processing_and_Coordinating_Center_Center_for_Research_on_Influenza_Pathogenesis__CRIP_|CEIRS_Data_Processing_">
<date value="2023.0383561643835" direction="forwards" units="years"/>
</taxon>
<taxon id="A/Pelican/Maule/231155-2/2023|EPI_ISL_17885921|A_/_H5N1|Original||2.3.4.4b|09.01.2023|GISAID_EpiFlu_Data_Curator|||19.07.2023|CEIRS_Data_Processing_and_Coordinating_Center_Center_for_Research_on_Influenza_Pathogenesis__CRIP_|CEIRS_Data_Processing_and">
<date value="2023.0246575342467" direction="forwards" units="years"/>
</taxon>

<taxon id="A/Pelican/Nuble/233947-
2/2023|EPI_ISL_17885918|A_/_H5N1|Original||2.3.4.4b|26.01.2023|GISAID_EpiFl
u_Data_Curator|||29.06.2023|CEIRS_Data_Processing_and_Coordinating_Center_C
enter_for_Research_on_Influenza_Pathogenesis__CRIP_|CEIRS_Data_Processing_a
nd">
<date value="2023.0712328767124" direction="forwards" units="years"/>
</taxon>
<taxon id="A/Pelican/OHiggins/233663-
1/2023|EPI_ISL_17885915|A_/_H5N1|Original||2.3.4.4b|24.01.2023|GISAID_EpiFl
u_Data_Curator|||19.07.2023|CEIRS_Data_Processing_and_Coordinating_Center_C
enter_for_Research_on_Influenza_Pathogenesis__CRIP_|CEIRS_Data_Processing_"
>
<date value="2023.0657534246575" direction="forwards" units="years"/>
</taxon>
<taxon id="A/Pelican/OHiggins/233721-
1/2023|EPI_ISL_17885912|A_/_H5N1|Original||2.3.4.4b|24.01.2023|GISAID_EpiFl
u_Data_Curator|||19.07.2023|CEIRS_Data_Processing_and_Coordinating_Center_C
enter_for_Research_on_Influenza_Pathogenesis__CRIP_|CEIRS_Data_Processing_"
>
<date value="2023.0657534246575" direction="forwards" units="years"/>
</taxon>
<taxon id="A/Pelican/Tarapaca/227436-
2/2022|EPI_ISL_17885942|A_/_H5N1|Original||2.3.4.4b|09.12.2022|GISAID_EpiFl
u_Data_Curator|||19.07.2023|CEIRS_Data_Processing_and_Coordinating_Center_C
enter_for_Research_on_Influenza_Pathogenesis__CRIP_|CEIRS_Data_Processing_"
>
<date value="2022.9397260273972" direction="forwards" units="years"/>
</taxon>
<taxon id="A/Peregrine_Falcon/BC/AIVPHL-
1151/2023|EPI_ISL_18665598|A_/_H5N1|Original||2.3.4.4b|05.06.2023|Caleta_Je
ssica_Marija|||21.12.2023|B.C._Centre_for_Disease_Control|Public_Health_Age
ncy_of_Canada|HA|4|A/Peregrine_Falcon/BC/AIVPHL-1151/2023_HA|EPI2856694|">
<date value="2023.427397260274" direction="forwards" units="years"/>
</taxon>
<taxon id="A/Peregrine_Falcon/BC/AIVPHL-
877/2023|EPI_ISL_18665560|A_/_H5N1|Original||2.3.4.4b|09.01.2023|Caleta_Jes
sica_Marija|||21.12.2023|B.C._Centre_for_Disease_Control|Public_Health_Agen
cy_of_Canada|HA|4|A/Peregrine_Falcon/BC/AIVPHL-877/2023_HA|EPI2856420|DN">
<date value="2023.0246575342467" direction="forwards" units="years"/>
</taxon>
<taxon id="A/Peruvean_booby/Atacama/230579-
1/2023|EPI_ISL_19139974|A_/_H5N1|Original||2.3.4.4b|03.01.2023||||15.05.202
4|Emory_University__CEIRR_||HA|4|A/Peruvean_booby/Atacama/230579-
1/2023_HA|EPI3293636|DNA_IN">
<date value="2023.0082191780823" direction="forwards" units="years"/>
</taxon>
<taxon id="A/Peruvean_booby/OHiggins/234887-
1/2023|EPI_ISL_19162636|A_/_H5N1|Original||2.3.4.4b|31.01.2023||||24.05.202
4|Emory_University__CEIRR_||HA|4|A/Peruvean_booby/OHiggins/234887-
1/2023_HA|EPI3333386|DNA_IN">
<date value="2023.0849315068492" direction="forwards" units="years"/>
</taxon>
<taxon id="A/Peruvean_booby/Valparaiso/238535/2023|EPI_ISL_19131163|A_/_H5N
1|Original||2.3.4.4b|24.02.2023||||10.05.2024|CEIRS_Data_Processing_and_Coo
rdinating_Center_Center_for_Research_on_Influenza_Pathogenesis__CRIP_||HA|4
|A/Peruvean_booby/Valparaiso/238535/2">
<date value="2023.150684931507" direction="forwards" units="years"/>
</taxon>
<taxon id="A/Peruvian_booby/Antofagasta/236109-
1/2023|EPI_ISL_18690723|A_/_H5N1|Original||2.3.4.4b|10.02.2023||||26.12.202

3|Emory_University__CEIRR_|Emory_University__CEIRR_|HA|4|A/Peruvian_booby/Antofagasta/236109-1/2023_HA|EPI2868235|DNA_IN">
<date value="2023.1123287671232" direction="forwards" units="years"/>
</taxon>
<taxon id="A/Peruvian_booby/OHiggins/242755-1/2023|EPI_ISL_18760067|A_/_H5N1|Original||2.3.4.4b|17.03.2023||||14.01.2024|Emory_University__CEIRR_|Emory_University__CEIRR_|HA|4|A/Peruvian_booby/OHiggins/242755-1/2023_HA|EPI2913229|DNA_IN">
<date value="2023.123287671233" direction="forwards" units="years"/>
</taxon>
<taxon id="A/Pluvialis_dominica/Sao_Paulo/2252-N/2023|EPI_ISL_19215181|A_/_H5N1|Original||2.3.4.4b|08.11.2023||||25.06.2024|Ministerio_da_Agricultura_e_Pecuaria||HA|4|A/Pluvialis_dominica/BertiogaBR/2252-N/2023_HA|EPI3395076|DNA_IN">
<date value="2023.854794520548" direction="forwards" units="years"/>
</taxon>
<taxon id="A/Procellaria_aequinoctialis/Sao_Paulo/2259-N/2023|EPI_ISL_19215182|A_/_H5N1|Original||2.3.4.4b|09.11.2023||||25.06.2024|Ministerio_da_Agricultura_e_Pecuaria||HA|4|A/Procellaria_aequinoctialis/SaoSebastiaoBR/2259-N/2023_HA|EPI3395084|DNA_IN">
<date value="2023.8575342465754" direction="forwards" units="years"/>
</taxon>
<taxon id="A/Procellaria_aequinoctialis/Sao_Paulo/2271-N/2023|EPI_ISL_19215190|A_/_H5N1|Original||2.3.4.4b|10.11.2023||||25.06.2024|Ministerio_da_Agricultura_e_Pecuaria||HA|4|A/Procellaria_aequinoctialis/UbatubaBR/2271-N/2023_HA|EPI3395148|DNA_IN">
<date value="2023.8602739726027" direction="forwards" units="years"/>
</taxon>
<taxon id="A/Red-Tailed_Hawk/ON/FAV-0002-1/2022|EPI_ISL_19155113|A_/_H5N1|||2.3.4.4b|16.12.2022|Signore_Anthony|||21.05.2024|Canadian_Food_Inspection_Agency|Canadian_Food_Inspection_Agency|HA|4|A/Red-Tailed_Hawk/ON/FAV-0002-1/2022_HA|EPI3315584|DNA_IN">
<date value="2022.958904109589" direction="forwards" units="years"/>
</taxon>
<taxon id="A/Red_Tailed_Hawk/BC/AIVPHL-1007/2023|EPI_ISL_18665553|A_/_H5N1|Original||2.3.4.4b|20.03.2023|Caleta_Jessica_Marija|||21.12.2023|B.C._Centre_for_Disease_Control|Public_Health_Agency_of_Canada|HA|4|A/Red_Tailed_Hawk/BC/AIVPHL-1007/2023_HA|EPI2856367|DN">
<date value="2023.131506849315" direction="forwards" units="years"/>
</taxon>
<taxon id="A/Red_Tailed_Hawk/BC/AIVPHL-1009/2023|EPI_ISL_18665559|A_/_H5N1|Original||2.3.4.4b|20.03.2023|Caleta_Jessica_Marija|||21.12.2023|B.C._Centre_for_Disease_Control|Public_Health_Agency_of_Canada|HA|4|A/Red_Tailed_Hawk/BC/AIVPHL-1009/2023_HA|EPI2856413|DN">
<date value="2023.131506849315" direction="forwards" units="years"/>
</taxon>
<taxon id="A/Red_Tailed_Hawk/BC/AIVPHL-1028/2023|EPI_ISL_18665567|A_/_H5N1|Original||2.3.4.4b|27.03.2023|Caleta_Jessica_Marija|||21.12.2023|B.C._Centre_for_Disease_Control|Public_Health_Agency_of_Canada|HA|4|A/Red_Tailed_Hawk/BC/AIVPHL-1028/2023_HA|EPI2856469|DN">
<date value="2023.150684931507" direction="forwards" units="years"/>
</taxon>
<taxon id="A/Red_Tailed_Hawk/BC/AIVPHL-859/2022|EPI_ISL_18665549|A_/_H5N1|Original||2.3.4.4b|30.12.2022|Caleta_Jessica_Marija|||21.12.2023|B.C._Centre_for_Disease_Control|Public_Health_Agency_of_Canada|HA|4|A/Red_Tailed_Hawk/BC/AIVPHL-859/2022_HA|EPI2856330|DNA_">
<date value="2022.9972602739726" direction="forwards" units="years"/>
</taxon>
<taxon id="A/Red_Tailed_Hawk/BC/AIVPHL-860/2022|EPI_ISL_18665552|A_/_H5N1|Original||2.3.4.4b|30.12.2022|Caleta_Jes

sica_Marija|||21.12.2023|B.C._Centre_for_Disease_Control|Public_Health_Agency_of_Canada|HA|4|A/Red_Tailed_Hawk/BC/AIVPHL-860/2022_HA|EPI2856355|DNA_">
<date value="2022.9972602739726" direction="forwards" units="years"/>
</taxon>
<taxon id="A/Red_Tailed_Hawk/BC/AIVPHL-931/2023|EPI_ISL_18665508|A_/_H5N1|Original||2.3.4.4b|30.01.2023|Caleta_Jessica_Marija|||21.12.2023|B.C._Centre_for_Disease_Control|Public_Health_Agency_of_Canada|HA|4|A/Red_Tailed_Hawk/BC/AIVPHL-931/2023_HA|EPI2856029|DNA_">
<date value="2023.0821917808219" direction="forwards" units="years"/>
</taxon>
<taxon id="A/Red_Tailed_Hawk/BC/AIVPHL-990/2023|EPI_ISL_18665544|A_/_H5N1|Original||2.3.4.4b|13.03.2023|Caleta_Jessica_Marija|||21.12.2023|B.C._Centre_for_Disease_Control|Public_Health_Agency_of_Canada|HA|4|A/Red_Tailed_Hawk/BC/AIVPHL-990/2023_HA|EPI2856299|DNA_">
<date value="2023.1123287671232" direction="forwards" units="years"/>
</taxon>
<taxon id="A/Sanderling/Arica_y_Parinacota/230758-1/2022|EPI_ISL_17885980|A_/_H5N1|Original||2.3.4.4b|30.12.2022|GISAID_EpiFlu_Data_Curator|||19.07.2023|CEIRS_Data_Processing_and_Coordinating_Center_Center_for_Research_on_Influenza_Pathogenesis__CRIP_|CEIRS_Dat">
<date value="2022.9972602739726" direction="forwards" units="years"/>
</taxon>
<taxon id="A/Sanderling/Arica_y_Parinacota/240265/2023|EPI_ISL_17885978|A_/_H5N1|Original||2.3.4.4b|03.03.2023|GISAID_EpiFlu_Data_Curator|||29.06.2023|CEIRS_Data_Processing_and_Coordinating_Center_Center_for_Research_on_Influenza_Pathogenesis__CRIP_|CEIRS_Data_">
<date value="2023.0849315068492" direction="forwards" units="years"/>
</taxon>
<taxon id="A/Sanderling/Chile/230758-2/2023|EPI_ISL_19404931|A_/_H5N1|Original||2.3.4.4b|17.03.2023||||10.09.2024|CEIRS_Data_Processing_and_Coordinating_Center_Center_for_Research_on_Influenza_Pathogenesis__CRIP_|CEIRS_Data_Processing_and_Coordinating_Center_Ce">
<date value="2023.123287671233" direction="forwards" units="years"/>
</taxon>
<taxon id="A/Sea_Lion/Argentina/3849-4/2023|EPI_ISL_18698754|A_/_H5N1|||2.3.4.4b|08.08.2023|Benedetti_Estefania|||03.01.2024|Direccion_del_Laboratorio_Animal_Direccion_General_de_Laboratorios_y_Control_Tecnico_SENASA.|Instituto_Nacional_de_Enfermedades_Infecci">
<date value="2023.6027397260275" direction="forwards" units="years"/>
</taxon>
<taxon id="A/Sea_Lion/Argentina/3893-1/2023|EPI_ISL_18698755|A_/_H5N1|||2.3.4.4b|11.08.2023|Benedetti_Estefania|||03.01.2024|Direccion_del_Laboratorio_Animal_Direccion_General_de_Laboratorios_y_Control_Tecnico_SENASA.|Instituto_Nacional_de_Enfermedades_Infecci">
<date value="2023.6109589041096" direction="forwards" units="years"/>
</taxon>
<taxon id="A/Sea_Lion/Peru/LIM-SER036/2023|EPI_ISL_18054502|A_/_H5N1|Original||2.3.4.4b|23.01.2023|GISAID_EpiFlu_Data_Curator|||01.08.2023|||HA|4|A/Sea_Lion/Peru/LIM-SER036/2023_HA|EPI2664215|DNA_IN">
<date value="2023.0630136986301" direction="forwards" units="years"/>
</taxon>
<taxon id="A/Snow_Goose/AB/FAV-0026-1/2022|EPI_ISL_19155037|A_/_H5N1|||2.3.4.4b|02.12.2022|Signore_Anthony|||21.05.2024|Canadian_Food_Inspection_Agency|Canadian_Food_Inspection_Agency|HA|4|A/Snow_Goose/AB/FAV-0026-1/2022_HA|EPI3314976|DNA_IN">
<date value="2022.9205479452055" direction="forwards" units="years"/>
</taxon>
<taxon id="A/South_American_fur_seal/Argentina/RN-PB019/2023|EPI_ISL_18945320|A_/_H5N1|Original||2.3.4.4b|26.08.2023||||01.03

.2024|Instituto_Nacional_de_Tecnologia_Agropecuaria_Instituto_de_Virologia_e_Innovaciones_Tecnologicas||HA|4|A/South_American_fur_seal/Ar">
<date value="2023.6520547945206" direction="forwards" units="years"/>
</taxon>
<taxon id="A/South_American_sea_lion/Argentina/RN-PB007/2023|EPI_ISL_18945319|A_/_H5N1|Original||2.3.4.4b|26.08.2023||||01.03.2024|Instituto_Nacional_de_Tecnologia_Agropecuaria_Instituto_de_Virologia_e_Innovaciones_Tecnologicas||HA|4|A/South_American_sea_lion/Ar">
<date value="2023.6520547945206" direction="forwards" units="years"/>
</taxon>
<taxon id="A/South_American_sea_lion/Argentina/RN-PB011/2023|EPI_ISL_18945317|A_/_H5N1|Original||2.3.4.4b|26.08.2023||||01.03.2024|Instituto_Nacional_de_Tecnologia_Agropecuaria_Instituto_de_Virologia_e_Innovaciones_Tecnologicas||HA|4|A/South_American_sea_lion/Ar">
<date value="2023.6520547945206" direction="forwards" units="years"/>
</taxon>
<taxon id="A/South_American_sea_lion/Argentina/RN-PB013/2023|EPI_ISL_18945316|A_/_H5N1|Original||2.3.4.4b|26.08.2023||||01.03.2024|Instituto_Nacional_de_Tecnologia_Agropecuaria_Instituto_de_Virologia_e_Innovaciones_Tecnologicas||HA|4|A/South_American_sea_lion/Ar">
<date value="2023.6520547945206" direction="forwards" units="years"/>
</taxon>
<taxon id="A/South_American_tern/Argentina/CH-PD030/2023|EPI_ISL_19466158|A_/_H5N1|Original||2.3.4.4b|10.10.2023||||08.10.2024|Instituto_Nacional_de_Tecnologia_Agropecuaria_Instituto_de_Virologia_e_Innovaciones_Tecnologicas||HA|4|A/South_American_tern/Argentina/">
<date value="2023.7205479452055" direction="forwards" units="years"/>
</taxon>
<taxon id="A/South_American_tern/Argentina/CH-PD037/2023|EPI_ISL_19466210|A_/_H5N1|Original||2.3.4.4b|10.10.2023||||08.10.2024|Instituto_Nacional_de_Tecnologia_Agropecuaria_Instituto_de_Virologia_e_Innovaciones_Tecnologicas||HA|4|A/South_American_tern/Argentina/">
<date value="2023.7205479452055" direction="forwards" units="years"/>
</taxon>
<taxon id="A/South_American_tern/Argentina/RN-PB015/2023|EPI_ISL_18945315|A_/_H5N1|Original||2.3.4.4b|26.08.2023||||01.03.2024|Instituto_Nacional_de_Tecnologia_Agropecuaria_Instituto_de_Virologia_e_Innovaciones_Tecnologicas||HA|4|A/South_American_tern/Argentina/">
<date value="2023.6520547945206" direction="forwards" units="years"/>
</taxon>
<taxon id="A/South_American_tern/Maule/238507/2023|EPI_ISL_17885973|A_/_H5N1|Original||2.3.4.4b|23.02.2023|GISAID_EpiFlu_Data_Curator|||29.06.2023|CEIRS_Data_Processing_and_Coordinating_Center_Center_for_Research_on_Influenza_Pathogenesis__CRIP_|CEIRS_Data_Proc">
<date value="2023.1479452054793" direction="forwards" units="years"/>
</taxon>
<taxon id="A/South_Georgia_Shag/King_Edward_Cove/141245/2023|EPI_ISL_18742219|A_/_H5N1|Clinical_Sample||2.3.4.4b|27.11.2023|Mollett_Benjamin|||25.06.2024|Animal_and_Plant_Health_Agency__APHA_|Animal_and_Plant_Health_Agency__APHA_|HA|4|A/South_Georgia_Shag/King_E">
<date value="2023.9068493150685" direction="forwards" units="years"/>
</taxon>
<taxon id="A/South_Georgia_Shag/South_Georgia_and_the_South_Sandwich_Islands/88/2023|EPI_ISL_19313558|A_/_H5N1|Original||2.3.4.4b|27.11.2023||||06.08.2024|Animal_and_Plant_Health_Agency__APHA_||HA|4|A/South_Georgia_Shag/South_Georgia_and_the_South_Sandwich_Island">
<date value="2023.9068493150685" direction="forwards" units="years"/>
</taxon>
<taxon id="A/South_polar_skua/Antarctica/INACH-UC-UCHILE-SKU1/2024|EPI_ISL_19410261|A_/_H5N1|Original||2.3.4.4b|03.03.2024||||12.09.

2024|Emory_University__CEIRR_|Emory_University__CEIRR_|HA|4|A/South_polar_skua/Antarctica/INACH-UC-UCHILE-SKU1/2024_HA|EPI3555175|D">
<date value="2024.0874316939892" direction="forwards" units="years"/>
</taxon>
<taxon id="A/South_polar_skua/Antarctica/INACH-UC-UCHILE-SKU2/2024|EPI_ISL_19410262|A_/_H5N1|Original||2.3.4.4b|03.03.2024||||12.09.2024|Emory_University__CEIRR_|Emory_University__CEIRR_|HA|4|A/South_polar_skua/Antarctica/INACH-UC-UCHILE-SKU2/2024_HA|EPI3555182|D">
<date value="2024.0874316939892" direction="forwards" units="years"/>
</taxon>
<taxon id="A/South_polar_skua/Antarctica/INACH-UC-UCHILE-SKU3/2024|EPI_ISL_19410263|A_/_H5N1|Original||2.3.4.4b|03.03.2024||||12.09.2024|Emory_University__CEIRR_|Emory_University__CEIRR_|HA|4|A/South_polar_skua/Antarctica/INACH-UC-UCHILE-SKU3/2024_HA|EPI3555189|D">
<date value="2024.0874316939892" direction="forwards" units="years"/>
</taxon>
<taxon id="A/South_polar_skua/Antarctica/INACH-UC-UCHILE-SKU4/2024|EPI_ISL_19410264|A_/_H5N1|Original||2.3.4.4b|03.03.2024||||12.09.2024|Emory_University__CEIRR_|Emory_University__CEIRR_|HA|4|A/South_polar_skua/Antarctica/INACH-UC-UCHILE-SKU4/2024_HA|EPI3555196|D">
<date value="2024.0874316939892" direction="forwards" units="years"/>
</taxon>
<taxon id="A/South_polar_skua/Antarctica/INACH-UC-UCHILE-SKU5/2024|EPI_ISL_19410265|A_/_H5N1|Original||2.3.4.4b|03.03.2024||||12.09.2024|Emory_University__CEIRR_|Emory_University__CEIRR_|HA|4|A/South_polar_skua/Antarctica/INACH-UC-UCHILE-SKU5/2024_HA|EPI3555203|D">
<date value="2024.0874316939892" direction="forwards" units="years"/>
</taxon>
<taxon id="A/Southern_Elephant_Seal/Jason_Harbour/141027/2023|EPI_ISL_18742221|A_/_H5N1|Clinical_Sample||2.3.4.4b|09.12.2023|Mollett_Benjamin|||25.06.2024|Animal_and_Plant_Health_Agency__APHA_|Animal_and_Plant_Health_Agency__APHA_|HA|4|A/Southern_Elephant_Seal/J">
<date value="2023.9397260273972" direction="forwards" units="years"/>
</taxon>
<taxon id="A/Southern_Elephant_Seal/Jason_Harbour/141078/2023|EPI_ISL_18742213|A_/_H5N1|Clinical_Sample||2.3.4.4b|09.12.2023|Mollett_Benjamin|||25.06.2024|Animal_and_Plant_Health_Agency__APHA_|Animal_and_Plant_Health_Agency__APHA_|HA|4|A/Southern_Elephant_Seal/J">
<date value="2023.9397260273972" direction="forwards" units="years"/>
</taxon>
<taxon id="A/Southern_Elephant_Seal/South_Georgia_and_the_South_Sandwich_Islands/120/2023|EPI_ISL_19313566|A_/_H5N1|Original||2.3.4.4b|09.12.2023||||06.08.2024|Animal_and_Plant_Health_Agency__APHA_||HA|4|A/Southern_Elephant_Seal/South_Georgia_and_the_South_Sandwi">
<date value="2023.9397260273972" direction="forwards" units="years"/>
</taxon>
<taxon id="A/Southern_Elephant_Seal/South_Georgia_and_the_South_Sandwich_Islands/24/2023|EPI_ISL_19313544|A_/_H5N1|Original||2.3.4.4b|09.12.2023||||06.08.2024|Animal_and_Plant_Health_Agency__APHA_||HA|4|A/Southern_Elephant_Seal/South_Georgia_and_the_South_Sandwic">
<date value="2023.9397260273972" direction="forwards" units="years"/>
</taxon>
<taxon id="A/Southern_Fulmar/South_Georgia_and_the_South_Sandwich_Islands/4/2023|EPI_ISL_19313538|A_/_H5N1|Original||2.3.4.4b|30.10.2023||||06.08.2024|Animal_and_Plant_Health_Agency__APHA_||HA|4|A/Southern_Fulmar/South_Georgia_and_the_South_Sandwich_Islands/4/202">
<date value="2023.7753424657535" direction="forwards" units="years"/>
</taxon>
<taxon id="A/Southern_elephant_seal/Peninsula_Valdes/HA_CH-PD027/2023|EPI_ISL_19466211|A_/_H5N1|Original||2.3.4.4b|10.10.2023||||08.10

.2024|Instituto_Nacional_de_Tecnologia_Agropecuaria_Instituto_de_Virologia_e_Innovaciones_Tecnologicas||HA|4|A/Southern_elephant">
<date value="2023.7205479452055" direction="forwards" units="years"/>
</taxon>
<taxon id="A/Southern_elephant_seal/Peninsula_Valdes/HA_CH-PD032rain/2023|EPI_ISL_19466216|A_/_H5N1|Original||2.3.4.4b|10.10.2023||||08.10.2024|Instituto_Nacional_de_Tecnologia_Agropecuaria_Instituto_de_Virologia_e_Innovaciones_Tecnologicas||HA|4|A/Southern_elep">
<date value="2023.7205479452055" direction="forwards" units="years"/>
</taxon>
<taxon id="A/Southern_fulmar/Falkland_Islands/133789/2023|EPI_ISL_18522961|A_/_H5N1|Clinical_Sample||2.3.4.4b|30.10.2023|Mollett_Benjamin|||20.11.2023|Animal_and_Plant_Health_Agency__APHA_|Animal_and_Plant_Health_Agency__APHA_|HA|4|A/Southern_fulmar/Falkland_Isl">
<date value="2023.7753424657535" direction="forwards" units="years"/>
</taxon>
<taxon id="A/Sterna_hirundo/Espirito_Santo/0155-N/2024|EPI_ISL_19215192|A_/_H5N1|Original||2.3.4.4b|02.02.2024||||25.06.2024|Ministerio_da_Agricultura_e_Pecuaria||HA|4|A/Sterna_hirundo/ItapemirimBR/0155-N/2024_HA|EPI3395164|DNA_IN">
<date value="2024.0928961748634" direction="forwards" units="years"/>
</taxon>
<taxon id="A/Sterna_hirundo/Espirito_Santo/0448-N/2024|EPI_ISL_19215194|A_/_H5N1|Original||2.3.4.4b|02.03.2024||||25.06.2024|Ministerio_da_Agricultura_e_Pecuaria||HA|4|A/Sterna_hirundo/PiumaBR/0448-N/2024_HA|EPI3395180|DNA_IN">
<date value="2024.0846994535518" direction="forwards" units="years"/>
</taxon>
<taxon id="A/Sterna_hirundo/Espirito_Santo/1455-N/2023|EPI_ISL_19215183|A_/_H5N1|Original||2.3.4.4b|27.05.2023||||25.06.2024|Ministerio_da_Agricultura_e_Pecuaria||HA|4|A/Sterna_hirundo/EspiritoSanto/1455-N/2023_HA|EPI3395092|DNA_IN">
<date value="2023.4027397260274" direction="forwards" units="years"/>
</taxon>
<taxon id="A/Sterna_hirundo/Rio_de_Janeiro/0481-R/2024|EPI_ISL_19215196|A_/_H5N1|Original||2.3.4.4b|02.03.2024||||25.06.2024|Ministerio_da_Agricultura_e_Pecuaria||HA|4|A/Sterna_hirundo/SaoFranciscodeItabapoanaBR/0481-R/2024_HA|EPI3395196|DNA_IN">
<date value="2024.0846994535518" direction="forwards" units="years"/>
</taxon>
<taxon id="A/Sterna_hirundo/Rio_de_Janeiro/0721-N/2024|EPI_ISL_19215195|A_/_H5N1|Original||2.3.4.4b|15.03.2024||||25.06.2024|Ministerio_da_Agricultura_e_Pecuaria||HA|4|A/Sterna_hirundo/RiodasOstrasBR/0721-N/2024_HA|EPI3395188|DNA_IN">
<date value="2024.120218579235" direction="forwards" units="years"/>
</taxon>
<taxon id="A/Sterna_hirundo/Santa_Catarina/2261-N/2023|EPI_ISL_19215184|A_/_H5N1|Original||2.3.4.4b|09.11.2023||||25.06.2024|Ministerio_da_Agricultura_e_Pecuaria||HA|4|A/Sterna_hirundo/PenhaBR/2261-N/2023_HA|EPI3395100|DNA_IN">
<date value="2023.8575342465754" direction="forwards" units="years"/>
</taxon>
<taxon id="A/Striped_Skunk/BC/AIVPHL-1080/2023|EPI_ISL_18665585|A_/_H5N1|Original||2.3.4.4b|20.04.2023|Caleta_Jessica_Marija|||21.12.2023|B.C._Centre_for_Disease_Control|Public_Health_Agency_of_Canada|HA|4|A/Striped_Skunk/BC/AIVPHL-1080/2023_HA|EPI2856601|DNA_IN">
<date value="2023.3013698630136" direction="forwards" units="years"/>
</taxon>
<taxon id="A/Striped_Skunk/BC/AIVPHL-1082/2023|EPI_ISL_18665588|A_/_H5N1|Original||2.3.4.4b|20.04.2023|Caleta_Je

ssica_Marija|||21.12.2023|B.C._Centre_for_Disease_Control|Public_Health_Agency_of_Canada|HA|4|A/Striped_Skunk/BC/AIVPHL-1082/2023_HA|EPI2856624|DNA_IN">
<date value="2023.3013698630136" direction="forwards" units="years"/>
</taxon>
<taxon id="A/Striped_Skunk/BC/AIVPHL-1083/2023|EPI_ISL_18665592|A_/_H5N1|Original||2.3.4.4b|20.04.2023|Caleta_Jessica_Marija|||21.12.2023|B.C._Centre_for_Disease_Control|Public_Health_Agency_of_Canada|HA|4|A/Striped_Skunk/BC/AIVPHL-1083/2023_HA|EPI2856648|DNA_IN">
<date value="2023.3013698630136" direction="forwards" units="years"/>
</taxon>
<taxon id="A/Striped_Skunk/BC/AIVPHL-966/2023|EPI_ISL_18665518|A_/_H5N1|Original||2.3.4.4b|28.02.2023|Caleta_Jessica_Marija|||21.12.2023|B.C._Centre_for_Disease_Control|Public_Health_Agency_of_Canada|HA|4|A/Striped_Skunk/BC/AIVPHL-966/2023_HA|EPI2856103|DNA_INS">
<date value="2023.1616438356164" direction="forwards" units="years"/>
</taxon>
<taxon id="A/Sula_leucogaster/Brazil-PR/A0001-IBTEC/2023|EPI_ISL_18975372|A_/_H5N1|Original||2.3.4.4b|25.07.2023|Hurtado_Eliana||AFLU1|10.08.2024|Laboratorio_de_Ecologia_e_Conservacao|Sao_Paulo_State_University_-_Biotechnology_Institute|HA|4|A/Sula_leucogaster/B">
<date value="2023.5643835616438" direction="forwards" units="years"/>
</taxon>
<taxon id="A/Sula_leucogaster/Sao_Francisco_do_Sul/2122-N/2023|EPI_ISL_19215185|A_/_H5N1|Original||2.3.4.4b|27.09.2023||||25.06.2024|Ministerio_da_Agricultura_e_Pecuaria||HA|4|A/Sula_leucogaster/Sao_Francisco_do_Sul/2122-N/2023_HA|EPI3395108|DNA_IN">
<date value="2023.7397260273972" direction="forwards" units="years"/>
</taxon>
<taxon id="A/Swainson_s_hawk/Colorado/23-014329-001/2023|EPI_ISL_17964959|A_/_H5N1|Original||2.3.4.4b|02.05.2023|Killian_Mary_Lea||23-014329-001|06.07.2023|National_Veterinary_Services_Laboratories_-_USDA|National_Veterinary_Services_Laboratories_-_USDA|HA|4|HA_">
<date value="2023.3342465753424" direction="forwards" units="years"/>
</taxon>
<taxon id="A/Thalasseus_acuflavidus/Brazil-ES/23ES1A0009/2023|EPI_ISL_18130622|A_/_H5N1|||2.3.4.4b|21.06.2023|Ferreira_Helena_Lage|Thalasseus_acuflavidus/Brazil-ES/23ES1A0009/2023||24.08.2023|University_of_Sao_Paulo|University_of_Sao_Paulo|HA|4|A/Thalasseus_acuf">
<date value="2023.4712328767123" direction="forwards" units="years"/>
</taxon>
<taxon id="A/Thalasseus_acuflavidus/Brazil-ES/23ES1A0025/2023|EPI_ISL_18130627|A_/_H5N1|||2.3.4.4b|24.06.2023|Ferreira_Helena_Lage|A/Thalasseus_acuflavidus/Brazil-ES/23ES1A0025/2023|A/Thalasseus_acuflavidus/Brazil-ES/23ES1A0025/2023|24.08.2023|University_of_Sao_">
<date value="2023.4794520547946" direction="forwards" units="years"/>
</taxon>
<taxon id="A/Thalasseus_acuflavidus/Parana/2277-N/2023|EPI_ISL_19215199|A_/_H5N1|Original||2.3.4.4b|14.11.2023||||25.06.2024|Ministerio_da_Agricultura_e_Pecuaria||HA|4|A/Thalasseus_acuflavidus/MatinhosBR/2277-N/2023_HA|EPI3395220|DNA_IN">
<date value="2023.8712328767124" direction="forwards" units="years"/>
</taxon>
<taxon id="A/Thalasseus_acuflavidus/Sao_Paulo/0291-N/2024|EPI_ISL_19215197|A_/_H5N1|Original||2.3.4.4b|22.02.2024||||25.06.202

4|Ministerio_da_Agricultura_e_Pecuaria||HA|4|A/Thalasseus_acuflavidus/BertiogaBR/0291-N/2024_HA|EPI3395204|DNA_IN">
<date value="2024.1475409836066" direction="forwards" units="years"/>
</taxon>
<taxon id="A/Thalasseus_acuflavidus/Sao_Paulo/2280-N/2023|EPI_ISL_19215198|A_/_H5N1|Original||2.3.4.4b|06.11.2023||||25.06.2024|Ministerio_da_Agricultura_e_Pecuaria||HA|4|A/Thalasseus_acuflavidus/IlhaCompridaBR/2280-N/2023_HA|EPI3395212|DNA_IN">
<date value="2023.849315068493" direction="forwards" units="years"/>
</taxon>
<taxon id="A/Thalasseus_maximus/Brazil-ES/23ES1A0008/2023|EPI_ISL_18130597|A_/_H5N1|||2.3.4.4b|21.06.2023|Ferreira_Helena_Lage|A/Thalasseus_maximus/Brazil-ES/23ES1A0008/2023|A/Thalasseus_maximus/Brazil-ES/23ES1A0008/2023|24.08.2023|University_of_Sao_Paulo|Univer">
<date value="2023.4712328767123" direction="forwards" units="years"/>
</taxon>
<taxon id="A/Thalasseus_maximus/Parana/1775-N/2023|EPI_ISL_19215186|A_/_H5N1|Original||2.3.4.4b|23.06.2023||||25.06.2024|Ministerio_da_Agricultura_e_Pecuaria||HA|4|A/Thalasseus_maximus/Antonina/1775-N/2023_HA|EPI3395116|DNA_IN">
<date value="2023.4767123287672" direction="forwards" units="years"/>
</taxon>
<taxon id="A/Thalasseus_maximus/Rio_Grande_do_Sul/2177-N/2023|EPI_ISL_19215188|A_/_H5N1|Original||2.3.4.4b|13.10.2023||||25.06.2024|Ministerio_da_Agricultura_e_Pecuaria||HA|4|A/Thalasseus_maximus/RioGrandeSul/2177-N/2023_HA|EPI3395132|DNA_IN">
<date value="2023.7287671232878" direction="forwards" units="years"/>
</taxon>
<taxon id="A/Thalasseus_maximus/Santa_Catarina/1941-N/2023|EPI_ISL_19215187|A_/_H5N1|Original||2.3.4.4b|25.09.2023||||25.06.2024|Ministerio_da_Agricultura_e_Pecuaria||HA|4|A/Thalasseus_maximus/Itapoa/1941-N/2023_HA|EPI3395124|DNA_IN">
<date value="2023.7342465753425" direction="forwards" units="years"/>
</taxon>
<taxon id="A/Thalasseus_maximus/Sao_Paulo/1546-N/2023|EPI_ISL_19215189|A_/_H5N1|Original||2.3.4.4b|05.06.2023||||25.06.2024|Ministerio_da_Agricultura_e_Pecuaria||HA|4|A/Thalasseus_maximus/Ubatuba/1546-N/2023_HA|EPI3395140|DNA_IN">
<date value="2023.427397260274" direction="forwards" units="years"/>
</taxon>
<taxon id="A/Thalasseus_maximus/Sao_Paulo/2339-N/2023|EPI_ISL_19215200|A_/_H5N1|Original||2.3.4.4b|13.12.2023||||25.06.2024|Ministerio_da_Agricultura_e_Pecuaria||HA|4|A/Thalasseus_maximus/PraiaGrandeBR/2339-N/2023_HA|EPI3395228|DNA_IN">
<date value="2023.9506849315069" direction="forwards" units="years"/>
</taxon>
<taxon id="A/Trumpeter_Swan/BC/AIVPHL-1003/2023|EPI_ISL_18665550|A_/_H5N1|Original||2.3.4.4b|20.03.2023|Caleta_Jessica_Marija|||21.12.2023|B.C._Centre_for_Disease_Control|Public_Health_Agency_of_Canada|HA|4|A/Trumpeter_Swan/BC/AIVPHL-1003/2023_HA|EPI2856347|DNA_">
<date value="2023.131506849315" direction="forwards" units="years"/>
</taxon>
<taxon id="A/Trumpeter_Swan/BC/AIVPHL-916/2023|EPI_ISL_18665494|A_/_H5N1|Original||2.3.4.4b|27.01.2023|Caleta_Jessica_Marija|||21.12.2023|B.C._Centre_for_Disease_Control|Public_Health_Agency_of_Canada|HA|4|A/Trumpeter_Swan/BC/AIVPHL-916/2023_HA|EPI2855919|DNA_IN">
<date value="2023.0739726027398" direction="forwards" units="years"/>
</taxon>

<taxon id="A/Trumpeter_Swan/BC/FAV-0053-25/2022|EPI_ISL_19155139|A_/_H5N1|||2.3.4.4b|31.12.2022|Signore_Anthony|||21.05.2024|Canadian_Food_Inspection_Agency|Canadian_Food_Inspection_Agency|HA|4|A/Trumpeter_Swan/BC/FAV-0053-25/2022_HA|EPI3315792|DNA_IN">
<date value="2023.0" direction="forwards" units="years"/>
</taxon>
<taxon id="A/Turkey/Argentina/1348-3/2023|EPI_ISL_18698508|A_/_H5N1|||2.3.4.4b|31.03.2023|Benedetti_Estefania|||28.12.2023|Direccion_del_Laboratorio_Animal_Direccion_General_de_Laboratorios_y_Control_Tecnico_SENASA.|Instituto_Nacional_de_Enfermedades_Infeccios">
<date value="2023.1616438356164" direction="forwards" units="years"/>
</taxon>
<taxon id="A/Turkey/Argentina/1710-1/2023|EPI_ISL_18698513|A_/_H5N1|||2.3.4.4b|17.04.2023|Benedetti_Estefania|||28.12.2023|Direccion_del_Laboratorio_Animal_Direccion_General_de_Laboratorios_y_Control_Tecnico_SENASA.|Instituto_Nacional_de_Enfermedades_Infeccios">
<date value="2023.2931506849316" direction="forwards" units="years"/>
</taxon>
<taxon id="A/Turkey/Argentina/1711-2/2023|EPI_ISL_18698514|A_/_H5N1|||2.3.4.4b|19.04.2023|Benedetti_Estefania|||28.12.2023|Direccion_del_Laboratorio_Animal_Direccion_General_de_Laboratorios_y_Control_Tecnico_SENASA.|Instituto_Nacional_de_Enfermedades_Infeccios">
<date value="2023.2986301369863" direction="forwards" units="years"/>
</taxon>
<taxon id="A/Turkey/Argentina/753-1/2023|EPI_ISL_18698497|A_/_H5N1|||2.3.4.4b|04.03.2023|Benedetti_Estefania|||28.12.2023|Direccion_del_Laboratorio_Animal_Direccion_General_de_Laboratorios_y_Control_Tecnico_SENASA.|Instituto_Nacional_de_Enfermedades_Infecciosa">
<date value="2023.0876712328768" direction="forwards" units="years"/>
</taxon>
<taxon id="A/Turkey/BC/FAV-1613-1/2022|EPI_ISL_19155056|A_/_H5N1|||2.3.4.4b|05.12.2022|Signore_Anthony|||21.05.2024|Canadian_Food_Inspection_Agency|Canadian_Food_Inspection_Agency|HA|4|A/Turkey/BC/FAV-1613-1/2022_HA|EPI3315128|DNA_IN">
<date value="2022.9287671232876" direction="forwards" units="years"/>
</taxon>
<taxon id="A/Turkey/BC/FAV-1613-2/2022|EPI_ISL_19155057|A_/_H5N1|||2.3.4.4b|05.12.2022|Signore_Anthony|||21.05.2024|Canadian_Food_Inspection_Agency|Canadian_Food_Inspection_Agency|HA|4|A/Turkey/BC/FAV-1613-2/2022_HA|EPI3315136|DNA_IN">
<date value="2022.9287671232876" direction="forwards" units="years"/>
</taxon>
<taxon id="A/Turkey_vulture/Antofagasta/228252-1/2022|EPI_ISL_17885970|A_/_H5N1|Original||2.3.4.4b|17.12.2022|GISAID_EpiFlu_Data_Curator|||19.07.2023|CEIRS_Data_Processing_and_Coordinating_Center_Center_for_Research_on_Influenza_Pathogenesis__CRIP_|CEIRS_Data_P">
<date value="2022.9616438356165" direction="forwards" units="years"/>
</taxon>
<taxon id="A/Turkey_vulture/Valparaiso/230187-1/2022|EPI_ISL_17885968|A_/_H5N1|Original||2.3.4.4b|31.12.2022|GISAID_EpiFlu_Data_Curator|||19.07.2023|CEIRS_Data_Processing_and_Coordinating_Center_Center_for_Research_on_Influenza_Pathogenesis__CRIP_|CEIRS_Data_Pr">
<date value="2023.0" direction="forwards" units="years"/>
</taxon>
<taxon id="A/Western_screech_owl/Washington/23-010027-001/2023|EPI_ISL_17964920|A_/_H5N1|Original||2.3.4.4b|23.03.2023|Killian_Mary_Lea||23-010027-

001|06.07.2023|National_Veterinary_Services_Laboratories_-_USDA|National_Veterinary_Services_Laboratories_-_USDA|HA">
<date value="2023.1397260273973" direction="forwards" units="years"/>
</taxon>
<taxon id="A/american_coot/Florida/23-002762-015/2022|EPI_ISL_19606663|A_/_H5N1|Original||2.3.4.4b|19.12.2022||||12.12.2024|USDA_APHIS_Veterinary_Services_Diagnostic_Virology_Laboratory_National_Veterinary_Services_Laboratories||HA|4|A/american_coot/Florida/2">
<date value="2022.9671232876713" direction="forwards" units="years"/>
</taxon>
<taxon id="A/american_crow/BC/AIVPHL-330/2022|EPI_ISL_17051463|A_/_H5N1|Original||2.3.4.4b|19.12.2022|Russell_Shannon_Laurel|||28.02.2023|B.C._Centre_for_Disease_Control|British_Columbia_Centre_for_Disease_Control|HA|4|A/american_crow/BC/AIVPHL-330/2022_HA|EPI24">
<date value="2022.9671232876713" direction="forwards" units="years"/>
</taxon>
<taxon id="A/american_crow/BC/AIVPHL-341/2022|EPI_ISL_17051468|A_/_H5N1|Original||2.3.4.4b|19.12.2022|Russell_Shannon_Laurel|||28.02.2023|B.C._Centre_for_Disease_Control|British_Columbia_Centre_for_Disease_Control|HA|4|A/american_crow/BC/AIVPHL-341/2022_HA|EPI24">
<date value="2022.9671232876713" direction="forwards" units="years"/>
</taxon>
<taxon id="A/american_crow/BC/AIVPHL-345/2022|EPI_ISL_17051473|A_/_H5N1|Original||2.3.4.4b|30.12.2022|Russell_Shannon_Laurel|||28.02.2023|B.C._Centre_for_Disease_Control|British_Columbia_Centre_for_Disease_Control|HA|4|A/american_crow/BC/AIVPHL-345/2022_HA|EPI24">
<date value="2022.9972602739726" direction="forwards" units="years"/>
</taxon>
<taxon id="A/american_crow/BC/AIVPHL-350/2022|EPI_ISL_17051471|A_/_H5N1|Original||2.3.4.4b|30.12.2022|Russell_Shannon_Laurel|||28.02.2023|B.C._Centre_for_Disease_Control|British_Columbia_Centre_for_Disease_Control|HA|4|A/american_crow/BC/AIVPHL-350/2022_HA|EPI24">
<date value="2022.9972602739726" direction="forwards" units="years"/>
</taxon>
<taxon id="A/american_crow/BC/AIVPHL-351/2022|EPI_ISL_17051472|A_/_H5N1|Original||2.3.4.4b|30.12.2022|Russell_Shannon_Laurel|||28.02.2023|B.C._Centre_for_Disease_Control|British_Columbia_Centre_for_Disease_Control|HA|4|A/american_crow/BC/AIVPHL-351/2022_HA|EPI24">
<date value="2022.9972602739726" direction="forwards" units="years"/>
</taxon>
<taxon id="A/american_crow/Colorado/22-042172-002/2022|EPI_ISL_19606664|A_/_H5N1|Original||2.3.4.4b|19.12.2022||||12.12.2024|USDA_APHIS_Veterinary_Services_Diagnostic_Virology_Laboratory_National_Veterinary_Services_Laboratories||HA|4|A/american_crow/Colorado">
<date value="2022.9671232876713" direction="forwards" units="years"/>
</taxon>
<taxon id="A/american_crow/Colorado/23-001476-001/2023|EPI_ISL_19606665|A_/_H5N1|Original||2.3.4.4b|05.01.2023||||12.12.2024|USDA_APHIS_Veterinary_Services_Diagnostic_Virology_Laboratory_National_Veterinary_Services_Laboratories||HA|4|A/american_crow/Colorado">
<date value="2023.013698630137" direction="forwards" units="years"/>
</taxon>
<taxon id="A/american_crow/Kansas/23-002150-001/2022|EPI_ISL_19606646|A_/_H5N1|Original||2.3.4.4b|23.12.2022||||12.12.2024|USDA_APHIS_Veterinary_Services_Diagnostic_Virology_Laboratory_National_Veterinary_Services_Laboratories||HA|4|A/american_crow/Kansas/23-">

<date value="2022.978082191781" direction="forwards" units="years"/>
</taxon>
<taxon id="A/american_green_winged_teal/Oregon/22-040176-004/2022|EPI_ISL_19606698|A_/_H5N1|Original||2.3.4.4b|05.12.2022||||12.12.2024|USDA_APHIS_Veterinary_Services_Diagnostic_Virology_Laboratory_National_Veterinary_Services_Laboratories||HA|4|A/american_gr">
<date value="2022.9287671232876" direction="forwards" units="years"/>
</taxon>
<taxon id="A/american_green_winged_teal/Texas/23-000110-004/2022|EPI_ISL_19606685|A_/_H5N1|Original||2.3.4.4b|04.12.2022||||12.12.2024|USDA_APHIS_Veterinary_Services_Diagnostic_Virology_Laboratory_National_Veterinary_Services_Laboratories||HA|4|A/american_gre">
<date value="2022.9260273972602" direction="forwards" units="years"/>
</taxon>
<taxon id="A/american_wigeon/Alaska/22-041761-007/2022|EPI_ISL_19606535|A_/_H5N1|Original||2.3.4.4b|10.12.2022||||12.12.2024|USDA_APHIS_Veterinary_Services_Diagnostic_Virology_Laboratory_National_Veterinary_Services_Laboratories||HA|4|A/american_wigeon/Alaska">
<date value="2022.9424657534246" direction="forwards" units="years"/>
</taxon>
<taxon id="A/american_wigeon/Florida/22-040720-004/2022|EPI_ISL_19606537|A_/_H5N1|Original||2.3.4.4b|10.12.2022||||12.12.2024|USDA_APHIS_Veterinary_Services_Diagnostic_Virology_Laboratory_National_Veterinary_Services_Laboratories||HA|4|A/american_wigeon/Flori">
<date value="2022.9424657534246" direction="forwards" units="years"/>
</taxon>
<taxon id="A/american_wigeon/Oregon/23-003438-001/2023|EPI_ISL_19606553|A_/_H5N1|Original||2.3.4.4b|07.01.2023||||12.12.2024|USDA_APHIS_Veterinary_Services_Diagnostic_Virology_Laboratory_National_Veterinary_Services_Laboratories||HA|4|A/american_wigeon/Oregon">
<date value="2023.0191780821917" direction="forwards" units="years"/>
</taxon>
<taxon id="A/american_wigeon/Texas/23-003062-016/2023|EPI_ISL_19606613|A_/_H5N1|Original||2.3.4.4b|18.01.2023||||12.12.2024|USDA_APHIS_Veterinary_Services_Diagnostic_Virology_Laboratory_National_Veterinary_Services_Laboratories||HA|4|A/american_wigeon/Texas/2">
<date value="2023.0493150684931" direction="forwards" units="years"/>
</taxon>
<taxon id="A/american_wigeon/Texas/23-003062-020/2023|EPI_ISL_19606614|A_/_H5N1|Original||2.3.4.4b|18.01.2023||||12.12.2024|USDA_APHIS_Veterinary_Services_Diagnostic_Virology_Laboratory_National_Veterinary_Services_Laboratories||HA|4|A/american_wigeon/Texas/2">
<date value="2023.0493150684931" direction="forwards" units="years"/>
</taxon>
<taxon id="A/backyard_chicken/Uruguay/UDELAR-040-M5/2023|EPI_ISL_18310942|A_/_H5N1|Original||2.3.4.4b|03.03.2023|GISAID_EpiFlu_Data_Curator|||26.09.2023|||HA|4|A/backyard_chicken/Uruguay/UDELAR-040-M5/2023_HA|EPI2758873|DNA_IN">
<date value="2023.0849315068492" direction="forwards" units="years"/>
</taxon>
<taxon id="A/backyard_chicken/Uruguay/UDELAR-040-M7/2023|EPI_ISL_18310967|A_/_H5N1|Original||2.3.4.4b|03.03.2023|GISAID_EpiFlu_Data_Curator|||26.09.2023|||HA|4|A/backyard_chicken/Uruguay/UDELAR-040-M7/2023_HA|EPI2758976|DNA_IN">
<date value="2023.0849315068492" direction="forwards" units="years"/>
</taxon>
<taxon id="A/backyard_chicken/Uruguay/UDELAR-047-M1/2023|EPI_ISL_18310966|A_/_H5N1|Original||2.3.4.4b|06.03.2023|GISAID_EpiFlu_Data_Curator|||26.09.2023|||HA|4|A/backyard_chicken/Uruguay/UDELAR-047-M1/2023_HA|EPI2758968|DNA_IN">
<date value="2023.0931506849315" direction="forwards" units="years"/>

</taxon>
<taxon id="A/backyard_chicken/Uruguay/UDELAR-047-M3/2023|EPI_ISL_18310965|A_/_H5N1|Original||2.3.4.4b|06.03.2023|GISAID_EpiFlu_Data_Curator|||26.09.2023|||HA|4|A/backyard_chicken/Uruguay/UDELAR-047-M3/2023_HA|EPI2758960|DNA_IN">
<date value="2023.0931506849315" direction="forwards" units="years"/>
</taxon>
<taxon id="A/backyard_chicken/Uruguay/UDELAR-124-M1/2023|EPI_ISL_18310964|A_/_H5N1|Original||2.3.4.4b|16.04.2023|GISAID_EpiFlu_Data_Curator|||26.09.2023|||HA|4|A/backyard_chicken/Uruguay/UDELAR-124-M1/2023_HA|EPI2758952|DNA_IN">
<date value="2023.2904109589042" direction="forwards" units="years"/>
</taxon>
<taxon id="A/backyard_chicken/Uruguay/UDELAR-127-M1/2023|EPI_ISL_18310963|A_/_H5N1|Original||2.3.4.4b|17.04.2023|GISAID_EpiFlu_Data_Curator|||26.09.2023|||HA|4|A/backyard_chicken/Uruguay/UDELAR-127-M1/2023_HA|EPI2758944|DNA_IN">
<date value="2023.2931506849316" direction="forwards" units="years"/>
</taxon>
<taxon id="A/backyard_chicken/Uruguay/UDELAR-127-M4/2023|EPI_ISL_18310962|A_/_H5N1|Original||2.3.4.4b|17.04.2023|GISAID_EpiFlu_Data_Curator|||26.09.2023|||HA|4|A/backyard_chicken/Uruguay/UDELAR-127-M4/2023_HA|EPI2758936|DNA_IN">
<date value="2023.2931506849316" direction="forwards" units="years"/>
</taxon>
<taxon id="A/backyard_chicken/Uruguay/UDELAR-144-M3/2023|EPI_ISL_18310961|A_/_H5N1|Original||2.3.4.4b|03.05.2023|GISAID_EpiFlu_Data_Curator|||26.09.2023|||HA|4|A/backyard_chicken/Uruguay/UDELAR-144-M3/2023_HA|EPI2758928|DNA_IN">
<date value="2023.33698630137" direction="forwards" units="years"/>
</taxon>
<taxon id="A/backyard_duck/Uruguay/UDELAR-124-M3/2023|EPI_ISL_18310960|A_/_H5N1|Original||2.3.4.4b|16.04.2023|GISAID_EpiFlu_Data_Curator|||26.09.2023|||HA|4|A/backyard_duck/Uruguay/UDELAR-124-M3/2023_HA|EPI2758920|DNA_IN">
<date value="2023.2904109589042" direction="forwards" units="years"/>
</taxon>
<taxon id="A/backyard_turkey/Uruguay/UDELAR-124-M6/2023|EPI_ISL_18310959|A_/_H5N1|Original||2.3.4.4b|16.04.2023|GISAID_EpiFlu_Data_Curator|||26.09.2023|||HA|4|A/backyard_turkey/Uruguay/UDELAR-124-M6/2023_HA|EPI2758912|DNA_IN">
<date value="2023.2904109589042" direction="forwards" units="years"/>
</taxon>
<taxon id="A/band-tailed_gull/Antofagasta/228525-2/2022|EPI_ISL_19131167|A_/_H5N1|Original||2.3.4.4b|19.12.2022||||10.05.2024|CEIRS_Data_Processing_and_Coordinating_Center_Center_for_Research_on_Influenza_Pathogenesis__CRIP_|CEIRS_Data_Processing_and_Coordinatin">
<date value="2022.9671232876713" direction="forwards" units="years"/>
</taxon>
<taxon id="A/band-tailed_gull/Tarapaca/238807-1/2023|EPI_ISL_19131176|A_/_H5N1|Original||2.3.4.4b|25.02.2023||||10.05.2024|CEIRS_Data_Processing_and_Coordinating_Center_Center_for_Research_on_Influenza_Pathogenesis__CRIP_||HA|4|A/band-tailed_gull/Tarapaca/238807">
<date value="2023.1534246575343" direction="forwards" units="years"/>
</taxon>
<taxon id="A/barn_owl/Washington/22-041081-001/2022|EPI_ISL_19607175|A_/_H5N1|Original||2.3.4.4b|12.12.2022||||12.12.2024|USDA_APHIS_Veterinary_Services_Diagnostic_Virology_Laboratory_National_Veterinary_Services_Laboratories||HA|4|A/barn_owl/Washington/22-04">
<date value="2022.9479452054795" direction="forwards" units="years"/>
</taxon>

<taxon id="A/barred_owl/BC/AIVPHL-
346/2022|EPI_ISL_17051474|A_/_H5N1|Original||2.3.4.4b|30.12.2022|Russell_Sh
annon_Laurel|||28.02.2023|B.C._Centre_for_Disease_Control|British_Columbia_
Centre_for_Disease_Control|HA|4|A/barred_owl/BC/AIVPHL-
346/2022_HA|EPI2429224|">
<date value="2022.9972602739726" direction="forwards" units="years"/>
</taxon>
<taxon id="A/black-necked_swan/Los_Rios/247292-
1/2023|EPI_ISL_18760070|A_/_H5N1|Original||2.3.4.4b|05.04.2023||||14.01.202
4|Emory_University__CEIRR_|Emory_University__CEIRR_|HA|4|A/black-
necked_swan/Los_Rios/247292-1/2023_HA|EPI2913251|DNA_IN">
<date value="2023.2602739726028" direction="forwards" units="years"/>
</taxon>
<taxon id="A/black-necked_swan/Uruguay/UDELAR-014-
M3/2023|EPI_ISL_18310958|A_/_H5N1|Original||2.3.4.4b|18.02.2023|GISAID_EpiF
lu_Data_Curator|||26.09.2023|||HA|4|A/black-necked_swan/Uruguay/UDELAR-014-
M3/2023_HA|EPI2758904|DNA_IN">
<date value="2023.1342465753426" direction="forwards" units="years"/>
</taxon>
<taxon id="A/black-necked_swan/Uruguay/UDELAR-078-
M2/2023|EPI_ISL_18310957|A_/_H5N1|Original||2.3.4.4b|15.03.2023|GISAID_EpiF
lu_Data_Curator|||26.09.2023|||HA|4|A/black-necked_swan/Uruguay/UDELAR-078-
M2/2023_HA|EPI2758896|DNA_IN">
<date value="2023.1178082191782" direction="forwards" units="years"/>
</taxon>
<taxon id="A/black_brant/California/23-000279-
001/2022|EPI_ISL_19606469|A_/_H5N1|Original||2.3.4.4b|15.12.2022||||12.12.2
024|USDA_APHIS_Veterinary_Services_Diagnostic_Virology_Laboratory_National_
Veterinary_Services_Laboratories||HA|4|A/black_brant/California">
<date value="2022.9561643835616" direction="forwards" units="years"/>
</taxon>
<taxon id="A/black_skimmer/Chile/C61962/2022|EPI_ISL_16891402|A_/_H5N1|||2.
3.4.4b|02.12.2022||||04.02.2023|St._Jude_Center_of_Excellence_for_Influenza
_Research_and_Response_-
_CEIRR_Influenza_Data_Processing_and_Communication_Center|St._Jude_Center_o
f_Excellence_f">
<date value="2022.9205479452055" direction="forwards" units="years"/>
</taxon>
<taxon id="A/brown-hooded_gull/Los_Rios/247093-
1/2023|EPI_ISL_18760071|A_/_H5N1|Original||2.3.4.4b|04.04.2023||||14.01.202
4|Emory_University__CEIRR_|Emory_University__CEIRR_|HA|4|A/brown-
hooded_gull/Los_Rios/247093-1/2023_HA|EPI2913258|DNA_IN">
<date value="2023.2575342465752" direction="forwards" units="years"/>
</taxon>
<taxon id="A/brown-hooded_gull/Los_Rios/247094-
1/2023|EPI_ISL_18760072|A_/_H5N1|Original||2.3.4.4b|04.04.2023||||14.01.202
4|Emory_University__CEIRR_|Emory_University__CEIRR_|HA|4|A/brown-
hooded_gull/Los_Rios/247094-1/2023_HA|EPI2913263|DNA_IN">
<date value="2023.2575342465752" direction="forwards" units="years"/>
</taxon>
<taxon id="A/brown_pelican/Panama/22-041124-
001/2022|EPI_ISL_17559276|A_/_H5N1|Original||2.3.4.4b|14.12.2022|Killian_Ma
ry_Lea|M1_LEA-32-22|22-041124-
001|27.04.2023|El_Laboratorio_de_Diagnostico_de_Enfermedades_Vesiculares__L
ADIVES_|National_Veterinary_Services_L">
<date value="2022.9534246575342" direction="forwards" units="years"/>
</taxon>
<taxon id="A/buff-necked_ibis/Bio_bio/247636-
1/2023|EPI_ISL_19418491|A_/_H5N1|Original||2.3.4.4b|07.04.2023||||17.09.202
4|Emory_University__CEIRR_|Emory_University__CEIRR_|HA|4|A/buff-
necked_ibis/Bio_bio/247636-1/2023_HA|EPI3558280|DNA_IN">

<date value="2023.2657534246575" direction="forwards" units="years"/>
</taxon>
<taxon id="A/bufflehead/California/23-001056-003/2022|EPI_ISL_19606428|A_/_H5N1|Original||2.3.4.4b|21.12.2022||||12.12.2024|USDA_APHIS_Veterinary_Services_Diagnostic_Virology_Laboratory_National_Veterinary_Services_Laboratories||HA|4|A/bufflehead/California/2">
<date value="2022.972602739726" direction="forwards" units="years"/>
</taxon>
<taxon id="A/burmeisters_porpoise/Chile/246506-2/2023|EPI_ISL_19391458|A_/_H5N1|Original||2.3.4.4b|31.03.2023||||09.09.2024|CEIRS_Data_Processing_and_Coordinating_Center_Center_for_Research_on_Influenza_Pathogenesis__CRIP_||HA|4|A/burmeisters_porpoise/Chile/2465">
<date value="2023.1616438356164" direction="forwards" units="years"/>
</taxon>
<taxon id="A/cackling_goose/BC/AIVPHL-367/2023|EPI_ISL_17051485|A_/_H5N1|Original||2.3.4.4b|16.01.2023|Russell_Shannon_Laurel|||28.02.2023|B.C._Centre_for_Disease_Control|British_Columbia_Centre_for_Disease_Control|HA|4|A/cackling_goose/BC/AIVPHL-367/2023_HA|EPI">
<date value="2023.0438356164384" direction="forwards" units="years"/>
</taxon>
<taxon id="A/cackling_goose/California/23-001053-001/2022|EPI_ISL_19606412|A_/_H5N1|Original||2.3.4.4b|17.12.2022||||12.12.2024|USDA_APHIS_Veterinary_Services_Diagnostic_Virology_Laboratory_National_Veterinary_Services_Laboratories||HA|4|A/cackling_goose/Cali">
<date value="2022.9616438356165" direction="forwards" units="years"/>
</taxon>
<taxon id="A/cackling_goose/Colorado/22-040752-002/2022|EPI_ISL_19606415|A_/_H5N1|Original||2.3.4.4b|13.12.2022||||12.12.2024|USDA_APHIS_Veterinary_Services_Diagnostic_Virology_Laboratory_National_Veterinary_Services_Laboratories||HA|4|A/cackling_goose/Colora">
<date value="2022.9506849315069" direction="forwards" units="years"/>
</taxon>
<taxon id="A/cackling_goose/Colorado/22-040753-001/2022|EPI_ISL_19606417|A_/_H5N1|Original||2.3.4.4b|13.12.2022||||12.12.2024|USDA_APHIS_Veterinary_Services_Diagnostic_Virology_Laboratory_National_Veterinary_Services_Laboratories||HA|4|A/cackling_goose/Colora">
<date value="2022.9506849315069" direction="forwards" units="years"/>
</taxon>
<taxon id="A/cackling_goose/Colorado/22-040763-001/2022|EPI_ISL_19606419|A_/_H5N1|Original||2.3.4.4b|10.12.2022||||12.12.2024|USDA_APHIS_Veterinary_Services_Diagnostic_Virology_Laboratory_National_Veterinary_Services_Laboratories||HA|4|A/cackling_goose/Colora">
<date value="2022.9424657534246" direction="forwards" units="years"/>
</taxon>
<taxon id="A/cackling_goose/Colorado/22-040763-003/2022|EPI_ISL_19606420|A_/_H5N1|Original||2.3.4.4b|10.12.2022||||12.12.2024|USDA_APHIS_Veterinary_Services_Diagnostic_Virology_Laboratory_National_Veterinary_Services_Laboratories||HA|4|A/cackling_goose/Colora">
<date value="2022.9424657534246" direction="forwards" units="years"/>
</taxon>
<taxon id="A/cackling_goose/Texas/23-001028-001/2023|EPI_ISL_19606425|A_/_H5N1|Original||2.3.4.4b|06.01.2023||||12.12.2024|USDA_APHIS_Veterinary_Services_Diagnostic_Virology_Laboratory_National_Veterinary_Services_Laboratories||HA|4|A/cackling_goose/Texas/23-">
<date value="2023.0164383561644" direction="forwards" units="years"/>
</taxon>
<taxon id="A/canada_goose/Alaska/22-041059-001/2022|EPI_ISL_19606393|A_/_H5N1|Original||2.3.4.4b|13.12.2022||||12.12.2024|USDA_APHIS_Veterinary_Services_Diagnostic_Virology_Laboratory_National_Veterinary_Services_Laboratories||HA|4|A/canada_goose/Alaska/22-04">

<date value="2022.9506849315069" direction="forwards" units="years"/>
</taxon>
<taxon id="A/canada_goose/BC/AIVPHL-321/2022|EPI_ISL_17051438|A_/_H5N1|Original||2.3.4.4b|08.12.2022|Russell_Shannon_Laurel|||28.02.2023|B.C._Centre_for_Disease_Control|British_Columbia_Centre_for_Disease_Control|HA|4|A/canada_goose/BC/AIVPHL-321/2022_HA|EPI2429">
<date value="2022.9369863013699" direction="forwards" units="years"/>
</taxon>
<taxon id="A/canada_goose/BC/AIVPHL-326/2022|EPI_ISL_17051457|A_/_H5N1|Original||2.3.4.4b|12.12.2022|Russell_Shannon_Laurel|||28.02.2023|B.C._Centre_for_Disease_Control|British_Columbia_Centre_for_Disease_Control|HA|4|A/canada_goose/BC/AIVPHL-326/2022_HA|EPI2429">
<date value="2022.9479452054795" direction="forwards" units="years"/>
</taxon>
<taxon id="A/canada_goose/California/22-041399-001/2022|EPI_ISL_19607747|A_/_H5N1|Original||2.3.4.4b|01.12.2022||||12.12.2024|USDA_APHIS_Veterinary_Services_Diagnostic_Virology_Laboratory_National_Veterinary_Services_Laboratories||HA|4|A/canada_goose/Californ">
<date value="2022.9178082191781" direction="forwards" units="years"/>
</taxon>
<taxon id="A/canada_goose/California/22-041399-002/2022|EPI_ISL_19607748|A_/_H5N1|Original||2.3.4.4b|01.12.2022||||12.12.2024|USDA_APHIS_Veterinary_Services_Diagnostic_Virology_Laboratory_National_Veterinary_Services_Laboratories||HA|4|A/canada_goose/Californ">
<date value="2022.9178082191781" direction="forwards" units="years"/>
</taxon>
<taxon id="A/canada_goose/Colorado/22-040763-002/2022|EPI_ISL_19607758|A_/_H5N1|Original||2.3.4.4b|11.12.2022||||12.12.2024|USDA_APHIS_Veterinary_Services_Diagnostic_Virology_Laboratory_National_Veterinary_Services_Laboratories||HA|4|A/canada_goose/Colorado/2">
<date value="2022.945205479452" direction="forwards" units="years"/>
</taxon>
<taxon id="A/canada_goose/Colorado/22-041829-001/2022|EPI_ISL_19607759|A_/_H5N1|Original||2.3.4.4b|19.12.2022||||12.12.2024|USDA_APHIS_Veterinary_Services_Diagnostic_Virology_Laboratory_National_Veterinary_Services_Laboratories||HA|4|A/canada_goose/Colorado/2">
<date value="2022.9671232876713" direction="forwards" units="years"/>
</taxon>
<taxon id="A/canada_goose/Colorado/22-042165-001/2022|EPI_ISL_19607760|A_/_H5N1|Original||2.3.4.4b|24.12.2022||||12.12.2024|USDA_APHIS_Veterinary_Services_Diagnostic_Virology_Laboratory_National_Veterinary_Services_Laboratories||HA|4|A/canada_goose/Colorado/2">
<date value="2022.9808219178083" direction="forwards" units="years"/>
</taxon>
<taxon id="A/canada_goose/Colorado/22-042169-001/2022|EPI_ISL_19607762|A_/_H5N1|Original||2.3.4.4b|27.12.2022||||12.12.2024|USDA_APHIS_Veterinary_Services_Diagnostic_Virology_Laboratory_National_Veterinary_Services_Laboratories||HA|4|A/canada_goose/Colorado/2">
<date value="2022.9890410958903" direction="forwards" units="years"/>
</taxon>
<taxon id="A/canada_goose/Colorado/23-000399-001/2022|EPI_ISL_19607792|A_/_H5N1|Original||2.3.4.4b|30.12.2022||||12.12.2024|USDA_APHIS_Veterinary_Services_Diagnostic_Virology_Laboratory_National_Veterinary_Services_Laboratories||HA|4|A/canada_goose/Colorado/2">
<date value="2022.9972602739726" direction="forwards" units="years"/>
</taxon>
<taxon id="A/canada_goose/Colorado/23-000496-001/2023|EPI_ISL_19607863|A_/_H5N1|Original||2.3.4.4b|04.01.2023||||12.12.2

024|USDA_APHIS_Veterinary_Services_Diagnostic_Virology_Laboratory_National_Veterinary_Services_Laboratories||HA|4|A/canada_goose/Colorado/2">
<date value="2023.0109589041097" direction="forwards" units="years"/>
</taxon>
<taxon id="A/canada_goose/Colorado/23-000497-001/2023|EPI_ISL_19607793|A_/_H5N1|Original||2.3.4.4b|01.01.2023||||12.12.2024|USDA_APHIS_Veterinary_Services_Diagnostic_Virology_Laboratory_National_Veterinary_Services_Laboratories||HA|4|A/canada_goose/Colorado/2">
<date value="2023.0027397260274" direction="forwards" units="years"/>
</taxon>
<taxon id="A/canada_goose/Colorado/23-000621-001/2023|EPI_ISL_19607820|A_/_H5N1|Original||2.3.4.4b|04.01.2023||||12.12.2024|USDA_APHIS_Veterinary_Services_Diagnostic_Virology_Laboratory_National_Veterinary_Services_Laboratories||HA|4|A/canada_goose/Colorado/2">
<date value="2023.0109589041097" direction="forwards" units="years"/>
</taxon>
<taxon id="A/canada_goose/Colorado/23-000622-001/2023|EPI_ISL_19607821|A_/_H5N1|Original||2.3.4.4b|04.01.2023||||12.12.2024|USDA_APHIS_Veterinary_Services_Diagnostic_Virology_Laboratory_National_Veterinary_Services_Laboratories||HA|4|A/canada_goose/Colorado/2">
<date value="2023.0109589041097" direction="forwards" units="years"/>
</taxon>
<taxon id="A/canada_goose/Colorado/23-000623-001/2023|EPI_ISL_19607822|A_/_H5N1|Original||2.3.4.4b|03.01.2023||||12.12.2024|USDA_APHIS_Veterinary_Services_Diagnostic_Virology_Laboratory_National_Veterinary_Services_Laboratories||HA|4|A/canada_goose/Colorado/2">
<date value="2023.0082191780823" direction="forwards" units="years"/>
</taxon>
<taxon id="A/canada_goose/Colorado/23-003283-001/2023|EPI_ISL_19607823|A_/_H5N1|Original||2.3.4.4b|19.01.2023||||12.12.2024|USDA_APHIS_Veterinary_Services_Diagnostic_Virology_Laboratory_National_Veterinary_Services_Laboratories||HA|4|A/canada_goose/Colorado/2">
<date value="2023.0520547945205" direction="forwards" units="years"/>
</taxon>
<taxon id="A/canada_goose/Colorado/23-003284-001/2023|EPI_ISL_19607824|A_/_H5N1|Original||2.3.4.4b|20.01.2023||||12.12.2024|USDA_APHIS_Veterinary_Services_Diagnostic_Virology_Laboratory_National_Veterinary_Services_Laboratories||HA|4|A/canada_goose/Colorado/2">
<date value="2023.054794520548" direction="forwards" units="years"/>
</taxon>
<taxon id="A/canada_goose/Iowa/22-040790-001/2022|EPI_ISL_19607832|A_/_H5N1|Original||2.3.4.4b|07.12.2022||||12.12.2024|USDA_APHIS_Veterinary_Services_Diagnostic_Virology_Laboratory_National_Veterinary_Services_Laboratories||HA|4|A/canada_goose/Iowa/22-040790">
<date value="2022.9342465753425" direction="forwards" units="years"/>
</taxon>
<taxon id="A/canada_goose/Iowa/22-040790-003/2022|EPI_ISL_19607835|A_/_H5N1|Original||2.3.4.4b|07.12.2022||||12.12.2024|USDA_APHIS_Veterinary_Services_Diagnostic_Virology_Laboratory_National_Veterinary_Services_Laboratories||HA|4|A/canada_goose/Iowa/22-040790">
<date value="2022.9342465753425" direction="forwards" units="years"/>
</taxon>
<taxon id="A/canada_goose/Iowa/22-040793-004/2022|EPI_ISL_19607846|A_/_H5N1|Original||2.3.4.4b|06.12.2022||||12.12.2024|USDA_APHIS_Veterinary_Services_Diagnostic_Virology_Laboratory_National_Veterinary_Services_Laboratories||HA|4|A/canada_goose/Iowa/22-040793">
<date value="2022.9315068493152" direction="forwards" units="years"/>
</taxon>
<taxon id="A/canada_goose/Minnesota/22-041306-001/2022|EPI_ISL_19607675|A_/_H5N1|Original||2.3.4.4b|19.12.2022||||12.12.2

024|USDA_APHIS_Veterinary_Services_Diagnostic_Virology_Laboratory_National_Veterinary_Services_Laboratories||HA|4|A/canada_goose/Minnesota">
<date value="2022.9671232876713" direction="forwards" units="years"/>
</taxon>
<taxon id="A/canada_goose/Nebraska/23-001495-001/2023|EPI_ISL_19607639|A_/_H5N1|Original||2.3.4.4b|10.01.2023||||12.12.2024|USDA_APHIS_Veterinary_Services_Diagnostic_Virology_Laboratory_National_Veterinary_Services_Laboratories||HA|4|A/canada_goose/Nebraska/2">
<date value="2023.027397260274" direction="forwards" units="years"/>
</taxon>
<taxon id="A/canada_goose/Nebraska/23-001495-002/2023|EPI_ISL_19607640|A_/_H5N1|Original||2.3.4.4b|10.01.2023||||12.12.2024|USDA_APHIS_Veterinary_Services_Diagnostic_Virology_Laboratory_National_Veterinary_Services_Laboratories||HA|4|A/canada_goose/Nebraska/2">
<date value="2023.027397260274" direction="forwards" units="years"/>
</taxon>
<taxon id="A/canada_goose/Wyoming/23-000980-001/2023|EPI_ISL_19608051|A_/_H5N1|Original||2.3.4.4b|05.01.2023||||12.12.2024|USDA_APHIS_Veterinary_Services_Diagnostic_Virology_Laboratory_National_Veterinary_Services_Laboratories||HA|4|A/canada_goose/Wyoming/23-">
<date value="2023.013698630137" direction="forwards" units="years"/>
</taxon>
<taxon id="A/canada_goose/Wyoming/23-002120-001/2023|EPI_ISL_19608054|A_/_H5N1|Original||2.3.4.4b|11.01.2023||||12.12.2024|USDA_APHIS_Veterinary_Services_Diagnostic_Virology_Laboratory_National_Veterinary_Services_Laboratories||HA|4|A/canada_goose/Wyoming/23-">
<date value="2023.0301369863014" direction="forwards" units="years"/>
</taxon>
<taxon id="A/caracara/Coquimbo/SJCEIRR-2423261/2023|EPI_ISL_18760065|A_/_H5N1|Original||2.3.4.4b|15.03.2023||||14.01.2024|Emory_University__CEIRR_|Emory_University__CEIRR_|HA|4|A/caracara/Coquimbo/SJCEIRR-2423261/2023|EPI2913216|DNA_IN">
<date value="2023.1178082191782" direction="forwards" units="years"/>
</taxon>
<taxon id="A/caspian_tern/Washington/23-024996-001/2023|EPI_ISL_18737481|A_/_H5N1|Original||2.3.4.4b|07.08.2023|Killian_Mary_Lea|23-024996-001||09.01.2024|National_Veterinary_Services_Laboratories_-_USDA|National_Veterinary_Services_Laboratories_-_USDA|HA|4|HA_A">
<date value="2023.6" direction="forwards" units="years"/>
</taxon>
<taxon id="A/caspian_tern/Washington/23-025001-001/2023|EPI_ISL_18737454|A_/_H5N1|Original||2.3.4.4b|11.08.2023|Killian_Mary_Lea|23-025001-001||09.01.2024|National_Veterinary_Services_Laboratories_-_USDA|National_Veterinary_Services_Laboratories_-_USDA|HA|4|HA_A">
<date value="2023.6109589041096" direction="forwards" units="years"/>
</taxon>
<taxon id="A/caspian_tern/Washington/23-025001-004/2023|EPI_ISL_18737472|A_/_H5N1|Original||2.3.4.4b|11.08.2023|Killian_Mary_Lea|23-025001-004||09.01.2024|National_Veterinary_Services_Laboratories_-_USDA|National_Veterinary_Services_Laboratories_-_USDA|HA|4|HA_A">
<date value="2023.6109589041096" direction="forwards" units="years"/>
</taxon>
<taxon id="A/cat/Nebraska/23-010184-001/2023|EPI_ISL_17821770|A_/_H5N1|Original||2.3.4.4b|30.03.2023|Killian_Mary_Lea||23-010184-001|21.06.2023|Nebraska_Veterinary_Diagnostic_Center|National_Veterinary_Services_Laboratories_-_USDA|HA|4|A/cat/Nebraska/23-010184-0">
<date value="2023.158904109589" direction="forwards" units="years"/>

</taxon>
<taxon id="A/chicken/Araucania/239189-
2/2023|EPI_ISL_17885958|A_/_H5N1|Original||2.3.4.4b|28.02.2023|GISAID_EpiFl
u_Data_Curator|||24.07.2023|CEIRS_Data_Processing_and_Coordinating_Center_C
enter_for_Research_on_Influenza_Pathogenesis__CRIP_|CEIRS_Data_Processing">
<date value="2023.1616438356164" direction="forwards" units="years"/>
</taxon>
<taxon id="A/chicken/Araucania/239569-
1/2023|EPI_ISL_17885954|A_/_H5N1|Original||2.3.4.4b|02.03.2023|GISAID_EpiFl
u_Data_Curator|||24.07.2023|CEIRS_Data_Processing_and_Coordinating_Center_C
enter_for_Research_on_Influenza_Pathogenesis__CRIP_|CEIRS_Data_Processing">
<date value="2023.0821917808219" direction="forwards" units="years"/>
</taxon>
<taxon id="A/chicken/Araucania/239569-
2/2023|EPI_ISL_17885952|A_/_H5N1|Original||2.3.4.4b|02.03.2023|GISAID_EpiFl
u_Data_Curator|||24.07.2023|CEIRS_Data_Processing_and_Coordinating_Center_C
enter_for_Research_on_Influenza_Pathogenesis__CRIP_|CEIRS_Data_Processing">
<date value="2023.0821917808219" direction="forwards" units="years"/>
</taxon>
<taxon id="A/chicken/Araucania/240481-
1/2023|EPI_ISL_17885949|A_/_H5N1|Original||2.3.4.4b|07.03.2023|GISAID_EpiFl
u_Data_Curator|||24.07.2023|CEIRS_Data_Processing_and_Coordinating_Center_C
enter_for_Research_on_Influenza_Pathogenesis__CRIP_|CEIRS_Data_Processing">
<date value="2023.0958904109589" direction="forwards" units="years"/>
</taxon>
<taxon id="A/chicken/Araucania/241892-
2/2023|EPI_ISL_17885946|A_/_H5N1|Original||2.3.4.4b|14.03.2023|GISAID_EpiFl
u_Data_Curator|||24.07.2023|CEIRS_Data_Processing_and_Coordinating_Center_C
enter_for_Research_on_Influenza_Pathogenesis__CRIP_|CEIRS_Data_Processing">
<date value="2023.1150684931506" direction="forwards" units="years"/>
</taxon>
<taxon id="A/chicken/Araucania/241914-
1/2023|EPI_ISL_17885945|A_/_H5N1|Original||2.3.4.4b|14.03.2023|GISAID_EpiFl
u_Data_Curator|||24.07.2023|CEIRS_Data_Processing_and_Coordinating_Center_C
enter_for_Research_on_Influenza_Pathogenesis__CRIP_|CEIRS_Data_Processing">
<date value="2023.1150684931506" direction="forwards" units="years"/>
</taxon>
<taxon id="A/chicken/Araucania/244469/2023|EPI_ISL_18760073|A_/_H5N1|Origin
al||2.3.4.4b|25.03.2023||||14.01.2024|Emory_University__CEIRR_|Emory_Univer
sity__CEIRR_|HA|4|A/chicken/Araucania/244469/2023_HA|EPI2913271|DNA_IN">
<date value="2023.145205479452" direction="forwards" units="years"/>
</taxon>
<taxon id="A/chicken/Atacama/235254-
2/2023|EPI_ISL_18760074|A_/_H5N1|Original||2.3.4.4b|04.02.2023||||14.01.202
4|Emory_University__CEIRR_|Emory_University__CEIRR_|HA|4|A/chicken/Atacama/
235254-2/2023_HA|EPI2913279|DNA_IN">
<date value="2023.0958904109589" direction="forwards" units="years"/>
</taxon>
<taxon id="A/chicken/Aysen/250755-
2/2023|EPI_ISL_19404916|A_/_H5N1|Original||2.3.4.4b|19.04.2023||||13.09.202
4|Emory_University__CEIRR_|Emory_University__CEIRR_|HA|4|A/chicken/Aysen/25
0755-2/2023_HA|EPI3550214|DNA_IN">
<date value="2023.2986301369863" direction="forwards" units="years"/>
</taxon>
<taxon id="A/chicken/Aysen/SJCEIRR-
2477921/2023|EPI_ISL_18760064|A_/_H5N1|Original||2.3.4.4b|09.04.2023||||14.
01.2024|Emory_University__CEIRR_|Emory_University__CEIRR_|HA|4|A/chicken/Ay
sen/SJCEIRR-2477921/2023|EPI2913211|DNA_IN">
<date value="2023.2712328767122" direction="forwards" units="years"/>
</taxon>

<taxon id="A/chicken/Aysen/SJCEIRR-
2477922/2023|EPI_ISL_18760068|A_/_H5N1|Original||2.3.4.4b|09.04.2023||||14.
01.2024|Emory_University__CEIRR_|Emory_University__CEIRR_|HA|4|A/chicken/Ay
sen/SJCEIRR-2477922/2023|EPI2913236|DNA_IN">
<date value="2023.2712328767122" direction="forwards" units="years"/>
</taxon>
<taxon id="A/chicken/Cochabamba/39356/2023|EPI_ISL_19410267|A_/_H5N1|Origin
al||2.3.4.4b|26.01.2023||||13.09.2024|Emory_University__CEIRR_||HA|4|A/chic
ken/Cochabamba/39356/2023_HA|EPI3555217|DNA_IN">
<date value="2023.0712328767124" direction="forwards" units="years"/>
</taxon>
<taxon id="A/chicken/Cochabamba/39383/2023|EPI_ISL_19410268|A_/_H5N1|Origin
al||2.3.4.4b|01.02.2023||||13.09.2024|Emory_University__CEIRR_||HA|4|A/chic
ken/Cochabamba/39383/2023_HA|EPI3555224|DNA_IN">
<date value="2023.0876712328768" direction="forwards" units="years"/>
</taxon>
<taxon id="A/chicken/Cochabamba/39391/2023|EPI_ISL_19410294|A_/_H5N1|Origin
al||2.3.4.4b|02.02.2023||||12.09.2024|Emory_University__CEIRR_||HA|4|A/chic
ken/Cochabamba/39391/2023_HA|EPI3555390|DNA_IN">
<date value="2023.0904109589042" direction="forwards" units="years"/>
</taxon>
<taxon id="A/chicken/Cochabamba/39404/2023|EPI_ISL_19410295|A_/_H5N1|Origin
al||2.3.4.4b|03.02.2023||||13.09.2024|Emory_University__CEIRR_||HA|4|A/chic
ken/Cochabamba/39404/2023_HA|EPI3555395|DNA_IN">
<date value="2023.0931506849315" direction="forwards" units="years"/>
</taxon>
<taxon id="A/chicken/Cochabamba/39425/2023|EPI_ISL_19410269|A_/_H5N1|Origin
al||2.3.4.4b|07.02.2023||||12.09.2024|Emory_University__CEIRR_||HA|4|A/chic
ken/Cochabamba/39425/2023_HA|EPI3555231|DNA_IN">
<date value="2023.1041095890412" direction="forwards" units="years"/>
</taxon>
<taxon id="A/chicken/Cochabamba/39472/2023|EPI_ISL_19410270|A_/_H5N1|Origin
al||2.3.4.4b|17.02.2023||||13.09.2024|Emory_University__CEIRR_||HA|4|A/chic
ken/Cochabamba/39472/2023_HA|EPI3555237|DNA_IN">
<date value="2023.131506849315" direction="forwards" units="years"/>
</taxon>
<taxon id="A/chicken/Cochabamba/39476/2023|EPI_ISL_19410271|A_/_H5N1|Origin
al||2.3.4.4b|18.02.2023||||13.09.2024|Emory_University__CEIRR_||HA|4|A/chic
ken/Cochabamba/39476/2023_HA|EPI3555242|DNA_IN">
<date value="2023.1342465753426" direction="forwards" units="years"/>
</taxon>
<taxon id="A/chicken/Cochabamba/39507/2023|EPI_ISL_19410272|A_/_H5N1|Origin
al||2.3.4.4b|01.03.2023||||13.09.2024|Emory_University__CEIRR_||HA|4|A/chic
ken/Cochabamba/39507/2023_HA|EPI3555249|DNA_IN">
<date value="2023.0794520547945" direction="forwards" units="years"/>
</taxon>
<taxon id="A/chicken/Cochabamba/39521/2023|EPI_ISL_19410296|A_/_H5N1|Origin
al||2.3.4.4b|03.03.2023||||13.09.2024|Emory_University__CEIRR_||HA|4|A/chic
ken/Cochabamba/39521/2023_HA|EPI3555401|DNA_IN">
<date value="2023.0849315068492" direction="forwards" units="years"/>
</taxon>
<taxon id="A/chicken/Cochabamba/39522/2023|EPI_ISL_19410273|A_/_H5N1|Origin
al||2.3.4.4b|03.03.2023||||12.09.2024|Emory_University__CEIRR_||HA|4|A/chic
ken/Cochabamba/39522/2023_HA|EPI3555255|DNA_IN">
<date value="2023.0849315068492" direction="forwards" units="years"/>
</taxon>
<taxon id="A/chicken/Cochabamba/39538/2023|EPI_ISL_19410274|A_/_H5N1|Origin
al||2.3.4.4b|03.03.2023||||13.09.2024|Emory_University__CEIRR_||HA|4|A/chic
ken/Cochabamba/39538/2023_HA|EPI3555259|DNA_IN">
<date value="2023.0849315068492" direction="forwards" units="years"/>
</taxon>

<taxon id="A/chicken/Cochabamba/39539/2023|EPI_ISL_19418487|A_/_H5N1|Original||2.3.4.4b|03.03.2023||||17.09.2024|Emory_University__CEIRR_||HA|4|A/chicken/Cochabamba/39539/2023_HA|EPI3558264|DNA_IN">
<date value="2023.0849315068492" direction="forwards" units="years"/>
</taxon>
<taxon id="A/chicken/Cochabamba/39555/2023|EPI_ISL_19410297|A_/_H5N1|Original||2.3.4.4b|09.03.2023||||12.09.2024|Emory_University__CEIRR_||HA|4|A/chicken/Cochabamba/39555/2023_HA|EPI3555406|DNA_IN">
<date value="2023.1013698630136" direction="forwards" units="years"/>
</taxon>
<taxon id="A/chicken/Cochabamba/39581/2023|EPI_ISL_19410298|A_/_H5N1|Original||2.3.4.4b|13.05.2023||||12.09.2024|Emory_University__CEIRR_||HA|4|A/chicken/Cochabamba/39581/2023_HA|EPI3555412|DNA_IN">
<date value="2023.3643835616438" direction="forwards" units="years"/>
</taxon>
<taxon id="A/chicken/Maule/247588-
1/2023|EPI_ISL_19410280|A_/_H5N1|Original||2.3.4.4b|07.04.2023||||12.09.2024|Emory_University__CEIRR_|Emory_University__CEIRR_|HA|4|A/chicken/Maule/247588-1/2023_HA|EPI3555295|DNA_IN">
<date value="2023.2657534246575" direction="forwards" units="years"/>
</taxon>
<taxon id="A/chicken/Metropolitana/243148-
1/2023|EPI_ISL_18760060|A_/_H5N1|Original||2.3.4.4b|20.03.2023||||14.01.2024|Emory_University__CEIRR_|Emory_University__CEIRR_|HA|4|A/chicken/Metropolitana/243148-1/2023_HA|EPI2913183|DNA_IN">
<date value="2023.131506849315" direction="forwards" units="years"/>
</taxon>
<taxon id="A/chicken/Michigan/23-008143-001-
original/2023|EPI_ISL_19490436|A_/_H5N1|original||2.3.4.4b|10.03.2023|Killian_Mary_Lea||23-008143-
001|18.10.2024|National_Veterinary_Services_Laboratories_-
_USDA|National_Veterinary_Services_Laboratories_-_USDA|HA|4|HA">
<date value="2023.1041095890412" direction="forwards" units="years"/>
</taxon>
<taxon id="A/chicken/Michigan/23-008143-002-
original/2023|EPI_ISL_19490438|A_/_H5N1|original||2.3.4.4b|10.03.2023|Killian_Mary_Lea||23-008143-
002|18.10.2024|National_Veterinary_Services_Laboratories_-
_USDA|National_Veterinary_Services_Laboratories_-_USDA|HA|4|HA">
<date value="2023.1041095890412" direction="forwards" units="years"/>
</taxon>
<taxon id="A/chicken/Mississippi/23-008358-001-
original/2023|EPI_ISL_19490460|A_/_H5N1|original||2.3.4.4b|15.03.2023|Killian_Mary_Lea||23-008358-
001|18.10.2024|National_Veterinary_Services_Laboratories_-
_USDA|National_Veterinary_Services_Laboratories_-_USDA|HA|4">
<date value="2023.1178082191782" direction="forwards" units="years"/>
</taxon>
<taxon id="A/chicken/Mississippi/23-008358-002-
original/2023|EPI_ISL_19490462|A_/_H5N1|original||2.3.4.4b|15.03.2023|Killian_Mary_Lea||23-008358-
002|18.10.2024|National_Veterinary_Services_Laboratories_-
_USDA|National_Veterinary_Services_Laboratories_-_USDA|HA|4">
<date value="2023.1178082191782" direction="forwards" units="years"/>
</taxon>
<taxon id="A/chicken/Nuble/239136/2023|EPI_ISL_17885878|A_/_H5N1|Original||2.3.4.4b|27.02.2023|GISAID_EpiFlu_Data_Curator|||19.07.2023|CEIRS_Data_Processing_and_Coordinating_Center_Center_for_Research_on_Influenza_Pathogenesis__CRIP_|CEIRS_Data_Processing_and_C">
<date value="2023.158904109589" direction="forwards" units="years"/>
</taxon>

<taxon id="A/chicken/Nuble/240155/2023|EPI_ISL_17885877|A_/_H5N1|Original||
2.3.4.4b|06.03.2023|GISAID_EpiFlu_Data_Curator|||29.06.2023|CEIRS_Data_Proc
essing_and_Coordinating_Center_Center_for_Research_on_Influenza_Pathogenesi
s__CRIP_|CEIRS_Data_Processing_and_C">
<date value="2023.0931506849315" direction="forwards" units="years"/>
</taxon>
<taxon id="A/chicken/Nuble/240684/2023|EPI_ISL_17885876|A_/_H5N1|Original||
2.3.4.4b|08.03.2023|GISAID_EpiFlu_Data_Curator|||19.07.2023|CEIRS_Data_Proc
essing_and_Coordinating_Center_Center_for_Research_on_Influenza_Pathogenesi
s__CRIP_|CEIRS_Data_Processing_and_C">
<date value="2023.0986301369862" direction="forwards" units="years"/>
</taxon>
<taxon id="A/chicken/Nuble/241557-
1/2023|EPI_ISL_17885874|A_/_H5N1|Original||2.3.4.4b|10.03.2023|GISAID_EpiFl
u_Data_Curator|||19.07.2023|CEIRS_Data_Processing_and_Coordinating_Center_C
enter_for_Research_on_Influenza_Pathogenesis__CRIP_|CEIRS_Data_Processing_a
nd">
<date value="2023.1041095890412" direction="forwards" units="years"/>
</taxon>
<taxon id="A/chicken/Nuble/241681-
1/2023|EPI_ISL_17885873|A_/_H5N1|Original||2.3.4.4b|10.03.2023|GISAID_EpiFl
u_Data_Curator|||19.07.2023|CEIRS_Data_Processing_and_Coordinating_Center_C
enter_for_Research_on_Influenza_Pathogenesis__CRIP_|CEIRS_Data_Processing_a
nd">
<date value="2023.1041095890412" direction="forwards" units="years"/>
</taxon>
<taxon id="A/chicken/OHiggins/241252-
1/2023|EPI_ISL_17885872|A_/_H5N1|Original||2.3.4.4b|12.03.2023|GISAID_EpiFl
u_Data_Curator|||19.07.2023|CEIRS_Data_Processing_and_Coordinating_Center_C
enter_for_Research_on_Influenza_Pathogenesis__CRIP_|CEIRS_Data_Processing_"
>
<date value="2023.109589041096" direction="forwards" units="years"/>
</taxon>
<taxon id="A/chicken/OHiggins/241252-
3/2023|EPI_ISL_17885871|A_/_H5N1|Original||2.3.4.4b|12.03.2023|GISAID_EpiFl
u_Data_Curator|||19.07.2023|CEIRS_Data_Processing_and_Coordinating_Center_C
enter_for_Research_on_Influenza_Pathogenesis__CRIP_|CEIRS_Data_Processing_"
>
<date value="2023.109589041096" direction="forwards" units="years"/>
</taxon>
<taxon id="A/chicken/OHiggins/241252-
6/2023|EPI_ISL_17885870|A_/_H5N1|Original||2.3.4.4b|12.03.2023|GISAID_EpiFl
u_Data_Curator|||19.07.2023|CEIRS_Data_Processing_and_Coordinating_Center_C
enter_for_Research_on_Influenza_Pathogenesis__CRIP_|CEIRS_Data_Processing_"
>
<date value="2023.109589041096" direction="forwards" units="years"/>
</taxon>
<taxon id="A/chicken/OHiggins/242581-
2/2023|EPI_ISL_18690682|A_/_H5N1|Original||2.3.4.4b|16.03.2023||||26.12.202
3|Emory_University__CEIRR_|Emory_University__CEIRR_|HA|4|A/chicken/OHiggins
/242581-2/2023_HA|EPI2868174|DNA_IN">
<date value="2023.1205479452055" direction="forwards" units="years"/>
</taxon>
<taxon id="A/chicken/Oregon/23-009029-001-
original/2023|EPI_ISL_19490488|A_/_H5N1|original||2.3.4.4b|15.03.2023|Killi
an_Mary_Lea||23-009029-
001|18.10.2024|National_Veterinary_Services_Laboratories_-
_USDA|National_Veterinary_Services_Laboratories_-_USDA|HA|4|HA_A">
<date value="2023.1178082191782" direction="forwards" units="years"/>
</taxon>

<taxon id="A/chicken/Panama/23-004630-
004/2023|EPI_ISL_17660068|A_/_H5N1|Original||2.3.4.4b|03.02.2023|Killian_Ma
ry_Lea|LEA-39-
23_M4|324|15.05.2023|El_Laboratorio_de_Diagnostico_de_Enfermedades_Vesicula
res__LADIVES_|National_Veterinary_Services_Laboratories_-_US">
<date value="2023.0931506849315" direction="forwards" units="years"/>
</taxon>
<taxon id="A/chicken/Panama/23-004630-
005/2023|EPI_ISL_17660069|A_/_H5N1|Original||2.3.4.4b|03.02.2023|Killian_Ma
ry_Lea|LEA-40-
23_M5|324|15.05.2023|El_Laboratorio_de_Diagnostico_de_Enfermedades_Vesicula
res__LADIVES_|National_Veterinary_Services_Laboratories_-_US">
<date value="2023.0931506849315" direction="forwards" units="years"/>
</taxon>
<taxon id="A/chicken/Peru/AIS0542/2022|EPI_ISL_17805989|A_/_H5N1|Original||
2.3.4.4b|01.12.2022|GISAID_EpiFlu_Data_Curator|||15.06.2023|||HA|4|A/chicke
n/Peru/AIS0542/2022_HA|EPI2597168|DNA_IN">
<date value="2022.9178082191781" direction="forwards" units="years"/>
</taxon>
<taxon id="A/chicken/Peru/AIS0543/2022|EPI_ISL_17805990|A_/_H5N1|Original||
2.3.4.4b|01.12.2022|GISAID_EpiFlu_Data_Curator|||15.06.2023|||HA|4|A/chicke
n/Peru/AIS0543/2022_HA|EPI2597176|DNA_IN">
<date value="2022.9178082191781" direction="forwards" units="years"/>
</taxon>
<taxon id="A/chicken/Peru/AIS0544/2022|EPI_ISL_17805991|A_/_H5N1|Original||
2.3.4.4b|03.12.2022|GISAID_EpiFlu_Data_Curator|||15.06.2023|||HA|4|A/chicke
n/Peru/AIS0544/2022_HA|EPI2597184|DNA_IN">
<date value="2022.9232876712329" direction="forwards" units="years"/>
</taxon>
<taxon id="A/chicken/Peru/AIS0545/2022|EPI_ISL_17805992|A_/_H5N1|Original||
2.3.4.4b|03.12.2022|GISAID_EpiFlu_Data_Curator|||15.06.2023|||HA|4|A/chicke
n/Peru/AIS0545/2022_HA|EPI2597191|DNA_IN">
<date value="2022.9232876712329" direction="forwards" units="years"/>
</taxon>
<taxon id="A/chicken/Peru/AIS0546/2022|EPI_ISL_17805986|A_/_H5N1|Original||
2.3.4.4b|18.12.2022|GISAID_EpiFlu_Data_Curator|||15.06.2023|||HA|4|A/chicke
n/Peru/AIS0546/2022_HA|EPI2597144|DNA_IN">
<date value="2022.964383561644" direction="forwards" units="years"/>
</taxon>
<taxon id="A/chicken/Peru/AIS0547/2022|EPI_ISL_17805993|A_/_H5N1|Original||
2.3.4.4b|28.12.2022|GISAID_EpiFlu_Data_Curator|||15.06.2023|||HA|4|A/chicke
n/Peru/AIS0547/2022_HA|EPI2597196|DNA_IN">
<date value="2022.9917808219177" direction="forwards" units="years"/>
</taxon>
<taxon id="A/chicken/Peru/AIS0548/2022|EPI_ISL_17805994|A_/_H5N1|Original||
2.3.4.4b|22.12.2022|GISAID_EpiFlu_Data_Curator|||15.06.2023|||HA|4|A/chicke
n/Peru/AIS0548/2022_HA|EPI2597204|DNA_IN">
<date value="2022.9753424657533" direction="forwards" units="years"/>
</taxon>
<taxon id="A/chicken/Peru/AIS0549/2022|EPI_ISL_17805996|A_/_H5N1|Original||
2.3.4.4b|22.12.2022|GISAID_EpiFlu_Data_Curator|||15.06.2023|||HA|4|A/chicke
n/Peru/AIS0549/2022_HA|EPI2597220|DNA_IN">
<date value="2022.9753424657533" direction="forwards" units="years"/>
</taxon>
<taxon id="A/chicken/Peru/AIS0550/2022|EPI_ISL_17805995|A_/_H5N1|Original||
2.3.4.4b|12.12.2022|GISAID_EpiFlu_Data_Curator|||15.06.2023|||HA|4|A/chicke
n/Peru/AIS0550/2022_HA|EPI2597212|DNA_IN">
<date value="2022.9479452054795" direction="forwards" units="years"/>
</taxon>

<taxon id="A/chicken/Peru/AIS0551/2022|EPI_ISL_17806001|A_/_H5N1|Original||2.3.4.4b|12.12.2022|GISAID_EpiFlu_Data_Curator|||15.06.2023|||HA|4|A/chicken/Peru/AIS0551/2022_HA|EPI2597260|DNA_IN">
<date value="2022.9479452054795" direction="forwards" units="years"/>
</taxon>
<taxon id="A/chicken/Potosi/39870/2023|EPI_ISL_19410299|A_/_H5N1|Original||2.3.4.4b|06.06.2023||||12.09.2024|Emory_University__CEIRR_||HA|4|A/chicken/Potosi/39870/2023_HA|EPI3555418|DNA_IN">
<date value="2023.4301369863015" direction="forwards" units="years"/>
</taxon>
<taxon id="A/chungungo/Chile/241436/2023|EPI_ISL_19391459|A_/_H5N1|Original||2.3.4.4b|10.03.2023||||09.09.2024|CEIRS_Data_Processing_and_Coordinating_Center_Center_for_Research_on_Influenza_Pathogenesis__CRIP_||HA|4|A/chungungo/Chile/241436/2023_HA|EPI3549620|DN">
<date value="2023.1041095890412" direction="forwards" units="years"/>
</taxon>
<taxon id="A/common_raven/Alaska/22-042128-001/2022|EPI_ISL_19158196|A_/_H5N1|Original||2.3.4.4b|18.12.2022||||22.05.2024|USDA_APHIS_Veterinary_Services_Diagnostic_Virology_Laboratory_National_Veterinary_Services_Laboratories||HA|4|A/common_raven/Alasca/22-04">
<date value="2022.964383561644" direction="forwards" units="years"/>
</taxon>
<taxon id="A/coopers_hawk/Colorado/22-042170-001/2022|EPI_ISL_19608100|A_/_H5N1|Original||2.3.4.4b|25.12.2022||||12.12.2024|USDA_APHIS_Veterinary_Services_Diagnostic_Virology_Laboratory_National_Veterinary_Services_Laboratories||HA|4|A/coopers_hawk/Colorado/2">
<date value="2022.9835616438356" direction="forwards" units="years"/>
</taxon>
<taxon id="A/coopers_hawk/Washington/22-041088-001/2022|EPI_ISL_19608106|A_/_H5N1|Original||2.3.4.4b|14.12.2022||||12.12.2024|USDA_APHIS_Veterinary_Services_Diagnostic_Virology_Laboratory_National_Veterinary_Services_Laboratories||HA|4|A/coopers_hawk/Washingt">
<date value="2022.9534246575342" direction="forwards" units="years"/>
</taxon>
<taxon id="A/crow/Colorado/22-040765-001/2022|EPI_ISL_19608110|A_/_H5N1|Original||2.3.4.4b|12.12.2022||||12.12.2024|USDA_APHIS_Veterinary_Services_Diagnostic_Virology_Laboratory_National_Veterinary_Services_Laboratories||HA|4|A/crow/Colorado/22-040765-001/202">
<date value="2022.9479452054795" direction="forwards" units="years"/>
</taxon>
<taxon id="A/dolphin/Maule/SJCEIRR-246026/2023|EPI_ISL_18777139|A_/_H5N1|Original||2.3.4.4b|31.03.2023||||16.01.2024|Emory_University__CEIRR_|Emory_University__CEIRR_|HA|4|A/dolphin/Maule/SJCEIRR-246026/2023|EPI2921521|DNA_IN">
<date value="2023.1616438356164" direction="forwards" units="years"/>
</taxon>
<taxon id="A/dolphin/Nuble/SJCEIRR-2482441/2023|EPI_ISL_18777138|A_/_H5N1|Original||2.3.4.4b|01.04.2023||||16.01.2024|Emory_University__CEIRR_|Emory_University__CEIRR_|HA|4|A/dolphin/Nuble/SJCEIRR-2482441/2023|EPI2921516|DNA_IN">
<date value="2023.2493150684932" direction="forwards" units="years"/>
</taxon>
<taxon id="A/duck/Araucania/239189-3/2023|EPI_ISL_17885963|A_/_H5N1|Original||2.3.4.4b|28.02.2023|GISAID_EpiFlu_Data_Curator|||29.06.2023|||HA|4|A/duck/Araucania/239189-3/2023_HA|EPI2609467|DNA_IN">
<date value="2023.1616438356164" direction="forwards" units="years"/>
</taxon>
<taxon id="A/duck/Araucania/240481-2/2023|EPI_ISL_17885961|A_/_H5N1|Original||2.3.4.4b|07.03.2023|GISAID_EpiFl

u_Data_Curator|||29.06.2023|||HA|4|A/duck/Araucania/240481-
2/2023_HA|EPI2609458|DNA_IN">
<date value="2023.0958904109589" direction="forwards" units="years"/>
</taxon>
<taxon id="A/duck/Araucania/241914-
2/2023|EPI_ISL_17885959|A_/_H5N1|Original||2.3.4.4b|14.03.2023|GISAID_EpiFl
u_Data_Curator|||29.06.2023|||HA|4|A/duck/Araucania/241914-
2/2023_HA|EPI2609449|DNA_IN">
<date value="2023.1150684931506" direction="forwards" units="years"/>
</taxon>
<taxon id="A/duck/Maule/240466-
1/2023|EPI_ISL_17885957|A_/_H5N1|Original||2.3.4.4b|07.03.2023|GISAID_EpiFl
u_Data_Curator|||29.06.2023|||HA|4|A/duck/Maule/240466-
1/2023_HA|EPI2609439|DNA_IN">
<date value="2023.0958904109589" direction="forwards" units="years"/>
</taxon>
<taxon id="A/duck/Peru/CAL-INS-
013/2023|EPI_ISL_18217104|A_/_H5N1|Original||2.3.4.4b|24.07.2023|Padilla_Ro
jas_Carlos_Patricio|||04.09.2023|Servicio_Nacional_Forestal_y_de_Fauna_Silv
estre__SERFOR_|Instituto_Nacional_de_Salud_Peru|HA|4|A/duck/Peru/CAL-INS-
013/2023">
<date value="2023.5616438356165" direction="forwards" units="years"/>
</taxon>
<taxon id="A/duck/Peru/LAM-INS-
014/2023|EPI_ISL_18497946|A_/_H5N1|Original||2.3.4.4b|26.09.2023|Padilla_Ro
jas_Carlos_Patricio|||13.11.2023|Servicio_Nacional_de_Sanidad_Agraria_del_P
eru_-_SENASA|Instituto_Nacional_de_Salud_Peru|HA|4|A/duck/Peru/LAM-INS-
014/2023|E">
<date value="2023.7369863013698" direction="forwards" units="years"/>
</taxon>
<taxon id="A/duck/Peru/LAM-INS-
015/2023|EPI_ISL_18497950|A_/_H5N1|Original||2.3.4.4b|20.09.2023|Padilla_Ro
jas_Carlos_Patricio|||13.11.2023|Servicio_Nacional_de_Sanidad_Agraria_del_P
eru_-_SENASA|Instituto_Nacional_de_Salud_Peru|HA|4|A/duck/Peru/LAM-INS-
015/2023|E">
<date value="2023.7205479452055" direction="forwards" units="years"/>
</taxon>
<taxon id="A/dunlin/BC/AIVPHL-
335/2022|EPI_ISL_17051470|A_/_H5N1|Original||2.3.4.4b|13.12.2022|Russell_Sh
annon_Laurel|||28.02.2023|B.C._Centre_for_Disease_Control|British_Columbia_
Centre_for_Disease_Control|HA|4|A/dunlin/BC/AIVPHL-
335/2022_HA|EPI2429201|DNA_INS">
<date value="2022.9506849315069" direction="forwards" units="years"/>
</taxon>
<taxon id="A/dunlin/Washington/22-040394-
001/2022|EPI_ISL_19607890|A_/_H5N1|Original||2.3.4.4b|03.12.2022||||12.12.2
024|USDA_APHIS_Veterinary_Services_Diagnostic_Virology_Laboratory_National_
Veterinary_Services_Laboratories||HA|4|A/dunlin/Washington/22-040394">
<date value="2022.9232876712329" direction="forwards" units="years"/>
</taxon>
<taxon id="A/eared_grebe/California/23-000276-
003/2022|EPI_ISL_19607866|A_/_H5N1|Original||2.3.4.4b|15.12.2022||||12.12.2
024|USDA_APHIS_Veterinary_Services_Diagnostic_Virology_Laboratory_National_
Veterinary_Services_Laboratories||HA|4|A/eared_grebe/California">
<date value="2022.9561643835616" direction="forwards" units="years"/>
</taxon>
<taxon id="A/eared_grebe/California/23-000276-
004/2022|EPI_ISL_19607867|A_/_H5N1|Original||2.3.4.4b|15.12.2022||||12.12.2
024|USDA_APHIS_Veterinary_Services_Diagnostic_Virology_Laboratory_National_
Veterinary_Services_Laboratories||HA|4|A/eared_grebe/California">
<date value="2022.9561643835616" direction="forwards" units="years"/>

</taxon>
<taxon id="A/environment/Chile/C62044/2022|EPI_ISL_19167924|A_/_H5N1|Original||2.3.4.4b|13.12.2022||||28.05.2024|St._Jude_Center_of_Excellence_for_Influenza_Research_and_Response_-
_CEIRR_Influenza_Data_Processing_and_Communication_Center||HA|4|A/environment/Chile">
<date value="2022.9506849315069" direction="forwards" units="years"/>
</taxon>
<taxon id="A/falcon/Costa_Rica/INC-0001-D0377-
23/2023|EPI_ISL_16958548|A_/_H5N1|Original||2.3.4.4b|26.01.2023|Soto-
Garita_Claudio|377-
23|783955|07.07.2023|Servicio_Nacional_de_Salud_Animal__SENASA_|Inciensa_Investigacion_y_Ensenanza_en_Nutricion_y_Salud|HA|4|A/">
<date value="2023.0712328767124" direction="forwards" units="years"/>
</taxon>
<taxon id="A/frigatebird/Rio_de_Janeiro/MAPA-
1532N/2023|EPI_ISL_18755251|A_/_H5N1|Original||2.3.4.4b|03.06.2023||||12.01.2024|Ministerio_da_Agricultura_e_Pecuaria|Ministerio_da_Agricultura_e_Pecuaria|HA|4|A/frigatebird/Rio_de_Janeiro/MAPA-1532N/2023_HA|EPI2908258">
<date value="2023.4219178082192" direction="forwards" units="years"/>
</taxon>
<taxon id="A/gadwall/Alaska/22-040806-
001/2022|EPI_ISL_19607884|A_/_H5N1|Original||2.3.4.4b|12.12.2022||||12.12.2024|USDA_APHIS_Veterinary_Services_Diagnostic_Virology_Laboratory_National_Veterinary_Services_Laboratories||HA|4|A/gadwall/Alaska/22-040806-001/2">
<date value="2022.9479452054795" direction="forwards" units="years"/>
</taxon>
<taxon id="A/gadwall/Alaska/22-041762-
001/2022|EPI_ISL_19607925|A_/_H5N1|Original||2.3.4.4b|10.12.2022||||12.12.2024|USDA_APHIS_Veterinary_Services_Diagnostic_Virology_Laboratory_National_Veterinary_Services_Laboratories||HA|4|A/gadwall/Alaska/22-041762-001/2">
<date value="2022.9424657534246" direction="forwards" units="years"/>
</taxon>
<taxon id="A/gadwall/Alaska/22-041762-
004/2022|EPI_ISL_19607930|A_/_H5N1|Original||2.3.4.4b|10.12.2022||||12.12.2024|USDA_APHIS_Veterinary_Services_Diagnostic_Virology_Laboratory_National_Veterinary_Services_Laboratories||HA|4|A/gadwall/Alaska/22-041762-004/2">
<date value="2022.9424657534246" direction="forwards" units="years"/>
</taxon>
<taxon id="A/gadwall/Alaska/22-041762-
005/2022|EPI_ISL_19607980|A_/_H5N1|Original||2.3.4.4b|10.12.2022||||12.12.2024|USDA_APHIS_Veterinary_Services_Diagnostic_Virology_Laboratory_National_Veterinary_Services_Laboratories||HA|4|A/gadwall/Alaska/22-041762-005/2">
<date value="2022.9424657534246" direction="forwards" units="years"/>
</taxon>
<taxon id="A/gadwall/Alaska/22-041762-
006/2022|EPI_ISL_19607982|A_/_H5N1|Original||2.3.4.4b|10.12.2022||||12.12.2024|USDA_APHIS_Veterinary_Services_Diagnostic_Virology_Laboratory_National_Veterinary_Services_Laboratories||HA|4|A/gadwall/Alaska/22-041762-006/2">
<date value="2022.9424657534246" direction="forwards" units="years"/>
</taxon>
<taxon id="A/gadwall/Alaska/23-002626-
001/2023|EPI_ISL_19607984|A_/_H5N1|Original||2.3.4.4b|17.01.2023||||12.12.2024|USDA_APHIS_Veterinary_Services_Diagnostic_Virology_Laboratory_National_Veterinary_Services_Laboratories||HA|4|A/gadwall/Alaska/23-002626-001/2">
<date value="2023.0465753424658" direction="forwards" units="years"/>
</taxon>
<taxon id="A/gadwall/Oregon/23-001494-
006/2023|EPI_ISL_19608014|A_/_H5N1|Original||2.3.4.4b|05.01.2023||||12.12.2024|USDA_APHIS_Veterinary_Services_Diagnostic_Virology_Laboratory_National_Veterinary_Services_Laboratories||HA|4|A/gadwall/Oregon/23-001494-006/2">

<date value="2023.013698630137" direction="forwards" units="years"/>
</taxon>
<taxon id="A/gadwall/South_Carolina/22-042019-001/2022|EPI_ISL_19608016|A_/_H5N1|Original||2.3.4.4b|15.12.2022||||12.12.2024|USDA_APHIS_Veterinary_Services_Diagnostic_Virology_Laboratory_National_Veterinary_Services_Laboratories||HA|4|A/gadwall/South_Carolina">
<date value="2022.9561643835616" direction="forwards" units="years"/>
</taxon>
<taxon id="A/gadwall/Texas/23-003062-010/2023|EPI_ISL_19607945|A_/_H5N1|Original||2.3.4.4b|18.01.2023||||12.12.2024|USDA_APHIS_Veterinary_Services_Diagnostic_Virology_Laboratory_National_Veterinary_Services_Laboratories||HA|4|A/gadwall/Texas/23-003062-010/202">
<date value="2023.0493150684931" direction="forwards" units="years"/>
</taxon>
<taxon id="A/gannet/Florida/23-002762-022/2022|EPI_ISL_19607954|A_/_H5N1|Original||2.3.4.4b|30.12.2022||||12.12.2024|USDA_APHIS_Veterinary_Services_Diagnostic_Virology_Laboratory_National_Veterinary_Services_Laboratories||HA|4|A/gannet/Florida/23-002762-022/2">
<date value="2022.9972602739726" direction="forwards" units="years"/>
</taxon>
<taxon id="A/glaucous_gull/Washington/23-025001-002/2023|EPI_ISL_18737455|A_/_H5N1|Original||2.3.4.4b|11.08.2023|Killian_Mary_Lea|23-025001-002||12.12.2024|National_Veterinary_Services_Laboratories_-_USDA|National_Veterinary_Services_Laboratories_-_USDA|HA|4|HA_">
<date value="2023.6109589041096" direction="forwards" units="years"/>
</taxon>
<taxon id="A/glaucous_gull/Washington/23-025001-003/2023|EPI_ISL_18737463|A_/_H5N1|Original||2.3.4.4b|11.08.2023|Killian_Mary_Lea|23-025001-003||12.12.2024|National_Veterinary_Services_Laboratories_-_USDA|National_Veterinary_Services_Laboratories_-_USDA|HA|4|HA_">
<date value="2023.6109589041096" direction="forwards" units="years"/>
</taxon>
<taxon id="A/goose/Araucania/239189-1/2023|EPI_ISL_17885869|A_/_H5N1|Original||2.3.4.4b|28.02.2023|GISAID_EpiFlu_Data_Curator|||24.07.2023|CEIRS_Data_Processing_and_Coordinating_Center_Center_for_Research_on_Influenza_Pathogenesis__CRIP_|CEIRS_Data_Processing_a">
<date value="2023.1616438356164" direction="forwards" units="years"/>
</taxon>
<taxon id="A/goose/Argentina/SENASA-140223/2023|EPI_ISL_17950994|A_/_H5N1|Original||2.3.4.4b|11.02.2023|GISAID_EpiFlu_Data_Curator|||30.06.2023|||HA|4|A/goose/Argentina/SENASA-140223/2023_HA|EPI2610769|DNA_IN">
<date value="2023.1150684931506" direction="forwards" units="years"/>
</taxon>
<taxon id="A/goose/California/23-001053-002/2022|EPI_ISL_19605288|A_/_H5N1|Original||2.3.4.4b|17.12.2022||||12.12.2024|USDA_APHIS_Veterinary_Services_Diagnostic_Virology_Laboratory_National_Veterinary_Services_Laboratories||HA|4|A/goose/California/23-001053-0">
<date value="2022.9616438356165" direction="forwards" units="years"/>
</taxon>
<taxon id="A/goose/Kansas/W22-1154/2022|EPI_ISL_17373141|A_/_H5N1|||2.3.4.4b|05.12.2022||||01.04.2023|St._Jude_Center_of_Excellence_for_Influenza_Research_and_Response_-_CEIRR_Influenza_Data_Processing_and_Communication_Center|St._Jude_Center_of_Excellence_for_In">
<date value="2022.9287671232876" direction="forwards" units="years"/>
</taxon>

<taxon id="A/goose/Louisiana/23-000409-
006/2022|EPI_ISL_19608587|A_/_H5N1|Original||2.3.4.4b|02.12.2022||||12.12.2
024|USDA_APHIS_Veterinary_Services_Diagnostic_Virology_Laboratory_National_
Veterinary_Services_Laboratories||HA|4|A/goose/Louisiana/23-000409-006">
<date value="2022.9205479452055" direction="forwards" units="years"/>
</taxon>
<taxon id="A/goose/Louisiana/W22-
1163/2022|EPI_ISL_17373068|A_/_H5N1|||2.3.4.4b|05.12.2022||||01.04.2023|St.
_Jude_Center_of_Excellence_for_Influenza_Research_and_Response_-
_CEIRR_Influenza_Data_Processing_and_Communication_Center|St._Jude_Center_o
f_Excellence_for">
<date value="2022.9287671232876" direction="forwards" units="years"/>
</taxon>
<taxon id="A/goose/Utah/23-001040-
001/2023|EPI_ISL_19605253|A_/_H5N1|Original||2.3.4.4b|04.01.2023||||12.12.2
024|USDA_APHIS_Veterinary_Services_Diagnostic_Virology_Laboratory_National_
Veterinary_Services_Laboratories||HA|4|A/goose/Utah/23-001040-
001/2023_HA|E">
<date value="2023.0109589041097" direction="forwards" units="years"/>
</taxon>
<taxon id="A/goose/Utah/23-001041-
001/2023|EPI_ISL_19605254|A_/_H5N1|Original||2.3.4.4b|06.01.2023||||12.12.2
024|USDA_APHIS_Veterinary_Services_Diagnostic_Virology_Laboratory_National_
Veterinary_Services_Laboratories||HA|4|A/goose/Utah/23-001041-
001/2023_HA|E">
<date value="2023.0164383561644" direction="forwards" units="years"/>
</taxon>
<taxon id="A/goose/Utah/23-001041-
003/2023|EPI_ISL_19605257|A_/_H5N1|Original||2.3.4.4b|06.01.2023||||12.12.2
024|USDA_APHIS_Veterinary_Services_Diagnostic_Virology_Laboratory_National_
Veterinary_Services_Laboratories||HA|4|A/goose/Utah/23-001041-
003/2023_HA|E">
<date value="2023.0164383561644" direction="forwards" units="years"/>
</taxon>
<taxon id="A/goose/Utah/23-001041-
007/2023|EPI_ISL_19605268|A_/_H5N1|Original||2.3.4.4b|06.01.2023||||12.12.2
024|USDA_APHIS_Veterinary_Services_Diagnostic_Virology_Laboratory_National_
Veterinary_Services_Laboratories||HA|4|A/goose/Utah/23-001041-
007/2023_HA|E">
<date value="2023.0164383561644" direction="forwards" units="years"/>
</taxon>
<taxon id="A/goose/Utah/23-001041-
009/2023|EPI_ISL_19605259|A_/_H5N1|Original||2.3.4.4b|06.01.2023||||12.12.2
024|USDA_APHIS_Veterinary_Services_Diagnostic_Virology_Laboratory_National_
Veterinary_Services_Laboratories||HA|4|A/goose/Utah/23-001041-
009/2023_HA|E">
<date value="2023.0164383561644" direction="forwards" units="years"/>
</taxon>
<taxon id="A/gray_gull/Arica/239053-
1/2023|EPI_ISL_19410281|A_/_H5N1|Original||2.3.4.4b|23.02.2023||||12.09.202
4|Emory_University__CEIRR_|Emory_University__CEIRR_|HA|4|A/gray_gull/Arica/
239053-1/2023_HA|EPI3555302|DNA_IN">
<date value="2023.1479452054793" direction="forwards" units="years"/>
</taxon>
<taxon id="A/gray_gull/Arica_y_Parinacota/235908-
1/2023|EPI_ISL_19404913|A_/_H5N1|Original||2.3.4.4b|07.02.2023||||10.09.202
4|Emory_University__CEIRR_|Emory_University__CEIRR_|HA|4|A/gray_gull/Arica_
y_Parinacota/235908-1/2023_HA|EPI3550208|DNA_IN">
<date value="2023.1041095890412" direction="forwards" units="years"/>
</taxon>

<taxon id="A/gray_gull/Chile/C61947/2022|EPI_ISL_16891400|A_/_H5N1|||2.3.4.4b|02.12.2022||||04.02.2023|||HA|4|A/gray_gull/Chile/C61947/2022|EPI2397300|DNA_IN">
<date value="2022.9205479452055" direction="forwards" units="years"/>
</taxon>
<taxon id="A/great-tailed_grackle/Kansas/22-041130-005/2022|EPI_ISL_19605261|A_/_H5N1|Original||2.3.4.4b|13.12.2022||||12.12.2024|USDA_APHIS_Veterinary_Services_Diagnostic_Virology_Laboratory_National_Veterinary_Services_Laboratories||HA|4|A/great-tailed_grac">
<date value="2022.9506849315069" direction="forwards" units="years"/>
</taxon>
<taxon id="A/great-tailed_grackle/Kansas/22-041130-006/2022|EPI_ISL_19605262|A_/_H5N1|Original||2.3.4.4b|13.12.2022||||12.12.2024|USDA_APHIS_Veterinary_Services_Diagnostic_Virology_Laboratory_National_Veterinary_Services_Laboratories||HA|4|A/great-tailed_grac">
<date value="2022.9506849315069" direction="forwards" units="years"/>
</taxon>
<taxon id="A/great-tailed_grackle/Kansas/W22-1223B/2022|EPI_ISL_17471582|A_/_H5N1|||2.3.4.4b|13.12.2022||||09.04.2023|St._Jude_Center_of_Excellence_for_Influenza_Research_and_Response_-_CEIRR_Influenza_Data_Processing_and_Communication_Center|St._Jude_Center_of_E">
<date value="2022.9506849315069" direction="forwards" units="years"/>
</taxon>
<taxon id="A/great-tailed_grackle/Kansas/W22-1223C/2022|EPI_ISL_17471577|A_/_H5N1|||2.3.4.4b|13.12.2022||||09.04.2023|St._Jude_Center_of_Excellence_for_Influenza_Research_and_Response_-_CEIRR_Influenza_Data_Processing_and_Communication_Center|St._Jude_Center_of_E">
<date value="2022.9506849315069" direction="forwards" units="years"/>
</taxon>
<taxon id="A/great_horned_owl/BC/AIVPHL-353/2023|EPI_ISL_17051479|A_/_H5N1|Original||2.3.4.4b|09.01.2023|Russell_Shannon_Laurel|||28.02.2023|B.C._Centre_for_Disease_Control|British_Columbia_Centre_for_Disease_Control|HA|4|A/great_horned_owl/BC/AIVPHL-353/2023_HA">
<date value="2023.0246575342467" direction="forwards" units="years"/>
</taxon>
<taxon id="A/great_horned_owl/BC/AIVPHL-372/2023|EPI_ISL_17051490|A_/_H5N1|Original||2.3.4.4b|16.01.2023|Russell_Shannon_Laurel|||28.02.2023|B.C._Centre_for_Disease_Control|British_Columbia_Centre_for_Disease_Control|HA|4|A/great_horned_owl/BC/AIVPHL-372/2023_HA">
<date value="2023.0438356164384" direction="forwards" units="years"/>
</taxon>
<taxon id="A/great_horned_owl/California/23-000277-003/2022|EPI_ISL_19607321|A_/_H5N1|Original||2.3.4.4b|14.12.2022||||12.12.2024|USDA_APHIS_Veterinary_Services_Diagnostic_Virology_Laboratory_National_Veterinary_Services_Laboratories||HA|4|A/great_horned_owl/">
<date value="2022.9534246575342" direction="forwards" units="years"/>
</taxon>
<taxon id="A/great_horned_owl/California/23-001056-001/2022|EPI_ISL_19607323|A_/_H5N1|Original||2.3.4.4b|21.12.2022||||12.12.2024|USDA_APHIS_Veterinary_Services_Diagnostic_Virology_Laboratory_National_Veterinary_Services_Laboratories||HA|4|A/great_horned_owl/">
<date value="2022.972602739726" direction="forwards" units="years"/>
</taxon>
<taxon id="A/great_horned_owl/California/23-001856-003/2022|EPI_ISL_19607324|A_/_H5N1|Original||2.3.4.4b|19.12.2022||||12.12.2024|USDA_APHIS_Veterinary_Services_Diagnostic_Virology_Laboratory_National_Veterinary_Services_Laboratories||HA|4|A/great_horned_owl/">
<date value="2022.9671232876713" direction="forwards" units="years"/>
</taxon>

<taxon id="A/great_horned_owl/California/23-006649-001/2023|EPI_ISL_17964879|A_/_H5N1|Original||2.3.4.4b|23.02.2023|Killian_Mary_Lea||23-006649-001|06.07.2023|National_Veterinary_Services_Laboratories_-_USDA|National_Veterinary_Services_Laboratories_-_USDA|HA|4|">
<date value="2023.1479452054793" direction="forwards" units="years"/>
</taxon>
<taxon id="A/great_horned_owl/Colorado/22-039952-001/2022|EPI_ISL_19607330|A_/_H5N1|Original||2.3.4.4b|05.12.2022||||12.12.2024|USDA_APHIS_Veterinary_Services_Diagnostic_Virology_Laboratory_National_Veterinary_Services_Laboratories||HA|4|A/great_horned_owl/Co">
<date value="2022.9287671232876" direction="forwards" units="years"/>
</taxon>
<taxon id="A/great_horned_owl/Colorado/22-040764-001/2022|EPI_ISL_19607333|A_/_H5N1|Original||2.3.4.4b|09.12.2022||||12.12.2024|USDA_APHIS_Veterinary_Services_Diagnostic_Virology_Laboratory_National_Veterinary_Services_Laboratories||HA|4|A/great_horned_owl/Co">
<date value="2022.9397260273972" direction="forwards" units="years"/>
</taxon>
<taxon id="A/great_horned_owl/Colorado/23-001467-002/2023|EPI_ISL_19607365|A_/_H5N1|Original||2.3.4.4b|07.01.2023||||12.12.2024|USDA_APHIS_Veterinary_Services_Diagnostic_Virology_Laboratory_National_Veterinary_Services_Laboratories||HA|4|A/great_horned_owl/Co">
<date value="2023.0191780821917" direction="forwards" units="years"/>
</taxon>
<taxon id="A/great_horned_owl/Colorado/23-001470-001/2023|EPI_ISL_19607367|A_/_H5N1|Original||2.3.4.4b|05.01.2023||||12.12.2024|USDA_APHIS_Veterinary_Services_Diagnostic_Virology_Laboratory_National_Veterinary_Services_Laboratories||HA|4|A/great_horned_owl/Co">
<date value="2023.013698630137" direction="forwards" units="years"/>
</taxon>
<taxon id="A/great_horned_owl/Colorado/23-002236-003/2023|EPI_ISL_19607368|A_/_H5N1|Original||2.3.4.4b|11.01.2023||||12.12.2024|USDA_APHIS_Veterinary_Services_Diagnostic_Virology_Laboratory_National_Veterinary_Services_Laboratories||HA|4|A/great_horned_owl/Co">
<date value="2023.0301369863014" direction="forwards" units="years"/>
</taxon>
<taxon id="A/great_horned_owl/Colorado/23-002760-001/2023|EPI_ISL_19607395|A_/_H5N1|Original||2.3.4.4b|16.01.2023||||12.12.2024|USDA_APHIS_Veterinary_Services_Diagnostic_Virology_Laboratory_National_Veterinary_Services_Laboratories||HA|4|A/great_horned_owl/Co">
<date value="2023.0438356164384" direction="forwards" units="years"/>
</taxon>
<taxon id="A/great_horned_owl/Colorado/23-002760-002/2023|EPI_ISL_19607396|A_/_H5N1|Original||2.3.4.4b|16.01.2023||||12.12.2024|USDA_APHIS_Veterinary_Services_Diagnostic_Virology_Laboratory_National_Veterinary_Services_Laboratories||HA|4|A/great_horned_owl/Co">
<date value="2023.0438356164384" direction="forwards" units="years"/>
</taxon>
<taxon id="A/great_horned_owl/Colorado/23-005619-001/2023|EPI_ISL_17964860|A_/_H5N1|Original||2.3.4.4b|14.02.2023|Killian_Mary_Lea||23-005619-001|06.07.2023|National_Veterinary_Services_Laboratories_-_USDA|National_Veterinary_Services_Laboratories_-_USDA|HA|4|HA">
<date value="2023.123287671233" direction="forwards" units="years"/>
</taxon>
<taxon id="A/great_horned_owl/Colorado/23-005620-001/2023|EPI_ISL_17964861|A_/_H5N1|Original||2.3.4.4b|14.02.2023|Killian_Mary_Lea||23-005620-001|06.07.2023|National_Veterinary_Services_Laboratories_-_USDA|National_Veterinary_Services_Laboratories_-_USDA|HA|4|HA">

<date value="2023.123287671233" direction="forwards" units="years"/>
</taxon>
<taxon id="A/great_horned_owl/Colorado/23-005885-001/2023|EPI_ISL_17964868|A_/_H5N1|Original||2.3.4.4b|17.02.2023|Killian_Mary_Lea||23-005885-001|06.07.2023|National_Veterinary_Services_Laboratories_-_USDA|National_Veterinary_Services_Laboratories_-_USDA|HA|4|HA">
<date value="2023.131506849315" direction="forwards" units="years"/>
</taxon>
<taxon id="A/great_horned_owl/Colorado/23-007822-001/2023|EPI_ISL_17964907|A_/_H5N1|Original||2.3.4.4b|03.03.2023|Killian_Mary_Lea||23-007822-001|06.07.2023|National_Veterinary_Services_Laboratories_-_USDA|National_Veterinary_Services_Laboratories_-_USDA|HA|4|HA">
<date value="2023.0849315068492" direction="forwards" units="years"/>
</taxon>
<taxon id="A/great_horned_owl/Colorado/23-007956-001/2023|EPI_ISL_17964897|A_/_H5N1|Original||2.3.4.4b|07.03.2023|Killian_Mary_Lea||23-007956-001|06.07.2023|National_Veterinary_Services_Laboratories_-_USDA|National_Veterinary_Services_Laboratories_-_USDA|HA|4|HA">
<date value="2023.0958904109589" direction="forwards" units="years"/>
</taxon>
<taxon id="A/great_horned_owl/Idaho/23-011169-003/2023|EPI_ISL_17964927|A_/_H5N1|Original||2.3.4.4b|28.03.2023|Killian_Mary_Lea||23-011169-003|06.07.2023|National_Veterinary_Services_Laboratories_-_USDA|National_Veterinary_Services_Laboratories_-_USDA|HA|4|HA_A/">
<date value="2023.1534246575343" direction="forwards" units="years"/>
</taxon>
<taxon id="A/great_horned_owl/Idaho/23-017158-001/2023|EPI_ISL_17964981|A_/_H5N1|Original||2.3.4.4b|20.05.2023|Killian_Mary_Lea||23-017158-001|06.07.2023|National_Veterinary_Services_Laboratories_-_USDA|National_Veterinary_Services_Laboratories_-_USDA|HA|4|HA_A/">
<date value="2023.3835616438357" direction="forwards" units="years"/>
</taxon>
<taxon id="A/great_horned_owl/Minnesota/22-041884-001/2022|EPI_ISL_19607258|A_/_H5N1|Original||2.3.4.4b|07.12.2022||||12.12.2024|USDA_APHIS_Veterinary_Services_Diagnostic_Virology_Laboratory_National_Veterinary_Services_Laboratories||HA|4|A/great_horned_owl/M">
<date value="2022.9342465753425" direction="forwards" units="years"/>
</taxon>
<taxon id="A/great_horned_owl/Montana/23-011850-001/2023|EPI_ISL_17964939|A_/_H5N1|Original||2.3.4.4b|12.04.2023|Killian_Mary_Lea||23-011850-001|06.07.2023|National_Veterinary_Services_Laboratories_-_USDA|National_Veterinary_Services_Laboratories_-_USDA|HA|4|HA_">
<date value="2023.2794520547945" direction="forwards" units="years"/>
</taxon>
<taxon id="A/great_horned_owl/Nevada/22-040618-001/2022|EPI_ISL_19607219|A_/_H5N1|Original||2.3.4.4b|06.12.2022||||12.12.2024|USDA_APHIS_Veterinary_Services_Diagnostic_Virology_Laboratory_National_Veterinary_Services_Laboratories||HA|4|A/great_horned_owl/Neva">
<date value="2022.9315068493152" direction="forwards" units="years"/>
</taxon>
<taxon id="A/great_horned_owl/Oregon/22-041042-001/2022|EPI_ISL_19607226|A_/_H5N1|Original||2.3.4.4b|12.12.2022||||12.12.2024|USDA_APHIS_Veterinary_Services_Diagnostic_Virology_Laboratory_National_Veterinary_Services_Laboratories||HA|4|A/great_horned_owl/Oreg">
<date value="2022.9479452054795" direction="forwards" units="years"/>

</taxon>
<taxon id="A/great_horned_owl/Wyoming/22-039597-001/2022|EPI_ISL_19607274|A_/_H5N1|Original||2.3.4.4b|06.12.2022||||12.12.2024|USDA_APHIS_Veterinary_Services_Diagnostic_Virology_Laboratory_National_Veterinary_Services_Laboratories||HA|4|A/great_horned_owl/Wyo">
<date value="2022.9315068493152" direction="forwards" units="years"/>
</taxon>
<taxon id="A/great_horned_owl/Wyoming/22-041771-002/2022|EPI_ISL_19607287|A_/_H5N1|Original||2.3.4.4b|20.12.2022||||12.12.2024|USDA_APHIS_Veterinary_Services_Diagnostic_Virology_Laboratory_National_Veterinary_Services_Laboratories||HA|4|A/great_horned_owl/Wyo">
<date value="2022.9698630136986" direction="forwards" units="years"/>
</taxon>
<taxon id="A/greater_scaup/Montana/23-001334-001/2023|EPI_ISL_19605263|A_/_H5N1|Original||2.3.4.4b|06.01.2023||||12.12.2024|USDA_APHIS_Veterinary_Services_Diagnostic_Virology_Laboratory_National_Veterinary_Services_Laboratories||HA|4|A/greater_scaup/Montana/2">
<date value="2023.0164383561644" direction="forwards" units="years"/>
</taxon>
<taxon id="A/guanay_cormorant/Atacama/235520-1/2023|EPI_ISL_19151400|A_/_H5N1|Original||2.3.4.4b|05.02.2023||||21.05.2024|Emory_University__CEIRR_|Emory_University__CEIRR_|HA|4|A/guanay_cormorant/Atacama/235520-1/2023_HA|EPI3302324|DNA_IN">
<date value="2023.0986301369862" direction="forwards" units="years"/>
</taxon>
<taxon id="A/guanay_cormorant/Peru/CAL-INS-009/2023|EPI_ISL_17777530|A_/_H5N1|Original||2.3.4.4b|17.03.2023|Padilla_Rojas_Carlos_Patricio||1375|01.07.2023|Laboratorio_de_Virus_Respiratorios_Centro_Nacional_de_Salud_Publica|Instituto_Nacional_de_Salud_Peru|HA|4|">
<date value="2023.123287671233" direction="forwards" units="years"/>
</taxon>
<taxon id="A/gull/Biobio/237012/2023|EPI_ISL_19410300|A_/_H5N1|Original||2.3.4.4b|15.02.2023||||12.09.2024|Emory_University__CEIRR_|Emory_University__CEIRR_|HA|4|A/gull/Biobio/237012/2023_HA|EPI3555425|DNA_IN">
<date value="2023.1260273972603" direction="forwards" units="years"/>
</taxon>
<taxon id="A/gull/California/23-000936-003/2022|EPI_ISL_19607556|A_/_H5N1|Original||2.3.4.4b|16.12.2022||||12.12.2024|USDA_APHIS_Veterinary_Services_Diagnostic_Virology_Laboratory_National_Veterinary_Services_Laboratories||HA|4|A/gull/California/23-000936-003">
<date value="2022.958904109589" direction="forwards" units="years"/>
</taxon>
<taxon id="A/gull/Maule/234329-1/2023|EPI_ISL_18777130|A_/_H5N1|Original||2.3.4.4b|26.01.2023||||16.01.2024|Emory_University__CEIRR_|Emory_University__CEIRR_|HA|4|A/gull/Maule/234329-1/2023_HA|EPI2921503|DNA_IN">
<date value="2023.0712328767124" direction="forwards" units="years"/>
</taxon>
<taxon id="A/gull/Peru/LIM-INS-006/2023|EPI_ISL_17777527|A_/_H5N1|Original||2.3.4.4b|16.03.2023|Padilla_Rojas_Carlos_Patricio||1375|01.07.2023|Laboratorio_de_Virus_Respiratorios_Centro_Nacional_de_Salud_Publica|Instituto_Nacional_de_Salud_Peru|HA|4|Peru_INS006_">
<date value="2023.1205479452055" direction="forwards" units="years"/>
</taxon>
<taxon id="A/harbor_seal/Washington/23-025991-001/2023|EPI_ISL_18311025|A_/_H5N1|Original||2.3.4.4b|25.08.2023|Killian_Mary_Lea||23-025991-001|26.09.2023|National_Veterinary_Services_Laboratories_-_USDA|National_Veterinary_Services_Laboratories_-_USDA|HA|4|HA_A/">
<date value="2023.6493150684933" direction="forwards" units="years"/>

</taxon>
<taxon id="A/harbor_seal/Washington/23-026504-001/2023|EPI_ISL_18311027|A_/_H5N1|Original||2.3.4.4b|29.08.2023|Killian_Mary_Lea||23-026504-001|26.09.2023|National_Veterinary_Services_Laboratories_-_USDA|National_Veterinary_Services_Laboratories_-_USDA|HA|4|HA_A/">
<date value="2023.6602739726027" direction="forwards" units="years"/>
</taxon>
<taxon id="A/harbor_seal/Washington/23-027069-002/2023|EPI_ISL_18731638|A_/_H5N1|Original||2.3.4.4b|13.08.2023|Killian_Mary_Lea|23-027069-002||10.06.2024|National_Veterinary_Services_Laboratories_-_USDA|National_Veterinary_Services_Laboratories_-_USDA|HA|4|HA_A/">
<date value="2023.6164383561643" direction="forwards" units="years"/>
</taxon>
<taxon id="A/hawk/Iowa/23-011213-001/2023|EPI_ISL_17964928|A_/_H5N1|Original||2.3.4.4b|10.04.2023|Killian_Mary_Lea||23-011213-001|06.07.2023|National_Veterinary_Services_Laboratories_-_USDA|National_Veterinary_Services_Laboratories_-_USDA|HA|4|HA_A/hawk/Iowa/23-">
<date value="2023.2739726027398" direction="forwards" units="years"/>
</taxon>
<taxon id="A/hawk/Missouri/22-040785-002/2022|EPI_ISL_19607573|A_/_H5N1|Original||2.3.4.4b|05.12.2022||||12.12.2024|USDA_APHIS_Veterinary_Services_Diagnostic_Virology_Laboratory_National_Veterinary_Services_Laboratories||HA|4|A/hawk/Missouri/22-040785-002/202">
<date value="2022.9287671232876" direction="forwards" units="years"/>
</taxon>
<taxon id="A/heron/Antofagasta/228705-3/2022|EPI_ISL_17885969|A_/_H5N1|Original||2.3.4.4b|20.12.2022|GISAID_EpiFlu_Data_Curator|||29.06.2023|CEIRS_Data_Processing_and_Coordinating_Center_Center_for_Research_on_Influenza_Pathogenesis__CRIP_|CEIRS_Data_Processing">
<date value="2022.9698630136986" direction="forwards" units="years"/>
</taxon>
<taxon id="A/herring_gull/California/23-001053-004/2022|EPI_ISL_19607536|A_/_H5N1|Original||2.3.4.4b|17.12.2022||||12.12.2024|USDA_APHIS_Veterinary_Services_Diagnostic_Virology_Laboratory_National_Veterinary_Services_Laboratories||HA|4|A/herring_gull/Californ">
<date value="2022.9616438356165" direction="forwards" units="years"/>
</taxon>
<taxon id="A/herring_gull/California/23-001053-006/2022|EPI_ISL_19607538|A_/_H5N1|Original||2.3.4.4b|17.12.2022||||12.12.2024|USDA_APHIS_Veterinary_Services_Diagnostic_Virology_Laboratory_National_Veterinary_Services_Laboratories||HA|4|A/herring_gull/Californ">
<date value="2022.9616438356165" direction="forwards" units="years"/>
</taxon>
<taxon id="A/hooded_merganser/Washington/23-003784-001/2023|EPI_ISL_19607623|A_/_H5N1|Original||2.3.4.4b|18.01.2023||||12.12.2024|USDA_APHIS_Veterinary_Services_Diagnostic_Virology_Laboratory_National_Veterinary_Services_Laboratories||HA|4|A/hooded_merganser/">
<date value="2023.0493150684931" direction="forwards" units="years"/>
</taxon>
<taxon id="A/humboldt_penguin/Tarapaca/238744-2/2023|EPI_ISL_17885947|A_/_H5N1|Original||2.3.4.4b|23.02.2023|GISAID_EpiFlu_Data_Curator|||24.07.2023|||HA|4|A/humboldt_penguin/Tarapaca/238744-2/2023_HA|EPI2644734|DNA_IN">
<date value="2023.1479452054793" direction="forwards" units="years"/>
</taxon>
<taxon id="A/kelp_gull/Atacama/234251-1/2023|EPI_ISL_19410282|A_/_H5N1|Original||2.3.4.4b|19.01.2023||||12.09.202

4|Emory_University__CEIRR_|Emory_University__CEIRR_|HA|4|A/kelp_gull/Atacam
a/234251-1/2023_HA|EPI3555308|DNA_IN">
<date value="2023.0520547945205" direction="forwards" units="years"/>
</taxon>
<taxon id="A/kelp_gull/Valparaiso/236011-
1/2023|EPI_ISL_19404906|A_/_H5N1|Original||2.3.4.4b|09.02.2023||||10.09.202
4|Emory_University__CEIRR_|Emory_University__CEIRR_|HA|4|A/kelp_gull/Valpar
aiso/236011-1/2023_HA|EPI3550194|DNA_IN">
<date value="2023.109589041096" direction="forwards" units="years"/>
</taxon>
<taxon id="A/lesser_white-fronted_goose/Mississippi/22-041057-
001/2022|EPI_ISL_19608573|A_/_H5N1|Original||2.3.4.4b|08.12.2022||||12.12.2
024|USDA_APHIS_Veterinary_Services_Diagnostic_Virology_Laboratory_National_
Veterinary_Services_Laboratories||HA|4|A/lesser">
<date value="2022.9369863013699" direction="forwards" units="years"/>
</taxon>
<taxon id="A/lion/Peru/AIS0554/2023|EPI_ISL_17805999|A_/_H5N1|Original||2.3
.4.4b|08.02.2023|GISAID_EpiFlu_Data_Curator|||15.06.2023|||HA|4|A/lion/Peru
/AIS0554/2023_HA|EPI2597244|DNA_IN">
<date value="2023.1068493150685" direction="forwards" units="years"/>
</taxon>
<taxon id="A/magpie/Colorado/23-000401-
001/2022|EPI_ISL_19608574|A_/_H5N1|Original||2.3.4.4b|27.12.2022||||12.12.2
024|USDA_APHIS_Veterinary_Services_Diagnostic_Virology_Laboratory_National_
Veterinary_Services_Laboratories||HA|4|A/magpie/Colorado/23-000401-001">
<date value="2022.9890410958903" direction="forwards" units="years"/>
</taxon>
<taxon id="A/magpie/Colorado/23-002236-
002/2023|EPI_ISL_19608575|A_/_H5N1|Original||2.3.4.4b|11.01.2023||||12.12.2
024|USDA_APHIS_Veterinary_Services_Diagnostic_Virology_Laboratory_National_
Veterinary_Services_Laboratories||HA|4|A/magpie/Colorado/23-002236-002">
<date value="2023.0301369863014" direction="forwards" units="years"/>
</taxon>
<taxon id="A/magpie/Colorado/23-003934-
002/2023|EPI_ISL_19608576|A_/_H5N1|Original||2.3.4.4b|17.01.2023||||12.12.2
024|USDA_APHIS_Veterinary_Services_Diagnostic_Virology_Laboratory_National_
Veterinary_Services_Laboratories||HA|4|A/magpie/Colorado/23-003934-002">
<date value="2023.0465753424658" direction="forwards" units="years"/>
</taxon>
<taxon id="A/magpie/Colorado/23-004705-001-
/2023|EPI_ISL_17964848|A_/_H5N1|Original||2.3.4.4b|07.02.2023|Killian_Mary_
Lea||23-004705-001|06.07.2023|National_Veterinary_Services_Laboratories_-
_USDA|National_Veterinary_Services_Laboratories_-_USDA|HA|4|HA_A/magpie">
<date value="2023.1041095890412" direction="forwards" units="years"/>
</taxon>
<taxon id="A/magpie/Idaho/23-001085-
001/2023|EPI_ISL_19608578|A_/_H5N1|Original||2.3.4.4b|04.01.2023||||12.12.2
024|USDA_APHIS_Veterinary_Services_Diagnostic_Virology_Laboratory_National_
Veterinary_Services_Laboratories||HA|4|A/magpie/Idaho/23-001085-001/2023_">
<date value="2023.0109589041097" direction="forwards" units="years"/>
</taxon>
<taxon id="A/magpie/Idaho/23-011169-
002/2023|EPI_ISL_17964926|A_/_H5N1|Original||2.3.4.4b|28.03.2023|Killian_Ma
ry_Lea||23-011169-
002|06.07.2023|National_Veterinary_Services_Laboratories_-
_USDA|National_Veterinary_Services_Laboratories_-
_USDA|HA|4|HA_A/magpie/Ida">
<date value="2023.1534246575343" direction="forwards" units="years"/>
</taxon>
<taxon id="A/mallard/Montana/22-039947-
005/2022|EPI_ISL_19604023|A_/_H5N1|Original||2.3.4.4b|02.12.2022||||11.12.2

024|USDA_APHIS_Veterinary_Services_Diagnostic_Virology_Laboratory_National_
Veterinary_Services_Laboratories||HA|4|A/mallard/Montana/22-039947-005">
<date value="2022.9205479452055" direction="forwards" units="years"/>
</taxon>
<taxon id="A/merganser/Colorado/22-039962-
001/2022|EPI_ISL_19604070|A_/_H5N1|Original||2.3.4.4b|06.12.2022||||12.12.2
024|USDA_APHIS_Veterinary_Services_Diagnostic_Virology_Laboratory_National_
Veterinary_Services_Laboratories||HA|4|A/merganser/Colorado/22-0399">
<date value="2022.9315068493152" direction="forwards" units="years"/>
</taxon>
<taxon id="A/mottled_duck/South_Carolina/22-042180-
002/2022|EPI_ISL_19604312|A_/_H5N1|Original||2.3.4.4b|16.12.2022||||12.12.2
024|USDA_APHIS_Veterinary_Services_Diagnostic_Virology_Laboratory_National_
Veterinary_Services_Laboratories||HA|4|A/mottled_duck/Sout">
<date value="2022.958904109589" direction="forwards" units="years"/>
</taxon>
<taxon id="A/mute_swan/Rhode_Island/23-007092-
001/2023|EPI_ISL_19604294|A_/_H5N1|Original||2.3.4.4b|13.02.2023||||12.12.2
024|USDA_APHIS_Veterinary_Services_Diagnostic_Virology_Laboratory_National_
Veterinary_Services_Laboratories||HA|4|A/mute_swan/Rhode_Island">
<date value="2023.1205479452055" direction="forwards" units="years"/>
</taxon>
<taxon id="A/northern_shoveler/Arkansas/23-002690-
001/2023|EPI_ISL_19604370|A_/_H5N1|Original||2.3.4.4b|14.01.2023||||12.12.2
024|USDA_APHIS_Veterinary_Services_Diagnostic_Virology_Laboratory_National_
Veterinary_Services_Laboratories||HA|4|A/northern_shoveler/">
<date value="2023.0383561643835" direction="forwards" units="years"/>
</taxon>
<taxon id="A/pelican/Antofagasta/228318-
1/2022|EPI_ISL_17885928|A_/_H5N1|Original||2.3.4.4b|16.12.2022|GISAID_EpiFl
u_Data_Curator|||19.07.2023|||HA|4|A/pelican/Antofagasta/228318-
1/2022_HA|EPI2609318|DNA_IN">
<date value="2022.958904109589" direction="forwards" units="years"/>
</taxon>
<taxon id="A/pelican/Bio_Bio/236574/2023|EPI_ISL_18760061|A_/_H5N1|Original
||2.3.4.4b|13.02.2023||||14.01.2024|Emory_University__CEIRR_|Emory_Universi
ty__CEIRR_|HA|4|A/pelican/Bio_Bio/236574/2023_HA|EPI2913191|DNA_IN">
<date value="2023.1205479452055" direction="forwards" units="years"/>
</taxon>
<taxon id="A/pelican/Chile/229424-
4/2022|EPI_ISL_19391461|A_/_H5N1|Original||2.3.4.4b|22.12.2022||||09.09.202
4|CEIRS_Data_Processing_and_Coordinating_Center_Center_for_Research_on_Infl
uenza_Pathogenesis__CRIP_||HA|4|A/pelican/Chile/229424-
4/2022_HA|EPI3549636|DN">
<date value="2022.9753424657533" direction="forwards" units="years"/>
</taxon>
<taxon id="A/pelican/Costa_Rica/INC-0018-D0370-
23/2023|EPI_ISL_17965774|A_/_H5N1|Original||2.3.4.4b|26.01.2023|Soto-
Garita_Claudio|D0370-P|786338|18.07.2023|SENASA-
LANASEVE|Inciensa_Investigacion_y_Ensenanza_en_Nutricion_y_Salud|HA|4|A/pel
ican/Costa_Rica/INC-00">
<date value="2023.0712328767124" direction="forwards" units="years"/>
</taxon>
<taxon id="A/pelican/Costa_Rica/INC-0019-D0618-
23/2023|EPI_ISL_17965775|A_/_H5N1|Original||2.3.4.4b|10.02.2023|Soto-
Garita_Claudio|D0618-23|786339|18.07.2023|SENASA-
LANASEVE|Inciensa_Investigacion_y_Ensenanza_en_Nutricion_y_Salud|HA|4|A/pel
ican/Costa_Rica/INC-0">
<date value="2023.1123287671232" direction="forwards" units="years"/>
</taxon>

<taxon id="A/pelican/Costa_Rica/INC-0020-D0716-
23/2023|EPI_ISL_17965776|A_/_H5N1|Original||2.3.4.4b|10.02.2023|Soto-
Garita_Claudio|D0726-
23Tc|786340|18.07.2023|Servicio_Nacional_de_Salud_Animal__SENASA_|Inciensa_
Investigacion_y_Ensenanza_en_Nutricion_y_Salud|HA">
<date value="2023.1123287671232" direction="forwards" units="years"/>
</taxon>
<taxon id="A/pelican/Costa_Rica/INC-0021-D0729-
23/2023|EPI_ISL_17965777|A_/_H5N1|Original||2.3.4.4b|10.02.2023|Soto-
Garita_Claudio|D0729-23|786341|18.07.2023|SENASA-
LANASEVE|Inciensa_Investigacion_y_Ensenanza_en_Nutricion_y_Salud|HA|4|A/pel
ican/Costa_Rica/INC-0">
<date value="2023.1123287671232" direction="forwards" units="years"/>
</taxon>
<taxon id="A/pelican/Guatemala/23-003730-
001/2023|EPI_ISL_18863889|A_/_H5N1|Original||2.3.4.4b|27.01.2023|Killian_Ma
ry_Lea||23-003730-
001|05.02.2024|National_Veterinary_Services_Laboratories_-
_USDA|National_Veterinary_Services_Laboratories_-_USDA|HA|4|HA_A/pelic">
<date value="2023.0739726027398" direction="forwards" units="years"/>
</taxon>
<taxon id="A/pelican/Guatemala/23-003730-
002/2023|EPI_ISL_18863890|A_/_H5N1|Original||2.3.4.4b|27.01.2023|Killian_Ma
ry_Lea||23-003730-
002|05.02.2024|National_Veterinary_Services_Laboratories_-
_USDA|National_Veterinary_Services_Laboratories_-_USDA|HA|4|HA_A/pelic">
<date value="2023.0739726027398" direction="forwards" units="years"/>
</taxon>
<taxon id="A/pelican/Honduras/23-000009-
001/2022|EPI_ISL_17558875|A_/_H5N1|E1||2.3.4.4b|27.12.2022|Killian_Mary_Lea
||23-000009-
001|27.04.2023|Instituto_Hondureno_de_Investigaciones_Medico_Veterinarias_S
AG-SENASA|National_Veterinary_Services_Laboratories_-_USDA|H">
<date value="2022.9890410958903" direction="forwards" units="years"/>
</taxon>
<taxon id="A/pelican/Valparaiso/233091-
1/2023|EPI_ISL_17885930|A_/_H5N1|Original||2.3.4.4b|20.01.2023|GISAID_EpiFl
u_Data_Curator|||19.07.2023|||HA|4|A/pelican/Valparaiso/233091-
1/2023_HA|EPI2609327|DNA_IN">
<date value="2023.054794520548" direction="forwards" units="years"/>
</taxon>
<taxon id="A/pelican/Valparaiso/233091-
2/2023|EPI_ISL_17885956|A_/_H5N1|Original||2.3.4.4b|20.01.2023|GISAID_EpiFl
u_Data_Curator|||29.06.2023|||HA|4|A/pelican/Valparaiso/233091-
2/2023_HA|EPI2609431|DNA_IN">
<date value="2023.054794520548" direction="forwards" units="years"/>
</taxon>
<taxon id="A/pelican/Valparaiso/233418-
1/2023|EPI_ISL_17885932|A_/_H5N1|Original||2.3.4.4b|20.01.2023|GISAID_EpiFl
u_Data_Curator|||24.07.2023|||HA|4|A/pelican/Valparaiso/233418-
1/2023_HA|EPI2637126|DNA_IN">
<date value="2023.054794520548" direction="forwards" units="years"/>
</taxon>
<taxon id="A/pelican/Valparaiso/233447-
2/2023|EPI_ISL_17885985|A_/_H5N1|Original||2.3.4.4b|21.01.2023|GISAID_EpiFl
u_Data_Curator|||19.07.2023|||HA|4|A/pelican/Valparaiso/233447-
2/2023_HA|EPI2609614|DNA_IN">
<date value="2023.0575342465754" direction="forwards" units="years"/>
</taxon>
<taxon id="A/pelican/Valparaiso/233450-
1/2023|EPI_ISL_17885983|A_/_H5N1|Original||2.3.4.4b|23.01.2023|GISAID_EpiFl

u_Data_Curator|||19.07.2023|||HA|4|A/pelican/Valparaiso/233450-
1/2023_HA|EPI2609600|DNA_IN">
<date value="2023.0630136986301" direction="forwards" units="years"/>
</taxon>
<taxon id="A/pelican/Valparaiso/234040/2023|EPI_ISL_17885982|A_/_H5N1|Original||2.3.4.4b|25.01.2023|GISAID_EpiFlu_Data_Curator|||19.07.2023|||HA|4|A/pelican/Valparaiso/234040/2023_HA|EPI2609588|DNA_IN">
<date value="2023.0684931506848" direction="forwards" units="years"/>
</taxon>
<taxon id="A/peregrine_falcon/Antofagasta/235144-
1/2023|EPI_ISL_19410283|A_/_H5N1|Original||2.3.4.4b|03.02.2023||||12.09.2024|Emory_University__CEIRR_|Emory_University__CEIRR_|HA|4|A/peregrine_falcon/Antofagasta/235144-1/2023_HA|EPI3555314|DNA_IN">
<date value="2023.0931506849315" direction="forwards" units="years"/>
</taxon>
<taxon id="A/peregrine_falcon/BC/AIVPHL-
322/2022|EPI_ISL_17051459|A_/_H5N1|Original||2.3.4.4b|12.12.2022|Russell_Shannon_Laurel|||28.02.2023|B.C._Centre_for_Disease_Control|British_Columbia_Centre_for_Disease_Control|HA|4|A/peregrine_falcon/BC/AIVPHL-322/2022_HA">
<date value="2022.9479452054795" direction="forwards" units="years"/>
</taxon>
<taxon id="A/peregrine_falcon/California/23-011277-
001/2023|EPI_ISL_17964933|A_/_H5N1|Original||2.3.4.4b|30.03.2023|Killian_Mary_Lea||23-011277-
001|06.07.2023|National_Veterinary_Services_Laboratories_-
_USDA|National_Veterinary_Services_Laboratories_-_USDA|HA|4|">
<date value="2023.158904109589" direction="forwards" units="years"/>
</taxon>
<taxon id="A/peregrine_falcon/Michigan/23-003553-
001/2023|EPI_ISL_19604230|A_/_H5N1|Original||2.3.4.4b|26.01.2023||||12.12.2024|USDA_APHIS_Veterinary_Services_Diagnostic_Virology_Laboratory_National_Veterinary_Services_Laboratories||HA|4|A/peregrine_falcon/Mi">
<date value="2023.0712328767124" direction="forwards" units="years"/>
</taxon>
<taxon id="A/peruvian_booby/Coquimbo/242304/2023|EPI_ISL_19410277|A_/_H5N1|Original||2.3.4.4b|15.03.2023||||12.09.2024|Emory_University__CEIRR_||HA|4|A/peruvian_booby/Coquimbo/242304/2023_HA|EPI3555275|DNA_IN">
<date value="2023.1178082191782" direction="forwards" units="years"/>
</taxon>
<taxon id="A/peruvian_booby/Peru/CAL-INS-
007/2023|EPI_ISL_17777528|A_/_H5N1|Original||2.3.4.4b|17.03.2023|Padilla_Rojas_Carlos_Patricio||1375|01.07.2023|Laboratorio_de_Virus_Respiratorios_Centro_Nacional_de_Salud_Publica|Instituto_Nacional_de_Salud_Peru|HA|4|Pe">
<date value="2023.123287671233" direction="forwards" units="years"/>
</taxon>
<taxon id="A/peruvian_booby/Peru/CAL-INS-
008/2023|EPI_ISL_17777529|A_/_H5N1|Original||2.3.4.4b|17.03.2023|Padilla_Rojas_Carlos_Patricio||1375|01.07.2023|Laboratorio_de_Virus_Respiratorios_Centro_Nacional_de_Salud_Publica|Instituto_Nacional_de_Salud_Peru|HA|4|Pe">
<date value="2023.123287671233" direction="forwards" units="years"/>
</taxon>
<taxon id="A/peruvian_booby/Peru/LIM-INS-
004/2023|EPI_ISL_17777525|A_/_H5N1|Original||2.3.4.4b|03.04.2023|Padilla_Rojas_Carlos_Patricio||1375|01.07.2023|Laboratorio_de_Virus_Respiratorios_Centro_Nacional_de_Salud_Publica|Instituto_Nacional_de_Salud_Peru|HA|4|Pe">
<date value="2023.2547945205479" direction="forwards" units="years"/>
</taxon>
<taxon id="A/peruvian_booby/Peru/LIM-INS-
005/2023|EPI_ISL_17777526|A_/_H5N1|Original||2.3.4.4b|20.03.2023|Padilla_Rojas_Carlos_Patricio||1375|01.07.2023|Laboratorio_de_Virus_Respiratorios_Centro_Nacional_de_Salud_Publica|Instituto_Nacional_de_Salud_Peru|HA|4|Pe">

<date value="2023.131506849315" direction="forwards" units="years"/>
</taxon>
<taxon id="A/peruvian_booby/Peru/LIM-INS-012/2023|EPI_ISL_17777533|A_/_H5N1|Original||2.3.4.4b|12.04.2023|Padilla_Rojas_Carlos_Patricio||1375|01.07.2023|Laboratorio_de_Virus_Respiratorios_Centro_Nacional_de_Salud_Publica|Instituto_Nacional_de_Salud_Peru|HA|4|Pe">
<date value="2023.2794520547945" direction="forwards" units="years"/>
</taxon>
<taxon id="A/pinniped/Uruguay/P10_8923/2023|EPI_ISL_19070481|A_/_H5N1|Original||2.3.4.4b|08.09.2023||||24.04.2024|Universidad_de_la_Republica_Facultad_de_Ciencias_Genetica_Evolutiva/Division_de_Laboratorios_Veterinarios_Plataforma_Genomica||HA|4|A/pinniped/Uru">
<date value="2023.6876712328767" direction="forwards" units="years"/>
</taxon>
<taxon id="A/pinniped/Uruguay/P13_11923/2023|EPI_ISL_19070482|A_/_H5N1|Original||2.3.4.4b|11.09.2023||||24.04.2024|Universidad_de_la_Republica_Facultad_de_Ciencias_Genetica_Evolutiva/Division_de_Laboratorios_Veterinarios_Plataforma_Genomica||HA|4|A/pinniped/Ur">
<date value="2023.695890410959" direction="forwards" units="years"/>
</taxon>
<taxon id="A/pinniped/Uruguay/P14_11923/2023|EPI_ISL_19070483|A_/_H5N1|Original||2.3.4.4b|11.09.2023||||24.04.2024|Universidad_de_la_Republica_Facultad_de_Ciencias_Genetica_Evolutiva/Division_de_Laboratorios_Veterinarios_Plataforma_Genomica||HA|4|A/pinniped/Ur">
<date value="2023.695890410959" direction="forwards" units="years"/>
</taxon>
<taxon id="A/pinniped/Uruguay/P15_14923/2023|EPI_ISL_19070484|A_/_H5N1|Original||2.3.4.4b|14.09.2023||||24.04.2024|Universidad_de_la_Republica_Facultad_de_Ciencias_Genetica_Evolutiva/Division_de_Laboratorios_Veterinarios_Plataforma_Genomica||HA|4|A/pinniped/Ur">
<date value="2023.704109589041" direction="forwards" units="years"/>
</taxon>
<taxon id="A/pinniped/Uruguay/P17_14923/2023|EPI_ISL_19070485|A_/_H5N1|Original||2.3.4.4b|14.09.2023||||24.04.2024|Universidad_de_la_Republica_Facultad_de_Ciencias_Genetica_Evolutiva/Division_de_Laboratorios_Veterinarios_Plataforma_Genomica||HA|4|A/pinniped/Ur">
<date value="2023.704109589041" direction="forwards" units="years"/>
</taxon>
<taxon id="A/pinniped/Uruguay/P18_14923/2023|EPI_ISL_19070486|A_/_H5N1|Original||2.3.4.4b|14.09.2023||||24.04.2024|Universidad_de_la_Republica_Facultad_de_Ciencias_Genetica_Evolutiva/Division_de_Laboratorios_Veterinarios_Plataforma_Genomica||HA|4|A/pinniped/Ur">
<date value="2023.704109589041" direction="forwards" units="years"/>
</taxon>
<taxon id="A/pinniped/Uruguay/P26_21023/2023|EPI_ISL_19070488|A_/_H5N1|Original||2.3.4.4b|02.10.2023||||24.04.2024|Universidad_de_la_Republica_Facultad_de_Ciencias_Genetica_Evolutiva/Division_de_Laboratorios_Veterinarios_Plataforma_Genomica||HA|4|A/pinniped/Ur">
<date value="2023.6986301369864" direction="forwards" units="years"/>
</taxon>
<taxon id="A/pinniped/Uruguay/P4_6923/2023|EPI_ISL_19070498|A_/_H5N1|Original||2.3.4.4b|06.09.2023||||24.04.2024|Universidad_de_la_Republica_Facultad_de_Ciencias_Genetica_Evolutiva/Division_de_Laboratorios_Veterinarios_Plataforma_Genomica||HA|4|A/pinniped/Urug">
<date value="2023.682191780822" direction="forwards" units="years"/>
</taxon>
<taxon id="A/pinniped/Uruguay/P5_6923/2023|EPI_ISL_19070489|A_/_H5N1|Original||2.3.4.4b|06.09.2023||||24.04.2024|Universidad_de_la_Republica_Facultad_de_Ciencias_Genetica_Evolutiva/Division_de_Laboratorios_Veterinarios_Plataforma_Genomica||HA|4|A/pinniped/Urug">
<date value="2023.682191780822" direction="forwards" units="years"/>

</taxon>
<taxon id="A/pinniped/Uruguay/P6_6923/2023|EPI_ISL_19070490|A_/_H5N1|Origin
al||2.3.4.4b|06.09.2023||||24.04.2024|Universidad_de_la_Republica_Facultad_
de_Ciencias_Genetica_Evolutiva/Division_de_Laboratorios_Veterinarios_Plataf
orma_Genomica||HA|4|A/pinniped/Urug">
<date value="2023.682191780822" direction="forwards" units="years"/>
</taxon>
<taxon id="A/pinniped/Uruguay/P7_6923/2023|EPI_ISL_19070491|A_/_H5N1|Origin
al||2.3.4.4b|06.09.2023||||24.04.2024|Universidad_de_la_Republica_Facultad_
de_Ciencias_Genetica_Evolutiva/Division_de_Laboratorios_Veterinarios_Plataf
orma_Genomica||HA|4|A/pinniped/Urug">
<date value="2023.682191780822" direction="forwards" units="years"/>
</taxon>
<taxon id="A/pinniped/Uruguay/P8_8923/2023|EPI_ISL_19070492|A_/_H5N1|Origin
al||2.3.4.4b|08.09.2023||||24.04.2024|Universidad_de_la_Republica_Facultad_
de_Ciencias_Genetica_Evolutiva/Division_de_Laboratorios_Veterinarios_Plataf
orma_Genomica||HA|4|A/pinniped/Urug">
<date value="2023.6876712328767" direction="forwards" units="years"/>
</taxon>
<taxon id="A/porpoise/Antofagasta/SJCEIRR-
2465061/2023|EPI_ISL_18777129|A_/_H5N1|Original||2.3.4.4b|31.03.2023||||16.
01.2024|Emory_University__CEIRR_|Emory_University__CEIRR_|HA|4|A/porpoise/A
ntofagasta/SJCEIRR-2465061/2023|EPI2921496|DNA_IN">
<date value="2023.1616438356164" direction="forwards" units="years"/>
</taxon>
<taxon id="A/porpoise/Antofagasta/SJCEIRR-
2465062/2023|EPI_ISL_18777141|A_/_H5N1|Original||2.3.4.4b|31.03.2023||||16.
01.2024|Emory_University__CEIRR_|Emory_University__CEIRR_|HA|4|A/porpoise/A
ntofagasta/SJCEIRR-2465062/2023|EPI2921533|DNA_IN">
<date value="2023.1616438356164" direction="forwards" units="years"/>
</taxon>
<taxon id="A/porpoise/Atacama/SJCEIRR-
245355/2023|EPI_ISL_18777140|A_/_H5N1|Original||2.3.4.4b|27.03.2023||||16.0
1.2024|Emory_University__CEIRR_|Emory_University__CEIRR_|HA|4|A/porpoise/At
acama/SJCEIRR-245355/2023|EPI2921527|DNA_IN">
<date value="2023.150684931507" direction="forwards" units="years"/>
</taxon>
<taxon id="A/raven/California/23-003756-
001/2023|EPI_ISL_19604281|A_/_H5N1|Original||2.3.4.4b|29.01.2023||||12.12.2
024|USDA_APHIS_Veterinary_Services_Diagnostic_Virology_Laboratory_National_
Veterinary_Services_Laboratories||HA|4|A/raven/California/23-003756-0">
<date value="2023.0794520547945" direction="forwards" units="years"/>
</taxon>
<taxon id="A/raven/Colorado/23-003562-
001/2023|EPI_ISL_19604258|A_/_H5N1|Original||2.3.4.4b|27.01.2023||||12.12.2
024|USDA_APHIS_Veterinary_Services_Diagnostic_Virology_Laboratory_National_
Veterinary_Services_Laboratories||HA|4|A/raven/Colorado/23-003562-001/2">
<date value="2023.0739726027398" direction="forwards" units="years"/>
</taxon>
<taxon id="A/raven/Colorado/23-003563-
001/2023|EPI_ISL_19604257|A_/_H5N1|Original||2.3.4.4b|25.01.2023||||12.12.2
024|USDA_APHIS_Veterinary_Services_Diagnostic_Virology_Laboratory_National_
Veterinary_Services_Laboratories||HA|4|A/raven/Colorado/23-003563-001/2">
<date value="2023.0684931506848" direction="forwards" units="years"/>
</taxon>
<taxon id="A/raven/Montana/23-003666-
002/2023|EPI_ISL_19604256|A_/_H5N1|Original||2.3.4.4b|30.01.2023||||12.12.2
024|USDA_APHIS_Veterinary_Services_Diagnostic_Virology_Laboratory_National_
Veterinary_Services_Laboratories||HA|4|A/raven/Montana/23-003666-002/202">
<date value="2023.0821917808219" direction="forwards" units="years"/>
</taxon>

<taxon id="A/red-tailed_hawk/BC/AIVPHL-339/2022|EPI_ISL_17051467|A_/_H5N1|Original||2.3.4.4b|19.12.2022|Russell_Shannon_Laurel|||28.02.2023|B.C._Centre_for_Disease_Control|British_Columbia_Centre_for_Disease_Control|HA|4|A/red-tailed_hawk/BC/AIVPHL-339/2022_HA|E">
<date value="2022.9671232876713" direction="forwards" units="years"/>
</taxon>
<taxon id="A/red-tailed_hawk/California/23-006644-003/2023|EPI_ISL_17964878|A_/_H5N1|Original||2.3.4.4b|14.02.2023|Killian_Mary_Lea||23-006644-003|06.07.2023|National_Veterinary_Services_Laboratories_-_USDA|National_Veterinary_Services_Laboratories_-_USDA|HA|4|H">
<date value="2023.123287671233" direction="forwards" units="years"/>
</taxon>
<taxon id="A/red-tailed_hawk/Colorado/23-005618-001/2023|EPI_ISL_17964859|A_/_H5N1|Original||2.3.4.4b|14.02.2023|Killian_Mary_Lea||23-005618-001|06.07.2023|National_Veterinary_Services_Laboratories_-_USDA|National_Veterinary_Services_Laboratories_-_USDA|HA|4|HA_">
<date value="2023.123287671233" direction="forwards" units="years"/>
</taxon>
<taxon id="A/red-tailed_hawk/Colorado/23-006671-001/2023|EPI_ISL_17964882|A_/_H5N1|Original||2.3.4.4b|18.02.2023|Killian_Mary_Lea||23-006671-001|06.07.2023|National_Veterinary_Services_Laboratories_-_USDA|National_Veterinary_Services_Laboratories_-_USDA|HA|4|HA_">
<date value="2023.1342465753426" direction="forwards" units="years"/>
</taxon>
<taxon id="A/red-tailed_hawk/Colorado/23-008901-001/2023|EPI_ISL_17964906|A_/_H5N1|Original||2.3.4.4b|15.03.2023|Killian_Mary_Lea||23-008901-001|06.07.2023|National_Veterinary_Services_Laboratories_-_USDA|National_Veterinary_Services_Laboratories_-_USDA|HA|4|HA_">
<date value="2023.1178082191782" direction="forwards" units="years"/>
</taxon>
<taxon id="A/red-tailed_hawk/Kansas/23-009211-001/2023|EPI_ISL_17964913|A_/_H5N1|Original||2.3.4.4b|15.03.2023|Killian_Mary_Lea||23-009211-001|06.07.2023|National_Veterinary_Services_Laboratories_-_USDA|National_Veterinary_Services_Laboratories_-_USDA|HA|4|HA_A/">
<date value="2023.1178082191782" direction="forwards" units="years"/>
</taxon>
<taxon id="A/red-tailed_hawk/Kentucky/W23-143/2022|EPI_ISL_17424631|A_/_H5N1|||2.3.4.4b|22.12.2022||||08.04.2023|St._Jude_Center_of_Excellence_for_Influenza_Research_and_Response_-_CEIRR_Influenza_Data_Processing_and_Communication_Center|St._Jude_Center_of_Excell">
<date value="2022.9753424657533" direction="forwards" units="years"/>
</taxon>
<taxon id="A/red-tailed_hawk/Minnesota/23-010018-001/2023|EPI_ISL_17964919|A_/_H5N1|Original||2.3.4.4b|26.03.2023|Killian_Mary_Lea||23-010018-001|06.07.2023|National_Veterinary_Services_Laboratories_-_USDA|National_Veterinary_Services_Laboratories_-_USDA|HA|4|HA">
<date value="2023.1479452054793" direction="forwards" units="years"/>
</taxon>
<taxon id="A/red-tailed_hawk/Montana/23-011216-001/2023|EPI_ISL_17964929|A_/_H5N1|Original||2.3.4.4b|03.04.2023|Killian_Mary_Lea||23-011216-001|06.07.2023|National_Veterinary_Services_Laboratories_-_USDA|National_Veterinary_Services_Laboratories_-_USDA|HA|4|HA_A">
<date value="2023.2547945205479" direction="forwards" units="years"/>

</taxon>
<taxon id="A/red-tailed_hawk/Nevada/23-012114-001/2023|EPI_ISL_17964944|A_/_H5N1|Original||2.3.4.4b|31.03.2023|Killian_Mary_Lea||23-012114-001|06.07.2023|National_Veterinary_Services_Laboratories_-_USDA|National_Veterinary_Services_Laboratories_-_USDA|HA|4|HA_A/">
<date value="2023.1616438356164" direction="forwards" units="years"/>
</taxon>
<taxon id="A/red-tailed_hawk/South_Dakota/23-012031-001/2023|EPI_ISL_17964942|A_/_H5N1|Original||2.3.4.4b|11.04.2023|Killian_Mary_Lea||23-012031-001|06.07.2023|National_Veterinary_Services_Laboratories_-_USDA|National_Veterinary_Services_Laboratories_-_USDA|HA|4">
<date value="2023.2767123287672" direction="forwards" units="years"/>
</taxon>
<taxon id="A/red_necked_phalarope/Oregon/23-000773-001/2023|EPI_ISL_19604238|A_/_H5N1|Original||2.3.4.4b|04.01.2023||||12.12.2024|USDA_APHIS_Veterinary_Services_Diagnostic_Virology_Laboratory_National_Veterinary_Services_Laboratories||HA|4|A/red_necked_phalar">
<date value="2023.0109589041097" direction="forwards" units="years"/>
</taxon>
<taxon id="A/red_tailed_hawk/California/22-041402-001/2022|EPI_ISL_19604249|A_/_H5N1|Original||2.3.4.4b|01.12.2022||||12.12.2024|USDA_APHIS_Veterinary_Services_Diagnostic_Virology_Laboratory_National_Veterinary_Services_Laboratories||HA|4|A/red_tailed_hawk/Ca">
<date value="2022.9178082191781" direction="forwards" units="years"/>
</taxon>
<taxon id="A/red_tailed_hawk/California/23-000278-001/2022|EPI_ISL_19604252|A_/_H5N1|Original||2.3.4.4b|14.12.2022||||12.12.2024|USDA_APHIS_Veterinary_Services_Diagnostic_Virology_Laboratory_National_Veterinary_Services_Laboratories||HA|4|A/red_tailed_hawk/Ca">
<date value="2022.9534246575342" direction="forwards" units="years"/>
</taxon>
<taxon id="A/red_tailed_hawk/California/23-000279-002/2022|EPI_ISL_19604253|A_/_H5N1|Original||2.3.4.4b|15.12.2022||||12.12.2024|USDA_APHIS_Veterinary_Services_Diagnostic_Virology_Laboratory_National_Veterinary_Services_Laboratories||HA|4|A/red_tailed_hawk/Ca">
<date value="2022.9561643835616" direction="forwards" units="years"/>
</taxon>
<taxon id="A/red_tailed_hawk/California/23-001055-001/2022|EPI_ISL_19604254|A_/_H5N1|Original||2.3.4.4b|21.12.2022||||12.12.2024|USDA_APHIS_Veterinary_Services_Diagnostic_Virology_Laboratory_National_Veterinary_Services_Laboratories||HA|4|A/red_tailed_hawk/Ca">
<date value="2022.972602739726" direction="forwards" units="years"/>
</taxon>
<taxon id="A/red_tailed_hawk/California/23-001856-004/2022|EPI_ISL_19604378|A_/_H5N1|Original||2.3.4.4b|19.12.2022||||12.12.2024|USDA_APHIS_Veterinary_Services_Diagnostic_Virology_Laboratory_National_Veterinary_Services_Laboratories||HA|4|A/red_tailed_hawk/Ca">
<date value="2022.9671232876713" direction="forwards" units="years"/>
</taxon>
<taxon id="A/red_tailed_hawk/California/23-004363-001/2023|EPI_ISL_19604802|A_/_H5N1|Original||2.3.4.4b|25.01.2023||||12.12.2024|USDA_APHIS_Veterinary_Services_Diagnostic_Virology_Laboratory_National_Veterinary_Services_Laboratories||HA|4|A/red_tailed_hawk/Ca">
<date value="2023.0684931506848" direction="forwards" units="years"/>
</taxon>
<taxon id="A/red_tailed_hawk/Colorado/22-041828-001/2022|EPI_ISL_19605765|A_/_H5N1|Original||2.3.4.4b|20.12.2022||||12.12.2024|USDA_APHIS_Veterinary_Services_Diagnostic_Virology_Laboratory_National_Veterinary_Services_Laboratories||HA|4|A/red_tailed_hawk/Colo">

<date value="2022.9698630136986" direction="forwards" units="years"/>
</taxon>
<taxon id="A/red_tailed_hawk/Colorado/22-041830-001/2022|EPI_ISL_19605766|A_/_H5N1|Original||2.3.4.4b|20.12.2022||||12.12.2024|USDA_APHIS_Veterinary_Services_Diagnostic_Virology_Laboratory_National_Veterinary_Services_Laboratories||HA|4|A/red_tailed_hawk/Colo">
<date value="2022.9698630136986" direction="forwards" units="years"/>
</taxon>
<taxon id="A/red_tailed_hawk/Colorado/22-042170-002/2022|EPI_ISL_19605768|A_/_H5N1|Original||2.3.4.4b|25.12.2022||||12.12.2024|USDA_APHIS_Veterinary_Services_Diagnostic_Virology_Laboratory_National_Veterinary_Services_Laboratories||HA|4|A/red_tailed_hawk/Colo">
<date value="2022.9835616438356" direction="forwards" units="years"/>
</taxon>
<taxon id="A/red_tailed_hawk/Colorado/23-000403-001/2023|EPI_ISL_19605769|A_/_H5N1|Original||2.3.4.4b|01.01.2023||||12.12.2024|USDA_APHIS_Veterinary_Services_Diagnostic_Virology_Laboratory_National_Veterinary_Services_Laboratories||HA|4|A/red_tailed_hawk/Colo">
<date value="2023.0027397260274" direction="forwards" units="years"/>
</taxon>
<taxon id="A/red_tailed_hawk/Colorado/23-000494-001/2023|EPI_ISL_19605770|A_/_H5N1|Original||2.3.4.4b|04.01.2023||||12.12.2024|USDA_APHIS_Veterinary_Services_Diagnostic_Virology_Laboratory_National_Veterinary_Services_Laboratories||HA|4|A/red_tailed_hawk/Colo">
<date value="2023.0109589041097" direction="forwards" units="years"/>
</taxon>
<taxon id="A/red_tailed_hawk/Colorado/23-000498-001/2023|EPI_ISL_19605771|A_/_H5N1|Original||2.3.4.4b|04.01.2023||||12.12.2024|USDA_APHIS_Veterinary_Services_Diagnostic_Virology_Laboratory_National_Veterinary_Services_Laboratories||HA|4|A/red_tailed_hawk/Colo">
<date value="2023.0109589041097" direction="forwards" units="years"/>
</taxon>
<taxon id="A/red_tailed_hawk/Colorado/23-001466-001/2023|EPI_ISL_19605772|A_/_H5N1|Original||2.3.4.4b|05.01.2023||||12.12.2024|USDA_APHIS_Veterinary_Services_Diagnostic_Virology_Laboratory_National_Veterinary_Services_Laboratories||HA|4|A/red_tailed_hawk/Colo">
<date value="2023.013698630137" direction="forwards" units="years"/>
</taxon>
<taxon id="A/red_tailed_hawk/Colorado/23-001466-002/2023|EPI_ISL_19605773|A_/_H5N1|Original||2.3.4.4b|05.01.2023||||12.12.2024|USDA_APHIS_Veterinary_Services_Diagnostic_Virology_Laboratory_National_Veterinary_Services_Laboratories||HA|4|A/red_tailed_hawk/Colo">
<date value="2023.013698630137" direction="forwards" units="years"/>
</taxon>
<taxon id="A/red_tailed_hawk/Colorado/23-001574-001/2023|EPI_ISL_19605775|A_/_H5N1|Original||2.3.4.4b|10.01.2023||||12.12.2024|USDA_APHIS_Veterinary_Services_Diagnostic_Virology_Laboratory_National_Veterinary_Services_Laboratories||HA|4|A/red_tailed_hawk/Colo">
<date value="2023.027397260274" direction="forwards" units="years"/>
</taxon>
<taxon id="A/red_tailed_hawk/Colorado/23-001576-001/2023|EPI_ISL_19605725|A_/_H5N1|Original||2.3.4.4b|10.01.2023||||12.12.2024|USDA_APHIS_Veterinary_Services_Diagnostic_Virology_Laboratory_National_Veterinary_Services_Laboratories||HA|4|A/red_tailed_hawk/Colo">
<date value="2023.027397260274" direction="forwards" units="years"/>
</taxon>
<taxon id="A/red_tailed_hawk/Colorado/23-002236-001/2023|EPI_ISL_19605724|A_/_H5N1|Original||2.3.4.4b|11.01.2023||||12.12.2024|USDA_APHIS_Veterinary_Services_Diagnostic_Virology_Laboratory_National_Veterinary_Services_Laboratories||HA|4|A/red_tailed_hawk/Colo">
<date value="2023.0301369863014" direction="forwards" units="years"/>

</taxon>
<taxon id="A/red_tailed_hawk/Colorado/23-002918-
002/2023|EPI_ISL_19605723|A_/_H5N1|Original||2.3.4.4b|21.01.2023||||12.12.2
024|USDA_APHIS_Veterinary_Services_Diagnostic_Virology_Laboratory_National_
Veterinary_Services_Laboratories||HA|4|A/red_tailed_hawk/Colo">
<date value="2023.0575342465754" direction="forwards" units="years"/>
</taxon>
<taxon id="A/red_tailed_hawk/Colorado/23-003934-
003/2023|EPI_ISL_19605711|A_/_H5N1|Original||2.3.4.4b|17.01.2023||||12.12.2
024|USDA_APHIS_Veterinary_Services_Diagnostic_Virology_Laboratory_National_
Veterinary_Services_Laboratories||HA|4|A/red_tailed_hawk/Colo">
<date value="2023.0465753424658" direction="forwards" units="years"/>
</taxon>
<taxon id="A/red_tailed_hawk/Colorado/23-004223-
001/2023|EPI_ISL_19605701|A_/_H5N1|Original||2.3.4.4b|31.01.2023||||12.12.2
024|USDA_APHIS_Veterinary_Services_Diagnostic_Virology_Laboratory_National_
Veterinary_Services_Laboratories||HA|4|A/red_tailed_hawk/Colo">
<date value="2023.0849315068492" direction="forwards" units="years"/>
</taxon>
<taxon id="A/red_tailed_hawk/Colorado/23-004224-
001/2023|EPI_ISL_19605702|A_/_H5N1|Original||2.3.4.4b|31.01.2023||||12.12.2
024|USDA_APHIS_Veterinary_Services_Diagnostic_Virology_Laboratory_National_
Veterinary_Services_Laboratories||HA|4|A/red_tailed_hawk/Colo">
<date value="2023.0849315068492" direction="forwards" units="years"/>
</taxon>
<taxon id="A/red_tailed_hawk/Missouri/23-001518-
001/2022|EPI_ISL_19605823|A_/_H5N1|Original||2.3.4.4b|21.12.2022||||12.12.2
024|USDA_APHIS_Veterinary_Services_Diagnostic_Virology_Laboratory_National_
Veterinary_Services_Laboratories||HA|4|A/red_tailed_hawk/Miss">
<date value="2022.972602739726" direction="forwards" units="years"/>
</taxon>
<taxon id="A/red_tailed_hawk/Montana/23-002509-
001/2023|EPI_ISL_19605805|A_/_H5N1|Original||2.3.4.4b|18.01.2023||||12.12.2
024|USDA_APHIS_Veterinary_Services_Diagnostic_Virology_Laboratory_National_
Veterinary_Services_Laboratories||HA|4|A/red_tailed_hawk/Monta">
<date value="2023.0493150684931" direction="forwards" units="years"/>
</taxon>
<taxon id="A/red_tailed_hawk/Oregon/22-040812-
001/2022|EPI_ISL_19605593|A_/_H5N1|Original||2.3.4.4b|13.12.2022||||12.12.2
024|USDA_APHIS_Veterinary_Services_Diagnostic_Virology_Laboratory_National_
Veterinary_Services_Laboratories||HA|4|A/red_tailed_hawk/Oregon">
<date value="2022.9506849315069" direction="forwards" units="years"/>
</taxon>
<taxon id="A/red_tailed_hawk/Oregon/23-001496-
001/2022|EPI_ISL_19605594|A_/_H5N1|Original||2.3.4.4b|28.12.2022||||12.12.2
024|USDA_APHIS_Veterinary_Services_Diagnostic_Virology_Laboratory_National_
Veterinary_Services_Laboratories||HA|4|A/red_tailed_hawk/Oregon">
<date value="2022.9917808219177" direction="forwards" units="years"/>
</taxon>
<taxon id="A/red_tailed_hawk/Oregon/23-003379-
001/2023|EPI_ISL_19605596|A_/_H5N1|Original||2.3.4.4b|20.01.2023||||12.12.2
024|USDA_APHIS_Veterinary_Services_Diagnostic_Virology_Laboratory_National_
Veterinary_Services_Laboratories||HA|4|A/red_tailed_hawk/Oregon">
<date value="2023.054794520548" direction="forwards" units="years"/>
</taxon>
<taxon id="A/red_tailed_hawk/Oregon/23-005360-
001/2022|EPI_ISL_19605597|A_/_H5N1|Original||2.3.4.4b|15.12.2022||||12.12.2
024|USDA_APHIS_Veterinary_Services_Diagnostic_Virology_Laboratory_National_
Veterinary_Services_Laboratories||HA|4|A/red_tailed_hawk/Oregon">
<date value="2022.9561643835616" direction="forwards" units="years"/>
</taxon>

<taxon id="A/red_tailed_hawk/South_Dakota/23-002914-001/2023|EPI_ISL_19605605|A_/_H5N1|Original||2.3.4.4b|17.01.2023||||12.12.2024|USDA_APHIS_Veterinary_Services_Diagnostic_Virology_Laboratory_National_Veterinary_Services_Laboratories||HA|4|A/red_tailed_hawk/">
<date value="2023.0465753424658" direction="forwards" units="years"/>
</taxon>
<taxon id="A/red_tailed_hawk/Wyoming/22-042090-001/2022|EPI_ISL_19605483|A_/_H5N1|Original||2.3.4.4b|22.12.2022||||12.12.2024|USDA_APHIS_Veterinary_Services_Diagnostic_Virology_Laboratory_National_Veterinary_Services_Laboratories||HA|4|A/red_tailed_hawk/Wyomi">
<date value="2022.9753424657533" direction="forwards" units="years"/>
</taxon>
<taxon id="A/rosss_goose/Arkansas/23-001508-001/2023|EPI_ISL_19605678|A_/_H5N1|Original||2.3.4.4b|06.01.2023||||12.12.2024|USDA_APHIS_Veterinary_Services_Diagnostic_Virology_Laboratory_National_Veterinary_Services_Laboratories||HA|4|A/rosss_goose/Arkansas/23-">
<date value="2023.0164383561644" direction="forwards" units="years"/>
</taxon>
<taxon id="A/rosss_goose/California/23-000936-001/2022|EPI_ISL_19605681|A_/_H5N1|Original||2.3.4.4b|16.12.2022||||12.12.2024|USDA_APHIS_Veterinary_Services_Diagnostic_Virology_Laboratory_National_Veterinary_Services_Laboratories||HA|4|A/rosss_goose/California">
<date value="2022.958904109589" direction="forwards" units="years"/>
</taxon>
<taxon id="A/rosss_goose/California/23-001856-002/2022|EPI_ISL_19605682|A_/_H5N1|Original||2.3.4.4b|13.12.2022||||12.12.2024|USDA_APHIS_Veterinary_Services_Diagnostic_Virology_Laboratory_National_Veterinary_Services_Laboratories||HA|4|A/rosss_goose/California">
<date value="2022.9506849315069" direction="forwards" units="years"/>
</taxon>
<taxon id="A/rosss_goose/Kansas/22-040832-001/2022|EPI_ISL_19605685|A_/_H5N1|Original||2.3.4.4b|07.12.2022||||12.12.2024|USDA_APHIS_Veterinary_Services_Diagnostic_Virology_Laboratory_National_Veterinary_Services_Laboratories||HA|4|A/rosss_goose/Kansas/22-0408">
<date value="2022.9342465753425" direction="forwards" units="years"/>
</taxon>
<taxon id="A/rosss_goose/Louisiana/23-000409-010/2022|EPI_ISL_19608589|A_/_H5N1|Original||2.3.4.4b|02.12.2022||||12.12.2024|USDA_APHIS_Veterinary_Services_Diagnostic_Virology_Laboratory_National_Veterinary_Services_Laboratories||HA|4|A/rosss_goose/Louisiana/2">
<date value="2022.9205479452055" direction="forwards" units="years"/>
</taxon>
<taxon id="A/rough-legged_hawk/BC/AIVPHL-352/2023|EPI_ISL_17051475|A_/_H5N1|Original||2.3.4.4b|03.01.2023|Russell_Shannon_Laurel|||28.02.2023|B.C._Centre_for_Disease_Control|British_Columbia_Centre_for_Disease_Control|HA|4|A/rough-legged_hawk/BC/AIVPHL-352/2023_">
<date value="2023.0082191780823" direction="forwards" units="years"/>
</taxon>
<taxon id="A/royal_tern/Argentina/CH-PD036/2023|EPI_ISL_19466181|A_/_H5N1|Original||2.3.4.4b|10.10.2023||||08.10.2024|Instituto_Nacional_de_Tecnologia_Agropecuaria_Instituto_de_Virologia_e_Innovaciones_Tecnologicas||HA|4|A/royal_tern/Argentina/CH-PD036/2023_HA|E">
<date value="2023.7205479452055" direction="forwards" units="years"/>
</taxon>
<taxon id="A/sanderling/California/23-005401-001/2023|EPI_ISL_17964856|A_/_H5N1|Original||2.3.4.4b|14.02.2023|Killian_Mary_Lea||23-005401-001|06.07.2023|National_Veterinary_Services_Laboratories_-_USDA|National_Veterinary_Services_Laboratories_-_USDA|HA|4|HA_A/s">
<date value="2023.123287671233" direction="forwards" units="years"/>

</taxon>
<taxon id="A/sanderling/California/23-006644-001/2023|EPI_ISL_17964876|A_/_H5N1|Original||2.3.4.4b|14.02.2023|Killian_Mary_Lea||23-006644-001|06.07.2023|National_Veterinary_Services_Laboratories_-_USDA|National_Veterinary_Services_Laboratories_-_USDA|HA|4|HA_A/s">
<date value="2023.123287671233" direction="forwards" units="years"/>
</taxon>
<taxon id="A/sanderling/Oregon/23-000771-001/2023|EPI_ISL_19605627|A_/_H5N1|Original||2.3.4.4b|03.01.2023||||12.12.2024|USDA_APHIS_Veterinary_Services_Diagnostic_Virology_Laboratory_National_Veterinary_Services_Laboratories||HA|4|A/sanderling/Oregon/23-000771">
<date value="2023.0082191780823" direction="forwards" units="years"/>
</taxon>
<taxon id="A/sanderling/Oregon/23-008234-001/2023|EPI_ISL_17964904|A_/_H5N1|Original||2.3.4.4b|04.02.2023|Killian_Mary_Lea||23-008234-001|06.07.2023|National_Veterinary_Services_Laboratories_-_USDA|National_Veterinary_Services_Laboratories_-_USDA|HA|4|HA_A/sande">
<date value="2023.0958904109589" direction="forwards" units="years"/>
</taxon>
<taxon id="A/sanderling/Oregon/23-011964-001/2023|EPI_ISL_17964941|A_/_H5N1|Original||2.3.4.4b|13.04.2023|Killian_Mary_Lea||23-011964-001|06.07.2023|National_Veterinary_Services_Laboratories_-_USDA|National_Veterinary_Services_Laboratories_-_USDA|HA|4|HA_A/sande">
<date value="2023.282191780822" direction="forwards" units="years"/>
</taxon>
<taxon id="A/sanderling/Washington/23-004228-001/2023|EPI_ISL_19605618|A_/_H5N1|Original||2.3.4.4b|26.01.2023||||12.12.2024|USDA_APHIS_Veterinary_Services_Diagnostic_Virology_Laboratory_National_Veterinary_Services_Laboratories||HA|4|A/sanderling/Washington/2">
<date value="2023.0712328767124" direction="forwards" units="years"/>
</taxon>
<taxon id="A/sea_lion/Arica_y_Parinacota/240270-1/2023|EPI_ISL_17885976|A_/_H5N1|Original||2.3.4.4b|01.03.2023|GISAID_EpiFlu_Data_Curator|||19.07.2023|||HA|4|A/sea_lion/Arica_y_Parinacota/240270-1/2023_HA|EPI2609545|DNA_IN">
<date value="2023.0794520547945" direction="forwards" units="years"/>
</taxon>
<taxon id="A/sea_lion/Atacama/242444-1/2023|EPI_ISL_19391462|A_/_H5N1|Original||2.3.4.4b|13.03.2023||||09.09.2024|CEIRS_Data_Processing_and_Coordinating_Center_Center_for_Research_on_Influenza_Pathogenesis__CRIP_||HA|4|A/sea_lion/Atacama/242444-1/2023_HA|EPI3549">
<date value="2023.1123287671232" direction="forwards" units="years"/>
</taxon>
<taxon id="A/sea_lion/Biobio/SJCEIRR-2462961/2023|EPI_ISL_18690733|A_/_H5N1|Original||2.3.4.4b|26.03.2023||||26.12.2023|Emory_University__CEIRR_|Emory_University__CEIRR_|HA|4|A/sea_lion/Biobio/SJCEIRR-2462961/2023|EPI2868255|DNA_IN">
<date value="2023.1479452054793" direction="forwards" units="years"/>
</taxon>
<taxon id="A/sea_lion/Brazil/KU-3584/2023|EPI_ISL_18773644|A_/_H5N1|Original||2.3.4.4b|12.10.2023||||16.01.2024|Konkuk_University|Konkuk_University|HA|4|A/sea_lion/Brazil/KU-3584/2023_HA|EPI2919521|DNA_IN">
<date value="2023.7260273972602" direction="forwards" units="years"/>
</taxon>
<taxon id="A/sea_lion/Brazil/KU-3594/2023|EPI_ISL_18773660|A_/_H5N1|Original||2.3.4.4b|12.10.2023||||16.01.

2024|Konkuk_University|Konkuk_University|HA|4|A/sea_lion/Brazil/KU-3594/2023_HA|EPI2919538|DNA_IN">
<date value="2023.7260273972602" direction="forwards" units="years"/>
</taxon>
<taxon id="A/sea_lion/Los_Lagos/247932/2023|EPI_ISL_19404920|A_/_H5N1|Original||2.3.4.4b|09.04.2023||||10.09.2024|Emory_University__CEIRR_||HA|4|A/sea_lion/Los_Lagos/247932/2023_HA|EPI3550220|DNA_IN">
<date value="2023.2712328767122" direction="forwards" units="years"/>
</taxon>
<taxon id="A/sea_lion/Peru/AQP-SER00B/2023|EPI_ISL_18054508|A_/_H5N1|Original||2.3.4.4b|25.01.2023|GISAID_EpiFlu_Data_Curator|||01.08.2023|||HA|4|A/sea_lion/Peru/AQP-SER00B/2023_HA|EPI2664262|DNA_IN">
<date value="2023.0684931506848" direction="forwards" units="years"/>
</taxon>
<taxon id="A/sea_lion/Peru/AQP-SER00K/2023|EPI_ISL_18054509|A_/_H5N1|Original||2.3.4.4b|07.02.2023|GISAID_EpiFlu_Data_Curator|||01.08.2023|||HA|4|A/sea_lion/Peru/AQP-SER00K/2023_HA|EPI2664269|DNA_IN">
<date value="2023.1041095890412" direction="forwards" units="years"/>
</taxon>
<taxon id="A/sea_lion/Peru/AQP-SER00R/2023|EPI_ISL_18054510|A_/_H5N1|Original||2.3.4.4b|06.03.2023|GISAID_EpiFlu_Data_Curator|||01.08.2023|||HA|4|A/sea_lion/Peru/AQP-SER00R/2023_HA|EPI2664277|DNA_IN">
<date value="2023.0931506849315" direction="forwards" units="years"/>
</taxon>
<taxon id="A/sea_lion/Peru/TAC-INS-010/2023|EPI_ISL_17777531|A_/_H5N1|Original||2.3.4.4b|07.03.2023|Padilla_Rojas_Carlos_Patricio||1375|01.07.2023|Laboratorio_de_Virus_Respiratorios_Centro_Nacional_de_Salud_Publica|Instituto_Nacional_de_Salud_Peru|HA|4|Peru_INS">
<date value="2023.0958904109589" direction="forwards" units="years"/>
</taxon>
<taxon id="A/sea_lion/Peru/TAC-INS-011/2023|EPI_ISL_17777532|A_/_H5N1|Original||2.3.4.4b|07.03.2023|Padilla_Rojas_Carlos_Patricio||1375|01.07.2023|Laboratorio_de_Virus_Respiratorios_Centro_Nacional_de_Salud_Publica|Instituto_Nacional_de_Salud_Peru|HA|4|Peru_INS">
<date value="2023.0958904109589" direction="forwards" units="years"/>
</taxon>
<taxon id="A/sea_lion/Tarapaca/240524-2/2023|EPI_ISL_17885975|A_/_H5N1|Original||2.3.4.4b|07.03.2023|GISAID_EpiFlu_Data_Curator|||19.07.2023|||HA|4|A/sea_lion/Tarapaca/240524-2/2023_HA|EPI2609536|DNA_IN">
<date value="2023.0958904109589" direction="forwards" units="years"/>
</taxon>
<taxon id="A/sea_lion/Tarapaca/245719-10/2023|EPI_ISL_19410279|A_/_H5N1|Original||2.3.4.4b|30.03.2023||||12.09.2024|Emory_University__CEIRR_||HA|4|A/sea_lion/Tarapaca/245719-10/2023_HA|EPI3555288|DNA_IN">
<date value="2023.158904109589" direction="forwards" units="years"/>
</taxon>
<taxon id="A/sea_lion/Valparaiso/SJCEIRR-2431361/2023|EPI_ISL_18760069|A_/_H5N1|Original||2.3.4.4b|19.03.2023||||14.01.2024|Emory_University__CEIRR_|Emory_University__CEIRR_|HA|4|A/sea_lion/Valparaiso/SJCEIRR-2431361/2023|EPI2913244|DNA_IN">
<date value="2023.1287671232876" direction="forwards" units="years"/>
</taxon>
<taxon id="A/seabird/Uruguay/P16_14926/2023|EPI_ISL_19070494|A_/_H5N1|Original||2.3.4.4b|14.09.2023||||24.04.2024|Universidad_de_la_Republica_Facultad

_de_Ciencias_Genetica_Evolutiva/Division_de_Laboratorios_Veterinarios_Plata
forma_Genomica||HA|4|A/seabird/Urug">
<date value="2023.704109589041" direction="forwards" units="years"/>
</taxon>
<taxon id="A/seabird/Uruguay/P23_41023/2023|EPI_ISL_19070495|A_/_H5N1|Origi
nal||2.3.4.4b|04.10.2023||||24.04.2024|Universidad_de_la_Republica_Facultad
_de_Ciencias_Genetica_Evolutiva/Division_de_Laboratorios_Veterinarios_Plata
forma_Genomica||HA|4|A/seabird/Urug">
<date value="2023.704109589041" direction="forwards" units="years"/>
</taxon>
<taxon id="A/seabird/Uruguay/P24_41023/2023|EPI_ISL_19070496|A_/_H5N1|Origi
nal||2.3.4.4b|04.10.2023||||24.04.2024|Universidad_de_la_Republica_Facultad
_de_Ciencias_Genetica_Evolutiva/Division_de_Laboratorios_Veterinarios_Plata
forma_Genomica||HA|4|A/seabird/Urug">
<date value="2023.704109589041" direction="forwards" units="years"/>
</taxon>
<taxon id="A/seabird/Uruguay/P25_41023/2023|EPI_ISL_19070497|A_/_H5N1|Origi
nal||2.3.4.4b|04.10.2023||||24.04.2024|Universidad_de_la_Republica_Facultad
_de_Ciencias_Genetica_Evolutiva/Division_de_Laboratorios_Veterinarios_Plata
forma_Genomica||HA|4|A/seabird/Urug">
<date value="2023.704109589041" direction="forwards" units="years"/>
</taxon>
<taxon id="A/snow_goose/Alaska/22-041764-
001/2022|EPI_ISL_19605880|A_/_H5N1|Original||2.3.4.4b|20.12.2022||||12.12.2
024|USDA_APHIS_Veterinary_Services_Diagnostic_Virology_Laboratory_National_
Veterinary_Services_Laboratories||HA|4|A/snow_goose/Alaska/22-041764">
<date value="2022.9698630136986" direction="forwards" units="years"/>
</taxon>
<taxon id="A/snow_goose/Arkansas/23-001513-
001/2023|EPI_ISL_19605884|A_/_H5N1|Original||2.3.4.4b|06.01.2023||||12.12.2
024|USDA_APHIS_Veterinary_Services_Diagnostic_Virology_Laboratory_National_
Veterinary_Services_Laboratories||HA|4|A/snow_goose/Arkansas/23-00">
<date value="2023.0164383561644" direction="forwards" units="years"/>
</taxon>
<taxon id="A/snow_goose/Arkansas/23-001514-
001/2023|EPI_ISL_19605885|A_/_H5N1|Original||2.3.4.4b|06.01.2023||||12.12.2
024|USDA_APHIS_Veterinary_Services_Diagnostic_Virology_Laboratory_National_
Veterinary_Services_Laboratories||HA|4|A/snow_goose/Arkansas/23-00">
<date value="2023.0164383561644" direction="forwards" units="years"/>
</taxon>
<taxon id="A/snow_goose/BC/AIVPHL-
337/2022|EPI_ISL_17051466|A_/_H5N1|Original||2.3.4.4b|19.12.2022|Russell_Sh
annon_Laurel|||28.02.2023|B.C._Centre_for_Disease_Control|British_Columbia_
Centre_for_Disease_Control|HA|4|A/snow_goose/BC/AIVPHL-
337/2022_HA|EPI2429175|">
<date value="2022.9671232876713" direction="forwards" units="years"/>
</taxon>
<taxon id="A/snow_goose/BC/AIVPHL-
340/2022|EPI_ISL_17051461|A_/_H5N1|Original||2.3.4.4b|15.12.2022|Russell_Sh
annon_Laurel|||28.02.2023|B.C._Centre_for_Disease_Control|British_Columbia_
Centre_for_Disease_Control|HA|4|A/snow_goose/BC/AIVPHL-
340/2022_HA|EPI2429142|">
<date value="2022.9561643835616" direction="forwards" units="years"/>
</taxon>
<taxon id="A/snow_goose/California/22-041398-
001/2022|EPI_ISL_19605887|A_/_H5N1|Original||2.3.4.4b|01.12.2022||||12.12.2
024|USDA_APHIS_Veterinary_Services_Diagnostic_Virology_Laboratory_National_
Veterinary_Services_Laboratories||HA|4|A/snow_goose/California/2">
<date value="2022.9178082191781" direction="forwards" units="years"/>
</taxon>

<taxon id="A/snow_goose/California/22-041400-002/2022|EPI_ISL_19605888|A_/_H5N1|Original||2.3.4.4b|01.12.2022||||12.12.2024|USDA_APHIS_Veterinary_Services_Diagnostic_Virology_Laboratory_National_Veterinary_Services_Laboratories||HA|4|A/snow_goose/California/2">
<date value="2022.9178082191781" direction="forwards" units="years"/>
</taxon>
<taxon id="A/snow_goose/California/22-041403-001/2022|EPI_ISL_19605889|A_/_H5N1|Original||2.3.4.4b|01.12.2022||||12.12.2024|USDA_APHIS_Veterinary_Services_Diagnostic_Virology_Laboratory_National_Veterinary_Services_Laboratories||HA|4|A/snow_goose/California/2">
<date value="2022.9178082191781" direction="forwards" units="years"/>
</taxon>
<taxon id="A/snow_goose/California/22-041403-002/2022|EPI_ISL_19605890|A_/_H5N1|Original||2.3.4.4b|01.12.2022||||12.12.2024|USDA_APHIS_Veterinary_Services_Diagnostic_Virology_Laboratory_National_Veterinary_Services_Laboratories||HA|4|A/snow_goose/California/2">
<date value="2022.9178082191781" direction="forwards" units="years"/>
</taxon>
<taxon id="A/snow_goose/California/22-041403-003/2022|EPI_ISL_19605891|A_/_H5N1|Original||2.3.4.4b|01.12.2022||||12.12.2024|USDA_APHIS_Veterinary_Services_Diagnostic_Virology_Laboratory_National_Veterinary_Services_Laboratories||HA|4|A/snow_goose/California/2">
<date value="2022.9178082191781" direction="forwards" units="years"/>
</taxon>
<taxon id="A/snow_goose/California/22-041408-001/2022|EPI_ISL_19605893|A_/_H5N1|Original||2.3.4.4b|01.12.2022||||12.12.2024|USDA_APHIS_Veterinary_Services_Diagnostic_Virology_Laboratory_National_Veterinary_Services_Laboratories||HA|4|A/snow_goose/California/2">
<date value="2022.9178082191781" direction="forwards" units="years"/>
</taxon>
<taxon id="A/snow_goose/California/22-041409-001/2022|EPI_ISL_19605894|A_/_H5N1|Original||2.3.4.4b|01.12.2022||||12.12.2024|USDA_APHIS_Veterinary_Services_Diagnostic_Virology_Laboratory_National_Veterinary_Services_Laboratories||HA|4|A/snow_goose/California/2">
<date value="2022.9178082191781" direction="forwards" units="years"/>
</taxon>
<taxon id="A/snow_goose/California/22-042007-002/2022|EPI_ISL_19605896|A_/_H5N1|Original||2.3.4.4b|14.12.2022||||12.12.2024|USDA_APHIS_Veterinary_Services_Diagnostic_Virology_Laboratory_National_Veterinary_Services_Laboratories||HA|4|A/snow_goose/California/2">
<date value="2022.9534246575342" direction="forwards" units="years"/>
</taxon>
<taxon id="A/snow_goose/California/22-042007-003/2022|EPI_ISL_19605897|A_/_H5N1|Original||2.3.4.4b|14.12.2022||||12.12.2024|USDA_APHIS_Veterinary_Services_Diagnostic_Virology_Laboratory_National_Veterinary_Services_Laboratories||HA|4|A/snow_goose/California/2">
<date value="2022.9534246575342" direction="forwards" units="years"/>
</taxon>
<taxon id="A/snow_goose/California/22-042007-004/2022|EPI_ISL_19605898|A_/_H5N1|Original||2.3.4.4b|14.12.2022||||12.12.2024|USDA_APHIS_Veterinary_Services_Diagnostic_Virology_Laboratory_National_Veterinary_Services_Laboratories||HA|4|A/snow_goose/California/2">
<date value="2022.9534246575342" direction="forwards" units="years"/>
</taxon>
<taxon id="A/snow_goose/California/23-000936-002/2022|EPI_ISL_19605899|A_/_H5N1|Original||2.3.4.4b|16.12.2022||||12.12.2024|USDA_APHIS_Veterinary_Services_Diagnostic_Virology_Laboratory_National_Veterinary_Services_Laboratories||HA|4|A/snow_goose/California/2">
<date value="2022.958904109589" direction="forwards" units="years"/>
</taxon>

<taxon id="A/snow_goose/California/23-000950-
001/2022|EPI_ISL_19605877|A_/_H5N1|Original||2.3.4.4b|19.12.2022||||12.12.2
024|USDA_APHIS_Veterinary_Services_Diagnostic_Virology_Laboratory_National_
Veterinary_Services_Laboratories||HA|4|A/snow_goose/California/2">
<date value="2022.9671232876713" direction="forwards" units="years"/>
</taxon>
<taxon id="A/snow_goose/California/23-002594-
003/2023|EPI_ISL_19605875|A_/_H5N1|Original||2.3.4.4b|10.01.2023||||12.12.2
024|USDA_APHIS_Veterinary_Services_Diagnostic_Virology_Laboratory_National_
Veterinary_Services_Laboratories||HA|4|A/snow_goose/California/2">
<date value="2023.027397260274" direction="forwards" units="years"/>
</taxon>
<taxon id="A/snow_goose/California/23-004358-
002/2023|EPI_ISL_19605862|A_/_H5N1|Original||2.3.4.4b|25.01.2023||||12.12.2
024|USDA_APHIS_Veterinary_Services_Diagnostic_Virology_Laboratory_National_
Veterinary_Services_Laboratories||HA|4|A/snow_goose/California/2">
<date value="2023.0684931506848" direction="forwards" units="years"/>
</taxon>
<taxon id="A/snow_goose/Kansas/22-041130-
003/2022|EPI_ISL_19605948|A_/_H5N1|Original||2.3.4.4b|06.12.2022||||12.12.2
024|USDA_APHIS_Veterinary_Services_Diagnostic_Virology_Laboratory_National_
Veterinary_Services_Laboratories||HA|4|A/snow_goose/Kansas/22-041130">
<date value="2022.9315068493152" direction="forwards" units="years"/>
</taxon>
<taxon id="A/snow_goose/Louisiana/23-000409-
016/2022|EPI_ISL_19608599|A_/_H5N1|Original||2.3.4.4b|02.12.2022||||12.12.2
024|USDA_APHIS_Veterinary_Services_Diagnostic_Virology_Laboratory_National_
Veterinary_Services_Laboratories||HA|4|A/snow_goose/Louisiana/23-">
<date value="2022.9205479452055" direction="forwards" units="years"/>
</taxon>
<taxon id="A/snow_goose/Louisiana/23-000409-
018/2022|EPI_ISL_19608600|A_/_H5N1|Original||2.3.4.4b|02.12.2022||||12.12.2
024|USDA_APHIS_Veterinary_Services_Diagnostic_Virology_Laboratory_National_
Veterinary_Services_Laboratories||HA|4|A/snow_goose/Louisiana/23-">
<date value="2022.9205479452055" direction="forwards" units="years"/>
</taxon>
<taxon id="A/snow_goose/Missouri/22-040427-
002/2022|EPI_ISL_19605947|A_/_H5N1|Original||2.3.4.4b|01.12.2022||||12.12.2
024|USDA_APHIS_Veterinary_Services_Diagnostic_Virology_Laboratory_National_
Veterinary_Services_Laboratories||HA|4|A/snow_goose/Missouri/22-04">
<date value="2022.9178082191781" direction="forwards" units="years"/>
</taxon>
<taxon id="A/snow_goose/Missouri/22-040786-
001/2022|EPI_ISL_19605928|A_/_H5N1|Original||2.3.4.4b|08.12.2022||||12.12.2
024|USDA_APHIS_Veterinary_Services_Diagnostic_Virology_Laboratory_National_
Veterinary_Services_Laboratories||HA|4|A/snow_goose/Missouri/22-04">
<date value="2022.9369863013699" direction="forwards" units="years"/>
</taxon>
<taxon id="A/snow_goose/Washington/22-041983-
001/2022|EPI_ISL_19605053|A_/_H5N1|Original||2.3.4.4b|16.12.2022||||12.12.2
024|USDA_APHIS_Veterinary_Services_Diagnostic_Virology_Laboratory_National_
Veterinary_Services_Laboratories||HA|4|A/snow_goose/Washington/2">
<date value="2022.958904109589" direction="forwards" units="years"/>
</taxon>
<taxon id="A/southern_elephant_seal/Argentina/CH-
PM053/2023|EPI_ISL_19466252|A_/_H5N1|Original||2.3.4.4b|01.11.2023||||08.10
.2024|Instituto_Nacional_de_Tecnologia_Agropecuaria_Instituto_de_Virologia_
e_Innovaciones_Tecnologicas||HA|4|A/southern_elephant_seal/Arge">
<date value="2023.835616438356" direction="forwards" units="years"/>
</taxon>

<taxon id="A/striped_skunk/Kansas/W23-094/2023|EPI_ISL_17424646|A_/_H5N1|||2.3.4.4b|27.01.2023||||08.04.2023|St._Jude_Center_of_Excellence_for_Influenza_Research_and_Response_-_CEIRR_Influenza_Data_Processing_and_Communication_Center|St._Jude_Center_of_Excellence">
<date value="2023.0739726027398" direction="forwards" units="years"/>
</taxon>
<taxon id="A/swainsons_hawk/Colorado/23-004704-001/2023|EPI_ISL_19605066|A_/_H5N1|Original||2.3.4.4b|30.01.2023||||12.12.2024|USDA_APHIS_Veterinary_Services_Diagnostic_Virology_Laboratory_National_Veterinary_Services_Laboratories||HA|4|A/swainsons_hawk/Colora">
<date value="2023.0821917808219" direction="forwards" units="years"/>
</taxon>
<taxon id="A/swan/Nebraska/22-041037-001/2022|EPI_ISL_19605145|A_/_H5N1|Original||2.3.4.4b|15.12.2022||||12.12.2024|USDA_APHIS_Veterinary_Services_Diagnostic_Virology_Laboratory_National_Veterinary_Services_Laboratories||HA|4|A/swan/Nebraska/22-041037-001/202">
<date value="2022.9561643835616" direction="forwards" units="years"/>
</taxon>
<taxon id="A/swan/Nebraska/22-041037-002/2022|EPI_ISL_19605146|A_/_H5N1|Original||2.3.4.4b|15.12.2022||||12.12.2024|USDA_APHIS_Veterinary_Services_Diagnostic_Virology_Laboratory_National_Veterinary_Services_Laboratories||HA|4|A/swan/Nebraska/22-041037-002/202">
<date value="2022.9561643835616" direction="forwards" units="years"/>
</taxon>
<taxon id="A/tern/Espirito_Santo/MAPA-1339N2/2023|EPI_ISL_18755338|A_/_H5N1|Original||2.3.4.4b|15.05.2023||||12.01.2024|Ministerio_da_Agricultura_e_Pecuaria|Ministerio_da_Agricultura_e_Pecuaria|HA|4|A/tern/Espirito_Santo/MAPA-1339N2/2023_HA|EPI2908529|DNA_IN">
<date value="2023.3698630136987" direction="forwards" units="years"/>
</taxon>
<taxon id="A/trumpeter_swan/BC/AIVPHL-320/2022|EPI_ISL_17051458|A_/_H5N1|Original||2.3.4.4b|12.12.2022|Russell_Shannon_Laurel|||28.02.2023|B.C._Centre_for_Disease_Control|British_Columbia_Centre_for_Disease_Control|HA|4|A/trumpeter_swan/BC/AIVPHL-320/2022_HA|EPI">
<date value="2022.9479452054795" direction="forwards" units="years"/>
</taxon>
<taxon id="A/trumpeter_swan/Iowa/23-002039-001/2023|EPI_ISL_19605154|A_/_H5N1|Original||2.3.4.4b|12.01.2023||||12.12.2024|USDA_APHIS_Veterinary_Services_Diagnostic_Virology_Laboratory_National_Veterinary_Services_Laboratories||HA|4|A/trumpeter_swan/Iowa/23-00">
<date value="2023.0328767123287" direction="forwards" units="years"/>
</taxon>
<taxon id="A/trumpeter_swan/Iowa/23-002039-002/2023|EPI_ISL_19605155|A_/_H5N1|Original||2.3.4.4b|12.01.2023||||12.12.2024|USDA_APHIS_Veterinary_Services_Diagnostic_Virology_Laboratory_National_Veterinary_Services_Laboratories||HA|4|A/trumpeter_swan/Iowa/23-00">
<date value="2023.0328767123287" direction="forwards" units="years"/>
</taxon>
<taxon id="A/trumpeter_swan/Iowa/23-002662-002/2023|EPI_ISL_19605156|A_/_H5N1|Original||2.3.4.4b|21.01.2023||||12.12.2024|USDA_APHIS_Veterinary_Services_Diagnostic_Virology_Laboratory_National_Veterinary_Services_Laboratories||HA|4|A/trumpeter_swan/Iowa/23-00">
<date value="2023.0575342465754" direction="forwards" units="years"/>
</taxon>
<taxon id="A/trumpeter_swan/Missouri/22-040427-001/2022|EPI_ISL_19605137|A_/_H5N1|Original||2.3.4.4b|01.12.2022||||12.12.2024|USDA_APHIS_Veterinary_Services_Diagnostic_Virology_Laboratory_National_Veterinary_Services_Laboratories||HA|4|A/trumpeter_swan/Missou">
<date value="2022.9178082191781" direction="forwards" units="years"/>

</taxon>
<taxon id="A/turkey/Araucania/241892-
3/2023|EPI_ISL_17885966|A_/_H5N1|Original||2.3.4.4b|14.03.2023|GISAID_EpiFl
u_Data_Curator|||24.07.2023|||HA|4|A/turkey/Araucania/241892-
3/2023_HA|EPI2609482|DNA_IN">
<date value="2023.1150684931506" direction="forwards" units="years"/>
</taxon>
<taxon id="A/turkey/Nuble/240489-
1/2023|EPI_ISL_17885964|A_/_H5N1|Original||2.3.4.4b|07.03.2023|GISAID_EpiFl
u_Data_Curator|||19.07.2023|||HA|4|A/turkey/Nuble/240489-
1/2023_HA|EPI2609474|DNA_IN">
<date value="2023.0958904109589" direction="forwards" units="years"/>
</taxon>
<taxon id="A/turkey/Nuble/241568-
1/2023|EPI_ISL_17885962|A_/_H5N1|Original||2.3.4.4b|10.03.2023|GISAID_EpiFl
u_Data_Curator|||19.07.2023|||HA|4|A/turkey/Nuble/241568-
1/2023_HA|EPI2609462|DNA_IN">
<date value="2023.1041095890412" direction="forwards" units="years"/>
</taxon>
<taxon id="A/turkey/Valparaiso/245562-
1/2023|EPI_ISL_18760063|A_/_H5N1|Original||2.3.4.4b|29.03.2023||||14.01.202
4|Emory_University__CEIRR_|Emory_University__CEIRR_|HA|4|A/turkey/Valparais
o/245562-1/2023_HA|EPI2913205|DNA_IN">
<date value="2023.1561643835616" direction="forwards" units="years"/>
</taxon>
<taxon id="A/turkey/Valparaiso/245886-
1/2023|EPI_ISL_19410284|A_/_H5N1|Original||2.3.4.4b|30.03.2023||||13.09.202
4|Emory_University__CEIRR_||HA|4|A/turkey/Valparaiso/245886-
1/2023_HA|EPI3555321|DNA_IN">
<date value="2023.158904109589" direction="forwards" units="years"/>
</taxon>
<taxon id="A/turkey/Valparaiso/245886-
3/2023|EPI_ISL_19410285|A_/_H5N1|Original||2.3.4.4b|30.03.2023||||13.09.202
4|Emory_University__CEIRR_||HA|4|A/turkey/Valparaiso/245886-
3/2023_HA|EPI3555328|DNA_IN">
<date value="2023.158904109589" direction="forwards" units="years"/>
</taxon>
<taxon id="A/turkey/Valparaiso/245886-
5/2023|EPI_ISL_19410286|A_/_H5N1|Original||2.3.4.4b|30.03.2023||||13.09.202
4|Emory_University__CEIRR_||HA|4|A/turkey/Valparaiso/245886-
5/2023_HA|EPI3555335|DNA_IN">
<date value="2023.158904109589" direction="forwards" units="years"/>
</taxon>
<taxon id="A/turkey/Valparaiso/248976-
12/2023|EPI_ISL_19404902|A_/_H5N1|Original||2.3.4.4b|13.04.2023||||13.09.20
24|Emory_University__CEIRR_||HA|4|A/turkey/Valparaiso/248976-
12/2023_HA|EPI3550180|DNA_IN">
<date value="2023.282191780822" direction="forwards" units="years"/>
</taxon>
<taxon id="A/turkey/Valparaiso/248976-
13/2023|EPI_ISL_19404939|A_/_H5N1|Original||2.3.4.4b|13.04.2023||||13.09.20
24|Emory_University__CEIRR_||HA|4|A/turkey/Valparaiso/248976-
13/2023_HA|EPI3550235|DNA_IN">
<date value="2023.282191780822" direction="forwards" units="years"/>
</taxon>
<taxon id="A/turkey_vulture/California/22-041407-
001/2022|EPI_ISL_19605121|A_/_H5N1|Original||2.3.4.4b|01.12.2022||||12.12.2
024|USDA_APHIS_Veterinary_Services_Diagnostic_Virology_Laboratory_National_
Veterinary_Services_Laboratories||HA|4|A/turkey_vulture/Cali">
<date value="2022.9178082191781" direction="forwards" units="years"/>
</taxon>

<taxon id="A/turkey_vulture/California/22-041409-003/2022|EPI_ISL_19605134|A_/_H5N1|Original||2.3.4.4b|01.12.2022||||12.12.2024|USDA_APHIS_Veterinary_Services_Diagnostic_Virology_Laboratory_National_Veterinary_Services_Laboratories||HA|4|A/turkey_vulture/Cali">
<date value="2022.9178082191781" direction="forwards" units="years"/>
</taxon>
<taxon id="A/turkey_vulture/California/23-000283-001/2022|EPI_ISL_19605126|A_/_H5N1|Original||2.3.4.4b|14.12.2022||||12.12.2024|USDA_APHIS_Veterinary_Services_Diagnostic_Virology_Laboratory_National_Veterinary_Services_Laboratories||HA|4|A/turkey_vulture/Cali">
<date value="2022.9534246575342" direction="forwards" units="years"/>
</taxon>
<taxon id="A/turkey_vulture/California/23-002030-001/2022|EPI_ISL_19605130|A_/_H5N1|Original||2.3.4.4b|29.12.2022||||12.12.2024|USDA_APHIS_Veterinary_Services_Diagnostic_Virology_Laboratory_National_Veterinary_Services_Laboratories||HA|4|A/turkey_vulture/Cali">
<date value="2022.9945205479453" direction="forwards" units="years"/>
</taxon>
<taxon id="A/turkey_vulture/California/23-002601-001/2023|EPI_ISL_19605131|A_/_H5N1|Original||2.3.4.4b|11.01.2023||||12.12.2024|USDA_APHIS_Veterinary_Services_Diagnostic_Virology_Laboratory_National_Veterinary_Services_Laboratories||HA|4|A/turkey_vulture/Cali">
<date value="2023.0301369863014" direction="forwards" units="years"/>
</taxon>
<taxon id="A/turkey_vulture/California/23-004361-001/2023|EPI_ISL_19605132|A_/_H5N1|Original||2.3.4.4b|25.01.2023||||12.12.2024|USDA_APHIS_Veterinary_Services_Diagnostic_Virology_Laboratory_National_Veterinary_Services_Laboratories||HA|4|A/turkey_vulture/Cali">
<date value="2023.0684931506848" direction="forwards" units="years"/>
</taxon>
<taxon id="A/turkey_vulture/California/23-016941-001/2023|EPI_ISL_17964980|A_/_H5N1|Original||2.3.4.4b|23.05.2023|Killian_Mary_Lea||23-016941-001|06.07.2023|National_Veterinary_Services_Laboratories_-_USDA|National_Veterinary_Services_Laboratories_-_USDA|HA|4|HA">
<date value="2023.3917808219178" direction="forwards" units="years"/>
</taxon>
<taxon id="A/turkey_vulture/Colorado/23-011447-001/2023|EPI_ISL_17964934|A_/_H5N1|Original||2.3.4.4b|10.04.2023|Killian_Mary_Lea||23-011447-001|06.07.2023|National_Veterinary_Services_Laboratories_-_USDA|National_Veterinary_Services_Laboratories_-_USDA|HA|4|HA_A">
<date value="2023.2739726027398" direction="forwards" units="years"/>
</taxon>
<taxon id="A/turkey_vulture/Utah/23-012747-001/2023|EPI_ISL_17964947|A_/_H5N1|Original||2.3.4.4b|19.04.2023|Killian_Mary_Lea||23-012747-001|06.07.2023|National_Veterinary_Services_Laboratories_-_USDA|National_Veterinary_Services_Laboratories_-_USDA|HA|4|HA_A/tur">
<date value="2023.2986301369863" direction="forwards" units="years"/>
</taxon>
<taxon id="A/turkey_vulture/Utah/23-014335-001/2023|EPI_ISL_17964961|A_/_H5N1|Original||2.3.4.4b|28.04.2023|Killian_Mary_Lea||23-014335-001|06.07.2023|National_Veterinary_Services_Laboratories_-_USDA|National_Veterinary_Services_Laboratories_-_USDA|HA|4|HA_A/tur">
<date value="2023.323287671233" direction="forwards" units="years"/>
</taxon>
<taxon id="A/turkey_vulture/Wyoming/23-012565-001/2023|EPI_ISL_17964946|A_/_H5N1|Original||2.3.4.4b|19.04.2023|Killian_Mary_Lea||23-012565-

001|06.07.2023|National_Veterinary_Services_Laboratories_-
_USDA|National_Veterinary_Services_Laboratories_-_USDA|HA|4|HA_A/">
<date value="2023.2986301369863" direction="forwards" units="years"/>
</taxon>
<taxon id="A/turkey_vulture/Wyoming/23-013715-
001/2023|EPI_ISL_17964953|A_/_H5N1|Original||2.3.4.4b|19.04.2023|Killian_Ma
ry_Lea||23-013715-
001|06.07.2023|National_Veterinary_Services_Laboratories_-
_USDA|National_Veterinary_Services_Laboratories_-_USDA|HA|4|HA_A/">
<date value="2023.2986301369863" direction="forwards" units="years"/>
</taxon>
<taxon id="A/western_grebe/California/23-000276-
001/2022|EPI_ISL_19605204|A_/_H5N1|Original||2.3.4.4b|15.12.2022||||12.12.2
024|USDA_APHIS_Veterinary_Services_Diagnostic_Virology_Laboratory_National_
Veterinary_Services_Laboratories||HA|4|A/western_grebe/Califo">
<date value="2022.9561643835616" direction="forwards" units="years"/>
</taxon>
<taxon id="A/whimbrel/Coquimbo/239964/2023|EPI_ISL_17885960|A_/_H5N1|Origin
al||2.3.4.4b|05.03.2023|GISAID_EpiFlu_Data_Curator|||19.07.2023|||HA|4|A/wh
imbrel/Coquimbo/239964/2023_HA|EPI2609454|DNA_IN">
<date value="2023.0904109589042" direction="forwards" units="years"/>
</taxon>
<taxon id="A/wildbird-Fregata-magnificens/Ecuador/IC03-
4587/2023|EPI_ISL_17973443|A_/_H5N1|Original||2.3.4.4b|11.01.2023|Carrazco_
Andres|IC03|4587|01.11.2023|Ministerio_del_Ambiente_Agua_y_Transicion_Ecolo
gica__MAATE_|Instituto_Nacional_de_Investigacion_en_Salu">
<date value="2023.0301369863014" direction="forwards" units="years"/>
</taxon>
<taxon id="A/wildbird-Fregata-magnificens/Ecuador/IC06-
4590/2023|EPI_ISL_17973458|A_/_H5N1|Original||2.3.4.4b|11.01.2023|Carrazco_
Andres|IC06|4590|16.10.2023|Ministerio_del_Ambiente_Agua_y_Transicion_Ecolo
gica__MAATE_|Instituto_Nacional_de_Investigacion_en_Salu">
<date value="2023.0301369863014" direction="forwards" units="years"/>
</taxon>
<taxon id="A/wood_duck/Colorado/23-000400-
001/2022|EPI_ISL_19605216|A_/_H5N1|Original||2.3.4.4b|29.12.2022||||12.12.2
024|USDA_APHIS_Veterinary_Services_Diagnostic_Virology_Laboratory_National_
Veterinary_Services_Laboratories||HA|4|A/wood_duck/Colorado/23-0004">
<date value="2022.9945205479453" direction="forwards" units="years"/>
</taxon>
<taxon id="OQ547327.1|InfluenzaAvirus_A/Nannopterumbrasilianus/Peru/AISA045
1/2022_H5N1__segment4hemagglutinin_HA_genecompletecds|2022-11-
22|4|H5N1|Peru|Phalacrocoraxbrasilianus|Peru|Alphainfluenzavirus|Alphainflu
enzavirusinfluenz">
<date value="2022.8931506849315" direction="forwards" units="years"/>
</taxon>
<taxon id="OQ547335.1|InfluenzaAvirus_A/Pelecanusthagus/Peru/AIS0541/2022_H
5N1__segment4hemagglutinin_HA_genecompletecds|2022-11-
16|4|H5N1|Peru|Pelecanusthagus|Peru|Alphainfluenzavirus|Alphainfluenzavirus
influenz">
<date value="2022.876712328767" direction="forwards" units="years"/>
</taxon>
<taxon id="OQ547343.1|InfluenzaAvirus_A/Gallusgallus/Peru/AIS0539/2022_H5N1
__segment4hemagglutinin_HA_genecompletecds|2022-11-
18|4|H5N1|Peru|Gallusgallus|Peru|Alphainfluenzavirus|Alphainfluenzavirusinf
luenz">
<date value="2022.8821917808218" direction="forwards" units="years"/>
</taxon>
<taxon id="OQ547351.1|InfluenzaAvirus_A/Gallusgallus/Peru/AIS0540/2022_H5N1
__segment4hemagglutinin_HA_genecompletecds|2022-11-

28|4|H5N1|Peru|Gallusgallus|Peru|Alphainfluenzavirus|Alphainfluenzavirusinf
luenz">
<date value="2022.9095890410958" direction="forwards" units="years"/>
</taxon>
<taxon id="OQ547423.1|InfluenzaAvirus_A/Nannopterumbrasilianus/Peru/AISA044
6/2022_H5N1__segment4hemagglutinin_HA_genecompletecds|2022-11-
22|4|H5N1|Peru|Phalacrocoraxbrasilianus|Peru|Alphainfluenzavirus|Alphainflu
enzavirusinfluenz">
<date value="2022.8931506849315" direction="forwards" units="years"/>
</taxon>
<taxon id="OQ547439.1|InfluenzaAvirus_A/Pelecanusthagus/Peru/AIS0538/2022_H
5N1__segment4hemagglutinin_HA_genecompletecds|2022-11-
10|4|H5N1|Peru|Pelecanusthagus|Peru|Alphainfluenzavirus|Alphainfluenzavirus
influenz">
<date value="2022.8602739726027" direction="forwards" units="years"/>
</taxon>
<taxon id="OQ547447.1|InfluenzaAvirus_A/Pelecanusthagus/Peru/AISA0464/2022_
H5N1__segment4hemagglutinin_HA_genecompletecds|2022-11-
22|4|H5N1|Peru|Pelecanusthagus|Peru|Alphainfluenzavirus|Alphainfluenzavirus
influenz">
<date value="2022.8931506849315" direction="forwards" units="years"/>
</taxon>
<taxon id="OQ550426.1|InfluenzaAvirus_A/Guanaycormorant/Peru/PIU-
SER024/2022_H5N1__segment4hemagglutinin_HA_genecompletecds|2022-11-
24|4|H5N1|Peru||Peru|Alphainfluenzavirus|Alphainfluenzavirusinfluenz">
<date value="2022.8986301369864" direction="forwards" units="years"/>
</taxon>
<taxon id="OQ550434.1|InfluenzaAvirus_A/pelican/Peru/PIU-
SER016/2022_H5N1__segment4hemagglutinin_HA_genecompletecds|2022-11-
24|4|H5N1|Peru|Pelecanidae|Peru|Alphainfluenzavirus|Alphainfluenzavirusinfl
uenz">
<date value="2022.8986301369864" direction="forwards" units="years"/>
</taxon>
<taxon id="OQ550442.1|InfluenzaAvirus_A/dolphin/Peru/PIU-
SER002/2022_H5N1__segment4hemagglutinin_HA_genecompletecds|2022-11-
22|4|H5N1|Peru|Delphinusdelphis|Peru|Alphainfluenzavirus|Alphainfluenzaviru
sinfluenz">
<date value="2022.8931506849315" direction="forwards" units="years"/>
</taxon>
<taxon id="OQ550450.1|InfluenzaAvirus_A/pelican/Peru/PIU-
SER019/2022_H5N1__segment4hemagglutinin_HA_genecompletecds|2022-11-
24|4|H5N1|Peru|Pelecanidae|Peru|Alphainfluenzavirus|Alphainfluenzavirusinfl
uenz">
<date value="2022.8986301369864" direction="forwards" units="years"/>
</taxon>
<taxon id="OQ550458.1|InfluenzaAvirus_A/pelican/Peru/PIU-
SER028/2022_H5N1__segment4hemagglutinin_HA_genecompletecds|2022-11-
24|4|H5N1|Peru|Pelecanidae|Peru|Alphainfluenzavirus|Alphainfluenzavirusinfl
uenz">
<date value="2022.8986301369864" direction="forwards" units="years"/>
</taxon>
<taxon id="OQ550466.1|InfluenzaAvirus_A/Sanderling/Peru/PIU-
SER005/2022_H5N1__segment4hemagglutinin_HA_genecompletecds|2022-11-
22|4|H5N1|Peru|Calidrisalba|Peru|Alphainfluenzavirus|Alphainfluenzavirusinf
luenz">
<date value="2022.8931506849315" direction="forwards" units="years"/>
</taxon>
<taxon id="OQ683458.1|InfluenzaAvirus_A/chicken/Colombia/Bolivar/3500/2022_
H5N1__segment4hemagglutinin_HA_genecompletecds|2022-11-
08|4|H5N1|Colombia|Gallusgallus|Colombia|Alphainfluenzavirus|Alphainfluenza
virusinfluenz">

<date value="2022.854794520548" direction="forwards" units="years"/>
</taxon>
<taxon id="OQ683482.1|InfluenzaAvirus_A/chicken/Colombia/Cordoba/3499/2022_H5N1__segment4hemagglutinin_HA_genecompletecds|2022-11-26|4|H5N1|Colombia|Gallusgallus|Colombia|Alphainfluenzavirus|Alphainfluenzavirusinfluenz">
<date value="2022.9041095890411" direction="forwards" units="years"/>
</taxon>
<taxon id="OQ683490.1|InfluenzaAvirus_A/chicken/Colombia/Magdalena/3503/2022_H5N1__segment4hemagglutinin_HA_genecompletecds|2022-11-18|4|H5N1|Colombia|Gallusgallus|Colombia|Alphainfluenzavirus|Alphainfluenzavirusinfluenz">
<date value="2022.8821917808218" direction="forwards" units="years"/>
</taxon>
<taxon id="OQ732972.1|InfluenzaAvirus_A/blue-wingedteal/Texas/UGAI22-2966/2022_H5N1__segment4hemagglutinin_HA_genecompletecds|2022-09|4|H5N1|USA|Spatuladiscors|USA|Alphainfluenzavirus|Alphainfluenzavirusinfluenz">
<date value="2022.6657534246576" direction="forwards" units="years"/>
</taxon>
<taxon id="OQ733012.1|InfluenzaAvirus_A/blue-wingedteal/Texas/UGAI22-3250/2022_H5N1__segment4hemagglutinin_HA_genecompletecds|2022-10|4|H5N1|USA|Spatuladiscors|USA|Alphainfluenzavirus|Alphainfluenzavirusinfluenz">
<date value="2022.6931506849314" direction="forwards" units="years"/>
</taxon>
<taxon id="OQ733028.1|InfluenzaAvirus_A/blue-wingedteal/Texas/UGAI22-3189/2022_H5N1__segment4hemagglutinin_HA_genecompletecds|2022-10|4|H5N1|USA|Spatuladiscors|USA|Alphainfluenzavirus|Alphainfluenzavirusinfluenz">
<date value="2022.6931506849314" direction="forwards" units="years"/>
</taxon>
<taxon id="OQ733036.1|InfluenzaAvirus_A/blue-wingedteal/Minnesota/AV22-675/2022_H5N1__segment4hemagglutinin_HA_genecompletecds|2022-09|4|H5N1|USA|Spatuladiscors|USA|Alphainfluenzavirus|Alphainfluenzavirusinfluenz">
<date value="2022.6657534246576" direction="forwards" units="years"/>
</taxon>
<taxon id="OQ733100.1|InfluenzaAvirus_A/blue-wingedteal/Texas/UGAI22-3190/2022_H5N1__segment4hemagglutinin_HA_genecompletecds|2022-10|4|H5N1|USA|Spatuladiscors|USA|Alphainfluenzavirus|Alphainfluenzavirusinfluenz">
<date value="2022.6931506849314" direction="forwards" units="years"/>
</taxon>
<taxon id="OQ733108.1|InfluenzaAvirus_A/blue-wingedteal/Texas/UGAI22-2961/2022_H5N1__segment4hemagglutinin_HA_genecompletecds|2022-09|4|H5N1|USA|Spatuladiscors|USA|Alphainfluenzavirus|Alphainfluenzavirusinfluenz">
<date value="2022.6657534246576" direction="forwards" units="years"/>
</taxon>
<taxon id="OQ734894.1|InfluenzaAvirus_A/Americangreen-wingedteal/Texas/UGAI22-3462/2022_H5N1__segment4hemagglutinin_HA_genecompletecds|2022-09-21|4|H5N1|USA|Anascarolinensis|USA|Alphainfluenzavirus|Alphainfluenzavirusinfluenz">
<date value="2022.7232876712328" direction="forwards" units="years"/>
</taxon>
<taxon id="OQ747759.1|InfluenzaAvirus_A/Belcher_s_gull/Peru/A102/2022_H5N1__segment4hemagglutinin_HA_genecompletecds|2022-11|4|H5N1|Peru||Peru|Alphainfluenzavirus|Alphainfluenzavirusinfluenz">
<date value="2022.8328767123287" direction="forwards" units="years"/>

</taxon>
<taxon id="OQ747761.1|InfluenzaAvirus_A/Belcher_s_gull/Peru/A267/2022_H5N1__segment4hemagglutinin_HA_genecompletecds|2022-12|4|H5N1|Peru||Peru|Alphainfluenzavirus|Alphainfluenzavirusinfluenz">
<date value="2022.9150684931508" direction="forwards" units="years"/>
</taxon>
<taxon id="OQ747762.1|InfluenzaAvirus_A/Americankestrel/Peru/A273/2022_H5N1__segment4hemagglutinin_HA_genecompletecds|2022-12|4|H5N1|Peru|Falcosparverius|Peru|Alphainfluenzavirus|Alphainfluenzavirusinfluenz">
<date value="2022.9150684931508" direction="forwards" units="years"/>
</taxon>
<taxon id="OQ851647.1|InfluenzaAvirus_A/Pekinduck/California/T2202390/2022_H5N1__segment4hemagglutinin_HA_genecompletecds|2022-11-14|4|H5N1|USA|Anasplatyrhynchos|USA_California|Alphainfluenzavirus|Alphainfluenzavirusinfluenz">
<date value="2022.8712328767124" direction="forwards" units="years"/>
</taxon>
<taxon id="OQ925704.1|InfluenzaAvirus_A/pelican/Peru/PIU-SER013/2022_H5N1__segment4hemagglutinin_HA_genecompletecds|2022-11-23|4|H5N1|Peru|Pelecanidae|Peru|Alphainfluenzavirus|Alphainfluenzavirusinfluenz">
<date value="2022.8958904109588" direction="forwards" units="years"/>
</taxon>
<taxon id="OQ954544.1|InfluenzaAvirus_A/stripedskunk/Kansas/W23-175/2023_H5N1__segment4hemagglutinin_HA_genecompletecds|2023-02-24|4|H5N1|USA|Mephitismephitis|USA|Alphainfluenzavirus|Alphainfluenzavirusinfluenz">
<date value="2023.150684931507" direction="forwards" units="years"/>
</taxon>
<taxon id="OQ968028.1|InfluenzaAvirus_A/goose/Alaska/22-013831-003/2022_H5N1__segment4hemagglutinin_HA_genecompletecds|2022-04-27|4|H5N1|USA|Anatidae|USA_Alaska|Alphainfluenzavirus|Alphainfluenzavirusinfluenz">
<date value="2022.3205479452056" direction="forwards" units="years"/>
</taxon>
<taxon id="OQ982404.1|InfluenzaAvirus_A/blue-wingedteal/Louisiana/UGAI22-3889/2022_H5N1__segment4hemagglutinin_HA_genecompletecds|2022-09-23|4|H5N1|USA|Spatuladiscors|USA|Alphainfluenzavirus|Alphainfluenzavirusinfluenz">
<date value="2022.7287671232878" direction="forwards" units="years"/>
</taxon>
<taxon id="OQ982420.1|InfluenzaAvirus_A/blue-wingedteal/Louisiana/UGAI22-3875/2022_H5N1__segment4hemagglutinin_HA_genecompletecds|2022-09-23|4|H5N1|USA|Spatuladiscors|USA|Alphainfluenzavirus|Alphainfluenzavirusinfluenz">
<date value="2022.7287671232878" direction="forwards" units="years"/>
</taxon>
<taxon id="OR591549.1|InfluenzaAvirus_A/Guanaycormorant/Peru/A275/2022_H5N1__segment4hemagglutinin_HA_genecompletecds|2022-12|4|H5N1|Peru||Peru|Alphainfluenzavirus|Alphainfluenzavirusinfluenz">
<date value="2022.9150684931508" direction="forwards" units="years"/>
</taxon>
<taxon id="OR591550.1|InfluenzaAvirus_A/Peruvianbooby/Peru/A296/2022_H5N1__segment4hemagglutinin_HA_genecompletecds|2022-12|4|H5N1|Peru|Sulavariegata|Peru|Alphainfluenzavirus|Alphainfluenzavirusinfluenz">
<date value="2022.9150684931508" direction="forwards" units="years"/>
</taxon>

<taxon id="OR591551.1|InfluenzaAvirus_A/Belchergull/Peru/A267/2022_H5N1__segment4hemagglutinin_HA_genecompletecds|2022-12|4|H5N1|Peru||Peru|Alphainfluenzavirus|Alphainfluenzavirusinfluenz">
<date value="2022.9150684931508" direction="forwards" units="years"/>
</taxon>
<taxon id="OR910145.1|InfluenzaAvirus_A/graygull/Atacama/235521-1/2023_H5N1__segment4hemagglutinin_HA_genecompletecds|2023-02-05|4|H5N1|Chile|Leucophaeusmodestus|Chile|Alphainfluenzavirus|Alphainfluenzavirusinfluenz">
<date value="2023.0986301369862" direction="forwards" units="years"/>
</taxon>
<taxon id="OR910159.1|InfluenzaAvirus_A/cormorant/Antofagasta/236025-1/2023_H5N1__segment4hemagglutinin_HA_genecompletecds|2023-02-06|4|H5N1|Chile|Phalacrocorax|Chile|Alphainfluenzavirus|Alphainfluenzavirusinfluenz">
<date value="2023.1013698630136" direction="forwards" units="years"/>
</taxon>
<taxon id="OR910172.1|InfluenzaAvirus_A/chicken/Nuble/241585/2023_H5N1__segment4hemagglutinin_HA_genecompletecds|2023-03-13|4|H5N1|Chile|Gallusgallus|Chile|Alphainfluenzavirus|Alphainfluenzavirusinfluenz">
<date value="2023.1123287671232" direction="forwards" units="years"/>
</taxon>
<taxon id="OR910178.1|InfluenzaAvirus_A/Peruvianpelican/Nuble/236068-1/2023_H5N1__segment4hemagglutinin_HA_genecompletecds|2023-02-10|4|H5N1|Chile|Pelecanusthagus|Chile|Alphainfluenzavirus|Alphainfluenzavirusinfluenz">
<date value="2023.1123287671232" direction="forwards" units="years"/>
</taxon>
<taxon id="OR910186.1|InfluenzaAvirus_A/Band-tailedgull/Tarapaca/236339/2023_H5N1__segment4hemagglutinin_HA_genecompletecds|2023-02-10|4|H5N1|Chile|Larusbelcheri|Chile|Alphainfluenzavirus|Alphainfluenzavirusinfluenz">
<date value="2023.1123287671232" direction="forwards" units="years"/>
</taxon>
<taxon id="OR910192.1|InfluenzaAvirus_A/turkey/Valparaiso/245562-2/2023_H5N1__segment4hemagglutinin_HA_genecompletecds|2023-03-29|4|H5N1|Chile|Meleagrisgallopavo|Chile|Alphainfluenzavirus|Alphainfluenzavirusinfluenz">
<date value="2023.1561643835616" direction="forwards" units="years"/>
</taxon>
<taxon id="OR910199.1|InfluenzaAvirus_A/Peruvianbooby/Antofagasta/236613/2023_H5N1__segment4hemagglutinin_HA_genecompletecds|2023-02-13|4|H5N1|Chile|Sulavariegata|Chile|Alphainfluenzavirus|Alphainfluenzavirusinfluenz">
<date value="2023.1205479452055" direction="forwards" units="years"/>
</taxon>
<taxon id="OR910223.1|InfluenzaAvirus_A/pelican/AricayParinacota/235876-4/2023_H5N1__segment4hemagglutinin_HA_genecompletecds|2023-02-02|4|H5N1|Chile|Pelecanus|Chile|Alphainfluenzavirus|Alphainfluenzavirusinfluenz">
<date value="2023.0904109589042" direction="forwards" units="years"/>
</taxon>
<taxon id="OR910231.1|InfluenzaAvirus_A/chicken/Atacama/235254-6/2023_H5N1__segment4hemagglutinin_HA_genecompletecds|2023-02-04|4|H5N1|Chile|Gallusgallus|Chile|Alphainfluenzavirus|Alphainfluenzavirusinfluenz">
<date value="2023.0958904109589" direction="forwards" units="years"/>
</taxon>

<taxon id="OR910238.1|InfluenzaAvirus_A/chicken/Atacama/235254-1/2023_H5N1__segment4hemagglutinin_HA_genecompletecds|2023-02-04|4|H5N1|Chile|Gallusgallus|Chile|Alphainfluenzavirus|Alphainfluenzavirusinfluenz">
<date value="2023.0958904109589" direction="forwards" units="years"/>
</taxon>
<taxon id="OR910248.1|InfluenzaAvirus_A/Peruvianbooby/AricayParinacota/235900-1/2023_H5N1__segment4hemagglutinin_HA_genecompletecds|2023-02-05|4|H5N1|Chile|Sulavariegata|Chile|Alphainfluenzavirus|Alphainfluenzavirusinfluenz">
<date value="2023.0986301369862" direction="forwards" units="years"/>
</taxon>
<taxon id="OR910254.1|InfluenzaAvirus_A/pelican/BioBio/235399-1/2023_H5N1__segment4hemagglutinin_HA_genecompletecds|2023-02-06|4|H5N1|Chile|Pelecanus|Chile|Alphainfluenzavirus|Alphainfluenzavirusinfluenz">
<date value="2023.1013698630136" direction="forwards" units="years"/>
</taxon>
<taxon id="OR910261.1|InfluenzaAvirus_A/Turkeyvulture/Antofagasta/236643/2023_H5N1__segment4hemagglutinin_HA_genecompletecds|2023-02-13|4|H5N1|Chile|Cathartesaura|Chile|Alphainfluenzavirus|Alphainfluenzavirusinfluenz">
<date value="2023.1205479452055" direction="forwards" units="years"/>
</taxon>
<taxon id="OR910268.1|InfluenzaAvirus_A/kelpgull/OHiggins/235977-1/2023_H5N1__segment4hemagglutinin_HA_genecompletecds|2023-02-09|4|H5N1|Chile|Larusdominicanus|Chile|Alphainfluenzavirus|Alphainfluenzavirusinfluenz">
<date value="2023.109589041096" direction="forwards" units="years"/>
</taxon>
<taxon id="OR910275.1|InfluenzaAvirus_A/chicken/Nuble/242159/2023_H5N1__segment4hemagglutinin_HA_genecompletecds|2023-03-15|4|H5N1|Chile|Gallusgallus|Chile|Alphainfluenzavirus|Alphainfluenzavirusinfluenz">
<date value="2023.1178082191782" direction="forwards" units="years"/>
</taxon>
<taxon id="OR910282.1|InfluenzaAvirus_A/chicken/Maule/242947-1/2023_H5N1__segment4hemagglutinin_HA_genecompletecds|2023-03-17|4|H5N1|Chile|Gallusgallus|Chile|Alphainfluenzavirus|Alphainfluenzavirusinfluenz">
<date value="2023.123287671233" direction="forwards" units="years"/>
</taxon>
<taxon id="OR910295.1|InfluenzaAvirus_A/turkey/Valparaiso/245558-4/2023_H5N1__segment4hemagglutinin_HA_genecompletecds|2023-03-29|4|H5N1|Chile|Meleagrisgallopavo|Chile|Alphainfluenzavirus|Alphainfluenzavirusinfluenz">
<date value="2023.1561643835616" direction="forwards" units="years"/>
</taxon>
<taxon id="OR910301.1|InfluenzaAvirus_A/Peruvianbooby/Coquimbo/238918/2023_H5N1__segment4hemagglutinin_HA_genecompletecds|2023-02-25|4|H5N1|Chile|Sulavariegata|Chile|Alphainfluenzavirus|Alphainfluenzavirusinfluenz">
<date value="2023.1534246575343" direction="forwards" units="years"/>
</taxon>
<taxon id="OR910308.1|InfluenzaAvirus_A/chicken/Araucania/245202/2023_H5N1__segment4hemagglutinin_HA_genecompletecds|2023-03-28|4|H5N1|Chile|Gallusgallus|Chile|Alphainfluenzavirus|Alphainfluenzavirusinfluenz">
<date value="2023.1534246575343" direction="forwards" units="years"/>
</taxon>

<taxon id="OR910318.1|InfluenzaAvirus_A/band-tailedgull/AricayParinacota/235901-2/2023_H5N1__segment4hemagglutinin_HA_genecompletecds|2023-02-06|4|H5N1|Chile|Larusbelcheri|Chile|Alphainfluenzavirus|Alphainfluenzavirus influenz">
<date value="2023.1013698630136" direction="forwards" units="years"/>
</taxon>
<taxon id="OR910331.1|InfluenzaAvirus_A/Peruvianbooby/AricayParinacota/235897-2/2023_H5N1__segment4hemagglutinin_HA_genecompletecds|2023-02-03|4|H5N1|Chile|Sulavariegata|Chile|Alphainfluenzavirus|Alphainfluenzavirus influenz">
<date value="2023.0931506849315" direction="forwards" units="years"/>
</taxon>
<taxon id="OR910346.1|InfluenzaAvirus_A/goose/Araucania/244373-1/2023_H5N1__segment4hemagglutinin_HA_genecompletecds|2023-03-21|4|H5N1|Chile|Anseranser|Chile|Alphainfluenzavirus|Alphainfluenzavirusinfluenz">
<date value="2023.1342465753426" direction="forwards" units="years"/>
</taxon>
<taxon id="OR910366.1|InfluenzaAvirus_A/Peruvianbooby/Coquimbo/239024/2023_H5N1__segment4hemagglutinin_HA_genecompletecds|2023-02-25|4|H5N1|Chile|Sulavariegata|Chile|Alphainfluenzavirus|Alphainfluenzavirus influenz">
<date value="2023.1534246575343" direction="forwards" units="years"/>
</taxon>
<taxon id="OR910371.1|InfluenzaAvirus_A/graygull/Antofagasta/236047-1/2023_H5N1__segment4hemagglutinin_HA_genecompletecds|2023-02-09|4|H5N1|Chile|Leucophaeusmodestus|Chile|Alphainfluenzavirus|Alphainfluenzavirusinfluenz">
<date value="2023.109589041096" direction="forwards" units="years"/>
</taxon>
<taxon id="OR910384.1|InfluenzaAvirus_A/chicken/BioBio/241781/2023_H5N1__segment4hemagglutinin_HA_genecompletecds|2023-03-14|4|H5N1|Chile|Gallusgallus|Chile|Alphainfluenzavirus|Alphainfluenzavirusinfluenz">
<date value="2023.1150684931506" direction="forwards" units="years"/>
</taxon>
<taxon id="OR910389.1|InfluenzaAvirus_A/turkey/Valparaiso/245558-3/2023_H5N1__segment4hemagglutinin_HA_genecompletecds|2023-03-29|4|H5N1|Chile|Meleagrisgallopavo|Chile|Alphainfluenzavirus|Alphainfluenzavirusinfluenz">
<date value="2023.1561643835616" direction="forwards" units="years"/>
</taxon>
<taxon id="OR910396.1|InfluenzaAvirus_A/chicken/Atacama/235254-5/2023_H5N1__segment4hemagglutinin_HA_genecompletecds|2023-02-04|4|H5N1|Chile|Gallusgallus|Chile|Alphainfluenzavirus|Alphainfluenzavirusinfluenz">
<date value="2023.0958904109589" direction="forwards" units="years"/>
</taxon>
<taxon id="OR910404.1|InfluenzaAvirus_A/Peruvianbooby/Tarapaca/236308/2023_H5N1__segment4hemagglutinin_HA_genecompletecds|2023-02-09|4|H5N1|Chile|Sulavariegata|Chile|Alphainfluenzavirus|Alphainfluenzavirus influenz">
<date value="2023.109589041096" direction="forwards" units="years"/>
</taxon>
<taxon id="OR910409.1|InfluenzaAvirus_A/domesticduck/OHiggins/245741-2/2023_H5N1__segment4hemagglutinin_HA_genecompletecds|2023-03-30|4|H5N1|Chile|Anasplatyrhynchos|Chile|Alphainfluenzavirus|Alphainfluenzavirusinfluenz">
<date value="2023.158904109589" direction="forwards" units="years"/>
</taxon>

<taxon id="OR910415.1|InfluenzaAvirus_A/turkeyvulture/Atacama/241294/2023_H
5N1__segment4hemagglutinin_HA_genecompletecds|2023-03-
07|4|H5N1|Chile|Coragypsatratus|Chile|Alphainfluenzavirus|Alphainfluenzavir
usinfluenz">
<date value="2023.0958904109589" direction="forwards" units="years"/>
</taxon>
<taxon id="OR910419.1|InfluenzaAvirus_A/Peruvianbooby/Valparaiso/236604/202
3_H5N1__segment4hemagglutinin_HA_genecompletecds|2023-02-
13|4|H5N1|Chile|Sulavariegata|Chile|Alphainfluenzavirus|Alphainfluenzavirus
influenz">
<date value="2023.1205479452055" direction="forwards" units="years"/>
</taxon>
<taxon id="OR910425.1|InfluenzaAvirus_A/domesticduck/Araucania/243954-
1/2023_H5N1__segment4hemagglutinin_HA_genecompletecds|2023-03-
21|4|H5N1|Chile|Anasplatyrhynchos|Chile|Alphainfluenzavirus|Alphainfluenzav
irusinfluenz">
<date value="2023.1342465753426" direction="forwards" units="years"/>
</taxon>
<taxon id="OR910435.1|InfluenzaAvirus_A/chicken/BioBio/245039-
2/2023_H5N1__segment4hemagglutinin_HA_genecompletecds|2023-03-
27|4|H5N1|Chile|Gallusgallus|Chile|Alphainfluenzavirus|Alphainfluenzavirusi
nfluenz">
<date value="2023.150684931507" direction="forwards" units="years"/>
</taxon>
<taxon id="OR910447.1|InfluenzaAvirus_A/graygull/Tarapaca/236305/2023_H5N1_
_segment4hemagglutinin_HA_genecompletecds|2023-02-
11|4|H5N1|Chile|Leucophaeusmodestus|Chile|Alphainfluenzavirus|Alphainfluenz
avirusinfluenz">
<date value="2023.1150684931506" direction="forwards" units="years"/>
</taxon>
<taxon id="OR910460.1|InfluenzaAvirus_A/domesticduck/Metropolitana/243148-
2/2023_H5N1__segment4hemagglutinin_HA_genecompletecds|2023-03-
20|4|H5N1|Chile|Anasplatyrhynchos|Chile|Alphainfluenzavirus|Alphainfluenzav
irusinfluenz">
<date value="2023.131506849315" direction="forwards" units="years"/>
</taxon>
<taxon id="OR910468.1|InfluenzaAvirus_A/SouthAmericansealion/Valparaiso/243
136-1/2023_H5N1__segment4hemagglutinin_HA_genecompletecds|2023-03-
19|4|H5N1|Chile|Otariabyronia|Chile|Alphainfluenzavirus|Alphainfluenzavirus
influenz">
<date value="2023.1287671232876" direction="forwards" units="years"/>
</taxon>
<taxon id="OR910474.1|InfluenzaAvirus_A/chicken/Maule/242133-
1/2023_H5N1__segment4hemagglutinin_HA_genecompletecds|2023-03-
15|4|H5N1|Chile|Gallusgallus|Chile|Alphainfluenzavirus|Alphainfluenzavirusi
nfluenz">
<date value="2023.1178082191782" direction="forwards" units="years"/>
</taxon>
<taxon id="OR910477.1|InfluenzaAvirus_A/chicken/Metropolitana/245704-
1/2023_H5N1__segment4hemagglutinin_HA_genecompletecds|2023-03-
30|4|H5N1|Chile|Gallusgallus|Chile|Alphainfluenzavirus|Alphainfluenzavirusi
nfluenz">
<date value="2023.158904109589" direction="forwards" units="years"/>
</taxon>
<taxon id="OR910481.1|InfluenzaAvirus_A/SouthAmericansealion/Valparaiso/244
738-1/2023_H5N1__segment4hemagglutinin_HA_genecompletecds|2023-03-
26|4|H5N1|Chile|Otariabyronia|Chile|Alphainfluenzavirus|Alphainfluenzavirus
influenz">
<date value="2023.1479452054793" direction="forwards" units="years"/>
</taxon>

<taxon id="OR910487.1|InfluenzaAvirus_A/greatgrabe/Nuble/244226-1/2023_H5N1__segment4hemagglutinin_HA_genecompletecds|2023-03-22|4|H5N1|Chile|Podicepsmajor|Chile|Alphainfluenzavirus|Alphainfluenzavirus influenz">
<date value="2023.13698630137" direction="forwards" units="years"/>
</taxon>
<taxon id="OR910502.1|InfluenzaAvirus_A/goose/Araucania/244409-2/2023_H5N1__segment4hemagglutinin_HA_genecompletecds|2023-03-24|4|H5N1|Chile|Anseranser|Chile|Alphainfluenzavirus|Alphainfluenzavirusinfluenz">
<date value="2023.1424657534246" direction="forwards" units="years"/>
</taxon>
<taxon id="OR910509.1|InfluenzaAvirus_A/Peruvianbooby/AricayParinacota/234906-1/2023_H5N1__segment4hemagglutinin_HA_genecompletecds|2023-01-31|4|H5N1|Chile|Sulavariegata|Chile|Alphainfluenzavirus|Alphainfluenzavirus influenz">
<date value="2023.0849315068492" direction="forwards" units="years"/>
</taxon>
<taxon id="OR910515.1|InfluenzaAvirus_A/penguin/Antofagasta/234905-1/2023_H5N1__segment4hemagglutinin_HA_genecompletecds|2023-01-27|4|H5N1|Chile|Pygoscelis|Chile|Alphainfluenzavirus|Alphainfluenzavirusinfluenz">
<date value="2023.0739726027398" direction="forwards" units="years"/>
</taxon>
<taxon id="OR910521.1|InfluenzaAvirus_A/kelpgull/Nuble/234619-1/2023_H5N1__segment4hemagglutinin_HA_genecompletecds|2023-01-30|4|H5N1|Chile|Larusdominicanus|Chile|Alphainfluenzavirus|Alphainfluenzavirusinfluenz">
<date value="2023.0821917808219" direction="forwards" units="years"/>
</taxon>
<taxon id="OR960985.1|InfluenzaAvirus_A/Chileandolphin/Maule/246026/2023_H5N1__segment4hemagglutinin_HA_genecompletecds|2023-03-31|4|H5N1|Chile|Cephalorhynchuseutropia|Chile|Alphainfluenzavirus|Alphainfluenzavirusinfluenz">
<date value="2023.1616438356164" direction="forwards" units="years"/>
</taxon>
<taxon id="OR960992.1|InfluenzaAvirus_A/guanaycormorant/Tarapaca/236301/2023_H5N1__segment4hemagglutinin_HA_genecompletecds|2023-02-09|4|H5N1|Chile|Leucocarbobougainvillii|Chile|Alphainfluenzavirus|Alphainfluenzavirusinfluenz">
<date value="2023.109589041096" direction="forwards" units="years"/>
</taxon>
<taxon id="OR960998.1|InfluenzaAvirus_A/guanaycormorant/Atacama/235246-1/2023_H5N1__segment4hemagglutinin_HA_genecompletecds|2023-02-03|4|H5N1|Chile|Leucocarbobougainvillii|Chile|Alphainfluenzavirus|Alphainfluenzavirusinfluenz">
<date value="2023.0931506849315" direction="forwards" units="years"/>
</taxon>
<taxon id="OR961005.1|InfluenzaAvirus_A/guanaycormorant/AricayParinacota/235902-2/2023_H5N1__segment4hemagglutinin_HA_genecompletecds|2023-02-06|4|H5N1|Chile|Leucocarbobougainvillii|Chile|Alphainfluenzavirus|Alphainfluenzavirusinfluenz">
<date value="2023.1013698630136" direction="forwards" units="years"/>
</taxon>
<taxon id="OR961011.1|InfluenzaAvirus_A/guanaycormorant/Coquimbo/239080/2023_H5N1__segment4hemagglutinin_HA_genecompletecds|2023-02-27|4|H5N1|Chile|Leucocarbobougainvillii|Chile|Alphainfluenzavirus|Alphainfluenzavirusinfluenz">
<date value="2023.158904109589" direction="forwards" units="years"/>
</taxon>

<taxon id="OR979626.1|InfluenzaAvirus_A/SouthAmericansealion/BioBio/246296-1/2023_H5N1__segment4hemagglutinin_HA_genecompletecds|2023-03-26|4|H5N1|Chile|Otariabyronia|Chile|Alphainfluenzavirus|Alphainfluenzavirusinfluenz">
<date value="2023.1479452054793" direction="forwards" units="years"/>
</taxon>
<taxon id="OR987084.1|InfluenzaAvirus_A/SouthAmericansealion/Argentina/RN-PB004/2023_H5N1__segment4hemagglutinin_HA_genecompletecds|2023-08-26|4|H5N1|Argentina|Otariabyronia|Argentina|Alphainfluenzavirus|Alphainfluenzavirusinfluenz">
<date value="2023.6520547945206" direction="forwards" units="years"/>
</taxon>
<taxon id="PP692235.1|InfluenzaAvirus_A/Calidrisalba/Lima/HA_4/2023_H5N1__segment4hemagglutinin_HA_genecompletecds|2023-04-20|4|H5N1|Peru|Calidrisalba|Peru_Lima|Alphainfluenzavirus|Alphainfluenzavirusinfluenz">
<date value="2023.3013698630136" direction="forwards" units="years"/>
</taxon>
<taxon id="PP761738.1|InfluenzaAvirus_A/snowgoose/SouthDakota/22-038501-001-original/2022_H5N1__segment4hemagglutinin_HA_genecompletecds|2022-11-20|4|H5N1|USA|Ansercaerulescens|USA|Alphainfluenzavirus|Alphainfluenzavirusinfluenz">
<date value="2022.8876712328768" direction="forwards" units="years"/>
</taxon>
<taxon id="PP801655.1|InfluenzaAvirus_A/commonraven/USA/22-020951-004/2022_H5N1__segment4hemagglutinin_HA_genecompletecds|2022-06-26|4|H5N1|USA|Corvuscorax|USA|Alphainfluenzavirus|Alphainfluenzavirusinfluenz">
<date value="2022.4849315068493" direction="forwards" units="years"/>
</taxon>
<taxon id="PP801696.1|InfluenzaAvirus_A/mallard/USA/22-029097-002/2022_H5N1__segment4hemagglutinin_HA_genecompletecds|2022-09-03|4|H5N1|USA|Anasplatyrhynchos|USA|Alphainfluenzavirus|Alphainfluenzavirusinfluenz">
<date value="2022.6739726027397" direction="forwards" units="years"/>
</taxon>
<taxon id="PP801703.1|InfluenzaAvirus_A/northernpintail/USA/22-029101-003/2022_H5N1__segment4hemagglutinin_HA_genecompletecds|2022-09-05|4|H5N1|USA|Anasacuta|USA|Alphainfluenzavirus|Alphainfluenzavirusinfluenz">
<date value="2022.6794520547944" direction="forwards" units="years"/>
</taxon>
<taxon id="PP801708.1|InfluenzaAvirus_A/green-wingedteal/USA/22-029101-004/2022_H5N1__segment4hemagglutinin_HA_genecompletecds|2022-09-05|4|H5N1|USA|Anascarolinensis|USA|Alphainfluenzavirus|Alphainfluenzavirusinfluenz">
<date value="2022.6794520547944" direction="forwards" units="years"/>
</taxon>
<taxon id="PP801715.1|InfluenzaAvirus_A/chicken/USA/22-031261-002/2022_H5N1__segment4hemagglutinin_HA_genecompletecds|2022-09-28|4|H5N1|USA|Gallusgallus|USA|Alphainfluenzavirus|Alphainfluenzavirusinfluenz">
<date value="2022.7424657534248" direction="forwards" units="years"/>
</taxon>
<taxon id="PP801722.1|InfluenzaAvirus_A/chicken/USA/22-031261-004/2022_H5N1__segment4hemagglutinin_HA_genecompletecds|2022-09-28|4|H5N1|USA|Gallusgallus|USA|Alphainfluenzavirus|Alphainfluenzavirusinfluenz">
<date value="2022.7424657534248" direction="forwards" units="years"/>
</taxon>

<taxon id="PP801727.1|InfluenzaAvirus_A/chicken/USA/22-031261-005/2022_H5N1__segment4hemagglutinin_HA_genecompletecds|2022-09-28|4|H5N1|USA|Gallusgallus|USA|Alphainfluenzavirus|Alphainfluenzavirusinfluenz">
<date value="2022.7424657534248" direction="forwards" units="years"/>
</taxon>
<taxon id="PP801741.1|InfluenzaAvirus_A/green-wingedteal/USA/22-031399-018/2022_H5N1__segment4hemagglutinin_HA_genecompletecds|2022-09-02|4|H5N1|USA|Anascarolinensis|USA|Alphainfluenzavirus|Alphainfluenzavirusinfluenz">
<date value="2022.6712328767123" direction="forwards" units="years"/>
</taxon>
<taxon id="PP801748.1|InfluenzaAvirus_A/chicken/USA/22-032677-005/2022_H5N1__segment4hemagglutinin_HA_genecompletecds|2022-10-11|4|H5N1|USA|Gallusgallus|USA|Alphainfluenzavirus|Alphainfluenzavirusinfluenz">
<date value="2022.7232876712328" direction="forwards" units="years"/>
</taxon>
<taxon id="PP801781.1|InfluenzaAvirus_A/northernpintail/USA/22-034099-001/2022_H5N1__segment4hemagglutinin_HA_genecompletecds|2022-10-05|4|H5N1|USA|Anasacuta|USA|Alphainfluenzavirus|Alphainfluenzavirusinfluenz">
<date value="2022.7068493150684" direction="forwards" units="years"/>
</taxon>
<taxon id="PP801792.1|InfluenzaAvirus_A/mallard/USA/22-036139-002/2022_H5N1__segment4hemagglutinin_HA_genecompletecds|2022-11-02|4|H5N1|USA|Anasplatyrhynchos|USA|Alphainfluenzavirus|Alphainfluenzavirusinfluenz">
<date value="2022.8383561643836" direction="forwards" units="years"/>
</taxon>
<taxon id="PP801798.1|InfluenzaAvirus_A/mallard/USA/22-036139-016/2022_H5N1__segment4hemagglutinin_HA_genecompletecds|2022-11-02|4|H5N1|USA|Anasplatyrhynchos|USA|Alphainfluenzavirus|Alphainfluenzavirusinfluenz">
<date value="2022.8383561643836" direction="forwards" units="years"/>
</taxon>
<taxon id="PP801802.1|InfluenzaAvirus_A/mallard/USA/22-036139-018/2022_H5N1__segment4hemagglutinin_HA_genecompletecds|2022-11-02|4|H5N1|USA|Anasplatyrhynchos|USA|Alphainfluenzavirus|Alphainfluenzavirusinfluenz">
<date value="2022.8383561643836" direction="forwards" units="years"/>
</taxon>
<taxon id="PP801810.1|InfluenzaAvirus_A/mallard/USA/22-036139-054/2022_H5N1__segment4hemagglutinin_HA_genecompletecds|2022-11-02|4|H5N1|USA|Anasplatyrhynchos|USA|Alphainfluenzavirus|Alphainfluenzavirusinfluenz">
<date value="2022.8383561643836" direction="forwards" units="years"/>
</taxon>
<taxon id="PP801816.1|InfluenzaAvirus_A/mallard/USA/22-036139-057/2022_H5N1__segment4hemagglutinin_HA_genecompletecds|2022-11-02|4|H5N1|USA|Anasplatyrhynchos|USA|Alphainfluenzavirus|Alphainfluenzavirusinfluenz">
<date value="2022.8383561643836" direction="forwards" units="years"/>
</taxon>
<taxon id="PP801843.1|InfluenzaAvirus_A/greatblueheron/USA/22-036616-001/2022_H5N1__segment4hemagglutinin_HA_genecompletecds|2022-11-08|4|H5N1|USA|Ardeaherodias|USA|Alphainfluenzavirus|Alphainfluenzavirusinfluenz">
<date value="2022.854794520548" direction="forwards" units="years"/>
</taxon>

<taxon id="PP801863.1|InfluenzaAvirus_A/chicken/USA/22-037940-001/2022_H5N1__segment4hemagglutinin_HA_genecompletecds|2022-11-23|4|H5N1|USA|Gallusgallus|USA|Alphainfluenzavirus|Alphainfluenzavirusinfluenz">
<date value="2022.8958904109588" direction="forwards" units="years"/>
</taxon>
<taxon id="PP801974.1|InfluenzaAvirus_A/mallard/USA/IZ22_0788/2022_H5N1__segment4hemagglutinin_HA_genecompletecds|2022-09-30|4|H5N1|USA|Anasplatyrhynchos|USA|Alphainfluenzavirus|Alphainfluenzavirusinfluenz">
<date value="2022.7479452054795" direction="forwards" units="years"/>
</taxon>
<taxon id="PP801982.1|InfluenzaAvirus_A/Americanwigeon/USA/22-034097-002/2022_H5N1__segment4hemagglutinin_HA_genecompletecds|2022-10-01|4|H5N1|USA|Marecaamericana|USA|Alphainfluenzavirus|Alphainfluenzavirusinfluenz">
<date value="2022.695890410959" direction="forwards" units="years"/>
</taxon>
<taxon id="PP802022.1|InfluenzaAvirus_A/Canadagoose/USA/22-013831-003/2022_H5N1__segment4hemagglutinin_HA_genecompletecds|2022-04-29|4|H5N1|USA|Brantacanadensis|USA|Alphainfluenzavirus|Alphainfluenzavirusinfluenz">
<date value="2022.3260273972603" direction="forwards" units="years"/>
</taxon>
<taxon id="PP802254.1|InfluenzaAvirus_A/cacklinggoose/USA/IZ22_0116/2022_H5N1__segment4hemagglutinin_HA_genecompletecds|2022-09-05|4|H5N1|USA|Brantahutchinsii|USA|Alphainfluenzavirus|Alphainfluenzavirusinfluenz">
<date value="2022.6794520547944" direction="forwards" units="years"/>
</taxon>
<taxon id="PP802262.1|InfluenzaAvirus_A/cacklinggoose/USA/IZ22_0117/2022_H5N1__segment4hemagglutinin_HA_genecompletecds|2022-09-05|4|H5N1|USA|Brantahutchinsii|USA|Alphainfluenzavirus|Alphainfluenzavirusinfluenz">
<date value="2022.6794520547944" direction="forwards" units="years"/>
</taxon>
<taxon id="PP802270.1|InfluenzaAvirus_A/cacklinggoose/USA/IZ22_0251/2022_H5N1__segment4hemagglutinin_HA_genecompletecds|2022-09-09|4|H5N1|USA|Brantahutchinsii|USA|Alphainfluenzavirus|Alphainfluenzavirusinfluenz">
<date value="2022.690410958904" direction="forwards" units="years"/>
</taxon>
<taxon id="PP802278.1|InfluenzaAvirus_A/cacklinggoose/USA/IZ22_0787/2022_H5N1__segment4hemagglutinin_HA_genecompletecds|2022-09-30|4|H5N1|USA|Brantahutchinsii|USA|Alphainfluenzavirus|Alphainfluenzavirusinfluenz">
<date value="2022.7479452054795" direction="forwards" units="years"/>
</taxon>
<taxon id="PP802310.1|InfluenzaAvirus_A/chicken/USA/22-031261-003/2022_H5N1__segment4hemagglutinin_HA_genecompletecds|2022-09-28|4|H5N1|USA|Gallusgallus|USA|Alphainfluenzavirus|Alphainfluenzavirusinfluenz">
<date value="2022.7424657534248" direction="forwards" units="years"/>
</taxon>
<taxon id="PP802318.1|InfluenzaAvirus_A/chicken/USA/22-032677-003/2022_H5N1__segment4hemagglutinin_HA_genecompletecds|2022-10-11|4|H5N1|USA|Gallusgallus|USA|Alphainfluenzavirus|Alphainfluenzavirusinfluenz">
<date value="2022.7232876712328" direction="forwards" units="years"/>
</taxon>

<taxon id="PP802350.1|InfluenzaAvirus_A/commonraven/USA/22-022817-001/2022_H5N1__segment4hemagglutinin_HA_genecompletecds|2022-07-20|4|H5N1|USA|Corvuscorax|USA|Alphainfluenzavirus|Alphainfluenzavirusinfluenz">
<date value="2022.5506849315068" direction="forwards" units="years"/>
</taxon>
<taxon id="PP802390.1|InfluenzaAvirus_A/domesticturkey/USA/22-032677-001/2022_H5N1__segment4hemagglutinin_HA_genecompletecds|2022-10-11|4|H5N1|USA|Meleagrisgallopavo|USA|Alphainfluenzavirus|Alphainfluenzavirusinfluenz">
<date value="2022.7232876712328" direction="forwards" units="years"/>
</taxon>
<taxon id="PP802398.1|InfluenzaAvirus_A/domesticturkey/USA/22-032677-004/2022_H5N1__segment4hemagglutinin_HA_genecompletecds|2022-10-11|4|H5N1|USA|Meleagrisgallopavo|USA|Alphainfluenzavirus|Alphainfluenzavirusinfluenz">
<date value="2022.7232876712328" direction="forwards" units="years"/>
</taxon>
<taxon id="PP802406.1|InfluenzaAvirus_A/domesticturkey/USA/22-032677-006/2022_H5N1__segment4hemagglutinin_HA_genecompletecds|2022-10-11|4|H5N1|USA|Meleagrisgallopavo|USA|Alphainfluenzavirus|Alphainfluenzavirusinfluenz">
<date value="2022.7232876712328" direction="forwards" units="years"/>
</taxon>
<taxon id="PP802518.1|InfluenzaAvirus_A/green-wingedteal/USA/22-031399-019/2022_H5N1__segment4hemagglutinin_HA_genecompletecds|2022-09-02|4|H5N1|USA|Anascarolinensis|USA|Alphainfluenzavirus|Alphainfluenzavirusinfluenz">
<date value="2022.6712328767123" direction="forwards" units="years"/>
</taxon>
<taxon id="PP802622.1|InfluenzaAvirus_A/mallard/USA/22-036139-048/2022_H5N1__segment4hemagglutinin_HA_genecompletecds|2022-11-02|4|H5N1|USA|Anasplatyrhynchos|USA|Alphainfluenzavirus|Alphainfluenzavirusinfluenz">
<date value="2022.8383561643836" direction="forwards" units="years"/>
</taxon>
<taxon id="PP802654.1|InfluenzaAvirus_A/mallard/USA/IZ22_0392/2022_H5N1__segment4hemagglutinin_HA_genecompletecds|2022-09-16|4|H5N1|USA|Anasplatyrhynchos|USA|Alphainfluenzavirus|Alphainfluenzavirusinfluenz">
<date value="2022.7095890410958" direction="forwards" units="years"/>
</taxon>
<taxon id="PP802662.1|InfluenzaAvirus_A/mallard/USA/IZ22_0766/2022_H5N1__segment4hemagglutinin_HA_genecompletecds|2022-09-30|4|H5N1|USA|Anasplatyrhynchos|USA|Alphainfluenzavirus|Alphainfluenzavirusinfluenz">
<date value="2022.7479452054795" direction="forwards" units="years"/>
</taxon>
<taxon id="PP802670.1|InfluenzaAvirus_A/mallard/USA/IZ22_0791/2022_H5N1__segment4hemagglutinin_HA_genecompletecds|2022-09-30|4|H5N1|USA|Anasplatyrhynchos|USA|Alphainfluenzavirus|Alphainfluenzavirusinfluenz">
<date value="2022.7479452054795" direction="forwards" units="years"/>
</taxon>
<taxon id="PP802678.1|InfluenzaAvirus_A/mallard/USA/IZ22_0879/2022_H5N1__segment4hemagglutinin_HA_genecompletecds|2022-10-09|4|H5N1|USA|Anasplatyrhynchos|USA|Alphainfluenzavirus|Alphainfluenzavirusinfluenz">
<date value="2022.717808219178" direction="forwards" units="years"/>
</taxon>

<taxon id="PP802686.1|InfluenzaAvirus_A/mallard/USA/IZ22_0894/2022_H5N1__segment4hemagglutinin_HA_genecompletecds|2022-10-14|4|H5N1|USA|Anasplatyrhynchos|USA|Alphainfluenzavirus|Alphainfluenzavirusinfluenz">
<date value="2022.731506849315" direction="forwards" units="years"/>
</taxon>
<taxon id="PP802702.1|InfluenzaAvirus_A/northernpintail/USA/22-031399-012/2022_H5N1__segment4hemagglutinin_HA_genecompletecds|2022-09-02|4|H5N1|USA|Anasacuta|USA|Alphainfluenzavirus|Alphainfluenzavirusinfluenz">
<date value="2022.6712328767123" direction="forwards" units="years"/>
</taxon>
<taxon id="PP802710.1|InfluenzaAvirus_A/northernpintail/USA/22-031399-015/2022_H5N1__segment4hemagglutinin_HA_genecompletecds|2022-09-02|4|H5N1|USA|Anasacuta|USA|Alphainfluenzavirus|Alphainfluenzavirusinfluenz">
<date value="2022.6712328767123" direction="forwards" units="years"/>
</taxon>
<taxon id="PQ002146.1|InfluenzaAvirus_A/Southernelephantseal/PeninsulaValdes/HA_CH-PD032_brain/2023_H5N1__segment4hemagglutinin_HA_genecompletecds|2023-10-10|4|H5N1|Argentina|Miroungaleonina|Argentina_ChubutPeninsulaValdes|Alphainfluenzavirus|">
<date value="2023.7205479452055" direction="forwards" units="years"/>
</taxon>
<taxon id="PQ113953.1|InfluenzaAvirus_A/SouthernElephantSeal/SouthGeorgiaandtheSouthSandwichIslands/24/2023_H5N1__segment4hemagglutinin_HA_genecompletecds|2023-12-09|4|H5N1|SouthGeorgiaandtheSouthSandwichIslands|Miroungaleonina|SouthGe">
<date value="2023.9397260273972" direction="forwards" units="years"/>
</taxon>
<taxon id="PQ113961.1|InfluenzaAvirus_A/KelpGull/SouthGeorgiaandtheSouthSandwichIslands/32/2023_H5N1__segment4hemagglutinin_HA_genecompletecds|2023-11-03|4|H5N1|SouthGeorgiaandtheSouthSandwichIslands|Larusdominicanus|SouthGeorgiaandt">
<date value="2023.841095890411" direction="forwards" units="years"/>
</taxon>
<taxon id="PQ113969.1|InfluenzaAvirus_A/AntarcticTern/SouthGeorgiaandtheSouthSandwichIslands/40/2023_H5N1__segment4hemagglutinin_HA_genecompletecds|2023-12-06|4|H5N1|SouthGeorgiaandtheSouthSandwichIslands|Sternavittata|SouthGeorgiaand">
<date value="2023.9315068493152" direction="forwards" units="years"/>
</taxon>
<taxon id="PQ113977.1|InfluenzaAvirus_A/BrownSkua/SouthGeorgiaandtheSouthSandwichIslands/48/2023_H5N1__segment4hemagglutinin_HA_genecompletecds|2023-10-31|4|H5N1|SouthGeorgiaandtheSouthSandwichIslands|Stercorariuslonnbergi|SouthGeorgia">
<date value="2023.7780821917809" direction="forwards" units="years"/>
</taxon>
<taxon id="PQ113985.1|InfluenzaAvirus_A/KelpGull/SouthGeorgiaandtheSouthSandwichIslands/56/2023_H5N1__segment4hemagglutinin_HA_genecompletecds|2023-10-31|4|H5N1|SouthGeorgiaandtheSouthSandwichIslands|Larusdominicanus|SouthGeorgiaandt">
<date value="2023.7780821917809" direction="forwards" units="years"/>
</taxon>
<taxon id="PQ113993.1|InfluenzaAvirus_A/BrownSkua/SouthGeorgiaandtheSouthSandwichIslands/64/2023_H5N1__segment4hemagglutinin_HA_genecompletecds|2023-

10-
31|4|H5N1|SouthGeorgiaandtheSouthSandwichIslands|Stercorariuslonnbergi|Sout
hGeorgia">
<date value="2023.7780821917809" direction="forwards" units="years"/>
</taxon>
<taxon id="PQ114017.1|InfluenzaAvirus_A/SouthGeorgiaShag/SouthGeorgiaandthe
SouthSandwichIslands/88/2023_H5N1__segment4hemagglutinin_HA_genecompletecds
|2023-11-
27|4|H5N1|SouthGeorgiaandtheSouthSandwichIslands|Leucocarbogeorgianus|South
G">
<date value="2023.9068493150685" direction="forwards" units="years"/>
</taxon>
<taxon id="PQ114025.1|InfluenzaAvirus_A/BrownSkua/SouthGeorgiaandtheSouthSa
ndwichIslands/96/2023_H5N1__segment4hemagglutinin_HA_genecompletecds|2023-
10-
30|4|H5N1|SouthGeorgiaandtheSouthSandwichIslands|Stercorariuslonnbergi|Sout
hGeorgia">
<date value="2023.7753424657535" direction="forwards" units="years"/>
</taxon>
<taxon id="PQ114033.1|InfluenzaAvirus_A/BrownSkua/SouthGeorgiaandtheSouthSa
ndwichIslands/104/2023_H5N1__segment4hemagglutinin_HA_genecompletecds|2023-
11-
15|4|H5N1|SouthGeorgiaandtheSouthSandwichIslands|Stercorariuslonnbergi|Sout
hGeorgi">
<date value="2023.8739726027397" direction="forwards" units="years"/>
</taxon>
<taxon id="PQ114073.1|InfluenzaAvirus_A/BrownSkua/SouthGeorgiaandtheSouthSa
ndwichIslands/144/2023_H5N1__segment4hemagglutinin_HA_genecompletecds|2023-
11-
11|4|H5N1|SouthGeorgiaandtheSouthSandwichIslands|Stercorariuslonnbergi|Sout
hGeorgi">
<date value="2023.86301369863" direction="forwards" units="years"/>
</taxon>
<taxon id="PQ114081.1|InfluenzaAvirus_A/KelpGull/SouthGeorgiaandtheSouthSan
dwichIslands/152/2023_H5N1__segment4hemagglutinin_HA_genecompletecds|2023-
10-
30|4|H5N1|SouthGeorgiaandtheSouthSandwichIslands|Larusdominicanus|SouthGeor
giaandt">
<date value="2023.7753424657535" direction="forwards" units="years"/>
</taxon>
<taxon id="PQ129506.1|InfluenzaAvirus_A/BrownSkua/SouthGeorgiaandtheSouthSa
ndwichIslands/8/2023_H5N1__segment4hemagglutinin_HA_genecompletecds|2023-
10-
08|4|H5N1|SouthGeorgiaandtheSouthSandwichIslands|Stercorariuslonnbergi|Sout
hGeorgiaa">
<date value="2023.7150684931507" direction="forwards" units="years"/>
</taxon>
<taxon id="PQ129514.1|InfluenzaAvirus_A/BrownSkua/SouthGeorgiaandtheSouthSa
ndwichIslands/16/2023_H5N1__segment4hemagglutinin_HA_genecompletecds|2023-
10-
08|4|H5N1|SouthGeorgiaandtheSouthSandwichIslands|Stercorariuslonnbergi|Sout
hGeorgia">
<date value="2023.7150684931507" direction="forwards" units="years"/>
</taxon>
<taxon id="PQ304566.1|InfluenzaAvirus_A/Southpolarskua/Antarctica/INACH-UC-
UCHILE-SKU5L/2024_H5N1__segment4hemagglutinin_HA_genecompletecds|2024-03-
03|4|H5N1|Chile|Stercorariusmaccormicki|Chile|Alphainfluenzavirus|Alphainfl
uenzavirusinfluenz">
<date value="2024.0874316939892" direction="forwards" units="years"/>
</taxon>

<taxon id="PQ701225.1|InfluenzaAvirus_A/AmericanBlue-wingedteal/MN/22-029882-002-original/2022_H5N1__segment4hemagglutinin_HA_genecompletecds|2022-09-11|4|H5N1|USA||USA_MN|Alphainfluenzavirus|Alphainfluenzavirusinfluenz">
<date value="2022.695890410959" direction="forwards" units="years"/>
</taxon>
<taxon id="PQ701297.1|InfluenzaAvirus_A/AmericanBlue-wingedteal/OK/22-032994-002-original/2022_H5N1__segment4hemagglutinin_HA_genecompletecds|2022-10-10|4|H5N1|USA||USA_OK|Alphainfluenzavirus|Alphainfluenzavirusinfluenz">
<date value="2022.7205479452055" direction="forwards" units="years"/>
</taxon>
<taxon id="PQ701305.1|InfluenzaAvirus_A/AmericanBlue-wingedteal/TX/22-029888-002-original/2022_H5N1__segment4hemagglutinin_HA_genecompletecds|2022-09-11|4|H5N1|USA||USA_TX|Alphainfluenzavirus|Alphainfluenzavirusinfluenz">
<date value="2022.695890410959" direction="forwards" units="years"/>
</taxon>
<taxon id="PQ701313.1|InfluenzaAvirus_A/AmericanBlue-wingedteal/TX/22-036754-004-original-repeat/2022_H5N1__segment4hemagglutinin_HA_genecompletecds|2022-11-06|4|H5N1|USA||USA_TX|Alphainfluenzavirus|Alphainfluenzavirusinfluenz">
<date value="2022.849315068493" direction="forwards" units="years"/>
</taxon>
<taxon id="PQ701329.1|InfluenzaAvirus_A/AmericanblackDuck/VA/22-038573-010-original/2022_H5N1__segment4hemagglutinin_HA_genecompletecds|2022-11-19|4|H5N1|USA|Anasrubripes|USA_VA|Alphainfluenzavirus|Alphainfluenzavirusinfluenz">
<date value="2022.8849315068494" direction="forwards" units="years"/>
</taxon>
<taxon id="PQ701569.1|InfluenzaAvirus_A/Americancrow/OR/23-002234-001-original/2022_H5N1__segment4hemagglutinin_HA_genecompletecds|2022-11-16|4|H5N1|USA|Corvusbrachyrhynchos|USA_OR|Alphainfluenzavirus|Alphainfluenzavirusinfluenz">
<date value="2022.876712328767" direction="forwards" units="years"/>
</taxon>
<taxon id="PQ701633.1|InfluenzaAvirus_A/Americangreen-wingedteal/AK/22-029101-004-original/2022_H5N1__segment4hemagglutinin_HA_genecompletecds|2022-09-05|4|H5N1|USA|Anascarolinensis|USA_AK|Alphainfluenzavirus|Alphainfluenzavirusinfluenz">
<date value="2022.6794520547944" direction="forwards" units="years"/>
</taxon>
<taxon id="PQ701649.1|InfluenzaAvirus_A/Americangreen-wingedteal/AK/22-039438-007-original/2022_H5N1__segment4hemagglutinin_HA_genecompletecds|2022-09-07|4|H5N1|USA|Anascarolinensis|USA_AK|Alphainfluenzavirus|Alphainfluenzavirusinfluenz">
<date value="2022.6849315068494" direction="forwards" units="years"/>
</taxon>
<taxon id="PQ701665.1|InfluenzaAvirus_A/Americangreen-wingedteal/CA/23-005876-017-original/2022_H5N1__segment4hemagglutinin_HA_genecompletecds|2022-10-23|4|H5N1|USA|Anascarolinensis|USA_CA|Alphainfluenzavirus|Alphainfluenzavirusinfluenz">
<date value="2022.7561643835616" direction="forwards" units="years"/>
</taxon>
<taxon id="PQ701697.1|InfluenzaAvirus_A/Americangreen-wingedteal/IA/22-034928-001-original-repeat2/2022_H5N1__segment4hemagglutinin_HA_genecompletecds|2022-10-

16|4|H5N1|USA|Anascarolinensis|USA_IA|Alphainfluenzavirus|Alphainfluenzavirusinfluenz">
<date value="2022.7369863013698" direction="forwards" units="years"/>
</taxon>
<taxon id="PQ701705.1|InfluenzaAvirus_A/Americangreen-wingedteal/ID/22-034533-007-original-repeat2/2022_H5N1__segment4hemagglutinin_HA_genecompletecds|2022-10-01|4|H5N1|USA|Anascarolinensis|USA_ID|Alphainfluenzavirus|Alphainfluenzavirusinfluenz">
<date value="2022.695890410959" direction="forwards" units="years"/>
</taxon>
<taxon id="PQ701745.1|InfluenzaAvirus_A/Americangreen-wingedteal/MN/23-013078-030-original/2022_H5N1__segment4hemagglutinin_HA_genecompletecds|2022-09-17|4|H5N1|USA|Anascarolinensis|USA_MN|Alphainfluenzavirus|Alphainfluenzavirusinfluenz">
<date value="2022.7123287671234" direction="forwards" units="years"/>
</taxon>
<taxon id="PQ701793.1|InfluenzaAvirus_A/Americangreen-wingedteal/ND/22-032267-001-original-repeat/2022_H5N1__segment4hemagglutinin_HA_genecompletecds|2022-10-01|4|H5N1|USA|Anascarolinensis|USA_ND|Alphainfluenzavirus|Alphainfluenzavirusinfluenz">
<date value="2022.695890410959" direction="forwards" units="years"/>
</taxon>
<taxon id="PQ701809.1|InfluenzaAvirus_A/Americangreen-wingedteal/NY/22-033592-029-original/2022_H5N1__segment4hemagglutinin_HA_genecompletecds|2022-10-15|4|H5N1|USA|Anascarolinensis|USA_NY|Alphainfluenzavirus|Alphainfluenzavirusinfluenz">
<date value="2022.7342465753425" direction="forwards" units="years"/>
</taxon>
<taxon id="PQ701833.1|InfluenzaAvirus_A/Americangreen-wingedteal/OR/22-037268-011-original/2022_H5N1__segment4hemagglutinin_HA_genecompletecds|2022-11-12|4|H5N1|USA|Anascarolinensis|USA_OR|Alphainfluenzavirus|Alphainfluenzavirusinfluenz">
<date value="2022.8657534246574" direction="forwards" units="years"/>
</taxon>
<taxon id="PQ701953.1|InfluenzaAvirus_A/AmericanwhitePelican/CA/22-035596-001-original/2022_H5N1__segment4hemagglutinin_HA_genecompletecds|2022-10-27|4|H5N1|USA|Pelecanuserythrorhynchos|USA_CA|Alphainfluenzavirus|Alphainfluenzavirusinfluenz">
<date value="2022.7671232876712" direction="forwards" units="years"/>
</taxon>
<taxon id="PQ702113.1|InfluenzaAvirus_A/AmericanwhitePelican/ND/22-032266-001-original-repeat/2022_H5N1__segment4hemagglutinin_HA_genecompletecds|2022-09-28|4|H5N1|USA|Pelecanuserythrorhynchos|USA_ND|Alphainfluenzavirus|Alphainfluenzavirusinfluen">
<date value="2022.7424657534248" direction="forwards" units="years"/>
</taxon>
<taxon id="PQ702161.1|InfluenzaAvirus_A/AmericanwhitePelican/UT/22-032952-010-original/2022_H5N1__segment4hemagglutinin_HA_genecompletecds|2022-10-13|4|H5N1|USA|Pelecanuserythrorhynchos|USA_UT|Alphainfluenzavirus|Alphainfluenzavirusinfluenz">
<date value="2022.7287671232878" direction="forwards" units="years"/>
</taxon>
<taxon id="PQ702273.1|InfluenzaAvirus_A/Americanwigeon/CA/23-001148-019-original/2022_H5N1__segment4hemagglutinin_HA_genecompletecds|2022-10-

22|4|H5N1|USA|Marecaamericana|USA_CA|Alphainfluenzavirus|Alphainfluenzaviru
sinfluenz">
<date value="2022.7534246575342" direction="forwards" units="years"/>
</taxon>
<taxon id="PQ702289.1|InfluenzaAvirus_A/Americanwigeon/ID/22-036873-001-
original/2022_H5N1__segment4hemagglutinin_HA_genecompletecds|2022-10-
26|4|H5N1|USA|Marecaamericana|USA_ID|Alphainfluenzavirus|Alphainfluenzaviru
sinfluenz">
<date value="2022.7643835616439" direction="forwards" units="years"/>
</taxon>
<taxon id="PQ702297.1|InfluenzaAvirus_A/Americanwigeon/ID/22-039598-001-
original/2022_H5N1__segment4hemagglutinin_HA_genecompletecds|2022-11-
23|4|H5N1|USA|Marecaamericana|USA_ID|Alphainfluenzavirus|Alphainfluenzaviru
sinfluenz">
<date value="2022.8958904109588" direction="forwards" units="years"/>
</taxon>
<taxon id="PQ702337.1|InfluenzaAvirus_A/Americanwigeon/OH/22-034080-032-
original/2022_H5N1__segment4hemagglutinin_HA_genecompletecds|2022-10-
15|4|H5N1|USA|Marecaamericana|USA_OH|Alphainfluenzavirus|Alphainfluenzaviru
sinfluenz">
<date value="2022.7342465753425" direction="forwards" units="years"/>
</taxon>
<taxon id="PQ702353.1|InfluenzaAvirus_A/Americanwigeon/OR/22-033876-004-
original/2022_H5N1__segment4hemagglutinin_HA_genecompletecds|2022-10-
15|4|H5N1|USA|Marecaamericana|USA_OR|Alphainfluenzavirus|Alphainfluenzaviru
sinfluenz">
<date value="2022.7342465753425" direction="forwards" units="years"/>
</taxon>
<taxon id="PQ702361.1|InfluenzaAvirus_A/Americanwigeon/OR/22-033876-019-
original/2022_H5N1__segment4hemagglutinin_HA_genecompletecds|2022-10-
15|4|H5N1|USA|Marecaamericana|USA_OR|Alphainfluenzavirus|Alphainfluenzaviru
sinfluenz">
<date value="2022.7342465753425" direction="forwards" units="years"/>
</taxon>
<taxon id="PQ702369.1|InfluenzaAvirus_A/Americanwigeon/OR/22-033877-003-
original/2022_H5N1__segment4hemagglutinin_HA_genecompletecds|2022-10-
16|4|H5N1|USA|Marecaamericana|USA_OR|Alphainfluenzavirus|Alphainfluenzaviru
sinfluenz">
<date value="2022.7369863013698" direction="forwards" units="years"/>
</taxon>
<taxon id="PQ702377.1|InfluenzaAvirus_A/Americanwigeon/OR/22-033877-019-
original-repeat2/2022_H5N1__segment4hemagglutinin_HA_genecompletecds|2022-
10-
16|4|H5N1|USA|Marecaamericana|USA_OR|Alphainfluenzavirus|Alphainfluenzaviru
sinfluenz">
<date value="2022.7369863013698" direction="forwards" units="years"/>
</taxon>
<taxon id="PQ702385.1|InfluenzaAvirus_A/Americanwigeon/OR/22-034677-053-
original-repeat2/2022_H5N1__segment4hemagglutinin_HA_genecompletecds|2022-
10-
08|4|H5N1|USA|Marecaamericana|USA_OR|Alphainfluenzavirus|Alphainfluenzaviru
sinfluenz">
<date value="2022.7150684931507" direction="forwards" units="years"/>
</taxon>
<taxon id="PQ702393.1|InfluenzaAvirus_A/Americanwigeon/OR/22-034677-056-
original-repeat2/2022_H5N1__segment4hemagglutinin_HA_genecompletecds|2022-
10-
08|4|H5N1|USA|Marecaamericana|USA_OR|Alphainfluenzavirus|Alphainfluenzaviru
sinfluenz">
<date value="2022.7150684931507" direction="forwards" units="years"/>
</taxon>

<taxon id="PQ702401.1|InfluenzaAvirus_A/Americanwigeon/OR/22-035736-015-original/2022_H5N1__segment4hemagglutinin_HA_genecompletecds|2022-10-27|4|H5N1|USA|Marecaamericana|USA_OR|Alphainfluenzavirus|Alphainfluenzavirusinfluenz">
<date value="2022.7671232876712" direction="forwards" units="years"/>
</taxon>
<taxon id="PQ702409.1|InfluenzaAvirus_A/Americanwigeon/OR/22-036880-012-original/2022_H5N1__segment4hemagglutinin_HA_genecompletecds|2022-11-07|4|H5N1|USA|Marecaamericana|USA_OR|Alphainfluenzavirus|Alphainfluenzavirusinfluenz">
<date value="2022.8520547945207" direction="forwards" units="years"/>
</taxon>
<taxon id="PQ702449.1|InfluenzaAvirus_A/Americanwigeon/TX/22-036753-014-original-repeat/2022_H5N1__segment4hemagglutinin_HA_genecompletecds|2022-11-05|4|H5N1|USA|Marecaamericana|USA_TX|Alphainfluenzavirus|Alphainfluenzavirusinfluenz">
<date value="2022.8465753424657" direction="forwards" units="years"/>
</taxon>
<taxon id="PQ702457.1|InfluenzaAvirus_A/Americanwigeon/TX/22-036753-025-original-repeat/2022_H5N1__segment4hemagglutinin_HA_genecompletecds|2022-11-05|4|H5N1|USA|Marecaamericana|USA_TX|Alphainfluenzavirus|Alphainfluenzavirusinfluenz">
<date value="2022.8465753424657" direction="forwards" units="years"/>
</taxon>
<taxon id="PQ702465.1|InfluenzaAvirus_A/Americanwigeon/TX/22-036753-038-original-repeat/2022_H5N1__segment4hemagglutinin_HA_genecompletecds|2022-11-05|4|H5N1|USA|Marecaamericana|USA_TX|Alphainfluenzavirus|Alphainfluenzavirusinfluenz">
<date value="2022.8465753424657" direction="forwards" units="years"/>
</taxon>
<taxon id="PQ702473.1|InfluenzaAvirus_A/Americanwigeon/TX/22-036753-056-original-repeat/2022_H5N1__segment4hemagglutinin_HA_genecompletecds|2022-11-05|4|H5N1|USA|Marecaamericana|USA_TX|Alphainfluenzavirus|Alphainfluenzavirusinfluenz">
<date value="2022.8465753424657" direction="forwards" units="years"/>
</taxon>
<taxon id="PQ702513.1|InfluenzaAvirus_A/Arctictern/AK/22-020952-002-original/2022_H5N1__segment4hemagglutinin_HA_genecompletecds|2022-07-22|4|H5N1|USA|Sternaparadisaea|USA_AK|Alphainfluenzavirus|Alphainfluenzavirusinfluenz">
<date value="2022.5561643835617" direction="forwards" units="years"/>
</taxon>
<taxon id="PQ702529.1|InfluenzaAvirus_A/Baldeagle/AK/22-014971-001-original/2022_H5N1__segment4hemagglutinin_HA_genecompletecds|2022-05-09|4|H5N1|USA|Haliaeetusleucocephalus|USA_AK|Alphainfluenzavirus|Alphainfluenzavirusinfluenz">
<date value="2022.3534246575343" direction="forwards" units="years"/>
</taxon>
<taxon id="PQ702593.1|InfluenzaAvirus_A/Baldeagle/AK/22-016934-004-original/2022_H5N1__segment4hemagglutinin_HA_genecompletecds|2022-05-22|4|H5N1|USA|Haliaeetusleucocephalus|USA_AK|Alphainfluenzavirus|Alphainfluenzavirusinfluenz">
<date value="2022.3890410958904" direction="forwards" units="years"/>
</taxon>
<taxon id="PQ703625.1|InfluenzaAvirus_A/Baldeagle/nan/23-029035-005-original/2022_H5N1__segment4hemagglutinin_HA_genecompletecds|2022-11-

22|4|H5N1|USA|Haliaeetusleucocephalus|USA_nan|Alphainfluenzavirus|Alphainfluenzavirusinfluenz">
<date value="2022.8931506849315" direction="forwards" units="years"/>
</taxon>
<taxon id="PQ703713.1|InfluenzaAvirus_A/BlackSwan/CA/22-032922-001-original/2022_H5N1__segment4hemagglutinin_HA_genecompletecds|2022-10-12|4|H5N1|USA|Cygnusatratus|USA_CA|Alphainfluenzavirus|Alphainfluenzavirusinfluenz">
<date value="2022.7260273972602" direction="forwards" units="years"/>
</taxon>
<taxon id="PQ705609.1|InfluenzaAvirus_A/Blackbrant/AK/22-017953-001-original/2022_H5N1__segment4hemagglutinin_HA_genecompletecds|2022-06-06|4|H5N1|USA||USA_AK|Alphainfluenzavirus|Alphainfluenzavirusinfluenz">
<date value="2022.4301369863015" direction="forwards" units="years"/>
</taxon>
<taxon id="PQ705617.1|InfluenzaAvirus_A/Blackbrant/AK/22-017953-002-original/2022_H5N1__segment4hemagglutinin_HA_genecompletecds|2022-06-06|4|H5N1|USA||USA_AK|Alphainfluenzavirus|Alphainfluenzavirusinfluenz">
<date value="2022.4301369863015" direction="forwards" units="years"/>
</taxon>
<taxon id="PQ705633.1|InfluenzaAvirus_A/Blackbrant/AK/22-023553-002-original/2022_H5N1__segment4hemagglutinin_HA_genecompletecds|2022-06-22|4|H5N1|USA||USA_AK|Alphainfluenzavirus|Alphainfluenzavirusinfluenz">
<date value="2022.4739726027397" direction="forwards" units="years"/>
</taxon>
<taxon id="PQ705697.1|InfluenzaAvirus_A/Blue-wingedteal/IA/22-034304-005-original-repeat2/2022_H5N1__segment4hemagglutinin_HA_genecompletecds|2022-10-22|4|H5N1|USA|Spatuladiscors|USA_IA|Alphainfluenzavirus|Alphainfluenzavirusinfluenz">
<date value="2022.7534246575342" direction="forwards" units="years"/>
</taxon>
<taxon id="PQ705713.1|InfluenzaAvirus_A/Blue-wingedteal/IN/22-034829-003-original-repeat2/2022_H5N1__segment4hemagglutinin_HA_genecompletecds|2022-10-28|4|H5N1|USA|Spatuladiscors|USA_IN|Alphainfluenzavirus|Alphainfluenzavirusinfluenz">
<date value="2022.7698630136986" direction="forwards" units="years"/>
</taxon>
<taxon id="PQ705753.1|InfluenzaAvirus_A/Blue-wingedteal/LA/22-033477-002-original/2022_H5N1__segment4hemagglutinin_HA_genecompletecds|2022-09-20|4|H5N1|USA|Spatuladiscors|USA_LA|Alphainfluenzavirus|Alphainfluenzavirusinfluenz">
<date value="2022.7205479452055" direction="forwards" units="years"/>
</taxon>
<taxon id="PQ705761.1|InfluenzaAvirus_A/Blue-wingedteal/LA/22-033477-003-original/2022_H5N1__segment4hemagglutinin_HA_genecompletecds|2022-09-20|4|H5N1|USA|Spatuladiscors|USA_LA|Alphainfluenzavirus|Alphainfluenzavirusinfluenz">
<date value="2022.7205479452055" direction="forwards" units="years"/>
</taxon>
<taxon id="PQ705769.1|InfluenzaAvirus_A/Blue-wingedteal/LA/22-033477-004-original/2022_H5N1__segment4hemagglutinin_HA_genecompletecds|2022-09-23|4|H5N1|USA|Spatuladiscors|USA_LA|Alphainfluenzavirus|Alphainfluenzavirusinfluenz">
<date value="2022.7287671232878" direction="forwards" units="years"/>
</taxon>
<taxon id="PQ705777.1|InfluenzaAvirus_A/Blue-wingedteal/LA/22-033477-005-original/2022_H5N1__segment4hemagglutinin_HA_genecompletecds|2022-09-23|4|H5N1|USA|Spatuladiscors|USA_LA|Alphainfluenzavirus|Alphainfluenzavirusinfluenz">

<date value="2022.7287671232878" direction="forwards" units="years"/>
</taxon>
<taxon id="PQ705785.1|InfluenzaAvirus_A/Blue-wingedteal/LA/22-033477-008-original/2022_H5N1__segment4hemagglutinin_HA_genecompletecds|2022-09-23|4|H5N1|USA|Spatuladiscors|USA_LA|Alphainfluenzavirus|Alphainfluenzavirus influenz">
<date value="2022.7287671232878" direction="forwards" units="years"/>
</taxon>
<taxon id="PQ705793.1|InfluenzaAvirus_A/Blue-wingedteal/LA/22-033477-009-original/2022_H5N1__segment4hemagglutinin_HA_genecompletecds|2022-09-23|4|H5N1|USA|Spatuladiscors|USA_LA|Alphainfluenzavirus|Alphainfluenzavirus influenz">
<date value="2022.7287671232878" direction="forwards" units="years"/>
</taxon>
<taxon id="PQ705817.1|InfluenzaAvirus_A/Blue-wingedteal/LA/22-033477-013-original/2022_H5N1__segment4hemagglutinin_HA_genecompletecds|2022-09-23|4|H5N1|USA|Spatuladiscors|USA_LA|Alphainfluenzavirus|Alphainfluenzavirus influenz">
<date value="2022.7287671232878" direction="forwards" units="years"/>
</taxon>
<taxon id="PQ705825.1|InfluenzaAvirus_A/Blue-wingedteal/LA/22-033477-014-original/2022_H5N1__segment4hemagglutinin_HA_genecompletecds|2022-09-23|4|H5N1|USA|Spatuladiscors|USA_LA|Alphainfluenzavirus|Alphainfluenzavirus influenz">
<date value="2022.7287671232878" direction="forwards" units="years"/>
</taxon>
<taxon id="PQ705841.1|InfluenzaAvirus_A/Blue-wingedteal/LA/22-033477-019-original/2022_H5N1__segment4hemagglutinin_HA_genecompletecds|2022-09-23|4|H5N1|USA|Spatuladiscors|USA_LA|Alphainfluenzavirus|Alphainfluenzavirus influenz">
<date value="2022.7287671232878" direction="forwards" units="years"/>
</taxon>
<taxon id="PQ705849.1|InfluenzaAvirus_A/Blue-wingedteal/LA/22-033477-020-original/2022_H5N1__segment4hemagglutinin_HA_genecompletecds|2022-09-23|4|H5N1|USA|Spatuladiscors|USA_LA|Alphainfluenzavirus|Alphainfluenzavirus influenz">
<date value="2022.7287671232878" direction="forwards" units="years"/>
</taxon>
<taxon id="PQ705865.1|InfluenzaAvirus_A/Blue-wingedteal/LA/23-013080-002-original/2022_H5N1__segment4hemagglutinin_HA_genecompletecds|2022-09-23|4|H5N1|USA|Spatuladiscors|USA_LA|Alphainfluenzavirus|Alphainfluenzavirus influenz">
<date value="2022.7287671232878" direction="forwards" units="years"/>
</taxon>
<taxon id="PQ705873.1|InfluenzaAvirus_A/Blue-wingedteal/LA/23-013080-004-original/2022_H5N1__segment4hemagglutinin_HA_genecompletecds|2022-09-23|4|H5N1|USA|Spatuladiscors|USA_LA|Alphainfluenzavirus|Alphainfluenzavirus influenz">
<date value="2022.7287671232878" direction="forwards" units="years"/>
</taxon>
<taxon id="PQ705881.1|InfluenzaAvirus_A/Blue-wingedteal/LA/23-013080-005-original/2022_H5N1__segment4hemagglutinin_HA_genecompletecds|2022-09-23|4|H5N1|USA|Spatuladiscors|USA_LA|Alphainfluenzavirus|Alphainfluenzavirus influenz">
<date value="2022.7287671232878" direction="forwards" units="years"/>
</taxon>
<taxon id="PQ705889.1|InfluenzaAvirus_A/Blue-wingedteal/LA/23-013080-006-original/2022_H5N1__segment4hemagglutinin_HA_genecompletecds|2022-09-23|4|H5N1|USA|Spatuladiscors|USA_LA|Alphainfluenzavirus|Alphainfluenzavirus influenz">
<date value="2022.7287671232878" direction="forwards" units="years"/>

</taxon>
<taxon id="PQ705897.1|InfluenzaAvirus_A/Blue-wingedteal/LA/23-013080-007-original/2022_H5N1__segment4hemagglutinin_HA_genecompletecds|2022-09-23|4|H5N1|USA|Spatuladiscors|USA_LA|Alphainfluenzavirus|Alphainfluenzavirus influenz">
<date value="2022.7287671232878" direction="forwards" units="years"/>
</taxon>
<taxon id="PQ705913.1|InfluenzaAvirus_A/Blue-wingedteal/LA/23-013080-009-original/2022_H5N1__segment4hemagglutinin_HA_genecompletecds|2022-09-23|4|H5N1|USA|Spatuladiscors|USA_LA|Alphainfluenzavirus|Alphainfluenzavirus influenz">
<date value="2022.7287671232878" direction="forwards" units="years"/>
</taxon>
<taxon id="PQ705921.1|InfluenzaAvirus_A/Blue-wingedteal/LA/23-013080-010-original/2022_H5N1__segment4hemagglutinin_HA_genecompletecds|2022-09-23|4|H5N1|USA|Spatuladiscors|USA_LA|Alphainfluenzavirus|Alphainfluenzavirus influenz">
<date value="2022.7287671232878" direction="forwards" units="years"/>
</taxon>
<taxon id="PQ705937.1|InfluenzaAvirus_A/Blue-wingedteal/MN/22-030423-014-original-repeat/2022_H5N1__segment4hemagglutinin_HA_genecompletecds|2022-09-10|4|H5N1|USA|Spatuladiscors|USA_MN|Alphainfluenzavirus|Alphainfluenzavirus influenz">
<date value="2022.6931506849314" direction="forwards" units="years"/>
</taxon>
<taxon id="PQ705945.1|InfluenzaAvirus_A/Blue-wingedteal/MN/22-030423-022-original-repeat/2022_H5N1__segment4hemagglutinin_HA_genecompletecds|2022-09-10|4|H5N1|USA|Spatuladiscors|USA_MN|Alphainfluenzavirus|Alphainfluenzavirus influenz">
<date value="2022.6931506849314" direction="forwards" units="years"/>
</taxon>
<taxon id="PQ705953.1|InfluenzaAvirus_A/Blue-wingedteal/MN/22-030423-047-original/2022_H5N1__segment4hemagglutinin_HA_genecompletecds|2022-09-11|4|H5N1|USA|Spatuladiscors|USA_MN|Alphainfluenzavirus|Alphainfluenzavirus influenz">
<date value="2022.695890410959" direction="forwards" units="years"/>
</taxon>
<taxon id="PQ706017.1|InfluenzaAvirus_A/Blue-wingedteal/MN/23-005533-005-original/2022_H5N1__segment4hemagglutinin_HA_genecompletecds|2022-09-15|4|H5N1|USA|Spatuladiscors|USA_MN|Alphainfluenzavirus|Alphainfluenzavirus influenz">
<date value="2022.7068493150684" direction="forwards" units="years"/>
</taxon>
<taxon id="PQ706121.1|InfluenzaAvirus_A/Blue-wingedteal/MN/23-005533-021-original/2022_H5N1__segment4hemagglutinin_HA_genecompletecds|2022-09-15|4|H5N1|USA|Spatuladiscors|USA_MN|Alphainfluenzavirus|Alphainfluenzavirus influenz">
<date value="2022.7068493150684" direction="forwards" units="years"/>
</taxon>
<taxon id="PQ706161.1|InfluenzaAvirus_A/Blue-wingedteal/MN/23-005533-030-original/2022_H5N1__segment4hemagglutinin_HA_genecompletecds|2022-09-16|4|H5N1|USA|Spatuladiscors|USA_MN|Alphainfluenzavirus|Alphainfluenzavirus influenz">
<date value="2022.7095890410958" direction="forwards" units="years"/>
</taxon>
<taxon id="PQ706169.1|InfluenzaAvirus_A/Blue-wingedteal/MN/23-005533-031-original/2022_H5N1__segment4hemagglutinin_HA_genecompletecds|2022-09-16|4|H5N1|USA|Spatuladiscors|USA_MN|Alphainfluenzavirus|Alphainfluenzavirus influenz">

<date value="2022.7095890410958" direction="forwards" units="years"/>
</taxon>
<taxon id="PQ706177.1|InfluenzaAvirus_A/Blue-wingedteal/MN/23-005533-034-original/2022_H5N1__segment4hemagglutinin_HA_genecompletecds|2022-09-16|4|H5N1|USA|Spatuladiscors|USA_MN|Alphainfluenzavirus|Alphainfluenzavirus influenz">
<date value="2022.7095890410958" direction="forwards" units="years"/>
</taxon>
<taxon id="PQ706193.1|InfluenzaAvirus_A/Blue-wingedteal/MN/23-013078-004-original/2022_H5N1__segment4hemagglutinin_HA_genecompletecds|2022-09-13|4|H5N1|USA|Spatuladiscors|USA_MN|Alphainfluenzavirus|Alphainfluenzavirus influenz">
<date value="2022.7013698630137" direction="forwards" units="years"/>
</taxon>
<taxon id="PQ706209.1|InfluenzaAvirus_A/Blue-wingedteal/MN/23-013078-009-original/2022_H5N1__segment4hemagglutinin_HA_genecompletecds|2022-09-14|4|H5N1|USA|Spatuladiscors|USA_MN|Alphainfluenzavirus|Alphainfluenzavirus influenz">
<date value="2022.704109589041" direction="forwards" units="years"/>
</taxon>
<taxon id="PQ706249.1|InfluenzaAvirus_A/Blue-wingedteal/MN/23-013078-018-original/2022_H5N1__segment4hemagglutinin_HA_genecompletecds|2022-09-14|4|H5N1|USA|Spatuladiscors|USA_MN|Alphainfluenzavirus|Alphainfluenzavirus influenz">
<date value="2022.704109589041" direction="forwards" units="years"/>
</taxon>
<taxon id="PQ706281.1|InfluenzaAvirus_A/Blue-wingedteal/MN/23-013078-022-original/2022_H5N1__segment4hemagglutinin_HA_genecompletecds|2022-09-14|4|H5N1|USA|Spatuladiscors|USA_MN|Alphainfluenzavirus|Alphainfluenzavirus influenz">
<date value="2022.704109589041" direction="forwards" units="years"/>
</taxon>
<taxon id="PQ706289.1|InfluenzaAvirus_A/Blue-wingedteal/MN/23-013078-024-original/2022_H5N1__segment4hemagglutinin_HA_genecompletecds|2022-09-14|4|H5N1|USA|Spatuladiscors|USA_MN|Alphainfluenzavirus|Alphainfluenzavirus influenz">
<date value="2022.704109589041" direction="forwards" units="years"/>
</taxon>
<taxon id="PQ706297.1|InfluenzaAvirus_A/Blue-wingedteal/MN/23-013078-025-original/2022_H5N1__segment4hemagglutinin_HA_genecompletecds|2022-09-14|4|H5N1|USA|Spatuladiscors|USA_MN|Alphainfluenzavirus|Alphainfluenzavirus influenz">
<date value="2022.704109589041" direction="forwards" units="years"/>
</taxon>
<taxon id="PQ706313.1|InfluenzaAvirus_A/Blue-wingedteal/MT/22-031163-004-original-repeat/2022_H5N1__segment4hemagglutinin_HA_genecompletecds|2022-09-24|4|H5N1|USA|Spatuladiscors|USA_MT|Alphainfluenzavirus|Alphainfluenzavirus influenz">
<date value="2022.731506849315" direction="forwards" units="years"/>
</taxon>
<taxon id="PQ706329.1|InfluenzaAvirus_A/Blue-wingedteal/OK/22-029332-001-original/2022_H5N1__segment4hemagglutinin_HA_genecompletecds|2022-09-10|4|H5N1|USA|Spatuladiscors|USA_OK|Alphainfluenzavirus|Alphainfluenzavirus influenz">
<date value="2022.6931506849314" direction="forwards" units="years"/>
</taxon>
<taxon id="PQ706337.1|InfluenzaAvirus_A/Blue-wingedteal/OK/22-029332-004-original/2022_H5N1__segment4hemagglutinin_HA_genecompletecds|2022-09-10|4|H5N1|USA|Spatuladiscors|USA_OK|Alphainfluenzavirus|Alphainfluenzavirus influenz">

<date value="2022.6931506849314" direction="forwards" units="years"/>
</taxon>
<taxon id="PQ706345.1|InfluenzaAvirus_A/Blue-wingedteal/OK/22-029332-005-original/2022_H5N1__segment4hemagglutinin_HA_genecompletecds|2022-09-10|4|H5N1|USA|Spatuladiscors|USA_OK|Alphainfluenzavirus|Alphainfluenzavirus influenz">
<date value="2022.6931506849314" direction="forwards" units="years"/>
</taxon>
<taxon id="PQ706353.1|InfluenzaAvirus_A/Blue-wingedteal/OK/22-029332-006-original/2022_H5N1__segment4hemagglutinin_HA_genecompletecds|2022-09-10|4|H5N1|USA|Spatuladiscors|USA_OK|Alphainfluenzavirus|Alphainfluenzavirus influenz">
<date value="2022.6931506849314" direction="forwards" units="years"/>
</taxon>
<taxon id="PQ706361.1|InfluenzaAvirus_A/Blue-wingedteal/OK/22-029332-008-original/2022_H5N1__segment4hemagglutinin_HA_genecompletecds|2022-09-10|4|H5N1|USA|Spatuladiscors|USA_OK|Alphainfluenzavirus|Alphainfluenzavirus influenz">
<date value="2022.6931506849314" direction="forwards" units="years"/>
</taxon>
<taxon id="PQ706369.1|InfluenzaAvirus_A/Blue-wingedteal/OK/22-029332-011-original/2022_H5N1__segment4hemagglutinin_HA_genecompletecds|2022-09-10|4|H5N1|USA|Spatuladiscors|USA_OK|Alphainfluenzavirus|Alphainfluenzavirus influenz">
<date value="2022.6931506849314" direction="forwards" units="years"/>
</taxon>
<taxon id="PQ706377.1|InfluenzaAvirus_A/Blue-wingedteal/OK/22-029332-018-original/2022_H5N1__segment4hemagglutinin_HA_genecompletecds|2022-09-10|4|H5N1|USA|Spatuladiscors|USA_OK|Alphainfluenzavirus|Alphainfluenzavirus influenz">
<date value="2022.6931506849314" direction="forwards" units="years"/>
</taxon>
<taxon id="PQ706385.1|InfluenzaAvirus_A/Blue-wingedteal/OK/22-029332-019-original/2022_H5N1__segment4hemagglutinin_HA_genecompletecds|2022-09-10|4|H5N1|USA|Spatuladiscors|USA_OK|Alphainfluenzavirus|Alphainfluenzavirus influenz">
<date value="2022.6931506849314" direction="forwards" units="years"/>
</taxon>
<taxon id="PQ706393.1|InfluenzaAvirus_A/Blue-wingedteal/OK/22-029332-023-original/2022_H5N1__segment4hemagglutinin_HA_genecompletecds|2022-09-10|4|H5N1|USA|Spatuladiscors|USA_OK|Alphainfluenzavirus|Alphainfluenzavirus influenz">
<date value="2022.6931506849314" direction="forwards" units="years"/>
</taxon>
<taxon id="PQ706417.1|InfluenzaAvirus_A/Blue-wingedteal/OK/22-029332-034-original/2022_H5N1__segment4hemagglutinin_HA_genecompletecds|2022-09-10|4|H5N1|USA|Spatuladiscors|USA_OK|Alphainfluenzavirus|Alphainfluenzavirus influenz">
<date value="2022.6931506849314" direction="forwards" units="years"/>
</taxon>
<taxon id="PQ706433.1|InfluenzaAvirus_A/Blue-wingedteal/TX/22-031350-016-original-repeat/2022_H5N1__segment4hemagglutinin_HA_genecompletecds|2022-09-10|4|H5N1|USA|Spatuladiscors|USA_TX|Alphainfluenzavirus|Alphainfluenzavirus influenz">
<date value="2022.6931506849314" direction="forwards" units="years"/>
</taxon>
<taxon id="PQ706441.1|InfluenzaAvirus_A/Blue-wingedteal/TX/22-031351-010-original-repeat/2022_H5N1__segment4hemagglutinin_HA_genecompletecds|2022-09-

17|4|H5N1|USA|Spatuladiscors|USA_TX|Alphainfluenzavirus|Alphainfluenzavirus influenz">
<date value="2022.7123287671234" direction="forwards" units="years"/>
</taxon>
<taxon id="PQ706449.1|InfluenzaAvirus_A/Blue-wingedteal/TX/22-031352-026-original-repeat/2022_H5N1__segment4hemagglutinin_HA_genecompletecds|2022-09-24|4|H5N1|USA|Spatuladiscors|USA_TX|Alphainfluenzavirus|Alphainfluenzavirus influenz">
<date value="2022.731506849315" direction="forwards" units="years"/>
</taxon>
<taxon id="PQ706457.1|InfluenzaAvirus_A/Blue-wingedteal/TX/22-031352-027-original-repeat/2022_H5N1__segment4hemagglutinin_HA_genecompletecds|2022-09-24|4|H5N1|USA|Spatuladiscors|USA_TX|Alphainfluenzavirus|Alphainfluenzavirus influenz">
<date value="2022.731506849315" direction="forwards" units="years"/>
</taxon>
<taxon id="PQ706465.1|InfluenzaAvirus_A/Blue-wingedteal/TX/22-031395-009-original-repeat/2022_H5N1__segment4hemagglutinin_HA_genecompletecds|2022-09-24|4|H5N1|USA|Spatuladiscors|USA_TX|Alphainfluenzavirus|Alphainfluenzavirus influenz">
<date value="2022.731506849315" direction="forwards" units="years"/>
</taxon>
<taxon id="PQ706481.1|InfluenzaAvirus_A/Blue-wingedteal/TX/22-033479-002-original/2022_H5N1__segment4hemagglutinin_HA_genecompletecds|2022-09-22|4|H5N1|USA|Spatuladiscors|USA_TX|Alphainfluenzavirus|Alphainfluenzavirus influenz">
<date value="2022.7260273972602" direction="forwards" units="years"/>
</taxon>
<taxon id="PQ706505.1|InfluenzaAvirus_A/Blue-wingedteal/TX/22-033479-007-original/2022_H5N1__segment4hemagglutinin_HA_genecompletecds|2022-09-22|4|H5N1|USA|Spatuladiscors|USA_TX|Alphainfluenzavirus|Alphainfluenzavirus influenz">
<date value="2022.7260273972602" direction="forwards" units="years"/>
</taxon>
<taxon id="PQ706513.1|InfluenzaAvirus_A/Blue-wingedteal/TX/22-033479-008-original/2022_H5N1__segment4hemagglutinin_HA_genecompletecds|2022-09-22|4|H5N1|USA|Spatuladiscors|USA_TX|Alphainfluenzavirus|Alphainfluenzavirus influenz">
<date value="2022.7260273972602" direction="forwards" units="years"/>
</taxon>
<taxon id="PQ706521.1|InfluenzaAvirus_A/Blue-wingedteal/TX/22-033479-009-original/2022_H5N1__segment4hemagglutinin_HA_genecompletecds|2022-09-22|4|H5N1|USA|Spatuladiscors|USA_TX|Alphainfluenzavirus|Alphainfluenzavirus influenz">
<date value="2022.7260273972602" direction="forwards" units="years"/>
</taxon>
<taxon id="PQ706529.1|InfluenzaAvirus_A/Blue-wingedteal/TX/22-033479-010-original/2022_H5N1__segment4hemagglutinin_HA_genecompletecds|2022-09-22|4|H5N1|USA|Spatuladiscors|USA_TX|Alphainfluenzavirus|Alphainfluenzavirus influenz">
<date value="2022.7260273972602" direction="forwards" units="years"/>
</taxon>
<taxon id="PQ706537.1|InfluenzaAvirus_A/Blue-wingedteal/TX/22-033479-011-original/2022_H5N1__segment4hemagglutinin_HA_genecompletecds|2022-09-22|4|H5N1|USA|Spatuladiscors|USA_TX|Alphainfluenzavirus|Alphainfluenzavirus influenz">
<date value="2022.7260273972602" direction="forwards" units="years"/>
</taxon>

<taxon id="PQ706561.1|InfluenzaAvirus_A/Blue-wingedteal/TX/22-033479-014-original/2022_H5N1__segment4hemagglutinin_HA_genecompletecds|2022-09-22|4|H5N1|USA|Spatuladiscors|USA_TX|Alphainfluenzavirus|Alphainfluenzavirus influenz">
<date value="2022.7260273972602" direction="forwards" units="years"/>
</taxon>
<taxon id="PQ706577.1|InfluenzaAvirus_A/Blue-wingedteal/TX/22-033479-017-original/2022_H5N1__segment4hemagglutinin_HA_genecompletecds|2022-09-24|4|H5N1|USA|Spatuladiscors|USA_TX|Alphainfluenzavirus|Alphainfluenzavirus influenz">
<date value="2022.731506849315" direction="forwards" units="years"/>
</taxon>
<taxon id="PQ706585.1|InfluenzaAvirus_A/Blue-wingedteal/TX/22-033479-018-original/2022_H5N1__segment4hemagglutinin_HA_genecompletecds|2022-09-24|4|H5N1|USA|Spatuladiscors|USA_TX|Alphainfluenzavirus|Alphainfluenzavirus influenz">
<date value="2022.731506849315" direction="forwards" units="years"/>
</taxon>
<taxon id="PQ706593.1|InfluenzaAvirus_A/Blue-wingedteal/TX/22-033479-019-original/2022_H5N1__segment4hemagglutinin_HA_genecompletecds|2022-09-24|4|H5N1|USA|Spatuladiscors|USA_TX|Alphainfluenzavirus|Alphainfluenzavirus influenz">
<date value="2022.731506849315" direction="forwards" units="years"/>
</taxon>
<taxon id="PQ706601.1|InfluenzaAvirus_A/Blue-wingedteal/TX/22-033479-020-original/2022_H5N1__segment4hemagglutinin_HA_genecompletecds|2022-09-24|4|H5N1|USA|Spatuladiscors|USA_TX|Alphainfluenzavirus|Alphainfluenzavirus influenz">
<date value="2022.731506849315" direction="forwards" units="years"/>
</taxon>
<taxon id="PQ706609.1|InfluenzaAvirus_A/Blue-wingedteal/TX/22-033479-021-original/2022_H5N1__segment4hemagglutinin_HA_genecompletecds|2022-09-24|4|H5N1|USA|Spatuladiscors|USA_TX|Alphainfluenzavirus|Alphainfluenzavirus influenz">
<date value="2022.731506849315" direction="forwards" units="years"/>
</taxon>
<taxon id="PQ706617.1|InfluenzaAvirus_A/Blue-wingedteal/TX/22-033479-023-original/2022_H5N1__segment4hemagglutinin_HA_genecompletecds|2022-09-24|4|H5N1|USA|Spatuladiscors|USA_TX|Alphainfluenzavirus|Alphainfluenzavirus influenz">
<date value="2022.731506849315" direction="forwards" units="years"/>
</taxon>
<taxon id="PQ706625.1|InfluenzaAvirus_A/Blue-wingedteal/TX/22-033479-024-original/2022_H5N1__segment4hemagglutinin_HA_genecompletecds|2022-09-24|4|H5N1|USA|Spatuladiscors|USA_TX|Alphainfluenzavirus|Alphainfluenzavirus influenz">
<date value="2022.731506849315" direction="forwards" units="years"/>
</taxon>
<taxon id="PQ706633.1|InfluenzaAvirus_A/Blue-wingedteal/TX/22-033479-025-original/2022_H5N1__segment4hemagglutinin_HA_genecompletecds|2022-09-24|4|H5N1|USA|Spatuladiscors|USA_TX|Alphainfluenzavirus|Alphainfluenzavirus influenz">
<date value="2022.731506849315" direction="forwards" units="years"/>
</taxon>
<taxon id="PQ706641.1|InfluenzaAvirus_A/Blue-wingedteal/TX/22-033479-026-original/2022_H5N1__segment4hemagglutinin_HA_genecompletecds|2022-09-24|4|H5N1|USA|Spatuladiscors|USA_TX|Alphainfluenzavirus|Alphainfluenzavirus influenz">
<date value="2022.731506849315" direction="forwards" units="years"/>
</taxon>

<taxon id="PQ706649.1|InfluenzaAvirus_A/Blue-wingedteal/TX/22-033479-028-original/2022_H5N1__segment4hemagglutinin_HA_genecompletecds|2022-09-24|4|H5N1|USA|Spatuladiscors|USA_TX|Alphainfluenzavirus|Alphainfluenzavirus influenz">
<date value="2022.731506849315" direction="forwards" units="years"/>
</taxon>
<taxon id="PQ706665.1|InfluenzaAvirus_A/Blue-wingedteal/TX/22-041132-005-original/2022_H5N1__segment4hemagglutinin_HA_genecompletecds|2022-09-20|4|H5N1|USA|Spatuladiscors|USA_TX|Alphainfluenzavirus|Alphainfluenzavirus influenz">
<date value="2022.7205479452055" direction="forwards" units="years"/>
</taxon>
<taxon id="PQ706673.1|InfluenzaAvirus_A/Blue-wingedteal/TX/22-041132-007-original/2022_H5N1__segment4hemagglutinin_HA_genecompletecds|2022-09-20|4|H5N1|USA|Spatuladiscors|USA_TX|Alphainfluenzavirus|Alphainfluenzavirus influenz">
<date value="2022.7205479452055" direction="forwards" units="years"/>
</taxon>
<taxon id="PQ706801.1|InfluenzaAvirus_A/CacklingGoose/AK/22-039438-003-original/2022_H5N1__segment4hemagglutinin_HA_genecompletecds|2022-09-05|4|H5N1|USA|Brantahutchinsii|USA_AK|Alphainfluenzavirus|Alphainfluenzavirusinfluenz">
<date value="2022.6794520547944" direction="forwards" units="years"/>
</taxon>
<taxon id="PQ706809.1|InfluenzaAvirus_A/CacklingGoose/AK/22-039438-004-original/2022_H5N1__segment4hemagglutinin_HA_genecompletecds|2022-09-05|4|H5N1|USA|Brantahutchinsii|USA_AK|Alphainfluenzavirus|Alphainfluenzavirusinfluenz">
<date value="2022.6794520547944" direction="forwards" units="years"/>
</taxon>
<taxon id="PQ706817.1|InfluenzaAvirus_A/CacklingGoose/AK/22-039438-012-original/2022_H5N1__segment4hemagglutinin_HA_genecompletecds|2022-09-09|4|H5N1|USA|Brantahutchinsii|USA_AK|Alphainfluenzavirus|Alphainfluenzavirusinfluenz">
<date value="2022.690410958904" direction="forwards" units="years"/>
</taxon>
<taxon id="PQ706825.1|InfluenzaAvirus_A/CacklingGoose/AK/23-002693-023-original/2022_H5N1__segment4hemagglutinin_HA_genecompletecds|2022-09-30|4|H5N1|USA|Brantahutchinsii|USA_AK|Alphainfluenzavirus|Alphainfluenzavirusinfluenz">
<date value="2022.7479452054795" direction="forwards" units="years"/>
</taxon>
<taxon id="PQ706905.1|InfluenzaAvirus_A/CacklingGoose/OR/22-036878-001-original-repeat2/2022_H5N1__segment4hemagglutinin_HA_genecompletecds|2022-10-02|4|H5N1|USA|Brantahutchinsii|USA_OR|Alphainfluenzavirus|Alphainfluenzavirusinfluenz">
<date value="2022.6986301369864" direction="forwards" units="years"/>
</taxon>
<taxon id="PQ706913.1|InfluenzaAvirus_A/CacklingGoose/OR/22-037259-001-original-repeat2/2022_H5N1__segment4hemagglutinin_HA_genecompletecds|2022-11-14|4|H5N1|USA|Brantahutchinsii|USA_OR|Alphainfluenzavirus|Alphainfluenzavirusinfluenz">
<date value="2022.8712328767124" direction="forwards" units="years"/>
</taxon>
<taxon id="PQ706929.1|InfluenzaAvirus_A/CacklingGoose/OR/22-037263-001-original/2022_H5N1__segment4hemagglutinin_HA_genecompletecds|2022-11-10|4|H5N1|USA|Brantahutchinsii|USA_OR|Alphainfluenzavirus|Alphainfluenzavirusinfluenz">
<date value="2022.8602739726027" direction="forwards" units="years"/>

</taxon>
<taxon id="PQ706945.1|InfluenzaAvirus_A/CacklingGoose/WA/22-037657-001-original/2022_H5N1__segment4hemagglutinin_HA_genecompletecds|2022-11-16|4|H5N1|USA|Brantahutchinsii|USA_WA|Alphainfluenzavirus|Alphainfluenzavirusinfluenz">
<date value="2022.876712328767" direction="forwards" units="years"/>
</taxon>
<taxon id="PQ707057.1|InfluenzaAvirus_A/Canadagoose/AK/22-014435-001-original/2022_H5N1__segment4hemagglutinin_HA_genecompletecds|2022-05-05|4|H5N1|USA|Brantacanadensis|USA_AK|Alphainfluenzavirus|Alphainfluenzavirusinfluenz">
<date value="2022.3424657534247" direction="forwards" units="years"/>
</taxon>
<taxon id="PQ707089.1|InfluenzaAvirus_A/Canadagoose/AZ/22-034128-001-original-repeat/2022_H5N1__segment4hemagglutinin_HA_genecompletecds|2022-10-17|4|H5N1|USA|Brantacanadensis|USA_AZ|Alphainfluenzavirus|Alphainfluenzavirusinfluenz">
<date value="2022.7397260273972" direction="forwards" units="years"/>
</taxon>
<taxon id="PQ707529.1|InfluenzaAvirus_A/Canadagoose/CO/22-033695-001-original/2022_H5N1__segment4hemagglutinin_HA_genecompletecds|2022-10-18|4|H5N1|USA|Brantacanadensis|USA_CO|Alphainfluenzavirus|Alphainfluenzavirusinfluenz">
<date value="2022.7424657534248" direction="forwards" units="years"/>
</taxon>
<taxon id="PQ707545.1|InfluenzaAvirus_A/Canadagoose/CO/22-038655-001-original/2022_H5N1__segment4hemagglutinin_HA_genecompletecds|2022-11-28|4|H5N1|USA|Brantacanadensis|USA_CO|Alphainfluenzavirus|Alphainfluenzavirusinfluenz">
<date value="2022.9095890410958" direction="forwards" units="years"/>
</taxon>
<taxon id="PQ707801.1|InfluenzaAvirus_A/Canadagoose/ID/22-014968-006-original/2022_H5N1__segment4hemagglutinin_HA_genecompletecds|2022-05-10|4|H5N1|USA|Brantacanadensis|USA_ID|Alphainfluenzavirus|Alphainfluenzavirusinfluenz">
<date value="2022.3561643835617" direction="forwards" units="years"/>
</taxon>
<taxon id="PQ707833.1|InfluenzaAvirus_A/Canadagoose/ID/22-017360-002-original/2022_H5N1__segment4hemagglutinin_HA_genecompletecds|2022-05-31|4|H5N1|USA|Brantacanadensis|USA_ID|Alphainfluenzavirus|Alphainfluenzavirusinfluenz">
<date value="2022.4136986301369" direction="forwards" units="years"/>
</taxon>
<taxon id="PQ707841.1|InfluenzaAvirus_A/Canadagoose/ID/22-018980-001-original/2022_H5N1__segment4hemagglutinin_HA_genecompletecds|2022-06-04|4|H5N1|USA|Brantacanadensis|USA_ID|Alphainfluenzavirus|Alphainfluenzavirusinfluenz">
<date value="2022.4246575342465" direction="forwards" units="years"/>
</taxon>
<taxon id="PQ708265.1|InfluenzaAvirus_A/Canadagoose/MT/22-033859-002-original/2022_H5N1__segment4hemagglutinin_HA_genecompletecds|2022-10-20|4|H5N1|USA|Brantacanadensis|USA_MT|Alphainfluenzavirus|Alphainfluenzavirusinfluenz">
<date value="2022.7479452054795" direction="forwards" units="years"/>
</taxon>
<taxon id="PQ708281.1|InfluenzaAvirus_A/Canadagoose/NC/22-036623-001-original-repeat2/2022_H5N1__segment4hemagglutinin_HA_genecompletecds|2022-10-30|4|H5N1|USA|Brantacanadensis|USA_NC|Alphainfluenzavirus|Alphainfluenzavirusinfluenz">

<date value="2022.7753424657535" direction="forwards" units="years"/>
</taxon>
<taxon id="PQ708313.1|InfluenzaAvirus_A/Canadagoose/ND/23-002238-004-
original/2022_H5N1__segment4hemagglutinin_HA_genecompletecds|2022-11-
29|4|H5N1|USA|Brantacanadensis|USA_ND|Alphainfluenzavirus|Alphainfluenzavir
usinfluenz">
<date value="2022.9123287671232" direction="forwards" units="years"/>
</taxon>
<taxon id="PQ708353.1|InfluenzaAvirus_A/Canadagoose/NV/22-033403-003-
original-repeat/2022_H5N1__segment4hemagglutinin_HA_genecompletecds|2022-
09-
23|4|H5N1|USA|Brantacanadensis|USA_NV|Alphainfluenzavirus|Alphainfluenzavir
usinfluenz">
<date value="2022.7287671232878" direction="forwards" units="years"/>
</taxon>
<taxon id="PQ708697.1|InfluenzaAvirus_A/Canadagoose/OR/22-037260-001-
original-repeat/2022_H5N1__segment4hemagglutinin_HA_genecompletecds|2022-
11-
02|4|H5N1|USA|Brantacanadensis|USA_OR|Alphainfluenzavirus|Alphainfluenzavir
usinfluenz">
<date value="2022.8383561643836" direction="forwards" units="years"/>
</taxon>
<taxon id="PQ708705.1|InfluenzaAvirus_A/Canadagoose/OR/22-037722-001-
original/2022_H5N1__segment4hemagglutinin_HA_genecompletecds|2022-11-
17|4|H5N1|USA|Brantacanadensis|USA_OR|Alphainfluenzavirus|Alphainfluenzavir
usinfluenz">
<date value="2022.8794520547945" direction="forwards" units="years"/>
</taxon>
<taxon id="PQ708929.1|InfluenzaAvirus_A/Canadagoose/WA/22-015526-002-
original/2022_H5N1__segment4hemagglutinin_HA_genecompletecds|2022-05-
11|4|H5N1|USA|Brantacanadensis|USA_WA|Alphainfluenzavirus|Alphainfluenzavir
usinfluenz">
<date value="2022.358904109589" direction="forwards" units="years"/>
</taxon>
<taxon id="PQ708937.1|InfluenzaAvirus_A/Canadagoose/WA/22-015526-003-
original/2022_H5N1__segment4hemagglutinin_HA_genecompletecds|2022-05-
11|4|H5N1|USA|Brantacanadensis|USA_WA|Alphainfluenzavirus|Alphainfluenzavir
usinfluenz">
<date value="2022.358904109589" direction="forwards" units="years"/>
</taxon>
<taxon id="PQ708945.1|InfluenzaAvirus_A/Canadagoose/WA/22-015526-005-
original/2022_H5N1__segment4hemagglutinin_HA_genecompletecds|2022-05-
09|4|H5N1|USA|Brantacanadensis|USA_WA|Alphainfluenzavirus|Alphainfluenzavir
usinfluenz">
<date value="2022.3534246575343" direction="forwards" units="years"/>
</taxon>
<taxon id="PQ709145.1|InfluenzaAvirus_A/Canadagoose/WY/22-033366-001-
original/2022_H5N1__segment4hemagglutinin_HA_genecompletecds|2022-10-
14|4|H5N1|USA|Brantacanadensis|USA_WY|Alphainfluenzavirus|Alphainfluenzavir
usinfluenz">
<date value="2022.731506849315" direction="forwards" units="years"/>
</taxon>
<taxon id="PQ709153.1|InfluenzaAvirus_A/Canadagoose/WY/22-036111-001-
original/2022_H5N1__segment4hemagglutinin_HA_genecompletecds|2022-11-
03|4|H5N1|USA|Brantacanadensis|USA_WY|Alphainfluenzavirus|Alphainfluenzavir
usinfluenz">
<date value="2022.841095890411" direction="forwards" units="years"/>
</taxon>
<taxon id="PQ709201.1|InfluenzaAvirus_A/Canadagoose/WY/23-000336-002-
original/2022_H5N1__segment4hemagglutinin_HA_genecompletecds|2022-10-

30|4|H5N1|USA|Brantacanadensis|USA_WY|Alphainfluenzavirus|Alphainfluenzavir usinfluenz">
<date value="2022.7753424657535" direction="forwards" units="years"/>
</taxon>
<taxon id="PQ709657.1|InfluenzaAvirus_A/Cormorant/CA/22-037516-001-original/2022_H5N1__segment4hemagglutinin_HA_genecompletecds|2022-11-06|4|H5N1|USA|Phalacrocoracidae|USA_CA|Alphainfluenzavirus|Alphainfluenzavi rusinfluenz">
<date value="2022.849315068493" direction="forwards" units="years"/>
</taxon>
<taxon id="PQ709665.1|InfluenzaAvirus_A/Cormorant/FL/22-032961-001-original/2022_H5N1__segment4hemagglutinin_HA_genecompletecds|2022-10-12|4|H5N1|USA|Phalacrocoracidae|USA_FL|Alphainfluenzavirus|Alphainfluenzavi rusinfluenz">
<date value="2022.7260273972602" direction="forwards" units="years"/>
</taxon>
<taxon id="PQ709697.1|InfluenzaAvirus_A/Double-crestedCormorant/FL/22-036552-001-original/2022_H5N1__segment4hemagglutinin_HA_genecompletecds|2022-11-03|4|H5N1|USA|Phalacrocoraxauritus|USA_FL|Alphainfluenzavirus|Alphainfluenz avirusinfluenz">
<date value="2022.841095890411" direction="forwards" units="years"/>
</taxon>
<taxon id="PQ709841.1|InfluenzaAvirus_A/Duck/ID/22-014968-005-original/2022_H5N1__segment4hemagglutinin_HA_genecompletecds|2022-05-09|4|H5N1|USA|Anatidae|USA_ID|Alphainfluenzavirus|Alphainfluenzavirusinflue nz">
<date value="2022.3534246575343" direction="forwards" units="years"/>
</taxon>
<taxon id="PQ709897.1|InfluenzaAvirus_A/Duck/UT/22-031728-004-original-repeat/2022_H5N1__segment4hemagglutinin_HA_genecompletecds|2022-10-04|4|H5N1|USA|Anatidae|USA_UT|Alphainfluenzavirus|Alphainfluenzavirusinflue nz">
<date value="2022.704109589041" direction="forwards" units="years"/>
</taxon>
<taxon id="PQ709905.1|InfluenzaAvirus_A/Duck/UT/22-031728-005-original/2022_H5N1__segment4hemagglutinin_HA_genecompletecds|2022-10-04|4|H5N1|USA|Anatidae|USA_UT|Alphainfluenzavirus|Alphainfluenzavirusinflue nz">
<date value="2022.704109589041" direction="forwards" units="years"/>
</taxon>
<taxon id="PQ709921.1|InfluenzaAvirus_A/Dunlin/WA/22-039678-002-original/2022_H5N1__segment4hemagglutinin_HA_genecompletecds|2022-11-21|4|H5N1|USA|Calidrisalpina|USA_WA|Alphainfluenzavirus|Alphainfluenzavirus influenz">
<date value="2022.890410958904" direction="forwards" units="years"/>
</taxon>
<taxon id="PQ710001.1|InfluenzaAvirus_A/Earedgrebe/UT/22-030915-001-original/2022_H5N1__segment4hemagglutinin_HA_genecompletecds|2022-09-27|4|H5N1|USA||USA_UT|Alphainfluenzavirus|Alphainfluenzavirusinfluenz">
<date value="2022.7397260273972" direction="forwards" units="years"/>
</taxon>
<taxon id="PQ710009.1|InfluenzaAvirus_A/Earedgrebe/UT/22-030915-002-original/2022_H5N1__segment4hemagglutinin_HA_genecompletecds|2022-09-27|4|H5N1|USA||USA_UT|Alphainfluenzavirus|Alphainfluenzavirusinfluenz">
<date value="2022.7397260273972" direction="forwards" units="years"/>
</taxon>
<taxon id="PQ710017.1|InfluenzaAvirus_A/Earedgrebe/UT/22-030915-003-original/2022_H5N1__segment4hemagglutinin_HA_genecompletecds|2022-09-27|4|H5N1|USA||USA_UT|Alphainfluenzavirus|Alphainfluenzavirusinfluenz">
<date value="2022.7397260273972" direction="forwards" units="years"/>

</taxon>
<taxon id="PQ710081.1|InfluenzaAvirus_A/Gadwall/AK/23-002693-002-original/2022_H5N1__segment4hemagglutinin_HA_genecompletecds|2022-09-20|4|H5N1|USA|Marecastrepera|USA_AK|Alphainfluenzavirus|Alphainfluenzavirus influenz">
<date value="2022.7205479452055" direction="forwards" units="years"/>
</taxon>
<taxon id="PQ710089.1|InfluenzaAvirus_A/Gadwall/AK/23-002693-003-original/2022_H5N1__segment4hemagglutinin_HA_genecompletecds|2022-09-20|4|H5N1|USA|Marecastrepera|USA_AK|Alphainfluenzavirus|Alphainfluenzavirus influenz">
<date value="2022.7205479452055" direction="forwards" units="years"/>
</taxon>
<taxon id="PQ710209.1|InfluenzaAvirus_A/Gadwall/LA/22-037240-002-original-repeat/2022_H5N1__segment4hemagglutinin_HA_genecompletecds|2022-11-13|4|H5N1|USA|Marecastrepera|USA_LA|Alphainfluenzavirus|Alphainfluenzavirus influenz">
<date value="2022.868493150685" direction="forwards" units="years"/>
</taxon>
<taxon id="PQ710337.1|InfluenzaAvirus_A/Gadwall/TX/22-036753-004-original-repeat/2022_H5N1__segment4hemagglutinin_HA_genecompletecds|2022-11-05|4|H5N1|USA|Marecastrepera|USA_TX|Alphainfluenzavirus|Alphainfluenzavirus influenz">
<date value="2022.8465753424657" direction="forwards" units="years"/>
</taxon>
<taxon id="PQ710345.1|InfluenzaAvirus_A/Gadwall/TX/22-036753-021-original-repeat/2022_H5N1__segment4hemagglutinin_HA_genecompletecds|2022-11-05|4|H5N1|USA|Marecastrepera|USA_TX|Alphainfluenzavirus|Alphainfluenzavirus influenz">
<date value="2022.8465753424657" direction="forwards" units="years"/>
</taxon>
<taxon id="PQ710353.1|InfluenzaAvirus_A/Gadwall/TX/22-036753-040-original-repeat/2022_H5N1__segment4hemagglutinin_HA_genecompletecds|2022-11-05|4|H5N1|USA|Marecastrepera|USA_TX|Alphainfluenzavirus|Alphainfluenzavirus influenz">
<date value="2022.8465753424657" direction="forwards" units="years"/>
</taxon>
<taxon id="PQ710361.1|InfluenzaAvirus_A/Gadwall/TX/22-036753-054-original-repeat/2022_H5N1__segment4hemagglutinin_HA_genecompletecds|2022-11-05|4|H5N1|USA|Marecastrepera|USA_TX|Alphainfluenzavirus|Alphainfluenzavirus influenz">
<date value="2022.8465753424657" direction="forwards" units="years"/>
</taxon>
<taxon id="PQ710369.1|InfluenzaAvirus_A/Gadwall/TX/22-036753-074-original/2022_H5N1__segment4hemagglutinin_HA_genecompletecds|2022-11-05|4|H5N1|USA|Marecastrepera|USA_TX|Alphainfluenzavirus|Alphainfluenzavirus influenz">
<date value="2022.8465753424657" direction="forwards" units="years"/>
</taxon>
<taxon id="PQ710377.1|InfluenzaAvirus_A/Gadwall/TX/22-036753-075-original-repeat/2022_H5N1__segment4hemagglutinin_HA_genecompletecds|2022-11-05|4|H5N1|USA|Marecastrepera|USA_TX|Alphainfluenzavirus|Alphainfluenzavirus influenz">
<date value="2022.8465753424657" direction="forwards" units="years"/>
</taxon>
<taxon id="PQ710385.1|InfluenzaAvirus_A/Gadwall/TX/22-036753-082-original-repeat/2022_H5N1__segment4hemagglutinin_HA_genecompletecds|2022-11-05|4|H5N1|USA|Marecastrepera|USA_TX|Alphainfluenzavirus|Alphainfluenzavirus influenz">
<date value="2022.8465753424657" direction="forwards" units="years"/>
</taxon>

<taxon id="PQ710393.1|InfluenzaAvirus_A/Gadwall/TX/22-036754-036-original/2022_H5N1__segment4hemagglutinin_HA_genecompletecds|2022-11-06|4|H5N1|USA|Marecastrepera|USA_TX|Alphainfluenzavirus|Alphainfluenzavirus influenz">
<date value="2022.849315068493" direction="forwards" units="years"/>
</taxon>
<taxon id="PQ710401.1|InfluenzaAvirus_A/Gadwall/TX/22-036754-046-original/2022_H5N1__segment4hemagglutinin_HA_genecompletecds|2022-11-06|4|H5N1|USA|Marecastrepera|USA_TX|Alphainfluenzavirus|Alphainfluenzavirus influenz">
<date value="2022.849315068493" direction="forwards" units="years"/>
</taxon>
<taxon id="PQ710409.1|InfluenzaAvirus_A/Gadwall/TX/22-036754-057-original/2022_H5N1__segment4hemagglutinin_HA_genecompletecds|2022-11-06|4|H5N1|USA|Marecastrepera|USA_TX|Alphainfluenzavirus|Alphainfluenzavirus influenz">
<date value="2022.849315068493" direction="forwards" units="years"/>
</taxon>
<taxon id="PQ710417.1|InfluenzaAvirus_A/Gadwall/TX/22-036754-066-original/2022_H5N1__segment4hemagglutinin_HA_genecompletecds|2022-11-06|4|H5N1|USA|Marecastrepera|USA_TX|Alphainfluenzavirus|Alphainfluenzavirus influenz">
<date value="2022.849315068493" direction="forwards" units="years"/>
</taxon>
<taxon id="PQ710465.1|InfluenzaAvirus_A/GlaucousGull/AK/22-017953-003-original/2022_H5N1__segment4hemagglutinin_HA_genecompletecds|2022-06-06|4|H5N1|USA|Larushyperboreus|USA_AK|Alphainfluenzavirus|Alphainfluenzavirusinfluenz">
<date value="2022.4301369863015" direction="forwards" units="years"/>
</taxon>
<taxon id="PQ710473.1|InfluenzaAvirus_A/GlaucousGull/AK/22-017953-004-original/2022_H5N1__segment4hemagglutinin_HA_genecompletecds|2022-06-06|4|H5N1|USA|Larushyperboreus|USA_AK|Alphainfluenzavirus|Alphainfluenzavirusinfluenz">
<date value="2022.4301369863015" direction="forwards" units="years"/>
</taxon>
<taxon id="PQ710481.1|InfluenzaAvirus_A/GlaucousGull/AK/22-017953-009-original/2022_H5N1__segment4hemagglutinin_HA_genecompletecds|2022-06-06|4|H5N1|USA|Larushyperboreus|USA_AK|Alphainfluenzavirus|Alphainfluenzavirusinfluenz">
<date value="2022.4301369863015" direction="forwards" units="years"/>
</taxon>
<taxon id="PQ710489.1|InfluenzaAvirus_A/GlaucousGull/AK/22-017953-010-original/2022_H5N1__segment4hemagglutinin_HA_genecompletecds|2022-06-06|4|H5N1|USA|Larushyperboreus|USA_AK|Alphainfluenzavirus|Alphainfluenzavirusinfluenz">
<date value="2022.4301369863015" direction="forwards" units="years"/>
</taxon>
<taxon id="PQ710497.1|InfluenzaAvirus_A/GlaucousGull/AK/22-018101-002-original/2022_H5N1__segment4hemagglutinin_HA_genecompletecds|2022-06-06|4|H5N1|USA|Larushyperboreus|USA_AK|Alphainfluenzavirus|Alphainfluenzavirusinfluenz">
<date value="2022.4301369863015" direction="forwards" units="years"/>
</taxon>
<taxon id="PQ710505.1|InfluenzaAvirus_A/GlaucousGull/AK/22-021855-001-original/2022_H5N1__segment4hemagglutinin_HA_genecompletecds|2022-05-31|4|H5N1|USA|Larushyperboreus|USA_AK|Alphainfluenzavirus|Alphainfluenzavirusinfluenz">
<date value="2022.4136986301369" direction="forwards" units="years"/>
</taxon>

<taxon id="PQ710529.1|InfluenzaAvirus_A/GlaucousGull/AK/22-024502-001-original/2022_H5N1__segment4hemagglutinin_HA_genecompletecds|2022-07-01|4|H5N1|USA|Larushyperboreus|USA_AK|Alphainfluenzavirus|Alphainfluenzavirusinfluenz">
<date value="2022.4986301369863" direction="forwards" units="years"/>
</taxon>
<taxon id="PQ710801.1|InfluenzaAvirus_A/Greathornedowl/CA/22-035595-002-original/2022_H5N1__segment4hemagglutinin_HA_genecompletecds|2022-10-27|4|H5N1|USA|Bubovirginianus|USA_CA|Alphainfluenzavirus|Alphainfluenzavirusinfluenz">
<date value="2022.7671232876712" direction="forwards" units="years"/>
</taxon>
<taxon id="PQ710897.1|InfluenzaAvirus_A/Greathornedowl/CO/22-033694-001-original/2022_H5N1__segment4hemagglutinin_HA_genecompletecds|2022-10-18|4|H5N1|USA|Bubovirginianus|USA_CO|Alphainfluenzavirus|Alphainfluenzavirusinfluenz">
<date value="2022.7424657534248" direction="forwards" units="years"/>
</taxon>
<taxon id="PQ710913.1|InfluenzaAvirus_A/Greathornedowl/CO/22-038652-003-original/2022_H5N1__segment4hemagglutinin_HA_genecompletecds|2022-11-22|4|H5N1|USA|Bubovirginianus|USA_CO|Alphainfluenzavirus|Alphainfluenzavirusinfluenz">
<date value="2022.8931506849315" direction="forwards" units="years"/>
</taxon>
<taxon id="PQ711169.1|InfluenzaAvirus_A/Greathornedowl/KS/22-040466-001-original/2022_H5N1__segment4hemagglutinin_HA_genecompletecds|2022-11-29|4|H5N1|USA|Bubovirginianus|USA_KS|Alphainfluenzavirus|Alphainfluenzavirusinfluenz">
<date value="2022.9123287671232" direction="forwards" units="years"/>
</taxon>
<taxon id="PQ711673.1|InfluenzaAvirus_A/Greathornedowl/ND/22-032264-001-original/2022_H5N1__segment4hemagglutinin_HA_genecompletecds|2022-09-28|4|H5N1|USA|Bubovirginianus|USA_ND|Alphainfluenzavirus|Alphainfluenzavirusinfluenz">
<date value="2022.7424657534248" direction="forwards" units="years"/>
</taxon>
<taxon id="PQ711681.1|InfluenzaAvirus_A/Greathornedowl/NE/22-038800-001-original/2022_H5N1__segment4hemagglutinin_HA_genecompletecds|2022-11-30|4|H5N1|USA|Bubovirginianus|USA_NE|Alphainfluenzavirus|Alphainfluenzavirusinfluenz">
<date value="2022.9150684931508" direction="forwards" units="years"/>
</taxon>
<taxon id="PQ711745.1|InfluenzaAvirus_A/Greathornedowl/OR/22-033001-001-original/2022_H5N1__segment4hemagglutinin_HA_genecompletecds|2022-10-11|4|H5N1|USA|Bubovirginianus|USA_OR|Alphainfluenzavirus|Alphainfluenzavirusinfluenz">
<date value="2022.7232876712328" direction="forwards" units="years"/>
</taxon>
<taxon id="PQ711849.1|InfluenzaAvirus_A/Greathornedowl/UT/22-032952-008-original/2022_H5N1__segment4hemagglutinin_HA_genecompletecds|2022-10-13|4|H5N1|USA|Bubovirginianus|USA_UT|Alphainfluenzavirus|Alphainfluenzavirusinfluenz">
<date value="2022.7287671232878" direction="forwards" units="years"/>
</taxon>
<taxon id="PQ711857.1|InfluenzaAvirus_A/Greathornedowl/UT/22-035956-007-original/2022_H5N1__segment4hemagglutinin_HA_genecompletecds|2022-10-25|4|H5N1|USA|Bubovirginianus|USA_UT|Alphainfluenzavirus|Alphainfluenzavirusinfluenz">
<date value="2022.7616438356165" direction="forwards" units="years"/>
</taxon>

<taxon id="PQ711873.1|InfluenzaAvirus_A/Greathornedowl/WA/22-015526-004-original/2022_H5N1__segment4hemagglutinin_HA_genecompletecds|2022-05-09|4|H5N1|USA|Bubovirginianus|USA_WA|Alphainfluenzavirus|Alphainfluenzavirusinfluenz">
<date value="2022.3534246575343" direction="forwards" units="years"/>
</taxon>
<taxon id="PQ711889.1|InfluenzaAvirus_A/Greathornedowl/WA/22-038418-001-original/2022_H5N1__segment4hemagglutinin_HA_genecompletecds|2022-11-15|4|H5N1|USA|Bubovirginianus|USA_WA|Alphainfluenzavirus|Alphainfluenzavirusinfluenz">
<date value="2022.8739726027397" direction="forwards" units="years"/>
</taxon>
<taxon id="PQ712073.1|InfluenzaAvirus_A/Greathornedowl/WY/22-029194-001-original/2022_H5N1__segment4hemagglutinin_HA_genecompletecds|2022-09-11|4|H5N1|USA|Bubovirginianus|USA_WY|Alphainfluenzavirus|Alphainfluenzavirusinfluenz">
<date value="2022.695890410959" direction="forwards" units="years"/>
</taxon>
<taxon id="PQ712081.1|InfluenzaAvirus_A/Greathornedowl/WY/22-030747-001-original/2022_H5N1__segment4hemagglutinin_HA_genecompletecds|2022-09-16|4|H5N1|USA|Bubovirginianus|USA_WY|Alphainfluenzavirus|Alphainfluenzavirusinfluenz">
<date value="2022.7095890410958" direction="forwards" units="years"/>
</taxon>
<taxon id="PQ712097.1|InfluenzaAvirus_A/Greathornedowl/WY/22-031607-001-original/2022_H5N1__segment4hemagglutinin_HA_genecompletecds|2022-09-29|4|H5N1|USA|Bubovirginianus|USA_WY|Alphainfluenzavirus|Alphainfluenzavirusinfluenz">
<date value="2022.7452054794521" direction="forwards" units="years"/>
</taxon>
<taxon id="PQ712105.1|InfluenzaAvirus_A/Greathornedowl/WY/22-032604-001-original/2022_H5N1__segment4hemagglutinin_HA_genecompletecds|2022-10-11|4|H5N1|USA|Bubovirginianus|USA_WY|Alphainfluenzavirus|Alphainfluenzavirusinfluenz">
<date value="2022.7232876712328" direction="forwards" units="years"/>
</taxon>
<taxon id="PQ712113.1|InfluenzaAvirus_A/Greathornedowl/WY/22-033035-001-original/2022_H5N1__segment4hemagglutinin_HA_genecompletecds|2022-10-12|4|H5N1|USA|Bubovirginianus|USA_WY|Alphainfluenzavirus|Alphainfluenzavirusinfluenz">
<date value="2022.7260273972602" direction="forwards" units="years"/>
</taxon>
<taxon id="PQ712121.1|InfluenzaAvirus_A/Greathornedowl/WY/22-033036-002-original/2022_H5N1__segment4hemagglutinin_HA_genecompletecds|2022-10-12|4|H5N1|USA|Bubovirginianus|USA_WY|Alphainfluenzavirus|Alphainfluenzavirusinfluenz">
<date value="2022.7260273972602" direction="forwards" units="years"/>
</taxon>
<taxon id="PQ712249.1|InfluenzaAvirus_A/Gull/AK/22-017953-006-original/2022_H5N1__segment4hemagglutinin_HA_genecompletecds|2022-06-06|4|H5N1|USA|Laridae|USA_AK|Alphainfluenzavirus|Alphainfluenzavirusinfluenz">
<date value="2022.4301369863015" direction="forwards" units="years"/>
</taxon>
<taxon id="PQ712257.1|InfluenzaAvirus_A/Gull/AK/22-017953-007-original/2022_H5N1__segment4hemagglutinin_HA_genecompletecds|2022-06-06|4|H5N1|USA|Laridae|USA_AK|Alphainfluenzavirus|Alphainfluenzavirusinfluenz">
<date value="2022.4301369863015" direction="forwards" units="years"/>
</taxon>

<taxon id="PQ712361.1|InfluenzaAvirus_A/Gull/UT/22-031729-002-original-repeat/2022_H5N1__segment4hemagglutinin_HA_genecompletecds|2022-09-29|4|H5N1|USA|Laridae|USA_UT|Alphainfluenzavirus|Alphainfluenzavirusinfluenz">
<date value="2022.7452054794521" direction="forwards" units="years"/>
</taxon>
<taxon id="PQ712369.1|InfluenzaAvirus_A/Gull/UT/22-031729-007-original/2022_H5N1__segment4hemagglutinin_HA_genecompletecds|2022-09-29|4|H5N1|USA|Laridae|USA_UT|Alphainfluenzavirus|Alphainfluenzavirusinfluenz">
<date value="2022.7452054794521" direction="forwards" units="years"/>
</taxon>
<taxon id="PQ712393.1|InfluenzaAvirus_A/Hawk/ID/22-017864-001-original/2022_H5N1__segment4hemagglutinin_HA_genecompletecds|2022-06-06|4|H5N1|USA|Accipitridae|USA_ID|Alphainfluenzavirus|Alphainfluenzavirusinfluenz">
<date value="2022.4301369863015" direction="forwards" units="years"/>
</taxon>
<taxon id="PQ712537.1|InfluenzaAvirus_A/Hawk/WY/23-000336-001-original/2022_H5N1__segment4hemagglutinin_HA_genecompletecds|2022-10-30|4|H5N1|USA|Accipitridae|USA_WY|Alphainfluenzavirus|Alphainfluenzavirusinfluenz">
<date value="2022.7753424657535" direction="forwards" units="years"/>
</taxon>
<taxon id="PQ712929.1|InfluenzaAvirus_A/Mallard/AK/22-029101-001-original/2022_H5N1__segment4hemagglutinin_HA_genecompletecds|2022-09-05|4|H5N1|USA|Anasplatyrhynchos|USA_AK|Alphainfluenzavirus|Alphainfluenzavirusinfluenz">
<date value="2022.6794520547944" direction="forwards" units="years"/>
</taxon>
<taxon id="PQ712937.1|InfluenzaAvirus_A/Mallard/AK/22-031400-001-original-repeat/2022_H5N1__segment4hemagglutinin_HA_genecompletecds|2022-09-03|4|H5N1|USA|Anasplatyrhynchos|USA_AK|Alphainfluenzavirus|Alphainfluenzavirusinfluenz">
<date value="2022.6739726027397" direction="forwards" units="years"/>
</taxon>
<taxon id="PQ712945.1|InfluenzaAvirus_A/Mallard/AK/22-036139-015-original/2022_H5N1__segment4hemagglutinin_HA_genecompletecds|2022-11-02|4|H5N1|USA|Anasplatyrhynchos|USA_AK|Alphainfluenzavirus|Alphainfluenzavirusinfluenz">
<date value="2022.8383561643836" direction="forwards" units="years"/>
</taxon>
<taxon id="PQ712953.1|InfluenzaAvirus_A/Mallard/AK/22-036139-029-original/2022_H5N1__segment4hemagglutinin_HA_genecompletecds|2022-11-02|4|H5N1|USA|Anasplatyrhynchos|USA_AK|Alphainfluenzavirus|Alphainfluenzavirusinfluenz">
<date value="2022.8383561643836" direction="forwards" units="years"/>
</taxon>
<taxon id="PQ712969.1|InfluenzaAvirus_A/Mallard/AK/22-036139-032-original/2022_H5N1__segment4hemagglutinin_HA_genecompletecds|2022-11-02|4|H5N1|USA|Anasplatyrhynchos|USA_AK|Alphainfluenzavirus|Alphainfluenzavirusinfluenz">
<date value="2022.8383561643836" direction="forwards" units="years"/>
</taxon>
<taxon id="PQ712977.1|InfluenzaAvirus_A/Mallard/AK/22-036139-037-original/2022_H5N1__segment4hemagglutinin_HA_genecompletecds|2022-11-02|4|H5N1|USA|Anasplatyrhynchos|USA_AK|Alphainfluenzavirus|Alphainfluenzavirusinfluenz">
<date value="2022.8383561643836" direction="forwards" units="years"/>
</taxon>

<taxon id="PQ712985.1|InfluenzaAvirus_A/Mallard/AK/22-036139-058-original/2022_H5N1__segment4hemagglutinin_HA_genecompletecds|2022-11-02|4|H5N1|USA|Anasplatyrhynchos|USA_AK|Alphainfluenzavirus|Alphainfluenzavirusinfluenz">
<date value="2022.8383561643836" direction="forwards" units="years"/>
</taxon>
<taxon id="PQ713001.1|InfluenzaAvirus_A/Mallard/AK/22-036139-062-original/2022_H5N1__segment4hemagglutinin_HA_genecompletecds|2022-11-02|4|H5N1|USA|Anasplatyrhynchos|USA_AK|Alphainfluenzavirus|Alphainfluenzavirusinfluenz">
<date value="2022.8383561643836" direction="forwards" units="years"/>
</taxon>
<taxon id="PQ713009.1|InfluenzaAvirus_A/Mallard/AK/22-036139-063-original/2022_H5N1__segment4hemagglutinin_HA_genecompletecds|2022-11-02|4|H5N1|USA|Anasplatyrhynchos|USA_AK|Alphainfluenzavirus|Alphainfluenzavirusinfluenz">
<date value="2022.8383561643836" direction="forwards" units="years"/>
</taxon>
<taxon id="PQ713017.1|InfluenzaAvirus_A/Mallard/AK/22-036139-081-original/2022_H5N1__segment4hemagglutinin_HA_genecompletecds|2022-11-02|4|H5N1|USA|Anasplatyrhynchos|USA_AK|Alphainfluenzavirus|Alphainfluenzavirusinfluenz">
<date value="2022.8383561643836" direction="forwards" units="years"/>
</taxon>
<taxon id="PQ713025.1|InfluenzaAvirus_A/Mallard/AK/22-036139-092-original/2022_H5N1__segment4hemagglutinin_HA_genecompletecds|2022-11-02|4|H5N1|USA|Anasplatyrhynchos|USA_AK|Alphainfluenzavirus|Alphainfluenzavirusinfluenz">
<date value="2022.8383561643836" direction="forwards" units="years"/>
</taxon>
<taxon id="PQ713033.1|InfluenzaAvirus_A/Mallard/AK/23-002693-020-original/2022_H5N1__segment4hemagglutinin_HA_genecompletecds|2022-09-30|4|H5N1|USA|Anasplatyrhynchos|USA_AK|Alphainfluenzavirus|Alphainfluenzavirusinfluenz">
<date value="2022.7479452054795" direction="forwards" units="years"/>
</taxon>
<taxon id="PQ713041.1|InfluenzaAvirus_A/Mallard/AK/23-002693-024-original/2022_H5N1__segment4hemagglutinin_HA_genecompletecds|2022-09-30|4|H5N1|USA|Anasplatyrhynchos|USA_AK|Alphainfluenzavirus|Alphainfluenzavirusinfluenz">
<date value="2022.7479452054795" direction="forwards" units="years"/>
</taxon>
<taxon id="PQ713049.1|InfluenzaAvirus_A/Mallard/AK/23-003143-001-original/2022_H5N1__segment4hemagglutinin_HA_genecompletecds|2022-09-30|4|H5N1|USA|Anasplatyrhynchos|USA_AK|Alphainfluenzavirus|Alphainfluenzavirusinfluenz">
<date value="2022.7479452054795" direction="forwards" units="years"/>
</taxon>
<taxon id="PQ713057.1|InfluenzaAvirus_A/Mallard/AK/23-003641-001-original/2022_H5N1__segment4hemagglutinin_HA_genecompletecds|2022-10-09|4|H5N1|USA|Anasplatyrhynchos|USA_AK|Alphainfluenzavirus|Alphainfluenzavirusinfluenz">
<date value="2022.717808219178" direction="forwards" units="years"/>
</taxon>
<taxon id="PQ713065.1|InfluenzaAvirus_A/Mallard/AK/23-003641-008-original/2022_H5N1__segment4hemagglutinin_HA_genecompletecds|2022-10-14|4|H5N1|USA|Anasplatyrhynchos|USA_AK|Alphainfluenzavirus|Alphainfluenzavirusinfluenz">
<date value="2022.731506849315" direction="forwards" units="years"/>
</taxon>

<taxon id="PQ713129.1|InfluenzaAvirus_A/Mallard/CO/22-033430-002-original/2022_H5N1__segment4hemagglutinin_HA_genecompletecds|2022-10-09|4|H5N1|USA|Anasplatyrhynchos|USA_CO|Alphainfluenzavirus|Alphainfluenzavirusinfluenz">
<date value="2022.717808219178" direction="forwards" units="years"/>
</taxon>
<taxon id="PQ713273.1|InfluenzaAvirus_A/Mallard/IN/22-034829-007-original-repeat2/2022_H5N1__segment4hemagglutinin_HA_genecompletecds|2022-10-28|4|H5N1|USA|Anasplatyrhynchos|USA_IN|Alphainfluenzavirus|Alphainfluenzavirusinfluenz">
<date value="2022.7698630136986" direction="forwards" units="years"/>
</taxon>
<taxon id="PQ713505.1|InfluenzaAvirus_A/Mallard/MN/22-033478-004-original/2022_H5N1__segment4hemagglutinin_HA_genecompletecds|2022-09-13|4|H5N1|USA|Anasplatyrhynchos|USA_MN|Alphainfluenzavirus|Alphainfluenzavirusinfluenz">
<date value="2022.7013698630137" direction="forwards" units="years"/>
</taxon>
<taxon id="PQ713929.1|InfluenzaAvirus_A/Mallard/OR/22-036880-048-original/2022_H5N1__segment4hemagglutinin_HA_genecompletecds|2022-11-07|4|H5N1|USA|Anasplatyrhynchos|USA_OR|Alphainfluenzavirus|Alphainfluenzavirusinfluenz">
<date value="2022.8520547945207" direction="forwards" units="years"/>
</taxon>
<taxon id="PQ714161.1|InfluenzaAvirus_A/Mallard/WA/22-039095-002-original/2022_H5N1__segment4hemagglutinin_HA_genecompletecds|2022-11-25|4|H5N1|USA|Anasplatyrhynchos|USA_WA|Alphainfluenzavirus|Alphainfluenzavirusinfluenz">
<date value="2022.9013698630138" direction="forwards" units="years"/>
</taxon>
<taxon id="PQ714241.1|InfluenzaAvirus_A/MuscovyDuck/CA/22-034331-001-original-repeat/2022_H5N1__segment4hemagglutinin_HA_genecompletecds|2022-10-20|4|H5N1|USA|Cairinamoschata|USA_CA|Alphainfluenzavirus|Alphainfluenzavirusinfluenz">
<date value="2022.7479452054795" direction="forwards" units="years"/>
</taxon>
<taxon id="PQ714249.1|InfluenzaAvirus_A/MuscovyDuck/CA/22-034331-003-original-repeat/2022_H5N1__segment4hemagglutinin_HA_genecompletecds|2022-10-20|4|H5N1|USA|Cairinamoschata|USA_CA|Alphainfluenzavirus|Alphainfluenzavirusinfluenz">
<date value="2022.7479452054795" direction="forwards" units="years"/>
</taxon>
<taxon id="PQ714257.1|InfluenzaAvirus_A/MuscovyDuck/CA/22-034331-004-original-repeat/2022_H5N1__segment4hemagglutinin_HA_genecompletecds|2022-10-20|4|H5N1|USA|Cairinamoschata|USA_CA|Alphainfluenzavirus|Alphainfluenzavirusinfluenz">
<date value="2022.7479452054795" direction="forwards" units="years"/>
</taxon>
<taxon id="PQ714313.1|InfluenzaAvirus_A/MuscovyDuck/FL/22-036966-002-original/2022_H5N1__segment4hemagglutinin_HA_genecompletecds|2022-11-04|4|H5N1|USA|Cairinamoschata|USA_FL|Alphainfluenzavirus|Alphainfluenzavirusinfluenz">
<date value="2022.8438356164384" direction="forwards" units="years"/>
</taxon>
<taxon id="PQ714457.1|InfluenzaAvirus_A/Northernpintail/AK/22-029100-001-original/2022_H5N1__segment4hemagglutinin_HA_genecompletecds|2022-09-04|4|H5N1|USA|Anasacuta|USA_AK|Alphainfluenzavirus|Alphainfluenzavirusinfluenz">

<date value="2022.676712328767" direction="forwards" units="years"/>
</taxon>
<taxon id="PQ714481.1|InfluenzaAvirus_A/Northernpintail/AK/22-039438-001-original/2022_H5N1__segment4hemagglutinin_HA_genecompletecds|2022-09-02|4|H5N1|USA|Anasacuta|USA_AK|Alphainfluenzavirus|Alphainfluenzavirusinfluenz">
<date value="2022.6712328767123" direction="forwards" units="years"/>
</taxon>
<taxon id="PQ714489.1|InfluenzaAvirus_A/Northernpintail/AK/22-039438-005-original/2022_H5N1__segment4hemagglutinin_HA_genecompletecds|2022-09-07|4|H5N1|USA|Anasacuta|USA_AK|Alphainfluenzavirus|Alphainfluenzavirusinfluenz">
<date value="2022.6849315068494" direction="forwards" units="years"/>
</taxon>
<taxon id="PQ714497.1|InfluenzaAvirus_A/Northernpintail/AK/22-039438-008-original/2022_H5N1__segment4hemagglutinin_HA_genecompletecds|2022-09-09|4|H5N1|USA|Anasacuta|USA_AK|Alphainfluenzavirus|Alphainfluenzavirusinfluenz">
<date value="2022.690410958904" direction="forwards" units="years"/>
</taxon>
<taxon id="PQ714505.1|InfluenzaAvirus_A/Northernpintail/AK/22-039438-009-original/2022_H5N1__segment4hemagglutinin_HA_genecompletecds|2022-09-09|4|H5N1|USA|Anasacuta|USA_AK|Alphainfluenzavirus|Alphainfluenzavirusinfluenz">
<date value="2022.690410958904" direction="forwards" units="years"/>
</taxon>
<taxon id="PQ714513.1|InfluenzaAvirus_A/Northernpintail/AK/22-039438-010-original/2022_H5N1__segment4hemagglutinin_HA_genecompletecds|2022-09-09|4|H5N1|USA|Anasacuta|USA_AK|Alphainfluenzavirus|Alphainfluenzavirusinfluenz">
<date value="2022.690410958904" direction="forwards" units="years"/>
</taxon>
<taxon id="PQ714521.1|InfluenzaAvirus_A/Northernpintail/AK/22-039438-011-original/2022_H5N1__segment4hemagglutinin_HA_genecompletecds|2022-09-09|4|H5N1|USA|Anasacuta|USA_AK|Alphainfluenzavirus|Alphainfluenzavirusinfluenz">
<date value="2022.690410958904" direction="forwards" units="years"/>
</taxon>
<taxon id="PQ714529.1|InfluenzaAvirus_A/Northernpintail/AK/22-039438-013-original/2022_H5N1__segment4hemagglutinin_HA_genecompletecds|2022-09-12|4|H5N1|USA|Anasacuta|USA_AK|Alphainfluenzavirus|Alphainfluenzavirusinfluenz">
<date value="2022.6986301369864" direction="forwards" units="years"/>
</taxon>
<taxon id="PQ714537.1|InfluenzaAvirus_A/Northernpintail/AK/22-039438-014-original/2022_H5N1__segment4hemagglutinin_HA_genecompletecds|2022-09-12|4|H5N1|USA|Anasacuta|USA_AK|Alphainfluenzavirus|Alphainfluenzavirusinfluenz">
<date value="2022.6986301369864" direction="forwards" units="years"/>
</taxon>
<taxon id="PQ714545.1|InfluenzaAvirus_A/Northernpintail/AK/22-039438-017-original/2022_H5N1__segment4hemagglutinin_HA_genecompletecds|2022-09-14|4|H5N1|USA|Anasacuta|USA_AK|Alphainfluenzavirus|Alphainfluenzavirusinfluenz">
<date value="2022.704109589041" direction="forwards" units="years"/>
</taxon>
<taxon id="PQ714553.1|InfluenzaAvirus_A/Northernpintail/AK/22-039438-018-original/2022_H5N1__segment4hemagglutinin_HA_genecompletecds|2022-09-14|4|H5N1|USA|Anasacuta|USA_AK|Alphainfluenzavirus|Alphainfluenzavirusinfluenz">
<date value="2022.704109589041" direction="forwards" units="years"/>

</taxon>
<taxon id="PQ714577.1|InfluenzaAvirus_A/Northernpintail/AK/23-002693-006-original/2022_H5N1__segment4hemagglutinin_HA_genecompletecds|2022-09-28|4|H5N1|USA|Anasacuta|USA_AK|Alphainfluenzavirus|Alphainfluenzavirusinfluenz">
<date value="2022.7424657534248" direction="forwards" units="years"/>
</taxon>
<taxon id="PQ714585.1|InfluenzaAvirus_A/Northernpintail/AK/23-002693-007-original/2022_H5N1__segment4hemagglutinin_HA_genecompletecds|2022-09-28|4|H5N1|USA|Anasacuta|USA_AK|Alphainfluenzavirus|Alphainfluenzavirusinfluenz">
<date value="2022.7424657534248" direction="forwards" units="years"/>
</taxon>
<taxon id="PQ714601.1|InfluenzaAvirus_A/Northernpintail/AK/23-002693-012-original/2022_H5N1__segment4hemagglutinin_HA_genecompletecds|2022-09-28|4|H5N1|USA|Anasacuta|USA_AK|Alphainfluenzavirus|Alphainfluenzavirusinfluenz">
<date value="2022.7424657534248" direction="forwards" units="years"/>
</taxon>
<taxon id="PQ714617.1|InfluenzaAvirus_A/Northernpintail/AK/23-002693-014-original/2022_H5N1__segment4hemagglutinin_HA_genecompletecds|2022-09-30|4|H5N1|USA|Anasacuta|USA_AK|Alphainfluenzavirus|Alphainfluenzavirusinfluenz">
<date value="2022.7479452054795" direction="forwards" units="years"/>
</taxon>
<taxon id="PQ714625.1|InfluenzaAvirus_A/Northernpintail/AK/23-002693-015-original/2022_H5N1__segment4hemagglutinin_HA_genecompletecds|2022-09-30|4|H5N1|USA|Anasacuta|USA_AK|Alphainfluenzavirus|Alphainfluenzavirusinfluenz">
<date value="2022.7479452054795" direction="forwards" units="years"/>
</taxon>
<taxon id="PQ714633.1|InfluenzaAvirus_A/Northernpintail/AK/23-002693-016-original/2022_H5N1__segment4hemagglutinin_HA_genecompletecds|2022-09-30|4|H5N1|USA|Anasacuta|USA_AK|Alphainfluenzavirus|Alphainfluenzavirusinfluenz">
<date value="2022.7479452054795" direction="forwards" units="years"/>
</taxon>
<taxon id="PQ714641.1|InfluenzaAvirus_A/Northernpintail/AK/23-002693-017-original/2022_H5N1__segment4hemagglutinin_HA_genecompletecds|2022-09-30|4|H5N1|USA|Anasacuta|USA_AK|Alphainfluenzavirus|Alphainfluenzavirusinfluenz">
<date value="2022.7479452054795" direction="forwards" units="years"/>
</taxon>
<taxon id="PQ714649.1|InfluenzaAvirus_A/Northernpintail/AK/23-002693-018-original/2022_H5N1__segment4hemagglutinin_HA_genecompletecds|2022-09-30|4|H5N1|USA|Anasacuta|USA_AK|Alphainfluenzavirus|Alphainfluenzavirusinfluenz">
<date value="2022.7479452054795" direction="forwards" units="years"/>
</taxon>
<taxon id="PQ714657.1|InfluenzaAvirus_A/Northernpintail/AK/23-002693-021-original/2022_H5N1__segment4hemagglutinin_HA_genecompletecds|2022-09-30|4|H5N1|USA|Anasacuta|USA_AK|Alphainfluenzavirus|Alphainfluenzavirusinfluenz">
<date value="2022.7479452054795" direction="forwards" units="years"/>
</taxon>
<taxon id="PQ714697.1|InfluenzaAvirus_A/Northernpintail/AK/23-002693-030-original/2022_H5N1__segment4hemagglutinin_HA_genecompletecds|2022-10-02|4|H5N1|USA|Anasacuta|USA_AK|Alphainfluenzavirus|Alphainfluenzavirusinfluenz">
<date value="2022.6986301369864" direction="forwards" units="years"/>
</taxon>

<taxon id="PQ714705.1|InfluenzaAvirus_A/Northernpintail/LA/22-039392-003-original-repeat/2022_H5N1__segment4hemagglutinin_HA_genecompletecds|2022-11-28|4|H5N1|USA|Anasacuta|USA_LA|Alphainfluenzavirus|Alphainfluenzavirusinfluenz">
<date value="2022.9095890410958" direction="forwards" units="years"/>
</taxon>
<taxon id="PQ714713.1|InfluenzaAvirus_A/Northernpintail/OR/22-034677-048-original-repeat2/2022_H5N1__segment4hemagglutinin_HA_genecompletecds|2022-10-08|4|H5N1|USA|Anasacuta|USA_OR|Alphainfluenzavirus|Alphainfluenzavirusinfluenz">
<date value="2022.7150684931507" direction="forwards" units="years"/>
</taxon>
<taxon id="PQ714737.1|InfluenzaAvirus_A/Northernshoveler/CA/23-005876-020-original/2022_H5N1__segment4hemagglutinin_HA_genecompletecds|2022-10-23|4|H5N1|USA|Spatulaclypeata|USA_CA|Alphainfluenzavirus|Alphainfluenzavirusinfluenz">
<date value="2022.7561643835616" direction="forwards" units="years"/>
</taxon>
<taxon id="PQ714745.1|InfluenzaAvirus_A/Northernshoveler/CA/23-005876-026-original/2022_H5N1__segment4hemagglutinin_HA_genecompletecds|2022-10-23|4|H5N1|USA|Spatulaclypeata|USA_CA|Alphainfluenzavirus|Alphainfluenzavirusinfluenz">
<date value="2022.7561643835616" direction="forwards" units="years"/>
</taxon>
<taxon id="PQ714761.1|InfluenzaAvirus_A/Northernshoveler/LA/22-037236-004-original/2022_H5N1__segment4hemagglutinin_HA_genecompletecds|2022-11-12|4|H5N1|USA|Spatulaclypeata|USA_LA|Alphainfluenzavirus|Alphainfluenzavirusinfluenz">
<date value="2022.8657534246574" direction="forwards" units="years"/>
</taxon>
<taxon id="PQ714777.1|InfluenzaAvirus_A/Northernshoveler/NV/22-037517-003-original/2022_H5N1__segment4hemagglutinin_HA_genecompletecds|2022-11-05|4|H5N1|USA|Spatulaclypeata|USA_NV|Alphainfluenzavirus|Alphainfluenzavirusinfluenz">
<date value="2022.8465753424657" direction="forwards" units="years"/>
</taxon>
<taxon id="PQ714785.1|InfluenzaAvirus_A/Northernshoveler/OR/22-036880-083-original/2022_H5N1__segment4hemagglutinin_HA_genecompletecds|2022-11-07|4|H5N1|USA|Spatulaclypeata|USA_OR|Alphainfluenzavirus|Alphainfluenzavirusinfluenz">
<date value="2022.8520547945207" direction="forwards" units="years"/>
</taxon>
<taxon id="PQ714937.1|InfluenzaAvirus_A/Parasiticjaeger/AK/22-020952-001-original/2022_H5N1__segment4hemagglutinin_HA_genecompletecds|2022-07-22|4|H5N1|USA||USA_AK|Alphainfluenzavirus|Alphainfluenzavirusinfluenz">
<date value="2022.5561643835617" direction="forwards" units="years"/>
</taxon>
<taxon id="PQ714953.1|InfluenzaAvirus_A/Pelican/AZ/22-038234-001-original/2022_H5N1__segment4hemagglutinin_HA_genecompletecds|2022-10-15|4|H5N1|USA|Pelecanidae|USA_AZ|Alphainfluenzavirus|Alphainfluenzavirusinfluenz">
<date value="2022.7342465753425" direction="forwards" units="years"/>
</taxon>
<taxon id="PQ714961.1|InfluenzaAvirus_A/Pelican/AZ/22-038234-002-original/2022_H5N1__segment4hemagglutinin_HA_genecompletecds|2022-10-15|4|H5N1|USA|Pelecanidae|USA_AZ|Alphainfluenzavirus|Alphainfluenzavirusinfluenz">
<date value="2022.7342465753425" direction="forwards" units="years"/>
</taxon>

<taxon id="PQ714969.1|InfluenzaAvirus_A/Pelican/AZ/22-038234-003-original/2022_H5N1__segment4hemagglutinin_HA_genecompletecds|2022-10-15|4|H5N1|USA|Pelecanidae|USA_AZ|Alphainfluenzavirus|Alphainfluenzavirusinfluenz">
<date value="2022.7342465753425" direction="forwards" units="years"/>
</taxon>
<taxon id="PQ714977.1|InfluenzaAvirus_A/Pelican/AZ/22-038234-004-original/2022_H5N1__segment4hemagglutinin_HA_genecompletecds|2022-10-15|4|H5N1|USA|Pelecanidae|USA_AZ|Alphainfluenzavirus|Alphainfluenzavirusinfluenz">
<date value="2022.7342465753425" direction="forwards" units="years"/>
</taxon>
<taxon id="PQ714985.1|InfluenzaAvirus_A/Pelican/AZ/22-038234-005-original/2022_H5N1__segment4hemagglutinin_HA_genecompletecds|2022-10-15|4|H5N1|USA|Pelecanidae|USA_AZ|Alphainfluenzavirus|Alphainfluenzavirusinfluenz">
<date value="2022.7342465753425" direction="forwards" units="years"/>
</taxon>
<taxon id="PQ714993.1|InfluenzaAvirus_A/Pelican/CA/22-037519-001-original/2022_H5N1__segment4hemagglutinin_HA_genecompletecds|2022-11-04|4|H5N1|USA|Pelecanidae|USA_CA|Alphainfluenzavirus|Alphainfluenzavirusinfluenz">
<date value="2022.8438356164384" direction="forwards" units="years"/>
</taxon>
<taxon id="PQ715033.1|InfluenzaAvirus_A/Pelican/KS/22-036624-001-original-repeat/2022_H5N1__segment4hemagglutinin_HA_genecompletecds|2022-10-17|4|H5N1|USA|Pelecanidae|USA_KS|Alphainfluenzavirus|Alphainfluenzavirusinfluenz">
<date value="2022.7397260273972" direction="forwards" units="years"/>
</taxon>
<taxon id="PQ715065.1|InfluenzaAvirus_A/Pelican/NE/22-032908-001-original/2022_H5N1__segment4hemagglutinin_HA_genecompletecds|2022-10-13|4|H5N1|USA|Pelecanidae|USA_NE|Alphainfluenzavirus|Alphainfluenzavirusinfluenz">
<date value="2022.7287671232878" direction="forwards" units="years"/>
</taxon>
<taxon id="PQ715089.1|InfluenzaAvirus_A/Peregrinefalcon/CA/22-032573-001-original/2022_H5N1__segment4hemagglutinin_HA_genecompletecds|2022-10-03|4|H5N1|USA|Falcoperegrinus|USA_CA|Alphainfluenzavirus|Alphainfluenzavirusinfluenz">
<date value="2022.7013698630137" direction="forwards" units="years"/>
</taxon>
<taxon id="PQ715105.1|InfluenzaAvirus_A/Peregrinefalcon/CA/22-039721-003-original-3/2022_H5N1__segment4hemagglutinin_HA_genecompletecds|2022-11-16|4|H5N1|USA|Falcoperegrinus|USA_CA|Alphainfluenzavirus|Alphainfluenzavirusinfluenz">
<date value="2022.876712328767" direction="forwards" units="years"/>
</taxon>
<taxon id="PQ715121.1|InfluenzaAvirus_A/Peregrinefalcon/FL/22-035514-001-original/2022_H5N1__segment4hemagglutinin_HA_genecompletecds|2022-10-29|4|H5N1|USA|Falcoperegrinus|USA_FL|Alphainfluenzavirus|Alphainfluenzavirusinfluenz">
<date value="2022.772602739726" direction="forwards" units="years"/>
</taxon>
<taxon id="PQ715129.1|InfluenzaAvirus_A/Peregrinefalcon/FL/22-035514-002-original/2022_H5N1__segment4hemagglutinin_HA_genecompletecds|2022-10-29|4|H5N1|USA|Falcoperegrinus|USA_FL|Alphainfluenzavirus|Alphainfluenzavirusinfluenz">
<date value="2022.772602739726" direction="forwards" units="years"/>
</taxon>

<taxon id="PQ715137.1|InfluenzaAvirus_A/Peregrinefalcon/IN/22-032710-001-original/2022_H5N1__segment4hemagglutinin_HA_genecompletecds|2022-10-07|4|H5N1|USA|Falcoperegrinus|USA_IN|Alphainfluenzavirus|Alphainfluenzavirusinfluenz">
<date value="2022.7123287671234" direction="forwards" units="years"/>
</taxon>
<taxon id="PQ715233.1|InfluenzaAvirus_A/Peregrinefalcon/MN/22-031157-003-original-repeat/2022_H5N1__segment4hemagglutinin_HA_genecompletecds|2022-09-29|4|H5N1|USA|Falcoperegrinus|USA_MN|Alphainfluenzavirus|Alphainfluenzavirusinfluenz">
<date value="2022.7452054794521" direction="forwards" units="years"/>
</taxon>
<taxon id="PQ715241.1|InfluenzaAvirus_A/Peregrinefalcon/MN/22-032849-001-original/2022_H5N1__segment4hemagglutinin_HA_genecompletecds|2022-10-13|4|H5N1|USA|Falcoperegrinus|USA_MN|Alphainfluenzavirus|Alphainfluenzavirusinfluenz">
<date value="2022.7287671232878" direction="forwards" units="years"/>
</taxon>
<taxon id="PQ715249.1|InfluenzaAvirus_A/Peregrinefalcon/MN/22-032849-002-original/2022_H5N1__segment4hemagglutinin_HA_genecompletecds|2022-10-13|4|H5N1|USA|Falcoperegrinus|USA_MN|Alphainfluenzavirus|Alphainfluenzavirusinfluenz">
<date value="2022.7287671232878" direction="forwards" units="years"/>
</taxon>
<taxon id="PQ715385.1|InfluenzaAvirus_A/Raven/AK/22-020951-001-original/2022_H5N1__segment4hemagglutinin_HA_genecompletecds|2022-06-26|4|H5N1|USA|Corvus|USA_AK|Alphainfluenzavirus|Alphainfluenzavirusinfluenz">
<date value="2022.4849315068493" direction="forwards" units="years"/>
</taxon>
<taxon id="PQ715393.1|InfluenzaAvirus_A/Raven/AK/22-020951-002-original/2022_H5N1__segment4hemagglutinin_HA_genecompletecds|2022-06-26|4|H5N1|USA|Corvus|USA_AK|Alphainfluenzavirus|Alphainfluenzavirusinfluenz">
<date value="2022.4849315068493" direction="forwards" units="years"/>
</taxon>
<taxon id="PQ715417.1|InfluenzaAvirus_A/Raven/AK/22-031265-001-original/2022_H5N1__segment4hemagglutinin_HA_genecompletecds|2022-09-20|4|H5N1|USA|Corvus|USA_AK|Alphainfluenzavirus|Alphainfluenzavirusinfluenz">
<date value="2022.7205479452055" direction="forwards" units="years"/>
</taxon>
<taxon id="PQ715537.1|InfluenzaAvirus_A/RedFox/AK/22-020792-002-original/2022_H5N1__segment4hemagglutinin_HA_genecompletecds|2022-06-13|4|H5N1|USA|Vulpesvulpes|USA_AK|Alphainfluenzavirus|Alphainfluenzavirusinfluenz">
<date value="2022.4493150684932" direction="forwards" units="years"/>
</taxon>
<taxon id="PQ715649.1|InfluenzaAvirus_A/Red-tailedhawk/CA/22-033187-001-original/2022_H5N1__segment4hemagglutinin_HA_genecompletecds|2022-10-12|4|H5N1|USA|Buteojamaicensis|USA_CA|Alphainfluenzavirus|Alphainfluenzavirusinfluenz">
<date value="2022.7260273972602" direction="forwards" units="years"/>
</taxon>
<taxon id="PQ715737.1|InfluenzaAvirus_A/Red-tailedhawk/CO/22-029818-001-original-repeat/2022_H5N1__segment4hemagglutinin_HA_genecompletecds|2022-09-19|4|H5N1|USA|Buteojamaicensis|USA_CO|Alphainfluenzavirus|Alphainfluenzavirusinfluenz">
<date value="2022.717808219178" direction="forwards" units="years"/>

</taxon>
<taxon id="PQ715745.1|InfluenzaAvirus_A/Red-tailedhawk/CO/22-031741-001-original/2022_H5N1__segment4hemagglutinin_HA_genecompletecds|2022-10-04|4|H5N1|USA|Buteojamaicensis|USA_CO|Alphainfluenzavirus|Alphainfluenzavirusinfluenz">
<date value="2022.704109589041" direction="forwards" units="years"/>
</taxon>
<taxon id="PQ715753.1|InfluenzaAvirus_A/Red-tailedhawk/CO/22-037213-001-original/2022_H5N1__segment4hemagglutinin_HA_genecompletecds|2022-11-14|4|H5N1|USA|Buteojamaicensis|USA_CO|Alphainfluenzavirus|Alphainfluenzavirusinfluenz">
<date value="2022.8712328767124" direction="forwards" units="years"/>
</taxon>
<taxon id="PQ715761.1|InfluenzaAvirus_A/Red-tailedhawk/CO/22-037824-001-original/2022_H5N1__segment4hemagglutinin_HA_genecompletecds|2022-11-18|4|H5N1|USA|Buteojamaicensis|USA_CO|Alphainfluenzavirus|Alphainfluenzavirusinfluenz">
<date value="2022.8821917808218" direction="forwards" units="years"/>
</taxon>
<taxon id="PQ716385.1|InfluenzaAvirus_A/Red-tailedhawk/MT/22-036316-001-original/2022_H5N1__segment4hemagglutinin_HA_genecompletecds|2022-11-09|4|H5N1|USA|Buteojamaicensis|USA_MT|Alphainfluenzavirus|Alphainfluenzavirusinfluenz">
<date value="2022.8575342465754" direction="forwards" units="years"/>
</taxon>
<taxon id="PQ716737.1|InfluenzaAvirus_A/Red-tailedhawk/WA/22-037658-001-original/2022_H5N1__segment4hemagglutinin_HA_genecompletecds|2022-11-16|4|H5N1|USA|Buteojamaicensis|USA_WA|Alphainfluenzavirus|Alphainfluenzavirusinfluenz">
<date value="2022.876712328767" direction="forwards" units="years"/>
</taxon>
<taxon id="PQ716913.1|InfluenzaAvirus_A/Ring-billedgull/CO/22-030941-001-original/2022_H5N1__segment4hemagglutinin_HA_genecompletecds|2022-09-28|4|H5N1|USA|Larusdelawarensis|USA_CO|Alphainfluenzavirus|Alphainfluenzavirusinfluenz">
<date value="2022.7424657534248" direction="forwards" units="years"/>
</taxon>
<taxon id="PQ717001.1|InfluenzaAvirus_A/Roseatespoonbill/TX/22-035763-001-original/2022_H5N1__segment4hemagglutinin_HA_genecompletecds|2022-10-11|4|H5N1|USA||USA_TX|Alphainfluenzavirus|Alphainfluenzavirusinfluenz">
<date value="2022.7232876712328" direction="forwards" units="years"/>
</taxon>
<taxon id="PQ717049.1|InfluenzaAvirus_A/Ross_sgoose/KS/22-038592-005-original/2022_H5N1__segment4hemagglutinin_HA_genecompletecds|2022-11-21|4|H5N1|USA||USA_KS|Alphainfluenzavirus|Alphainfluenzavirusinfluenz">
<date value="2022.890410958904" direction="forwards" units="years"/>
</taxon>
<taxon id="PQ717073.1|InfluenzaAvirus_A/Ross_sgoose/KS/22-041130-002-original/2022_H5N1__segment4hemagglutinin_HA_genecompletecds|2022-11-29|4|H5N1|USA||USA_KS|Alphainfluenzavirus|Alphainfluenzavirusinfluenz">
<date value="2022.9123287671232" direction="forwards" units="years"/>
</taxon>
<taxon id="PQ717225.1|InfluenzaAvirus_A/Sandhillcrane/AK/22-019268-001-original/2022_H5N1__segment4hemagglutinin_HA_genecompletecds|2022-06-14|4|H5N1|USA|Antigonecanadensis|USA_AK|Alphainfluenzavirus|Alphainfluenzavirusinfluenz">
<date value="2022.4520547945206" direction="forwards" units="years"/>
</taxon>
<taxon id="PQ717329.1|InfluenzaAvirus_A/Short-earedOwl/WA/22-039678-004-original/2022_H5N1__segment4hemagglutinin_HA_genecompletecds|2022-11-

21|4|H5N1|USA|Asioflammeus|USA_WA|Alphainfluenzavirus|Alphainfluenzavirusin
fluenz">
<date value="2022.890410958904" direction="forwards" units="years"/>
</taxon>
<taxon id="PQ717337.1|InfluenzaAvirus_A/Snowgoose/AK/22-017364-001-
original/2022_H5N1__segment4hemagglutinin_HA_genecompletecds|2022-05-
27|4|H5N1|USA|Ansercaerulescens|USA_AK|Alphainfluenzavirus|Alphainfluenzavi
rusinfluenz">
<date value="2022.4027397260274" direction="forwards" units="years"/>
</taxon>
<taxon id="PQ717353.1|InfluenzaAvirus_A/Snowgoose/AR/22-038202-001-
original/2022_H5N1__segment4hemagglutinin_HA_genecompletecds|2022-11-
25|4|H5N1|USA|Ansercaerulescens|USA_AR|Alphainfluenzavirus|Alphainfluenzavi
rusinfluenz">
<date value="2022.9013698630138" direction="forwards" units="years"/>
</taxon>
<taxon id="PQ717361.1|InfluenzaAvirus_A/Snowgoose/AR/22-039436-005-
original/2022_H5N1__segment4hemagglutinin_HA_genecompletecds|2022-11-
29|4|H5N1|USA|Ansercaerulescens|USA_AR|Alphainfluenzavirus|Alphainfluenzavi
rusinfluenz">
<date value="2022.9123287671232" direction="forwards" units="years"/>
</taxon>
<taxon id="PQ717441.1|InfluenzaAvirus_A/Snowgoose/CA/22-041406-001-
original/2022_H5N1__segment4hemagglutinin_HA_genecompletecds|2022-11-
28|4|H5N1|USA|Ansercaerulescens|USA_CA|Alphainfluenzavirus|Alphainfluenzavi
rusinfluenz">
<date value="2022.9095890410958" direction="forwards" units="years"/>
</taxon>
<taxon id="PQ717513.1|InfluenzaAvirus_A/Snowgoose/CA/23-001856-001-
original/2022_H5N1__segment4hemagglutinin_HA_genecompletecds|2022-11-
28|4|H5N1|USA|Ansercaerulescens|USA_CA|Alphainfluenzavirus|Alphainfluenzavi
rusinfluenz">
<date value="2022.9095890410958" direction="forwards" units="years"/>
</taxon>
<taxon id="PQ717537.1|InfluenzaAvirus_A/Snowgoose/CO/22-036625-001-
original-repeat/2022_H5N1__segment4hemagglutinin_HA_genecompletecds|2022-
11-
08|4|H5N1|USA|Ansercaerulescens|USA_CO|Alphainfluenzavirus|Alphainfluenzavi
rusinfluenz">
<date value="2022.854794520548" direction="forwards" units="years"/>
</taxon>
<taxon id="PQ717545.1|InfluenzaAvirus_A/Snowgoose/CO/22-037670-001-
original/2022_H5N1__segment4hemagglutinin_HA_genecompletecds|2022-11-
16|4|H5N1|USA|Ansercaerulescens|USA_CO|Alphainfluenzavirus|Alphainfluenzavi
rusinfluenz">
<date value="2022.876712328767" direction="forwards" units="years"/>
</taxon>
<taxon id="PQ717569.1|InfluenzaAvirus_A/Snowgoose/ID/22-038611-001-
original/2022_H5N1__segment4hemagglutinin_HA_genecompletecds|2022-11-
18|4|H5N1|USA|Ansercaerulescens|USA_ID|Alphainfluenzavirus|Alphainfluenzavi
rusinfluenz">
<date value="2022.8821917808218" direction="forwards" units="years"/>
</taxon>
<taxon id="PQ717577.1|InfluenzaAvirus_A/Snowgoose/ID/22-038611-002-
original/2022_H5N1__segment4hemagglutinin_HA_genecompletecds|2022-11-
18|4|H5N1|USA|Ansercaerulescens|USA_ID|Alphainfluenzavirus|Alphainfluenzavi
rusinfluenz">
<date value="2022.8821917808218" direction="forwards" units="years"/>
</taxon>
<taxon id="PQ717593.1|InfluenzaAvirus_A/Snowgoose/IN/22-038827-002-
original-repeat/2022_H5N1__segment4hemagglutinin_HA_genecompletecds|2022-

11-
23|4|H5N1|USA|Ansercaerulescens|USA_IN|Alphainfluenzavirus|Alphainfluenzavi
rusinfluenz">
<date value="2022.8958904109588" direction="forwards" units="years"/>
</taxon>
<taxon id="PQ717601.1|InfluenzaAvirus_A/Snowgoose/IN/22-038827-003-
original-repeat/2022_H5N1__segment4hemagglutinin_HA_genecompletecds|2022-
11-
23|4|H5N1|USA|Ansercaerulescens|USA_IN|Alphainfluenzavirus|Alphainfluenzavi
rusinfluenz">
<date value="2022.8958904109588" direction="forwards" units="years"/>
</taxon>
<taxon id="PQ717633.1|InfluenzaAvirus_A/Snowgoose/IN/22-038827-008-
original-repeat/2022_H5N1__segment4hemagglutinin_HA_genecompletecds|2022-
11-
23|4|H5N1|USA|Ansercaerulescens|USA_IN|Alphainfluenzavirus|Alphainfluenzavi
rusinfluenz">
<date value="2022.8958904109588" direction="forwards" units="years"/>
</taxon>
<taxon id="PQ717657.1|InfluenzaAvirus_A/Snowgoose/KS/22-039690-001-
original/2022_H5N1__segment4hemagglutinin_HA_genecompletecds|2022-11-
21|4|H5N1|USA|Ansercaerulescens|USA_KS|Alphainfluenzavirus|Alphainfluenzavi
rusinfluenz">
<date value="2022.890410958904" direction="forwards" units="years"/>
</taxon>
<taxon id="PQ717665.1|InfluenzaAvirus_A/Snowgoose/KS/22-039690-002-
original/2022_H5N1__segment4hemagglutinin_HA_genecompletecds|2022-11-
23|4|H5N1|USA|Ansercaerulescens|USA_KS|Alphainfluenzavirus|Alphainfluenzavi
rusinfluenz">
<date value="2022.8958904109588" direction="forwards" units="years"/>
</taxon>
<taxon id="PQ717689.1|InfluenzaAvirus_A/Snowgoose/KS/22-039690-005-
original/2022_H5N1__segment4hemagglutinin_HA_genecompletecds|2022-11-
23|4|H5N1|USA|Ansercaerulescens|USA_KS|Alphainfluenzavirus|Alphainfluenzavi
rusinfluenz">
<date value="2022.8958904109588" direction="forwards" units="years"/>
</taxon>
<taxon id="PQ717697.1|InfluenzaAvirus_A/Snowgoose/KS/22-039690-006-
original/2022_H5N1__segment4hemagglutinin_HA_genecompletecds|2022-11-
28|4|H5N1|USA|Ansercaerulescens|USA_KS|Alphainfluenzavirus|Alphainfluenzavi
rusinfluenz">
<date value="2022.9095890410958" direction="forwards" units="years"/>
</taxon>
<taxon id="PQ717705.1|InfluenzaAvirus_A/Snowgoose/KS/22-039690-007-
original/2022_H5N1__segment4hemagglutinin_HA_genecompletecds|2022-11-
27|4|H5N1|USA|Ansercaerulescens|USA_KS|Alphainfluenzavirus|Alphainfluenzavi
rusinfluenz">
<date value="2022.9068493150685" direction="forwards" units="years"/>
</taxon>
<taxon id="PQ717769.1|InfluenzaAvirus_A/Snowgoose/LA/22-039688-006-
original/2022_H5N1__segment4hemagglutinin_HA_genecompletecds|2022-11-
28|4|H5N1|USA|Ansercaerulescens|USA_LA|Alphainfluenzavirus|Alphainfluenzavi
rusinfluenz">
<date value="2022.9095890410958" direction="forwards" units="years"/>
</taxon>
<taxon id="PQ717777.1|InfluenzaAvirus_A/Snowgoose/LA/22-039688-009-
original/2022_H5N1__segment4hemagglutinin_HA_genecompletecds|2022-11-
30|4|H5N1|USA|Ansercaerulescens|USA_LA|Alphainfluenzavirus|Alphainfluenzavi
rusinfluenz">
<date value="2022.9150684931508" direction="forwards" units="years"/>
</taxon>

<taxon id="PQ717793.1|InfluenzaAvirus_A/Snowgoose/LA/23-000409-003-original/2022_H5N1__segment4hemagglutinin_HA_genecompletecds|2022-11-28|4|H5N1|USA|Ansercaerulescens|USA_LA|Alphainfluenzavirus|Alphainfluenzavirusinfluenz">
<date value="2022.9095890410958" direction="forwards" units="years"/>
</taxon>
<taxon id="PQ717841.1|InfluenzaAvirus_A/Snowgoose/MN/22-037439-001-original/2022_H5N1__segment4hemagglutinin_HA_genecompletecds|2022-11-17|4|H5N1|USA|Ansercaerulescens|USA_MN|Alphainfluenzavirus|Alphainfluenzavirusinfluenz">
<date value="2022.8794520547945" direction="forwards" units="years"/>
</taxon>
<taxon id="PQ717881.1|InfluenzaAvirus_A/Snowgoose/MO/22-037787-001-original/2022_H5N1__segment4hemagglutinin_HA_genecompletecds|2022-11-13|4|H5N1|USA|Ansercaerulescens|USA_MO|Alphainfluenzavirus|Alphainfluenzavirusinfluenz">
<date value="2022.868493150685" direction="forwards" units="years"/>
</taxon>
<taxon id="PQ717897.1|InfluenzaAvirus_A/Snowgoose/MO/22-038564-002-original/2022_H5N1__segment4hemagglutinin_HA_genecompletecds|2022-11-23|4|H5N1|USA|Ansercaerulescens|USA_MO|Alphainfluenzavirus|Alphainfluenzavirusinfluenz">
<date value="2022.8958904109588" direction="forwards" units="years"/>
</taxon>
<taxon id="PQ717945.1|InfluenzaAvirus_A/Snowgoose/MS/22-038820-002-original-repeat/2022_H5N1__segment4hemagglutinin_HA_genecompletecds|2022-11-27|4|H5N1|USA|Ansercaerulescens|USA_MS|Alphainfluenzavirus|Alphainfluenzavirusinfluenz">
<date value="2022.9068493150685" direction="forwards" units="years"/>
</taxon>
<taxon id="PQ717969.1|InfluenzaAvirus_A/Snowgoose/MS/22-038820-006-original-repeat/2022_H5N1__segment4hemagglutinin_HA_genecompletecds|2022-11-27|4|H5N1|USA|Ansercaerulescens|USA_MS|Alphainfluenzavirus|Alphainfluenzavirusinfluenz">
<date value="2022.9068493150685" direction="forwards" units="years"/>
</taxon>
<taxon id="PQ717985.1|InfluenzaAvirus_A/Snowgoose/MS/22-038821-004-original/2022_H5N1__segment4hemagglutinin_HA_genecompletecds|2022-11-23|4|H5N1|USA|Ansercaerulescens|USA_MS|Alphainfluenzavirus|Alphainfluenzavirusinfluenz">
<date value="2022.8958904109588" direction="forwards" units="years"/>
</taxon>
<taxon id="PQ718009.1|InfluenzaAvirus_A/Snowgoose/MS/22-038821-008-original/2022_H5N1__segment4hemagglutinin_HA_genecompletecds|2022-11-23|4|H5N1|USA|Ansercaerulescens|USA_MS|Alphainfluenzavirus|Alphainfluenzavirusinfluenz">
<date value="2022.8958904109588" direction="forwards" units="years"/>
</taxon>
<taxon id="PQ718017.1|InfluenzaAvirus_A/Snowgoose/MS/22-038821-010-original/2022_H5N1__segment4hemagglutinin_HA_genecompletecds|2022-11-23|4|H5N1|USA|Ansercaerulescens|USA_MS|Alphainfluenzavirus|Alphainfluenzavirusinfluenz">
<date value="2022.8958904109588" direction="forwards" units="years"/>
</taxon>
<taxon id="PQ718025.1|InfluenzaAvirus_A/Snowgoose/MS/22-038821-011-original/2022_H5N1__segment4hemagglutinin_HA_genecompletecds|2022-11-23|4|H5N1|USA|Ansercaerulescens|USA_MS|Alphainfluenzavirus|Alphainfluenzavirusinfluenz">
<date value="2022.8958904109588" direction="forwards" units="years"/>

</taxon>
<taxon id="PQ718033.1|InfluenzaAvirus_A/Snowgoose/MS/22-038821-012-original/2022_H5N1__segment4hemagglutinin_HA_genecompletecds|2022-11-23|4|H5N1|USA|Ansercaerulescens|USA_MS|Alphainfluenzavirus|Alphainfluenzavirusinfluenz">
<date value="2022.8958904109588" direction="forwards" units="years"/>
</taxon>
<taxon id="PQ718049.1|InfluenzaAvirus_A/Snowgoose/MS/22-038821-019-original/2022_H5N1__segment4hemagglutinin_HA_genecompletecds|2022-11-23|4|H5N1|USA|Ansercaerulescens|USA_MS|Alphainfluenzavirus|Alphainfluenzavirusinfluenz">
<date value="2022.8958904109588" direction="forwards" units="years"/>
</taxon>
<taxon id="PQ718073.1|InfluenzaAvirus_A/Snowgoose/MS/22-038821-023-original/2022_H5N1__segment4hemagglutinin_HA_genecompletecds|2022-11-23|4|H5N1|USA|Ansercaerulescens|USA_MS|Alphainfluenzavirus|Alphainfluenzavirusinfluenz">
<date value="2022.8958904109588" direction="forwards" units="years"/>
</taxon>
<taxon id="PQ718089.1|InfluenzaAvirus_A/Snowgoose/MS/22-038821-025-original/2022_H5N1__segment4hemagglutinin_HA_genecompletecds|2022-11-23|4|H5N1|USA|Ansercaerulescens|USA_MS|Alphainfluenzavirus|Alphainfluenzavirusinfluenz">
<date value="2022.8958904109588" direction="forwards" units="years"/>
</taxon>
<taxon id="PQ718097.1|InfluenzaAvirus_A/Snowgoose/MS/22-038821-026-original/2022_H5N1__segment4hemagglutinin_HA_genecompletecds|2022-11-23|4|H5N1|USA|Ansercaerulescens|USA_MS|Alphainfluenzavirus|Alphainfluenzavirusinfluenz">
<date value="2022.8958904109588" direction="forwards" units="years"/>
</taxon>
<taxon id="PQ718121.1|InfluenzaAvirus_A/Snowgoose/MS/22-038821-030-original/2022_H5N1__segment4hemagglutinin_HA_genecompletecds|2022-11-23|4|H5N1|USA|Ansercaerulescens|USA_MS|Alphainfluenzavirus|Alphainfluenzavirusinfluenz">
<date value="2022.8958904109588" direction="forwards" units="years"/>
</taxon>
<taxon id="PQ718153.1|InfluenzaAvirus_A/Snowgoose/MS/22-038821-039-original/2022_H5N1__segment4hemagglutinin_HA_genecompletecds|2022-11-23|4|H5N1|USA|Ansercaerulescens|USA_MS|Alphainfluenzavirus|Alphainfluenzavirusinfluenz">
<date value="2022.8958904109588" direction="forwards" units="years"/>
</taxon>
<taxon id="PQ718169.1|InfluenzaAvirus_A/Snowgoose/MS/22-038821-044-original/2022_H5N1__segment4hemagglutinin_HA_genecompletecds|2022-11-23|4|H5N1|USA|Ansercaerulescens|USA_MS|Alphainfluenzavirus|Alphainfluenzavirusinfluenz">
<date value="2022.8958904109588" direction="forwards" units="years"/>
</taxon>
<taxon id="PQ718281.1|InfluenzaAvirus_A/Snowgoose/ND/23-002238-001-original/2022_H5N1__segment4hemagglutinin_HA_genecompletecds|2022-11-29|4|H5N1|USA|Ansercaerulescens|USA_ND|Alphainfluenzavirus|Alphainfluenzavirusinfluenz">
<date value="2022.9123287671232" direction="forwards" units="years"/>
</taxon>
<taxon id="PQ718385.1|InfluenzaAvirus_A/Snowgoose/OR/22-038527-001-original/2022_H5N1__segment4hemagglutinin_HA_genecompletecds|2022-11-17|4|H5N1|USA|Ansercaerulescens|USA_OR|Alphainfluenzavirus|Alphainfluenzavirusinfluenz">
<date value="2022.8794520547945" direction="forwards" units="years"/>
</taxon>

<taxon id="PQ718393.1|InfluenzaAvirus_A/Snowgoose/OR/23-002231-001-original/2022_H5N1__segment4hemagglutinin_HA_genecompletecds|2022-11-19|4|H5N1|USA|Ansercaerulescens|USA_OR|Alphainfluenzavirus|Alphainfluenzavirusinfluenz">
<date value="2022.8849315068494" direction="forwards" units="years"/>
</taxon>
<taxon id="PQ718409.1|InfluenzaAvirus_A/Snowgoose/SD/22-038546-001-original/2022_H5N1__segment4hemagglutinin_HA_genecompletecds|2022-11-21|4|H5N1|USA|Ansercaerulescens|USA_SD|Alphainfluenzavirus|Alphainfluenzavirusinfluenz">
<date value="2022.890410958904" direction="forwards" units="years"/>
</taxon>
<taxon id="PQ718417.1|InfluenzaAvirus_A/Snowgoose/SD/22-038788-002-original/2022_H5N1__segment4hemagglutinin_HA_genecompletecds|2022-11-21|4|H5N1|USA|Ansercaerulescens|USA_SD|Alphainfluenzavirus|Alphainfluenzavirusinfluenz">
<date value="2022.890410958904" direction="forwards" units="years"/>
</taxon>
<taxon id="PQ718441.1|InfluenzaAvirus_A/Snowgoose/WA/22-037636-001-original/2022_H5N1__segment4hemagglutinin_HA_genecompletecds|2022-11-15|4|H5N1|USA|Ansercaerulescens|USA_WA|Alphainfluenzavirus|Alphainfluenzavirusinfluenz">
<date value="2022.8739726027397" direction="forwards" units="years"/>
</taxon>
<taxon id="PQ718449.1|InfluenzaAvirus_A/Snowgoose/WA/22-039678-001-original/2022_H5N1__segment4hemagglutinin_HA_genecompletecds|2022-11-21|4|H5N1|USA|Ansercaerulescens|USA_WA|Alphainfluenzavirus|Alphainfluenzavirusinfluenz">
<date value="2022.890410958904" direction="forwards" units="years"/>
</taxon>
<taxon id="PQ718473.1|InfluenzaAvirus_A/Snowyowl/AK/22-022807-001-original/2022_H5N1__segment4hemagglutinin_HA_genecompletecds|2022-07-19|4|H5N1|USA|Buboscandiacus|USA_AK|Alphainfluenzavirus|Alphainfluenzavirusinfluenz">
<date value="2022.5479452054794" direction="forwards" units="years"/>
</taxon>
<taxon id="PQ718537.1|InfluenzaAvirus_A/Sparrow/NE/22-033885-001-original/2022_H5N1__segment4hemagglutinin_HA_genecompletecds|2022-10-19|4|H5N1|USA|Passeridae|USA_NE|Alphainfluenzavirus|Alphainfluenzavirusinfluenz">
<date value="2022.7452054794521" direction="forwards" units="years"/>
</taxon>
<taxon id="PQ718577.1|InfluenzaAvirus_A/Swainson_sHawk/OR/22-034146-001-original-repeat/2022_H5N1__segment4hemagglutinin_HA_genecompletecds|2022-10-10|4|H5N1|USA|Buteoswainsoni|USA_OR|Alphainfluenzavirus|Alphainfluenzavirusinfluenz">
<date value="2022.7205479452055" direction="forwards" units="years"/>
</taxon>
<taxon id="PQ718761.1|InfluenzaAvirus_A/TrumpeterSwan/MN/22-037438-001-original/2022_H5N1__segment4hemagglutinin_HA_genecompletecds|2022-11-17|4|H5N1|USA||USA_MN|Alphainfluenzavirus|Alphainfluenzavirusinfluenz">
<date value="2022.8794520547945" direction="forwards" units="years"/>
</taxon>
<taxon id="PQ718777.1|InfluenzaAvirus_A/TrumpeterSwan/MT/22-030263-001-original/2022_H5N1__segment4hemagglutinin_HA_genecompletecds|2022-09-16|4|H5N1|USA||USA_MT|Alphainfluenzavirus|Alphainfluenzavirusinfluenz">
<date value="2022.7095890410958" direction="forwards" units="years"/>
</taxon>

<taxon id="PQ718785.1|InfluenzaAvirus_A/TrumpeterSwan/WY/22-030049-002-original/2022_H5N1__segment4hemagglutinin_HA_genecompletecds|2022-09-14|4|H5N1|USA||USA_WY|Alphainfluenzavirus|Alphainfluenzavirusinfluenz">
<date value="2022.704109589041" direction="forwards" units="years"/>
</taxon>
<taxon id="PQ718793.1|InfluenzaAvirus_A/TrumpeterSwan/WY/22-030049-003-original/2022_H5N1__segment4hemagglutinin_HA_genecompletecds|2022-09-14|4|H5N1|USA||USA_WY|Alphainfluenzavirus|Alphainfluenzavirusinfluenz">
<date value="2022.704109589041" direction="forwards" units="years"/>
</taxon>
<taxon id="PQ718817.1|InfluenzaAvirus_A/TundraSwan/WI/22-038828-001-original-repeat/2022_H5N1__segment4hemagglutinin_HA_genecompletecds|2022-11-22|4|H5N1|USA|Cygnuscolumbianus|USA_WI|Alphainfluenzavirus|Alphainfluenzavirusinfluenz">
<date value="2022.8931506849315" direction="forwards" units="years"/>
</taxon>
<taxon id="PQ719113.1|InfluenzaAvirus_A/TurkeyVulture/MN/22-035505-002-original/2022_H5N1__segment4hemagglutinin_HA_genecompletecds|2022-10-29|4|H5N1|USA|Cathartesaura|USA_MN|Alphainfluenzavirus|Alphainfluenzavirusinfluenz">
<date value="2022.772602739726" direction="forwards" units="years"/>
</taxon>
<taxon id="PQ719425.1|InfluenzaAvirus_A/TurkeyVulture/WY/22-031606-001-original-repeat/2022_H5N1__segment4hemagglutinin_HA_genecompletecds|2022-10-03|4|H5N1|USA|Cathartesaura|USA_WY|Alphainfluenzavirus|Alphainfluenzavirusinfluenz">
<date value="2022.7013698630137" direction="forwards" units="years"/>
</taxon>
<taxon id="PQ719473.1|InfluenzaAvirus_A/WesternGull/CA/22-035882-001-original/2022_H5N1__segment4hemagglutinin_HA_genecompletecds|2022-11-03|4|H5N1|USA|Larusoccidentalis|USA_CA|Alphainfluenzavirus|Alphainfluenzavirusinfluenz">
<date value="2022.841095890411" direction="forwards" units="years"/>
</taxon>
<taxon id="PQ719481.1|InfluenzaAvirus_A/WesternGull/CA/22-037512-001-original/2022_H5N1__segment4hemagglutinin_HA_genecompletecds|2022-11-08|4|H5N1|USA|Larusoccidentalis|USA_CA|Alphainfluenzavirus|Alphainfluenzavirusinfluenz">
<date value="2022.854794520548" direction="forwards" units="years"/>
</taxon>
<taxon id="PQ719521.1|InfluenzaAvirus_A/Westernscreechowl/WA/22-015526-006-original/2022_H5N1__segment4hemagglutinin_HA_genecompletecds|2022-05-11|4|H5N1|USA||USA_WA|Alphainfluenzavirus|Alphainfluenzavirusinfluenz">
<date value="2022.358904109589" direction="forwards" units="years"/>
</taxon>
<taxon id="PQ719529.1|InfluenzaAvirus_A/Westernsnowyplover/OR/22-038785-001-original-repeat/2022_H5N1__segment4hemagglutinin_HA_genecompletecds|2022-10-25|4|H5N1|USA||USA_OR|Alphainfluenzavirus|Alphainfluenzavirusinfluenz">
<date value="2022.7616438356165" direction="forwards" units="years"/>
</taxon>
<taxon id="PQ719905.1|InfluenzaAvirus_A/blackskimmer/FL/22-036966-001-original/2022_H5N1__segment4hemagglutinin_HA_genecompletecds|2022-11-04|4|H5N1|USA|Rynchopsniger|USA_FL|Alphainfluenzavirus|Alphainfluenzavirusinfluenz">
<date value="2022.8438356164384" direction="forwards" units="years"/>
</taxon>
<taxon id="PQ719945.1|InfluenzaAvirus_A/goose/AR/22-036798-001-original/2022_H5N1__segment4hemagglutinin_HA_genecompletecds|2022-11-

14|4|H5N1|USA|Anatidae|USA_AR|Alphainfluenzavirus|Alphainfluenzavirusinflue
nz">
<date value="2022.8712328767124" direction="forwards" units="years"/>
</taxon>
<taxon id="PQ720105.1|InfluenzaAvirus_A/goose/TX/22-038236-001-
original/2022_H5N1__segment4hemagglutinin_HA_genecompletecds|2022-11-
23|4|H5N1|USA|Anatidae|USA_TX|Alphainfluenzavirus|Alphainfluenzavirusinflue
nz">
<date value="2022.8958904109588" direction="forwards" units="years"/>
</taxon>
<taxon id="PQ720289.1|InfluenzaAvirus_A/lesserscaup/LA/22-037240-003-
original/2022_H5N1__segment4hemagglutinin_HA_genecompletecds|2022-11-
13|4|H5N1|USA|Aythyaaffinis|USA_LA|Alphainfluenzavirus|Alphainfluenzavirusi
nfluenz">
<date value="2022.868493150685" direction="forwards" units="years"/>
</taxon>
<taxon id="PQ720297.1|InfluenzaAvirus_A/lessersnowgoose/OR/22-037719-001-
original/2022_H5N1__segment4hemagglutinin_HA_genecompletecds|2022-11-
08|4|H5N1|USA|Ansercaerulescenscaerulescens|USA_OR|Alphainfluenzavirus|Alph
ainfluenzavirusinfluenz">
<date value="2022.854794520548" direction="forwards" units="years"/>
</taxon>
<taxon id="A/Brown_skua/Fildes_Peninsula/B1/2024(H5N1)|2024-12-
10|2.3.4.4b||PB2|EPI_ISL_19847535">
<date value="2024.93989071038" direction="forwards" units="years"/>
</taxon>
<taxon id="A/Brown_skua/Fildes_Peninsula/B3/2024(H5N1)|2024-12-
26|2.3.4.4b||PB2|EPI_ISL_19847538">
<date value="2024.98633879781" direction="forwards" units="years"/>
</taxon>
<taxon id="A/Brown_skua/Fildes_Peninsula/F4/2024(H5N1)|2024-12-
26|2.3.4.4b||PB2|EPI_ISL_19847539">
<date value="2024.98633879781" direction="forwards" units="years"/>
</taxon>
<taxon id="A/Brown_skua/Fildes_Peninsula/B2/2024(H5N1)|2024-12-
25|2.3.4.4b||PB2|EPI_ISL_19847536">
<date value="2024.98360655738" direction="forwards" units="years"/>
</taxon>
<taxon id="A/Brown_Skua/Torgersen_Island/o81-b82/2024|2024-12-
17|2.3.4.4b||PB2|EPI_ISL_19645365">
<date value="2024.95901639344" direction="forwards" units="years"/>
</taxon>
<taxon id="A/Brown_skua/Torgersen_Island/o8182/2024|2024-12-
01|2.3.4.4b||PB2|EPI_ISL_19745586">
<date value="2024.9153005464482" direction="forwards" units="years"/>
</taxon>
</taxa>
<!-- The sequence alignment (each sequence refers to a taxon above).
-->
<!-- ntax=1227 nchar=16649
-->
<alignment id="alignment" dataType="nucleotide">
<sequence>
<taxon idref="24P021402_H5N1_D-24-09077_HP_B3.2_CRO_2024-10-20"/>
NNN
</sequence>
<sequence>
<taxon idref="24P021403_H5N1_D-24-09077_HP_B3.2_CRO_2024-10-20"/>
NNN
</sequence>
<sequence>

<taxon idref="24P021404_H5N1_D-24-09077_HP_B3.2_CRO_2024-10-20"/>
NNN
</sequence>
<sequence>
<taxon idref="24P021407_H5N1_D-24-09077_HP_B3.2_CRO_2024-10-20"/>
NNN
</sequence>
<sequence>
<taxon idref="24P021412_H5N1_D-24-09077_HP_B3.2_CRO_2024-10-20"/>
NNN
</sequence>
<sequence>
<taxon idref="24P021415_H5N1_D-24-09077_HP_B3.2_CRO_2024-10-20"/>
NNN
</sequence>
<sequence>
<taxon idref="24P021416_H5N1_D-24-09077_HP_B3.2_CRO_2024-10-20"/>
NNN
</sequence>
<sequence>
<taxon idref="24P021417_H5N1_D-24-09077_HP_B3.2_CRO_2024-10-20"/>
NNN
</sequence>
<sequence>
<taxon idref="24P021420_H5N1_D-24-09077_HP_B3.2_CRO_2024-10-25"/>
NNN
</sequence>
<sequence>
<taxon idref="24P021422_H5N1_D-24-09077_HP_B3.2_CRO_2024-10-25"/>
NNN
</sequence>
<sequence>
<taxon idref="24P023398_H5N1_D-24-10045_HP_B3.2_CRO_2024-11-07"/>
NNN
</sequence>
<sequence>
<taxon idref="24P023401_H5N1_D-24-10045_HP_B3.2_CRO_2024-11-07"/>
NNN
</sequence>
<sequence>
<taxon idref="24P023403_H5N1_D-24-10045_HP_B3.2_CRO_2024-11-06"/>
NNN
</sequence>
<sequence>
<taxon idref="24P023407_H5N1_D-24-10045_HP_B3.2_CRO_2024-11-08"/>
NNN
</sequence>
<sequence>
<taxon idref="24P023412_H5N1_D-24-10045_HP_B3.2_CRO_2024-11-14"/>
NNN
</sequence>
<sequence>
<taxon idref="24P023414_H5N1_D-24-10045_HP_B3.2_CRO_2024-11-11"/>
NNN
</sequence>
<sequence>
<taxon idref="24P023417_H5N1_D-24-10045_HP_B3.2_CRO_2024-11-12"/>
NNN
</sequence>
<sequence>
<taxon idref="24P023419_H5N1_D-24-10045_HP_B3.2_CRO_2024-11-12"/>

NNN
</sequence>
<sequence>
<taxon idref="24P023422_H5N1_D-24-10045_HP_B3.2_CRO_2024-11-16"/>
NNN
</sequence>
<sequence>
<taxon idref="24P023427_H5N1_D-24-10045_HP_B3.2_CRO_2024-11-22"/>
NNN
</sequence>
<sequence>
<taxon idref="24P023433_H5N1_D-24-10045_HP_B3.2_KER_2024-12-01"/>
NNN
</sequence>
<sequence>
<taxon idref="24P023439_H5N1_D-24-10045_HP_B3.2_KER_2024-12-01"/>
NNN
</sequence>
<sequence>
<taxon idref="24P023440_H5N1_D-24-10045_HP_B3.2_KER_2024-12-01"/>
NNN
</sequence>
<sequence>
<taxon idref="24P023443_H5N1_D-24-10045_HP_B3.2_KER_2024-12-02"/>
NNN
</sequence>
<sequence>
<taxon idref="24P023446_H5N1_D-24-10045_HP_B3.2_KER_2024-12-01"/>
NNN
</sequence>
<sequence>
<taxon idref="A/American_Crow/BC/AIVPHL-
861/2022|EPI_ISL_18665555|A_/_H5N1|Original||2.3.4.4b|30.12.2022|Caleta_Jes
sica_Marija|||21.12.2023|B.C._Centre_for_Disease_Control|Public_Health_Agen
cy_of_Canada|HA|4|A/American_Crow/BC/AIVPHL-
861/2022_HA|EPI2856376|DNA_INS"/>
NNN
</sequence>
<sequence>
<taxon idref="A/American_Crow/BC/AIVPHL-
864/2022|EPI_ISL_18665557|A_/_H5N1|Original||2.3.4.4b|30.12.2022|Caleta_Jes
sica_Marija|||21.12.2023|B.C._Centre_for_Disease_Control|Public_Health_Agen
cy_of_Canada|HA|4|A/American_Crow/BC/AIVPHL-
864/2022_HA|EPI2856394|DNA_INS"/>
NNN
</sequence>
<sequence>
<taxon idref="A/American_Crow/BC/AIVPHL-
874/2023|EPI_ISL_18665565|A_/_H5N1|Original||2.3.4.4b|09.01.2023|Caleta_Jes
sica_Marija|||21.12.2023|B.C._Centre_for_Disease_Control|Public_Health_Agen
cy_of_Canada|HA|4|A/American_Crow/BC/AIVPHL-
874/2023_HA|EPI2856448|DNA_INS"/>
NNN
</sequence>
<sequence>
<taxon idref="A/American_Crow/BC/AIVPHL-
900/2023|EPI_ISL_18665492|A_/_H5N1|Original||2.3.4.4b|16.01.2023|Caleta_Jes
sica_Marija|||21.12.2023|B.C._Centre_for_Disease_Control|Public_Health_Agen
cy_of_Canada|HA|4|A/American_Crow/BC/AIVPHL-
900/2023_HA|EPI2855906|DNA_INS"/>
NNN

</sequence>
<sequence>
<taxon idref="A/American_Crow/BC/AIVPHL-921/2023|EPI_ISL_18665496|A_/_H5N1|Original||2.3.4.4b|30.01.2023|Caleta_Jessica_Marija|||21.12.2023|B.C._Centre_for_Disease_Control|Public_Health_Agency_of_Canada|HA|4|A/American_Crow/BC/AIVPHL-921/2023_HA|EPI2855933|DNA_INS"/>
NNN
</sequence>
<sequence>
<taxon idref="A/American_Crow/BC/AIVPHL-922/2023|EPI_ISL_18665498|A_/_H5N1|Original||2.3.4.4b|30.01.2023|Caleta_Jessica_Marija|||21.12.2023|B.C._Centre_for_Disease_Control|Public_Health_Agency_of_Canada|HA|4|A/American_Crow/BC/AIVPHL-922/2023_HA|EPI2855949|DNA_INS"/>
NNN
</sequence>
<sequence>
<taxon idref="A/American_Crow/BC/AIVPHL-929/2023|EPI_ISL_18665506|A_/_H5N1|Original||2.3.4.4b|30.01.2023|Caleta_Jessica_Marija|||21.12.2023|B.C._Centre_for_Disease_Control|Public_Health_Agency_of_Canada|HA|4|A/American_Crow/BC/AIVPHL-929/2023_HA|EPI2856013|DNA_INS"/>
NNN
</sequence>
<sequence>
<taxon idref="A/American_Crow/BC/FAV-0053-11/2022|EPI_ISL_19155031|A_/_H5N1|||2.3.4.4b|02.12.2022|Signore_Anthony|||21.05.2024|Canadian_Food_Inspection_Agency|Canadian_Food_Inspection_Agency|HA|4|A/American_Crow/BC/FAV-0053-11/2022_HA|EPI3314928|DNA_IN"/>
NNN
</sequence>
<sequence>
<taxon idref="A/American_Crow/BC/FAV-0053-19/2022|EPI_ISL_19155032|A_/_H5N1|||2.3.4.4b|02.12.2022|Signore_Anthony|||21.05.2024|Canadian_Food_Inspection_Agency|Canadian_Food_Inspection_Agency|HA|4|A/American_Crow/BC/FAV-0053-19/2022_HA|EPI3314936|DNA_IN"/>
NNN
</sequence>
<sequence>
<taxon idref="A/American_Crow/BC/FAV-0053-21/2022|EPI_ISL_19155079|A_/_H5N1|||2.3.4.4b|11.12.2022|Signore_Anthony|||21.05.2024|Canadian_Food_Inspection_Agency|Canadian_Food_Inspection_Agency|HA|4|A/American_Crow/BC/FAV-0053-21/2022_HA|EPI3315312|DNA_IN"/>
NNN
</sequence>
<sequence>
<taxon idref="A/American_Crow/BC/FAV-0053-22/2022|EPI_ISL_19155033|A_/_H5N1|||2.3.4.4b|02.12.2022|Signore_Anthony|||21.05.2024|Canadian_Food_Inspection_Agency|Canadian_Food_Inspection_Agency|HA|4|A/American_Crow/BC/FAV-0053-22/2022_HA|EPI3314944|DNA_IN"/>
NNN
</sequence>
<sequence>
<taxon idref="A/American_Crow/BC/FAV-0053-24/2022|EPI_ISL_19155111|A_/_H5N1|||2.3.4.4b|16.12.2022|Signore_Anthony|||21.05.2024|Canadian_Food_Inspection_Agency|Canadian_Food_Inspection_Agency|HA|4|A/American_Crow/BC/FAV-0053-24/2022_HA|EPI3315568|DNA_IN"/>
NNN
</sequence>
<sequence>

<taxon idref="A/American_Crow/BC/FAV-0053-42/2023|EPI_ISL_19155140|A_/_H5N1|||2.3.4.4b|02.01.2023|Signore_Anthony|||21.05.2024|B.C._Centre_for_Disease_Control|Canadian_Food_Inspection_Agency|HA|4|A/American_Crow/BC/FAV-0053-42/2023_HA|EPI3315800|DNA_IN"/>
NNN
</sequence>
<sequence>
<taxon idref="A/American_crow/Iowa/23-007468-001/2023|EPI_ISL_17964890|A_/_H5N1|Original||2.3.4.4b|02.03.2023|Killian_Mary_Lea||23-007468-001|06.07.2023|National_Veterinary_Services_Laboratories_-_USDA|National_Veterinary_Services_Laboratories_-_USDA|HA|4|HA_A/Amer"/>
NNN
</sequence>
<sequence>
<taxon idref="A/American_crow/Iowa/23-007781-002/2023|EPI_ISL_17964893|A_/_H5N1|Original||2.3.4.4b|05.03.2023|Killian_Mary_Lea||23-007781-002|06.07.2023|National_Veterinary_Services_Laboratories_-_USDA|National_Veterinary_Services_Laboratories_-_USDA|HA|4|HA_A/Amer"/>
NNN
</sequence>
<sequence>
<taxon idref="A/American_crow/Utah/23-007867-006/2023|EPI_ISL_17964896|A_/_H5N1|Original||2.3.4.4b|03.03.2023|Killian_Mary_Lea||23-007867-006|06.07.2023|National_Veterinary_Services_Laboratories_-_USDA|National_Veterinary_Services_Laboratories_-_USDA|HA|4|HA_A/Amer"/>
NNN
</sequence>
<sequence>
<taxon idref="A/Antarctic_Fur_Seal/Jason_Harbour/141037/2023|EPI_ISL_18742212|A_/_H5N1|Clinical_Sample||2.3.4.4b|09.12.2023|Mollett_Benjamin|||25.06.2024|Animal_and_Plant_Health_Agency__APHA_|Animal_and_Plant_Health_Agency__APHA_|HA|4|A/Antarctic_Fur_Seal/Jason_Har"/>
NNN
</sequence>
<sequence>
<taxon idref="A/Antarctic_Fur_Seal/South_Georgia_and_the_South_Sandwich_Islands/128/2023|EPI_ISL_19313608|A_/_H5N1|Original||2.3.4.4b|09.12.2023||||06.08.2024|Animal_and_Plant_Health_Agency__APHA_||HA|4|A/Antarctic_Fur_Seal/South_Georgia_and_the_South_Sandwich_Islan"/>
NNN
</sequence>
<sequence>
<taxon idref="A/Antarctic_Tern/South_Georgia_and_the_South_Sandwich_Islands/40/2023|EPI_ISL_19313616|A_/_H5N1|Original||2.3.4.4b|06.12.2023||||06.08.2024|Animal_and_Plant_Health_Agency__APHA_||HA|4|A/Antarctic_Tern/South_Georgia_and_the_South_Sandwich_Islands/40/202"/>
NNN
</sequence>
<sequence>
<taxon idref="A/Antarctic_tern/King_Edward_Point/141271/2023|EPI_ISL_18742220|A_/_H5N1|Clinical_Sample||2.3.4.4b|06.12.2023|Mollett_Benjamin|||25.06.2024|Animal_and_Plant_Health_Agency__APHA_|Animal_and_Plant_Health_Agency__APHA_|HA|4|A/Antarctic_tern/King_Edward_P"/>
NNN
</sequence>
<sequence>

<taxon idref="A/Antofagasta/25945/2023|EPI_ISL_19391463|A_/_H5N1|Original||
2.3.4.4b|24.03.2023||||09.09.2024|Instituto_de_Salud_Publica_de_Chile||HA|4
|A/Antofagasta/25945/2023_HA|EPI3549652|DNA_IN"/>
NNN
</sequence>
<sequence>
<taxon idref="A/Avian/Argentina/1762-
2/2023|EPI_ISL_18698516|A_/_H5N1|||2.3.4.4b|21.04.2023|Benedetti_Estefania|
||28.12.2023|Direccion_del_Laboratorio_Animal_Direccion_General_de_Laborato
rios_y_Control_Tecnico_SENASA.|Instituto_Nacional_de_Enfermedades_Infeccios
a"/>
NNN
</sequence>
<sequence>
<taxon idref="A/Avian/Argentina/1790-
5/2023|EPI_ISL_18698517|A_/_H5N1|||2.3.4.4b|24.04.2023|Benedetti_Estefania|
||28.12.2023|Direccion_del_Laboratorio_Animal_Direccion_General_de_Laborato
rios_y_Control_Tecnico_SENASA.|Instituto_Nacional_de_Enfermedades_Infeccios
a"/>
NNN
</sequence>
<sequence>
<taxon idref="A/Avian/Argentina/579-
5/2023|EPI_ISL_18698485|A_/_H5N1|||2.3.4.4b|24.02.2023|Benedetti_Estefania|
||28.12.2023|Direccion_del_Laboratorio_Animal_Direccion_General_de_Laborato
rios_y_Control_Tecnico_SENASA.|Instituto_Nacional_de_Enfermedades_Infeccios
as"/>
NNN
</sequence>
<sequence>
<taxon idref="A/Avian/Argentina/586-
4/2023|EPI_ISL_18698487|A_/_H5N1|||2.3.4.4b|24.02.2023|Benedetti_Estefania|
||28.12.2023|Direccion_del_Laboratorio_Animal_Direccion_General_de_Laborato
rios_y_Control_Tecnico_SENASA.|Instituto_Nacional_de_Enfermedades_Infeccios
as"/>
NNN
</sequence>
<sequence>
<taxon idref="A/Band-tailed_gull/Antofagasta/228525-
1/2022|EPI_ISL_19131166|A_/_H5N1|Original||2.3.4.4b|19.12.2022||||10.05.202
4|Emory_University__CEIRR_|Emory_University__CEIRR_|HA|4|A/Band-
tailed_gull/Antofagasta/228525-1/2022_HA|EPI3276855|DNA_IN"/>
NNN
</sequence>
<sequence>
<taxon idref="A/Band-tailed_gull/Arica_y_Parinacota/232200-
1/2023|EPI_ISL_19131168|A_/_H5N1|Original||2.3.4.4b|11.01.2023||||10.05.202
4|Emory_University__CEIRR_|Emory_University__CEIRR_|HA|4|A/Band-
tailed_gull/Arica_y_Parinacota/232200-1/2023_HA|EPI3276870|DNA_IN"/>
NNN
</sequence>
<sequence>
<taxon idref="A/Barn_Owl/BC/AIVPHL-
1011/2023|EPI_ISL_18665563|A_/_H5N1|Original||2.3.4.4b|20.03.2023|Caleta_Je
ssica_Marija|||21.12.2023|B.C._Centre_for_Disease_Control|Public_Health_Age
ncy_of_Canada|HA|4|A/Barn_Owl/BC/AIVPHL-1011/2023_HA|EPI2856439|DNA_INS"/>
NNN
</sequence>
<sequence>
<taxon idref="A/Barn_Owl/BC/AIVPHL-
926/2023|EPI_ISL_18665504|A_/_H5N1|Original||2.3.4.4b|30.01.2023|Caleta_Jes

sica_Marija|||21.12.2023|B.C._Centre_for_Disease_Control|Public_Health_Agency_of_Canada|HA|4|A/Barn_Owl/BC/AIVPHL-926/2023_HA|EPI2855997|DNA_INS"/>
NNN
</sequence>
<sequence>
<taxon idref="A/Barn_Owl/BC/AIVPHL-995/2023|EPI_ISL_18665547|A_/_H5N1|Original||2.3.4.4b|13.03.2023|Caleta_Jessica_Marija|||21.12.2023|B.C._Centre_for_Disease_Control|Public_Health_Agency_of_Canada|HA|4|A/Barn_Owl/BC/AIVPHL-995/2023_HA|EPI2856323|DNA_INS"/>
NNN
</sequence>
<sequence>
<taxon idref="A/Barred_Owl/BC/AIVPHL-1008/2023|EPI_ISL_18665556|A_/_H5N1|Original||2.3.4.4b|20.03.2023|Caleta_Jessica_Marija|||21.12.2023|B.C._Centre_for_Disease_Control|Public_Health_Agency_of_Canada|HA|4|A/Barred_Owl/BC/AIVPHL-1008/2023_HA|EPI2856390|DNA_INS"/>
NNN
</sequence>
<sequence>
<taxon idref="A/Barred_Owl/BC/AIVPHL-982/2023|EPI_ISL_18665541|A_/_H5N1|Original||2.3.4.4b|10.03.2023|Caleta_Jessica_Marija|||21.12.2023|B.C._Centre_for_Disease_Control|Public_Health_Agency_of_Canada|HA|4|A/Barred_Owl/BC/AIVPHL-982/2023_HA|EPI2856275|DNA_INS"/>
NNN
</sequence>
<sequence>
<taxon idref="A/Barred_Owl/BC/FAV-0053-23/2022|EPI_ISL_19155098|A_/_H5N1|||2.3.4.4b|13.12.2022|Signore_Anthony|||21.05.2024|Canadian_Food_Inspection_Agency|Canadian_Food_Inspection_Agency|HA|4|A/Barred_Owl/BC/FAV-0053-23/2022_HA|EPI3315464|DNA_IN"/>
NNN
</sequence>
<sequence>
<taxon idref="A/Black-crowned_night-heron/Antofagasta/228705-2/2022|EPI_ISL_18005786|A_/_H5N1|Original||2.3.4.4b|20.12.2022|GISAID_EpiFlu_Data_Curator|||19.07.2023|CEIRS_Data_Processing_and_Coordinating_Center_Center_for_Research_on_Influenza_Pathogenesis__CRIP_|C"/>
NNN
</sequence>
<sequence>
<taxon idref="A/Black_Skimmer/Maule/240379/2023|EPI_ISL_17885849|A_/_H5N1|Original||2.3.4.4b|06.03.2023|GISAID_EpiFlu_Data_Curator|||19.07.2023|CEIRS_Data_Processing_and_Coordinating_Center_Center_for_Research_on_Influenza_Pathogenesis__CRIP_|CEIRS_Data_Processing"/>
NNN
</sequence>
<sequence>
<taxon idref="A/Blackish_oystercatcher/OHiggins/240628/2023|EPI_ISL_17885967|A_/_H5N1|Original||2.3.4.4b|06.03.2023|GISAID_EpiFlu_Data_Curator|||24.07.2023|CEIRS_Data_Processing_and_Coordinating_Center_Center_for_Research_on_Influenza_Pathogenesis__CRIP_|CEIRS_Dat"/>
NNN
</sequence>
<sequence>
<taxon idref="A/Broiler_Breeders_Chicken/BC/FAV-1616-1/2022|EPI_ISL_19155042|A_/_H5N1|||2.3.4.4b|05.12.2022|Signore_Anthony|||21.05.2024|Canadian_Food_Inspection_Agency|Canadian_Food_Inspection_Agency|HA|4|A/Broiler_Breeders_Chicken/BC/FAV-1616-1/2022_HA|EPI3315016"/>
NNN
</sequence>

<sequence>
<taxon idref="A/Broiler_Breeders_Chicken/BC/FAV-1616-
2/2022|EPI_ISL_19155043|A_/_H5N1|||2.3.4.4b|05.12.2022|Signore_Anthony|||21
.05.2024|Canadian_Food_Inspection_Agency|Canadian_Food_Inspection_Agency|HA
|4|A/Broiler_Breeders_Chicken/BC/FAV-1616-2/2022_HA|EPI3315024"/>
NNN
</sequence>
<sequence>
<taxon idref="A/Brown_Skua/Bird_Island/141232/2023|EPI_ISL_18742214|A_/_H5N
1|Clinical_Sample||2.3.4.4b|11.11.2023|Mollett_Benjamin|||25.06.2024|Animal
_and_Plant_Health_Agency__APHA_|Animal_and_Plant_Health_Agency__APHA_|HA|4|
A/Brown_Skua/Bird_Island/141232/2023|HA|"/>
NNN
</sequence>
<sequence>
<taxon idref="A/Brown_Skua/Hound_Bay/133949/2023|EPI_ISL_18592429|A_/_H5N1|
Egg_Passage_1||2.3.4.4b|30.10.2023|Mollett_Benjamin|||06.12.2023|Animal_and
_Plant_Health_Agency__APHA_|Animal_and_Plant_Health_Agency__APHA_|HA|4|A/Br
own_Skua/Hound_Bay/133949/2023|HA|EPI281"/>
NNN
</sequence>
<sequence>
<taxon idref="A/Brown_Skua/Moltke_Harbour/133752/2023|EPI_ISL_18592424|A_/_
H5N1|Clinical_Sample||2.3.4.4b|31.10.2023|Mollett_Benjamin|||06.12.2023|Ani
mal_and_Plant_Health_Agency__APHA_|Animal_and_Plant_Health_Agency__APHA_|HA
|4|A/Brown_Skua/Moltke_Harbour/133752/20"/>
NNN
</sequence>
<sequence>
<taxon idref="A/Brown_Skua/Moltke_Harbour/133755/2023|EPI_ISL_18592426|A_/_
H5N1|Clinical_Sample||2.3.4.4b|31.10.2023|Mollett_Benjamin|||06.12.2023|Ani
mal_and_Plant_Health_Agency__APHA_|Animal_and_Plant_Health_Agency__APHA_|HA
|4|A/Brown_Skua/Moltke_Harbour/133755/20"/>
NNN
</sequence>
<sequence>
<taxon idref="A/Brown_Skua/Penguin_River/141236/2023|EPI_ISL_18742216|A_/_H
5N1|Clinical_Sample||2.3.4.4b|08.11.2023|Mollett_Benjamin|||25.06.2024|Anim
al_and_Plant_Health_Agency__APHA_|Animal_and_Plant_Health_Agency__APHA_|HA|
4|A/Brown_Skua/Penguin_River/141236/2023"/>
NNN
</sequence>
<sequence>
<taxon idref="A/Brown_Skua/Penguin_River/141240/2023|EPI_ISL_18742218|A_/_H
5N1|Clinical_Sample||2.3.4.4b|08.11.2023|Mollett_Benjamin|||25.06.2024|Anim
al_and_Plant_Health_Agency__APHA_|Animal_and_Plant_Health_Agency__APHA_|HA|
4|A/Brown_Skua/Penguin_River/141240/2023"/>
NNN
</sequence>
<sequence>
<taxon idref="A/Brown_Skua/South_Georgia_and_the_South_Sandwich_Islands/104
/2023|EPI_ISL_19313621|A_/_H5N1|Original||2.3.4.4b|15.11.2023||||06.08.2024
|Animal_and_Plant_Health_Agency__APHA_||HA|4|A/Brown_Skua/South_Georgia_and
_the_South_Sandwich_Islands/104/2023_HA|E"/>
NNN
</sequence>
<sequence>
<taxon idref="A/Brown_Skua/South_Georgia_and_the_South_Sandwich_Islands/112
/2023|EPI_ISL_19313575|A_/_H5N1|Original||2.3.4.4b|30.10.2023||||06.08.2024
|Animal_and_Plant_Health_Agency__APHA_||HA|4|A/Brown_Skua/South_Georgia_and
_the_South_Sandwich_Islands/112/2023_HA|E"/>

NNN
</sequence>
<sequence>
<taxon idref="A/Brown_Skua/South_Georgia_and_the_South_Sandwich_Islands/144/2023|EPI_ISL_19313583|A_/_H5N1|Original||2.3.4.4b|11.11.2023||||06.08.2024|Animal_and_Plant_Health_Agency__APHA_||HA|4|A/Brown_Skua/South_Georgia_and_the_South_Sandwich_Islands/144/2023_HA|E"/>
NNN
</sequence>
<sequence>
<taxon idref="A/Brown_Skua/South_Georgia_and_the_South_Sandwich_Islands/16/2023|EPI_ISL_19321280|A_/_H5N1|Original||2.3.4.4b|08.10.2023||||08.08.2024|Animal_and_Plant_Health_Agency__APHA_||HA|4|A/Brown_Skua/South_Georgia_and_the_South_Sandwich_Islands/16/2023_HA|EPI"/>
NNN
</sequence>
<sequence>
<taxon idref="A/Brown_Skua/South_Georgia_and_the_South_Sandwich_Islands/24/2023|EPI_ISL_19321271|A_/_H5N1|Original||2.3.4.4b|08.10.2023||||08.08.2024|Animal_and_Plant_Health_Agency__APHA_||HA|4|A/Brown_Skua/South_Georgia_and_the_South_Sandwich_Islands/24/2023_HA|EPI"/>
NNN
</sequence>
<sequence>
<taxon idref="A/Brown_Skua/South_Georgia_and_the_South_Sandwich_Islands/48/2023|EPI_ISL_19313591|A_/_H5N1|Original||2.3.4.4b|31.10.2023||||06.08.2024|Animal_and_Plant_Health_Agency__APHA_||HA|4|A/Brown_Skua/South_Georgia_and_the_South_Sandwich_Islands/48/2023_HA|EPI"/>
NNN
</sequence>
<sequence>
<taxon idref="A/Brown_Skua/South_Georgia_and_the_South_Sandwich_Islands/64/2023|EPI_ISL_19313626|A_/_H5N1|Original||2.3.4.4b|31.10.2023||||06.08.2024|Animal_and_Plant_Health_Agency__APHA_||HA|4|A/Brown_Skua/South_Georgia_and_the_South_Sandwich_Islands/64/2023_HA|EPI"/>
NNN
</sequence>
<sequence>
<taxon idref="A/Brown_Skua/South_Georgia_and_the_South_Sandwich_Islands/8/2023|EPI_ISL_19321287|A_/_H5N1|Original||2.3.4.4b|08.10.2023||||08.08.2024|Animal_and_Plant_Health_Agency__APHA_||HA|4|A/Brown_Skua/South_Georgia_and_the_South_Sandwich_Islands/8/2023_HA|EPI34"/>
NNN
</sequence>
<sequence>
<taxon idref="A/Brown_Skua/South_Georgia_and_the_South_Sandwich_Islands/80/2023|EPI_ISL_19313660|A_/_H5N1|Original||2.3.4.4b|08.11.2023||||06.08.2024|Animal_and_Plant_Health_Agency__APHA_||HA|4|A/Brown_Skua/South_Georgia_and_the_South_Sandwich_Islands/80/2023_HA|EPI"/>
NNN
</sequence>
<sequence>
<taxon idref="A/Brown_Skua/South_Georgia_and_the_South_Sandwich_Islands/96/2023|EPI_ISL_19313668|A_/_H5N1|Original||2.3.4.4b|30.10.2023||||06.08.2024|Animal_and_Plant_Health_Agency__APHA_||HA|4|A/Brown_Skua/South_Georgia_and_the_South_Sandwich_Islands/96/2023_HA|EPI"/>
NNN
</sequence>
<sequence>
<taxon idref="A/Brown_skua/Bird_Island/128287/2023|EPI_ISL_18439562|A_/_H5N1|Clinical_Sample||2.3.4.4b|08.10.2023|Byrne_Alex|||27.10.2023|Animal_and_P

lant_Health_Agency__APHA_|Animal_and_Plant_Health_Agency__APHA_|HA|4|A/Brown_skua/Bird_Island/128287/2023|HA|EPI278"/>
NNN
</sequence>
<sequence>
<taxon idref="A/Brown_skua/Bird_Island/128288/2023|EPI_ISL_18439563|A_/_H5N1|Clinical_Sample||2.3.4.4b|08.10.2023|Byrne_Alex|||27.10.2023|Animal_and_Plant_Health_Agency__APHA_|Animal_and_Plant_Health_Agency__APHA_|HA|4|A/Brown_skua/Bird_Island/128288/2023|HA|EPI278"/>
NNN
</sequence>
<sequence>
<taxon idref="A/Brown_skua/Bird_Island/128289/2023|EPI_ISL_18439564|A_/_H5N1|Clinical_Sample||2.3.4.4b|08.10.2023|Byrne_Alex|||27.10.2023|Animal_and_Plant_Health_Agency__APHA_|Animal_and_Plant_Health_Agency__APHA_|HA|4|A/Brown_skua/Bird_Island/128289/2023|HA|EPI278"/>
NNN
</sequence>
<sequence>
<taxon idref="A/Cackling_Goose/BC/AIVPHL-
875/2023|EPI_ISL_18665568|A_/_H5N1|Original||2.3.4.4b|09.01.2023|Caleta_Jessica_Marija|||21.12.2023|B.C._Centre_for_Disease_Control|Public_Health_Agency_of_Canada|HA|4|A/Cackling_Goose/BC/AIVPHL-
875/2023_HA|EPI2856470|DNA_IN"/>
NNN
</sequence>
<sequence>
<taxon idref="A/Cackling_Goose/BC/AIVPHL-
876/2023|EPI_ISL_18665571|A_/_H5N1|Original||2.3.4.4b|09.01.2023|Caleta_Jessica_Marija|||21.12.2023|B.C._Centre_for_Disease_Control|Public_Health_Agency_of_Canada|HA|4|A/Cackling_Goose/BC/AIVPHL-
876/2023_HA|EPI2856496|DNA_IN"/>
NNN
</sequence>
<sequence>
<taxon idref="A/Calidris_alba/Lima/HA_4/2023|EPI_ISL_19158339|A_/_H5N1|Original||2.3.4.4b|20.04.2023||||22.05.2024|National_University_of_San_Marcos_Biology_Sciences||HA|4|A/Calidris_alba/Lima/HA_4/2023_HA|EPI3324598|DNA_IN"/>
NNN
</sequence>
<sequence>
<taxon idref="A/Calidris_alba/Lima/Villa01/2023___this_|EPI_ISL_19070288|A_/_H5N1|||2.3.4.4b|20.04.2023|Landazabal_Castillo_Sandra_Liliana|||24.04.2024|Molecular_and_Clinical_Virology_Laboratory_National_University_of_San_Marcos|National_University_of_San_Marcos|HA"/>
NNN
</sequence>
<sequence>
<taxon idref="A/Canada_Goose/BC/AIVPHL-
844/2022|EPI_ISL_18665543|A_/_H5N1|Original||2.3.4.4b|16.12.2022|Caleta_Jessica_Marija|||21.12.2023|B.C._Centre_for_Disease_Control|Public_Health_Agency_of_Canada|HA|4|A/Canada_Goose/BC/AIVPHL-
844/2022_HA|EPI2856282|DNA_INS"/>
NNN
</sequence>
<sequence>
<taxon idref="A/Canada_goose/Iowa/23-028688-001-
original/2023|EPI_ISL_19371749|A_/_H5N1|original||2.3.4.4b|13.01.2023|Killian_Mary_Lea||23-028688-

001|31.08.2024|National_Veterinary_Services_Laboratories_-
_USDA|National_Veterinary_Services_Laboratories_-_USDA|HA|4|H"/>
NNN
</sequence>
<sequence>
<taxon idref="A/Canada_goose/Iowa/23-030290-
001/2022|EPI_ISL_19131437|A_/_H5N1|Original||2.3.4.4b|07.12.2022|Killian_Ma
ry_Lea||23-030290-
001|12.12.2024|National_Veterinary_Services_Laboratories_-
_USDA|National_Veterinary_Services_Laboratories_-_USDA|HA|4|HA_A/Canad"/>
NNN
</sequence>
<sequence>
<taxon idref="A/Canada_goose/Washington/23-007799-
001/2023|EPI_ISL_17964894|A_/_H5N1|Original||2.3.4.4b|27.02.2023|Killian_Ma
ry_Lea||23-007799-
001|07.07.2023|National_Veterinary_Services_Laboratories_-
_USDA|National_Veterinary_Services_Laboratories_-_USDA|HA|4|HA_A"/>
NNN
</sequence>
<sequence>
<taxon idref="A/Caspian_tern/Oregon/23-018836-
001/2023|EPI_ISL_18311014|A_/_H5N1|Original||2.3.4.4b|15.06.2023|Killian_Ma
ry_Lea||23-018836-
001|26.09.2023|National_Veterinary_Services_Laboratories_-
_USDA|National_Veterinary_Services_Laboratories_-_USDA|HA|4|HA_A/Cas"/>
NNN
</sequence>
<sequence>
<taxon idref="A/Caspian_tern/Oregon/23-018836-
002/2023|EPI_ISL_18311015|A_/_H5N1|Original||2.3.4.4b|15.06.2023|Killian_Ma
ry_Lea||23-018836-
002|26.09.2023|National_Veterinary_Services_Laboratories_-
_USDA|National_Veterinary_Services_Laboratories_-_USDA|HA|4|HA_A/Cas"/>
NNN
</sequence>
<sequence>
<taxon idref="A/Caspian_tern/Oregon/23-018836-
003/2023|EPI_ISL_18311016|A_/_H5N1|Original||2.3.4.4b|15.06.2023|Killian_Ma
ry_Lea||23-018836-
003|26.09.2023|National_Veterinary_Services_Laboratories_-
_USDA|National_Veterinary_Services_Laboratories_-_USDA|HA|4|HA_A/Cas"/>
NNN
</sequence>
<sequence>
<taxon idref="A/Caspian_tern/Washington/23-021119-
003/2023|EPI_ISL_18311017|A_/_H5N1|Original||2.3.4.4b|11.07.2023|Killian_Ma
ry_Lea||23-021119-
003|26.09.2023|National_Veterinary_Services_Laboratories_-
_USDA|National_Veterinary_Services_Laboratories_-_USDA|HA|4|HA_A"/>
NNN
</sequence>
<sequence>
<taxon idref="A/Caspian_tern/Washington/23-023703-
001/2023|EPI_ISL_18311018|A_/_H5N1|Original||2.3.4.4b|31.07.2023|Killian_Ma
ry_Lea||23-023703-
001|26.09.2023|National_Veterinary_Services_Laboratories_-
_USDA|National_Veterinary_Services_Laboratories_-_USDA|HA|4|HA_A"/>
NNN
</sequence>
<sequence>

<taxon idref="A/Caspian_tern/Washington/23-023703-
002/2023|EPI_ISL_18311019|A_/_H5N1|Original||2.3.4.4b|31.07.2023|Killian_Ma
ry_Lea||23-023703-
002|26.09.2023|National_Veterinary_Services_Laboratories_-
_USDA|National_Veterinary_Services_Laboratories_-_USDA|HA|4|HA_A"/>
NNN
</sequence>
<sequence>
<taxon idref="A/Caspian_tern/Washington/W231930045-
3/2023|EPI_ISL_19592591|A_/_H5N1|Original||2.3.4.4b|10.07.2023|||06.12.202
4|Washington_State_University||HA|4|A/Caspian_tern/Washington/W231930045-
3/2023_HA|EPI3675293|DNA_IN"/>
NNN
</sequence>
<sequence>
<taxon idref="A/Caspian_tern/Washington/W232140074-
1/2023|EPI_ISL_19592592|A_/_H5N1|Original||2.3.4.4b|31.07.2023|||06.12.202
4|Washington_State_University||HA|4|A/Caspian_tern/Washington/W232140074-
1/2023_HA|EPI3675301|DNA_IN"/>
NNN
</sequence>
<sequence>
<taxon idref="A/Caspian_tern/Washington/W232140074-
2/2023|EPI_ISL_19592593|A_/_H5N1|Original||2.3.4.4b|31.07.2023|||06.12.202
4|Washington_State_University||HA|4|A/Caspian_tern/Washington/W232140074-
2/2023_HA|EPI3675309|DNA_IN"/>
NNN
</sequence>
<sequence>
<taxon idref="A/Caspian_tern/Washington/W232270041-
1/2023|EPI_ISL_19592594|A_/_H5N1|Original||2.3.4.4b|11.08.2023|||06.12.202
4|Washington_State_University||HA|4|A/Caspian_tern/Washington/W232270041-
1/2023_HA|EPI3675317|DNA_IN"/>
NNN
</sequence>
<sequence>
<taxon idref="A/Chicken/Argentina/1035-
1/2023|EPI_ISL_18698504|A_/_H5N1|||2.3.4.4b|16.03.2023|Benedetti_Estefania|
||28.12.2023|Direccion_del_Laboratorio_Animal_Direccion_General_de_Laborato
rios_y_Control_Tecnico_SENASA.|Instituto_Nacional_de_Enfermedades_Infeccio"
/>
NNN
</sequence>
<sequence>
<taxon idref="A/Chicken/Argentina/1147-
2/2023|EPI_ISL_18698505|A_/_H5N1|||2.3.4.4b|21.03.2023|Benedetti_Estefania|
||28.12.2023|Direccion_del_Laboratorio_Animal_Direccion_General_de_Laborato
rios_y_Control_Tecnico_SENASA.|Instituto_Nacional_de_Enfermedades_Infeccio"
/>
NNN
</sequence>
<sequence>
<taxon idref="A/Chicken/Argentina/1200-
1/2023|EPI_ISL_18698506|A_/_H5N1|||2.3.4.4b|27.03.2023|Benedetti_Estefania|
||28.12.2023|Direccion_del_Laboratorio_Animal_Direccion_General_de_Laborato
rios_y_Control_Tecnico_SENASA.|Instituto_Nacional_de_Enfermedades_Infeccio"
/>
NNN
</sequence>
<sequence>

<taxon idref="A/Chicken/Argentina/1340-
2/2023|EPI_ISL_18698507|A_/_H5N1|||2.3.4.4b|31.03.2023|Benedetti_Estefania|
||28.12.2023|Direccion_del_Laboratorio_Animal_Direccion_General_de_Laborato
rios_y_Control_Tecnico_SENASA.|Instituto_Nacional_de_Enfermedades_Infeccio"
/>
NNN
</sequence>
<sequence>
<taxon idref="A/Chicken/Argentina/1375-
6/2023|EPI_ISL_18698509|A_/_H5N1|||2.3.4.4b|03.04.2023|Benedetti_Estefania|
||28.12.2023|Direccion_del_Laboratorio_Animal_Direccion_General_de_Laborato
rios_y_Control_Tecnico_SENASA.|Instituto_Nacional_de_Enfermedades_Infeccio"
/>
NNN
</sequence>
<sequence>
<taxon idref="A/Chicken/Argentina/1416-
3/2023|EPI_ISL_18698510|A_/_H5N1|||2.3.4.4b|03.04.2023|Benedetti_Estefania|
||28.12.2023|Direccion_del_Laboratorio_Animal_Direccion_General_de_Laborato
rios_y_Control_Tecnico_SENASA.|Instituto_Nacional_de_Enfermedades_Infeccio"
/>
NNN
</sequence>
<sequence>
<taxon idref="A/Chicken/Argentina/1495-
4/2023|EPI_ISL_18698719|A_/_H5N1|||2.3.4.4b|10.04.2023|Benedetti_Estefania|
||28.12.2023|Direccion_del_Laboratorio_Animal_Direccion_General_de_Laborato
rios_y_Control_Tecnico_SENASA.|Instituto_Nacional_de_Enfermedades_Infeccio"
/>
NNN
</sequence>
<sequence>
<taxon idref="A/Chicken/Argentina/1530-
3/2023|EPI_ISL_18698511|A_/_H5N1|||2.3.4.4b|12.04.2023|Benedetti_Estefania|
||28.12.2023|Direccion_del_Laboratorio_Animal_Direccion_General_de_Laborato
rios_y_Control_Tecnico_SENASA.|Instituto_Nacional_de_Enfermedades_Infeccio"
/>
NNN
</sequence>
<sequence>
<taxon idref="A/Chicken/Argentina/1708-
1/2023|EPI_ISL_18698512|A_/_H5N1|||2.3.4.4b|19.04.2023|Benedetti_Estefania|
||28.12.2023|Direccion_del_Laboratorio_Animal_Direccion_General_de_Laborato
rios_y_Control_Tecnico_SENASA.|Instituto_Nacional_de_Enfermedades_Infeccio"
/>
NNN
</sequence>
<sequence>
<taxon idref="A/Chicken/Argentina/1976-
2/2023|EPI_ISL_18698518|A_/_H5N1|||2.3.4.4b|01.05.2023|Benedetti_Estefania|
||28.12.2023|Direccion_del_Laboratorio_Animal_Direccion_General_de_Laborato
rios_y_Control_Tecnico_SENASA.|Instituto_Nacional_de_Enfermedades_Infeccio"
/>
NNN
</sequence>
<sequence>
<taxon idref="A/Chicken/Argentina/1984-
4/2023|EPI_ISL_18698519|A_/_H5N1|||2.3.4.4b|04.05.2023|Benedetti_Estefania|
||28.12.2023|Direccion_del_Laboratorio_Animal_Direccion_General_de_Laborato
rios_y_Control_Tecnico_SENASA.|Instituto_Nacional_de_Enfermedades_Infeccio"
/>

NNN
</sequence>
<sequence>
<taxon idref="A/Chicken/Argentina/2016-
2/2023|EPI_ISL_18698732|A_/_H5N1|||2.3.4.4b|03.05.2023|Benedetti_Estefania|
||28.12.2023|Direccion_del_Laboratorio_Animal_Direccion_General_de_Laborato
rios_y_Control_Tecnico_SENASA.|Instituto_Nacional_de_Enfermedades_Infeccio"
/>
NNN
</sequence>
<sequence>
<taxon idref="A/Chicken/Argentina/2034-
5/2023|EPI_ISL_18698520|A_/_H5N1|||2.3.4.4b|05.05.2023|Benedetti_Estefania|
||28.12.2023|Direccion_del_Laboratorio_Animal_Direccion_General_de_Laborato
rios_y_Control_Tecnico_SENASA.|Instituto_Nacional_de_Enfermedades_Infeccio"
/>
NNN
</sequence>
<sequence>
<taxon idref="A/Chicken/Argentina/2049-
3/2023|EPI_ISL_18698521|A_/_H5N1|||2.3.4.4b|08.05.2023|Benedetti_Estefania|
||28.12.2023|Direccion_del_Laboratorio_Animal_Direccion_General_de_Laborato
rios_y_Control_Tecnico_SENASA.|Instituto_Nacional_de_Enfermedades_Infeccio"
/>
NNN
</sequence>
<sequence>
<taxon idref="A/Chicken/Argentina/2064-
3/2023|EPI_ISL_18698522|A_/_H5N1|||2.3.4.4b|08.05.2023|Benedetti_Estefania|
||28.12.2023|Direccion_del_Laboratorio_Animal_Direccion_General_de_Laborato
rios_y_Control_Tecnico_SENASA.|Instituto_Nacional_de_Enfermedades_Infeccio"
/>
NNN
</sequence>
<sequence>
<taxon idref="A/Chicken/Argentina/2305-
1/2023|EPI_ISL_18698524|A_/_H5N1|||2.3.4.4b|18.05.2023|Benedetti_Estefania|
||28.12.2023|Direccion_del_Laboratorio_Animal_Direccion_General_de_Laborato
rios_y_Control_Tecnico_SENASA.|Instituto_Nacional_de_Enfermedades_Infeccio"
/>
NNN
</sequence>
<sequence>
<taxon idref="A/Chicken/Argentina/2305-
6/2023|EPI_ISL_18698525|A_/_H5N1|||2.3.4.4b|18.05.2023|Benedetti_Estefania|
||28.12.2023|Direccion_del_Laboratorio_Animal_Direccion_General_de_Laborato
rios_y_Control_Tecnico_SENASA.|Instituto_Nacional_de_Enfermedades_Infeccio"
/>
NNN
</sequence>
<sequence>
<taxon idref="A/Chicken/Argentina/2483-
3/2023|EPI_ISL_18698526|A_/_H5N1|||2.3.4.4b|29.05.2023|Benedetti_Estefania|
||28.12.2023|Direccion_del_Laboratorio_Animal_Direccion_General_de_Laborato
rios_y_Control_Tecnico_SENASA.|Instituto_Nacional_de_Enfermedades_Infeccio"
/>
NNN
</sequence>
<sequence>
<taxon idref="A/Chicken/Argentina/2796-
2/2023|EPI_ISL_18698527|A_/_H5N1|||2.3.4.4b|13.06.2023|Benedetti_Estefania|

||28.12.2023|Direccion_del_Laboratorio_Animal_Direccion_General_de_Laboratorios_y_Control_Tecnico_SENASA.|Instituto_Nacional_de_Enfermedades_Infeccio"
/>
NNN
</sequence>
<sequence>
<taxon idref="A/Chicken/Argentina/3346-1/2023|EPI_ISL_18698528|A_/_H5N1|||2.3.4.4b|06.07.2023|Benedetti_Estefania|
||28.12.2023|Direccion_del_Laboratorio_Animal_Direccion_General_de_Laboratorios_y_Control_Tecnico_SENASA.|Instituto_Nacional_de_Enfermedades_Infeccio"
/>
NNN
</sequence>
<sequence>
<taxon idref="A/Chicken/Argentina/3695-3/2023|EPI_ISL_18698530|A_/_H5N1|||2.3.4.4b|27.07.2023|Benedetti_Estefania|
||28.12.2023|Direccion_del_Laboratorio_Animal_Direccion_General_de_Laboratorios_y_Control_Tecnico_SENASA.|Instituto_Nacional_de_Enfermedades_Infeccio"
/>
NNN
</sequence>
<sequence>
<taxon idref="A/Chicken/Argentina/464-4/2023|EPI_ISL_18698460|A_/_H5N1|||2.3.4.4b|16.02.2023|Benedetti_Estefania|
||28.12.2023|Direccion_del_Laboratorio_Animal_Direccion_General_de_Laboratorios_y_Control_Tecnico_SENASA.|Instituto_Nacional_de_Enfermedades_Infeccios
"/>
NNN
</sequence>
<sequence>
<taxon idref="A/Chicken/Argentina/477-5/2023|EPI_ISL_18698462|A_/_H5N1|||2.3.4.4b|18.02.2023|Benedetti_Estefania|
||28.12.2023|Direccion_del_Laboratorio_Animal_Direccion_General_de_Laboratorios_y_Control_Tecnico_SENASA.|Instituto_Nacional_de_Enfermedades_Infeccios
"/>
NNN
</sequence>
<sequence>
<taxon idref="A/Chicken/Argentina/481-2/2023|EPI_ISL_18698464|A_/_H5N1|||2.3.4.4b|18.02.2023|Benedetti_Estefania|
||28.12.2023|Direccion_del_Laboratorio_Animal_Direccion_General_de_Laboratorios_y_Control_Tecnico_SENASA.|Instituto_Nacional_de_Enfermedades_Infeccios
"/>
NNN
</sequence>
<sequence>
<taxon idref="A/Chicken/Argentina/485-6/2023|EPI_ISL_18698466|A_/_H5N1|||2.3.4.4b|19.02.2023|Benedetti_Estefania|
||28.12.2023|Direccion_del_Laboratorio_Animal_Direccion_General_de_Laboratorios_y_Control_Tecnico_SENASA.|Instituto_Nacional_de_Enfermedades_Infeccios
"/>
NNN
</sequence>
<sequence>
<taxon idref="A/Chicken/Argentina/491-2/2023|EPI_ISL_18698469|A_/_H5N1|||2.3.4.4b|20.02.2023|Benedetti_Estefania|
||28.12.2023|Direccion_del_Laboratorio_Animal_Direccion_General_de_Laboratorios_y_Control_Tecnico_SENASA.|Instituto_Nacional_de_Enfermedades_Infeccios
"/>
NNN
</sequence>

<sequence>
<taxon idref="A/Chicken/Argentina/501-
1/2023|EPI_ISL_18698471|A_/_H5N1|||2.3.4.4b|20.02.2023|Benedetti_Estefania|
||28.12.2023|Direccion_del_Laboratorio_Animal_Direccion_General_de_Laborato
rios_y_Control_Tecnico_SENASA.|Instituto_Nacional_de_Enfermedades_Infeccios
"/>
NNN
</sequence>
<sequence>
<taxon idref="A/Chicken/Argentina/506-
2/2023|EPI_ISL_18698474|A_/_H5N1|||2.3.4.4b|20.02.2023|Benedetti_Estefania|
||28.12.2023|Direccion_del_Laboratorio_Animal_Direccion_General_de_Laborato
rios_y_Control_Tecnico_SENASA.|Instituto_Nacional_de_Enfermedades_Infeccios
"/>
NNN
</sequence>
<sequence>
<taxon idref="A/Chicken/Argentina/509-
2/2023|EPI_ISL_18698476|A_/_H5N1|||2.3.4.4b|21.02.2023|Benedetti_Estefania|
||28.12.2023|Direccion_del_Laboratorio_Animal_Direccion_General_de_Laborato
rios_y_Control_Tecnico_SENASA.|Instituto_Nacional_de_Enfermedades_Infeccios
"/>
NNN
</sequence>
<sequence>
<taxon idref="A/Chicken/Argentina/556-
6/2023|EPI_ISL_18698478|A_/_H5N1|||2.3.4.4b|23.02.2023|Benedetti_Estefania|
||28.12.2023|Direccion_del_Laboratorio_Animal_Direccion_General_de_Laborato
rios_y_Control_Tecnico_SENASA.|Instituto_Nacional_de_Enfermedades_Infeccios
"/>
NNN
</sequence>
<sequence>
<taxon idref="A/Chicken/Argentina/559-
8/2023|EPI_ISL_18698480|A_/_H5N1|||2.3.4.4b|22.02.2023|Benedetti_Estefania|
||28.12.2023|Direccion_del_Laboratorio_Animal_Direccion_General_de_Laborato
rios_y_Control_Tecnico_SENASA.|Instituto_Nacional_de_Enfermedades_Infeccios
"/>
NNN
</sequence>
<sequence>
<taxon idref="A/Chicken/Argentina/578-
2/2023|EPI_ISL_18698482|A_/_H5N1|||2.3.4.4b|23.02.2023|Benedetti_Estefania|
||28.12.2023|Direccion_del_Laboratorio_Animal_Direccion_General_de_Laborato
rios_y_Control_Tecnico_SENASA.|Instituto_Nacional_de_Enfermedades_Infeccios
"/>
NNN
</sequence>
<sequence>
<taxon idref="A/Chicken/Argentina/588-
4/2023|EPI_ISL_18698490|A_/_H5N1|||2.3.4.4b|24.02.2023|Benedetti_Estefania|
||28.12.2023|Direccion_del_Laboratorio_Animal_Direccion_General_de_Laborato
rios_y_Control_Tecnico_SENASA.|Instituto_Nacional_de_Enfermedades_Infeccios
"/>
NNN
</sequence>
<sequence>
<taxon idref="A/Chicken/Argentina/606-
1/2023|EPI_ISL_18698492|A_/_H5N1|||2.3.4.4b|27.02.2023|Benedetti_Estefania|
||28.12.2023|Direccion_del_Laboratorio_Animal_Direccion_General_de_Laborato

rios_y_Control_Tecnico_SENASA.|Instituto_Nacional_de_Enfermedades_Infeccios
"/>
NNN
</sequence>
<sequence>
<taxon idref="A/Chicken/Argentina/736-
1/2023|EPI_ISL_18698494|A_/_H5N1|||2.3.4.4b|03.03.2023|Benedetti_Estefania|
||28.12.2023|Direccion_del_Laboratorio_Animal_Direccion_General_de_Laborato
rios_y_Control_Tecnico_SENASA.|Instituto_Nacional_de_Enfermedades_Infeccios
"/>
NNN
</sequence>
<sequence>
<taxon idref="A/Chicken/Argentina/747-
1/2023|EPI_ISL_18698730|A_/_H5N1|||2.3.4.4b|04.03.2023|Benedetti_Estefania|
||28.12.2023|Direccion_del_Laboratorio_Animal_Direccion_General_de_Laborato
rios_y_Control_Tecnico_SENASA.|Instituto_Nacional_de_Enfermedades_Infeccios
"/>
NNN
</sequence>
<sequence>
<taxon idref="A/Chicken/Argentina/858-
1/2023|EPI_ISL_18698499|A_/_H5N1|||2.3.4.4b|08.03.2023|Benedetti_Estefania|
||28.12.2023|Direccion_del_Laboratorio_Animal_Direccion_General_de_Laborato
rios_y_Control_Tecnico_SENASA.|Instituto_Nacional_de_Enfermedades_Infeccios
"/>
NNN
</sequence>
<sequence>
<taxon idref="A/Chicken/Argentina/895-
1/2023|EPI_ISL_18698731|A_/_H5N1|||2.3.4.4b|09.03.2023|Benedetti_Estefania|
||28.12.2023|Direccion_del_Laboratorio_Animal_Direccion_General_de_Laborato
rios_y_Control_Tecnico_SENASA.|Instituto_Nacional_de_Enfermedades_Infeccios
"/>
NNN
</sequence>
<sequence>
<taxon idref="A/Chicken/Argentina/919-
3/2023|EPI_ISL_18698502|A_/_H5N1|||2.3.4.4b|13.03.2023|Benedetti_Estefania|
||28.12.2023|Direccion_del_Laboratorio_Animal_Direccion_General_de_Laborato
rios_y_Control_Tecnico_SENASA.|Instituto_Nacional_de_Enfermedades_Infeccios
"/>
NNN
</sequence>
<sequence>
<taxon idref="A/Chicken/BC/FAV-0005-
1/2023|EPI_ISL_19155141|A_/_H5N1|||2.3.4.4b|03.01.2023|Signore_Anthony|||21
.05.2024|Canadian_Food_Inspection_Agency|Canadian_Food_Inspection_Agency|HA
|4|A/Chicken/BC/FAV-0005-1/2023_HA|EPI3315808|DNA_IN"/>
NNN
</sequence>
<sequence>
<taxon idref="A/Chicken/BC/FAV-0005-
2/2023|EPI_ISL_19155142|A_/_H5N1|||2.3.4.4b|03.01.2023|Signore_Anthony|||21
.05.2024|Canadian_Food_Inspection_Agency|Canadian_Food_Inspection_Agency|HA
|4|A/Chicken/BC/FAV-0005-2/2023_HA|EPI3315816|DNA_IN"/>
NNN
</sequence>
<sequence>
<taxon idref="A/Chicken/BC/FAV-0010-
1/2023|EPI_ISL_19155147|A_/_H5N1|||2.3.4.4b|09.01.2023|Signore_Anthony|||21

.05.2024|Canadian_Food_Inspection_Agency|Canadian_Food_Inspection_Agency|HA|4|A/Chicken/BC/FAV-0010-1/2023_HA|EPI3315856|DNA_IN"/>
NNN
</sequence>
<sequence>
<taxon idref="A/Chicken/BC/FAV-0010-2/2023|EPI_ISL_19155148|A_/_H5N1|||2.3.4.4b|09.01.2023|Signore_Anthony|||21.05.2024|Canadian_Food_Inspection_Agency|Canadian_Food_Inspection_Agency|HA|4|A/Chicken/BC/FAV-0010-2/2023_HA|EPI3315864|DNA_IN"/>
NNN
</sequence>
<sequence>
<taxon idref="A/Chicken/BC/FAV-0011-1/2023|EPI_ISL_19155149|A_/_H5N1|||2.3.4.4b|09.01.2023|Signore_Anthony|||21.05.2024|Canadian_Food_Inspection_Agency|Canadian_Food_Inspection_Agency|HA|4|A/Chicken/BC/FAV-0011-1/2023_HA|EPI3315872|DNA_IN"/>
NNN
</sequence>
<sequence>
<taxon idref="A/Chicken/BC/FAV-0011-2/2023|EPI_ISL_19155150|A_/_H5N1|||2.3.4.4b|09.01.2023|Signore_Anthony|||21.05.2024|Canadian_Food_Inspection_Agency|Canadian_Food_Inspection_Agency|HA|4|A/Chicken/BC/FAV-0011-2/2023_HA|EPI3315880|DNA_IN"/>
NNN
</sequence>
<sequence>
<taxon idref="A/Chicken/BC/FAV-1617-1/2022|EPI_ISL_19155051|A_/_H5N1|||2.3.4.4b|05.12.2022|Signore_Anthony|||21.05.2024|Canadian_Food_Inspection_Agency|Canadian_Food_Inspection_Agency|HA|4|A/Chicken/BC/FAV-1617-1/2022_HA|EPI3315088|DNA_IN"/>
NNN
</sequence>
<sequence>
<taxon idref="A/Chicken/BC/FAV-1617-2/2022|EPI_ISL_19155052|A_/_H5N1|||2.3.4.4b|05.12.2022|Signore_Anthony|||21.05.2024|Canadian_Food_Inspection_Agency|Canadian_Food_Inspection_Agency|HA|4|A/Chicken/BC/FAV-1617-2/2022_HA|EPI3315096|DNA_IN"/>
NNN
</sequence>
<sequence>
<taxon idref="A/Chicken/BC/FAV-1630-1/2022|EPI_ISL_19155066|A_/_H5N1|||2.3.4.4b|07.12.2022|Signore_Anthony|||21.05.2024|Canadian_Food_Inspection_Agency|Canadian_Food_Inspection_Agency|HA|4|A/Chicken/BC/FAV-1630-1/2022_HA|EPI3315208|DNA_IN"/>
NNN
</sequence>
<sequence>
<taxon idref="A/Chicken/BC/FAV-1630-2/2022|EPI_ISL_19155067|A_/_H5N1|||2.3.4.4b|07.12.2022|Signore_Anthony|||21.05.2024|Canadian_Food_Inspection_Agency|Canadian_Food_Inspection_Agency|HA|4|A/Chicken/BC/FAV-1630-2/2022_HA|EPI3315216|DNA_IN"/>
NNN
</sequence>
<sequence>
<taxon idref="A/Chicken/BC/FAV-1647-1/2022|EPI_ISL_19155091|A_/_H5N1|||2.3.4.4b|12.12.2022|Signore_Anthony|||21.05.2024|Canadian_Food_Inspection_Agency|Canadian_Food_Inspection_Agency|HA|4|A/Chicken/BC/FAV-1647-1/2022_HA|EPI3315408|DNA_IN"/>
NNN
</sequence>
<sequence>

<taxon idref="A/Chicken/BC/FAV-1647-
2/2022|EPI_ISL_19155092|A_/_H5N1|||2.3.4.4b|12.12.2022|Signore_Anthony|||21
.05.2024|Canadian_Food_Inspection_Agency|Canadian_Food_Inspection_Agency|HA
|4|A/Chicken/BC/FAV-1647-2/2022_HA|EPI3315416|DNA_IN"/>
NNN
</sequence>
<sequence>
<taxon idref="A/Chicken/BC/FAV-1685-
1/2022|EPI_ISL_19155130|A_/_H5N1|||2.3.4.4b|28.12.2022|Signore_Anthony|||21
.05.2024|Canadian_Food_Inspection_Agency|Canadian_Food_Inspection_Agency|HA
|4|A/Chicken/BC/FAV-1685-1/2022_HA|EPI3315720|DNA_IN"/>
NNN
</sequence>
<sequence>
<taxon idref="A/Chicken/BC/FAV-1685-
2/2022|EPI_ISL_19155131|A_/_H5N1|||2.3.4.4b|28.12.2022|Signore_Anthony|||21
.05.2024|Canadian_Food_Inspection_Agency|Canadian_Food_Inspection_Agency|HA
|4|A/Chicken/BC/FAV-1685-2/2022_HA|EPI3315728|DNA_INS"/>
NNN
</sequence>
<sequence>
<taxon idref="A/Chicken/BC/FAV-1689-
1/2022|EPI_ISL_19155136|A_/_H5N1|||2.3.4.4b|28.12.2022|Signore_Anthony|||21
.05.2024|Canadian_Food_Inspection_Agency|Canadian_Food_Inspection_Agency|HA
|4|A/Chicken/BC/FAV-1689-1/2022_HA|EPI3315768|DNA_IN"/>
NNN
</sequence>
<sequence>
<taxon idref="A/Chicken/BC/FAV-1689-
2/2022|EPI_ISL_19155137|A_/_H5N1|||2.3.4.4b|28.12.2022|Signore_Anthony|||21
.05.2024|Canadian_Food_Inspection_Agency|Canadian_Food_Inspection_Agency|HA
|4|A/Chicken/BC/FAV-1689-2/2022_HA|EPI3315776|DNA_IN"/>
NNN
</sequence>
<sequence>
<taxon idref="A/Chicken/NB/FAV-
0013/2023|EPI_ISL_19155156|A_/_H5N1|||2.3.4.4b|10.01.2023|Signore_Anthony||
|21.05.2024|Canadian_Food_Inspection_Agency|Canadian_Food_Inspection_Agency
|HA|4|A/Chicken/NB/FAV-0013/2023_HA|EPI3315927|DNA_IN"/>
NNN
</sequence>
<sequence>
<taxon idref="A/Chile/25945/2023|EPI_ISL_17468386|A_/_H5N1|||2.3.4.4b|24.03
.2023|Campano_Constanza|||13.04.2023|Instituto_de_Salud_Publica_de_Chile|In
stituto_de_Salud_Publica_de_Chile|HA|4|A/Chile/25945/2023|EPI2510183|DNA_IN
"/>
NNN
</sequence>
<sequence>
<taxon idref="A/Chiloe_wigeon/OHiggins/240893-
2/2023|EPI_ISL_17885965|A_/_H5N1|Original||2.3.4.4b|09.03.2023|GISAID_EpiFl
u_Data_Curator|||19.07.2023|CEIRS_Data_Processing_and_Coordinating_Center_C
enter_for_Research_on_Influenza_Pathogenesis__CRIP_|CEIRS_Data_Proce"/>
NNN
</sequence>
<sequence>
<taxon idref="A/Cooper_s_Hawk/BC/AIVPHL-
1125/2023|EPI_ISL_18665595|A_/_H5N1|Original||2.3.4.4b|23.05.2023|Caleta_Je
ssica_Marija|||21.12.2023|B.C._Centre_for_Disease_Control|Public_Health_Age
ncy_of_Canada|HA|4|A/Cooper_s_Hawk/BC/AIVPHL-
1125/2023_HA|EPI2856671|DNA_IN"/>

NNN
</sequence>
<sequence>
<taxon idref="A/Cooper_s_Hawk/BC/AIVPHL-924/2023|EPI_ISL_18665500|A_/_H5N1|Original||2.3.4.4b|30.01.2023|Caleta_Jessica_Marija|||21.12.2023|B.C._Centre_for_Disease_Control|Public_Health_Agency_of_Canada|HA|4|A/Cooper_s_Hawk/BC/AIVPHL-924/2023_HA|EPI2855965|DNA_INS"/>
NNN
</sequence>
<sequence>
<taxon idref="A/Cooper_s_hawk/Montana/23-016105-001/2023|EPI_ISL_17964974|A_/_H5N1|Original||2.3.4.4b|16.04.2023|Killian_Mary_Lea||23-016105-001|06.07.2023|National_Veterinary_Services_Laboratories_-_USDA|National_Veterinary_Services_Laboratories_-_USDA|HA|4|HA_A/C"/>
NNN
</sequence>
<sequence>
<taxon idref="A/Cooper_s_s_hawk/Colorado/23-005617-001/2023|EPI_ISL_17964858|A_/_H5N1|Original||2.3.4.4b|14.02.2023|Killian_Mary_Lea||23-005617-001|06.07.2023|National_Veterinary_Services_Laboratories_-_USDA|National_Veterinary_Services_Laboratories_-_USDA|HA|4|HA_"/>
NNN
</sequence>
<sequence>
<taxon idref="A/Duck/Argentina/1712-5/2023|EPI_ISL_18698515|A_/_H5N1|||2.3.4.4b|19.04.2023|Benedetti_Estefania|||28.12.2023|Direccion_del_Laboratorio_Animal_Direccion_General_de_Laboratorios_y_Control_Tecnico_SENASA.|Instituto_Nacional_de_Enfermedades_Infecciosas"/>
NNN
</sequence>
<sequence>
<taxon idref="A/Duck/Argentina/2197-1/2023|EPI_ISL_18698523|A_/_H5N1|||2.3.4.4b|11.05.2023|Benedetti_Estefania|||28.12.2023|Direccion_del_Laboratorio_Animal_Direccion_General_de_Laboratorios_y_Control_Tecnico_SENASA.|Instituto_Nacional_de_Enfermedades_Infecciosas"/>
NNN
</sequence>
<sequence>
<taxon idref="A/Duck/BC/FAV-1593-3/2022|EPI_ISL_19155028|A_/_H5N1|||2.3.4.4b|01.12.2022|Signore_Anthony|||21.05.2024|Canadian_Food_Inspection_Agency|Canadian_Food_Inspection_Agency|HA|4|A/Duck/BC/FAV-1593-3/2022_HA|EPI3314904|DNA_IN"/>
NNN
</sequence>
<sequence>
<taxon idref="A/Duck/BC/FAV-1593-4/2022|EPI_ISL_19155029|A_/_H5N1|||2.3.4.4b|01.12.2022|Signore_Anthony|||21.05.2024|Canadian_Food_Inspection_Agency|Canadian_Food_Inspection_Agency|HA|4|A/Duck/BC/FAV-1593-4/2022_HA|EPI3314912|DNA_IN"/>
NNN
</sequence>
<sequence>
<taxon idref="A/Dunlin/BC/AIVPHL-940/2023|EPI_ISL_18665510|A_/_H5N1|Original||2.3.4.4b|06.02.2023|Caleta_Jessica_Marija|||21.12.2023|B.C._Centre_for_Disease_Control|Public_Health_Agency_of_Canada|HA|4|A/Dunlin/BC/AIVPHL-940/2023_HA|EPI2856045|DNA_INS"/>

NNN
</sequence>
<sequence>
<taxon idref="A/Dunlin/BC/FAV-0053-
16/2022|EPI_ISL_19155099|A_/_H5N1|||2.3.4.4b|13.12.2022|Signore_Anthony|||2
1.05.2024|Canadian_Food_Inspection_Agency|Canadian_Food_Inspection_Agency|H
A|4|A/Dunlin/BC/FAV-0053-16/2022_HA|EPI3315472|DNA_IN"/>
NNN
</sequence>
<sequence>
<taxon idref="A/Elegant_tern/Arica_y_Parinacota/229476-
1/2022|EPI_ISL_17885955|A_/_H5N1|Original||2.3.4.4b|24.12.2022|GISAID_EpiFl
u_Data_Curator|||19.07.2023|CEIRS_Data_Processing_and_Coordinating_Center_C
enter_for_Research_on_Influenza_Pathogenesis__CRIP_|CEIRS_D"/>
NNN
</sequence>
<sequence>
<taxon idref="A/Elegant_tern/Tarapaca/229133-
1/2022|EPI_ISL_17885953|A_/_H5N1|Original||2.3.4.4b|23.12.2022|GISAID_EpiFl
u_Data_Curator|||19.07.2023|CEIRS_Data_Processing_and_Coordinating_Center_C
enter_for_Research_on_Influenza_Pathogenesis__CRIP_|CEIRS_Data_Proces"/>
NNN
</sequence>
<sequence>
<taxon idref="A/Franklin_gull/OHiggins/236195-
1/2023|EPI_ISL_19162729|A_/_H5N1|Original||2.3.4.4b|12.02.2023||||24.05.202
4|Emory_University__CEIRR_|Emory_University__CEIRR_|HA|4|A/Franklin_gull/OH
iggins/236195-1/2023_HA|EPI3333780|DNA_IN"/>
NNN
</sequence>
<sequence>
<taxon idref="A/Gallus_gallus/Mato_Grosso_do_Sul/2108-
SN52/2023|EPI_ISL_19215176|A_/_H5N1|Original||2.3.4.4b|22.09.2023||||25.06.
2024|Ministerio_da_Agricultura_e_Pecuaria||HA|4|A/Gallus_gallus/Bonito/2108
-SN52/2023_HA|EPI3395036|DNA_IN"/>
NNN
</sequence>
<sequence>
<taxon idref="A/Glaucous-winged_gull/Washington/W231990001-
1/2023|EPI_ISL_19592595|A_/_H5N1|Original||2.3.4.4b|13.07.2023||||06.12.202
4|Washington_State_University||HA|4|A/Glaucous-
winged_gull/Washington/W231990001-1/2023_HA|EPI3675325|DNA_IN"/>
NNN
</sequence>
<sequence>
<taxon idref="A/Glaucous-winged_gull/Washington/W232270041-
2/2023|EPI_ISL_19592596|A_/_H5N1|Original||2.3.4.4b|11.08.2023||||06.12.202
4|Washington_State_University||HA|4|A/Glaucous-
winged_gull/Washington/W232270041-2/2023_HA|EPI3675333|DNA_IN"/>
NNN
</sequence>
<sequence>
<taxon idref="A/Glaucous-winged_gull/Washington/W232270041-
4/2023|EPI_ISL_19592597|A_/_H5N1|Original||2.3.4.4b|11.08.2023||||06.12.202
4|Washington_State_University||HA|4|A/Glaucous-
winged_gull/Washington/W232270041-4/2023_HA|EPI3675341|DNA_IN"/>
NNN
</sequence>
<sequence>
<taxon idref="A/Goose/Argentina/389-
1/2023|EPI_ISL_18698459|A_/_H5N1|||2.3.4.4b|11.02.2023|Benedetti_Estefania|

||28.12.2023|Direccion_del_Laboratorio_Animal_Direccion_General_de_Laboratorios_y_Control_Tecnico_SENASA.|Instituto_Nacional_de_Enfermedades_Infecciosas"/>
NNN
</sequence>
<sequence>
<taxon idref="A/Gray_gull/Tarapaca/232825-1/2023|EPI_ISL_17885951|A_/_H5N1|Original||2.3.4.4b|18.01.2023|GISAID_EpiFlu_Data_Curator|||19.07.2023|CEIRS_Data_Processing_and_Coordinating_Center_Center_for_Research_on_Influenza_Pathogenesis__CRIP_|CEIRS_Data_Processin"/>
NNN
</sequence>
<sequence>
<taxon idref="A/Great_Blue_Heron/BC/AIVPHL-835/2022|EPI_ISL_18665537|A_/_H5N1|Original||2.3.4.4b|14.12.2022|Caleta_Jessica_Marija|||21.12.2023|B.C._Centre_for_Disease_Control|Public_Health_Agency_of_Canada|HA|4|A/Great_Blue_Heron/BC/AIVPHL-835/2022_HA|EPI2856234|DN"/>
NNN
</sequence>
<sequence>
<taxon idref="A/Great_Horned_Owl/ON/FAV-0002-2/2022|EPI_ISL_19155078|A_/_H5N1|||2.3.4.4b|10.12.2022|Signore_Anthony|||21.05.2024|Canadian_Food_Inspection_Agency|Canadian_Food_Inspection_Agency|HA|4|A/Great_Horned_Owl/ON/FAV-0002-2/2022_HA|EPI3315304|DNA_IN"/>
NNN
</sequence>
<sequence>
<taxon idref="A/Great_egret/Araucania/240518/2023|EPI_ISL_17885950|A_/_H5N1|Original||2.3.4.4b|07.03.2023|GISAID_EpiFlu_Data_Curator|||24.07.2023|CEIRS_Data_Processing_and_Coordinating_Center_Center_for_Research_on_Influenza_Pathogenesis__CRIP_|CEIRS_Data_Processi"/>
NNN
</sequence>
<sequence>
<taxon idref="A/Great_grabe/Atacama/231482-1/2023|EPI_ISL_19162730|A_/_H5N1|Original||2.3.4.4b|09.01.2023||||24.05.2024|Emory_University__CEIRR_|Emory_University__CEIRR_|HA|4|A/Great_grabe/Atacama/231482-1/2023_HA|EPI3333788|DNA_IN"/>
NNN
</sequence>
<sequence>
<taxon idref="A/Great_grabe/Atacama/231482-2/2023|EPI_ISL_19162731|A_/_H5N1|Original||2.3.4.4b|09.01.2023||||24.05.2024|Emory_University__CEIRR_|Emory_University__CEIRR_|HA|4|A/Great_grabe/Atacama/231482-2/2023_HA|EPI3333795|DNA_IN"/>
NNN
</sequence>
<sequence>
<taxon idref="A/Guanay_cormorant/Chile/239584/2023|EPI_ISL_19391460|A_/_H5N1|Original||2.3.4.4b|01.03.2023||||09.09.2024|CEIRS_Data_Processing_and_Coordinating_Center_Center_for_Research_on_Influenza_Pathogenesis__CRIP_|CEIRS_Data_Processing_and_Coordinating_Center"/>
NNN
</sequence>
<sequence>
<taxon idref="A/Gull/Chile/227023-2/2022|EPI_ISL_17011964|A_/_H5N1|||2.3.4.4b|07.12.2022||||22.02.2023|CEIRS_Data_Processing_and_Coordinating_Center_Center_for_Research_on_Influenza_Pathogenesis__CRIP_|CEIRS_Data_Processing_and_Coordinating_Center_Center_for_Resea"/>
NNN

</sequence>
<sequence>
<taxon idref="A/Gull/Chile/227023-3/2022|EPI_ISL_17011958|A_/_H5N1|||2.3.4.4b|07.12.2022||||22.02.2023|CEIRS_Data_Processing_and_Coordinating_Center_Center_for_Research_on_Influenza_Pathogenesis__CRIP_|CEIRS_Data_Processing_and_Coordinating_Center_Center_for_Resea"/>
NNN
</sequence>
<sequence>
<taxon idref="A/Harbor_seal/Washington/W232490069-3/2023|EPI_ISL_19592610|A_/_H5N1|Original||2.3.4.4b|13.08.2023||||06.12.2024|Washington_State_University||HA|4|A/Harbor_seal/Washington/W232490069-3/2023_HA|EPI3675357|DNA_IN"/>
NNN
</sequence>
<sequence>
<taxon idref="A/Humboldt_penguin/Antofagasta/236063-2/2023|EPI_ISL_18760066|A_/_H5N1|Original||2.3.4.4b|05.02.2023||||14.01.2024|Emory_University__CEIRR_|Emory_University__CEIRR_|HA|4|A/Humboldt_penguin/Antofagasta/236063-2/2023_HA|EPI2913222|DNA_IN"/>
NNN
</sequence>
<sequence>
<taxon idref="A/Humboldt_penguin/Coquimbo/239590/2023|EPI_ISL_17885948|A_/_H5N1|Original||2.3.4.4b|28.02.2023|GISAID_EpiFlu_Data_Curator|||19.07.2023|CEIRS_Data_Processing_and_Coordinating_Center_Center_for_Research_on_Influenza_Pathogenesis__CRIP_|CEIRS_Data_Proc"/>
NNN
</sequence>
<sequence>
<taxon idref="A/Inca_tern/Antofagasta/238083/2023|EPI_ISL_19131156|A_/_H5N1|Original||2.3.4.4b|22.02.2023||||10.05.2024|CEIRS_Data_Processing_and_Coordinating_Center_Center_for_Research_on_Influenza_Pathogenesis__CRIP_||HA|4|A/Inca_tern/Antofagasta/238083/2023_HA|E"/>
NNN
</sequence>
<sequence>
<taxon idref="A/Inca_tern/Arica_y_Parinacota/227519-1/2022|EPI_ISL_19131159|A_/_H5N1|Original||2.3.4.4b|12.12.2022||||10.05.2024|CEIRS_Data_Processing_and_Coordinating_Center_Center_for_Research_on_Influenza_Pathogenesis__CRIP_||HA|4|A/Inca_tern/Arica_y_Parinacota/"/>
NNN
</sequence>
<sequence>
<taxon idref="A/Inca_tern/Coquimbo/239619/2023|EPI_ISL_19131162|A_/_H5N1|Original||2.3.4.4b|01.03.2023||||10.05.2024|CEIRS_Data_Processing_and_Coordinating_Center_Center_for_Research_on_Influenza_Pathogenesis__CRIP_||HA|4|A/Inca_tern/Coquimbo/239619/2023_HA|EPI3276"/>
NNN
</sequence>
<sequence>
<taxon idref="A/Kelp_Gull/Hound_Bay/133744/2023|EPI_ISL_18592422|A_/_H5N1|Clinical_Sample||2.3.4.4b|30.10.2023|Mollett_Benjamin|||06.12.2023|Animal_and_Plant_Health_Agency__APHA_|Animal_and_Plant_Health_Agency__APHA_|HA|4|A/Kelp_Gull/Hound_Bay/133744/2023|HA|EPI281"/>
NNN
</sequence>
<sequence>
<taxon idref="A/Kelp_Gull/Hound_Bay/133747/2023|EPI_ISL_18592423|A_/_H5N1|Clinical_Sample||2.3.4.4b|30.10.2023|Mollett_Benjamin|||06.12.2023|Animal_an

d_Plant_Health_Agency__APHA_|Animal_and_Plant_Health_Agency__APHA_|HA|4|A/K
elp_Gull/Hound_Bay/133747/2023|HA|EPI281"/>
NNN
</sequence>
<sequence>
<taxon idref="A/Kelp_Gull/Moltke_Harbour/133754/2023|EPI_ISL_18592425|A_/_H
5N1|Clinical_Sample||2.3.4.4b|31.10.2023|Mollett_Benjamin|||06.12.2023|Anim
al_and_Plant_Health_Agency__APHA_|Animal_and_Plant_Health_Agency__APHA_|HA|
4|A/Kelp_Gull/Moltke_Harbour/133754/2023"/>
NNN
</sequence>
<sequence>
<taxon idref="A/Kelp_Gull/Penguin_River/141234/2023|EPI_ISL_18742215|A_/_H5
N1|Clinical_Sample||2.3.4.4b|08.11.2023|Mollett_Benjamin|||25.06.2024|Anima
l_and_Plant_Health_Agency__APHA_|Animal_and_Plant_Health_Agency__APHA_|HA|4
|A/Kelp_Gull/Penguin_River/141234/2023|H"/>
NNN
</sequence>
<sequence>
<taxon idref="A/Kelp_Gull/Penguin_River/141239/2023|EPI_ISL_18742217|A_/_H5
N1|Clinical_Sample||2.3.4.4b|08.11.2023|Mollett_Benjamin|||25.06.2024|Anima
l_and_Plant_Health_Agency__APHA_|Animal_and_Plant_Health_Agency__APHA_|HA|4
|A/Kelp_Gull/Penguin_River/141239/2023|H"/>
NNN
</sequence>
<sequence>
<taxon idref="A/Kelp_Gull/South_Georgia_and_the_South_Sandwich_Islands/136/
2023|EPI_ISL_19313644|A_/_H5N1|Original||2.3.4.4b|08.11.2023||||06.08.2024|
Animal_and_Plant_Health_Agency__APHA_||HA|4|A/Kelp_Gull/South_Georgia_and_t
he_South_Sandwich_Islands/136/2023_HA|EPI"/>
NNN
</sequence>
<sequence>
<taxon idref="A/Kelp_Gull/South_Georgia_and_the_South_Sandwich_Islands/152/
2023|EPI_ISL_19313573|A_/_H5N1|Original||2.3.4.4b|30.10.2023||||06.08.2024|
Animal_and_Plant_Health_Agency__APHA_||HA|4|A/Kelp_Gull/South_Georgia_and_t
he_South_Sandwich_Islands/152/2023_HA|EPI"/>
NNN
</sequence>
<sequence>
<taxon idref="A/Kelp_Gull/South_Georgia_and_the_South_Sandwich_Islands/160/
2023|EPI_ISL_19313506|A_/_H5N1|Original||2.3.4.4b|30.10.2023||||06.08.2024|
Animal_and_Plant_Health_Agency__APHA_||HA|4|A/Kelp_Gull/South_Georgia_and_t
he_South_Sandwich_Islands/160/2023_HA|EPI"/>
NNN
</sequence>
<sequence>
<taxon idref="A/Kelp_Gull/South_Georgia_and_the_South_Sandwich_Islands/32/2
023|EPI_ISL_19313514|A_/_H5N1|Original||2.3.4.4b|03.11.2023||||06.08.2024|A
nimal_and_Plant_Health_Agency__APHA_||HA|4|A/Kelp_Gull/South_Georgia_and_th
e_South_Sandwich_Islands/32/2023_HA|EPI34"/>
NNN
</sequence>
<sequence>
<taxon idref="A/Kelp_Gull/South_Georgia_and_the_South_Sandwich_Islands/56/2
023|EPI_ISL_19313522|A_/_H5N1|Original||2.3.4.4b|31.10.2023||||06.08.2024|A
nimal_and_Plant_Health_Agency__APHA_||HA|4|A/Kelp_Gull/South_Georgia_and_th
e_South_Sandwich_Islands/56/2023_HA|EPI34"/>
NNN
</sequence>
<sequence>

<taxon idref="A/Kelp_Gull/South_Georgia_and_the_South_Sandwich_Islands/72/2
023|EPI_ISL_19313477|A_/_H5N1|Original||2.3.4.4b|08.11.2023||||06.08.2024|A
nimal_and_Plant_Health_Agency__APHA_||HA|4|A/Kelp_Gull/South_Georgia_and_th
e_South_Sandwich_Islands/72/2023_HA|EPI34"/>
NNN
</sequence>
<sequence>
<taxon idref="A/Kelp_gull/Maule/239349/2023|EPI_ISL_17885972|A_/_H5N1|Origi
nal||2.3.4.4b|01.03.2023|GISAID_EpiFlu_Data_Curator|||19.07.2023|CEIRS_Data
_Processing_and_Coordinating_Center_Center_for_Research_on_Influenza_Pathog
enesis__CRIP_|CEIRS_Data_Processing_and"/>
NNN
</sequence>
<sequence>
<taxon idref="A/Layer_Chicken/BC/FAV-1623-
1/2022|EPI_ISL_19155058|A_/_H5N1|||2.3.4.4b|06.12.2022|Signore_Anthony|||21
.05.2024|Canadian_Food_Inspection_Agency|Canadian_Food_Inspection_Agency|HA
|4|A/Layer_Chicken/BC/FAV-1623-1/2022_HA|EPI3315144|DNA_IN"/>
NNN
</sequence>
<sequence>
<taxon idref="A/Layer_Chicken/BC/FAV-1623-
2/2022|EPI_ISL_19155059|A_/_H5N1|||2.3.4.4b|06.12.2022|Signore_Anthony|||21
.05.2024|Canadian_Food_Inspection_Agency|Canadian_Food_Inspection_Agency|HA
|4|A/Layer_Chicken/BC/FAV-1623-2/2022_HA|EPI3315152|DNA_IN"/>
NNN
</sequence>
<sequence>
<taxon idref="A/Long_Eared_Owl/BC/AIVPHL-
925/2023|EPI_ISL_18665502|A_/_H5N1|Original||2.3.4.4b|30.01.2023|Caleta_Jes
sica_Marija|||21.12.2023|B.C._Centre_for_Disease_Control|Public_Health_Agen
cy_of_Canada|HA|4|A/Long_Eared_Owl/BC/AIVPHL-
925/2023_HA|EPI2855981|DNA_IN"/>
NNN
</sequence>
<sequence>
<taxon idref="A/Mixed/AB/FAV-
1654/2022|EPI_ISL_19155100|A_/_H5N1|||2.3.4.4b|14.12.2022|Signore_Anthony||
|21.05.2024|Canadian_Food_Inspection_Agency|Canadian_Food_Inspection_Agency
|HA|4|A/Mixed/AB/FAV-1654/2022_HA|EPI3315480|DNA_IN"/>
NNN
</sequence>
<sequence>
<taxon idref="A/Northern_Pintail/BC/FAV-0053-
43/2023|EPI_ISL_19155143|A_/_H5N1|||2.3.4.4b|06.01.2023|Signore_Anthony|||2
1.05.2024|B.C._Centre_for_Disease_Control|Canadian_Food_Inspection_Agency|H
A|4|A/Northern_Pintail/BC/FAV-0053-43/2023_HA|EPI3315824|DNA_IN"/>
NNN
</sequence>
<sequence>
<taxon idref="A/Numida_meleagris/Santa_Catarina/1843-
N3/2023|EPI_ISL_19215178|A_/_H5N1|Original||2.3.4.4b|15.09.2023||||25.11.20
24|Ministerio_da_Agricultura_e_Pecuaria||HA|4|A/Numida_meleagris/Maracaja/1
843-N3/2023_HA|EPI3395052|DNA_IN"/>
NNN
</sequence>
<sequence>
<taxon idref="A/Otaria_flavescens/Rio_Grande_do_Sul/2165-
SO/2023|EPI_ISL_19215180|A_/_H5N1|Original||2.3.4.4b|11.10.2023||||25.06.20
24|Ministerio_da_Agricultura_e_Pecuaria||HA|4|A/Otaria_flavescens/Torres/21
65-SO/2023_HA|EPI3395068|DNA_IN"/>

NNN
</sequence>
<sequence>
<taxon idref="A/Pelecanus/Peru/VFAR-140/2022|EPI_ISL_17099964|A_/_H5N1|SPF_Eggs||2.3.4.4b|01.12.2022|Tataje_Luis|VFAR-140|VFAR-140|06.03.2023|Farmacologicos_Veterinarios_S.A.C.__FARVET_SAC_|Universidad_Privada_San_Juan_Bautista|HA|4|A/Pelecanus/Peru/VFAR-140/2022|E"/>
NNN
</sequence>
<sequence>
<taxon idref="A/Pelican/Antofagasta/228244-2/2022|EPI_ISL_17885944|A_/_H5N1|Original||2.3.4.4b|16.12.2022|GISAID_EpiFlu_Data_Curator|||29.06.2023|CEIRS_Data_Processing_and_Coordinating_Center_Center_for_Research_on_Influenza_Pathogenesis__CRIP_|CEIRS_Data_Processi"/>
NNN
</sequence>
<sequence>
<taxon idref="A/Pelican/Antofagasta/228246-2/2022|EPI_ISL_17885934|A_/_H5N1|Original||2.3.4.4b|15.12.2022|GISAID_EpiFlu_Data_Curator|||19.07.2023|CEIRS_Data_Processing_and_Coordinating_Center_Center_for_Research_on_Influenza_Pathogenesis__CRIP_|CEIRS_Data_Processi"/>
NNN
</sequence>
<sequence>
<taxon idref="A/Pelican/Antofagasta/228246-3/2022|EPI_ISL_17885931|A_/_H5N1|Original||2.3.4.4b|15.12.2022|GISAID_EpiFlu_Data_Curator|||19.07.2023|CEIRS_Data_Processing_and_Coordinating_Center_Center_for_Research_on_Influenza_Pathogenesis__CRIP_|CEIRS_Data_Processi"/>
NNN
</sequence>
<sequence>
<taxon idref="A/Pelican/Antofagasta/228272-1/2022|EPI_ISL_17885929|A_/_H5N1|Original||2.3.4.4b|18.12.2022|GISAID_EpiFlu_Data_Curator|||19.07.2023|CEIRS_Data_Processing_and_Coordinating_Center_Center_for_Research_on_Influenza_Pathogenesis__CRIP_|CEIRS_Data_Processi"/>
NNN
</sequence>
<sequence>
<taxon idref="A/Pelican/Atacama/229424-2/2022|EPI_ISL_17885927|A_/_H5N1|Original||2.3.4.4b|22.12.2022|GISAID_EpiFlu_Data_Curator|||19.07.2023|CEIRS_Data_Processing_and_Coordinating_Center_Center_for_Research_on_Influenza_Pathogenesis__CRIP_|CEIRS_Data_Processing_a"/>
NNN
</sequence>
<sequence>
<taxon idref="A/Pelican/Atacama/229450-2/2022|EPI_ISL_17885926|A_/_H5N1|Original||2.3.4.4b|26.12.2022|GISAID_EpiFlu_Data_Curator|||19.07.2023|CEIRS_Data_Processing_and_Coordinating_Center_Center_for_Research_on_Influenza_Pathogenesis__CRIP_|CEIRS_Data_Processing_a"/>
NNN
</sequence>
<sequence>
<taxon idref="A/Pelican/Atacama/230158-1/2022|EPI_ISL_17885925|A_/_H5N1|Original||2.3.4.4b|30.12.2022|GISAID_EpiFlu_Data_Curator|||19.07.2023|CEIRS_Data_Processing_and_Coordinating_Center_Center_for_Research_on_Influenza_Pathogenesis__CRIP_|CEIRS_Data_Processing_a"/>
NNN

</sequence>
<sequence>
<taxon idref="A/Pelican/Atacama/230158-2/2022|EPI_ISL_17885924|A_/_H5N1|Original||2.3.4.4b|30.12.2022|GISAID_EpiFlu_Data_Curator|||29.06.2023|CEIRS_Data_Processing_and_Coordinating_Center_Center_for_Research_on_Influenza_Pathogenesis__CRIP_|CEIRS_Data_Processing_a"/>
NNN
</sequence>
<sequence>
<taxon idref="A/Pelican/Chile/227023-1/2022|EPI_ISL_17050893|A_/_H5N1|||2.3.4.4b|07.12.2022||||26.02.2023|||HA|4|A/Pelican/Chile/227023-1/2022|EPI2427757|DNA_IN"/>
NNN
</sequence>
<sequence>
<taxon idref="A/Pelican/Chile/6618-1/2022|EPI_ISL_17011962|A_/_H5N1|||2.3.4.4b|05.12.2022||||22.02.2023|||HA|4|A/Pelican/Chile/6618-1/2022|EPI2416961|DNA_IN"/>
NNN
</sequence>
<sequence>
<taxon idref="A/Pelican/Chile/6618-2/2022|EPI_ISL_17011961|A_/_H5N1|||2.3.4.4b|05.12.2022||||22.02.2023|||HA|4|A/Pelican/Chile/6618-2/2022|EPI2416955|DNA_IN"/>
NNN
</sequence>
<sequence>
<taxon idref="A/Pelican/Chile/6924-1/2022|EPI_ISL_17011963|A_/_H5N1|||2.3.4.4b|06.12.2022||||22.02.2023|||HA|4|A/Pelican/Chile/6924-1/2022|EPI2417011|DNA_IN"/>
NNN
</sequence>
<sequence>
<taxon idref="A/Pelican/Chile/6955-1/2022|EPI_ISL_17011956|A_/_H5N1|||2.3.4.4b|06.12.2022||||22.02.2023|||HA|4|A/Pelican/Chile/6955-1/2022|EPI2416941|DNA_IN"/>
NNN
</sequence>
<sequence>
<taxon idref="A/Pelican/Chile/6955-3/2022|EPI_ISL_17011957|A_/_H5N1|||2.3.4.4b|06.12.2022||||22.02.2023|||HA|4|A/Pelican/Chile/6955-3/2022|EPI2416946|DNA_IN"/>
NNN
</sequence>
<sequence>
<taxon idref="A/Pelican/Chile/6958-1/2022|EPI_ISL_17011959|A_/_H5N1|||2.3.4.4b|06.12.2022||||22.02.2023|||HA|4|A/Pelican/Chile/6958-1/2022|EPI2416967|DNA_IN"/>
NNN
</sequence>
<sequence>
<taxon idref="A/Pelican/Chile/7087-1/2022|EPI_ISL_17012018|A_/_H5N1|||2.3.4.4b|07.12.2022||||22.02.2023|||HA|4|A/Pelican/Chile/7087-1/2022|EPI2417003|DNA_IN"/>
NNN
</sequence>
<sequence>
<taxon idref="A/Pelican/Coquimbo/230310-1/2023|EPI_ISL_17885923|A_/_H5N1|Original||2.3.4.4b|02.01.2023|GISAID_EpiFlu_Data_Curator|||19.07.2023|CEIRS_Data_Processing_and_Coordinating_Center_C

enter_for_Research_on_Influenza_Pathogenesis__CRIP_|CEIRS_Data_Processing_"
/>
NNN
</sequence>
<sequence>
<taxon idref="A/Pelican/Coquimbo/231946-
1/2023|EPI_ISL_17885922|A_/_H5N1|Original||2.3.4.4b|14.01.2023|GISAID_EpiFl
u_Data_Curator|||24.07.2023|CEIRS_Data_Processing_and_Coordinating_Center_C
enter_for_Research_on_Influenza_Pathogenesis__CRIP_|CEIRS_Data_Processing_"
/>
NNN
</sequence>
<sequence>
<taxon idref="A/Pelican/Maule/231155-
2/2023|EPI_ISL_17885921|A_/_H5N1|Original||2.3.4.4b|09.01.2023|GISAID_EpiFl
u_Data_Curator|||19.07.2023|CEIRS_Data_Processing_and_Coordinating_Center_C
enter_for_Research_on_Influenza_Pathogenesis__CRIP_|CEIRS_Data_Processing_a
nd"/>
NNN
</sequence>
<sequence>
<taxon idref="A/Pelican/Nuble/233947-
2/2023|EPI_ISL_17885918|A_/_H5N1|Original||2.3.4.4b|26.01.2023|GISAID_EpiFl
u_Data_Curator|||29.06.2023|CEIRS_Data_Processing_and_Coordinating_Center_C
enter_for_Research_on_Influenza_Pathogenesis__CRIP_|CEIRS_Data_Processing_a
nd"/>
NNN
</sequence>
<sequence>
<taxon idref="A/Pelican/OHiggins/233663-
1/2023|EPI_ISL_17885915|A_/_H5N1|Original||2.3.4.4b|24.01.2023|GISAID_EpiFl
u_Data_Curator|||19.07.2023|CEIRS_Data_Processing_and_Coordinating_Center_C
enter_for_Research_on_Influenza_Pathogenesis__CRIP_|CEIRS_Data_Processing_"
/>
NNN
</sequence>
<sequence>
<taxon idref="A/Pelican/OHiggins/233721-
1/2023|EPI_ISL_17885912|A_/_H5N1|Original||2.3.4.4b|24.01.2023|GISAID_EpiFl
u_Data_Curator|||19.07.2023|CEIRS_Data_Processing_and_Coordinating_Center_C
enter_for_Research_on_Influenza_Pathogenesis__CRIP_|CEIRS_Data_Processing_"
/>
NNN
</sequence>
<sequence>
<taxon idref="A/Pelican/Tarapaca/227436-
2/2022|EPI_ISL_17885942|A_/_H5N1|Original||2.3.4.4b|09.12.2022|GISAID_EpiFl
u_Data_Curator|||19.07.2023|CEIRS_Data_Processing_and_Coordinating_Center_C
enter_for_Research_on_Influenza_Pathogenesis__CRIP_|CEIRS_Data_Processing_"
/>
NNN
</sequence>
<sequence>
<taxon idref="A/Peregrine_Falcon/BC/AIVPHL-
1151/2023|EPI_ISL_18665598|A_/_H5N1|Original||2.3.4.4b|05.06.2023|Caleta_Je
ssica_Marija|||21.12.2023|B.C._Centre_for_Disease_Control|Public_Health_Age
ncy_of_Canada|HA|4|A/Peregrine_Falcon/BC/AIVPHL-1151/2023_HA|EPI2856694|"/>
NNN
</sequence>
<sequence>

<taxon idref="A/Peregrine_Falcon/BC/AIVPHL-877/2023|EPI_ISL_18665560|A_/_H5N1|Original||2.3.4.4b|09.01.2023|Caleta_Jessica_Marija|||21.12.2023|B.C._Centre_for_Disease_Control|Public_Health_Agency_of_Canada|HA|4|A/Peregrine_Falcon/BC/AIVPHL-877/2023_HA|EPI2856420|DN"/>
NNN
</sequence>
<sequence>
<taxon idref="A/Peruvean_booby/Atacama/230579-1/2023|EPI_ISL_19139974|A_/_H5N1|Original||2.3.4.4b|03.01.2023||||15.05.2024|Emory_University__CEIRR_||HA|4|A/Peruvean_booby/Atacama/230579-1/2023_HA|EPI3293636|DNA_IN"/>
NNN
</sequence>
<sequence>
<taxon idref="A/Peruvean_booby/OHiggins/234887-1/2023|EPI_ISL_19162636|A_/_H5N1|Original||2.3.4.4b|31.01.2023||||24.05.2024|Emory_University__CEIRR_||HA|4|A/Peruvean_booby/OHiggins/234887-1/2023_HA|EPI3333386|DNA_IN"/>
NNN
</sequence>
<sequence>
<taxon idref="A/Peruvean_booby/Valparaiso/238535/2023|EPI_ISL_19131163|A_/_H5N1|Original||2.3.4.4b|24.02.2023||||10.05.2024|CEIRS_Data_Processing_and_Coordinating_Center_Center_for_Research_on_Influenza_Pathogenesis__CRIP_||HA|4|A/Peruvean_booby/Valparaiso/238535/2"/>
NNN
</sequence>
<sequence>
<taxon idref="A/Peruvian_booby/Antofagasta/236109-1/2023|EPI_ISL_18690723|A_/_H5N1|Original||2.3.4.4b|10.02.2023||||26.12.2023|Emory_University__CEIRR_|Emory_University__CEIRR_|HA|4|A/Peruvian_booby/Antofagasta/236109-1/2023_HA|EPI2868235|DNA_IN"/>
NNN
</sequence>
<sequence>
<taxon idref="A/Peruvian_booby/OHiggins/242755-1/2023|EPI_ISL_18760067|A_/_H5N1|Original||2.3.4.4b|17.03.2023||||14.01.2024|Emory_University__CEIRR_|Emory_University__CEIRR_|HA|4|A/Peruvian_booby/OHiggins/242755-1/2023_HA|EPI2913229|DNA_IN"/>
NNN
</sequence>
<sequence>
<taxon idref="A/Pluvialis_dominica/Sao_Paulo/2252-N/2023|EPI_ISL_19215181|A_/_H5N1|Original||2.3.4.4b|08.11.2023||||25.06.2024|Ministerio_da_Agricultura_e_Pecuaria||HA|4|A/Pluvialis_dominica/BertiogaBR/2252-N/2023_HA|EPI3395076|DNA_IN"/>
NNN
</sequence>
<sequence>
<taxon idref="A/Procellaria_aequinoctialis/Sao_Paulo/2259-N/2023|EPI_ISL_19215182|A_/_H5N1|Original||2.3.4.4b|09.11.2023||||25.06.2024|Ministerio_da_Agricultura_e_Pecuaria||HA|4|A/Procellaria_aequinoctialis/SaoSebastiaoBR/2259-N/2023_HA|EPI3395084|DNA_IN"/>
NNN
</sequence>
<sequence>
<taxon idref="A/Procellaria_aequinoctialis/Sao_Paulo/2271-N/2023|EPI_ISL_19215190|A_/_H5N1|Original||2.3.4.4b|10.11.2023||||25.06.2024|Ministerio_da_Agricultura_e_Pecuaria||HA|4|A/Procellaria_aequinoctialis/UbatubaBR/2271-N/2023_HA|EPI3395148|DNA_IN"/>
NNN

</sequence>
<sequence>
<taxon idref="A/Red-Tailed_Hawk/ON/FAV-0002-1/2022|EPI_ISL_19155113|A_/_H5N1|||2.3.4.4b|16.12.2022|Signore_Anthony|||21.05.2024|Canadian_Food_Inspection_Agency|Canadian_Food_Inspection_Agency|HA|4|A/Red-Tailed_Hawk/ON/FAV-0002-1/2022_HA|EPI3315584|DNA_IN"/>
NNN
</sequence>
<sequence>
<taxon idref="A/Red_Tailed_Hawk/BC/AIVPHL-1007/2023|EPI_ISL_18665553|A_/_H5N1|Original||2.3.4.4b|20.03.2023|Caleta_Jessica_Marija|||21.12.2023|B.C._Centre_for_Disease_Control|Public_Health_Agency_of_Canada|HA|4|A/Red_Tailed_Hawk/BC/AIVPHL-1007/2023_HA|EPI2856367|DN"/>
NNN
</sequence>
<sequence>
<taxon idref="A/Red_Tailed_Hawk/BC/AIVPHL-1009/2023|EPI_ISL_18665559|A_/_H5N1|Original||2.3.4.4b|20.03.2023|Caleta_Jessica_Marija|||21.12.2023|B.C._Centre_for_Disease_Control|Public_Health_Agency_of_Canada|HA|4|A/Red_Tailed_Hawk/BC/AIVPHL-1009/2023_HA|EPI2856413|DN"/>
NNN
</sequence>
<sequence>
<taxon idref="A/Red_Tailed_Hawk/BC/AIVPHL-1028/2023|EPI_ISL_18665567|A_/_H5N1|Original||2.3.4.4b|27.03.2023|Caleta_Jessica_Marija|||21.12.2023|B.C._Centre_for_Disease_Control|Public_Health_Agency_of_Canada|HA|4|A/Red_Tailed_Hawk/BC/AIVPHL-1028/2023_HA|EPI2856469|DN"/>
NNN
</sequence>
<sequence>
<taxon idref="A/Red_Tailed_Hawk/BC/AIVPHL-859/2022|EPI_ISL_18665549|A_/_H5N1|Original||2.3.4.4b|30.12.2022|Caleta_Jessica_Marija|||21.12.2023|B.C._Centre_for_Disease_Control|Public_Health_Agency_of_Canada|HA|4|A/Red_Tailed_Hawk/BC/AIVPHL-859/2022_HA|EPI2856330|DNA_"/>
NNN
</sequence>
<sequence>
<taxon idref="A/Red_Tailed_Hawk/BC/AIVPHL-860/2022|EPI_ISL_18665552|A_/_H5N1|Original||2.3.4.4b|30.12.2022|Caleta_Jessica_Marija|||21.12.2023|B.C._Centre_for_Disease_Control|Public_Health_Agency_of_Canada|HA|4|A/Red_Tailed_Hawk/BC/AIVPHL-860/2022_HA|EPI2856355|DNA_"/>
NNN
</sequence>
<sequence>
<taxon idref="A/Red_Tailed_Hawk/BC/AIVPHL-931/2023|EPI_ISL_18665508|A_/_H5N1|Original||2.3.4.4b|30.01.2023|Caleta_Jessica_Marija|||21.12.2023|B.C._Centre_for_Disease_Control|Public_Health_Agency_of_Canada|HA|4|A/Red_Tailed_Hawk/BC/AIVPHL-931/2023_HA|EPI2856029|DNA_"/>
NNN
</sequence>
<sequence>
<taxon idref="A/Red_Tailed_Hawk/BC/AIVPHL-990/2023|EPI_ISL_18665544|A_/_H5N1|Original||2.3.4.4b|13.03.2023|Caleta_Jessica_Marija|||21.12.2023|B.C._Centre_for_Disease_Control|Public_Health_Agen

cy_of_Canada|HA|4|A/Red_Tailed_Hawk/BC/AIVPHL-
990/2023_HA|EPI2856299|DNA_"/>
NNN
</sequence>
<sequence>
<taxon idref="A/Sanderling/Arica_y_Parinacota/230758-
1/2022|EPI_ISL_17885980|A_/_H5N1|Original||2.3.4.4b|30.12.2022|GISAID_EpiFl
u_Data_Curator|||19.07.2023|CEIRS_Data_Processing_and_Coordinating_Center_C
enter_for_Research_on_Influenza_Pathogenesis__CRIP_|CEIRS_Dat"/>
NNN
</sequence>
<sequence>
<taxon idref="A/Sanderling/Arica_y_Parinacota/240265/2023|EPI_ISL_17885978|
A_/_H5N1|Original||2.3.4.4b|03.03.2023|GISAID_EpiFlu_Data_Curator|||29.06.2
023|CEIRS_Data_Processing_and_Coordinating_Center_Center_for_Research_on_In
fluenza_Pathogenesis__CRIP_|CEIRS_Data_"/>
NNN
</sequence>
<sequence>
<taxon idref="A/Sanderling/Chile/230758-
2/2023|EPI_ISL_19404931|A_/_H5N1|Original||2.3.4.4b|17.03.2023||||10.09.202
4|CEIRS_Data_Processing_and_Coordinating_Center_Center_for_Research_on_Infl
uenza_Pathogenesis__CRIP_|CEIRS_Data_Processing_and_Coordinating_Center_Ce"
/>
NNN
</sequence>
<sequence>
<taxon idref="A/Sea_Lion/Argentina/3849-
4/2023|EPI_ISL_18698754|A_/_H5N1|||2.3.4.4b|08.08.2023|Benedetti_Estefania|
||03.01.2024|Direccion_del_Laboratorio_Animal_Direccion_General_de_Laborato
rios_y_Control_Tecnico_SENASA.|Instituto_Nacional_de_Enfermedades_Infecci"/
>
NNN
</sequence>
<sequence>
<taxon idref="A/Sea_Lion/Argentina/3893-
1/2023|EPI_ISL_18698755|A_/_H5N1|||2.3.4.4b|11.08.2023|Benedetti_Estefania|
||03.01.2024|Direccion_del_Laboratorio_Animal_Direccion_General_de_Laborato
rios_y_Control_Tecnico_SENASA.|Instituto_Nacional_de_Enfermedades_Infecci"/
>
NNN
</sequence>
<sequence>
<taxon idref="A/Sea_Lion/Peru/LIM-
SER036/2023|EPI_ISL_18054502|A_/_H5N1|Original||2.3.4.4b|23.01.2023|GISAID_
EpiFlu_Data_Curator|||01.08.2023|||HA|4|A/Sea_Lion/Peru/LIM-
SER036/2023_HA|EPI2664215|DNA_IN"/>
NNN
</sequence>
<sequence>
<taxon idref="A/Snow_Goose/AB/FAV-0026-
1/2022|EPI_ISL_19155037|A_/_H5N1|||2.3.4.4b|02.12.2022|Signore_Anthony|||21
.05.2024|Canadian_Food_Inspection_Agency|Canadian_Food_Inspection_Agency|HA
|4|A/Snow_Goose/AB/FAV-0026-1/2022_HA|EPI3314976|DNA_IN"/>
NNN
</sequence>
<sequence>
<taxon idref="A/South_American_fur_seal/Argentina/RN-
PB019/2023|EPI_ISL_18945320|A_/_H5N1|Original||2.3.4.4b|26.08.2023||||01.03
.2024|Instituto_Nacional_de_Tecnologia_Agropecuaria_Instituto_de_Virologia_
e_Innovaciones_Tecnologicas||HA|4|A/South_American_fur_seal/Ar"/>

NNN
</sequence>
<sequence>
<taxon idref="A/South_American_sea_lion/Argentina/RN-
PB007/2023|EPI_ISL_18945319|A_/_H5N1|Original||2.3.4.4b|26.08.2023||||01.03
.2024|Instituto_Nacional_de_Tecnologia_Agropecuaria_Instituto_de_Virologia_
e_Innovaciones_Tecnologicas||HA|4|A/South_American_sea_lion/Ar"/>
NNN
</sequence>
<sequence>
<taxon idref="A/South_American_sea_lion/Argentina/RN-
PB011/2023|EPI_ISL_18945317|A_/_H5N1|Original||2.3.4.4b|26.08.2023||||01.03
.2024|Instituto_Nacional_de_Tecnologia_Agropecuaria_Instituto_de_Virologia_
e_Innovaciones_Tecnologicas||HA|4|A/South_American_sea_lion/Ar"/>
NNN
</sequence>
<sequence>
<taxon idref="A/South_American_sea_lion/Argentina/RN-
PB013/2023|EPI_ISL_18945316|A_/_H5N1|Original||2.3.4.4b|26.08.2023||||01.03
.2024|Instituto_Nacional_de_Tecnologia_Agropecuaria_Instituto_de_Virologia_
e_Innovaciones_Tecnologicas||HA|4|A/South_American_sea_lion/Ar"/>
NNN
</sequence>
<sequence>
<taxon idref="A/South_American_tern/Argentina/CH-
PD030/2023|EPI_ISL_19466158|A_/_H5N1|Original||2.3.4.4b|10.10.2023||||08.10
.2024|Instituto_Nacional_de_Tecnologia_Agropecuaria_Instituto_de_Virologia_
e_Innovaciones_Tecnologicas||HA|4|A/South_American_tern/Argentina/"/>
NNN
</sequence>
<sequence>
<taxon idref="A/South_American_tern/Argentina/CH-
PD037/2023|EPI_ISL_19466210|A_/_H5N1|Original||2.3.4.4b|10.10.2023||||08.10
.2024|Instituto_Nacional_de_Tecnologia_Agropecuaria_Instituto_de_Virologia_
e_Innovaciones_Tecnologicas||HA|4|A/South_American_tern/Argentina/"/>
NNN
</sequence>
<sequence>
<taxon idref="A/South_American_tern/Argentina/RN-
PB015/2023|EPI_ISL_18945315|A_/_H5N1|Original||2.3.4.4b|26.08.2023||||01.03
.2024|Instituto_Nacional_de_Tecnologia_Agropecuaria_Instituto_de_Virologia_
e_Innovaciones_Tecnologicas||HA|4|A/South_American_tern/Argentina/"/>
NNN
</sequence>
<sequence>
<taxon idref="A/South_American_tern/Maule/238507/2023|EPI_ISL_17885973|A_/_
H5N1|Original||2.3.4.4b|23.02.2023|GISAID_EpiFlu_Data_Curator|||29.06.2023|
CEIRS_Data_Processing_and_Coordinating_Center_Center_for_Research_on_Influe
nza_Pathogenesis__CRIP_|CEIRS_Data_Proc"/>
NNN
</sequence>
<sequence>
<taxon idref="A/South_Georgia_Shag/King_Edward_Cove/141245/2023|EPI_ISL_187
42219|A_/_H5N1|Clinical_Sample||2.3.4.4b|27.11.2023|Mollett_Benjamin|||25.0
6.2024|Animal_and_Plant_Health_Agency__APHA_|Animal_and_Plant_Health_Agency
__APHA_|HA|4|A/South_Georgia_Shag/King_E"/>
NNN
</sequence>
<sequence>
<taxon idref="A/South_Georgia_Shag/South_Georgia_and_the_South_Sandwich_Isl
ands/88/2023|EPI_ISL_19313558|A_/_H5N1|Original||2.3.4.4b|27.11.2023||||06.

08.2024|Animal_and_Plant_Health_Agency__APHA_||HA|4|A/South_Georgia_Shag/South_Georgia_and_the_South_Sandwich_Island"/>
NNN
</sequence>
<sequence>
<taxon idref="A/South_polar_skua/Antarctica/INACH-UC-UCHILE-SKU1/2024|EPI_ISL_19410261|A_/_H5N1|Original||2.3.4.4b|03.03.2024||||12.09.2024|Emory_University__CEIRR_|Emory_University__CEIRR_|HA|4|A/South_polar_skua/Antarctica/INACH-UC-UCHILE-SKU1/2024_HA|EPI3555175|D"/>
NNN
</sequence>
<sequence>
<taxon idref="A/South_polar_skua/Antarctica/INACH-UC-UCHILE-SKU2/2024|EPI_ISL_19410262|A_/_H5N1|Original||2.3.4.4b|03.03.2024||||12.09.2024|Emory_University__CEIRR_|Emory_University__CEIRR_|HA|4|A/South_polar_skua/Antarctica/INACH-UC-UCHILE-SKU2/2024_HA|EPI3555182|D"/>
NNN
</sequence>
<sequence>
<taxon idref="A/South_polar_skua/Antarctica/INACH-UC-UCHILE-SKU3/2024|EPI_ISL_19410263|A_/_H5N1|Original||2.3.4.4b|03.03.2024||||12.09.2024|Emory_University__CEIRR_|Emory_University__CEIRR_|HA|4|A/South_polar_skua/Antarctica/INACH-UC-UCHILE-SKU3/2024_HA|EPI3555189|D"/>
NNN
</sequence>
<sequence>
<taxon idref="A/South_polar_skua/Antarctica/INACH-UC-UCHILE-SKU4/2024|EPI_ISL_19410264|A_/_H5N1|Original||2.3.4.4b|03.03.2024||||12.09.2024|Emory_University__CEIRR_|Emory_University__CEIRR_|HA|4|A/South_polar_skua/Antarctica/INACH-UC-UCHILE-SKU4/2024_HA|EPI3555196|D"/>
NNN
</sequence>
<sequence>
<taxon idref="A/South_polar_skua/Antarctica/INACH-UC-UCHILE-SKU5/2024|EPI_ISL_19410265|A_/_H5N1|Original||2.3.4.4b|03.03.2024||||12.09.2024|Emory_University__CEIRR_|Emory_University__CEIRR_|HA|4|A/South_polar_skua/Antarctica/INACH-UC-UCHILE-SKU5/2024_HA|EPI3555203|D"/>
NNN
</sequence>
<sequence>
<taxon idref="A/Southern_Elephant_Seal/Jason_Harbour/141027/2023|EPI_ISL_18742221|A_/_H5N1|Clinical_Sample||2.3.4.4b|09.12.2023|Mollett_Benjamin|||25.06.2024|Animal_and_Plant_Health_Agency__APHA_|Animal_and_Plant_Health_Agency__APHA_|HA|4|A/Southern_Elephant_Seal/J"/>
NNN
</sequence>
<sequence>
<taxon idref="A/Southern_Elephant_Seal/Jason_Harbour/141078/2023|EPI_ISL_18742213|A_/_H5N1|Clinical_Sample||2.3.4.4b|09.12.2023|Mollett_Benjamin|||25.06.2024|Animal_and_Plant_Health_Agency__APHA_|Animal_and_Plant_Health_Agency__APHA_|HA|4|A/Southern_Elephant_Seal/J"/>
NNN
</sequence>
<sequence>
<taxon idref="A/Southern_Elephant_Seal/South_Georgia_and_the_South_Sandwich_Islands/120/2023|EPI_ISL_19313566|A_/_H5N1|Original||2.3.4.4b|09.12.2023||||06.08.2024|Animal_and_Plant_Health_Agency__APHA_||HA|4|A/Southern_Elephant_Seal/South_Georgia_and_the_South_Sandwi"/>
NNN
</sequence>
<sequence>

<taxon idref="A/Southern_Elephant_Seal/South_Georgia_and_the_South_Sandwich_Islands/24/2023|EPI_ISL_19313544|A_/_H5N1|Original||2.3.4.4b|09.12.2023||||06.08.2024|Animal_and_Plant_Health_Agency__APHA_||HA|4|A/Southern_Elephant_Seal/South_Georgia_and_the_South_Sandwic"/>
NNN
</sequence>
<sequence>
<taxon idref="A/Southern_Fulmar/South_Georgia_and_the_South_Sandwich_Islands/4/2023|EPI_ISL_19313538|A_/_H5N1|Original||2.3.4.4b|30.10.2023||||06.08.2024|Animal_and_Plant_Health_Agency__APHA_||HA|4|A/Southern_Fulmar/South_Georgia_and_the_South_Sandwich_Islands/4/202"/>
NNN
</sequence>
<sequence>
<taxon idref="A/Southern_elephant_seal/Peninsula_Valdes/HA_CH-PD027/2023|EPI_ISL_19466211|A_/_H5N1|Original||2.3.4.4b|10.10.2023||||08.10.2024|Instituto_Nacional_de_Tecnologia_Agropecuaria_Instituto_de_Virologia_e_Innovaciones_Tecnologicas||HA|4|A/Southern_elephant"/>
NNN
</sequence>
<sequence>
<taxon idref="A/Southern_elephant_seal/Peninsula_Valdes/HA_CH-PD032rain/2023|EPI_ISL_19466216|A_/_H5N1|Original||2.3.4.4b|10.10.2023||||08.10.2024|Instituto_Nacional_de_Tecnologia_Agropecuaria_Instituto_de_Virologia_e_Innovaciones_Tecnologicas||HA|4|A/Southern_elep"/>
NNN
</sequence>
<sequence>
<taxon idref="A/Southern_fulmar/Falkland_Islands/133789/2023|EPI_ISL_18522961|A_/_H5N1|Clinical_Sample||2.3.4.4b|30.10.2023|Mollett_Benjamin|||20.11.2023|Animal_and_Plant_Health_Agency__APHA_|Animal_and_Plant_Health_Agency__APHA_|HA|4|A/Southern_fulmar/Falkland_Isl"/>
NNN
</sequence>
<sequence>
<taxon idref="A/Sterna_hirundo/Espirito_Santo/0155-N/2024|EPI_ISL_19215192|A_/_H5N1|Original||2.3.4.4b|02.02.2024||||25.06.2024|Ministerio_da_Agricultura_e_Pecuaria||HA|4|A/Sterna_hirundo/ItapemirimBR/0155-N/2024_HA|EPI3395164|DNA_IN"/>
NNN
</sequence>
<sequence>
<taxon idref="A/Sterna_hirundo/Espirito_Santo/0448-N/2024|EPI_ISL_19215194|A_/_H5N1|Original||2.3.4.4b|02.03.2024||||25.06.2024|Ministerio_da_Agricultura_e_Pecuaria||HA|4|A/Sterna_hirundo/PiumaBR/0448-N/2024_HA|EPI3395180|DNA_IN"/>
NNN
</sequence>
<sequence>
<taxon idref="A/Sterna_hirundo/Espirito_Santo/1455-N/2023|EPI_ISL_19215183|A_/_H5N1|Original||2.3.4.4b|27.05.2023||||25.06.2024|Ministerio_da_Agricultura_e_Pecuaria||HA|4|A/Sterna_hirundo/EspiritoSanto/1455-N/2023_HA|EPI3395092|DNA_IN"/>
NNN
</sequence>
<sequence>
<taxon idref="A/Sterna_hirundo/Rio_de_Janeiro/0481-R/2024|EPI_ISL_19215196|A_/_H5N1|Original||2.3.4.4b|02.03.2024||||25.06.2024|Ministerio_da_Agricultura_e_Pecuaria||HA|4|A/Sterna_hirundo/SaoFranciscodeItabapoanaBR/0481-R/2024_HA|EPI3395196|DNA_IN"/>
NNN

</sequence>
<sequence>
<taxon idref="A/Sterna_hirundo/Rio_de_Janeiro/0721-N/2024|EPI_ISL_19215195|A_/_H5N1|Original||2.3.4.4b|15.03.2024||||25.06.2024|Ministerio_da_Agricultura_e_Pecuaria||HA|4|A/Sterna_hirundo/RiodasOstrasBR/0721-N/2024_HA|EPI3395188|DNA_IN"/>
NNN
</sequence>
<sequence>
<taxon idref="A/Sterna_hirundo/Santa_Catarina/2261-N/2023|EPI_ISL_19215184|A_/_H5N1|Original||2.3.4.4b|09.11.2023||||25.06.2024|Ministerio_da_Agricultura_e_Pecuaria||HA|4|A/Sterna_hirundo/PenhaBR/2261-N/2023_HA|EPI3395100|DNA_IN"/>
NNN
</sequence>
<sequence>
<taxon idref="A/Striped_Skunk/BC/AIVPHL-1080/2023|EPI_ISL_18665585|A_/_H5N1|Original||2.3.4.4b|20.04.2023|Caleta_Jessica_Marija|||21.12.2023|B.C._Centre_for_Disease_Control|Public_Health_Agency_of_Canada|HA|4|A/Striped_Skunk/BC/AIVPHL-1080/2023_HA|EPI2856601|DNA_IN"/>
NNN
</sequence>
<sequence>
<taxon idref="A/Striped_Skunk/BC/AIVPHL-1082/2023|EPI_ISL_18665588|A_/_H5N1|Original||2.3.4.4b|20.04.2023|Caleta_Jessica_Marija|||21.12.2023|B.C._Centre_for_Disease_Control|Public_Health_Agency_of_Canada|HA|4|A/Striped_Skunk/BC/AIVPHL-1082/2023_HA|EPI2856624|DNA_IN"/>
NNN
</sequence>
<sequence>
<taxon idref="A/Striped_Skunk/BC/AIVPHL-1083/2023|EPI_ISL_18665592|A_/_H5N1|Original||2.3.4.4b|20.04.2023|Caleta_Jessica_Marija|||21.12.2023|B.C._Centre_for_Disease_Control|Public_Health_Agency_of_Canada|HA|4|A/Striped_Skunk/BC/AIVPHL-1083/2023_HA|EPI2856648|DNA_IN"/>
NNN
</sequence>
<sequence>
<taxon idref="A/Striped_Skunk/BC/AIVPHL-966/2023|EPI_ISL_18665518|A_/_H5N1|Original||2.3.4.4b|28.02.2023|Caleta_Jessica_Marija|||21.12.2023|B.C._Centre_for_Disease_Control|Public_Health_Agency_of_Canada|HA|4|A/Striped_Skunk/BC/AIVPHL-966/2023_HA|EPI2856103|DNA_INS"/>
NNN
</sequence>
<sequence>
<taxon idref="A/Sula_leucogaster/Brazil-PR/A0001-IBTEC/2023|EPI_ISL_18975372|A_/_H5N1|Original||2.3.4.4b|25.07.2023|Hurtado_Eliana||AFLU1|10.08.2024|Laboratorio_de_Ecologia_e_Conservacao|Sao_Paulo_State_University_-_Biotechnology_Institute|HA|4|A/Sula_leucogaster/B"/>
NNN
</sequence>
<sequence>
<taxon idref="A/Sula_leucogaster/Sao_Francisco_do_Sul/2122-N/2023|EPI_ISL_19215185|A_/_H5N1|Original||2.3.4.4b|27.09.2023||||25.06.2024|Ministerio_da_Agricultura_e_Pecuaria||HA|4|A/Sula_leucogaster/Sao_Francisco_do_Sul/2122-N/2023_HA|EPI3395108|DNA_IN"/>
NNN
</sequence>

<sequence>
<taxon idref="A/Swainson_s_hawk/Colorado/23-014329-001/2023|EPI_ISL_17964959|A_/_H5N1|Original||2.3.4.4b|02.05.2023|Killian_Mary_Lea||23-014329-001|06.07.2023|National_Veterinary_Services_Laboratories_-_USDA|National_Veterinary_Services_Laboratories_-_USDA|HA|4|HA_"/>
NNN
</sequence>
<sequence>
<taxon idref="A/Thalasseus_acuflavidus/Brazil-ES/23ES1A0009/2023|EPI_ISL_18130622|A_/_H5N1|||2.3.4.4b|21.06.2023|Ferreira_Helena_Lage|Thalasseus_acuflavidus/Brazil-ES/23ES1A0009/2023||24.08.2023|University_of_Sao_Paulo|University_of_Sao_Paulo|HA|4|A/Thalasseus_acuf"/>
NNN
</sequence>
<sequence>
<taxon idref="A/Thalasseus_acuflavidus/Brazil-ES/23ES1A0025/2023|EPI_ISL_18130627|A_/_H5N1|||2.3.4.4b|24.06.2023|Ferreira_Helena_Lage|A/Thalasseus_acuflavidus/Brazil-ES/23ES1A0025/2023|A/Thalasseus_acuflavidus/Brazil-ES/23ES1A0025/2023|24.08.2023|University_of_Sao_"/>
NNN
</sequence>
<sequence>
<taxon idref="A/Thalasseus_acuflavidus/Parana/2277-N/2023|EPI_ISL_19215199|A_/_H5N1|Original||2.3.4.4b|14.11.2023||||25.06.2024|Ministerio_da_Agricultura_e_Pecuaria||HA|4|A/Thalasseus_acuflavidus/MatinhosBR/2277-N/2023_HA|EPI3395220|DNA_IN"/>
NNN
</sequence>
<sequence>
<taxon idref="A/Thalasseus_acuflavidus/Sao_Paulo/0291-N/2024|EPI_ISL_19215197|A_/_H5N1|Original||2.3.4.4b|22.02.2024||||25.06.2024|Ministerio_da_Agricultura_e_Pecuaria||HA|4|A/Thalasseus_acuflavidus/BertiogaBR/0291-N/2024_HA|EPI3395204|DNA_IN"/>
NNN
</sequence>
<sequence>
<taxon idref="A/Thalasseus_acuflavidus/Sao_Paulo/2280-N/2023|EPI_ISL_19215198|A_/_H5N1|Original||2.3.4.4b|06.11.2023||||25.06.2024|Ministerio_da_Agricultura_e_Pecuaria||HA|4|A/Thalasseus_acuflavidus/IlhaCompridaBR/2280-N/2023_HA|EPI3395212|DNA_IN"/>
NNN
</sequence>
<sequence>
<taxon idref="A/Thalasseus_maximus/Brazil-ES/23ES1A0008/2023|EPI_ISL_18130597|A_/_H5N1|||2.3.4.4b|21.06.2023|Ferreira_Helena_Lage|A/Thalasseus_maximus/Brazil-ES/23ES1A0008/2023|A/Thalasseus_maximus/Brazil-ES/23ES1A0008/2023|24.08.2023|University_of_Sao_Paulo|Univer"/>
NNN
</sequence>
<sequence>
<taxon idref="A/Thalasseus_maximus/Parana/1775-N/2023|EPI_ISL_19215186|A_/_H5N1|Original||2.3.4.4b|23.06.2023||||25.06.2024|Ministerio_da_Agricultura_e_Pecuaria||HA|4|A/Thalasseus_maximus/Antonina/1775-N/2023_HA|EPI3395116|DNA_IN"/>
NNN
</sequence>
<sequence>

<taxon idref="A/Thalasseus_maximus/Rio_Grande_do_Sul/2177-N/2023|EPI_ISL_19215188|A_/_H5N1|Original||2.3.4.4b|13.10.2023||||25.06.2024|Ministerio_da_Agricultura_e_Pecuaria||HA|4|A/Thalasseus_maximus/RioGrandeSul/2177-N/2023_HA|EPI3395132|DNA_IN"/>
NNN
</sequence>
<sequence>
<taxon idref="A/Thalasseus_maximus/Santa_Catarina/1941-N/2023|EPI_ISL_19215187|A_/_H5N1|Original||2.3.4.4b|25.09.2023||||25.06.2024|Ministerio_da_Agricultura_e_Pecuaria||HA|4|A/Thalasseus_maximus/Itapoa/1941-N/2023_HA|EPI3395124|DNA_IN"/>
NNN
</sequence>
<sequence>
<taxon idref="A/Thalasseus_maximus/Sao_Paulo/1546-N/2023|EPI_ISL_19215189|A_/_H5N1|Original||2.3.4.4b|05.06.2023||||25.06.2024|Ministerio_da_Agricultura_e_Pecuaria||HA|4|A/Thalasseus_maximus/Ubatuba/1546-N/2023_HA|EPI3395140|DNA_IN"/>
NNN
</sequence>
<sequence>
<taxon idref="A/Thalasseus_maximus/Sao_Paulo/2339-N/2023|EPI_ISL_19215200|A_/_H5N1|Original||2.3.4.4b|13.12.2023||||25.06.2024|Ministerio_da_Agricultura_e_Pecuaria||HA|4|A/Thalasseus_maximus/PraiaGrandeBR/2339-N/2023_HA|EPI3395228|DNA_IN"/>
NNN
</sequence>
<sequence>
<taxon idref="A/Trumpeter_Swan/BC/AIVPHL-1003/2023|EPI_ISL_18665550|A_/_H5N1|Original||2.3.4.4b|20.03.2023|Caleta_Jessica_Marija|||21.12.2023|B.C._Centre_for_Disease_Control|Public_Health_Agency_of_Canada|HA|4|A/Trumpeter_Swan/BC/AIVPHL-1003/2023_HA|EPI2856347|DNA_"/>
NNN
</sequence>
<sequence>
<taxon idref="A/Trumpeter_Swan/BC/AIVPHL-916/2023|EPI_ISL_18665494|A_/_H5N1|Original||2.3.4.4b|27.01.2023|Caleta_Jessica_Marija|||21.12.2023|B.C._Centre_for_Disease_Control|Public_Health_Agency_of_Canada|HA|4|A/Trumpeter_Swan/BC/AIVPHL-916/2023_HA|EPI2855919|DNA_IN"/>
NNN
</sequence>
<sequence>
<taxon idref="A/Trumpeter_Swan/BC/FAV-0053-25/2022|EPI_ISL_19155139|A_/_H5N1|||2.3.4.4b|31.12.2022|Signore_Anthony|||21.05.2024|Canadian_Food_Inspection_Agency|Canadian_Food_Inspection_Agency|HA|4|A/Trumpeter_Swan/BC/FAV-0053-25/2022_HA|EPI3315792|DNA_IN"/>
NNN
</sequence>
<sequence>
<taxon idref="A/Turkey/Argentina/1348-3/2023|EPI_ISL_18698508|A_/_H5N1|||2.3.4.4b|31.03.2023|Benedetti_Estefania|||28.12.2023|Direccion_del_Laboratorio_Animal_Direccion_General_de_Laboratorios_y_Control_Tecnico_SENASA.|Instituto_Nacional_de_Enfermedades_Infeccios"/>
NNN
</sequence>
<sequence>
<taxon idref="A/Turkey/Argentina/1710-1/2023|EPI_ISL_18698513|A_/_H5N1|||2.3.4.4b|17.04.2023|Benedetti_Estefania|

||28.12.2023|Direccion_del_Laboratorio_Animal_Direccion_General_de_Laboratorios_y_Control_Tecnico_SENASA.|Instituto_Nacional_de_Enfermedades_Infeccios"/>
NNN
</sequence>
<sequence>
<taxon idref="A/Turkey/Argentina/1711-2/2023|EPI_ISL_18698514|A_/_H5N1|||2.3.4.4b|19.04.2023|Benedetti_Estefania|||28.12.2023|Direccion_del_Laboratorio_Animal_Direccion_General_de_Laboratorios_y_Control_Tecnico_SENASA.|Instituto_Nacional_de_Enfermedades_Infeccios"/>
NNN
</sequence>
<sequence>
<taxon idref="A/Turkey/Argentina/753-1/2023|EPI_ISL_18698497|A_/_H5N1|||2.3.4.4b|04.03.2023|Benedetti_Estefania|||28.12.2023|Direccion_del_Laboratorio_Animal_Direccion_General_de_Laboratorios_y_Control_Tecnico_SENASA.|Instituto_Nacional_de_Enfermedades_Infecciosa"/>
NNN
</sequence>
<sequence>
<taxon idref="A/Turkey/BC/FAV-1613-1/2022|EPI_ISL_19155056|A_/_H5N1|||2.3.4.4b|05.12.2022|Signore_Anthony|||21.05.2024|Canadian_Food_Inspection_Agency|Canadian_Food_Inspection_Agency|HA|4|A/Turkey/BC/FAV-1613-1/2022_HA|EPI3315128|DNA_IN"/>
NNN
</sequence>
<sequence>
<taxon idref="A/Turkey/BC/FAV-1613-2/2022|EPI_ISL_19155057|A_/_H5N1|||2.3.4.4b|05.12.2022|Signore_Anthony|||21.05.2024|Canadian_Food_Inspection_Agency|Canadian_Food_Inspection_Agency|HA|4|A/Turkey/BC/FAV-1613-2/2022_HA|EPI3315136|DNA_IN"/>
NNN
</sequence>
<sequence>
<taxon idref="A/Turkey_vulture/Antofagasta/228252-1/2022|EPI_ISL_17885970|A_/_H5N1|Original||2.3.4.4b|17.12.2022|GISAID_EpiFlu_Data_Curator|||19.07.2023|CEIRS_Data_Processing_and_Coordinating_Center_Center_for_Research_on_Influenza_Pathogenesis__CRIP_|CEIRS_Data_P"/>
NNN
</sequence>
<sequence>
<taxon idref="A/Turkey_vulture/Valparaiso/230187-1/2022|EPI_ISL_17885968|A_/_H5N1|Original||2.3.4.4b|31.12.2022|GISAID_EpiFlu_Data_Curator|||19.07.2023|CEIRS_Data_Processing_and_Coordinating_Center_Center_for_Research_on_Influenza_Pathogenesis__CRIP_|CEIRS_Data_Pr"/>
NNN
</sequence>
<sequence>
<taxon idref="A/Western_screech_owl/Washington/23-010027-001/2023|EPI_ISL_17964920|A_/_H5N1|Original||2.3.4.4b|23.03.2023|Killian_Mary_Lea||23-010027-001|06.07.2023|National_Veterinary_Services_Laboratories_-_USDA|National_Veterinary_Services_Laboratories_-_USDA|HA"/>
NNN
</sequence>
<sequence>
<taxon idref="A/american_coot/Florida/23-002762-015/2022|EPI_ISL_19606663|A_/_H5N1|Original||2.3.4.4b|19.12.2022||||12.12.2

024|USDA_APHIS_Veterinary_Services_Diagnostic_Virology_Laboratory_National_Veterinary_Services_Laboratories||HA|4|A/american_coot/Florida/2"/>
NNN
</sequence>
<sequence>
<taxon idref="A/american_crow/BC/AIVPHL-330/2022|EPI_ISL_17051463|A_/_H5N1|Original||2.3.4.4b|19.12.2022|Russell_Shannon_Laurel|||28.02.2023|B.C._Centre_for_Disease_Control|British_Columbia_Centre_for_Disease_Control|HA|4|A/american_crow/BC/AIVPHL-330/2022_HA|EPI24"/>
NNN
</sequence>
<sequence>
<taxon idref="A/american_crow/BC/AIVPHL-341/2022|EPI_ISL_17051468|A_/_H5N1|Original||2.3.4.4b|19.12.2022|Russell_Shannon_Laurel|||28.02.2023|B.C._Centre_for_Disease_Control|British_Columbia_Centre_for_Disease_Control|HA|4|A/american_crow/BC/AIVPHL-341/2022_HA|EPI24"/>
NNN
</sequence>
<sequence>
<taxon idref="A/american_crow/BC/AIVPHL-345/2022|EPI_ISL_17051473|A_/_H5N1|Original||2.3.4.4b|30.12.2022|Russell_Shannon_Laurel|||28.02.2023|B.C._Centre_for_Disease_Control|British_Columbia_Centre_for_Disease_Control|HA|4|A/american_crow/BC/AIVPHL-345/2022_HA|EPI24"/>
NNN
</sequence>
<sequence>
<taxon idref="A/american_crow/BC/AIVPHL-350/2022|EPI_ISL_17051471|A_/_H5N1|Original||2.3.4.4b|30.12.2022|Russell_Shannon_Laurel|||28.02.2023|B.C._Centre_for_Disease_Control|British_Columbia_Centre_for_Disease_Control|HA|4|A/american_crow/BC/AIVPHL-350/2022_HA|EPI24"/>
NNN
</sequence>
<sequence>
<taxon idref="A/american_crow/BC/AIVPHL-351/2022|EPI_ISL_17051472|A_/_H5N1|Original||2.3.4.4b|30.12.2022|Russell_Shannon_Laurel|||28.02.2023|B.C._Centre_for_Disease_Control|British_Columbia_Centre_for_Disease_Control|HA|4|A/american_crow/BC/AIVPHL-351/2022_HA|EPI24"/>
NNN
</sequence>
<sequence>
<taxon idref="A/american_crow/Colorado/22-042172-002/2022|EPI_ISL_19606664|A_/_H5N1|Original||2.3.4.4b|19.12.2022||||12.12.2024|USDA_APHIS_Veterinary_Services_Diagnostic_Virology_Laboratory_National_Veterinary_Services_Laboratories||HA|4|A/american_crow/Colorado"/>
NNN
</sequence>
<sequence>
<taxon idref="A/american_crow/Colorado/23-001476-001/2023|EPI_ISL_19606665|A_/_H5N1|Original||2.3.4.4b|05.01.2023||||12.12.2024|USDA_APHIS_Veterinary_Services_Diagnostic_Virology_Laboratory_National_Veterinary_Services_Laboratories||HA|4|A/american_crow/Colorado"/>
NNN
</sequence>
<sequence>
<taxon idref="A/american_crow/Kansas/23-002150-001/2022|EPI_ISL_19606646|A_/_H5N1|Original||2.3.4.4b|23.12.2022||||12.12.2

024|USDA_APHIS_Veterinary_Services_Diagnostic_Virology_Laboratory_National_Veterinary_Services_Laboratories||HA|4|A/american_crow/Kansas/23-"/>
NNN
</sequence>
<sequence>
<taxon idref="A/american_green_winged_teal/Oregon/22-040176-004/2022|EPI_ISL_19606698|A_/_H5N1|Original||2.3.4.4b|05.12.2022||||12.12.2024|USDA_APHIS_Veterinary_Services_Diagnostic_Virology_Laboratory_National_Veterinary_Services_Laboratories||HA|4|A/american_gr"/>
NNN
</sequence>
<sequence>
<taxon idref="A/american_green_winged_teal/Texas/23-000110-004/2022|EPI_ISL_19606685|A_/_H5N1|Original||2.3.4.4b|04.12.2022||||12.12.2024|USDA_APHIS_Veterinary_Services_Diagnostic_Virology_Laboratory_National_Veterinary_Services_Laboratories||HA|4|A/american_gre"/>
NNN
</sequence>
<sequence>
<taxon idref="A/american_wigeon/Alaska/22-041761-007/2022|EPI_ISL_19606535|A_/_H5N1|Original||2.3.4.4b|10.12.2022||||12.12.2024|USDA_APHIS_Veterinary_Services_Diagnostic_Virology_Laboratory_National_Veterinary_Services_Laboratories||HA|4|A/american_wigeon/Alaska"/>
NNN
</sequence>
<sequence>
<taxon idref="A/american_wigeon/Florida/22-040720-004/2022|EPI_ISL_19606537|A_/_H5N1|Original||2.3.4.4b|10.12.2022||||12.12.2024|USDA_APHIS_Veterinary_Services_Diagnostic_Virology_Laboratory_National_Veterinary_Services_Laboratories||HA|4|A/american_wigeon/Flori"/>
NNN
</sequence>
<sequence>
<taxon idref="A/american_wigeon/Oregon/23-003438-001/2023|EPI_ISL_19606553|A_/_H5N1|Original||2.3.4.4b|07.01.2023||||12.12.2024|USDA_APHIS_Veterinary_Services_Diagnostic_Virology_Laboratory_National_Veterinary_Services_Laboratories||HA|4|A/american_wigeon/Oregon"/>
NNN
</sequence>
<sequence>
<taxon idref="A/american_wigeon/Texas/23-003062-016/2023|EPI_ISL_19606613|A_/_H5N1|Original||2.3.4.4b|18.01.2023||||12.12.2024|USDA_APHIS_Veterinary_Services_Diagnostic_Virology_Laboratory_National_Veterinary_Services_Laboratories||HA|4|A/american_wigeon/Texas/2"/>
NNN
</sequence>
<sequence>
<taxon idref="A/american_wigeon/Texas/23-003062-020/2023|EPI_ISL_19606614|A_/_H5N1|Original||2.3.4.4b|18.01.2023||||12.12.2024|USDA_APHIS_Veterinary_Services_Diagnostic_Virology_Laboratory_National_Veterinary_Services_Laboratories||HA|4|A/american_wigeon/Texas/2"/>
NNN
</sequence>
<sequence>
<taxon idref="A/backyard_chicken/Uruguay/UDELAR-040-M5/2023|EPI_ISL_18310942|A_/_H5N1|Original||2.3.4.4b|03.03.2023|GISAID_EpiFlu_Data_Curator|||26.09.2023|||HA|4|A/backyard_chicken/Uruguay/UDELAR-040-M5/2023_HA|EPI2758873|DNA_IN"/>
NNN
</sequence>
<sequence>

<taxon idref="A/backyard_chicken/Uruguay/UDELAR-040-
M7/2023|EPI_ISL_18310967|A_/_H5N1|Original||2.3.4.4b|03.03.2023|GISAID_EpiF
lu_Data_Curator|||26.09.2023|||HA|4|A/backyard_chicken/Uruguay/UDELAR-040-
M7/2023_HA|EPI2758976|DNA_IN"/>
NNN
</sequence>
<sequence>
<taxon idref="A/backyard_chicken/Uruguay/UDELAR-047-
M1/2023|EPI_ISL_18310966|A_/_H5N1|Original||2.3.4.4b|06.03.2023|GISAID_EpiF
lu_Data_Curator|||26.09.2023|||HA|4|A/backyard_chicken/Uruguay/UDELAR-047-
M1/2023_HA|EPI2758968|DNA_IN"/>
NNN
</sequence>
<sequence>
<taxon idref="A/backyard_chicken/Uruguay/UDELAR-047-
M3/2023|EPI_ISL_18310965|A_/_H5N1|Original||2.3.4.4b|06.03.2023|GISAID_EpiF
lu_Data_Curator|||26.09.2023|||HA|4|A/backyard_chicken/Uruguay/UDELAR-047-
M3/2023_HA|EPI2758960|DNA_IN"/>
NNN
</sequence>
<sequence>
<taxon idref="A/backyard_chicken/Uruguay/UDELAR-124-
M1/2023|EPI_ISL_18310964|A_/_H5N1|Original||2.3.4.4b|16.04.2023|GISAID_EpiF
lu_Data_Curator|||26.09.2023|||HA|4|A/backyard_chicken/Uruguay/UDELAR-124-
M1/2023_HA|EPI2758952|DNA_IN"/>
NNN
</sequence>
<sequence>
<taxon idref="A/backyard_chicken/Uruguay/UDELAR-127-
M1/2023|EPI_ISL_18310963|A_/_H5N1|Original||2.3.4.4b|17.04.2023|GISAID_EpiF
lu_Data_Curator|||26.09.2023|||HA|4|A/backyard_chicken/Uruguay/UDELAR-127-
M1/2023_HA|EPI2758944|DNA_IN"/>
NNN
</sequence>
<sequence>
<taxon idref="A/backyard_chicken/Uruguay/UDELAR-127-
M4/2023|EPI_ISL_18310962|A_/_H5N1|Original||2.3.4.4b|17.04.2023|GISAID_EpiF
lu_Data_Curator|||26.09.2023|||HA|4|A/backyard_chicken/Uruguay/UDELAR-127-
M4/2023_HA|EPI2758936|DNA_IN"/>
NNN
</sequence>
<sequence>
<taxon idref="A/backyard_chicken/Uruguay/UDELAR-144-
M3/2023|EPI_ISL_18310961|A_/_H5N1|Original||2.3.4.4b|03.05.2023|GISAID_EpiF
lu_Data_Curator|||26.09.2023|||HA|4|A/backyard_chicken/Uruguay/UDELAR-144-
M3/2023_HA|EPI2758928|DNA_IN"/>
NNN
</sequence>
<sequence>
<taxon idref="A/backyard_duck/Uruguay/UDELAR-124-
M3/2023|EPI_ISL_18310960|A_/_H5N1|Original||2.3.4.4b|16.04.2023|GISAID_EpiF
lu_Data_Curator|||26.09.2023|||HA|4|A/backyard_duck/Uruguay/UDELAR-124-
M3/2023_HA|EPI2758920|DNA_IN"/>
NNN
</sequence>
<sequence>
<taxon idref="A/backyard_turkey/Uruguay/UDELAR-124-
M6/2023|EPI_ISL_18310959|A_/_H5N1|Original||2.3.4.4b|16.04.2023|GISAID_EpiF
lu_Data_Curator|||26.09.2023|||HA|4|A/backyard_turkey/Uruguay/UDELAR-124-
M6/2023_HA|EPI2758912|DNA_IN"/>
NNN

</sequence>
<sequence>
<taxon idref="A/band-tailed_gull/Antofagasta/228525-2/2022|EPI_ISL_19131167|A_/_H5N1|Original||2.3.4.4b|19.12.2022||||10.05.2024|CEIRS_Data_Processing_and_Coordinating_Center_Center_for_Research_on_Influenza_Pathogenesis__CRIP_|CEIRS_Data_Processing_and_Coordinatin"/>
NNN
</sequence>
<sequence>
<taxon idref="A/band-tailed_gull/Tarapaca/238807-1/2023|EPI_ISL_19131176|A_/_H5N1|Original||2.3.4.4b|25.02.2023||||10.05.2024|CEIRS_Data_Processing_and_Coordinating_Center_Center_for_Research_on_Influenza_Pathogenesis__CRIP_||HA|4|A/band-tailed_gull/Tarapaca/238807"/>
NNN
</sequence>
<sequence>
<taxon idref="A/barn_owl/Washington/22-041081-001/2022|EPI_ISL_19607175|A_/_H5N1|Original||2.3.4.4b|12.12.2022||||12.12.2024|USDA_APHIS_Veterinary_Services_Diagnostic_Virology_Laboratory_National_Veterinary_Services_Laboratories||HA|4|A/barn_owl/Washington/22-04"/>
NNN
</sequence>
<sequence>
<taxon idref="A/barred_owl/BC/AIVPHL-346/2022|EPI_ISL_17051474|A_/_H5N1|Original||2.3.4.4b|30.12.2022|Russell_Shannon_Laurel|||28.02.2023|B.C._Centre_for_Disease_Control|British_Columbia_Centre_for_Disease_Control|HA|4|A/barred_owl/BC/AIVPHL-346/2022_HA|EPI2429224|"/>
NNN
</sequence>
<sequence>
<taxon idref="A/black-necked_swan/Los_Rios/247292-1/2023|EPI_ISL_18760070|A_/_H5N1|Original||2.3.4.4b|05.04.2023||||14.01.2024|Emory_University__CEIRR_|Emory_University__CEIRR_|HA|4|A/black-necked_swan/Los_Rios/247292-1/2023_HA|EPI2913251|DNA_IN"/>
NNN
</sequence>
<sequence>
<taxon idref="A/black-necked_swan/Uruguay/UDELAR-014-M3/2023|EPI_ISL_18310958|A_/_H5N1|Original||2.3.4.4b|18.02.2023|GISAID_EpiFlu_Data_Curator|||26.09.2023|||HA|4|A/black-necked_swan/Uruguay/UDELAR-014-M3/2023_HA|EPI2758904|DNA_IN"/>
NNN
</sequence>
<sequence>
<taxon idref="A/black-necked_swan/Uruguay/UDELAR-078-M2/2023|EPI_ISL_18310957|A_/_H5N1|Original||2.3.4.4b|15.03.2023|GISAID_EpiFlu_Data_Curator|||26.09.2023|||HA|4|A/black-necked_swan/Uruguay/UDELAR-078-M2/2023_HA|EPI2758896|DNA_IN"/>
NNN
</sequence>
<sequence>
<taxon idref="A/black_brant/California/23-000279-001/2022|EPI_ISL_19606469|A_/_H5N1|Original||2.3.4.4b|15.12.2022||||12.12.2024|USDA_APHIS_Veterinary_Services_Diagnostic_Virology_Laboratory_National_Veterinary_Services_Laboratories||HA|4|A/black_brant/California"/>
NNN
</sequence>
<sequence>
<taxon idref="A/black_skimmer/Chile/C61962/2022|EPI_ISL_16891402|A_/_H5N1|||2.3.4.4b|02.12.2022||||04.02.2023|St._Jude_Center_of_Excellence_for_Influe

nza_Research_and_Response_-
_CEIRR_Influenza_Data_Processing_and_Communication_Center|St._Jude_Center_o
f_Excellence_f"/>
NNN
</sequence>
<sequence>
<taxon idref="A/brown-hooded_gull/Los_Rios/247093-
1/2023|EPI_ISL_18760071|A_/_H5N1|Original||2.3.4.4b|04.04.2023||||14.01.202
4|Emory_University__CEIRR_|Emory_University__CEIRR_|HA|4|A/brown-
hooded_gull/Los_Rios/247093-1/2023_HA|EPI2913258|DNA_IN"/>
NNN
</sequence>
<sequence>
<taxon idref="A/brown-hooded_gull/Los_Rios/247094-
1/2023|EPI_ISL_18760072|A_/_H5N1|Original||2.3.4.4b|04.04.2023||||14.01.202
4|Emory_University__CEIRR_|Emory_University__CEIRR_|HA|4|A/brown-
hooded_gull/Los_Rios/247094-1/2023_HA|EPI2913263|DNA_IN"/>
NNN
</sequence>
<sequence>
<taxon idref="A/brown_pelican/Panama/22-041124-
001/2022|EPI_ISL_17559276|A_/_H5N1|Original||2.3.4.4b|14.12.2022|Killian_Ma
ry_Lea|M1_LEA-32-22|22-041124-
001|27.04.2023|El_Laboratorio_de_Diagnostico_de_Enfermedades_Vesiculares__L
ADIVES_|National_Veterinary_Services_L"/>
NNN
</sequence>
<sequence>
<taxon idref="A/buff-necked_ibis/Bio_bio/247636-
1/2023|EPI_ISL_19418491|A_/_H5N1|Original||2.3.4.4b|07.04.2023||||17.09.202
4|Emory_University__CEIRR_|Emory_University__CEIRR_|HA|4|A/buff-
necked_ibis/Bio_bio/247636-1/2023_HA|EPI3558280|DNA_IN"/>
NNN
</sequence>
<sequence>
<taxon idref="A/bufflehead/California/23-001056-
003/2022|EPI_ISL_19606428|A_/_H5N1|Original||2.3.4.4b|21.12.2022||||12.12.2
024|USDA_APHIS_Veterinary_Services_Diagnostic_Virology_Laboratory_National_
Veterinary_Services_Laboratories||HA|4|A/bufflehead/California/2"/>
NNN
</sequence>
<sequence>
<taxon idref="A/burmeisters_porpoise/Chile/246506-
2/2023|EPI_ISL_19391458|A_/_H5N1|Original||2.3.4.4b|31.03.2023||||09.09.202
4|CEIRS_Data_Processing_and_Coordinating_Center_Center_for_Research_on_Infl
uenza_Pathogenesis__CRIP_||HA|4|A/burmeisters_porpoise/Chile/2465"/>
NNN
</sequence>
<sequence>
<taxon idref="A/cackling_goose/BC/AIVPHL-
367/2023|EPI_ISL_17051485|A_/_H5N1|Original||2.3.4.4b|16.01.2023|Russell_Sh
annon_Laurel|||28.02.2023|B.C._Centre_for_Disease_Control|British_Columbia_
Centre_for_Disease_Control|HA|4|A/cackling_goose/BC/AIVPHL-
367/2023_HA|EPI"/>
NNN
</sequence>
<sequence>
<taxon idref="A/cackling_goose/California/23-001053-
001/2022|EPI_ISL_19606412|A_/_H5N1|Original||2.3.4.4b|17.12.2022||||12.12.2
024|USDA_APHIS_Veterinary_Services_Diagnostic_Virology_Laboratory_National_
Veterinary_Services_Laboratories||HA|4|A/cackling_goose/Cali"/>

NNN
</sequence>
<sequence>
<taxon idref="A/cackling_goose/Colorado/22-040752-
002/2022|EPI_ISL_19606415|A_/_H5N1|Original||2.3.4.4b|13.12.2022||||12.12.2
024|USDA_APHIS_Veterinary_Services_Diagnostic_Virology_Laboratory_National_
Veterinary_Services_Laboratories||HA|4|A/cackling_goose/Colora"/>
NNN
</sequence>
<sequence>
<taxon idref="A/cackling_goose/Colorado/22-040753-
001/2022|EPI_ISL_19606417|A_/_H5N1|Original||2.3.4.4b|13.12.2022||||12.12.2
024|USDA_APHIS_Veterinary_Services_Diagnostic_Virology_Laboratory_National_
Veterinary_Services_Laboratories||HA|4|A/cackling_goose/Colora"/>
NNN
</sequence>
<sequence>
<taxon idref="A/cackling_goose/Colorado/22-040763-
001/2022|EPI_ISL_19606419|A_/_H5N1|Original||2.3.4.4b|10.12.2022||||12.12.2
024|USDA_APHIS_Veterinary_Services_Diagnostic_Virology_Laboratory_National_
Veterinary_Services_Laboratories||HA|4|A/cackling_goose/Colora"/>
NNN
</sequence>
<sequence>
<taxon idref="A/cackling_goose/Colorado/22-040763-
003/2022|EPI_ISL_19606420|A_/_H5N1|Original||2.3.4.4b|10.12.2022||||12.12.2
024|USDA_APHIS_Veterinary_Services_Diagnostic_Virology_Laboratory_National_
Veterinary_Services_Laboratories||HA|4|A/cackling_goose/Colora"/>
NNN
</sequence>
<sequence>
<taxon idref="A/cackling_goose/Texas/23-001028-
001/2023|EPI_ISL_19606425|A_/_H5N1|Original||2.3.4.4b|06.01.2023||||12.12.2
024|USDA_APHIS_Veterinary_Services_Diagnostic_Virology_Laboratory_National_
Veterinary_Services_Laboratories||HA|4|A/cackling_goose/Texas/23-"/>
NNN
</sequence>
<sequence>
<taxon idref="A/canada_goose/Alaska/22-041059-
001/2022|EPI_ISL_19606393|A_/_H5N1|Original||2.3.4.4b|13.12.2022||||12.12.2
024|USDA_APHIS_Veterinary_Services_Diagnostic_Virology_Laboratory_National_
Veterinary_Services_Laboratories||HA|4|A/canada_goose/Alaska/22-04"/>
NNN
</sequence>
<sequence>
<taxon idref="A/canada_goose/BC/AIVPHL-
321/2022|EPI_ISL_17051438|A_/_H5N1|Original||2.3.4.4b|08.12.2022|Russell_Sh
annon_Laurel|||28.02.2023|B.C._Centre_for_Disease_Control|British_Columbia_
Centre_for_Disease_Control|HA|4|A/canada_goose/BC/AIVPHL-
321/2022_HA|EPI2429"/>
NNN
</sequence>
<sequence>
<taxon idref="A/canada_goose/BC/AIVPHL-
326/2022|EPI_ISL_17051457|A_/_H5N1|Original||2.3.4.4b|12.12.2022|Russell_Sh
annon_Laurel|||28.02.2023|B.C._Centre_for_Disease_Control|British_Columbia_
Centre_for_Disease_Control|HA|4|A/canada_goose/BC/AIVPHL-
326/2022_HA|EPI2429"/>
NNN
</sequence>
<sequence>

<taxon idref="A/canada_goose/California/22-041399-
001/2022|EPI_ISL_19607747|A_/_H5N1|Original||2.3.4.4b|01.12.2022||||12.12.2
024|USDA_APHIS_Veterinary_Services_Diagnostic_Virology_Laboratory_National_
Veterinary_Services_Laboratories||HA|4|A/canada_goose/Californ"/>
NNN
</sequence>
<sequence>
<taxon idref="A/canada_goose/California/22-041399-
002/2022|EPI_ISL_19607748|A_/_H5N1|Original||2.3.4.4b|01.12.2022||||12.12.2
024|USDA_APHIS_Veterinary_Services_Diagnostic_Virology_Laboratory_National_
Veterinary_Services_Laboratories||HA|4|A/canada_goose/Californ"/>
NNN
</sequence>
<sequence>
<taxon idref="A/canada_goose/Colorado/22-040763-
002/2022|EPI_ISL_19607758|A_/_H5N1|Original||2.3.4.4b|11.12.2022||||12.12.2
024|USDA_APHIS_Veterinary_Services_Diagnostic_Virology_Laboratory_National_
Veterinary_Services_Laboratories||HA|4|A/canada_goose/Colorado/2"/>
NNN
</sequence>
<sequence>
<taxon idref="A/canada_goose/Colorado/22-041829-
001/2022|EPI_ISL_19607759|A_/_H5N1|Original||2.3.4.4b|19.12.2022||||12.12.2
024|USDA_APHIS_Veterinary_Services_Diagnostic_Virology_Laboratory_National_
Veterinary_Services_Laboratories||HA|4|A/canada_goose/Colorado/2"/>
NNN
</sequence>
<sequence>
<taxon idref="A/canada_goose/Colorado/22-042165-
001/2022|EPI_ISL_19607760|A_/_H5N1|Original||2.3.4.4b|24.12.2022||||12.12.2
024|USDA_APHIS_Veterinary_Services_Diagnostic_Virology_Laboratory_National_
Veterinary_Services_Laboratories||HA|4|A/canada_goose/Colorado/2"/>
NNN
</sequence>
<sequence>
<taxon idref="A/canada_goose/Colorado/22-042169-
001/2022|EPI_ISL_19607762|A_/_H5N1|Original||2.3.4.4b|27.12.2022||||12.12.2
024|USDA_APHIS_Veterinary_Services_Diagnostic_Virology_Laboratory_National_
Veterinary_Services_Laboratories||HA|4|A/canada_goose/Colorado/2"/>
NNN
</sequence>
<sequence>
<taxon idref="A/canada_goose/Colorado/23-000399-
001/2022|EPI_ISL_19607792|A_/_H5N1|Original||2.3.4.4b|30.12.2022||||12.12.2
024|USDA_APHIS_Veterinary_Services_Diagnostic_Virology_Laboratory_National_
Veterinary_Services_Laboratories||HA|4|A/canada_goose/Colorado/2"/>
NNN
</sequence>
<sequence>
<taxon idref="A/canada_goose/Colorado/23-000496-
001/2023|EPI_ISL_19607863|A_/_H5N1|Original||2.3.4.4b|04.01.2023||||12.12.2
024|USDA_APHIS_Veterinary_Services_Diagnostic_Virology_Laboratory_National_
Veterinary_Services_Laboratories||HA|4|A/canada_goose/Colorado/2"/>
NNN
</sequence>
<sequence>
<taxon idref="A/canada_goose/Colorado/23-000497-
001/2023|EPI_ISL_19607793|A_/_H5N1|Original||2.3.4.4b|01.01.2023||||12.12.2
024|USDA_APHIS_Veterinary_Services_Diagnostic_Virology_Laboratory_National_
Veterinary_Services_Laboratories||HA|4|A/canada_goose/Colorado/2"/>
NNN

</sequence>
<sequence>
<taxon idref="A/canada_goose/Colorado/23-000621-001/2023|EPI_ISL_19607820|A_/_H5N1|Original||2.3.4.4b|04.01.2023||||12.12.2024|USDA_APHIS_Veterinary_Services_Diagnostic_Virology_Laboratory_National_Veterinary_Services_Laboratories||HA|4|A/canada_goose/Colorado/2"/>
NNN
</sequence>
<sequence>
<taxon idref="A/canada_goose/Colorado/23-000622-001/2023|EPI_ISL_19607821|A_/_H5N1|Original||2.3.4.4b|04.01.2023||||12.12.2024|USDA_APHIS_Veterinary_Services_Diagnostic_Virology_Laboratory_National_Veterinary_Services_Laboratories||HA|4|A/canada_goose/Colorado/2"/>
NNN
</sequence>
<sequence>
<taxon idref="A/canada_goose/Colorado/23-000623-001/2023|EPI_ISL_19607822|A_/_H5N1|Original||2.3.4.4b|03.01.2023||||12.12.2024|USDA_APHIS_Veterinary_Services_Diagnostic_Virology_Laboratory_National_Veterinary_Services_Laboratories||HA|4|A/canada_goose/Colorado/2"/>
NNN
</sequence>
<sequence>
<taxon idref="A/canada_goose/Colorado/23-003283-001/2023|EPI_ISL_19607823|A_/_H5N1|Original||2.3.4.4b|19.01.2023||||12.12.2024|USDA_APHIS_Veterinary_Services_Diagnostic_Virology_Laboratory_National_Veterinary_Services_Laboratories||HA|4|A/canada_goose/Colorado/2"/>
NNN
</sequence>
<sequence>
<taxon idref="A/canada_goose/Colorado/23-003284-001/2023|EPI_ISL_19607824|A_/_H5N1|Original||2.3.4.4b|20.01.2023||||12.12.2024|USDA_APHIS_Veterinary_Services_Diagnostic_Virology_Laboratory_National_Veterinary_Services_Laboratories||HA|4|A/canada_goose/Colorado/2"/>
NNN
</sequence>
<sequence>
<taxon idref="A/canada_goose/Iowa/22-040790-001/2022|EPI_ISL_19607832|A_/_H5N1|Original||2.3.4.4b|07.12.2022||||12.12.2024|USDA_APHIS_Veterinary_Services_Diagnostic_Virology_Laboratory_National_Veterinary_Services_Laboratories||HA|4|A/canada_goose/Iowa/22-040790"/>
NNN
</sequence>
<sequence>
<taxon idref="A/canada_goose/Iowa/22-040790-003/2022|EPI_ISL_19607835|A_/_H5N1|Original||2.3.4.4b|07.12.2022||||12.12.2024|USDA_APHIS_Veterinary_Services_Diagnostic_Virology_Laboratory_National_Veterinary_Services_Laboratories||HA|4|A/canada_goose/Iowa/22-040790"/>
NNN
</sequence>
<sequence>
<taxon idref="A/canada_goose/Iowa/22-040793-004/2022|EPI_ISL_19607846|A_/_H5N1|Original||2.3.4.4b|06.12.2022||||12.12.2024|USDA_APHIS_Veterinary_Services_Diagnostic_Virology_Laboratory_National_Veterinary_Services_Laboratories||HA|4|A/canada_goose/Iowa/22-040793"/>
NNN
</sequence>
<sequence>
<taxon idref="A/canada_goose/Minnesota/22-041306-001/2022|EPI_ISL_19607675|A_/_H5N1|Original||2.3.4.4b|19.12.2022||||12.12.2

024|USDA_APHIS_Veterinary_Services_Diagnostic_Virology_Laboratory_National_
Veterinary_Services_Laboratories||HA|4|A/canada_goose/Minnesota"/>
NNN
</sequence>
<sequence>
<taxon idref="A/canada_goose/Nebraska/23-001495-
001/2023|EPI_ISL_19607639|A_/_H5N1|Original||2.3.4.4b|10.01.2023||||12.12.2
024|USDA_APHIS_Veterinary_Services_Diagnostic_Virology_Laboratory_National_
Veterinary_Services_Laboratories||HA|4|A/canada_goose/Nebraska/2"/>
NNN
</sequence>
<sequence>
<taxon idref="A/canada_goose/Nebraska/23-001495-
002/2023|EPI_ISL_19607640|A_/_H5N1|Original||2.3.4.4b|10.01.2023||||12.12.2
024|USDA_APHIS_Veterinary_Services_Diagnostic_Virology_Laboratory_National_
Veterinary_Services_Laboratories||HA|4|A/canada_goose/Nebraska/2"/>
NNN
</sequence>
<sequence>
<taxon idref="A/canada_goose/Wyoming/23-000980-
001/2023|EPI_ISL_19608051|A_/_H5N1|Original||2.3.4.4b|05.01.2023||||12.12.2
024|USDA_APHIS_Veterinary_Services_Diagnostic_Virology_Laboratory_National_
Veterinary_Services_Laboratories||HA|4|A/canada_goose/Wyoming/23-"/>
NNN
</sequence>
<sequence>
<taxon idref="A/canada_goose/Wyoming/23-002120-
001/2023|EPI_ISL_19608054|A_/_H5N1|Original||2.3.4.4b|11.01.2023||||12.12.2
024|USDA_APHIS_Veterinary_Services_Diagnostic_Virology_Laboratory_National_
Veterinary_Services_Laboratories||HA|4|A/canada_goose/Wyoming/23-"/>
NNN
</sequence>
<sequence>
<taxon idref="A/caracara/Coquimbo/SJCEIRR-
2423261/2023|EPI_ISL_18760065|A_/_H5N1|Original||2.3.4.4b|15.03.2023||||14.
01.2024|Emory_University__CEIRR_|Emory_University__CEIRR_|HA|4|A/caracara/C
oquimbo/SJCEIRR-2423261/2023|EPI2913216|DNA_IN"/>
NNN
</sequence>
<sequence>
<taxon idref="A/caspian_tern/Washington/23-024996-
001/2023|EPI_ISL_18737481|A_/_H5N1|Original||2.3.4.4b|07.08.2023|Killian_Ma
ry_Lea|23-024996-
001||09.01.2024|National_Veterinary_Services_Laboratories_-
_USDA|National_Veterinary_Services_Laboratories_-_USDA|HA|4|HA_A"/>
NNN
</sequence>
<sequence>
<taxon idref="A/caspian_tern/Washington/23-025001-
001/2023|EPI_ISL_18737454|A_/_H5N1|Original||2.3.4.4b|11.08.2023|Killian_Ma
ry_Lea|23-025001-
001||09.01.2024|National_Veterinary_Services_Laboratories_-
_USDA|National_Veterinary_Services_Laboratories_-_USDA|HA|4|HA_A"/>
NNN
</sequence>
<sequence>
<taxon idref="A/caspian_tern/Washington/23-025001-
004/2023|EPI_ISL_18737472|A_/_H5N1|Original||2.3.4.4b|11.08.2023|Killian_Ma
ry_Lea|23-025001-
004||09.01.2024|National_Veterinary_Services_Laboratories_-
_USDA|National_Veterinary_Services_Laboratories_-_USDA|HA|4|HA_A"/>

NNN
</sequence>
<sequence>
<taxon idref="A/cat/Nebraska/23-010184-001/2023|EPI_ISL_17821770|A_/_H5N1|Original||2.3.4.4b|30.03.2023|Killian_Mary_Lea||23-010184-001|21.06.2023|Nebraska_Veterinary_Diagnostic_Center|National_Veterinary_Services_Laboratories_-_USDA|HA|4|A/cat/Nebraska/23-010184-0"/>
NNN
</sequence>
<sequence>
<taxon idref="A/chicken/Araucania/239189-2/2023|EPI_ISL_17885958|A_/_H5N1|Original||2.3.4.4b|28.02.2023|GISAID_EpiFlu_Data_Curator|||24.07.2023|CEIRS_Data_Processing_and_Coordinating_Center_Center_for_Research_on_Influenza_Pathogenesis__CRIP_|CEIRS_Data_Processing"/>
NNN
</sequence>
<sequence>
<taxon idref="A/chicken/Araucania/239569-1/2023|EPI_ISL_17885954|A_/_H5N1|Original||2.3.4.4b|02.03.2023|GISAID_EpiFlu_Data_Curator|||24.07.2023|CEIRS_Data_Processing_and_Coordinating_Center_Center_for_Research_on_Influenza_Pathogenesis__CRIP_|CEIRS_Data_Processing"/>
NNN
</sequence>
<sequence>
<taxon idref="A/chicken/Araucania/239569-2/2023|EPI_ISL_17885952|A_/_H5N1|Original||2.3.4.4b|02.03.2023|GISAID_EpiFlu_Data_Curator|||24.07.2023|CEIRS_Data_Processing_and_Coordinating_Center_Center_for_Research_on_Influenza_Pathogenesis__CRIP_|CEIRS_Data_Processing"/>
NNN
</sequence>
<sequence>
<taxon idref="A/chicken/Araucania/240481-1/2023|EPI_ISL_17885949|A_/_H5N1|Original||2.3.4.4b|07.03.2023|GISAID_EpiFlu_Data_Curator|||24.07.2023|CEIRS_Data_Processing_and_Coordinating_Center_Center_for_Research_on_Influenza_Pathogenesis__CRIP_|CEIRS_Data_Processing"/>
NNN
</sequence>
<sequence>
<taxon idref="A/chicken/Araucania/241892-2/2023|EPI_ISL_17885946|A_/_H5N1|Original||2.3.4.4b|14.03.2023|GISAID_EpiFlu_Data_Curator|||24.07.2023|CEIRS_Data_Processing_and_Coordinating_Center_Center_for_Research_on_Influenza_Pathogenesis__CRIP_|CEIRS_Data_Processing"/>
NNN
</sequence>
<sequence>
<taxon idref="A/chicken/Araucania/241914-1/2023|EPI_ISL_17885945|A_/_H5N1|Original||2.3.4.4b|14.03.2023|GISAID_EpiFlu_Data_Curator|||24.07.2023|CEIRS_Data_Processing_and_Coordinating_Center_Center_for_Research_on_Influenza_Pathogenesis__CRIP_|CEIRS_Data_Processing"/>
NNN
</sequence>
<sequence>
<taxon idref="A/chicken/Araucania/244469/2023|EPI_ISL_18760073|A_/_H5N1|Original||2.3.4.4b|25.03.2023||||14.01.2024|Emory_University__CEIRR_|Emory_Uni

versity__CEIRR_|HA|4|A/chicken/Araucania/244469/2023_HA|EPI2913271|DNA_IN"/
>
NNN
</sequence>
<sequence>
<taxon idref="A/chicken/Atacama/235254-
2/2023|EPI_ISL_18760074|A_/_H5N1|Original||2.3.4.4b|04.02.2023||||14.01.202
4|Emory_University__CEIRR_|Emory_University__CEIRR_|HA|4|A/chicken/Atacama/
235254-2/2023_HA|EPI2913279|DNA_IN"/>
NNN
</sequence>
<sequence>
<taxon idref="A/chicken/Aysen/250755-
2/2023|EPI_ISL_19404916|A_/_H5N1|Original||2.3.4.4b|19.04.2023||||13.09.202
4|Emory_University__CEIRR_|Emory_University__CEIRR_|HA|4|A/chicken/Aysen/25
0755-2/2023_HA|EPI3550214|DNA_IN"/>
NNN
</sequence>
<sequence>
<taxon idref="A/chicken/Aysen/SJCEIRR-
2477921/2023|EPI_ISL_18760064|A_/_H5N1|Original||2.3.4.4b|09.04.2023||||14.
01.2024|Emory_University__CEIRR_|Emory_University__CEIRR_|HA|4|A/chicken/Ay
sen/SJCEIRR-2477921/2023|EPI2913211|DNA_IN"/>
NNN
</sequence>
<sequence>
<taxon idref="A/chicken/Aysen/SJCEIRR-
2477922/2023|EPI_ISL_18760068|A_/_H5N1|Original||2.3.4.4b|09.04.2023||||14.
01.2024|Emory_University__CEIRR_|Emory_University__CEIRR_|HA|4|A/chicken/Ay
sen/SJCEIRR-2477922/2023|EPI2913236|DNA_IN"/>
NNN
</sequence>
<sequence>
<taxon idref="A/chicken/Cochabamba/39356/2023|EPI_ISL_19410267|A_/_H5N1|Ori
ginal||2.3.4.4b|26.01.2023||||13.09.2024|Emory_University__CEIRR_||HA|4|A/c
hicken/Cochabamba/39356/2023_HA|EPI3555217|DNA_IN"/>
NNN
</sequence>
<sequence>
<taxon idref="A/chicken/Cochabamba/39383/2023|EPI_ISL_19410268|A_/_H5N1|Ori
ginal||2.3.4.4b|01.02.2023||||13.09.2024|Emory_University__CEIRR_||HA|4|A/c
hicken/Cochabamba/39383/2023_HA|EPI3555224|DNA_IN"/>
NNN
</sequence>
<sequence>
<taxon idref="A/chicken/Cochabamba/39391/2023|EPI_ISL_19410294|A_/_H5N1|Ori
ginal||2.3.4.4b|02.02.2023||||12.09.2024|Emory_University__CEIRR_||HA|4|A/c
hicken/Cochabamba/39391/2023_HA|EPI3555390|DNA_IN"/>
NNN
</sequence>
<sequence>
<taxon idref="A/chicken/Cochabamba/39404/2023|EPI_ISL_19410295|A_/_H5N1|Ori
ginal||2.3.4.4b|03.02.2023||||13.09.2024|Emory_University__CEIRR_||HA|4|A/c
hicken/Cochabamba/39404/2023_HA|EPI3555395|DNA_IN"/>
NNN
</sequence>
<sequence>
<taxon idref="A/chicken/Cochabamba/39425/2023|EPI_ISL_19410269|A_/_H5N1|Ori
ginal||2.3.4.4b|07.02.2023||||12.09.2024|Emory_University__CEIRR_||HA|4|A/c
hicken/Cochabamba/39425/2023_HA|EPI3555231|DNA_IN"/>
NNN

</sequence>
<sequence>
<taxon idref="A/chicken/Cochabamba/39472/2023|EPI_ISL_19410270|A_/_H5N1|Original||2.3.4.4b|17.02.2023||||13.09.2024|Emory_University__CEIRR_||HA|4|A/chicken/Cochabamba/39472/2023_HA|EPI3555237|DNA_IN"/>
NNN
</sequence>
<sequence>
<taxon idref="A/chicken/Cochabamba/39476/2023|EPI_ISL_19410271|A_/_H5N1|Original||2.3.4.4b|18.02.2023||||13.09.2024|Emory_University__CEIRR_||HA|4|A/chicken/Cochabamba/39476/2023_HA|EPI3555242|DNA_IN"/>
NNN
</sequence>
<sequence>
<taxon idref="A/chicken/Cochabamba/39507/2023|EPI_ISL_19410272|A_/_H5N1|Original||2.3.4.4b|01.03.2023||||13.09.2024|Emory_University__CEIRR_||HA|4|A/chicken/Cochabamba/39507/2023_HA|EPI3555249|DNA_IN"/>
NNN
</sequence>
<sequence>
<taxon idref="A/chicken/Cochabamba/39521/2023|EPI_ISL_19410296|A_/_H5N1|Original||2.3.4.4b|03.03.2023||||13.09.2024|Emory_University__CEIRR_||HA|4|A/chicken/Cochabamba/39521/2023_HA|EPI3555401|DNA_IN"/>
NNN
</sequence>
<sequence>
<taxon idref="A/chicken/Cochabamba/39522/2023|EPI_ISL_19410273|A_/_H5N1|Original||2.3.4.4b|03.03.2023||||12.09.2024|Emory_University__CEIRR_||HA|4|A/chicken/Cochabamba/39522/2023_HA|EPI3555255|DNA_IN"/>
NNN
</sequence>
<sequence>
<taxon idref="A/chicken/Cochabamba/39538/2023|EPI_ISL_19410274|A_/_H5N1|Original||2.3.4.4b|03.03.2023||||13.09.2024|Emory_University__CEIRR_||HA|4|A/chicken/Cochabamba/39538/2023_HA|EPI3555259|DNA_IN"/>
NNN
</sequence>
<sequence>
<taxon idref="A/chicken/Cochabamba/39539/2023|EPI_ISL_19418487|A_/_H5N1|Original||2.3.4.4b|03.03.2023||||17.09.2024|Emory_University__CEIRR_||HA|4|A/chicken/Cochabamba/39539/2023_HA|EPI3558264|DNA_IN"/>
NNN
</sequence>
<sequence>
<taxon idref="A/chicken/Cochabamba/39555/2023|EPI_ISL_19410297|A_/_H5N1|Original||2.3.4.4b|09.03.2023||||12.09.2024|Emory_University__CEIRR_||HA|4|A/chicken/Cochabamba/39555/2023_HA|EPI3555406|DNA_IN"/>
NNN
</sequence>
<sequence>
<taxon idref="A/chicken/Cochabamba/39581/2023|EPI_ISL_19410298|A_/_H5N1|Original||2.3.4.4b|13.05.2023||||12.09.2024|Emory_University__CEIRR_||HA|4|A/chicken/Cochabamba/39581/2023_HA|EPI3555412|DNA_IN"/>
NNN
</sequence>
<sequence>
<taxon idref="A/chicken/Maule/247588-1/2023|EPI_ISL_19410280|A_/_H5N1|Original||2.3.4.4b|07.04.2023||||12.09.2024|Emory_University__CEIRR_|Emory_University__CEIRR_|HA|4|A/chicken/Maule/247588-1/2023_HA|EPI3555295|DNA_IN"/>
NNN

</sequence>
<sequence>
<taxon idref="A/chicken/Metropolitana/243148-1/2023|EPI_ISL_18760060|A_/_H5N1|Original||2.3.4.4b|20.03.2023||||14.01.2024|Emory_University__CEIRR_|Emory_University__CEIRR_|HA|4|A/chicken/Metropolitana/243148-1/2023_HA|EPI2913183|DNA_IN"/>
NNN
</sequence>
<sequence>
<taxon idref="A/chicken/Michigan/23-008143-001-original/2023|EPI_ISL_19490436|A_/_H5N1|original||2.3.4.4b|10.03.2023|Killian_Mary_Lea||23-008143-001|18.10.2024|National_Veterinary_Services_Laboratories_-_USDA|National_Veterinary_Services_Laboratories_-_USDA|HA|4|HA"/>
NNN
</sequence>
<sequence>
<taxon idref="A/chicken/Michigan/23-008143-002-original/2023|EPI_ISL_19490438|A_/_H5N1|original||2.3.4.4b|10.03.2023|Killian_Mary_Lea||23-008143-002|18.10.2024|National_Veterinary_Services_Laboratories_-_USDA|National_Veterinary_Services_Laboratories_-_USDA|HA|4|HA"/>
NNN
</sequence>
<sequence>
<taxon idref="A/chicken/Mississippi/23-008358-001-original/2023|EPI_ISL_19490460|A_/_H5N1|original||2.3.4.4b|15.03.2023|Killian_Mary_Lea||23-008358-001|18.10.2024|National_Veterinary_Services_Laboratories_-_USDA|National_Veterinary_Services_Laboratories_-_USDA|HA|4"/>
NNN
</sequence>
<sequence>
<taxon idref="A/chicken/Mississippi/23-008358-002-original/2023|EPI_ISL_19490462|A_/_H5N1|original||2.3.4.4b|15.03.2023|Killian_Mary_Lea||23-008358-002|18.10.2024|National_Veterinary_Services_Laboratories_-_USDA|National_Veterinary_Services_Laboratories_-_USDA|HA|4"/>
NNN
</sequence>
<sequence>
<taxon idref="A/chicken/Nuble/239136/2023|EPI_ISL_17885878|A_/_H5N1|Original||2.3.4.4b|27.02.2023|GISAID_EpiFlu_Data_Curator|||19.07.2023|CEIRS_Data_Processing_and_Coordinating_Center_Center_for_Research_on_Influenza_Pathogenesis__CRIP_|CEIRS_Data_Processing_and_C"/>
NNN
</sequence>
<sequence>
<taxon idref="A/chicken/Nuble/240155/2023|EPI_ISL_17885877|A_/_H5N1|Original||2.3.4.4b|06.03.2023|GISAID_EpiFlu_Data_Curator|||29.06.2023|CEIRS_Data_Processing_and_Coordinating_Center_Center_for_Research_on_Influenza_Pathogenesis__CRIP_|CEIRS_Data_Processing_and_C"/>
NNN
</sequence>
<sequence>
<taxon idref="A/chicken/Nuble/240684/2023|EPI_ISL_17885876|A_/_H5N1|Original||2.3.4.4b|08.03.2023|GISAID_EpiFlu_Data_Curator|||19.07.2023|CEIRS_Data_Processing_and_Coordinating_Center_Center_for_Research_on_Influenza_Pathogenesis__CRIP_|CEIRS_Data_Processing_and_C"/>
NNN
</sequence>

<sequence>
<taxon idref="A/chicken/Nuble/241557-
1/2023|EPI_ISL_17885874|A_/_H5N1|Original||2.3.4.4b|10.03.2023|GISAID_EpiFl
u_Data_Curator|||19.07.2023|CEIRS_Data_Processing_and_Coordinating_Center_C
enter_for_Research_on_Influenza_Pathogenesis__CRIP_|CEIRS_Data_Processing_a
nd"/>
NNN
</sequence>
<sequence>
<taxon idref="A/chicken/Nuble/241681-
1/2023|EPI_ISL_17885873|A_/_H5N1|Original||2.3.4.4b|10.03.2023|GISAID_EpiFl
u_Data_Curator|||19.07.2023|CEIRS_Data_Processing_and_Coordinating_Center_C
enter_for_Research_on_Influenza_Pathogenesis__CRIP_|CEIRS_Data_Processing_a
nd"/>
NNN
</sequence>
<sequence>
<taxon idref="A/chicken/OHiggins/241252-
1/2023|EPI_ISL_17885872|A_/_H5N1|Original||2.3.4.4b|12.03.2023|GISAID_EpiFl
u_Data_Curator|||19.07.2023|CEIRS_Data_Processing_and_Coordinating_Center_C
enter_for_Research_on_Influenza_Pathogenesis__CRIP_|CEIRS_Data_Processing_"
/>
NNN
</sequence>
<sequence>
<taxon idref="A/chicken/OHiggins/241252-
3/2023|EPI_ISL_17885871|A_/_H5N1|Original||2.3.4.4b|12.03.2023|GISAID_EpiFl
u_Data_Curator|||19.07.2023|CEIRS_Data_Processing_and_Coordinating_Center_C
enter_for_Research_on_Influenza_Pathogenesis__CRIP_|CEIRS_Data_Processing_"
/>
NNN
</sequence>
<sequence>
<taxon idref="A/chicken/OHiggins/241252-
6/2023|EPI_ISL_17885870|A_/_H5N1|Original||2.3.4.4b|12.03.2023|GISAID_EpiFl
u_Data_Curator|||19.07.2023|CEIRS_Data_Processing_and_Coordinating_Center_C
enter_for_Research_on_Influenza_Pathogenesis__CRIP_|CEIRS_Data_Processing_"
/>
NNN
</sequence>
<sequence>
<taxon idref="A/chicken/OHiggins/242581-
2/2023|EPI_ISL_18690682|A_/_H5N1|Original||2.3.4.4b|16.03.2023||||26.12.202
3|Emory_University__CEIRR_|Emory_University__CEIRR_|HA|4|A/chicken/OHiggins
/242581-2/2023_HA|EPI2868174|DNA_IN"/>
NNN
</sequence>
<sequence>
<taxon idref="A/chicken/Oregon/23-009029-001-
original/2023|EPI_ISL_19490488|A_/_H5N1|original||2.3.4.4b|15.03.2023|Killi
an_Mary_Lea||23-009029-
001|18.10.2024|National_Veterinary_Services_Laboratories_-
_USDA|National_Veterinary_Services_Laboratories_-_USDA|HA|4|HA_A"/>
NNN
</sequence>
<sequence>
<taxon idref="A/chicken/Panama/23-004630-
004/2023|EPI_ISL_17660068|A_/_H5N1|Original||2.3.4.4b|03.02.2023|Killian_Ma
ry_Lea|LEA-39-
23_M4|324|15.05.2023|El_Laboratorio_de_Diagnostico_de_Enfermedades_Vesicula
res__LADIVES_|National_Veterinary_Services_Laboratories_-_US"/>

NNN
</sequence>
<sequence>
<taxon idref="A/chicken/Panama/23-004630-
005/2023|EPI_ISL_17660069|A_/_H5N1|Original||2.3.4.4b|03.02.2023|Killian_Ma
ry_Lea|LEA-40-
23_M5|324|15.05.2023|El_Laboratorio_de_Diagnostico_de_Enfermedades_Vesicula
res__LADIVES_|National_Veterinary_Services_Laboratories_-_US"/>
NNN
</sequence>
<sequence>
<taxon idref="A/chicken/Peru/AIS0542/2022|EPI_ISL_17805989|A_/_H5N1|Origina
l||2.3.4.4b|01.12.2022|GISAID_EpiFlu_Data_Curator|||15.06.2023|||HA|4|A/chi
cken/Peru/AIS0542/2022_HA|EPI2597168|DNA_IN"/>
NNN
</sequence>
<sequence>
<taxon idref="A/chicken/Peru/AIS0543/2022|EPI_ISL_17805990|A_/_H5N1|Origina
l||2.3.4.4b|01.12.2022|GISAID_EpiFlu_Data_Curator|||15.06.2023|||HA|4|A/chi
cken/Peru/AIS0543/2022_HA|EPI2597176|DNA_IN"/>
NNN
</sequence>
<sequence>
<taxon idref="A/chicken/Peru/AIS0544/2022|EPI_ISL_17805991|A_/_H5N1|Origina
l||2.3.4.4b|03.12.2022|GISAID_EpiFlu_Data_Curator|||15.06.2023|||HA|4|A/chi
cken/Peru/AIS0544/2022_HA|EPI2597184|DNA_IN"/>
NNN
</sequence>
<sequence>
<taxon idref="A/chicken/Peru/AIS0545/2022|EPI_ISL_17805992|A_/_H5N1|Origina
l||2.3.4.4b|03.12.2022|GISAID_EpiFlu_Data_Curator|||15.06.2023|||HA|4|A/chi
cken/Peru/AIS0545/2022_HA|EPI2597191|DNA_IN"/>
NNN
</sequence>
<sequence>
<taxon idref="A/chicken/Peru/AIS0546/2022|EPI_ISL_17805986|A_/_H5N1|Origina
l||2.3.4.4b|18.12.2022|GISAID_EpiFlu_Data_Curator|||15.06.2023|||HA|4|A/chi
cken/Peru/AIS0546/2022_HA|EPI2597144|DNA_IN"/>
NNN
</sequence>
<sequence>
<taxon idref="A/chicken/Peru/AIS0547/2022|EPI_ISL_17805993|A_/_H5N1|Origina
l||2.3.4.4b|28.12.2022|GISAID_EpiFlu_Data_Curator|||15.06.2023|||HA|4|A/chi
cken/Peru/AIS0547/2022_HA|EPI2597196|DNA_IN"/>
NNN
</sequence>
<sequence>
<taxon idref="A/chicken/Peru/AIS0548/2022|EPI_ISL_17805994|A_/_H5N1|Origina
l||2.3.4.4b|22.12.2022|GISAID_EpiFlu_Data_Curator|||15.06.2023|||HA|4|A/chi
cken/Peru/AIS0548/2022_HA|EPI2597204|DNA_IN"/>
NNN
</sequence>
<sequence>
<taxon idref="A/chicken/Peru/AIS0549/2022|EPI_ISL_17805996|A_/_H5N1|Origina
l||2.3.4.4b|22.12.2022|GISAID_EpiFlu_Data_Curator|||15.06.2023|||HA|4|A/chi
cken/Peru/AIS0549/2022_HA|EPI2597220|DNA_IN"/>
NNN
</sequence>
<sequence>

<taxon idref="A/chicken/Peru/AIS0550/2022|EPI_ISL_17805995|A_/_H5N1|Origina
l||2.3.4.4b|12.12.2022|GISAID_EpiFlu_Data_Curator|||15.06.2023|||HA|4|A/chi
cken/Peru/AIS0550/2022_HA|EPI2597212|DNA_IN"/>
NNN
</sequence>
<sequence>
<taxon idref="A/chicken/Peru/AIS0551/2022|EPI_ISL_17806001|A_/_H5N1|Origina
l||2.3.4.4b|12.12.2022|GISAID_EpiFlu_Data_Curator|||15.06.2023|||HA|4|A/chi
cken/Peru/AIS0551/2022_HA|EPI2597260|DNA_IN"/>
NNN
</sequence>
<sequence>
<taxon idref="A/chicken/Potosi/39870/2023|EPI_ISL_19410299|A_/_H5N1|Origina
l||2.3.4.4b|06.06.2023||||12.09.2024|Emory_University__CEIRR_||HA|4|A/chick
en/Potosi/39870/2023_HA|EPI3555418|DNA_IN"/>
NNN
</sequence>
<sequence>
<taxon idref="A/chungungo/Chile/241436/2023|EPI_ISL_19391459|A_/_H5N1|Origi
nal||2.3.4.4b|10.03.2023||||09.09.2024|CEIRS_Data_Processing_and_Coordinati
ng_Center_Center_for_Research_on_Influenza_Pathogenesis__CRIP_||HA|4|A/chun
gungo/Chile/241436/2023_HA|EPI3549620|DN"/>
NNN
</sequence>
<sequence>
<taxon idref="A/common_raven/Alaska/22-042128-
001/2022|EPI_ISL_19158196|A_/_H5N1|Original||2.3.4.4b|18.12.2022||||22.05.2
024|USDA_APHIS_Veterinary_Services_Diagnostic_Virology_Laboratory_National_
Veterinary_Services_Laboratories||HA|4|A/common_raven/Alasca/22-04"/>
NNN
</sequence>
<sequence>
<taxon idref="A/coopers_hawk/Colorado/22-042170-
001/2022|EPI_ISL_19608100|A_/_H5N1|Original||2.3.4.4b|25.12.2022||||12.12.2
024|USDA_APHIS_Veterinary_Services_Diagnostic_Virology_Laboratory_National_
Veterinary_Services_Laboratories||HA|4|A/coopers_hawk/Colorado/2"/>
NNN
</sequence>
<sequence>
<taxon idref="A/coopers_hawk/Washington/22-041088-
001/2022|EPI_ISL_19608106|A_/_H5N1|Original||2.3.4.4b|14.12.2022||||12.12.2
024|USDA_APHIS_Veterinary_Services_Diagnostic_Virology_Laboratory_National_
Veterinary_Services_Laboratories||HA|4|A/coopers_hawk/Washingt"/>
NNN
</sequence>
<sequence>
<taxon idref="A/crow/Colorado/22-040765-
001/2022|EPI_ISL_19608110|A_/_H5N1|Original||2.3.4.4b|12.12.2022||||12.12.2
024|USDA_APHIS_Veterinary_Services_Diagnostic_Virology_Laboratory_National_
Veterinary_Services_Laboratories||HA|4|A/crow/Colorado/22-040765-001/202"/>
NNN
</sequence>
<sequence>
<taxon idref="A/dolphin/Maule/SJCEIRR-
246026/2023|EPI_ISL_18777139|A_/_H5N1|Original||2.3.4.4b|31.03.2023||||16.0
1.2024|Emory_University__CEIRR_|Emory_University__CEIRR_|HA|4|A/dolphin/Mau
le/SJCEIRR-246026/2023|EPI2921521|DNA_IN"/>
NNN
</sequence>
<sequence>

<taxon idref="A/dolphin/Nuble/SJCEIRR-2482441/2023|EPI_ISL_18777138|A_/_H5N1|Original||2.3.4.4b|01.04.2023||||16.01.2024|Emory_University__CEIRR_|Emory_University__CEIRR_|HA|4|A/dolphin/Nuble/SJCEIRR-2482441/2023|EPI2921516|DNA_IN"/>
NNN
</sequence>
<sequence>
<taxon idref="A/duck/Araucania/239189-3/2023|EPI_ISL_17885963|A_/_H5N1|Original||2.3.4.4b|28.02.2023|GISAID_EpiFlu_Data_Curator|||29.06.2023|||HA|4|A/duck/Araucania/239189-3/2023_HA|EPI2609467|DNA_IN"/>
NNN
</sequence>
<sequence>
<taxon idref="A/duck/Araucania/240481-2/2023|EPI_ISL_17885961|A_/_H5N1|Original||2.3.4.4b|07.03.2023|GISAID_EpiFlu_Data_Curator|||29.06.2023|||HA|4|A/duck/Araucania/240481-2/2023_HA|EPI2609458|DNA_IN"/>
NNN
</sequence>
<sequence>
<taxon idref="A/duck/Araucania/241914-2/2023|EPI_ISL_17885959|A_/_H5N1|Original||2.3.4.4b|14.03.2023|GISAID_EpiFlu_Data_Curator|||29.06.2023|||HA|4|A/duck/Araucania/241914-2/2023_HA|EPI2609449|DNA_IN"/>
NNN
</sequence>
<sequence>
<taxon idref="A/duck/Maule/240466-1/2023|EPI_ISL_17885957|A_/_H5N1|Original||2.3.4.4b|07.03.2023|GISAID_EpiFlu_Data_Curator|||29.06.2023|||HA|4|A/duck/Maule/240466-1/2023_HA|EPI2609439|DNA_IN"/>
NNN
</sequence>
<sequence>
<taxon idref="A/duck/Peru/CAL-INS-013/2023|EPI_ISL_18217104|A_/_H5N1|Original||2.3.4.4b|24.07.2023|Padilla_Rojas_Carlos_Patricio|||04.09.2023|Servicio_Nacional_Forestal_y_de_Fauna_Silvestre__SERFOR_|Instituto_Nacional_de_Salud_Peru|HA|4|A/duck/Peru/CAL-INS-013/2023"/>
NNN
</sequence>
<sequence>
<taxon idref="A/duck/Peru/LAM-INS-014/2023|EPI_ISL_18497946|A_/_H5N1|Original||2.3.4.4b|26.09.2023|Padilla_Rojas_Carlos_Patricio|||13.11.2023|Servicio_Nacional_de_Sanidad_Agraria_del_Peru_-_SENASA|Instituto_Nacional_de_Salud_Peru|HA|4|A/duck/Peru/LAM-INS-014/2023|E"/>
NNN
</sequence>
<sequence>
<taxon idref="A/duck/Peru/LAM-INS-015/2023|EPI_ISL_18497950|A_/_H5N1|Original||2.3.4.4b|20.09.2023|Padilla_Rojas_Carlos_Patricio|||13.11.2023|Servicio_Nacional_de_Sanidad_Agraria_del_Peru_-_SENASA|Instituto_Nacional_de_Salud_Peru|HA|4|A/duck/Peru/LAM-INS-015/2023|E"/>
NNN
</sequence>
<sequence>
<taxon idref="A/dunlin/BC/AIVPHL-335/2022|EPI_ISL_17051470|A_/_H5N1|Original||2.3.4.4b|13.12.2022|Russell_Sh

annon_Laurel|||28.02.2023|B.C._Centre_for_Disease_Control|British_Columbia_Centre_for_Disease_Control|HA|4|A/dunlin/BC/AIVPHL-335/2022_HA|EPI2429201|DNA_INS"/>
NNN
</sequence>
<sequence>
<taxon idref="A/dunlin/Washington/22-040394-001/2022|EPI_ISL_19607890|A_/_H5N1|Original||2.3.4.4b|03.12.2022||||12.12.2024|USDA_APHIS_Veterinary_Services_Diagnostic_Virology_Laboratory_National_Veterinary_Services_Laboratories||HA|4|A/dunlin/Washington/22-040394"/>
NNN
</sequence>
<sequence>
<taxon idref="A/eared_grebe/California/23-000276-003/2022|EPI_ISL_19607866|A_/_H5N1|Original||2.3.4.4b|15.12.2022||||12.12.2024|USDA_APHIS_Veterinary_Services_Diagnostic_Virology_Laboratory_National_Veterinary_Services_Laboratories||HA|4|A/eared_grebe/California"/>
NNN
</sequence>
<sequence>
<taxon idref="A/eared_grebe/California/23-000276-004/2022|EPI_ISL_19607867|A_/_H5N1|Original||2.3.4.4b|15.12.2022||||12.12.2024|USDA_APHIS_Veterinary_Services_Diagnostic_Virology_Laboratory_National_Veterinary_Services_Laboratories||HA|4|A/eared_grebe/California"/>
NNN
</sequence>
<sequence>
<taxon idref="A/environment/Chile/C62044/2022|EPI_ISL_19167924|A_/_H5N1|Original||2.3.4.4b|13.12.2022||||28.05.2024|St._Jude_Center_of_Excellence_for_Influenza_Research_and_Response_-_CEIRR_Influenza_Data_Processing_and_Communication_Center||HA|4|A/environment/Chile"/>
NNN
</sequence>
<sequence>
<taxon idref="A/falcon/Costa_Rica/INC-0001-D0377-23/2023|EPI_ISL_16958548|A_/_H5N1|Original||2.3.4.4b|26.01.2023|Soto-Garita_Claudio|377-23|783955|07.07.2023|Servicio_Nacional_de_Salud_Animal__SENASA_|Inciensa_Investigacion_y_Ensenanza_en_Nutricion_y_Salud|HA|4|A/"/>
NNN
</sequence>
<sequence>
<taxon idref="A/frigatebird/Rio_de_Janeiro/MAPA-1532N/2023|EPI_ISL_18755251|A_/_H5N1|Original||2.3.4.4b|03.06.2023||||12.01.2024|Ministerio_da_Agricultura_e_Pecuaria|Ministerio_da_Agricultura_e_Pecuaria|HA|4|A/frigatebird/Rio_de_Janeiro/MAPA-1532N/2023_HA|EPI2908258"/>
NNN
</sequence>
<sequence>
<taxon idref="A/gadwall/Alaska/22-040806-001/2022|EPI_ISL_19607884|A_/_H5N1|Original||2.3.4.4b|12.12.2022||||12.12.2024|USDA_APHIS_Veterinary_Services_Diagnostic_Virology_Laboratory_National_Veterinary_Services_Laboratories||HA|4|A/gadwall/Alaska/22-040806-001/2"/>
NNN
</sequence>
<sequence>
<taxon idref="A/gadwall/Alaska/22-041762-001/2022|EPI_ISL_19607925|A_/_H5N1|Original||2.3.4.4b|10.12.2022||||12.12.2024|USDA_APHIS_Veterinary_Services_Diagnostic_Virology_Laboratory_National_Veterinary_Services_Laboratories||HA|4|A/gadwall/Alaska/22-041762-001/2"/>

NNN
</sequence>
<sequence>
<taxon idref="A/gadwall/Alaska/22-041762-
004/2022|EPI_ISL_19607930|A_/_H5N1|Original||2.3.4.4b|10.12.2022||||12.12.2
024|USDA_APHIS_Veterinary_Services_Diagnostic_Virology_Laboratory_National_
Veterinary_Services_Laboratories||HA|4|A/gadwall/Alaska/22-041762-004/2"/>
NNN
</sequence>
<sequence>
<taxon idref="A/gadwall/Alaska/22-041762-
005/2022|EPI_ISL_19607980|A_/_H5N1|Original||2.3.4.4b|10.12.2022||||12.12.2
024|USDA_APHIS_Veterinary_Services_Diagnostic_Virology_Laboratory_National_
Veterinary_Services_Laboratories||HA|4|A/gadwall/Alaska/22-041762-005/2"/>
NNN
</sequence>
<sequence>
<taxon idref="A/gadwall/Alaska/22-041762-
006/2022|EPI_ISL_19607982|A_/_H5N1|Original||2.3.4.4b|10.12.2022||||12.12.2
024|USDA_APHIS_Veterinary_Services_Diagnostic_Virology_Laboratory_National_
Veterinary_Services_Laboratories||HA|4|A/gadwall/Alaska/22-041762-006/2"/>
NNN
</sequence>
<sequence>
<taxon idref="A/gadwall/Alaska/23-002626-
001/2023|EPI_ISL_19607984|A_/_H5N1|Original||2.3.4.4b|17.01.2023||||12.12.2
024|USDA_APHIS_Veterinary_Services_Diagnostic_Virology_Laboratory_National_
Veterinary_Services_Laboratories||HA|4|A/gadwall/Alaska/23-002626-001/2"/>
NNN
</sequence>
<sequence>
<taxon idref="A/gadwall/Oregon/23-001494-
006/2023|EPI_ISL_19608014|A_/_H5N1|Original||2.3.4.4b|05.01.2023||||12.12.2
024|USDA_APHIS_Veterinary_Services_Diagnostic_Virology_Laboratory_National_
Veterinary_Services_Laboratories||HA|4|A/gadwall/Oregon/23-001494-006/2"/>
NNN
</sequence>
<sequence>
<taxon idref="A/gadwall/South_Carolina/22-042019-
001/2022|EPI_ISL_19608016|A_/_H5N1|Original||2.3.4.4b|15.12.2022||||12.12.2
024|USDA_APHIS_Veterinary_Services_Diagnostic_Virology_Laboratory_National_
Veterinary_Services_Laboratories||HA|4|A/gadwall/South_Carolina"/>
NNN
</sequence>
<sequence>
<taxon idref="A/gadwall/Texas/23-003062-
010/2023|EPI_ISL_19607945|A_/_H5N1|Original||2.3.4.4b|18.01.2023||||12.12.2
024|USDA_APHIS_Veterinary_Services_Diagnostic_Virology_Laboratory_National_
Veterinary_Services_Laboratories||HA|4|A/gadwall/Texas/23-003062-010/202"/>
NNN
</sequence>
<sequence>
<taxon idref="A/gannet/Florida/23-002762-
022/2022|EPI_ISL_19607954|A_/_H5N1|Original||2.3.4.4b|30.12.2022||||12.12.2
024|USDA_APHIS_Veterinary_Services_Diagnostic_Virology_Laboratory_National_
Veterinary_Services_Laboratories||HA|4|A/gannet/Florida/23-002762-022/2"/>
NNN
</sequence>
<sequence>
<taxon idref="A/glaucous_gull/Washington/23-025001-
002/2023|EPI_ISL_18737455|A_/_H5N1|Original||2.3.4.4b|11.08.2023|Killian_Ma

ry_Lea|23-025001-
002||12.12.2024|National_Veterinary_Services_Laboratories_-
_USDA|National_Veterinary_Services_Laboratories_-_USDA|HA|4|HA_"/>
NNN
</sequence>
<sequence>
<taxon idref="A/glaucous_gull/Washington/23-025001-
003/2023|EPI_ISL_18737463|A_/_H5N1|Original||2.3.4.4b|11.08.2023|Killian_Ma
ry_Lea|23-025001-
003||12.12.2024|National_Veterinary_Services_Laboratories_-
_USDA|National_Veterinary_Services_Laboratories_-_USDA|HA|4|HA_"/>
NNN
</sequence>
<sequence>
<taxon idref="A/goose/Araucania/239189-
1/2023|EPI_ISL_17885869|A_/_H5N1|Original||2.3.4.4b|28.02.2023|GISAID_EpiFl
u_Data_Curator|||24.07.2023|CEIRS_Data_Processing_and_Coordinating_Center_C
enter_for_Research_on_Influenza_Pathogenesis__CRIP_|CEIRS_Data_Processing_a
"/>
NNN
</sequence>
<sequence>
<taxon idref="A/goose/Argentina/SENASA-
140223/2023|EPI_ISL_17950994|A_/_H5N1|Original||2.3.4.4b|11.02.2023|GISAID_
EpiFlu_Data_Curator|||30.06.2023|||HA|4|A/goose/Argentina/SENASA-
140223/2023_HA|EPI2610769|DNA_IN"/>
NNN
</sequence>
<sequence>
<taxon idref="A/goose/California/23-001053-
002/2022|EPI_ISL_19605288|A_/_H5N1|Original||2.3.4.4b|17.12.2022||||12.12.2
024|USDA_APHIS_Veterinary_Services_Diagnostic_Virology_Laboratory_National_
Veterinary_Services_Laboratories||HA|4|A/goose/California/23-001053-0"/>
NNN
</sequence>
<sequence>
<taxon idref="A/goose/Kansas/W22-
1154/2022|EPI_ISL_17373141|A_/_H5N1|||2.3.4.4b|05.12.2022||||01.04.2023|St.
_Jude_Center_of_Excellence_for_Influenza_Research_and_Response_-
_CEIRR_Influenza_Data_Processing_and_Communication_Center|St._Jude_Center_o
f_Excellence_for_In"/>
NNN
</sequence>
<sequence>
<taxon idref="A/goose/Louisiana/23-000409-
006/2022|EPI_ISL_19608587|A_/_H5N1|Original||2.3.4.4b|02.12.2022||||12.12.2
024|USDA_APHIS_Veterinary_Services_Diagnostic_Virology_Laboratory_National_
Veterinary_Services_Laboratories||HA|4|A/goose/Louisiana/23-000409-006"/>
NNN
</sequence>
<sequence>
<taxon idref="A/goose/Louisiana/W22-
1163/2022|EPI_ISL_17373068|A_/_H5N1|||2.3.4.4b|05.12.2022||||01.04.2023|St.
_Jude_Center_of_Excellence_for_Influenza_Research_and_Response_-
_CEIRR_Influenza_Data_Processing_and_Communication_Center|St._Jude_Center_o
f_Excellence_for"/>
NNN
</sequence>
<sequence>
<taxon idref="A/goose/Utah/23-001040-
001/2023|EPI_ISL_19605253|A_/_H5N1|Original||2.3.4.4b|04.01.2023||||12.12.2

024|USDA_APHIS_Veterinary_Services_Diagnostic_Virology_Laboratory_National_Veterinary_Services_Laboratories||HA|4|A/goose/Utah/23-001040-001/2023_HA|E"/>
NNN
</sequence>
<sequence>
<taxon idref="A/goose/Utah/23-001041-001/2023|EPI_ISL_19605254|A_/_H5N1|Original||2.3.4.4b|06.01.2023||||12.12.2024|USDA_APHIS_Veterinary_Services_Diagnostic_Virology_Laboratory_National_Veterinary_Services_Laboratories||HA|4|A/goose/Utah/23-001041-001/2023_HA|E"/>
NNN
</sequence>
<sequence>
<taxon idref="A/goose/Utah/23-001041-003/2023|EPI_ISL_19605257|A_/_H5N1|Original||2.3.4.4b|06.01.2023||||12.12.2024|USDA_APHIS_Veterinary_Services_Diagnostic_Virology_Laboratory_National_Veterinary_Services_Laboratories||HA|4|A/goose/Utah/23-001041-003/2023_HA|E"/>
NNN
</sequence>
<sequence>
<taxon idref="A/goose/Utah/23-001041-007/2023|EPI_ISL_19605268|A_/_H5N1|Original||2.3.4.4b|06.01.2023||||12.12.2024|USDA_APHIS_Veterinary_Services_Diagnostic_Virology_Laboratory_National_Veterinary_Services_Laboratories||HA|4|A/goose/Utah/23-001041-007/2023_HA|E"/>
NNN
</sequence>
<sequence>
<taxon idref="A/goose/Utah/23-001041-009/2023|EPI_ISL_19605259|A_/_H5N1|Original||2.3.4.4b|06.01.2023||||12.12.2024|USDA_APHIS_Veterinary_Services_Diagnostic_Virology_Laboratory_National_Veterinary_Services_Laboratories||HA|4|A/goose/Utah/23-001041-009/2023_HA|E"/>
NNN
</sequence>
<sequence>
<taxon idref="A/gray_gull/Arica/239053-1/2023|EPI_ISL_19410281|A_/_H5N1|Original||2.3.4.4b|23.02.2023||||12.09.2024|Emory_University__CEIRR_|Emory_University__CEIRR_|HA|4|A/gray_gull/Arica/239053-1/2023_HA|EPI3555302|DNA_IN"/>
NNN
</sequence>
<sequence>
<taxon idref="A/gray_gull/Arica_y_Parinacota/235908-1/2023|EPI_ISL_19404913|A_/_H5N1|Original||2.3.4.4b|07.02.2023||||10.09.2024|Emory_University__CEIRR_|Emory_University__CEIRR_|HA|4|A/gray_gull/Arica_y_Parinacota/235908-1/2023_HA|EPI3550208|DNA_IN"/>
NNN
</sequence>
<sequence>
<taxon idref="A/gray_gull/Chile/C61947/2022|EPI_ISL_16891400|A_/_H5N1|||2.3.4.4b|02.12.2022||||04.02.2023|||HA|4|A/gray_gull/Chile/C61947/2022|EPI2397300|DNA_IN"/>
NNN
</sequence>
<sequence>
<taxon idref="A/great-tailed_grackle/Kansas/22-041130-005/2022|EPI_ISL_19605261|A_/_H5N1|Original||2.3.4.4b|13.12.2022||||12.12.2

024|USDA_APHIS_Veterinary_Services_Diagnostic_Virology_Laboratory_National_Veterinary_Services_Laboratories||HA|4|A/great-tailed_grac"/>
NNN
</sequence>
<sequence>
<taxon idref="A/great-tailed_grackle/Kansas/22-041130-006/2022|EPI_ISL_19605262|A_/_H5N1|Original||2.3.4.4b|13.12.2022||||12.12.2024|USDA_APHIS_Veterinary_Services_Diagnostic_Virology_Laboratory_National_Veterinary_Services_Laboratories||HA|4|A/great-tailed_grac"/>
NNN
</sequence>
<sequence>
<taxon idref="A/great-tailed_grackle/Kansas/W22-1223B/2022|EPI_ISL_17471582|A_/_H5N1|||2.3.4.4b|13.12.2022||||09.04.2023|St._Jude_Center_of_Excellence_for_Influenza_Research_and_Response_-_CEIRR_Influenza_Data_Processing_and_Communication_Center|St._Jude_Center_of_E"/>
NNN
</sequence>
<sequence>
<taxon idref="A/great-tailed_grackle/Kansas/W22-1223C/2022|EPI_ISL_17471577|A_/_H5N1|||2.3.4.4b|13.12.2022||||09.04.2023|St._Jude_Center_of_Excellence_for_Influenza_Research_and_Response_-_CEIRR_Influenza_Data_Processing_and_Communication_Center|St._Jude_Center_of_E"/>
NNN
</sequence>
<sequence>
<taxon idref="A/great_horned_owl/BC/AIVPHL-353/2023|EPI_ISL_17051479|A_/_H5N1|Original||2.3.4.4b|09.01.2023|Russell_Shannon_Laurel|||28.02.2023|B.C._Centre_for_Disease_Control|British_Columbia_Centre_for_Disease_Control|HA|4|A/great_horned_owl/BC/AIVPHL-353/2023_HA"/>
NNN
</sequence>
<sequence>
<taxon idref="A/great_horned_owl/BC/AIVPHL-372/2023|EPI_ISL_17051490|A_/_H5N1|Original||2.3.4.4b|16.01.2023|Russell_Shannon_Laurel|||28.02.2023|B.C._Centre_for_Disease_Control|British_Columbia_Centre_for_Disease_Control|HA|4|A/great_horned_owl/BC/AIVPHL-372/2023_HA"/>
NNN
</sequence>
<sequence>
<taxon idref="A/great_horned_owl/California/23-000277-003/2022|EPI_ISL_19607321|A_/_H5N1|Original||2.3.4.4b|14.12.2022||||12.12.2024|USDA_APHIS_Veterinary_Services_Diagnostic_Virology_Laboratory_National_Veterinary_Services_Laboratories||HA|4|A/great_horned_owl/"/>
NNN
</sequence>
<sequence>
<taxon idref="A/great_horned_owl/California/23-001056-001/2022|EPI_ISL_19607323|A_/_H5N1|Original||2.3.4.4b|21.12.2022||||12.12.2024|USDA_APHIS_Veterinary_Services_Diagnostic_Virology_Laboratory_National_Veterinary_Services_Laboratories||HA|4|A/great_horned_owl/"/>
NNN
</sequence>
<sequence>
<taxon idref="A/great_horned_owl/California/23-001856-003/2022|EPI_ISL_19607324|A_/_H5N1|Original||2.3.4.4b|19.12.2022||||12.12.2024|USDA_APHIS_Veterinary_Services_Diagnostic_Virology_Laboratory_National_Veterinary_Services_Laboratories||HA|4|A/great_horned_owl/"/>
NNN

</sequence>
<sequence>
<taxon idref="A/great_horned_owl/California/23-006649-
001/2023|EPI_ISL_17964879|A_/_H5N1|Original||2.3.4.4b|23.02.2023|Killian_Ma
ry_Lea||23-006649-
001|06.07.2023|National_Veterinary_Services_Laboratories_-
_USDA|National_Veterinary_Services_Laboratories_-_USDA|HA|4|"/>
NNN
</sequence>
<sequence>
<taxon idref="A/great_horned_owl/Colorado/22-039952-
001/2022|EPI_ISL_19607330|A_/_H5N1|Original||2.3.4.4b|05.12.2022||||12.12.2
024|USDA_APHIS_Veterinary_Services_Diagnostic_Virology_Laboratory_National_
Veterinary_Services_Laboratories||HA|4|A/great_horned_owl/Co"/>
NNN
</sequence>
<sequence>
<taxon idref="A/great_horned_owl/Colorado/22-040764-
001/2022|EPI_ISL_19607333|A_/_H5N1|Original||2.3.4.4b|09.12.2022||||12.12.2
024|USDA_APHIS_Veterinary_Services_Diagnostic_Virology_Laboratory_National_
Veterinary_Services_Laboratories||HA|4|A/great_horned_owl/Co"/>
NNN
</sequence>
<sequence>
<taxon idref="A/great_horned_owl/Colorado/23-001467-
002/2023|EPI_ISL_19607365|A_/_H5N1|Original||2.3.4.4b|07.01.2023||||12.12.2
024|USDA_APHIS_Veterinary_Services_Diagnostic_Virology_Laboratory_National_
Veterinary_Services_Laboratories||HA|4|A/great_horned_owl/Co"/>
NNN
</sequence>
<sequence>
<taxon idref="A/great_horned_owl/Colorado/23-001470-
001/2023|EPI_ISL_19607367|A_/_H5N1|Original||2.3.4.4b|05.01.2023||||12.12.2
024|USDA_APHIS_Veterinary_Services_Diagnostic_Virology_Laboratory_National_
Veterinary_Services_Laboratories||HA|4|A/great_horned_owl/Co"/>
NNN
</sequence>
<sequence>
<taxon idref="A/great_horned_owl/Colorado/23-002236-
003/2023|EPI_ISL_19607368|A_/_H5N1|Original||2.3.4.4b|11.01.2023||||12.12.2
024|USDA_APHIS_Veterinary_Services_Diagnostic_Virology_Laboratory_National_
Veterinary_Services_Laboratories||HA|4|A/great_horned_owl/Co"/>
NNN
</sequence>
<sequence>
<taxon idref="A/great_horned_owl/Colorado/23-002760-
001/2023|EPI_ISL_19607395|A_/_H5N1|Original||2.3.4.4b|16.01.2023||||12.12.2
024|USDA_APHIS_Veterinary_Services_Diagnostic_Virology_Laboratory_National_
Veterinary_Services_Laboratories||HA|4|A/great_horned_owl/Co"/>
NNN
</sequence>
<sequence>
<taxon idref="A/great_horned_owl/Colorado/23-002760-
002/2023|EPI_ISL_19607396|A_/_H5N1|Original||2.3.4.4b|16.01.2023||||12.12.2
024|USDA_APHIS_Veterinary_Services_Diagnostic_Virology_Laboratory_National_
Veterinary_Services_Laboratories||HA|4|A/great_horned_owl/Co"/>
NNN
</sequence>
<sequence>
<taxon idref="A/great_horned_owl/Colorado/23-005619-
001/2023|EPI_ISL_17964860|A_/_H5N1|Original||2.3.4.4b|14.02.2023|Killian_Ma

ry_Lea||23-005619-
001|06.07.2023|National_Veterinary_Services_Laboratories_-
_USDA|National_Veterinary_Services_Laboratories_-_USDA|HA|4|HA"/>
NNN
</sequence>
<sequence>
<taxon idref="A/great_horned_owl/Colorado/23-005620-
001/2023|EPI_ISL_17964861|A_/_H5N1|Original||2.3.4.4b|14.02.2023|Killian_Ma
ry_Lea||23-005620-
001|06.07.2023|National_Veterinary_Services_Laboratories_-
_USDA|National_Veterinary_Services_Laboratories_-_USDA|HA|4|HA"/>
NNN
</sequence>
<sequence>
<taxon idref="A/great_horned_owl/Colorado/23-005885-
001/2023|EPI_ISL_17964868|A_/_H5N1|Original||2.3.4.4b|17.02.2023|Killian_Ma
ry_Lea||23-005885-
001|06.07.2023|National_Veterinary_Services_Laboratories_-
_USDA|National_Veterinary_Services_Laboratories_-_USDA|HA|4|HA"/>
NNN
</sequence>
<sequence>
<taxon idref="A/great_horned_owl/Colorado/23-007822-
001/2023|EPI_ISL_17964907|A_/_H5N1|Original||2.3.4.4b|03.03.2023|Killian_Ma
ry_Lea||23-007822-
001|06.07.2023|National_Veterinary_Services_Laboratories_-
_USDA|National_Veterinary_Services_Laboratories_-_USDA|HA|4|HA"/>
NNN
</sequence>
<sequence>
<taxon idref="A/great_horned_owl/Colorado/23-007956-
001/2023|EPI_ISL_17964897|A_/_H5N1|Original||2.3.4.4b|07.03.2023|Killian_Ma
ry_Lea||23-007956-
001|06.07.2023|National_Veterinary_Services_Laboratories_-
_USDA|National_Veterinary_Services_Laboratories_-_USDA|HA|4|HA"/>
NNN
</sequence>
<sequence>
<taxon idref="A/great_horned_owl/Idaho/23-011169-
003/2023|EPI_ISL_17964927|A_/_H5N1|Original||2.3.4.4b|28.03.2023|Killian_Ma
ry_Lea||23-011169-
003|06.07.2023|National_Veterinary_Services_Laboratories_-
_USDA|National_Veterinary_Services_Laboratories_-_USDA|HA|4|HA_A/"/>
NNN
</sequence>
<sequence>
<taxon idref="A/great_horned_owl/Idaho/23-017158-
001/2023|EPI_ISL_17964981|A_/_H5N1|Original||2.3.4.4b|20.05.2023|Killian_Ma
ry_Lea||23-017158-
001|06.07.2023|National_Veterinary_Services_Laboratories_-
_USDA|National_Veterinary_Services_Laboratories_-_USDA|HA|4|HA_A/"/>
NNN
</sequence>
<sequence>
<taxon idref="A/great_horned_owl/Minnesota/22-041884-
001/2022|EPI_ISL_19607258|A_/_H5N1|Original||2.3.4.4b|07.12.2022||||12.12.2
024|USDA_APHIS_Veterinary_Services_Diagnostic_Virology_Laboratory_National_
Veterinary_Services_Laboratories||HA|4|A/great_horned_owl/M"/>
NNN
</sequence>
<sequence>

<taxon idref="A/great_horned_owl/Montana/23-011850-001/2023|EPI_ISL_17964939|A_/_H5N1|Original||2.3.4.4b|12.04.2023|Killian_Mary_Lea||23-011850-001|06.07.2023|National_Veterinary_Services_Laboratories_-_USDA|National_Veterinary_Services_Laboratories_-_USDA|HA|4|HA_"/>
NNN
</sequence>
<sequence>
<taxon idref="A/great_horned_owl/Nevada/22-040618-001/2022|EPI_ISL_19607219|A_/_H5N1|Original||2.3.4.4b|06.12.2022||||12.12.2024|USDA_APHIS_Veterinary_Services_Diagnostic_Virology_Laboratory_National_Veterinary_Services_Laboratories||HA|4|A/great_horned_owl/Neva"/>
NNN
</sequence>
<sequence>
<taxon idref="A/great_horned_owl/Oregon/22-041042-001/2022|EPI_ISL_19607226|A_/_H5N1|Original||2.3.4.4b|12.12.2022||||12.12.2024|USDA_APHIS_Veterinary_Services_Diagnostic_Virology_Laboratory_National_Veterinary_Services_Laboratories||HA|4|A/great_horned_owl/Oreg"/>
NNN
</sequence>
<sequence>
<taxon idref="A/great_horned_owl/Wyoming/22-039597-001/2022|EPI_ISL_19607274|A_/_H5N1|Original||2.3.4.4b|06.12.2022||||12.12.2024|USDA_APHIS_Veterinary_Services_Diagnostic_Virology_Laboratory_National_Veterinary_Services_Laboratories||HA|4|A/great_horned_owl/Wyo"/>
NNN
</sequence>
<sequence>
<taxon idref="A/great_horned_owl/Wyoming/22-041771-002/2022|EPI_ISL_19607287|A_/_H5N1|Original||2.3.4.4b|20.12.2022||||12.12.2024|USDA_APHIS_Veterinary_Services_Diagnostic_Virology_Laboratory_National_Veterinary_Services_Laboratories||HA|4|A/great_horned_owl/Wyo"/>
NNN
</sequence>
<sequence>
<taxon idref="A/greater_scaup/Montana/23-001334-001/2023|EPI_ISL_19605263|A_/_H5N1|Original||2.3.4.4b|06.01.2023||||12.12.2024|USDA_APHIS_Veterinary_Services_Diagnostic_Virology_Laboratory_National_Veterinary_Services_Laboratories||HA|4|A/greater_scaup/Montana/2"/>
NNN
</sequence>
<sequence>
<taxon idref="A/guanay_cormorant/Atacama/235520-1/2023|EPI_ISL_19151400|A_/_H5N1|Original||2.3.4.4b|05.02.2023||||21.05.2024|Emory_University__CEIRR_|Emory_University__CEIRR_|HA|4|A/guanay_cormorant/Atacama/235520-1/2023_HA|EPI3302324|DNA_IN"/>
NNN
</sequence>
<sequence>
<taxon idref="A/guanay_cormorant/Peru/CAL-INS-009/2023|EPI_ISL_17777530|A_/_H5N1|Original||2.3.4.4b|17.03.2023|Padilla_Rojas_Carlos_Patricio||1375|01.07.2023|Laboratorio_de_Virus_Respiratorios_Centro_Nacional_de_Salud_Publica|Instituto_Nacional_de_Salud_Peru|HA|4|"/>
NNN
</sequence>
<sequence>
<taxon idref="A/gull/Biobio/237012/2023|EPI_ISL_19410300|A_/_H5N1|Original||2.3.4.4b|15.02.2023||||12.09.2024|Emory_University__CEIRR_|Emory_University__CEIRR_|HA|4|A/gull/Biobio/237012/2023_HA|EPI3555425|DNA_IN"/>
NNN

</sequence>
<sequence>
<taxon idref="A/gull/California/23-000936-003/2022|EPI_ISL_19607556|A_/_H5N1|Original||2.3.4.4b|16.12.2022||||12.12.2024|USDA_APHIS_Veterinary_Services_Diagnostic_Virology_Laboratory_National_Veterinary_Services_Laboratories||HA|4|A/gull/California/23-000936-003"/>
NNN
</sequence>
<sequence>
<taxon idref="A/gull/Maule/234329-1/2023|EPI_ISL_18777130|A_/_H5N1|Original||2.3.4.4b|26.01.2023||||16.01.2024|Emory_University__CEIRR_|Emory_University__CEIRR_|HA|4|A/gull/Maule/234329-1/2023_HA|EPI2921503|DNA_IN"/>
NNN
</sequence>
<sequence>
<taxon idref="A/gull/Peru/LIM-INS-006/2023|EPI_ISL_17777527|A_/_H5N1|Original||2.3.4.4b|16.03.2023|Padilla_Rojas_Carlos_Patricio||1375|01.07.2023|Laboratorio_de_Virus_Respiratorios_Centro_Nacional_de_Salud_Publica|Instituto_Nacional_de_Salud_Peru|HA|4|Peru_INS006_"/>
NNN
</sequence>
<sequence>
<taxon idref="A/harbor_seal/Washington/23-025991-001/2023|EPI_ISL_18311025|A_/_H5N1|Original||2.3.4.4b|25.08.2023|Killian_Mary_Lea||23-025991-001|26.09.2023|National_Veterinary_Services_Laboratories_-_USDA|National_Veterinary_Services_Laboratories_-_USDA|HA|4|HA_A/"/>
NNN
</sequence>
<sequence>
<taxon idref="A/harbor_seal/Washington/23-026504-001/2023|EPI_ISL_18311027|A_/_H5N1|Original||2.3.4.4b|29.08.2023|Killian_Mary_Lea||23-026504-001|26.09.2023|National_Veterinary_Services_Laboratories_-_USDA|National_Veterinary_Services_Laboratories_-_USDA|HA|4|HA_A/"/>
NNN
</sequence>
<sequence>
<taxon idref="A/harbor_seal/Washington/23-027069-002/2023|EPI_ISL_18731638|A_/_H5N1|Original||2.3.4.4b|13.08.2023|Killian_Mary_Lea|23-027069-002||10.06.2024|National_Veterinary_Services_Laboratories_-_USDA|National_Veterinary_Services_Laboratories_-_USDA|HA|4|HA_A/"/>
NNN
</sequence>
<sequence>
<taxon idref="A/hawk/Iowa/23-011213-001/2023|EPI_ISL_17964928|A_/_H5N1|Original||2.3.4.4b|10.04.2023|Killian_Mary_Lea||23-011213-001|06.07.2023|National_Veterinary_Services_Laboratories_-_USDA|National_Veterinary_Services_Laboratories_-_USDA|HA|4|HA_A/hawk/Iowa/23-"/>
NNN
</sequence>
<sequence>
<taxon idref="A/hawk/Missouri/22-040785-002/2022|EPI_ISL_19607573|A_/_H5N1|Original||2.3.4.4b|05.12.2022||||12.12.2024|USDA_APHIS_Veterinary_Services_Diagnostic_Virology_Laboratory_National_Veterinary_Services_Laboratories||HA|4|A/hawk/Missouri/22-040785-002/202"/>

NNN
</sequence>
<sequence>
<taxon idref="A/heron/Antofagasta/228705-
3/2022|EPI_ISL_17885969|A_/_H5N1|Original||2.3.4.4b|20.12.2022|GISAID_EpiFl
u_Data_Curator|||29.06.2023|CEIRS_Data_Processing_and_Coordinating_Center_C
enter_for_Research_on_Influenza_Pathogenesis__CRIP_|CEIRS_Data_Processing"/
>
NNN
</sequence>
<sequence>
<taxon idref="A/herring_gull/California/23-001053-
004/2022|EPI_ISL_19607536|A_/_H5N1|Original||2.3.4.4b|17.12.2022||||12.12.2
024|USDA_APHIS_Veterinary_Services_Diagnostic_Virology_Laboratory_National_
Veterinary_Services_Laboratories||HA|4|A/herring_gull/Californ"/>
NNN
</sequence>
<sequence>
<taxon idref="A/herring_gull/California/23-001053-
006/2022|EPI_ISL_19607538|A_/_H5N1|Original||2.3.4.4b|17.12.2022||||12.12.2
024|USDA_APHIS_Veterinary_Services_Diagnostic_Virology_Laboratory_National_
Veterinary_Services_Laboratories||HA|4|A/herring_gull/Californ"/>
NNN
</sequence>
<sequence>
<taxon idref="A/hooded_merganser/Washington/23-003784-
001/2023|EPI_ISL_19607623|A_/_H5N1|Original||2.3.4.4b|18.01.2023||||12.12.2
024|USDA_APHIS_Veterinary_Services_Diagnostic_Virology_Laboratory_National_
Veterinary_Services_Laboratories||HA|4|A/hooded_merganser/"/>
NNN
</sequence>
<sequence>
<taxon idref="A/humboldt_penguin/Tarapaca/238744-
2/2023|EPI_ISL_17885947|A_/_H5N1|Original||2.3.4.4b|23.02.2023|GISAID_EpiFl
u_Data_Curator|||24.07.2023|||HA|4|A/humboldt_penguin/Tarapaca/238744-
2/2023_HA|EPI2644734|DNA_IN"/>
NNN
</sequence>
<sequence>
<taxon idref="A/kelp_gull/Atacama/234251-
1/2023|EPI_ISL_19410282|A_/_H5N1|Original||2.3.4.4b|19.01.2023||||12.09.202
4|Emory_University__CEIRR_|Emory_University__CEIRR_|HA|4|A/kelp_gull/Atacam
a/234251-1/2023_HA|EPI3555308|DNA_IN"/>
NNN
</sequence>
<sequence>
<taxon idref="A/kelp_gull/Valparaiso/236011-
1/2023|EPI_ISL_19404906|A_/_H5N1|Original||2.3.4.4b|09.02.2023||||10.09.202
4|Emory_University__CEIRR_|Emory_University__CEIRR_|HA|4|A/kelp_gull/Valpar
aiso/236011-1/2023_HA|EPI3550194|DNA_IN"/>
NNN
</sequence>
<sequence>
<taxon idref="A/lesser_white-fronted_goose/Mississippi/22-041057-
001/2022|EPI_ISL_19608573|A_/_H5N1|Original||2.3.4.4b|08.12.2022||||12.12.2
024|USDA_APHIS_Veterinary_Services_Diagnostic_Virology_Laboratory_National_
Veterinary_Services_Laboratories||HA|4|A/lesser"/>
NNN
</sequence>
<sequence>

<taxon idref="A/lion/Peru/AIS0554/2023|EPI_ISL_17805999|A_/_H5N1|Original||2.3.4.4b|08.02.2023|GISAID_EpiFlu_Data_Curator|||15.06.2023|||HA|4|A/lion/Peru/AIS0554/2023_HA|EPI2597244|DNA_IN"/>
NNN
</sequence>
<sequence>
<taxon idref="A/magpie/Colorado/23-000401-001/2022|EPI_ISL_19608574|A_/_H5N1|Original||2.3.4.4b|27.12.2022||||12.12.2024|USDA_APHIS_Veterinary_Services_Diagnostic_Virology_Laboratory_National_Veterinary_Services_Laboratories||HA|4|A/magpie/Colorado/23-000401-001"/>
NNN
</sequence>
<sequence>
<taxon idref="A/magpie/Colorado/23-002236-002/2023|EPI_ISL_19608575|A_/_H5N1|Original||2.3.4.4b|11.01.2023||||12.12.2024|USDA_APHIS_Veterinary_Services_Diagnostic_Virology_Laboratory_National_Veterinary_Services_Laboratories||HA|4|A/magpie/Colorado/23-002236-002"/>
NNN
</sequence>
<sequence>
<taxon idref="A/magpie/Colorado/23-003934-002/2023|EPI_ISL_19608576|A_/_H5N1|Original||2.3.4.4b|17.01.2023||||12.12.2024|USDA_APHIS_Veterinary_Services_Diagnostic_Virology_Laboratory_National_Veterinary_Services_Laboratories||HA|4|A/magpie/Colorado/23-003934-002"/>
NNN
</sequence>
<sequence>
<taxon idref="A/magpie/Colorado/23-004705-001-/2023|EPI_ISL_17964848|A_/_H5N1|Original||2.3.4.4b|07.02.2023|Killian_Mary_Lea||23-004705-001|06.07.2023|National_Veterinary_Services_Laboratories_-_USDA|National_Veterinary_Services_Laboratories_-_USDA|HA|4|HA_A/magpie"/>
NNN
</sequence>
<sequence>
<taxon idref="A/magpie/Idaho/23-001085-001/2023|EPI_ISL_19608578|A_/_H5N1|Original||2.3.4.4b|04.01.2023||||12.12.2024|USDA_APHIS_Veterinary_Services_Diagnostic_Virology_Laboratory_National_Veterinary_Services_Laboratories||HA|4|A/magpie/Idaho/23-001085-001/2023_"/>
NNN
</sequence>
<sequence>
<taxon idref="A/magpie/Idaho/23-011169-002/2023|EPI_ISL_17964926|A_/_H5N1|Original||2.3.4.4b|28.03.2023|Killian_Mary_Lea||23-011169-002|06.07.2023|National_Veterinary_Services_Laboratories_-_USDA|National_Veterinary_Services_Laboratories_-_USDA|HA|4|HA_A/magpie/Ida"/>
NNN
</sequence>
<sequence>
<taxon idref="A/mallard/Montana/22-039947-005/2022|EPI_ISL_19604023|A_/_H5N1|Original||2.3.4.4b|02.12.2022||||11.12.2024|USDA_APHIS_Veterinary_Services_Diagnostic_Virology_Laboratory_National_Veterinary_Services_Laboratories||HA|4|A/mallard/Montana/22-039947-005"/>
NNN
</sequence>
<sequence>
<taxon idref="A/merganser/Colorado/22-039962-001/2022|EPI_ISL_19604070|A_/_H5N1|Original||2.3.4.4b|06.12.2022||||12.12.2

024|USDA_APHIS_Veterinary_Services_Diagnostic_Virology_Laboratory_National_
Veterinary_Services_Laboratories||HA|4|A/merganser/Colorado/22-0399"/>
NNN
</sequence>
<sequence>
<taxon idref="A/mottled_duck/South_Carolina/22-042180-
002/2022|EPI_ISL_19604312|A_/_H5N1|Original||2.3.4.4b|16.12.2022||||12.12.2
024|USDA_APHIS_Veterinary_Services_Diagnostic_Virology_Laboratory_National_
Veterinary_Services_Laboratories||HA|4|A/mottled_duck/Sout"/>
NNN
</sequence>
<sequence>
<taxon idref="A/mute_swan/Rhode_Island/23-007092-
001/2023|EPI_ISL_19604294|A_/_H5N1|Original||2.3.4.4b|13.02.2023||||12.12.2
024|USDA_APHIS_Veterinary_Services_Diagnostic_Virology_Laboratory_National_
Veterinary_Services_Laboratories||HA|4|A/mute_swan/Rhode_Island"/>
NNN
</sequence>
<sequence>
<taxon idref="A/northern_shoveler/Arkansas/23-002690-
001/2023|EPI_ISL_19604370|A_/_H5N1|Original||2.3.4.4b|14.01.2023||||12.12.2
024|USDA_APHIS_Veterinary_Services_Diagnostic_Virology_Laboratory_National_
Veterinary_Services_Laboratories||HA|4|A/northern_shoveler/"/>
NNN
</sequence>
<sequence>
<taxon idref="A/pelican/Antofagasta/228318-
1/2022|EPI_ISL_17885928|A_/_H5N1|Original||2.3.4.4b|16.12.2022|GISAID_EpiFl
u_Data_Curator|||19.07.2023|||HA|4|A/pelican/Antofagasta/228318-
1/2022_HA|EPI2609318|DNA_IN"/>
NNN
</sequence>
<sequence>
<taxon idref="A/pelican/Bio_Bio/236574/2023|EPI_ISL_18760061|A_/_H5N1|Origi
nal||2.3.4.4b|13.02.2023||||14.01.2024|Emory_University__CEIRR_|Emory_Unive
rsity__CEIRR_|HA|4|A/pelican/Bio_Bio/236574/2023_HA|EPI2913191|DNA_IN"/>
NNN
</sequence>
<sequence>
<taxon idref="A/pelican/Chile/229424-
4/2022|EPI_ISL_19391461|A_/_H5N1|Original||2.3.4.4b|22.12.2022||||09.09.202
4|CEIRS_Data_Processing_and_Coordinating_Center_Center_for_Research_on_Infl
uenza_Pathogenesis__CRIP_||HA|4|A/pelican/Chile/229424-
4/2022_HA|EPI3549636|DN"/>
NNN
</sequence>
<sequence>
<taxon idref="A/pelican/Costa_Rica/INC-0018-D0370-
23/2023|EPI_ISL_17965774|A_/_H5N1|Original||2.3.4.4b|26.01.2023|Soto-
Garita_Claudio|D0370-P|786338|18.07.2023|SENASA-
LANASEVE|Inciensa_Investigacion_y_Ensenanza_en_Nutricion_y_Salud|HA|4|A/pel
ican/Costa_Rica/INC-00"/>
NNN
</sequence>
<sequence>
<taxon idref="A/pelican/Costa_Rica/INC-0019-D0618-
23/2023|EPI_ISL_17965775|A_/_H5N1|Original||2.3.4.4b|10.02.2023|Soto-
Garita_Claudio|D0618-23|786339|18.07.2023|SENASA-
LANASEVE|Inciensa_Investigacion_y_Ensenanza_en_Nutricion_y_Salud|HA|4|A/pel
ican/Costa_Rica/INC-0"/>
NNN

</sequence>
<sequence>
<taxon idref="A/pelican/Costa_Rica/INC-0020-D0716-
23/2023|EPI_ISL_17965776|A_/_H5N1|Original||2.3.4.4b|10.02.2023|Soto-
Garita_Claudio|D0726-
23Tc|786340|18.07.2023|Servicio_Nacional_de_Salud_Animal__SENASA_|Inciensa_
Investigacion_y_Ensenanza_en_Nutricion_y_Salud|HA"/>
NNN
</sequence>
<sequence>
<taxon idref="A/pelican/Costa_Rica/INC-0021-D0729-
23/2023|EPI_ISL_17965777|A_/_H5N1|Original||2.3.4.4b|10.02.2023|Soto-
Garita_Claudio|D0729-23|786341|18.07.2023|SENASA-
LANASEVE|Inciensa_Investigacion_y_Ensenanza_en_Nutricion_y_Salud|HA|4|A/pel
ican/Costa_Rica/INC-0"/>
NNN
</sequence>
<sequence>
<taxon idref="A/pelican/Guatemala/23-003730-
001/2023|EPI_ISL_18863889|A_/_H5N1|Original||2.3.4.4b|27.01.2023|Killian_Ma
ry_Lea||23-003730-
001|05.02.2024|National_Veterinary_Services_Laboratories_-
_USDA|National_Veterinary_Services_Laboratories_-_USDA|HA|4|HA_A/pelic"/>
NNN
</sequence>
<sequence>
<taxon idref="A/pelican/Guatemala/23-003730-
002/2023|EPI_ISL_18863890|A_/_H5N1|Original||2.3.4.4b|27.01.2023|Killian_Ma
ry_Lea||23-003730-
002|05.02.2024|National_Veterinary_Services_Laboratories_-
_USDA|National_Veterinary_Services_Laboratories_-_USDA|HA|4|HA_A/pelic"/>
NNN
</sequence>
<sequence>
<taxon idref="A/pelican/Honduras/23-000009-
001/2022|EPI_ISL_17558875|A_/_H5N1|E1||2.3.4.4b|27.12.2022|Killian_Mary_Lea
||23-000009-
001|27.04.2023|Instituto_Hondureno_de_Investigaciones_Medico_Veterinarias_S
AG-SENASA|National_Veterinary_Services_Laboratories_-_USDA|H"/>
NNN
</sequence>
<sequence>
<taxon idref="A/pelican/Valparaiso/233091-
1/2023|EPI_ISL_17885930|A_/_H5N1|Original||2.3.4.4b|20.01.2023|GISAID_EpiFl
u_Data_Curator|||19.07.2023|||HA|4|A/pelican/Valparaiso/233091-
1/2023_HA|EPI2609327|DNA_IN"/>
NNN
</sequence>
<sequence>
<taxon idref="A/pelican/Valparaiso/233091-
2/2023|EPI_ISL_17885956|A_/_H5N1|Original||2.3.4.4b|20.01.2023|GISAID_EpiFl
u_Data_Curator|||29.06.2023|||HA|4|A/pelican/Valparaiso/233091-
2/2023_HA|EPI2609431|DNA_IN"/>
NNN
</sequence>
<sequence>
<taxon idref="A/pelican/Valparaiso/233418-
1/2023|EPI_ISL_17885932|A_/_H5N1|Original||2.3.4.4b|20.01.2023|GISAID_EpiFl
u_Data_Curator|||24.07.2023|||HA|4|A/pelican/Valparaiso/233418-
1/2023_HA|EPI2637126|DNA_IN"/>
NNN

</sequence>
<sequence>
<taxon idref="A/pelican/Valparaiso/233447-2/2023|EPI_ISL_17885985|A_/_H5N1|Original||2.3.4.4b|21.01.2023|GISAID_EpiFlu_Data_Curator|||19.07.2023|||HA|4|A/pelican/Valparaiso/233447-2/2023_HA|EPI2609614|DNA_IN"/>
NNN
</sequence>
<sequence>
<taxon idref="A/pelican/Valparaiso/233450-1/2023|EPI_ISL_17885983|A_/_H5N1|Original||2.3.4.4b|23.01.2023|GISAID_EpiFlu_Data_Curator|||19.07.2023|||HA|4|A/pelican/Valparaiso/233450-1/2023_HA|EPI2609600|DNA_IN"/>
NNN
</sequence>
<sequence>
<taxon idref="A/pelican/Valparaiso/234040/2023|EPI_ISL_17885982|A_/_H5N1|Original||2.3.4.4b|25.01.2023|GISAID_EpiFlu_Data_Curator|||19.07.2023|||HA|4|A/pelican/Valparaiso/234040/2023_HA|EPI2609588|DNA_IN"/>
NNN
</sequence>
<sequence>
<taxon idref="A/peregrine_falcon/Antofagasta/235144-1/2023|EPI_ISL_19410283|A_/_H5N1|Original||2.3.4.4b|03.02.2023||||12.09.2024|Emory_University__CEIRR_|Emory_University__CEIRR_|HA|4|A/peregrine_falcon/Antofagasta/235144-1/2023_HA|EPI3555314|DNA_IN"/>
NNN
</sequence>
<sequence>
<taxon idref="A/peregrine_falcon/BC/AIVPHL-322/2022|EPI_ISL_17051459|A_/_H5N1|Original||2.3.4.4b|12.12.2022|Russell_Shannon_Laurel|||28.02.2023|B.C._Centre_for_Disease_Control|British_Columbia_Centre_for_Disease_Control|HA|4|A/peregrine_falcon/BC/AIVPHL-322/2022_HA"/>
NNN
</sequence>
<sequence>
<taxon idref="A/peregrine_falcon/California/23-011277-001/2023|EPI_ISL_17964933|A_/_H5N1|Original||2.3.4.4b|30.03.2023|Killian_Mary_Lea||23-011277-001|06.07.2023|National_Veterinary_Services_Laboratories_-_USDA|National_Veterinary_Services_Laboratories_-_USDA|HA|4|"/>
NNN
</sequence>
<sequence>
<taxon idref="A/peregrine_falcon/Michigan/23-003553-001/2023|EPI_ISL_19604230|A_/_H5N1|Original||2.3.4.4b|26.01.2023||||12.12.2024|USDA_APHIS_Veterinary_Services_Diagnostic_Virology_Laboratory_National_Veterinary_Services_Laboratories||HA|4|A/peregrine_falcon/Mi"/>
NNN
</sequence>
<sequence>
<taxon idref="A/peruvian_booby/Coquimbo/242304/2023|EPI_ISL_19410277|A_/_H5N1|Original||2.3.4.4b|15.03.2023||||12.09.2024|Emory_University__CEIRR_||HA|4|A/peruvian_booby/Coquimbo/242304/2023_HA|EPI3555275|DNA_IN"/>
NNN
</sequence>
<sequence>
<taxon idref="A/peruvian_booby/Peru/CAL-INS-007/2023|EPI_ISL_17777528|A_/_H5N1|Original||2.3.4.4b|17.03.2023|Padilla_Rojas_Carlos_Patricio||1375|01.07.2023|Laboratorio_de_Virus_Respiratorios_Centro_Nacional_de_Salud_Publica|Instituto_Nacional_de_Salud_Peru|HA|4|Pe"/>

NNN
</sequence>
<sequence>
<taxon idref="A/peruvian_booby/Peru/CAL-INS-008/2023|EPI_ISL_17777529|A_/_H5N1|Original||2.3.4.4b|17.03.2023|Padilla_Rojas_Carlos_Patricio||1375|01.07.2023|Laboratorio_de_Virus_Respiratorios_Centro_Nacional_de_Salud_Publica|Instituto_Nacional_de_Salud_Peru|HA|4|Pe"/>
NNN
</sequence>
<sequence>
<taxon idref="A/peruvian_booby/Peru/LIM-INS-004/2023|EPI_ISL_17777525|A_/_H5N1|Original||2.3.4.4b|03.04.2023|Padilla_Rojas_Carlos_Patricio||1375|01.07.2023|Laboratorio_de_Virus_Respiratorios_Centro_Nacional_de_Salud_Publica|Instituto_Nacional_de_Salud_Peru|HA|4|Pe"/>
NNN
</sequence>
<sequence>
<taxon idref="A/peruvian_booby/Peru/LIM-INS-005/2023|EPI_ISL_17777526|A_/_H5N1|Original||2.3.4.4b|20.03.2023|Padilla_Rojas_Carlos_Patricio||1375|01.07.2023|Laboratorio_de_Virus_Respiratorios_Centro_Nacional_de_Salud_Publica|Instituto_Nacional_de_Salud_Peru|HA|4|Pe"/>
NNN
</sequence>
<sequence>
<taxon idref="A/peruvian_booby/Peru/LIM-INS-012/2023|EPI_ISL_17777533|A_/_H5N1|Original||2.3.4.4b|12.04.2023|Padilla_Rojas_Carlos_Patricio||1375|01.07.2023|Laboratorio_de_Virus_Respiratorios_Centro_Nacional_de_Salud_Publica|Instituto_Nacional_de_Salud_Peru|HA|4|Pe"/>
NNN
</sequence>
<sequence>
<taxon idref="A/pinniped/Uruguay/P10_8923/2023|EPI_ISL_19070481|A_/_H5N1|Original||2.3.4.4b|08.09.2023||||24.04.2024|Universidad_de_la_Republica_Facultad_de_Ciencias_Genetica_Evolutiva/Division_de_Laboratorios_Veterinarios_Plataforma_Genomica||HA|4|A/pinniped/Uru"/>
NNN
</sequence>
<sequence>
<taxon idref="A/pinniped/Uruguay/P13_11923/2023|EPI_ISL_19070482|A_/_H5N1|Original||2.3.4.4b|11.09.2023||||24.04.2024|Universidad_de_la_Republica_Facultad_de_Ciencias_Genetica_Evolutiva/Division_de_Laboratorios_Veterinarios_Plataforma_Genomica||HA|4|A/pinniped/Ur"/>
NNN
</sequence>
<sequence>
<taxon idref="A/pinniped/Uruguay/P14_11923/2023|EPI_ISL_19070483|A_/_H5N1|Original||2.3.4.4b|11.09.2023||||24.04.2024|Universidad_de_la_Republica_Facultad_de_Ciencias_Genetica_Evolutiva/Division_de_Laboratorios_Veterinarios_Plataforma_Genomica||HA|4|A/pinniped/Ur"/>
NNN
</sequence>
<sequence>
<taxon idref="A/pinniped/Uruguay/P15_14923/2023|EPI_ISL_19070484|A_/_H5N1|Original||2.3.4.4b|14.09.2023||||24.04.2024|Universidad_de_la_Republica_Facultad_de_Ciencias_Genetica_Evolutiva/Division_de_Laboratorios_Veterinarios_Plataforma_Genomica||HA|4|A/pinniped/Ur"/>
NNN
</sequence>
<sequence>
<taxon idref="A/pinniped/Uruguay/P17_14923/2023|EPI_ISL_19070485|A_/_H5N1|Original||2.3.4.4b|14.09.2023||||24.04.2024|Universidad_de_la_Republica_Facu

ltad_de_Ciencias_Genetica_Evolutiva/Division_de_Laboratorios_Veterinarios_P
lataforma_Genomica||HA|4|A/pinniped/Ur"/>
NNN
</sequence>
<sequence>
<taxon idref="A/pinniped/Uruguay/P18_14923/2023|EPI_ISL_19070486|A_/_H5N1|O
riginal||2.3.4.4b|14.09.2023||||24.04.2024|Universidad_de_la_Republica_Facu
ltad_de_Ciencias_Genetica_Evolutiva/Division_de_Laboratorios_Veterinarios_P
lataforma_Genomica||HA|4|A/pinniped/Ur"/>
NNN
</sequence>
<sequence>
<taxon idref="A/pinniped/Uruguay/P26_21023/2023|EPI_ISL_19070488|A_/_H5N1|O
riginal||2.3.4.4b|02.10.2023||||24.04.2024|Universidad_de_la_Republica_Facu
ltad_de_Ciencias_Genetica_Evolutiva/Division_de_Laboratorios_Veterinarios_P
lataforma_Genomica||HA|4|A/pinniped/Ur"/>
NNN
</sequence>
<sequence>
<taxon idref="A/pinniped/Uruguay/P4_6923/2023|EPI_ISL_19070498|A_/_H5N1|Ori
ginal||2.3.4.4b|06.09.2023||||24.04.2024|Universidad_de_la_Republica_Facult
ad_de_Ciencias_Genetica_Evolutiva/Division_de_Laboratorios_Veterinarios_Pla
taforma_Genomica||HA|4|A/pinniped/Urug"/>
NNN
</sequence>
<sequence>
<taxon idref="A/pinniped/Uruguay/P5_6923/2023|EPI_ISL_19070489|A_/_H5N1|Ori
ginal||2.3.4.4b|06.09.2023||||24.04.2024|Universidad_de_la_Republica_Facult
ad_de_Ciencias_Genetica_Evolutiva/Division_de_Laboratorios_Veterinarios_Pla
taforma_Genomica||HA|4|A/pinniped/Urug"/>
NNN
</sequence>
<sequence>
<taxon idref="A/pinniped/Uruguay/P6_6923/2023|EPI_ISL_19070490|A_/_H5N1|Ori
ginal||2.3.4.4b|06.09.2023||||24.04.2024|Universidad_de_la_Republica_Facult
ad_de_Ciencias_Genetica_Evolutiva/Division_de_Laboratorios_Veterinarios_Pla
taforma_Genomica||HA|4|A/pinniped/Urug"/>
NNN
</sequence>
<sequence>
<taxon idref="A/pinniped/Uruguay/P7_6923/2023|EPI_ISL_19070491|A_/_H5N1|Ori
ginal||2.3.4.4b|06.09.2023||||24.04.2024|Universidad_de_la_Republica_Facult
ad_de_Ciencias_Genetica_Evolutiva/Division_de_Laboratorios_Veterinarios_Pla
taforma_Genomica||HA|4|A/pinniped/Urug"/>
NNN
</sequence>
<sequence>
<taxon idref="A/pinniped/Uruguay/P8_8923/2023|EPI_ISL_19070492|A_/_H5N1|Ori
ginal||2.3.4.4b|08.09.2023||||24.04.2024|Universidad_de_la_Republica_Facult
ad_de_Ciencias_Genetica_Evolutiva/Division_de_Laboratorios_Veterinarios_Pla
taforma_Genomica||HA|4|A/pinniped/Urug"/>
NNN
</sequence>
<sequence>
<taxon idref="A/porpoise/Antofagasta/SJCEIRR-
2465061/2023|EPI_ISL_18777129|A_/_H5N1|Original||2.3.4.4b|31.03.2023||||16.
01.2024|Emory_University__CEIRR_|Emory_University__CEIRR_|HA|4|A/porpoise/A
ntofagasta/SJCEIRR-2465061/2023|EPI2921496|DNA_IN"/>
NNN
</sequence>
<sequence>

<taxon idref="A/porpoise/Antofagasta/SJCEIRR-
2465062/2023|EPI_ISL_18777141|A_/_H5N1|Original||2.3.4.4b|31.03.2023||||16.
01.2024|Emory_University__CEIRR_|Emory_University__CEIRR_|HA|4|A/porpoise/A
ntofagasta/SJCEIRR-2465062/2023|EPI2921533|DNA_IN"/>
NNN
</sequence>
<sequence>
<taxon idref="A/porpoise/Atacama/SJCEIRR-
245355/2023|EPI_ISL_18777140|A_/_H5N1|Original||2.3.4.4b|27.03.2023||||16.0
1.2024|Emory_University__CEIRR_|Emory_University__CEIRR_|HA|4|A/porpoise/At
acama/SJCEIRR-245355/2023|EPI2921527|DNA_IN"/>
NNN
</sequence>
<sequence>
<taxon idref="A/raven/California/23-003756-
001/2023|EPI_ISL_19604281|A_/_H5N1|Original||2.3.4.4b|29.01.2023||||12.12.2
024|USDA_APHIS_Veterinary_Services_Diagnostic_Virology_Laboratory_National_
Veterinary_Services_Laboratories||HA|4|A/raven/California/23-003756-0"/>
NNN
</sequence>
<sequence>
<taxon idref="A/raven/Colorado/23-003562-
001/2023|EPI_ISL_19604258|A_/_H5N1|Original||2.3.4.4b|27.01.2023||||12.12.2
024|USDA_APHIS_Veterinary_Services_Diagnostic_Virology_Laboratory_National_
Veterinary_Services_Laboratories||HA|4|A/raven/Colorado/23-003562-001/2"/>
NNN
</sequence>
<sequence>
<taxon idref="A/raven/Colorado/23-003563-
001/2023|EPI_ISL_19604257|A_/_H5N1|Original||2.3.4.4b|25.01.2023||||12.12.2
024|USDA_APHIS_Veterinary_Services_Diagnostic_Virology_Laboratory_National_
Veterinary_Services_Laboratories||HA|4|A/raven/Colorado/23-003563-001/2"/>
NNN
</sequence>
<sequence>
<taxon idref="A/raven/Montana/23-003666-
002/2023|EPI_ISL_19604256|A_/_H5N1|Original||2.3.4.4b|30.01.2023||||12.12.2
024|USDA_APHIS_Veterinary_Services_Diagnostic_Virology_Laboratory_National_
Veterinary_Services_Laboratories||HA|4|A/raven/Montana/23-003666-002/202"/>
NNN
</sequence>
<sequence>
<taxon idref="A/red-tailed_hawk/BC/AIVPHL-
339/2022|EPI_ISL_17051467|A_/_H5N1|Original||2.3.4.4b|19.12.2022|Russell_Sh
annon_Laurel|||28.02.2023|B.C._Centre_for_Disease_Control|British_Columbia_
Centre_for_Disease_Control|HA|4|A/red-tailed_hawk/BC/AIVPHL-
339/2022_HA|E"/>
NNN
</sequence>
<sequence>
<taxon idref="A/red-tailed_hawk/California/23-006644-
003/2023|EPI_ISL_17964878|A_/_H5N1|Original||2.3.4.4b|14.02.2023|Killian_Ma
ry_Lea||23-006644-
003|06.07.2023|National_Veterinary_Services_Laboratories_-
_USDA|National_Veterinary_Services_Laboratories_-_USDA|HA|4|H"/>
NNN
</sequence>
<sequence>
<taxon idref="A/red-tailed_hawk/Colorado/23-005618-
001/2023|EPI_ISL_17964859|A_/_H5N1|Original||2.3.4.4b|14.02.2023|Killian_Ma
ry_Lea||23-005618-

001|06.07.2023|National_Veterinary_Services_Laboratories_-
_USDA|National_Veterinary_Services_Laboratories_-_USDA|HA|4|HA_"/>
NNN
</sequence>
<sequence>
<taxon idref="A/red-tailed_hawk/Colorado/23-006671-
001/2023|EPI_ISL_17964882|A_/_H5N1|Original||2.3.4.4b|18.02.2023|Killian_Ma
ry_Lea||23-006671-
001|06.07.2023|National_Veterinary_Services_Laboratories_-
_USDA|National_Veterinary_Services_Laboratories_-_USDA|HA|4|HA_"/>
NNN
</sequence>
<sequence>
<taxon idref="A/red-tailed_hawk/Colorado/23-008901-
001/2023|EPI_ISL_17964906|A_/_H5N1|Original||2.3.4.4b|15.03.2023|Killian_Ma
ry_Lea||23-008901-
001|06.07.2023|National_Veterinary_Services_Laboratories_-
_USDA|National_Veterinary_Services_Laboratories_-_USDA|HA|4|HA_"/>
NNN
</sequence>
<sequence>
<taxon idref="A/red-tailed_hawk/Kansas/23-009211-
001/2023|EPI_ISL_17964913|A_/_H5N1|Original||2.3.4.4b|15.03.2023|Killian_Ma
ry_Lea||23-009211-
001|06.07.2023|National_Veterinary_Services_Laboratories_-
_USDA|National_Veterinary_Services_Laboratories_-_USDA|HA|4|HA_A/"/>
NNN
</sequence>
<sequence>
<taxon idref="A/red-tailed_hawk/Kentucky/W23-
143/2022|EPI_ISL_17424631|A_/_H5N1|||2.3.4.4b|22.12.2022||||08.04.2023|St._
Jude_Center_of_Excellence_for_Influenza_Research_and_Response_-
_CEIRR_Influenza_Data_Processing_and_Communication_Center|St._Jude_Center_o
f_Excell"/>
NNN
</sequence>
<sequence>
<taxon idref="A/red-tailed_hawk/Minnesota/23-010018-
001/2023|EPI_ISL_17964919|A_/_H5N1|Original||2.3.4.4b|26.03.2023|Killian_Ma
ry_Lea||23-010018-
001|06.07.2023|National_Veterinary_Services_Laboratories_-
_USDA|National_Veterinary_Services_Laboratories_-_USDA|HA|4|HA"/>
NNN
</sequence>
<sequence>
<taxon idref="A/red-tailed_hawk/Montana/23-011216-
001/2023|EPI_ISL_17964929|A_/_H5N1|Original||2.3.4.4b|03.04.2023|Killian_Ma
ry_Lea||23-011216-
001|06.07.2023|National_Veterinary_Services_Laboratories_-
_USDA|National_Veterinary_Services_Laboratories_-_USDA|HA|4|HA_A"/>
NNN
</sequence>
<sequence>
<taxon idref="A/red-tailed_hawk/Nevada/23-012114-
001/2023|EPI_ISL_17964944|A_/_H5N1|Original||2.3.4.4b|31.03.2023|Killian_Ma
ry_Lea||23-012114-
001|06.07.2023|National_Veterinary_Services_Laboratories_-
_USDA|National_Veterinary_Services_Laboratories_-_USDA|HA|4|HA_A/"/>
NNN
</sequence>
<sequence>

<taxon idref="A/red-tailed_hawk/South_Dakota/23-012031-
001/2023|EPI_ISL_17964942|A_/_H5N1|Original||2.3.4.4b|11.04.2023|Killian_Ma
ry_Lea||23-012031-
001|06.07.2023|National_Veterinary_Services_Laboratories_-
_USDA|National_Veterinary_Services_Laboratories_-_USDA|HA|4"/>
NNN
</sequence>
<sequence>
<taxon idref="A/red_necked_phalarope/Oregon/23-000773-
001/2023|EPI_ISL_19604238|A_/_H5N1|Original||2.3.4.4b|04.01.2023||||12.12.2
024|USDA_APHIS_Veterinary_Services_Diagnostic_Virology_Laboratory_National_
Veterinary_Services_Laboratories||HA|4|A/red_necked_phalar"/>
NNN
</sequence>
<sequence>
<taxon idref="A/red_tailed_hawk/California/22-041402-
001/2022|EPI_ISL_19604249|A_/_H5N1|Original||2.3.4.4b|01.12.2022||||12.12.2
024|USDA_APHIS_Veterinary_Services_Diagnostic_Virology_Laboratory_National_
Veterinary_Services_Laboratories||HA|4|A/red_tailed_hawk/Ca"/>
NNN
</sequence>
<sequence>
<taxon idref="A/red_tailed_hawk/California/23-000278-
001/2022|EPI_ISL_19604252|A_/_H5N1|Original||2.3.4.4b|14.12.2022||||12.12.2
024|USDA_APHIS_Veterinary_Services_Diagnostic_Virology_Laboratory_National_
Veterinary_Services_Laboratories||HA|4|A/red_tailed_hawk/Ca"/>
NNN
</sequence>
<sequence>
<taxon idref="A/red_tailed_hawk/California/23-000279-
002/2022|EPI_ISL_19604253|A_/_H5N1|Original||2.3.4.4b|15.12.2022||||12.12.2
024|USDA_APHIS_Veterinary_Services_Diagnostic_Virology_Laboratory_National_
Veterinary_Services_Laboratories||HA|4|A/red_tailed_hawk/Ca"/>
NNN
</sequence>
<sequence>
<taxon idref="A/red_tailed_hawk/California/23-001055-
001/2022|EPI_ISL_19604254|A_/_H5N1|Original||2.3.4.4b|21.12.2022||||12.12.2
024|USDA_APHIS_Veterinary_Services_Diagnostic_Virology_Laboratory_National_
Veterinary_Services_Laboratories||HA|4|A/red_tailed_hawk/Ca"/>
NNN
</sequence>
<sequence>
<taxon idref="A/red_tailed_hawk/California/23-001856-
004/2022|EPI_ISL_19604378|A_/_H5N1|Original||2.3.4.4b|19.12.2022||||12.12.2
024|USDA_APHIS_Veterinary_Services_Diagnostic_Virology_Laboratory_National_
Veterinary_Services_Laboratories||HA|4|A/red_tailed_hawk/Ca"/>
NNN
</sequence>
<sequence>
<taxon idref="A/red_tailed_hawk/California/23-004363-
001/2023|EPI_ISL_19604802|A_/_H5N1|Original||2.3.4.4b|25.01.2023||||12.12.2
024|USDA_APHIS_Veterinary_Services_Diagnostic_Virology_Laboratory_National_
Veterinary_Services_Laboratories||HA|4|A/red_tailed_hawk/Ca"/>
NNN
</sequence>
<sequence>
<taxon idref="A/red_tailed_hawk/Colorado/22-041828-
001/2022|EPI_ISL_19605765|A_/_H5N1|Original||2.3.4.4b|20.12.2022||||12.12.2
024|USDA_APHIS_Veterinary_Services_Diagnostic_Virology_Laboratory_National_
Veterinary_Services_Laboratories||HA|4|A/red_tailed_hawk/Colo"/>

NNN
</sequence>
<sequence>
<taxon idref="A/red_tailed_hawk/Colorado/22-041830-001/2022|EPI_ISL_19605766|A_/_H5N1|Original||2.3.4.4b|20.12.2022||||12.12.2024|USDA_APHIS_Veterinary_Services_Diagnostic_Virology_Laboratory_National_Veterinary_Services_Laboratories||HA|4|A/red_tailed_hawk/Colo"/>
NNN
</sequence>
<sequence>
<taxon idref="A/red_tailed_hawk/Colorado/22-042170-002/2022|EPI_ISL_19605768|A_/_H5N1|Original||2.3.4.4b|25.12.2022||||12.12.2024|USDA_APHIS_Veterinary_Services_Diagnostic_Virology_Laboratory_National_Veterinary_Services_Laboratories||HA|4|A/red_tailed_hawk/Colo"/>
NNN
</sequence>
<sequence>
<taxon idref="A/red_tailed_hawk/Colorado/23-000403-001/2023|EPI_ISL_19605769|A_/_H5N1|Original||2.3.4.4b|01.01.2023||||12.12.2024|USDA_APHIS_Veterinary_Services_Diagnostic_Virology_Laboratory_National_Veterinary_Services_Laboratories||HA|4|A/red_tailed_hawk/Colo"/>
NNN
</sequence>
<sequence>
<taxon idref="A/red_tailed_hawk/Colorado/23-000494-001/2023|EPI_ISL_19605770|A_/_H5N1|Original||2.3.4.4b|04.01.2023||||12.12.2024|USDA_APHIS_Veterinary_Services_Diagnostic_Virology_Laboratory_National_Veterinary_Services_Laboratories||HA|4|A/red_tailed_hawk/Colo"/>
NNN
</sequence>
<sequence>
<taxon idref="A/red_tailed_hawk/Colorado/23-000498-001/2023|EPI_ISL_19605771|A_/_H5N1|Original||2.3.4.4b|04.01.2023||||12.12.2024|USDA_APHIS_Veterinary_Services_Diagnostic_Virology_Laboratory_National_Veterinary_Services_Laboratories||HA|4|A/red_tailed_hawk/Colo"/>
NNN
</sequence>
<sequence>
<taxon idref="A/red_tailed_hawk/Colorado/23-001466-001/2023|EPI_ISL_19605772|A_/_H5N1|Original||2.3.4.4b|05.01.2023||||12.12.2024|USDA_APHIS_Veterinary_Services_Diagnostic_Virology_Laboratory_National_Veterinary_Services_Laboratories||HA|4|A/red_tailed_hawk/Colo"/>
NNN
</sequence>
<sequence>
<taxon idref="A/red_tailed_hawk/Colorado/23-001466-002/2023|EPI_ISL_19605773|A_/_H5N1|Original||2.3.4.4b|05.01.2023||||12.12.2024|USDA_APHIS_Veterinary_Services_Diagnostic_Virology_Laboratory_National_Veterinary_Services_Laboratories||HA|4|A/red_tailed_hawk/Colo"/>
NNN
</sequence>
<sequence>
<taxon idref="A/red_tailed_hawk/Colorado/23-001574-001/2023|EPI_ISL_19605775|A_/_H5N1|Original||2.3.4.4b|10.01.2023||||12.12.2024|USDA_APHIS_Veterinary_Services_Diagnostic_Virology_Laboratory_National_Veterinary_Services_Laboratories||HA|4|A/red_tailed_hawk/Colo"/>
NNN
</sequence>
<sequence>
<taxon idref="A/red_tailed_hawk/Colorado/23-001576-001/2023|EPI_ISL_19605725|A_/_H5N1|Original||2.3.4.4b|10.01.2023||||12.12.2

024|USDA_APHIS_Veterinary_Services_Diagnostic_Virology_Laboratory_National_
Veterinary_Services_Laboratories||HA|4|A/red_tailed_hawk/Colo"/>
NNN
</sequence>
<sequence>
<taxon idref="A/red_tailed_hawk/Colorado/23-002236-
001/2023|EPI_ISL_19605724|A_/_H5N1|Original||2.3.4.4b|11.01.2023||||12.12.2
024|USDA_APHIS_Veterinary_Services_Diagnostic_Virology_Laboratory_National_
Veterinary_Services_Laboratories||HA|4|A/red_tailed_hawk/Colo"/>
NNN
</sequence>
<sequence>
<taxon idref="A/red_tailed_hawk/Colorado/23-002918-
002/2023|EPI_ISL_19605723|A_/_H5N1|Original||2.3.4.4b|21.01.2023||||12.12.2
024|USDA_APHIS_Veterinary_Services_Diagnostic_Virology_Laboratory_National_
Veterinary_Services_Laboratories||HA|4|A/red_tailed_hawk/Colo"/>
NNN
</sequence>
<sequence>
<taxon idref="A/red_tailed_hawk/Colorado/23-003934-
003/2023|EPI_ISL_19605711|A_/_H5N1|Original||2.3.4.4b|17.01.2023||||12.12.2
024|USDA_APHIS_Veterinary_Services_Diagnostic_Virology_Laboratory_National_
Veterinary_Services_Laboratories||HA|4|A/red_tailed_hawk/Colo"/>
NNN
</sequence>
<sequence>
<taxon idref="A/red_tailed_hawk/Colorado/23-004223-
001/2023|EPI_ISL_19605701|A_/_H5N1|Original||2.3.4.4b|31.01.2023||||12.12.2
024|USDA_APHIS_Veterinary_Services_Diagnostic_Virology_Laboratory_National_
Veterinary_Services_Laboratories||HA|4|A/red_tailed_hawk/Colo"/>
NNN
</sequence>
<sequence>
<taxon idref="A/red_tailed_hawk/Colorado/23-004224-
001/2023|EPI_ISL_19605702|A_/_H5N1|Original||2.3.4.4b|31.01.2023||||12.12.2
024|USDA_APHIS_Veterinary_Services_Diagnostic_Virology_Laboratory_National_
Veterinary_Services_Laboratories||HA|4|A/red_tailed_hawk/Colo"/>
NNN
</sequence>
<sequence>
<taxon idref="A/red_tailed_hawk/Missouri/23-001518-
001/2022|EPI_ISL_19605823|A_/_H5N1|Original||2.3.4.4b|21.12.2022||||12.12.2
024|USDA_APHIS_Veterinary_Services_Diagnostic_Virology_Laboratory_National_
Veterinary_Services_Laboratories||HA|4|A/red_tailed_hawk/Miss"/>
NNN
</sequence>
<sequence>
<taxon idref="A/red_tailed_hawk/Montana/23-002509-
001/2023|EPI_ISL_19605805|A_/_H5N1|Original||2.3.4.4b|18.01.2023||||12.12.2
024|USDA_APHIS_Veterinary_Services_Diagnostic_Virology_Laboratory_National_
Veterinary_Services_Laboratories||HA|4|A/red_tailed_hawk/Monta"/>
NNN
</sequence>
<sequence>
<taxon idref="A/red_tailed_hawk/Oregon/22-040812-
001/2022|EPI_ISL_19605593|A_/_H5N1|Original||2.3.4.4b|13.12.2022||||12.12.2
024|USDA_APHIS_Veterinary_Services_Diagnostic_Virology_Laboratory_National_
Veterinary_Services_Laboratories||HA|4|A/red_tailed_hawk/Oregon"/>
NNN
</sequence>
<sequence>

<taxon idref="A/red_tailed_hawk/Oregon/23-001496-001/2022|EPI_ISL_19605594|A_/_H5N1|Original||2.3.4.4b|28.12.2022||||12.12.2024|USDA_APHIS_Veterinary_Services_Diagnostic_Virology_Laboratory_National_Veterinary_Services_Laboratories||HA|4|A/red_tailed_hawk/Oregon"/>
NNN
</sequence>
<sequence>
<taxon idref="A/red_tailed_hawk/Oregon/23-003379-001/2023|EPI_ISL_19605596|A_/_H5N1|Original||2.3.4.4b|20.01.2023||||12.12.2024|USDA_APHIS_Veterinary_Services_Diagnostic_Virology_Laboratory_National_Veterinary_Services_Laboratories||HA|4|A/red_tailed_hawk/Oregon"/>
NNN
</sequence>
<sequence>
<taxon idref="A/red_tailed_hawk/Oregon/23-005360-001/2022|EPI_ISL_19605597|A_/_H5N1|Original||2.3.4.4b|15.12.2022||||12.12.2024|USDA_APHIS_Veterinary_Services_Diagnostic_Virology_Laboratory_National_Veterinary_Services_Laboratories||HA|4|A/red_tailed_hawk/Oregon"/>
NNN
</sequence>
<sequence>
<taxon idref="A/red_tailed_hawk/South_Dakota/23-002914-001/2023|EPI_ISL_19605605|A_/_H5N1|Original||2.3.4.4b|17.01.2023||||12.12.2024|USDA_APHIS_Veterinary_Services_Diagnostic_Virology_Laboratory_National_Veterinary_Services_Laboratories||HA|4|A/red_tailed_hawk/"/>
NNN
</sequence>
<sequence>
<taxon idref="A/red_tailed_hawk/Wyoming/22-042090-001/2022|EPI_ISL_19605483|A_/_H5N1|Original||2.3.4.4b|22.12.2022||||12.12.2024|USDA_APHIS_Veterinary_Services_Diagnostic_Virology_Laboratory_National_Veterinary_Services_Laboratories||HA|4|A/red_tailed_hawk/Wyomi"/>
NNN
</sequence>
<sequence>
<taxon idref="A/rosss_goose/Arkansas/23-001508-001/2023|EPI_ISL_19605678|A_/_H5N1|Original||2.3.4.4b|06.01.2023||||12.12.2024|USDA_APHIS_Veterinary_Services_Diagnostic_Virology_Laboratory_National_Veterinary_Services_Laboratories||HA|4|A/rosss_goose/Arkansas/23-"/>
NNN
</sequence>
<sequence>
<taxon idref="A/rosss_goose/California/23-000936-001/2022|EPI_ISL_19605681|A_/_H5N1|Original||2.3.4.4b|16.12.2022||||12.12.2024|USDA_APHIS_Veterinary_Services_Diagnostic_Virology_Laboratory_National_Veterinary_Services_Laboratories||HA|4|A/rosss_goose/California"/>
NNN
</sequence>
<sequence>
<taxon idref="A/rosss_goose/California/23-001856-002/2022|EPI_ISL_19605682|A_/_H5N1|Original||2.3.4.4b|13.12.2022||||12.12.2024|USDA_APHIS_Veterinary_Services_Diagnostic_Virology_Laboratory_National_Veterinary_Services_Laboratories||HA|4|A/rosss_goose/California"/>
NNN
</sequence>
<sequence>
<taxon idref="A/rosss_goose/Kansas/22-040832-001/2022|EPI_ISL_19605685|A_/_H5N1|Original||2.3.4.4b|07.12.2022||||12.12.2024|USDA_APHIS_Veterinary_Services_Diagnostic_Virology_Laboratory_National_Veterinary_Services_Laboratories||HA|4|A/rosss_goose/Kansas/22-0408"/>
NNN

</sequence>
<sequence>
<taxon idref="A/rosss_goose/Louisiana/23-000409-010/2022|EPI_ISL_19608589|A_/_H5N1|Original||2.3.4.4b|02.12.2022||||12.12.2024|USDA_APHIS_Veterinary_Services_Diagnostic_Virology_Laboratory_National_Veterinary_Services_Laboratories||HA|4|A/rosss_goose/Louisiana/2"/>
NNN
</sequence>
<sequence>
<taxon idref="A/rough-legged_hawk/BC/AIVPHL-352/2023|EPI_ISL_17051475|A_/_H5N1|Original||2.3.4.4b|03.01.2023|Russell_Shannon_Laurel|||28.02.2023|B.C._Centre_for_Disease_Control|British_Columbia_Centre_for_Disease_Control|HA|4|A/rough-legged_hawk/BC/AIVPHL-352/2023_"/>
NNN
</sequence>
<sequence>
<taxon idref="A/royal_tern/Argentina/CH-PD036/2023|EPI_ISL_19466181|A_/_H5N1|Original||2.3.4.4b|10.10.2023||||08.10.2024|Instituto_Nacional_de_Tecnologia_Agropecuaria_Instituto_de_Virologia_e_Innovaciones_Tecnologicas||HA|4|A/royal_tern/Argentina/CH-PD036/2023_HA|E"/>
NNN
</sequence>
<sequence>
<taxon idref="A/sanderling/California/23-005401-001/2023|EPI_ISL_17964856|A_/_H5N1|Original||2.3.4.4b|14.02.2023|Killian_Mary_Lea||23-005401-001|06.07.2023|National_Veterinary_Services_Laboratories_-_USDA|National_Veterinary_Services_Laboratories_-_USDA|HA|4|HA_A/s"/>
NNN
</sequence>
<sequence>
<taxon idref="A/sanderling/California/23-006644-001/2023|EPI_ISL_17964876|A_/_H5N1|Original||2.3.4.4b|14.02.2023|Killian_Mary_Lea||23-006644-001|06.07.2023|National_Veterinary_Services_Laboratories_-_USDA|National_Veterinary_Services_Laboratories_-_USDA|HA|4|HA_A/s"/>
NNN
</sequence>
<sequence>
<taxon idref="A/sanderling/Oregon/23-000771-001/2023|EPI_ISL_19605627|A_/_H5N1|Original||2.3.4.4b|03.01.2023||||12.12.2024|USDA_APHIS_Veterinary_Services_Diagnostic_Virology_Laboratory_National_Veterinary_Services_Laboratories||HA|4|A/sanderling/Oregon/23-000771"/>
NNN
</sequence>
<sequence>
<taxon idref="A/sanderling/Oregon/23-008234-001/2023|EPI_ISL_17964904|A_/_H5N1|Original||2.3.4.4b|04.02.2023|Killian_Mary_Lea||23-008234-001|06.07.2023|National_Veterinary_Services_Laboratories_-_USDA|National_Veterinary_Services_Laboratories_-_USDA|HA|4|HA_A/sande"/>
NNN
</sequence>
<sequence>
<taxon idref="A/sanderling/Oregon/23-011964-001/2023|EPI_ISL_17964941|A_/_H5N1|Original||2.3.4.4b|13.04.2023|Killian_Mary_Lea||23-011964-001|06.07.2023|National_Veterinary_Services_Laboratories_-_USDA|National_Veterinary_Services_Laboratories_-_USDA|HA|4|HA_A/sande"/>
NNN

</sequence>
<sequence>
<taxon idref="A/sanderling/Washington/23-004228-001/2023|EPI_ISL_19605618|A_/_H5N1|Original||2.3.4.4b|26.01.2023||||12.12.2024|USDA_APHIS_Veterinary_Services_Diagnostic_Virology_Laboratory_National_Veterinary_Services_Laboratories||HA|4|A/sanderling/Washington/2"/>
NNN
</sequence>
<sequence>
<taxon idref="A/sea_lion/Arica_y_Parinacota/240270-1/2023|EPI_ISL_17885976|A_/_H5N1|Original||2.3.4.4b|01.03.2023|GISAID_EpiFlu_Data_Curator|||19.07.2023|||HA|4|A/sea_lion/Arica_y_Parinacota/240270-1/2023_HA|EPI2609545|DNA_IN"/>
NNN
</sequence>
<sequence>
<taxon idref="A/sea_lion/Atacama/242444-1/2023|EPI_ISL_19391462|A_/_H5N1|Original||2.3.4.4b|13.03.2023||||09.09.2024|CEIRS_Data_Processing_and_Coordinating_Center_Center_for_Research_on_Influenza_Pathogenesis__CRIP_||HA|4|A/sea_lion/Atacama/242444-1/2023_HA|EPI3549"/>
NNN
</sequence>
<sequence>
<taxon idref="A/sea_lion/Biobio/SJCEIRR-2462961/2023|EPI_ISL_18690733|A_/_H5N1|Original||2.3.4.4b|26.03.2023||||26.12.2023|Emory_University__CEIRR_|Emory_University__CEIRR_|HA|4|A/sea_lion/Biobio/SJCEIRR-2462961/2023|EPI2868255|DNA_IN"/>
NNN
</sequence>
<sequence>
<taxon idref="A/sea_lion/Brazil/KU-3584/2023|EPI_ISL_18773644|A_/_H5N1|Original||2.3.4.4b|12.10.2023||||16.01.2024|Konkuk_University|Konkuk_University|HA|4|A/sea_lion/Brazil/KU-3584/2023_HA|EPI2919521|DNA_IN"/>
NNN
</sequence>
<sequence>
<taxon idref="A/sea_lion/Brazil/KU-3594/2023|EPI_ISL_18773660|A_/_H5N1|Original||2.3.4.4b|12.10.2023||||16.01.2024|Konkuk_University|Konkuk_University|HA|4|A/sea_lion/Brazil/KU-3594/2023_HA|EPI2919538|DNA_IN"/>
NNN
</sequence>
<sequence>
<taxon idref="A/sea_lion/Los_Lagos/247932/2023|EPI_ISL_19404920|A_/_H5N1|Original||2.3.4.4b|09.04.2023||||10.09.2024|Emory_University__CEIRR_||HA|4|A/sea_lion/Los_Lagos/247932/2023_HA|EPI3550220|DNA_IN"/>
NNN
</sequence>
<sequence>
<taxon idref="A/sea_lion/Peru/AQP-SER00B/2023|EPI_ISL_18054508|A_/_H5N1|Original||2.3.4.4b|25.01.2023|GISAID_EpiFlu_Data_Curator|||01.08.2023|||HA|4|A/sea_lion/Peru/AQP-SER00B/2023_HA|EPI2664262|DNA_IN"/>
NNN
</sequence>
<sequence>
<taxon idref="A/sea_lion/Peru/AQP-SER00K/2023|EPI_ISL_18054509|A_/_H5N1|Original||2.3.4.4b|07.02.2023|GISAID_

EpiFlu_Data_Curator|||01.08.2023|||HA|4|A/sea_lion/Peru/AQP-
SER00K/2023_HA|EPI2664269|DNA_IN"/>
NNN
</sequence>
<sequence>
<taxon idref="A/sea_lion/Peru/AQP-
SER00R/2023|EPI_ISL_18054510|A_/_H5N1|Original||2.3.4.4b|06.03.2023|GISAID_
EpiFlu_Data_Curator|||01.08.2023|||HA|4|A/sea_lion/Peru/AQP-
SER00R/2023_HA|EPI2664277|DNA_IN"/>
NNN
</sequence>
<sequence>
<taxon idref="A/sea_lion/Peru/TAC-INS-
010/2023|EPI_ISL_17777531|A_/_H5N1|Original||2.3.4.4b|07.03.2023|Padilla_Ro
jas_Carlos_Patricio||1375|01.07.2023|Laboratorio_de_Virus_Respiratorios_Cen
tro_Nacional_de_Salud_Publica|Instituto_Nacional_de_Salud_Peru|HA|4|Peru_IN
S"/>
NNN
</sequence>
<sequence>
<taxon idref="A/sea_lion/Peru/TAC-INS-
011/2023|EPI_ISL_17777532|A_/_H5N1|Original||2.3.4.4b|07.03.2023|Padilla_Ro
jas_Carlos_Patricio||1375|01.07.2023|Laboratorio_de_Virus_Respiratorios_Cen
tro_Nacional_de_Salud_Publica|Instituto_Nacional_de_Salud_Peru|HA|4|Peru_IN
S"/>
NNN
</sequence>
<sequence>
<taxon idref="A/sea_lion/Tarapaca/240524-
2/2023|EPI_ISL_17885975|A_/_H5N1|Original||2.3.4.4b|07.03.2023|GISAID_EpiFl
u_Data_Curator|||19.07.2023|||HA|4|A/sea_lion/Tarapaca/240524-
2/2023_HA|EPI2609536|DNA_IN"/>
NNN
</sequence>
<sequence>
<taxon idref="A/sea_lion/Tarapaca/245719-
10/2023|EPI_ISL_19410279|A_/_H5N1|Original||2.3.4.4b|30.03.2023||||12.09.20
24|Emory_University__CEIRR_||HA|4|A/sea_lion/Tarapaca/245719-
10/2023_HA|EPI3555288|DNA_IN"/>
NNN
</sequence>
<sequence>
<taxon idref="A/sea_lion/Valparaiso/SJCEIRR-
2431361/2023|EPI_ISL_18760069|A_/_H5N1|Original||2.3.4.4b|19.03.2023||||14.
01.2024|Emory_University__CEIRR_|Emory_University__CEIRR_|HA|4|A/sea_lion/V
alparaiso/SJCEIRR-2431361/2023|EPI2913244|DNA_IN"/>
NNN
</sequence>
<sequence>
<taxon idref="A/seabird/Uruguay/P16_14926/2023|EPI_ISL_19070494|A_/_H5N1|Or
iginal||2.3.4.4b|14.09.2023||||24.04.2024|Universidad_de_la_Republica_Facul
tad_de_Ciencias_Genetica_Evolutiva/Division_de_Laboratorios_Veterinarios_Pl
ataforma_Genomica||HA|4|A/seabird/Urug"/>
NNN
</sequence>
<sequence>
<taxon idref="A/seabird/Uruguay/P23_41023/2023|EPI_ISL_19070495|A_/_H5N1|Or
iginal||2.3.4.4b|04.10.2023||||24.04.2024|Universidad_de_la_Republica_Facul
tad_de_Ciencias_Genetica_Evolutiva/Division_de_Laboratorios_Veterinarios_Pl
ataforma_Genomica||HA|4|A/seabird/Urug"/>
NNN

</sequence>
<sequence>
<taxon idref="A/seabird/Uruguay/P24_41023/2023|EPI_ISL_19070496|A_/_H5N1|Original||2.3.4.4b|04.10.2023||||24.04.2024|Universidad_de_la_Republica_Facultad_de_Ciencias_Genetica_Evolutiva/Division_de_Laboratorios_Veterinarios_Plataforma_Genomica||HA|4|A/seabird/Urug"/>
NNN
</sequence>
<sequence>
<taxon idref="A/seabird/Uruguay/P25_41023/2023|EPI_ISL_19070497|A_/_H5N1|Original||2.3.4.4b|04.10.2023||||24.04.2024|Universidad_de_la_Republica_Facultad_de_Ciencias_Genetica_Evolutiva/Division_de_Laboratorios_Veterinarios_Plataforma_Genomica||HA|4|A/seabird/Urug"/>
NNN
</sequence>
<sequence>
<taxon idref="A/snow_goose/Alaska/22-041764-001/2022|EPI_ISL_19605880|A_/_H5N1|Original||2.3.4.4b|20.12.2022||||12.12.2024|USDA_APHIS_Veterinary_Services_Diagnostic_Virology_Laboratory_National_Veterinary_Services_Laboratories||HA|4|A/snow_goose/Alaska/22-041764"/>
NNN
</sequence>
<sequence>
<taxon idref="A/snow_goose/Arkansas/23-001513-001/2023|EPI_ISL_19605884|A_/_H5N1|Original||2.3.4.4b|06.01.2023||||12.12.2024|USDA_APHIS_Veterinary_Services_Diagnostic_Virology_Laboratory_National_Veterinary_Services_Laboratories||HA|4|A/snow_goose/Arkansas/23-00"/>
NNN
</sequence>
<sequence>
<taxon idref="A/snow_goose/Arkansas/23-001514-001/2023|EPI_ISL_19605885|A_/_H5N1|Original||2.3.4.4b|06.01.2023||||12.12.2024|USDA_APHIS_Veterinary_Services_Diagnostic_Virology_Laboratory_National_Veterinary_Services_Laboratories||HA|4|A/snow_goose/Arkansas/23-00"/>
NNN
</sequence>
<sequence>
<taxon idref="A/snow_goose/BC/AIVPHL-337/2022|EPI_ISL_17051466|A_/_H5N1|Original||2.3.4.4b|19.12.2022|Russell_Shannon_Laurel|||28.02.2023|B.C._Centre_for_Disease_Control|British_Columbia_Centre_for_Disease_Control|HA|4|A/snow_goose/BC/AIVPHL-337/2022_HA|EPI2429175|"/>
NNN
</sequence>
<sequence>
<taxon idref="A/snow_goose/BC/AIVPHL-340/2022|EPI_ISL_17051461|A_/_H5N1|Original||2.3.4.4b|15.12.2022|Russell_Shannon_Laurel|||28.02.2023|B.C._Centre_for_Disease_Control|British_Columbia_Centre_for_Disease_Control|HA|4|A/snow_goose/BC/AIVPHL-340/2022_HA|EPI2429142|"/>
NNN
</sequence>
<sequence>
<taxon idref="A/snow_goose/California/22-041398-001/2022|EPI_ISL_19605887|A_/_H5N1|Original||2.3.4.4b|01.12.2022||||12.12.2024|USDA_APHIS_Veterinary_Services_Diagnostic_Virology_Laboratory_National_Veterinary_Services_Laboratories||HA|4|A/snow_goose/California/2"/>
NNN
</sequence>
<sequence>

<taxon idref="A/snow_goose/California/22-041400-
002/2022|EPI_ISL_19605888|A_/_H5N1|Original||2.3.4.4b|01.12.2022||||12.12.2
024|USDA_APHIS_Veterinary_Services_Diagnostic_Virology_Laboratory_National_
Veterinary_Services_Laboratories||HA|4|A/snow_goose/California/2"/>
NNN
</sequence>
<sequence>
<taxon idref="A/snow_goose/California/22-041403-
001/2022|EPI_ISL_19605889|A_/_H5N1|Original||2.3.4.4b|01.12.2022||||12.12.2
024|USDA_APHIS_Veterinary_Services_Diagnostic_Virology_Laboratory_National_
Veterinary_Services_Laboratories||HA|4|A/snow_goose/California/2"/>
NNN
</sequence>
<sequence>
<taxon idref="A/snow_goose/California/22-041403-
002/2022|EPI_ISL_19605890|A_/_H5N1|Original||2.3.4.4b|01.12.2022||||12.12.2
024|USDA_APHIS_Veterinary_Services_Diagnostic_Virology_Laboratory_National_
Veterinary_Services_Laboratories||HA|4|A/snow_goose/California/2"/>
NNN
</sequence>
<sequence>
<taxon idref="A/snow_goose/California/22-041403-
003/2022|EPI_ISL_19605891|A_/_H5N1|Original||2.3.4.4b|01.12.2022||||12.12.2
024|USDA_APHIS_Veterinary_Services_Diagnostic_Virology_Laboratory_National_
Veterinary_Services_Laboratories||HA|4|A/snow_goose/California/2"/>
NNN
</sequence>
<sequence>
<taxon idref="A/snow_goose/California/22-041408-
001/2022|EPI_ISL_19605893|A_/_H5N1|Original||2.3.4.4b|01.12.2022||||12.12.2
024|USDA_APHIS_Veterinary_Services_Diagnostic_Virology_Laboratory_National_
Veterinary_Services_Laboratories||HA|4|A/snow_goose/California/2"/>
NNN
</sequence>
<sequence>
<taxon idref="A/snow_goose/California/22-041409-
001/2022|EPI_ISL_19605894|A_/_H5N1|Original||2.3.4.4b|01.12.2022||||12.12.2
024|USDA_APHIS_Veterinary_Services_Diagnostic_Virology_Laboratory_National_
Veterinary_Services_Laboratories||HA|4|A/snow_goose/California/2"/>
NNN
</sequence>
<sequence>
<taxon idref="A/snow_goose/California/22-042007-
002/2022|EPI_ISL_19605896|A_/_H5N1|Original||2.3.4.4b|14.12.2022||||12.12.2
024|USDA_APHIS_Veterinary_Services_Diagnostic_Virology_Laboratory_National_
Veterinary_Services_Laboratories||HA|4|A/snow_goose/California/2"/>
NNN
</sequence>
<sequence>
<taxon idref="A/snow_goose/California/22-042007-
003/2022|EPI_ISL_19605897|A_/_H5N1|Original||2.3.4.4b|14.12.2022||||12.12.2
024|USDA_APHIS_Veterinary_Services_Diagnostic_Virology_Laboratory_National_
Veterinary_Services_Laboratories||HA|4|A/snow_goose/California/2"/>
NNN
</sequence>
<sequence>
<taxon idref="A/snow_goose/California/22-042007-
004/2022|EPI_ISL_19605898|A_/_H5N1|Original||2.3.4.4b|14.12.2022||||12.12.2
024|USDA_APHIS_Veterinary_Services_Diagnostic_Virology_Laboratory_National_
Veterinary_Services_Laboratories||HA|4|A/snow_goose/California/2"/>
NNN

</sequence>
<sequence>
<taxon idref="A/snow_goose/California/23-000936-002/2022|EPI_ISL_19605899|A_/_H5N1|Original||2.3.4.4b|16.12.2022||||12.12.2024|USDA_APHIS_Veterinary_Services_Diagnostic_Virology_Laboratory_National_Veterinary_Services_Laboratories||HA|4|A/snow_goose/California/2"/>
NNN
</sequence>
<sequence>
<taxon idref="A/snow_goose/California/23-000950-001/2022|EPI_ISL_19605877|A_/_H5N1|Original||2.3.4.4b|19.12.2022||||12.12.2024|USDA_APHIS_Veterinary_Services_Diagnostic_Virology_Laboratory_National_Veterinary_Services_Laboratories||HA|4|A/snow_goose/California/2"/>
NNN
</sequence>
<sequence>
<taxon idref="A/snow_goose/California/23-002594-003/2023|EPI_ISL_19605875|A_/_H5N1|Original||2.3.4.4b|10.01.2023||||12.12.2024|USDA_APHIS_Veterinary_Services_Diagnostic_Virology_Laboratory_National_Veterinary_Services_Laboratories||HA|4|A/snow_goose/California/2"/>
NNN
</sequence>
<sequence>
<taxon idref="A/snow_goose/California/23-004358-002/2023|EPI_ISL_19605862|A_/_H5N1|Original||2.3.4.4b|25.01.2023||||12.12.2024|USDA_APHIS_Veterinary_Services_Diagnostic_Virology_Laboratory_National_Veterinary_Services_Laboratories||HA|4|A/snow_goose/California/2"/>
NNN
</sequence>
<sequence>
<taxon idref="A/snow_goose/Kansas/22-041130-003/2022|EPI_ISL_19605948|A_/_H5N1|Original||2.3.4.4b|06.12.2022||||12.12.2024|USDA_APHIS_Veterinary_Services_Diagnostic_Virology_Laboratory_National_Veterinary_Services_Laboratories||HA|4|A/snow_goose/Kansas/22-041130"/>
NNN
</sequence>
<sequence>
<taxon idref="A/snow_goose/Louisiana/23-000409-016/2022|EPI_ISL_19608599|A_/_H5N1|Original||2.3.4.4b|02.12.2022||||12.12.2024|USDA_APHIS_Veterinary_Services_Diagnostic_Virology_Laboratory_National_Veterinary_Services_Laboratories||HA|4|A/snow_goose/Louisiana/23-"/>
NNN
</sequence>
<sequence>
<taxon idref="A/snow_goose/Louisiana/23-000409-018/2022|EPI_ISL_19608600|A_/_H5N1|Original||2.3.4.4b|02.12.2022||||12.12.2024|USDA_APHIS_Veterinary_Services_Diagnostic_Virology_Laboratory_National_Veterinary_Services_Laboratories||HA|4|A/snow_goose/Louisiana/23-"/>
NNN
</sequence>
<sequence>
<taxon idref="A/snow_goose/Missouri/22-040427-002/2022|EPI_ISL_19605947|A_/_H5N1|Original||2.3.4.4b|01.12.2022||||12.12.2024|USDA_APHIS_Veterinary_Services_Diagnostic_Virology_Laboratory_National_Veterinary_Services_Laboratories||HA|4|A/snow_goose/Missouri/22-04"/>
NNN
</sequence>
<sequence>
<taxon idref="A/snow_goose/Missouri/22-040786-001/2022|EPI_ISL_19605928|A_/_H5N1|Original||2.3.4.4b|08.12.2022||||12.12.2

024|USDA_APHIS_Veterinary_Services_Diagnostic_Virology_Laboratory_National_Veterinary_Services_Laboratories||HA|4|A/snow_goose/Missouri/22-04"/>
NNN
</sequence>
<sequence>
<taxon idref="A/snow_goose/Washington/22-041983-001/2022|EPI_ISL_19605053|A_/_H5N1|Original||2.3.4.4b|16.12.2022||||12.12.2024|USDA_APHIS_Veterinary_Services_Diagnostic_Virology_Laboratory_National_Veterinary_Services_Laboratories||HA|4|A/snow_goose/Washington/2"/>
NNN
</sequence>
<sequence>
<taxon idref="A/southern_elephant_seal/Argentina/CH-PM053/2023|EPI_ISL_19466252|A_/_H5N1|Original||2.3.4.4b|01.11.2023||||08.10.2024|Instituto_Nacional_de_Tecnologia_Agropecuaria_Instituto_de_Virologia_e_Innovaciones_Tecnologicas||HA|4|A/southern_elephant_seal/Arge"/>
NNN
</sequence>
<sequence>
<taxon idref="A/striped_skunk/Kansas/W23-094/2023|EPI_ISL_17424646|A_/_H5N1|||2.3.4.4b|27.01.2023||||08.04.2023|St._Jude_Center_of_Excellence_for_Influenza_Research_and_Response_-_CEIRR_Influenza_Data_Processing_and_Communication_Center|St._Jude_Center_of_Excellence"/>
NNN
</sequence>
<sequence>
<taxon idref="A/swainsons_hawk/Colorado/23-004704-001/2023|EPI_ISL_19605066|A_/_H5N1|Original||2.3.4.4b|30.01.2023||||12.12.2024|USDA_APHIS_Veterinary_Services_Diagnostic_Virology_Laboratory_National_Veterinary_Services_Laboratories||HA|4|A/swainsons_hawk/Colora"/>
NNN
</sequence>
<sequence>
<taxon idref="A/swan/Nebraska/22-041037-001/2022|EPI_ISL_19605145|A_/_H5N1|Original||2.3.4.4b|15.12.2022||||12.12.2024|USDA_APHIS_Veterinary_Services_Diagnostic_Virology_Laboratory_National_Veterinary_Services_Laboratories||HA|4|A/swan/Nebraska/22-041037-001/202"/>
NNN
</sequence>
<sequence>
<taxon idref="A/swan/Nebraska/22-041037-002/2022|EPI_ISL_19605146|A_/_H5N1|Original||2.3.4.4b|15.12.2022||||12.12.2024|USDA_APHIS_Veterinary_Services_Diagnostic_Virology_Laboratory_National_Veterinary_Services_Laboratories||HA|4|A/swan/Nebraska/22-041037-002/202"/>
NNN
</sequence>
<sequence>
<taxon idref="A/tern/Espirito_Santo/MAPA-1339N2/2023|EPI_ISL_18755338|A_/_H5N1|Original||2.3.4.4b|15.05.2023||||12.01.2024|Ministerio_da_Agricultura_e_Pecuaria|Ministerio_da_Agricultura_e_Pecuaria|HA|4|A/tern/Espirito_Santo/MAPA-1339N2/2023_HA|EPI2908529|DNA_IN"/>
NNN
</sequence>
<sequence>
<taxon idref="A/trumpeter_swan/BC/AIVPHL-320/2022|EPI_ISL_17051458|A_/_H5N1|Original||2.3.4.4b|12.12.2022|Russell_Shannon_Laurel|||28.02.2023|B.C._Centre_for_Disease_Control|British_Columbia_Centre_for_Disease_Control|HA|4|A/trumpeter_swan/BC/AIVPHL-320/2022_HA|EPI"/>
NNN

</sequence>
<sequence>
<taxon idref="A/trumpeter_swan/Iowa/23-002039-
001/2023|EPI_ISL_19605154|A_/_H5N1|Original||2.3.4.4b|12.01.2023||||12.12.2
024|USDA_APHIS_Veterinary_Services_Diagnostic_Virology_Laboratory_National_
Veterinary_Services_Laboratories||HA|4|A/trumpeter_swan/Iowa/23-00"/>
NNN
</sequence>
<sequence>
<taxon idref="A/trumpeter_swan/Iowa/23-002039-
002/2023|EPI_ISL_19605155|A_/_H5N1|Original||2.3.4.4b|12.01.2023||||12.12.2
024|USDA_APHIS_Veterinary_Services_Diagnostic_Virology_Laboratory_National_
Veterinary_Services_Laboratories||HA|4|A/trumpeter_swan/Iowa/23-00"/>
NNN
</sequence>
<sequence>
<taxon idref="A/trumpeter_swan/Iowa/23-002662-
002/2023|EPI_ISL_19605156|A_/_H5N1|Original||2.3.4.4b|21.01.2023||||12.12.2
024|USDA_APHIS_Veterinary_Services_Diagnostic_Virology_Laboratory_National_
Veterinary_Services_Laboratories||HA|4|A/trumpeter_swan/Iowa/23-00"/>
NNN
</sequence>
<sequence>
<taxon idref="A/trumpeter_swan/Missouri/22-040427-
001/2022|EPI_ISL_19605137|A_/_H5N1|Original||2.3.4.4b|01.12.2022||||12.12.2
024|USDA_APHIS_Veterinary_Services_Diagnostic_Virology_Laboratory_National_
Veterinary_Services_Laboratories||HA|4|A/trumpeter_swan/Missou"/>
NNN
</sequence>
<sequence>
<taxon idref="A/turkey/Araucania/241892-
3/2023|EPI_ISL_17885966|A_/_H5N1|Original||2.3.4.4b|14.03.2023|GISAID_EpiFl
u_Data_Curator|||24.07.2023|||HA|4|A/turkey/Araucania/241892-
3/2023_HA|EPI2609482|DNA_IN"/>
NNN
</sequence>
<sequence>
<taxon idref="A/turkey/Nuble/240489-
1/2023|EPI_ISL_17885964|A_/_H5N1|Original||2.3.4.4b|07.03.2023|GISAID_EpiFl
u_Data_Curator|||19.07.2023|||HA|4|A/turkey/Nuble/240489-
1/2023_HA|EPI2609474|DNA_IN"/>
NNN
</sequence>
<sequence>
<taxon idref="A/turkey/Nuble/241568-
1/2023|EPI_ISL_17885962|A_/_H5N1|Original||2.3.4.4b|10.03.2023|GISAID_EpiFl
u_Data_Curator|||19.07.2023|||HA|4|A/turkey/Nuble/241568-
1/2023_HA|EPI2609462|DNA_IN"/>
NNN
</sequence>
<sequence>
<taxon idref="A/turkey/Valparaiso/245562-
1/2023|EPI_ISL_18760063|A_/_H5N1|Original||2.3.4.4b|29.03.2023||||14.01.202
4|Emory_University__CEIRR_|Emory_University__CEIRR_|HA|4|A/turkey/Valparais
o/245562-1/2023_HA|EPI2913205|DNA_IN"/>
NNN
</sequence>
<sequence>
<taxon idref="A/turkey/Valparaiso/245886-
1/2023|EPI_ISL_19410284|A_/_H5N1|Original||2.3.4.4b|30.03.2023||||13.09.202

4|Emory_University__CEIRR_||HA|4|A/turkey/Valparaiso/245886-
1/2023_HA|EPI3555321|DNA_IN"/>
NNN
</sequence>
<sequence>
<taxon idref="A/turkey/Valparaiso/245886-
3/2023|EPI_ISL_19410285|A_/_H5N1|Original||2.3.4.4b|30.03.2023||||13.09.202
4|Emory_University__CEIRR_||HA|4|A/turkey/Valparaiso/245886-
3/2023_HA|EPI3555328|DNA_IN"/>
NNN
</sequence>
<sequence>
<taxon idref="A/turkey/Valparaiso/245886-
5/2023|EPI_ISL_19410286|A_/_H5N1|Original||2.3.4.4b|30.03.2023||||13.09.202
4|Emory_University__CEIRR_||HA|4|A/turkey/Valparaiso/245886-
5/2023_HA|EPI3555335|DNA_IN"/>
NNN
</sequence>
<sequence>
<taxon idref="A/turkey/Valparaiso/248976-
12/2023|EPI_ISL_19404902|A_/_H5N1|Original||2.3.4.4b|13.04.2023||||13.09.20
24|Emory_University__CEIRR_||HA|4|A/turkey/Valparaiso/248976-
12/2023_HA|EPI3550180|DNA_IN"/>
NNN
</sequence>
<sequence>
<taxon idref="A/turkey/Valparaiso/248976-
13/2023|EPI_ISL_19404939|A_/_H5N1|Original||2.3.4.4b|13.04.2023||||13.09.20
24|Emory_University__CEIRR_||HA|4|A/turkey/Valparaiso/248976-
13/2023_HA|EPI3550235|DNA_IN"/>
NNN
</sequence>
<sequence>
<taxon idref="A/turkey_vulture/California/22-041407-
001/2022|EPI_ISL_19605121|A_/_H5N1|Original||2.3.4.4b|01.12.2022||||12.12.2
024|USDA_APHIS_Veterinary_Services_Diagnostic_Virology_Laboratory_National_
Veterinary_Services_Laboratories||HA|4|A/turkey_vulture/Cali"/>
NNN
</sequence>
<sequence>
<taxon idref="A/turkey_vulture/California/22-041409-
003/2022|EPI_ISL_19605134|A_/_H5N1|Original||2.3.4.4b|01.12.2022||||12.12.2
024|USDA_APHIS_Veterinary_Services_Diagnostic_Virology_Laboratory_National_
Veterinary_Services_Laboratories||HA|4|A/turkey_vulture/Cali"/>
NNN
</sequence>
<sequence>
<taxon idref="A/turkey_vulture/California/23-000283-
001/2022|EPI_ISL_19605126|A_/_H5N1|Original||2.3.4.4b|14.12.2022||||12.12.2
024|USDA_APHIS_Veterinary_Services_Diagnostic_Virology_Laboratory_National_
Veterinary_Services_Laboratories||HA|4|A/turkey_vulture/Cali"/>
NNN
</sequence>
<sequence>
<taxon idref="A/turkey_vulture/California/23-002030-
001/2022|EPI_ISL_19605130|A_/_H5N1|Original||2.3.4.4b|29.12.2022||||12.12.2
024|USDA_APHIS_Veterinary_Services_Diagnostic_Virology_Laboratory_National_
Veterinary_Services_Laboratories||HA|4|A/turkey_vulture/Cali"/>
NNN
</sequence>
<sequence>

<taxon idref="A/turkey_vulture/California/23-002601-
001/2023|EPI_ISL_19605131|A_/_H5N1|Original||2.3.4.4b|11.01.2023||||12.12.2
024|USDA_APHIS_Veterinary_Services_Diagnostic_Virology_Laboratory_National_
Veterinary_Services_Laboratories||HA|4|A/turkey_vulture/Cali"/>
NNN
</sequence>
<sequence>
<taxon idref="A/turkey_vulture/California/23-004361-
001/2023|EPI_ISL_19605132|A_/_H5N1|Original||2.3.4.4b|25.01.2023||||12.12.2
024|USDA_APHIS_Veterinary_Services_Diagnostic_Virology_Laboratory_National_
Veterinary_Services_Laboratories||HA|4|A/turkey_vulture/Cali"/>
NNN
</sequence>
<sequence>
<taxon idref="A/turkey_vulture/California/23-016941-
001/2023|EPI_ISL_17964980|A_/_H5N1|Original||2.3.4.4b|23.05.2023|Killian_Ma
ry_Lea||23-016941-
001|06.07.2023|National_Veterinary_Services_Laboratories_-
_USDA|National_Veterinary_Services_Laboratories_-_USDA|HA|4|HA"/>
NNN
</sequence>
<sequence>
<taxon idref="A/turkey_vulture/Colorado/23-011447-
001/2023|EPI_ISL_17964934|A_/_H5N1|Original||2.3.4.4b|10.04.2023|Killian_Ma
ry_Lea||23-011447-
001|06.07.2023|National_Veterinary_Services_Laboratories_-
_USDA|National_Veterinary_Services_Laboratories_-_USDA|HA|4|HA_A"/>
NNN
</sequence>
<sequence>
<taxon idref="A/turkey_vulture/Utah/23-012747-
001/2023|EPI_ISL_17964947|A_/_H5N1|Original||2.3.4.4b|19.04.2023|Killian_Ma
ry_Lea||23-012747-
001|06.07.2023|National_Veterinary_Services_Laboratories_-
_USDA|National_Veterinary_Services_Laboratories_-_USDA|HA|4|HA_A/tur"/>
NNN
</sequence>
<sequence>
<taxon idref="A/turkey_vulture/Utah/23-014335-
001/2023|EPI_ISL_17964961|A_/_H5N1|Original||2.3.4.4b|28.04.2023|Killian_Ma
ry_Lea||23-014335-
001|06.07.2023|National_Veterinary_Services_Laboratories_-
_USDA|National_Veterinary_Services_Laboratories_-_USDA|HA|4|HA_A/tur"/>
NNN
</sequence>
<sequence>
<taxon idref="A/turkey_vulture/Wyoming/23-012565-
001/2023|EPI_ISL_17964946|A_/_H5N1|Original||2.3.4.4b|19.04.2023|Killian_Ma
ry_Lea||23-012565-
001|06.07.2023|National_Veterinary_Services_Laboratories_-
_USDA|National_Veterinary_Services_Laboratories_-_USDA|HA|4|HA_A/"/>
NNN
</sequence>
<sequence>
<taxon idref="A/turkey_vulture/Wyoming/23-013715-
001/2023|EPI_ISL_17964953|A_/_H5N1|Original||2.3.4.4b|19.04.2023|Killian_Ma
ry_Lea||23-013715-
001|06.07.2023|National_Veterinary_Services_Laboratories_-
_USDA|National_Veterinary_Services_Laboratories_-_USDA|HA|4|HA_A/"/>
NNN
</sequence>

<sequence>
<taxon idref="A/western_grebe/California/23-000276-001/2022|EPI_ISL_19605204|A_/_H5N1|Original||2.3.4.4b|15.12.2022||||12.12.2024|USDA_APHIS_Veterinary_Services_Diagnostic_Virology_Laboratory_National_Veterinary_Services_Laboratories||HA|4|A/western_grebe/Califo"/>
NNN
</sequence>
<sequence>
<taxon idref="A/whimbrel/Coquimbo/239964/2023|EPI_ISL_17885960|A_/_H5N1|Original||2.3.4.4b|05.03.2023|GISAID_EpiFlu_Data_Curator|||19.07.2023|||HA|4|A/whimbrel/Coquimbo/239964/2023_HA|EPI2609454|DNA_IN"/>
NNN
</sequence>
<sequence>
<taxon idref="A/wildbird-Fregata-magnificens/Ecuador/IC03-4587/2023|EPI_ISL_17973443|A_/_H5N1|Original||2.3.4.4b|11.01.2023|Carrazco_Andres|IC03|4587|01.11.2023|Ministerio_del_Ambiente_Agua_y_Transicion_Ecologica__MAATE_|Instituto_Nacional_de_Investigacion_en_Salu"/>
NNN
</sequence>
<sequence>
<taxon idref="A/wildbird-Fregata-magnificens/Ecuador/IC06-4590/2023|EPI_ISL_17973458|A_/_H5N1|Original||2.3.4.4b|11.01.2023|Carrazco_Andres|IC06|4590|16.10.2023|Ministerio_del_Ambiente_Agua_y_Transicion_Ecologica__MAATE_|Instituto_Nacional_de_Investigacion_en_Salu"/>
NNN
</sequence>
<sequence>
<taxon idref="A/wood_duck/Colorado/23-000400-001/2022|EPI_ISL_19605216|A_/_H5N1|Original||2.3.4.4b|29.12.2022||||12.12.2024|USDA_APHIS_Veterinary_Services_Diagnostic_Virology_Laboratory_National_Veterinary_Services_Laboratories||HA|4|A/wood_duck/Colorado/23-0004"/>
NNN
</sequence>
<sequence>
<taxon idref="OQ547327.1|InfluenzaAvirus_A/Nannopterumbrasilianus/Peru/AISA0451/2022_H5N1__segment4hemagglutinin_HA_genecompletecds|2022-11-22|4|H5N1|Peru|Phalacrocoraxbrasilianus|Peru|Alphainfluenzavirus|Alphainfluenzavirusinfluenz"/>
NNN
</sequence>
<sequence>
<taxon idref="OQ547335.1|InfluenzaAvirus_A/Pelecanusthagus/Peru/AIS0541/2022_H5N1__segment4hemagglutinin_HA_genecompletecds|2022-11-16|4|H5N1|Peru|Pelecanusthagus|Peru|Alphainfluenzavirus|Alphainfluenzavirusinfluenz"/>
NNN
</sequence>
<sequence>
<taxon idref="OQ547343.1|InfluenzaAvirus_A/Gallusgallus/Peru/AIS0539/2022_H5N1__segment4hemagglutinin_HA_genecompletecds|2022-11-18|4|H5N1|Peru|Gallusgallus|Peru|Alphainfluenzavirus|Alphainfluenzavirusinfluenz"/>
NNN
</sequence>
<sequence>
<taxon idref="OQ547351.1|InfluenzaAvirus_A/Gallusgallus/Peru/AIS0540/2022_H5N1__segment4hemagglutinin_HA_genecompletecds|2022-11-28|4|H5N1|Peru|Gallusgallus|Peru|Alphainfluenzavirus|Alphainfluenzavirusinfluenz"/>
NNN

</sequence>
<sequence>
<taxon idref="OQ547423.1|InfluenzaAvirus_A/Nannopterumbrasilianus/Peru/AISA0446/2022_H5N1__segment4hemagglutinin_HA_genecompletecds|2022-11-22|4|H5N1|Peru|Phalacrocoraxbrasilianus|Peru|Alphainfluenzavirus|Alphainfluenzavirusinfluenz"/>
NNN
</sequence>
<sequence>
<taxon idref="OQ547439.1|InfluenzaAvirus_A/Pelecanusthagus/Peru/AIS0538/2022_H5N1__segment4hemagglutinin_HA_genecompletecds|2022-11-10|4|H5N1|Peru|Pelecanusthagus|Peru|Alphainfluenzavirus|Alphainfluenzavirusinfluenz"/>
NNN
</sequence>
<sequence>
<taxon idref="OQ547447.1|InfluenzaAvirus_A/Pelecanusthagus/Peru/AISA0464/2022_H5N1__segment4hemagglutinin_HA_genecompletecds|2022-11-22|4|H5N1|Peru|Pelecanusthagus|Peru|Alphainfluenzavirus|Alphainfluenzavirusinfluenz"/>
NNN
</sequence>
<sequence>
<taxon idref="OQ550426.1|InfluenzaAvirus_A/Guanaycormorant/Peru/PIU-SER024/2022_H5N1__segment4hemagglutinin_HA_genecompletecds|2022-11-24|4|H5N1|Peru||Peru|Alphainfluenzavirus|Alphainfluenzavirusinfluenz"/>
NNN
</sequence>
<sequence>
<taxon idref="OQ550434.1|InfluenzaAvirus_A/pelican/Peru/PIU-SER016/2022_H5N1__segment4hemagglutinin_HA_genecompletecds|2022-11-24|4|H5N1|Peru|Pelecanidae|Peru|Alphainfluenzavirus|Alphainfluenzavirusinfluenz"/>
NNN
</sequence>
<sequence>
<taxon idref="OQ550442.1|InfluenzaAvirus_A/dolphin/Peru/PIU-SER002/2022_H5N1__segment4hemagglutinin_HA_genecompletecds|2022-11-22|4|H5N1|Peru|Delphinusdelphis|Peru|Alphainfluenzavirus|Alphainfluenzavirusinfluenz"/>
NNN
</sequence>
<sequence>
<taxon idref="OQ550450.1|InfluenzaAvirus_A/pelican/Peru/PIU-SER019/2022_H5N1__segment4hemagglutinin_HA_genecompletecds|2022-11-24|4|H5N1|Peru|Pelecanidae|Peru|Alphainfluenzavirus|Alphainfluenzavirusinfluenz"/>
NNN
</sequence>
<sequence>
<taxon idref="OQ550458.1|InfluenzaAvirus_A/pelican/Peru/PIU-SER028/2022_H5N1__segment4hemagglutinin_HA_genecompletecds|2022-11-24|4|H5N1|Peru|Pelecanidae|Peru|Alphainfluenzavirus|Alphainfluenzavirusinfluenz"/>
NNN
</sequence>
<sequence>
<taxon idref="OQ550466.1|InfluenzaAvirus_A/Sanderling/Peru/PIU-SER005/2022_H5N1__segment4hemagglutinin_HA_genecompletecds|2022-11-22|4|H5N1|Peru|Calidrisalba|Peru|Alphainfluenzavirus|Alphainfluenzavirusinfluenz"/>

NNN
</sequence>
<sequence>
<taxon idref="OQ683458.1|InfluenzaAvirus_A/chicken/Colombia/Bolivar/3500/20
22_H5N1__segment4hemagglutinin_HA_genecompletecds|2022-11-
08|4|H5N1|Colombia|Gallusgallus|Colombia|Alphainfluenzavirus|Alphainfluenza
virusinfluenz"/>
NNN
</sequence>
<sequence>
<taxon idref="OQ683482.1|InfluenzaAvirus_A/chicken/Colombia/Cordoba/3499/20
22_H5N1__segment4hemagglutinin_HA_genecompletecds|2022-11-
26|4|H5N1|Colombia|Gallusgallus|Colombia|Alphainfluenzavirus|Alphainfluenza
virusinfluenz"/>
NNN
</sequence>
<sequence>
<taxon idref="OQ683490.1|InfluenzaAvirus_A/chicken/Colombia/Magdalena/3503/
2022_H5N1__segment4hemagglutinin_HA_genecompletecds|2022-11-
18|4|H5N1|Colombia|Gallusgallus|Colombia|Alphainfluenzavirus|Alphainfluenza
virusinfluenz"/>
NNN
</sequence>
<sequence>
<taxon idref="OQ732972.1|InfluenzaAvirus_A/blue-wingedteal/Texas/UGAI22-
2966/2022_H5N1__segment4hemagglutinin_HA_genecompletecds|2022-
09|4|H5N1|USA|Spatuladiscors|USA|Alphainfluenzavirus|Alphainfluenzavirusinf
luenz"/>
NNN
</sequence>
<sequence>
<taxon idref="OQ733012.1|InfluenzaAvirus_A/blue-wingedteal/Texas/UGAI22-
3250/2022_H5N1__segment4hemagglutinin_HA_genecompletecds|2022-
10|4|H5N1|USA|Spatuladiscors|USA|Alphainfluenzavirus|Alphainfluenzavirusinf
luenz"/>
NNN
</sequence>
<sequence>
<taxon idref="OQ733028.1|InfluenzaAvirus_A/blue-wingedteal/Texas/UGAI22-
3189/2022_H5N1__segment4hemagglutinin_HA_genecompletecds|2022-
10|4|H5N1|USA|Spatuladiscors|USA|Alphainfluenzavirus|Alphainfluenzavirusinf
luenz"/>
NNN
</sequence>
<sequence>
<taxon idref="OQ733036.1|InfluenzaAvirus_A/blue-wingedteal/Minnesota/AV22-
675/2022_H5N1__segment4hemagglutinin_HA_genecompletecds|2022-
09|4|H5N1|USA|Spatuladiscors|USA|Alphainfluenzavirus|Alphainfluenzavirusinf
luenz"/>
NNN
</sequence>
<sequence>
<taxon idref="OQ733100.1|InfluenzaAvirus_A/blue-wingedteal/Texas/UGAI22-
3190/2022_H5N1__segment4hemagglutinin_HA_genecompletecds|2022-
10|4|H5N1|USA|Spatuladiscors|USA|Alphainfluenzavirus|Alphainfluenzavirusinf
luenz"/>
NNN
</sequence>
<sequence>
<taxon idref="OQ733108.1|InfluenzaAvirus_A/blue-wingedteal/Texas/UGAI22-
2961/2022_H5N1__segment4hemagglutinin_HA_genecompletecds|2022-

09|4|H5N1|USA|Spatuladiscors|USA|Alphainfluenzavirus|Alphainfluenzavirusinf
luenz"/>
NNN
</sequence>
<sequence>
<taxon idref="OQ734894.1|InfluenzaAvirus_A/Americangreen-
wingedteal/Texas/UGAI22-
3462/2022_H5N1__segment4hemagglutinin_HA_genecompletecds|2022-09-
21|4|H5N1|USA|Anascarolinensis|USA|Alphainfluenzavirus|Alphainfluenzavirusi
nfluenz"/>
NNN
</sequence>
<sequence>
<taxon idref="OQ747759.1|InfluenzaAvirus_A/Belcher_s_gull/Peru/A102/2022_H5
N1__segment4hemagglutinin_HA_genecompletecds|2022-
11|4|H5N1|Peru||Peru|Alphainfluenzavirus|Alphainfluenzavirusinfluenz"/>
NNN
</sequence>
<sequence>
<taxon idref="OQ747761.1|InfluenzaAvirus_A/Belcher_s_gull/Peru/A267/2022_H5
N1__segment4hemagglutinin_HA_genecompletecds|2022-
12|4|H5N1|Peru||Peru|Alphainfluenzavirus|Alphainfluenzavirusinfluenz"/>
NNN
</sequence>
<sequence>
<taxon idref="OQ747762.1|InfluenzaAvirus_A/Americankestrel/Peru/A273/2022_H
5N1__segment4hemagglutinin_HA_genecompletecds|2022-
12|4|H5N1|Peru|Falcosparverius|Peru|Alphainfluenzavirus|Alphainfluenzavirus
influenz"/>
NNN
</sequence>
<sequence>
<taxon idref="OQ851647.1|InfluenzaAvirus_A/Pekinduck/California/T2202390/20
22_H5N1__segment4hemagglutinin_HA_genecompletecds|2022-11-
14|4|H5N1|USA|Anasplatyrhynchos|USA_California|Alphainfluenzavirus|Alphainf
luenzavirusinfluenz"/>
NNN
</sequence>
<sequence>
<taxon idref="OQ925704.1|InfluenzaAvirus_A/pelican/Peru/PIU-
SER013/2022_H5N1__segment4hemagglutinin_HA_genecompletecds|2022-11-
23|4|H5N1|Peru|Pelecanidae|Peru|Alphainfluenzavirus|Alphainfluenzavirusinfl
uenz"/>
NNN
</sequence>
<sequence>
<taxon idref="OQ954544.1|InfluenzaAvirus_A/stripedskunk/Kansas/W23-
175/2023_H5N1__segment4hemagglutinin_HA_genecompletecds|2023-02-
24|4|H5N1|USA|Mephitismephitis|USA|Alphainfluenzavirus|Alphainfluenzavirusi
nfluenz"/>
NNN
</sequence>
<sequence>
<taxon idref="OQ968028.1|InfluenzaAvirus_A/goose/Alaska/22-013831-
003/2022_H5N1__segment4hemagglutinin_HA_genecompletecds|2022-04-
27|4|H5N1|USA|Anatidae|USA_Alaska|Alphainfluenzavirus|Alphainfluenzavirusin
fluenz"/>
NNN
</sequence>
<sequence>

<taxon idref="OQ982404.1|InfluenzaAvirus_A/blue-
wingedteal/Louisiana/UGAI22-
3889/2022_H5N1__segment4hemagglutinin_HA_genecompletecds|2022-09-
23|4|H5N1|USA|Spatuladiscors|USA|Alphainfluenzavirus|Alphainfluenzavirusinf
luenz"/>
NNN
</sequence>
<sequence>
<taxon idref="OQ982420.1|InfluenzaAvirus_A/blue-
wingedteal/Louisiana/UGAI22-
3875/2022_H5N1__segment4hemagglutinin_HA_genecompletecds|2022-09-
23|4|H5N1|USA|Spatuladiscors|USA|Alphainfluenzavirus|Alphainfluenzavirusinf
luenz"/>
NNN
</sequence>
<sequence>
<taxon idref="OR591549.1|InfluenzaAvirus_A/Guanaycormorant/Peru/A275/2022_H
5N1__segment4hemagglutinin_HA_genecompletecds|2022-
12|4|H5N1|Peru||Peru|Alphainfluenzavirus|Alphainfluenzavirusinfluenz"/>
NNN
</sequence>
<sequence>
<taxon idref="OR591550.1|InfluenzaAvirus_A/Peruvianbooby/Peru/A296/2022_H5N
1__segment4hemagglutinin_HA_genecompletecds|2022-
12|4|H5N1|Peru|Sulavariegata|Peru|Alphainfluenzavirus|Alphainfluenzavirusin
fluenz"/>
NNN
</sequence>
<sequence>
<taxon idref="OR591551.1|InfluenzaAvirus_A/Belchergull/Peru/A267/2022_H5N1_
_segment4hemagglutinin_HA_genecompletecds|2022-
12|4|H5N1|Peru||Peru|Alphainfluenzavirus|Alphainfluenzavirusinfluenz"/>
NNN
</sequence>
<sequence>
<taxon idref="OR910145.1|InfluenzaAvirus_A/graygull/Atacama/235521-
1/2023_H5N1__segment4hemagglutinin_HA_genecompletecds|2023-02-
05|4|H5N1|Chile|Leucophaeusmodestus|Chile|Alphainfluenzavirus|Alphainfluenz
avirusinfluenz"/>
NNN
</sequence>
<sequence>
<taxon idref="OR910159.1|InfluenzaAvirus_A/cormorant/Antofagasta/236025-
1/2023_H5N1__segment4hemagglutinin_HA_genecompletecds|2023-02-
06|4|H5N1|Chile|Phalacrocorax|Chile|Alphainfluenzavirus|Alphainfluenzavirus
influenz"/>
NNN
</sequence>
<sequence>
<taxon idref="OR910172.1|InfluenzaAvirus_A/chicken/Nuble/241585/2023_H5N1__
segment4hemagglutinin_HA_genecompletecds|2023-03-
13|4|H5N1|Chile|Gallusgallus|Chile|Alphainfluenzavirus|Alphainfluenzavirusi
nfluenz"/>
NNN
</sequence>
<sequence>
<taxon idref="OR910178.1|InfluenzaAvirus_A/Peruvianpelican/Nuble/236068-
1/2023_H5N1__segment4hemagglutinin_HA_genecompletecds|2023-02-
10|4|H5N1|Chile|Pelecanusthagus|Chile|Alphainfluenzavirus|Alphainfluenzavir
usinfluenz"/>
NNN

</sequence>
<sequence>
<taxon idref="OR910186.1|InfluenzaAvirus_A/Band-tailedgull/Tarapaca/236339/2023_H5N1__segment4hemagglutinin_HA_genecompletecds|2023-02-10|4|H5N1|Chile|Larusbelcheri|Chile|Alphainfluenzavirus|Alphainfluenzavirusinfluenz"/>
NNN
</sequence>
<sequence>
<taxon idref="OR910192.1|InfluenzaAvirus_A/turkey/Valparaiso/245562-2/2023_H5N1__segment4hemagglutinin_HA_genecompletecds|2023-03-29|4|H5N1|Chile|Meleagrisgallopavo|Chile|Alphainfluenzavirus|Alphainfluenzavirusinfluenz"/>
NNN
</sequence>
<sequence>
<taxon idref="OR910199.1|InfluenzaAvirus_A/Peruvianbooby/Antofagasta/236613/2023_H5N1__segment4hemagglutinin_HA_genecompletecds|2023-02-13|4|H5N1|Chile|Sulavariegata|Chile|Alphainfluenzavirus|Alphainfluenzavirusinfluenz"/>
NNN
</sequence>
<sequence>
<taxon idref="OR910223.1|InfluenzaAvirus_A/pelican/AricayParinacota/235876-4/2023_H5N1__segment4hemagglutinin_HA_genecompletecds|2023-02-02|4|H5N1|Chile|Pelecanus|Chile|Alphainfluenzavirus|Alphainfluenzavirusinfluenz"/>
NNN
</sequence>
<sequence>
<taxon idref="OR910231.1|InfluenzaAvirus_A/chicken/Atacama/235254-6/2023_H5N1__segment4hemagglutinin_HA_genecompletecds|2023-02-04|4|H5N1|Chile|Gallusgallus|Chile|Alphainfluenzavirus|Alphainfluenzavirusinfluenz"/>
NNN
</sequence>
<sequence>
<taxon idref="OR910238.1|InfluenzaAvirus_A/chicken/Atacama/235254-1/2023_H5N1__segment4hemagglutinin_HA_genecompletecds|2023-02-04|4|H5N1|Chile|Gallusgallus|Chile|Alphainfluenzavirus|Alphainfluenzavirusinfluenz"/>
NNN
</sequence>
<sequence>
<taxon idref="OR910248.1|InfluenzaAvirus_A/Peruvianbooby/AricayParinacota/235900-1/2023_H5N1__segment4hemagglutinin_HA_genecompletecds|2023-02-05|4|H5N1|Chile|Sulavariegata|Chile|Alphainfluenzavirus|Alphainfluenzavirusinfluenz"/>
NNN
</sequence>
<sequence>
<taxon idref="OR910254.1|InfluenzaAvirus_A/pelican/BioBio/235399-1/2023_H5N1__segment4hemagglutinin_HA_genecompletecds|2023-02-06|4|H5N1|Chile|Pelecanus|Chile|Alphainfluenzavirus|Alphainfluenzavirusinfluenz"/>
NNN
</sequence>
<sequence>
<taxon idref="OR910261.1|InfluenzaAvirus_A/Turkeyvulture/Antofagasta/236643/2023_H5N1__segment4hemagglutinin_HA_genecompletecds|2023-02-

13|4|H5N1|Chile|Cathartesaura|Chile|Alphainfluenzavirus|Alphainfluenzavirus
influenz"/>
NNN
</sequence>
<sequence>
<taxon idref="OR910268.1|InfluenzaAvirus_A/kelpgull/OHiggins/235977-
1/2023_H5N1__segment4hemagglutinin_HA_genecompletecds|2023-02-
09|4|H5N1|Chile|Larusdominicanus|Chile|Alphainfluenzavirus|Alphainfluenzavi
rusinfluenz"/>
NNN
</sequence>
<sequence>
<taxon idref="OR910275.1|InfluenzaAvirus_A/chicken/Nuble/242159/2023_H5N1__
segment4hemagglutinin_HA_genecompletecds|2023-03-
15|4|H5N1|Chile|Gallusgallus|Chile|Alphainfluenzavirus|Alphainfluenzavirusi
nfluenz"/>
NNN
</sequence>
<sequence>
<taxon idref="OR910282.1|InfluenzaAvirus_A/chicken/Maule/242947-
1/2023_H5N1__segment4hemagglutinin_HA_genecompletecds|2023-03-
17|4|H5N1|Chile|Gallusgallus|Chile|Alphainfluenzavirus|Alphainfluenzavirusi
nfluenz"/>
NNN
</sequence>
<sequence>
<taxon idref="OR910295.1|InfluenzaAvirus_A/turkey/Valparaiso/245558-
4/2023_H5N1__segment4hemagglutinin_HA_genecompletecds|2023-03-
29|4|H5N1|Chile|Meleagrisgallopavo|Chile|Alphainfluenzavirus|Alphainfluenza
virusinfluenz"/>
NNN
</sequence>
<sequence>
<taxon idref="OR910301.1|InfluenzaAvirus_A/Peruvianbooby/Coquimbo/238918/20
23_H5N1__segment4hemagglutinin_HA_genecompletecds|2023-02-
25|4|H5N1|Chile|Sulavariegata|Chile|Alphainfluenzavirus|Alphainfluenzavirus
influenz"/>
NNN
</sequence>
<sequence>
<taxon idref="OR910308.1|InfluenzaAvirus_A/chicken/Araucania/245202/2023_H5
N1__segment4hemagglutinin_HA_genecompletecds|2023-03-
28|4|H5N1|Chile|Gallusgallus|Chile|Alphainfluenzavirus|Alphainfluenzavirusi
nfluenz"/>
NNN
</sequence>
<sequence>
<taxon idref="OR910318.1|InfluenzaAvirus_A/band-
tailedgull/AricayParinacota/235901-
2/2023_H5N1__segment4hemagglutinin_HA_genecompletecds|2023-02-
06|4|H5N1|Chile|Larusbelcheri|Chile|Alphainfluenzavirus|Alphainfluenzavirus
influenz"/>
NNN
</sequence>
<sequence>
<taxon idref="OR910331.1|InfluenzaAvirus_A/Peruvianbooby/AricayParinacota/2
35897-2/2023_H5N1__segment4hemagglutinin_HA_genecompletecds|2023-02-
03|4|H5N1|Chile|Sulavariegata|Chile|Alphainfluenzavirus|Alphainfluenzavirus
influenz"/>
NNN
</sequence>

<sequence>
<taxon idref="OR910346.1|InfluenzaAvirus_A/goose/Araucania/244373-1/2023_H5N1__segment4hemagglutinin_HA_genecompletecds|2023-03-21|4|H5N1|Chile|Anseranser|Chile|Alphainfluenzavirus|Alphainfluenzavirusinfluenz"/>
NNN
</sequence>
<sequence>
<taxon idref="OR910366.1|InfluenzaAvirus_A/Peruvianbooby/Coquimbo/239024/2023_H5N1__segment4hemagglutinin_HA_genecompletecds|2023-02-25|4|H5N1|Chile|Sulavariegata|Chile|Alphainfluenzavirus|Alphainfluenzavirusinfluenz"/>
NNN
</sequence>
<sequence>
<taxon idref="OR910371.1|InfluenzaAvirus_A/graygull/Antofagasta/236047-1/2023_H5N1__segment4hemagglutinin_HA_genecompletecds|2023-02-09|4|H5N1|Chile|Leucophaeusmodestus|Chile|Alphainfluenzavirus|Alphainfluenzavirusinfluenz"/>
NNN
</sequence>
<sequence>
<taxon idref="OR910384.1|InfluenzaAvirus_A/chicken/BioBio/241781/2023_H5N1__segment4hemagglutinin_HA_genecompletecds|2023-03-14|4|H5N1|Chile|Gallusgallus|Chile|Alphainfluenzavirus|Alphainfluenzavirusinfluenz"/>
NNN
</sequence>
<sequence>
<taxon idref="OR910389.1|InfluenzaAvirus_A/turkey/Valparaiso/245558-3/2023_H5N1__segment4hemagglutinin_HA_genecompletecds|2023-03-29|4|H5N1|Chile|Meleagrisgallopavo|Chile|Alphainfluenzavirus|Alphainfluenzavirusinfluenz"/>
NNN
</sequence>
<sequence>
<taxon idref="OR910396.1|InfluenzaAvirus_A/chicken/Atacama/235254-5/2023_H5N1__segment4hemagglutinin_HA_genecompletecds|2023-02-04|4|H5N1|Chile|Gallusgallus|Chile|Alphainfluenzavirus|Alphainfluenzavirusinfluenz"/>
NNN
</sequence>
<sequence>
<taxon idref="OR910404.1|InfluenzaAvirus_A/Peruvianbooby/Tarapaca/236308/2023_H5N1__segment4hemagglutinin_HA_genecompletecds|2023-02-09|4|H5N1|Chile|Sulavariegata|Chile|Alphainfluenzavirus|Alphainfluenzavirusinfluenz"/>
NNN
</sequence>
<sequence>
<taxon idref="OR910409.1|InfluenzaAvirus_A/domesticduck/OHiggins/245741-2/2023_H5N1__segment4hemagglutinin_HA_genecompletecds|2023-03-30|4|H5N1|Chile|Anasplatyrhynchos|Chile|Alphainfluenzavirus|Alphainfluenzavirusinfluenz"/>
NNN
</sequence>
<sequence>
<taxon idref="OR910415.1|InfluenzaAvirus_A/turkeyvulture/Atacama/241294/2023_H5N1__segment4hemagglutinin_HA_genecompletecds|2023-03-07|4|H5N1|Chile|Coragypsatratus|Chile|Alphainfluenzavirus|Alphainfluenzavirusinfluenz"/>

NNN
</sequence>
<sequence>
<taxon idref="OR910419.1|InfluenzaAvirus_A/Peruvianbooby/Valparaiso/236604/2023_H5N1__segment4hemagglutinin_HA_genecompletecds|2023-02-13|4|H5N1|Chile|Sulavariegata|Chile|Alphainfluenzavirus|Alphainfluenzavirusinfluenz"/>
NNN
</sequence>
<sequence>
<taxon idref="OR910425.1|InfluenzaAvirus_A/domesticduck/Araucania/243954-1/2023_H5N1__segment4hemagglutinin_HA_genecompletecds|2023-03-21|4|H5N1|Chile|Anasplatyrhynchos|Chile|Alphainfluenzavirus|Alphainfluenzavirusinfluenz"/>
NNN
</sequence>
<sequence>
<taxon idref="OR910435.1|InfluenzaAvirus_A/chicken/BioBio/245039-2/2023_H5N1__segment4hemagglutinin_HA_genecompletecds|2023-03-27|4|H5N1|Chile|Gallusgallus|Chile|Alphainfluenzavirus|Alphainfluenzavirusinfluenz"/>
NNN
</sequence>
<sequence>
<taxon idref="OR910447.1|InfluenzaAvirus_A/graygull/Tarapaca/236305/2023_H5N1__segment4hemagglutinin_HA_genecompletecds|2023-02-11|4|H5N1|Chile|Leucophaeusmodestus|Chile|Alphainfluenzavirus|Alphainfluenzavirusinfluenz"/>
NNN
</sequence>
<sequence>
<taxon idref="OR910460.1|InfluenzaAvirus_A/domesticduck/Metropolitana/243148-2/2023_H5N1__segment4hemagglutinin_HA_genecompletecds|2023-03-20|4|H5N1|Chile|Anasplatyrhynchos|Chile|Alphainfluenzavirus|Alphainfluenzavirusinfluenz"/>
NNN
</sequence>
<sequence>
<taxon idref="OR910468.1|InfluenzaAvirus_A/SouthAmericansealion/Valparaiso/243136-1/2023_H5N1__segment4hemagglutinin_HA_genecompletecds|2023-03-19|4|H5N1|Chile|Otariabyronia|Chile|Alphainfluenzavirus|Alphainfluenzavirusinfluenz"/>
NNN
</sequence>
<sequence>
<taxon idref="OR910474.1|InfluenzaAvirus_A/chicken/Maule/242133-1/2023_H5N1__segment4hemagglutinin_HA_genecompletecds|2023-03-15|4|H5N1|Chile|Gallusgallus|Chile|Alphainfluenzavirus|Alphainfluenzavirusinfluenz"/>
NNN
</sequence>
<sequence>
<taxon idref="OR910477.1|InfluenzaAvirus_A/chicken/Metropolitana/245704-1/2023_H5N1__segment4hemagglutinin_HA_genecompletecds|2023-03-30|4|H5N1|Chile|Gallusgallus|Chile|Alphainfluenzavirus|Alphainfluenzavirusinfluenz"/>
NNN
</sequence>
<sequence>
<taxon idref="OR910481.1|InfluenzaAvirus_A/SouthAmericansealion/Valparaiso/244738-1/2023_H5N1__segment4hemagglutinin_HA_genecompletecds|2023-03-

26|4|H5N1|Chile|Otariabyronia|Chile|Alphainfluenzavirus|Alphainfluenzavirus
influenz"/>
NNN
</sequence>
<sequence>
<taxon idref="OR910487.1|InfluenzaAvirus_A/greatgrabe/Nuble/244226-
1/2023_H5N1__segment4hemagglutinin_HA_genecompletecds|2023-03-
22|4|H5N1|Chile|Podicepsmajor|Chile|Alphainfluenzavirus|Alphainfluenzavirus
influenz"/>
NNN
</sequence>
<sequence>
<taxon idref="OR910502.1|InfluenzaAvirus_A/goose/Araucania/244409-
2/2023_H5N1__segment4hemagglutinin_HA_genecompletecds|2023-03-
24|4|H5N1|Chile|Anseranser|Chile|Alphainfluenzavirus|Alphainfluenzavirusinf
luenz"/>
NNN
</sequence>
<sequence>
<taxon idref="OR910509.1|InfluenzaAvirus_A/Peruvianbooby/AricayParinacota/2
34906-1/2023_H5N1__segment4hemagglutinin_HA_genecompletecds|2023-01-
31|4|H5N1|Chile|Sulavariegata|Chile|Alphainfluenzavirus|Alphainfluenzavirus
influenz"/>
NNN
</sequence>
<sequence>
<taxon idref="OR910515.1|InfluenzaAvirus_A/penguin/Antofagasta/234905-
1/2023_H5N1__segment4hemagglutinin_HA_genecompletecds|2023-01-
27|4|H5N1|Chile|Pygoscelis|Chile|Alphainfluenzavirus|Alphainfluenzavirusinf
luenz"/>
NNN
</sequence>
<sequence>
<taxon idref="OR910521.1|InfluenzaAvirus_A/kelpgull/Nuble/234619-
1/2023_H5N1__segment4hemagglutinin_HA_genecompletecds|2023-01-
30|4|H5N1|Chile|Larusdominicanus|Chile|Alphainfluenzavirus|Alphainfluenzavi
rusinfluenz"/>
NNN
</sequence>
<sequence>
<taxon idref="OR960985.1|InfluenzaAvirus_A/Chileandolphin/Maule/246026/2023
_H5N1__segment4hemagglutinin_HA_genecompletecds|2023-03-
31|4|H5N1|Chile|Cephalorhynchuseutropia|Chile|Alphainfluenzavirus|Alphainfl
uenzavirusinfluenz"/>
NNN
</sequence>
<sequence>
<taxon idref="OR960992.1|InfluenzaAvirus_A/guanaycormorant/Tarapaca/236301/
2023_H5N1__segment4hemagglutinin_HA_genecompletecds|2023-02-
09|4|H5N1|Chile|Leucocarbobougainvillii|Chile|Alphainfluenzavirus|Alphainfl
uenzavirusinfluenz"/>
NNN
</sequence>
<sequence>
<taxon idref="OR960998.1|InfluenzaAvirus_A/guanaycormorant/Atacama/235246-
1/2023_H5N1__segment4hemagglutinin_HA_genecompletecds|2023-02-
03|4|H5N1|Chile|Leucocarbobougainvillii|Chile|Alphainfluenzavirus|Alphainfl
uenzavirusinfluenz"/>
NNN
</sequence>
<sequence>

<taxon idref="OR961005.1|InfluenzaAvirus_A/guanaycormorant/AricayParinacota/235902-2/2023_H5N1__segment4hemagglutinin_HA_genecompletecds|2023-02-06|4|H5N1|Chile|Leucocarbobougainvillii|Chile|Alphainfluenzavirus|Alphainfluenzavirusinfluenz"/>
NNN
</sequence>
<sequence>
<taxon idref="OR961011.1|InfluenzaAvirus_A/guanaycormorant/Coquimbo/239080/2023_H5N1__segment4hemagglutinin_HA_genecompletecds|2023-02-27|4|H5N1|Chile|Leucocarbobougainvillii|Chile|Alphainfluenzavirus|Alphainfluenzavirusinfluenz"/>
NNN
</sequence>
<sequence>
<taxon idref="OR979626.1|InfluenzaAvirus_A/SouthAmericansealion/BioBio/246296-1/2023_H5N1__segment4hemagglutinin_HA_genecompletecds|2023-03-26|4|H5N1|Chile|Otariabyronia|Chile|Alphainfluenzavirus|Alphainfluenzavirusinfluenz"/>
NNN
</sequence>
<sequence>
<taxon idref="OR987084.1|InfluenzaAvirus_A/SouthAmericansealion/Argentina/RN-PB004/2023_H5N1__segment4hemagglutinin_HA_genecompletecds|2023-08-26|4|H5N1|Argentina|Otariabyronia|Argentina|Alphainfluenzavirus|Alphainfluenzavirusinfluenz"/>
NNN
</sequence>
<sequence>
<taxon idref="PP692235.1|InfluenzaAvirus_A/Calidrisalba/Lima/HA_4/2023_H5N1__segment4hemagglutinin_HA_genecompletecds|2023-04-20|4|H5N1|Peru|Calidrisalba|Peru_Lima|Alphainfluenzavirus|Alphainfluenzavirusinfluenz"/>
NNN
</sequence>
<sequence>
<taxon idref="PP761738.1|InfluenzaAvirus_A/snowgoose/SouthDakota/22-038501-001-original/2022_H5N1__segment4hemagglutinin_HA_genecompletecds|2022-11-20|4|H5N1|USA|Ansercaerulescens|USA|Alphainfluenzavirus|Alphainfluenzavirusinfluenz"/>
NNN
</sequence>
<sequence>
<taxon idref="PP801655.1|InfluenzaAvirus_A/commonraven/USA/22-020951-004/2022_H5N1__segment4hemagglutinin_HA_genecompletecds|2022-06-26|4|H5N1|USA|Corvuscorax|USA|Alphainfluenzavirus|Alphainfluenzavirusinfluenz"/>
NNN
</sequence>
<sequence>
<taxon idref="PP801696.1|InfluenzaAvirus_A/mallard/USA/22-029097-002/2022_H5N1__segment4hemagglutinin_HA_genecompletecds|2022-09-03|4|H5N1|USA|Anasplatyrhynchos|USA|Alphainfluenzavirus|Alphainfluenzavirusinfluenz"/>
NNN
</sequence>
<sequence>
<taxon idref="PP801703.1|InfluenzaAvirus_A/northernpintail/USA/22-029101-003/2022_H5N1__segment4hemagglutinin_HA_genecompletecds|2022-09-05|4|H5N1|USA|Anasacuta|USA|Alphainfluenzavirus|Alphainfluenzavirusinfluenz"/>
NNN

</sequence>
<sequence>
<taxon idref="PP801708.1|InfluenzaAvirus_A/green-wingedteal/USA/22-029101-004/2022_H5N1__segment4hemagglutinin_HA_genecompletecds|2022-09-05|4|H5N1|USA|Anascarolinensis|USA|Alphainfluenzavirus|Alphainfluenzavirusinfluenz"/>
NNN
</sequence>
<sequence>
<taxon idref="PP801715.1|InfluenzaAvirus_A/chicken/USA/22-031261-002/2022_H5N1__segment4hemagglutinin_HA_genecompletecds|2022-09-28|4|H5N1|USA|Gallusgallus|USA|Alphainfluenzavirus|Alphainfluenzavirusinfluenz"/>
NNN
</sequence>
<sequence>
<taxon idref="PP801722.1|InfluenzaAvirus_A/chicken/USA/22-031261-004/2022_H5N1__segment4hemagglutinin_HA_genecompletecds|2022-09-28|4|H5N1|USA|Gallusgallus|USA|Alphainfluenzavirus|Alphainfluenzavirusinfluenz"/>
NNN
</sequence>
<sequence>
<taxon idref="PP801727.1|InfluenzaAvirus_A/chicken/USA/22-031261-005/2022_H5N1__segment4hemagglutinin_HA_genecompletecds|2022-09-28|4|H5N1|USA|Gallusgallus|USA|Alphainfluenzavirus|Alphainfluenzavirusinfluenz"/>
NNN
</sequence>
<sequence>
<taxon idref="PP801741.1|InfluenzaAvirus_A/green-wingedteal/USA/22-031399-018/2022_H5N1__segment4hemagglutinin_HA_genecompletecds|2022-09-02|4|H5N1|USA|Anascarolinensis|USA|Alphainfluenzavirus|Alphainfluenzavirusinfluenz"/>
NNN
</sequence>
<sequence>
<taxon idref="PP801748.1|InfluenzaAvirus_A/chicken/USA/22-032677-005/2022_H5N1__segment4hemagglutinin_HA_genecompletecds|2022-10-11|4|H5N1|USA|Gallusgallus|USA|Alphainfluenzavirus|Alphainfluenzavirusinfluenz"/>
NNN
</sequence>
<sequence>
<taxon idref="PP801781.1|InfluenzaAvirus_A/northernpintail/USA/22-034099-001/2022_H5N1__segment4hemagglutinin_HA_genecompletecds|2022-10-05|4|H5N1|USA|Anasacuta|USA|Alphainfluenzavirus|Alphainfluenzavirusinfluenz"/>
NNN
</sequence>
<sequence>
<taxon idref="PP801792.1|InfluenzaAvirus_A/mallard/USA/22-036139-002/2022_H5N1__segment4hemagglutinin_HA_genecompletecds|2022-11-02|4|H5N1|USA|Anasplatyrhynchos|USA|Alphainfluenzavirus|Alphainfluenzavirusinfluenz"/>
NNN
</sequence>
<sequence>
<taxon idref="PP801798.1|InfluenzaAvirus_A/mallard/USA/22-036139-016/2022_H5N1__segment4hemagglutinin_HA_genecompletecds|2022-11-

02|4|H5N1|USA|Anasplatyrhynchos|USA|Alphainfluenzavirus|Alphainfluenzavirus influenz"/>
NNN
</sequence>
<sequence>
<taxon idref="PP801802.1|InfluenzaAvirus_A/mallard/USA/22-036139-018/2022_H5N1__segment4hemagglutinin_HA_genecompletecds|2022-11-02|4|H5N1|USA|Anasplatyrhynchos|USA|Alphainfluenzavirus|Alphainfluenzavirus influenz"/>
NNN
</sequence>
<sequence>
<taxon idref="PP801810.1|InfluenzaAvirus_A/mallard/USA/22-036139-054/2022_H5N1__segment4hemagglutinin_HA_genecompletecds|2022-11-02|4|H5N1|USA|Anasplatyrhynchos|USA|Alphainfluenzavirus|Alphainfluenzavirus influenz"/>
NNN
</sequence>
<sequence>
<taxon idref="PP801816.1|InfluenzaAvirus_A/mallard/USA/22-036139-057/2022_H5N1__segment4hemagglutinin_HA_genecompletecds|2022-11-02|4|H5N1|USA|Anasplatyrhynchos|USA|Alphainfluenzavirus|Alphainfluenzavirus influenz"/>
NNN
</sequence>
<sequence>
<taxon idref="PP801843.1|InfluenzaAvirus_A/greatblueheron/USA/22-036616-001/2022_H5N1__segment4hemagglutinin_HA_genecompletecds|2022-11-08|4|H5N1|USA|Ardeaherodias|USA|Alphainfluenzavirus|Alphainfluenzavirusinfluenz"/>
NNN
</sequence>
<sequence>
<taxon idref="PP801863.1|InfluenzaAvirus_A/chicken/USA/22-037940-001/2022_H5N1__segment4hemagglutinin_HA_genecompletecds|2022-11-23|4|H5N1|USA|Gallusgallus|USA|Alphainfluenzavirus|Alphainfluenzavirusinfluenz"/>
NNN
</sequence>
<sequence>
<taxon idref="PP801974.1|InfluenzaAvirus_A/mallard/USA/IZ22_0788/2022_H5N1__segment4hemagglutinin_HA_genecompletecds|2022-09-30|4|H5N1|USA|Anasplatyrhynchos|USA|Alphainfluenzavirus|Alphainfluenzavirus influenz"/>
NNN
</sequence>
<sequence>
<taxon idref="PP801982.1|InfluenzaAvirus_A/Americanwigeon/USA/22-034097-002/2022_H5N1__segment4hemagglutinin_HA_genecompletecds|2022-10-01|4|H5N1|USA|Marecaamericana|USA|Alphainfluenzavirus|Alphainfluenzavirusinfluenz"/>
NNN
</sequence>
<sequence>
<taxon idref="PP802022.1|InfluenzaAvirus_A/Canadagoose/USA/22-013831-003/2022_H5N1__segment4hemagglutinin_HA_genecompletecds|2022-04-29|4|H5N1|USA|Brantacanadensis|USA|Alphainfluenzavirus|Alphainfluenzavirusinfluenz"/>
NNN
</sequence>
<sequence>

<taxon idref="PP802254.1|InfluenzaAvirus_A/cacklinggoose/USA/IZ22_0116/2022_H5N1__segment4hemagglutinin_HA_genecompletecds|2022-09-05|4|H5N1|USA|Brantahutchinsii|USA|Alphainfluenzavirus|Alphainfluenzavirusinfluenz"/>
NNN
</sequence>
<sequence>
<taxon idref="PP802262.1|InfluenzaAvirus_A/cacklinggoose/USA/IZ22_0117/2022_H5N1__segment4hemagglutinin_HA_genecompletecds|2022-09-05|4|H5N1|USA|Brantahutchinsii|USA|Alphainfluenzavirus|Alphainfluenzavirusinfluenz"/>
NNN
</sequence>
<sequence>
<taxon idref="PP802270.1|InfluenzaAvirus_A/cacklinggoose/USA/IZ22_0251/2022_H5N1__segment4hemagglutinin_HA_genecompletecds|2022-09-09|4|H5N1|USA|Brantahutchinsii|USA|Alphainfluenzavirus|Alphainfluenzavirusinfluenz"/>
NNN
</sequence>
<sequence>
<taxon idref="PP802278.1|InfluenzaAvirus_A/cacklinggoose/USA/IZ22_0787/2022_H5N1__segment4hemagglutinin_HA_genecompletecds|2022-09-30|4|H5N1|USA|Brantahutchinsii|USA|Alphainfluenzavirus|Alphainfluenzavirusinfluenz"/>
NNN
</sequence>
<sequence>
<taxon idref="PP802310.1|InfluenzaAvirus_A/chicken/USA/22-031261-003/2022_H5N1__segment4hemagglutinin_HA_genecompletecds|2022-09-28|4|H5N1|USA|Gallusgallus|USA|Alphainfluenzavirus|Alphainfluenzavirusinfluenz"/>
NNN
</sequence>
<sequence>
<taxon idref="PP802318.1|InfluenzaAvirus_A/chicken/USA/22-032677-003/2022_H5N1__segment4hemagglutinin_HA_genecompletecds|2022-10-11|4|H5N1|USA|Gallusgallus|USA|Alphainfluenzavirus|Alphainfluenzavirusinfluenz"/>
NNN
</sequence>
<sequence>
<taxon idref="PP802350.1|InfluenzaAvirus_A/commonraven/USA/22-022817-001/2022_H5N1__segment4hemagglutinin_HA_genecompletecds|2022-07-20|4|H5N1|USA|Corvuscorax|USA|Alphainfluenzavirus|Alphainfluenzavirusinfluenz"/>
NNN
</sequence>
<sequence>
<taxon idref="PP802390.1|InfluenzaAvirus_A/domesticturkey/USA/22-032677-001/2022_H5N1__segment4hemagglutinin_HA_genecompletecds|2022-10-11|4|H5N1|USA|Meleagrisgallopavo|USA|Alphainfluenzavirus|Alphainfluenzavirusinfluenz"/>
NNN
</sequence>
<sequence>
<taxon idref="PP802398.1|InfluenzaAvirus_A/domesticturkey/USA/22-032677-004/2022_H5N1__segment4hemagglutinin_HA_genecompletecds|2022-10-11|4|H5N1|USA|Meleagrisgallopavo|USA|Alphainfluenzavirus|Alphainfluenzavirusinfluenz"/>
NNN

</sequence>
<sequence>
<taxon idref="PP802406.1|InfluenzaAvirus_A/domesticturkey/USA/22-032677-006/2022_H5N1__segment4hemagglutinin_HA_genecompletecds|2022-10-11|4|H5N1|USA|Meleagrisgallopavo|USA|Alphainfluenzavirus|Alphainfluenzavirusinfluenz"/>
NNN
</sequence>
<sequence>
<taxon idref="PP802518.1|InfluenzaAvirus_A/green-wingedteal/USA/22-031399-019/2022_H5N1__segment4hemagglutinin_HA_genecompletecds|2022-09-02|4|H5N1|USA|Anascarolinensis|USA|Alphainfluenzavirus|Alphainfluenzavirusinfluenz"/>
NNN
</sequence>
<sequence>
<taxon idref="PP802622.1|InfluenzaAvirus_A/mallard/USA/22-036139-048/2022_H5N1__segment4hemagglutinin_HA_genecompletecds|2022-11-02|4|H5N1|USA|Anasplatyrhynchos|USA|Alphainfluenzavirus|Alphainfluenzavirusinfluenz"/>
NNN
</sequence>
<sequence>
<taxon idref="PP802654.1|InfluenzaAvirus_A/mallard/USA/IZ22_0392/2022_H5N1__segment4hemagglutinin_HA_genecompletecds|2022-09-16|4|H5N1|USA|Anasplatyrhynchos|USA|Alphainfluenzavirus|Alphainfluenzavirusinfluenz"/>
NNN
</sequence>
<sequence>
<taxon idref="PP802662.1|InfluenzaAvirus_A/mallard/USA/IZ22_0766/2022_H5N1__segment4hemagglutinin_HA_genecompletecds|2022-09-30|4|H5N1|USA|Anasplatyrhynchos|USA|Alphainfluenzavirus|Alphainfluenzavirusinfluenz"/>
NNN
</sequence>
<sequence>
<taxon idref="PP802670.1|InfluenzaAvirus_A/mallard/USA/IZ22_0791/2022_H5N1__segment4hemagglutinin_HA_genecompletecds|2022-09-30|4|H5N1|USA|Anasplatyrhynchos|USA|Alphainfluenzavirus|Alphainfluenzavirusinfluenz"/>
NNN
</sequence>
<sequence>
<taxon idref="PP802678.1|InfluenzaAvirus_A/mallard/USA/IZ22_0879/2022_H5N1__segment4hemagglutinin_HA_genecompletecds|2022-10-09|4|H5N1|USA|Anasplatyrhynchos|USA|Alphainfluenzavirus|Alphainfluenzavirusinfluenz"/>
NNN
</sequence>
<sequence>
<taxon idref="PP802686.1|InfluenzaAvirus_A/mallard/USA/IZ22_0894/2022_H5N1__segment4hemagglutinin_HA_genecompletecds|2022-10-14|4|H5N1|USA|Anasplatyrhynchos|USA|Alphainfluenzavirus|Alphainfluenzavirusinfluenz"/>
NNN
</sequence>
<sequence>
<taxon idref="PP802702.1|InfluenzaAvirus_A/northernpintail/USA/22-031399-012/2022_H5N1__segment4hemagglutinin_HA_genecompletecds|2022-09-

02|4|H5N1|USA|Anasacuta|USA|Alphainfluenzavirus|Alphainfluenzavirusinfluenz
"/>
NNN
</sequence>
<sequence>
<taxon idref="PP802710.1|InfluenzaAvirus_A/northernpintail/USA/22-031399-
015/2022_H5N1__segment4hemagglutinin_HA_genecompletecds|2022-09-
02|4|H5N1|USA|Anasacuta|USA|Alphainfluenzavirus|Alphainfluenzavirusinfluenz
"/>
NNN
</sequence>
<sequence>
<taxon idref="PQ002146.1|InfluenzaAvirus_A/Southernelephantseal/PeninsulaVa
ldes/HA_CH-
PD032_brain/2023_H5N1__segment4hemagglutinin_HA_genecompletecds|2023-10-
10|4|H5N1|Argentina|Miroungaleonina|Argentina_ChubutPeninsulaValdes|Alphain
fluenzavirus|"/>
NNN
</sequence>
<sequence>
<taxon idref="PQ113953.1|InfluenzaAvirus_A/SouthernElephantSeal/SouthGeorgi
aandtheSouthSandwichIslands/24/2023_H5N1__segment4hemagglutinin_HA_genecomp
letecds|2023-12-
09|4|H5N1|SouthGeorgiaandtheSouthSandwichIslands|Miroungaleonina|SouthGe"/>
NNN
</sequence>
<sequence>
<taxon idref="PQ113961.1|InfluenzaAvirus_A/KelpGull/SouthGeorgiaandtheSouth
SandwichIslands/32/2023_H5N1__segment4hemagglutinin_HA_genecompletecds|2023
-11-
03|4|H5N1|SouthGeorgiaandtheSouthSandwichIslands|Larusdominicanus|SouthGeor
giaandt"/>
NNN
</sequence>
<sequence>
<taxon idref="PQ113969.1|InfluenzaAvirus_A/AntarcticTern/SouthGeorgiaandthe
SouthSandwichIslands/40/2023_H5N1__segment4hemagglutinin_HA_genecompletecds
|2023-12-
06|4|H5N1|SouthGeorgiaandtheSouthSandwichIslands|Sternavittata|SouthGeorgia
and"/>
NNN
</sequence>
<sequence>
<taxon idref="PQ113977.1|InfluenzaAvirus_A/BrownSkua/SouthGeorgiaandtheSout
hSandwichIslands/48/2023_H5N1__segment4hemagglutinin_HA_genecompletecds|202
3-10-
31|4|H5N1|SouthGeorgiaandtheSouthSandwichIslands|Stercorariuslonnbergi|Sout
hGeorgia"/>
NNN
</sequence>
<sequence>
<taxon idref="PQ113985.1|InfluenzaAvirus_A/KelpGull/SouthGeorgiaandtheSouth
SandwichIslands/56/2023_H5N1__segment4hemagglutinin_HA_genecompletecds|2023
-10-
31|4|H5N1|SouthGeorgiaandtheSouthSandwichIslands|Larusdominicanus|SouthGeor
giaandt"/>
NNN
</sequence>
<sequence>
<taxon idref="PQ113993.1|InfluenzaAvirus_A/BrownSkua/SouthGeorgiaandtheSout
hSandwichIslands/64/2023_H5N1__segment4hemagglutinin_HA_genecompletecds|202

3-10-
31|4|H5N1|SouthGeorgiaandtheSouthSandwichIslands|Stercorariuslonnbergi|Sout
hGeorgia"/>
NNN
</sequence>
<sequence>
<taxon idref="PQ114017.1|InfluenzaAvirus_A/SouthGeorgiaShag/SouthGeorgiaand
theSouthSandwichIslands/88/2023_H5N1__segment4hemagglutinin_HA_genecomplete
cds|2023-11-
27|4|H5N1|SouthGeorgiaandtheSouthSandwichIslands|Leucocarbogeorgianus|South
G"/>
NNN
</sequence>
<sequence>
<taxon idref="PQ114025.1|InfluenzaAvirus_A/BrownSkua/SouthGeorgiaandtheSout
hSandwichIslands/96/2023_H5N1__segment4hemagglutinin_HA_genecompletecds|202
3-10-
30|4|H5N1|SouthGeorgiaandtheSouthSandwichIslands|Stercorariuslonnbergi|Sout
hGeorgia"/>
NNN
</sequence>
<sequence>
<taxon idref="PQ114033.1|InfluenzaAvirus_A/BrownSkua/SouthGeorgiaandtheSout
hSandwichIslands/104/2023_H5N1__segment4hemagglutinin_HA_genecompletecds|20
23-11-
15|4|H5N1|SouthGeorgiaandtheSouthSandwichIslands|Stercorariuslonnbergi|Sout
hGeorgi"/>
NNN
</sequence>
<sequence>
<taxon idref="PQ114073.1|InfluenzaAvirus_A/BrownSkua/SouthGeorgiaandtheSout
hSandwichIslands/144/2023_H5N1__segment4hemagglutinin_HA_genecompletecds|20
23-11-
11|4|H5N1|SouthGeorgiaandtheSouthSandwichIslands|Stercorariuslonnbergi|Sout
hGeorgi"/>
NNN
</sequence>
<sequence>
<taxon idref="PQ114081.1|InfluenzaAvirus_A/KelpGull/SouthGeorgiaandtheSouth
SandwichIslands/152/2023_H5N1__segment4hemagglutinin_HA_genecompletecds|202
3-10-
30|4|H5N1|SouthGeorgiaandtheSouthSandwichIslands|Larusdominicanus|SouthGeor
giaandt"/>
NNN
</sequence>
<sequence>
<taxon idref="PQ129506.1|InfluenzaAvirus_A/BrownSkua/SouthGeorgiaandtheSout
hSandwichIslands/8/2023_H5N1__segment4hemagglutinin_HA_genecompletecds|2023
-10-
08|4|H5N1|SouthGeorgiaandtheSouthSandwichIslands|Stercorariuslonnbergi|Sout
hGeorgiaa"/>
NNN
</sequence>
<sequence>
<taxon idref="PQ129514.1|InfluenzaAvirus_A/BrownSkua/SouthGeorgiaandtheSout
hSandwichIslands/16/2023_H5N1__segment4hemagglutinin_HA_genecompletecds|202
3-10-
08|4|H5N1|SouthGeorgiaandtheSouthSandwichIslands|Stercorariuslonnbergi|Sout
hGeorgia"/>
NNN
</sequence>

<sequence>
<taxon idref="PQ304566.1|InfluenzaAvirus_A/Southpolarskua/Antarctica/INACH-UC-UCHILE-SKU5L/2024_H5N1__segment4hemagglutinin_HA_genecompletecds|2024-03-03|4|H5N1|Chile|Stercorariusmaccormicki|Chile|Alphainfluenzavirus|Alphainfluenzavirusinfluenz"/>
NNN
</sequence>
<sequence>
<taxon idref="PQ701225.1|InfluenzaAvirus_A/AmericanBlue-wingedteal/MN/22-029882-002-original/2022_H5N1__segment4hemagglutinin_HA_genecompletecds|2022-09-11|4|H5N1|USA||USA_MN|Alphainfluenzavirus|Alphainfluenzavirusinfluenz"/>
NNN
</sequence>
<sequence>
<taxon idref="PQ701297.1|InfluenzaAvirus_A/AmericanBlue-wingedteal/OK/22-032994-002-original/2022_H5N1__segment4hemagglutinin_HA_genecompletecds|2022-10-10|4|H5N1|USA||USA_OK|Alphainfluenzavirus|Alphainfluenzavirusinfluenz"/>
NNN
</sequence>
<sequence>
<taxon idref="PQ701305.1|InfluenzaAvirus_A/AmericanBlue-wingedteal/TX/22-029888-002-original/2022_H5N1__segment4hemagglutinin_HA_genecompletecds|2022-09-11|4|H5N1|USA||USA_TX|Alphainfluenzavirus|Alphainfluenzavirusinfluenz"/>
NNN
</sequence>
<sequence>
<taxon idref="PQ701313.1|InfluenzaAvirus_A/AmericanBlue-wingedteal/TX/22-036754-004-original-repeat/2022_H5N1__segment4hemagglutinin_HA_genecompletecds|2022-11-06|4|H5N1|USA||USA_TX|Alphainfluenzavirus|Alphainfluenzavirusinfluenz"/>
NNN
</sequence>
<sequence>
<taxon idref="PQ701329.1|InfluenzaAvirus_A/AmericanblackDuck/VA/22-038573-010-original/2022_H5N1__segment4hemagglutinin_HA_genecompletecds|2022-11-19|4|H5N1|USA|Anasrubripes|USA_VA|Alphainfluenzavirus|Alphainfluenzavirusinfluenz"/>
NNN
</sequence>
<sequence>
<taxon idref="PQ701569.1|InfluenzaAvirus_A/Americancrow/OR/23-002234-001-original/2022_H5N1__segment4hemagglutinin_HA_genecompletecds|2022-11-16|4|H5N1|USA|Corvusbrachyrhynchos|USA_OR|Alphainfluenzavirus|Alphainfluenzavirusinfluenz"/>
NNN
</sequence>
<sequence>
<taxon idref="PQ701633.1|InfluenzaAvirus_A/Americangreen-wingedteal/AK/22-029101-004-original/2022_H5N1__segment4hemagglutinin_HA_genecompletecds|2022-09-05|4|H5N1|USA|Anascarolinensis|USA_AK|Alphainfluenzavirus|Alphainfluenzavirusinfluenz"/>
NNN
</sequence>
<sequence>
<taxon idref="PQ701649.1|InfluenzaAvirus_A/Americangreen-wingedteal/AK/22-039438-007-

original/2022_H5N1__segment4hemagglutinin_HA_genecompletecds|2022-09-07|4|H5N1|USA|Anascarolinensis|USA_AK|Alphainfluenzavirus|Alphainfluenzavirusinfluenz"/>
NNN
</sequence>
<sequence>
<taxon idref="PQ701665.1|InfluenzaAvirus_A/Americangreen-wingedteal/CA/23-005876-017-original/2022_H5N1__segment4hemagglutinin_HA_genecompletecds|2022-10-23|4|H5N1|USA|Anascarolinensis|USA_CA|Alphainfluenzavirus|Alphainfluenzavirusinfluenz"/>
NNN
</sequence>
<sequence>
<taxon idref="PQ701697.1|InfluenzaAvirus_A/Americangreen-wingedteal/IA/22-034928-001-original-repeat2/2022_H5N1__segment4hemagglutinin_HA_genecompletecds|2022-10-16|4|H5N1|USA|Anascarolinensis|USA_IA|Alphainfluenzavirus|Alphainfluenzavirusinfluenz"/>
NNN
</sequence>
<sequence>
<taxon idref="PQ701705.1|InfluenzaAvirus_A/Americangreen-wingedteal/ID/22-034533-007-original-repeat2/2022_H5N1__segment4hemagglutinin_HA_genecompletecds|2022-10-01|4|H5N1|USA|Anascarolinensis|USA_ID|Alphainfluenzavirus|Alphainfluenzavirusinfluenz"/>
NNN
</sequence>
<sequence>
<taxon idref="PQ701745.1|InfluenzaAvirus_A/Americangreen-wingedteal/MN/23-013078-030-original/2022_H5N1__segment4hemagglutinin_HA_genecompletecds|2022-09-17|4|H5N1|USA|Anascarolinensis|USA_MN|Alphainfluenzavirus|Alphainfluenzavirusinfluenz"/>
NNN
</sequence>
<sequence>
<taxon idref="PQ701793.1|InfluenzaAvirus_A/Americangreen-wingedteal/ND/22-032267-001-original-repeat/2022_H5N1__segment4hemagglutinin_HA_genecompletecds|2022-10-01|4|H5N1|USA|Anascarolinensis|USA_ND|Alphainfluenzavirus|Alphainfluenzavirusinfluenz"/>
NNN
</sequence>
<sequence>
<taxon idref="PQ701809.1|InfluenzaAvirus_A/Americangreen-wingedteal/NY/22-033592-029-original/2022_H5N1__segment4hemagglutinin_HA_genecompletecds|2022-10-15|4|H5N1|USA|Anascarolinensis|USA_NY|Alphainfluenzavirus|Alphainfluenzavirusinfluenz"/>
NNN
</sequence>
<sequence>
<taxon idref="PQ701833.1|InfluenzaAvirus_A/Americangreen-wingedteal/OR/22-037268-011-original/2022_H5N1__segment4hemagglutinin_HA_genecompletecds|2022-11-12|4|H5N1|USA|Anascarolinensis|USA_OR|Alphainfluenzavirus|Alphainfluenzavirusinfluenz"/>
NNN
</sequence>

<sequence>
<taxon idref="PQ701953.1|InfluenzaAvirus_A/AmericanwhitePelican/CA/22-035596-001-original/2022_H5N1__segment4hemagglutinin_HA_genecompletecds|2022-10-27|4|H5N1|USA|Pelecanuserythrorhynchos|USA_CA|Alphainfluenzavirus|Alphainfluenzavirusinfluenz"/>
NNN
</sequence>
<sequence>
<taxon idref="PQ702113.1|InfluenzaAvirus_A/AmericanwhitePelican/ND/22-032266-001-original-repeat/2022_H5N1__segment4hemagglutinin_HA_genecompletecds|2022-09-28|4|H5N1|USA|Pelecanuserythrorhynchos|USA_ND|Alphainfluenzavirus|Alphainfluenzavirusinfluen"/>
NNN
</sequence>
<sequence>
<taxon idref="PQ702161.1|InfluenzaAvirus_A/AmericanwhitePelican/UT/22-032952-010-original/2022_H5N1__segment4hemagglutinin_HA_genecompletecds|2022-10-13|4|H5N1|USA|Pelecanuserythrorhynchos|USA_UT|Alphainfluenzavirus|Alphainfluenzavirusinfluenz"/>
NNN
</sequence>
<sequence>
<taxon idref="PQ702273.1|InfluenzaAvirus_A/Americanwigeon/CA/23-001148-019-original/2022_H5N1__segment4hemagglutinin_HA_genecompletecds|2022-10-22|4|H5N1|USA|Marecaamericana|USA_CA|Alphainfluenzavirus|Alphainfluenzavirusinfluenz"/>
NNN
</sequence>
<sequence>
<taxon idref="PQ702289.1|InfluenzaAvirus_A/Americanwigeon/ID/22-036873-001-original/2022_H5N1__segment4hemagglutinin_HA_genecompletecds|2022-10-26|4|H5N1|USA|Marecaamericana|USA_ID|Alphainfluenzavirus|Alphainfluenzavirusinfluenz"/>
NNN
</sequence>
<sequence>
<taxon idref="PQ702297.1|InfluenzaAvirus_A/Americanwigeon/ID/22-039598-001-original/2022_H5N1__segment4hemagglutinin_HA_genecompletecds|2022-11-23|4|H5N1|USA|Marecaamericana|USA_ID|Alphainfluenzavirus|Alphainfluenzavirusinfluenz"/>
NNN
</sequence>
<sequence>
<taxon idref="PQ702337.1|InfluenzaAvirus_A/Americanwigeon/OH/22-034080-032-original/2022_H5N1__segment4hemagglutinin_HA_genecompletecds|2022-10-15|4|H5N1|USA|Marecaamericana|USA_OH|Alphainfluenzavirus|Alphainfluenzavirusinfluenz"/>
NNN
</sequence>
<sequence>
<taxon idref="PQ702353.1|InfluenzaAvirus_A/Americanwigeon/OR/22-033876-004-original/2022_H5N1__segment4hemagglutinin_HA_genecompletecds|2022-10-15|4|H5N1|USA|Marecaamericana|USA_OR|Alphainfluenzavirus|Alphainfluenzavirusinfluenz"/>
NNN
</sequence>
<sequence>

<taxon idref="PQ702361.1|InfluenzaAvirus_A/Americanwigeon/OR/22-033876-019-original/2022_H5N1__segment4hemagglutinin_HA_genecompletecds|2022-10-15|4|H5N1|USA|Marecaamericana|USA_OR|Alphainfluenzavirus|Alphainfluenzavirusinfluenz"/>
NNN
</sequence>
<sequence>
<taxon idref="PQ702369.1|InfluenzaAvirus_A/Americanwigeon/OR/22-033877-003-original/2022_H5N1__segment4hemagglutinin_HA_genecompletecds|2022-10-16|4|H5N1|USA|Marecaamericana|USA_OR|Alphainfluenzavirus|Alphainfluenzavirusinfluenz"/>
NNN
</sequence>
<sequence>
<taxon idref="PQ702377.1|InfluenzaAvirus_A/Americanwigeon/OR/22-033877-019-original-repeat2/2022_H5N1__segment4hemagglutinin_HA_genecompletecds|2022-10-16|4|H5N1|USA|Marecaamericana|USA_OR|Alphainfluenzavirus|Alphainfluenzavirusinfluenz"/>
NNN
</sequence>
<sequence>
<taxon idref="PQ702385.1|InfluenzaAvirus_A/Americanwigeon/OR/22-034677-053-original-repeat2/2022_H5N1__segment4hemagglutinin_HA_genecompletecds|2022-10-08|4|H5N1|USA|Marecaamericana|USA_OR|Alphainfluenzavirus|Alphainfluenzavirusinfluenz"/>
NNN
</sequence>
<sequence>
<taxon idref="PQ702393.1|InfluenzaAvirus_A/Americanwigeon/OR/22-034677-056-original-repeat2/2022_H5N1__segment4hemagglutinin_HA_genecompletecds|2022-10-08|4|H5N1|USA|Marecaamericana|USA_OR|Alphainfluenzavirus|Alphainfluenzavirusinfluenz"/>
NNN
</sequence>
<sequence>
<taxon idref="PQ702401.1|InfluenzaAvirus_A/Americanwigeon/OR/22-035736-015-original/2022_H5N1__segment4hemagglutinin_HA_genecompletecds|2022-10-27|4|H5N1|USA|Marecaamericana|USA_OR|Alphainfluenzavirus|Alphainfluenzavirusinfluenz"/>
NNN
</sequence>
<sequence>
<taxon idref="PQ702409.1|InfluenzaAvirus_A/Americanwigeon/OR/22-036880-012-original/2022_H5N1__segment4hemagglutinin_HA_genecompletecds|2022-11-07|4|H5N1|USA|Marecaamericana|USA_OR|Alphainfluenzavirus|Alphainfluenzavirusinfluenz"/>
NNN
</sequence>
<sequence>
<taxon idref="PQ702449.1|InfluenzaAvirus_A/Americanwigeon/TX/22-036753-014-original-repeat/2022_H5N1__segment4hemagglutinin_HA_genecompletecds|2022-11-05|4|H5N1|USA|Marecaamericana|USA_TX|Alphainfluenzavirus|Alphainfluenzavirusinfluenz"/>
NNN
</sequence>
<sequence>

<taxon idref="PQ702457.1|InfluenzaAvirus_A/Americanwigeon/TX/22-036753-025-original-repeat/2022_H5N1__segment4hemagglutinin_HA_genecompletecds|2022-11-05|4|H5N1|USA|Marecaamericana|USA_TX|Alphainfluenzavirus|Alphainfluenzavirusinfluenz"/>
NNN
</sequence>
<sequence>
<taxon idref="PQ702465.1|InfluenzaAvirus_A/Americanwigeon/TX/22-036753-038-original-repeat/2022_H5N1__segment4hemagglutinin_HA_genecompletecds|2022-11-05|4|H5N1|USA|Marecaamericana|USA_TX|Alphainfluenzavirus|Alphainfluenzavirusinfluenz"/>
NNN
</sequence>
<sequence>
<taxon idref="PQ702473.1|InfluenzaAvirus_A/Americanwigeon/TX/22-036753-056-original-repeat/2022_H5N1__segment4hemagglutinin_HA_genecompletecds|2022-11-05|4|H5N1|USA|Marecaamericana|USA_TX|Alphainfluenzavirus|Alphainfluenzavirusinfluenz"/>
NNN
</sequence>
<sequence>
<taxon idref="PQ702513.1|InfluenzaAvirus_A/Arctictern/AK/22-020952-002-original/2022_H5N1__segment4hemagglutinin_HA_genecompletecds|2022-07-22|4|H5N1|USA|Sternaparadisaea|USA_AK|Alphainfluenzavirus|Alphainfluenzavirusinfluenz"/>
NNN
</sequence>
<sequence>
<taxon idref="PQ702529.1|InfluenzaAvirus_A/Baldeagle/AK/22-014971-001-original/2022_H5N1__segment4hemagglutinin_HA_genecompletecds|2022-05-09|4|H5N1|USA|Haliaeetusleucocephalus|USA_AK|Alphainfluenzavirus|Alphainfluenzavirusinfluenz"/>
NNN
</sequence>
<sequence>
<taxon idref="PQ702593.1|InfluenzaAvirus_A/Baldeagle/AK/22-016934-004-original/2022_H5N1__segment4hemagglutinin_HA_genecompletecds|2022-05-22|4|H5N1|USA|Haliaeetusleucocephalus|USA_AK|Alphainfluenzavirus|Alphainfluenzavirusinfluenz"/>
NNN
</sequence>
<sequence>
<taxon idref="PQ703625.1|InfluenzaAvirus_A/Baldeagle/nan/23-029035-005-original/2022_H5N1__segment4hemagglutinin_HA_genecompletecds|2022-11-22|4|H5N1|USA|Haliaeetusleucocephalus|USA_nan|Alphainfluenzavirus|Alphainfluenzavirusinfluenz"/>
NNN
</sequence>
<sequence>
<taxon idref="PQ703713.1|InfluenzaAvirus_A/BlackSwan/CA/22-032922-001-original/2022_H5N1__segment4hemagglutinin_HA_genecompletecds|2022-10-12|4|H5N1|USA|Cygnusatratus|USA_CA|Alphainfluenzavirus|Alphainfluenzavirusinfluenz"/>
NNN
</sequence>
<sequence>

<taxon idref="PQ705609.1|InfluenzaAvirus_A/Blackbrant/AK/22-017953-001-original/2022_H5N1__segment4hemagglutinin_HA_genecompletecds|2022-06-06|4|H5N1|USA||USA_AK|Alphainfluenzavirus|Alphainfluenzavirusinfluenz"/>
NNN
</sequence>
<sequence>
<taxon idref="PQ705617.1|InfluenzaAvirus_A/Blackbrant/AK/22-017953-002-original/2022_H5N1__segment4hemagglutinin_HA_genecompletecds|2022-06-06|4|H5N1|USA||USA_AK|Alphainfluenzavirus|Alphainfluenzavirusinfluenz"/>
NNN
</sequence>
<sequence>
<taxon idref="PQ705633.1|InfluenzaAvirus_A/Blackbrant/AK/22-023553-002-original/2022_H5N1__segment4hemagglutinin_HA_genecompletecds|2022-06-22|4|H5N1|USA||USA_AK|Alphainfluenzavirus|Alphainfluenzavirusinfluenz"/>
NNN
</sequence>
<sequence>
<taxon idref="PQ705697.1|InfluenzaAvirus_A/Blue-wingedteal/IA/22-034304-005-original-repeat2/2022_H5N1__segment4hemagglutinin_HA_genecompletecds|2022-10-22|4|H5N1|USA|Spatuladiscors|USA_IA|Alphainfluenzavirus|Alphainfluenzavirusinfluenz"/>
NNN
</sequence>
<sequence>
<taxon idref="PQ705713.1|InfluenzaAvirus_A/Blue-wingedteal/IN/22-034829-003-original-repeat2/2022_H5N1__segment4hemagglutinin_HA_genecompletecds|2022-10-28|4|H5N1|USA|Spatuladiscors|USA_IN|Alphainfluenzavirus|Alphainfluenzavirusinfluenz"/>
NNN
</sequence>
<sequence>
<taxon idref="PQ705753.1|InfluenzaAvirus_A/Blue-wingedteal/LA/22-033477-002-original/2022_H5N1__segment4hemagglutinin_HA_genecompletecds|2022-09-20|4|H5N1|USA|Spatuladiscors|USA_LA|Alphainfluenzavirus|Alphainfluenzavirusinfluenz"/>
NNN
</sequence>
<sequence>
<taxon idref="PQ705761.1|InfluenzaAvirus_A/Blue-wingedteal/LA/22-033477-003-original/2022_H5N1__segment4hemagglutinin_HA_genecompletecds|2022-09-20|4|H5N1|USA|Spatuladiscors|USA_LA|Alphainfluenzavirus|Alphainfluenzavirusinfluenz"/>
NNN
</sequence>
<sequence>
<taxon idref="PQ705769.1|InfluenzaAvirus_A/Blue-wingedteal/LA/22-033477-004-original/2022_H5N1__segment4hemagglutinin_HA_genecompletecds|2022-09-23|4|H5N1|USA|Spatuladiscors|USA_LA|Alphainfluenzavirus|Alphainfluenzavirusinfluenz"/>
NNN
</sequence>
<sequence>
<taxon idref="PQ705777.1|InfluenzaAvirus_A/Blue-wingedteal/LA/22-033477-005-original/2022_H5N1__segment4hemagglutinin_HA_genecompletecds|2022-09-23|4|H5N1|USA|Spatuladiscors|USA_LA|Alphainfluenzavirus|Alphainfluenzavirusinfluenz"/>
NNN
</sequence>

<sequence>
<taxon idref="PQ705785.1|InfluenzaAvirus_A/Blue-wingedteal/LA/22-033477-008-original/2022_H5N1__segment4hemagglutinin_HA_genecompletecds|2022-09-23|4|H5N1|USA|Spatuladiscors|USA_LA|Alphainfluenzavirus|Alphainfluenzavirus influenz"/>
NNN
</sequence>
<sequence>
<taxon idref="PQ705793.1|InfluenzaAvirus_A/Blue-wingedteal/LA/22-033477-009-original/2022_H5N1__segment4hemagglutinin_HA_genecompletecds|2022-09-23|4|H5N1|USA|Spatuladiscors|USA_LA|Alphainfluenzavirus|Alphainfluenzavirus influenz"/>
NNN
</sequence>
<sequence>
<taxon idref="PQ705817.1|InfluenzaAvirus_A/Blue-wingedteal/LA/22-033477-013-original/2022_H5N1__segment4hemagglutinin_HA_genecompletecds|2022-09-23|4|H5N1|USA|Spatuladiscors|USA_LA|Alphainfluenzavirus|Alphainfluenzavirus influenz"/>
NNN
</sequence>
<sequence>
<taxon idref="PQ705825.1|InfluenzaAvirus_A/Blue-wingedteal/LA/22-033477-014-original/2022_H5N1__segment4hemagglutinin_HA_genecompletecds|2022-09-23|4|H5N1|USA|Spatuladiscors|USA_LA|Alphainfluenzavirus|Alphainfluenzavirus influenz"/>
NNN
</sequence>
<sequence>
<taxon idref="PQ705841.1|InfluenzaAvirus_A/Blue-wingedteal/LA/22-033477-019-original/2022_H5N1__segment4hemagglutinin_HA_genecompletecds|2022-09-23|4|H5N1|USA|Spatuladiscors|USA_LA|Alphainfluenzavirus|Alphainfluenzavirus influenz"/>
NNN
</sequence>
<sequence>
<taxon idref="PQ705849.1|InfluenzaAvirus_A/Blue-wingedteal/LA/22-033477-020-original/2022_H5N1__segment4hemagglutinin_HA_genecompletecds|2022-09-23|4|H5N1|USA|Spatuladiscors|USA_LA|Alphainfluenzavirus|Alphainfluenzavirus influenz"/>
NNN
</sequence>
<sequence>
<taxon idref="PQ705865.1|InfluenzaAvirus_A/Blue-wingedteal/LA/23-013080-002-original/2022_H5N1__segment4hemagglutinin_HA_genecompletecds|2022-09-23|4|H5N1|USA|Spatuladiscors|USA_LA|Alphainfluenzavirus|Alphainfluenzavirus influenz"/>
NNN
</sequence>
<sequence>
<taxon idref="PQ705873.1|InfluenzaAvirus_A/Blue-wingedteal/LA/23-013080-004-original/2022_H5N1__segment4hemagglutinin_HA_genecompletecds|2022-09-23|4|H5N1|USA|Spatuladiscors|USA_LA|Alphainfluenzavirus|Alphainfluenzavirus influenz"/>
NNN
</sequence>
<sequence>
<taxon idref="PQ705881.1|InfluenzaAvirus_A/Blue-wingedteal/LA/23-013080-005-original/2022_H5N1__segment4hemagglutinin_HA_genecompletecds|2022-09-23|4|H5N1|USA|Spatuladiscors|USA_LA|Alphainfluenzavirus|Alphainfluenzavirus influenz"/>

NNN
</sequence>
<sequence>
<taxon idref="PQ705889.1|InfluenzaAvirus_A/Blue-wingedteal/LA/23-013080-006-original/2022_H5N1__segment4hemagglutinin_HA_genecompletecds|2022-09-23|4|H5N1|USA|Spatuladiscors|USA_LA|Alphainfluenzavirus|Alphainfluenzavirus influenz"/>
NNN
</sequence>
<sequence>
<taxon idref="PQ705897.1|InfluenzaAvirus_A/Blue-wingedteal/LA/23-013080-007-original/2022_H5N1__segment4hemagglutinin_HA_genecompletecds|2022-09-23|4|H5N1|USA|Spatuladiscors|USA_LA|Alphainfluenzavirus|Alphainfluenzavirus influenz"/>
NNN
</sequence>
<sequence>
<taxon idref="PQ705913.1|InfluenzaAvirus_A/Blue-wingedteal/LA/23-013080-009-original/2022_H5N1__segment4hemagglutinin_HA_genecompletecds|2022-09-23|4|H5N1|USA|Spatuladiscors|USA_LA|Alphainfluenzavirus|Alphainfluenzavirus influenz"/>
NNN
</sequence>
<sequence>
<taxon idref="PQ705921.1|InfluenzaAvirus_A/Blue-wingedteal/LA/23-013080-010-original/2022_H5N1__segment4hemagglutinin_HA_genecompletecds|2022-09-23|4|H5N1|USA|Spatuladiscors|USA_LA|Alphainfluenzavirus|Alphainfluenzavirus influenz"/>
NNN
</sequence>
<sequence>
<taxon idref="PQ705937.1|InfluenzaAvirus_A/Blue-wingedteal/MN/22-030423-014-original-repeat/2022_H5N1__segment4hemagglutinin_HA_genecompletecds|2022-09-10|4|H5N1|USA|Spatuladiscors|USA_MN|Alphainfluenzavirus|Alphainfluenzavirus influenz"/>
NNN
</sequence>
<sequence>
<taxon idref="PQ705945.1|InfluenzaAvirus_A/Blue-wingedteal/MN/22-030423-022-original-repeat/2022_H5N1__segment4hemagglutinin_HA_genecompletecds|2022-09-10|4|H5N1|USA|Spatuladiscors|USA_MN|Alphainfluenzavirus|Alphainfluenzavirus influenz"/>
NNN
</sequence>
<sequence>
<taxon idref="PQ705953.1|InfluenzaAvirus_A/Blue-wingedteal/MN/22-030423-047-original/2022_H5N1__segment4hemagglutinin_HA_genecompletecds|2022-09-11|4|H5N1|USA|Spatuladiscors|USA_MN|Alphainfluenzavirus|Alphainfluenzavirus influenz"/>
NNN
</sequence>
<sequence>
<taxon idref="PQ706017.1|InfluenzaAvirus_A/Blue-wingedteal/MN/23-005533-005-original/2022_H5N1__segment4hemagglutinin_HA_genecompletecds|2022-09-15|4|H5N1|USA|Spatuladiscors|USA_MN|Alphainfluenzavirus|Alphainfluenzavirus influenz"/>
NNN
</sequence>
<sequence>

<taxon idref="PQ706121.1|InfluenzaAvirus_A/Blue-wingedteal/MN/23-005533-021-original/2022_H5N1__segment4hemagglutinin_HA_genecompletecds|2022-09-15|4|H5N1|USA|Spatuladiscors|USA_MN|Alphainfluenzavirus|Alphainfluenzavirus influenz"/>
NNN
</sequence>
<sequence>
<taxon idref="PQ706161.1|InfluenzaAvirus_A/Blue-wingedteal/MN/23-005533-030-original/2022_H5N1__segment4hemagglutinin_HA_genecompletecds|2022-09-16|4|H5N1|USA|Spatuladiscors|USA_MN|Alphainfluenzavirus|Alphainfluenzavirus influenz"/>
NNN
</sequence>
<sequence>
<taxon idref="PQ706169.1|InfluenzaAvirus_A/Blue-wingedteal/MN/23-005533-031-original/2022_H5N1__segment4hemagglutinin_HA_genecompletecds|2022-09-16|4|H5N1|USA|Spatuladiscors|USA_MN|Alphainfluenzavirus|Alphainfluenzavirus influenz"/>
NNN
</sequence>
<sequence>
<taxon idref="PQ706177.1|InfluenzaAvirus_A/Blue-wingedteal/MN/23-005533-034-original/2022_H5N1__segment4hemagglutinin_HA_genecompletecds|2022-09-16|4|H5N1|USA|Spatuladiscors|USA_MN|Alphainfluenzavirus|Alphainfluenzavirus influenz"/>
NNN
</sequence>
<sequence>
<taxon idref="PQ706193.1|InfluenzaAvirus_A/Blue-wingedteal/MN/23-013078-004-original/2022_H5N1__segment4hemagglutinin_HA_genecompletecds|2022-09-13|4|H5N1|USA|Spatuladiscors|USA_MN|Alphainfluenzavirus|Alphainfluenzavirus influenz"/>
NNN
</sequence>
<sequence>
<taxon idref="PQ706209.1|InfluenzaAvirus_A/Blue-wingedteal/MN/23-013078-009-original/2022_H5N1__segment4hemagglutinin_HA_genecompletecds|2022-09-14|4|H5N1|USA|Spatuladiscors|USA_MN|Alphainfluenzavirus|Alphainfluenzavirus influenz"/>
NNN
</sequence>
<sequence>
<taxon idref="PQ706249.1|InfluenzaAvirus_A/Blue-wingedteal/MN/23-013078-018-original/2022_H5N1__segment4hemagglutinin_HA_genecompletecds|2022-09-14|4|H5N1|USA|Spatuladiscors|USA_MN|Alphainfluenzavirus|Alphainfluenzavirus influenz"/>
NNN
</sequence>
<sequence>
<taxon idref="PQ706281.1|InfluenzaAvirus_A/Blue-wingedteal/MN/23-013078-022-original/2022_H5N1__segment4hemagglutinin_HA_genecompletecds|2022-09-14|4|H5N1|USA|Spatuladiscors|USA_MN|Alphainfluenzavirus|Alphainfluenzavirus influenz"/>
NNN
</sequence>
<sequence>
<taxon idref="PQ706289.1|InfluenzaAvirus_A/Blue-wingedteal/MN/23-013078-024-original/2022_H5N1__segment4hemagglutinin_HA_genecompletecds|2022-09-14|4|H5N1|USA|Spatuladiscors|USA_MN|Alphainfluenzavirus|Alphainfluenzavirus influenz"/>
NNN

</sequence>
<sequence>
<taxon idref="PQ706297.1|InfluenzaAvirus_A/Blue-wingedteal/MN/23-013078-025-original/2022_H5N1__segment4hemagglutinin_HA_genecompletecds|2022-09-14|4|H5N1|USA|Spatuladiscors|USA_MN|Alphainfluenzavirus|Alphainfluenzavirus influenz"/>
NNN
</sequence>
<sequence>
<taxon idref="PQ706313.1|InfluenzaAvirus_A/Blue-wingedteal/MT/22-031163-004-original-repeat/2022_H5N1__segment4hemagglutinin_HA_genecompletecds|2022-09-24|4|H5N1|USA|Spatuladiscors|USA_MT|Alphainfluenzavirus|Alphainfluenzavirus influenz"/>
NNN
</sequence>
<sequence>
<taxon idref="PQ706329.1|InfluenzaAvirus_A/Blue-wingedteal/OK/22-029332-001-original/2022_H5N1__segment4hemagglutinin_HA_genecompletecds|2022-09-10|4|H5N1|USA|Spatuladiscors|USA_OK|Alphainfluenzavirus|Alphainfluenzavirus influenz"/>
NNN
</sequence>
<sequence>
<taxon idref="PQ706337.1|InfluenzaAvirus_A/Blue-wingedteal/OK/22-029332-004-original/2022_H5N1__segment4hemagglutinin_HA_genecompletecds|2022-09-10|4|H5N1|USA|Spatuladiscors|USA_OK|Alphainfluenzavirus|Alphainfluenzavirus influenz"/>
NNN
</sequence>
<sequence>
<taxon idref="PQ706345.1|InfluenzaAvirus_A/Blue-wingedteal/OK/22-029332-005-original/2022_H5N1__segment4hemagglutinin_HA_genecompletecds|2022-09-10|4|H5N1|USA|Spatuladiscors|USA_OK|Alphainfluenzavirus|Alphainfluenzavirus influenz"/>
NNN
</sequence>
<sequence>
<taxon idref="PQ706353.1|InfluenzaAvirus_A/Blue-wingedteal/OK/22-029332-006-original/2022_H5N1__segment4hemagglutinin_HA_genecompletecds|2022-09-10|4|H5N1|USA|Spatuladiscors|USA_OK|Alphainfluenzavirus|Alphainfluenzavirus influenz"/>
NNN
</sequence>
<sequence>
<taxon idref="PQ706361.1|InfluenzaAvirus_A/Blue-wingedteal/OK/22-029332-008-original/2022_H5N1__segment4hemagglutinin_HA_genecompletecds|2022-09-10|4|H5N1|USA|Spatuladiscors|USA_OK|Alphainfluenzavirus|Alphainfluenzavirus influenz"/>
NNN
</sequence>
<sequence>
<taxon idref="PQ706369.1|InfluenzaAvirus_A/Blue-wingedteal/OK/22-029332-011-original/2022_H5N1__segment4hemagglutinin_HA_genecompletecds|2022-09-10|4|H5N1|USA|Spatuladiscors|USA_OK|Alphainfluenzavirus|Alphainfluenzavirus influenz"/>
NNN
</sequence>
<sequence>
<taxon idref="PQ706377.1|InfluenzaAvirus_A/Blue-wingedteal/OK/22-029332-018-original/2022_H5N1__segment4hemagglutinin_HA_genecompletecds|2022-09-

10|4|H5N1|USA|Spatuladiscors|USA_OK|Alphainfluenzavirus|Alphainfluenzavirus influenz"/>
NNN
</sequence>
<sequence>
<taxon idref="PQ706385.1|InfluenzaAvirus_A/Blue-wingedteal/OK/22-029332-019-original/2022_H5N1__segment4hemagglutinin_HA_genecompletecds|2022-09-10|4|H5N1|USA|Spatuladiscors|USA_OK|Alphainfluenzavirus|Alphainfluenzavirus influenz"/>
NNN
</sequence>
<sequence>
<taxon idref="PQ706393.1|InfluenzaAvirus_A/Blue-wingedteal/OK/22-029332-023-original/2022_H5N1__segment4hemagglutinin_HA_genecompletecds|2022-09-10|4|H5N1|USA|Spatuladiscors|USA_OK|Alphainfluenzavirus|Alphainfluenzavirus influenz"/>
NNN
</sequence>
<sequence>
<taxon idref="PQ706417.1|InfluenzaAvirus_A/Blue-wingedteal/OK/22-029332-034-original/2022_H5N1__segment4hemagglutinin_HA_genecompletecds|2022-09-10|4|H5N1|USA|Spatuladiscors|USA_OK|Alphainfluenzavirus|Alphainfluenzavirus influenz"/>
NNN
</sequence>
<sequence>
<taxon idref="PQ706433.1|InfluenzaAvirus_A/Blue-wingedteal/TX/22-031350-016-original-repeat/2022_H5N1__segment4hemagglutinin_HA_genecompletecds|2022-09-10|4|H5N1|USA|Spatuladiscors|USA_TX|Alphainfluenzavirus|Alphainfluenzavirus influenz"/>
NNN
</sequence>
<sequence>
<taxon idref="PQ706441.1|InfluenzaAvirus_A/Blue-wingedteal/TX/22-031351-010-original-repeat/2022_H5N1__segment4hemagglutinin_HA_genecompletecds|2022-09-17|4|H5N1|USA|Spatuladiscors|USA_TX|Alphainfluenzavirus|Alphainfluenzavirus influenz"/>
NNN
</sequence>
<sequence>
<taxon idref="PQ706449.1|InfluenzaAvirus_A/Blue-wingedteal/TX/22-031352-026-original-repeat/2022_H5N1__segment4hemagglutinin_HA_genecompletecds|2022-09-24|4|H5N1|USA|Spatuladiscors|USA_TX|Alphainfluenzavirus|Alphainfluenzavirus influenz"/>
NNN
</sequence>
<sequence>
<taxon idref="PQ706457.1|InfluenzaAvirus_A/Blue-wingedteal/TX/22-031352-027-original-repeat/2022_H5N1__segment4hemagglutinin_HA_genecompletecds|2022-09-24|4|H5N1|USA|Spatuladiscors|USA_TX|Alphainfluenzavirus|Alphainfluenzavirus influenz"/>
NNN
</sequence>
<sequence>
<taxon idref="PQ706465.1|InfluenzaAvirus_A/Blue-wingedteal/TX/22-031395-009-original-repeat/2022_H5N1__segment4hemagglutinin_HA_genecompletecds|2022-09-

24|4|H5N1|USA|Spatuladiscors|USA_TX|Alphainfluenzavirus|Alphainfluenzavirus influenz"/>
NNN
</sequence>
<sequence>
<taxon idref="PQ706481.1|InfluenzaAvirus_A/Blue-wingedteal/TX/22-033479-002-original/2022_H5N1__segment4hemagglutinin_HA_genecompletecds|2022-09-22|4|H5N1|USA|Spatuladiscors|USA_TX|Alphainfluenzavirus|Alphainfluenzavirus influenz"/>
NNN
</sequence>
<sequence>
<taxon idref="PQ706505.1|InfluenzaAvirus_A/Blue-wingedteal/TX/22-033479-007-original/2022_H5N1__segment4hemagglutinin_HA_genecompletecds|2022-09-22|4|H5N1|USA|Spatuladiscors|USA_TX|Alphainfluenzavirus|Alphainfluenzavirus influenz"/>
NNN
</sequence>
<sequence>
<taxon idref="PQ706513.1|InfluenzaAvirus_A/Blue-wingedteal/TX/22-033479-008-original/2022_H5N1__segment4hemagglutinin_HA_genecompletecds|2022-09-22|4|H5N1|USA|Spatuladiscors|USA_TX|Alphainfluenzavirus|Alphainfluenzavirus influenz"/>
NNN
</sequence>
<sequence>
<taxon idref="PQ706521.1|InfluenzaAvirus_A/Blue-wingedteal/TX/22-033479-009-original/2022_H5N1__segment4hemagglutinin_HA_genecompletecds|2022-09-22|4|H5N1|USA|Spatuladiscors|USA_TX|Alphainfluenzavirus|Alphainfluenzavirus influenz"/>
NNN
</sequence>
<sequence>
<taxon idref="PQ706529.1|InfluenzaAvirus_A/Blue-wingedteal/TX/22-033479-010-original/2022_H5N1__segment4hemagglutinin_HA_genecompletecds|2022-09-22|4|H5N1|USA|Spatuladiscors|USA_TX|Alphainfluenzavirus|Alphainfluenzavirus influenz"/>
NNN
</sequence>
<sequence>
<taxon idref="PQ706537.1|InfluenzaAvirus_A/Blue-wingedteal/TX/22-033479-011-original/2022_H5N1__segment4hemagglutinin_HA_genecompletecds|2022-09-22|4|H5N1|USA|Spatuladiscors|USA_TX|Alphainfluenzavirus|Alphainfluenzavirus influenz"/>
NNN
</sequence>
<sequence>
<taxon idref="PQ706561.1|InfluenzaAvirus_A/Blue-wingedteal/TX/22-033479-014-original/2022_H5N1__segment4hemagglutinin_HA_genecompletecds|2022-09-22|4|H5N1|USA|Spatuladiscors|USA_TX|Alphainfluenzavirus|Alphainfluenzavirus influenz"/>
NNN
</sequence>
<sequence>
<taxon idref="PQ706577.1|InfluenzaAvirus_A/Blue-wingedteal/TX/22-033479-017-original/2022_H5N1__segment4hemagglutinin_HA_genecompletecds|2022-09-24|4|H5N1|USA|Spatuladiscors|USA_TX|Alphainfluenzavirus|Alphainfluenzavirus influenz"/>
NNN
</sequence>
<sequence>

<taxon idref="PQ706585.1|InfluenzaAvirus_A/Blue-wingedteal/TX/22-033479-018-original/2022_H5N1__segment4hemagglutinin_HA_genecompletecds|2022-09-24|4|H5N1|USA|Spatuladiscors|USA_TX|Alphainfluenzavirus|Alphainfluenzavirus influenz"/>
NNN
</sequence>
<sequence>
<taxon idref="PQ706593.1|InfluenzaAvirus_A/Blue-wingedteal/TX/22-033479-019-original/2022_H5N1__segment4hemagglutinin_HA_genecompletecds|2022-09-24|4|H5N1|USA|Spatuladiscors|USA_TX|Alphainfluenzavirus|Alphainfluenzavirus influenz"/>
NNN
</sequence>
<sequence>
<taxon idref="PQ706601.1|InfluenzaAvirus_A/Blue-wingedteal/TX/22-033479-020-original/2022_H5N1__segment4hemagglutinin_HA_genecompletecds|2022-09-24|4|H5N1|USA|Spatuladiscors|USA_TX|Alphainfluenzavirus|Alphainfluenzavirus influenz"/>
NNN
</sequence>
<sequence>
<taxon idref="PQ706609.1|InfluenzaAvirus_A/Blue-wingedteal/TX/22-033479-021-original/2022_H5N1__segment4hemagglutinin_HA_genecompletecds|2022-09-24|4|H5N1|USA|Spatuladiscors|USA_TX|Alphainfluenzavirus|Alphainfluenzavirus influenz"/>
NNN
</sequence>
<sequence>
<taxon idref="PQ706617.1|InfluenzaAvirus_A/Blue-wingedteal/TX/22-033479-023-original/2022_H5N1__segment4hemagglutinin_HA_genecompletecds|2022-09-24|4|H5N1|USA|Spatuladiscors|USA_TX|Alphainfluenzavirus|Alphainfluenzavirus influenz"/>
NNN
</sequence>
<sequence>
<taxon idref="PQ706625.1|InfluenzaAvirus_A/Blue-wingedteal/TX/22-033479-024-original/2022_H5N1__segment4hemagglutinin_HA_genecompletecds|2022-09-24|4|H5N1|USA|Spatuladiscors|USA_TX|Alphainfluenzavirus|Alphainfluenzavirus influenz"/>
NNN
</sequence>
<sequence>
<taxon idref="PQ706633.1|InfluenzaAvirus_A/Blue-wingedteal/TX/22-033479-025-original/2022_H5N1__segment4hemagglutinin_HA_genecompletecds|2022-09-24|4|H5N1|USA|Spatuladiscors|USA_TX|Alphainfluenzavirus|Alphainfluenzavirus influenz"/>
NNN
</sequence>
<sequence>
<taxon idref="PQ706641.1|InfluenzaAvirus_A/Blue-wingedteal/TX/22-033479-026-original/2022_H5N1__segment4hemagglutinin_HA_genecompletecds|2022-09-24|4|H5N1|USA|Spatuladiscors|USA_TX|Alphainfluenzavirus|Alphainfluenzavirus influenz"/>
NNN
</sequence>
<sequence>
<taxon idref="PQ706649.1|InfluenzaAvirus_A/Blue-wingedteal/TX/22-033479-028-original/2022_H5N1__segment4hemagglutinin_HA_genecompletecds|2022-09-24|4|H5N1|USA|Spatuladiscors|USA_TX|Alphainfluenzavirus|Alphainfluenzavirus influenz"/>
NNN

</sequence>
<sequence>
<taxon idref="PQ706665.1|InfluenzaAvirus_A/Blue-wingedteal/TX/22-041132-005-original/2022_H5N1__segment4hemagglutinin_HA_genecompletecds|2022-09-20|4|H5N1|USA|Spatuladiscors|USA_TX|Alphainfluenzavirus|Alphainfluenzavirus influenz"/>
NNN
</sequence>
<sequence>
<taxon idref="PQ706673.1|InfluenzaAvirus_A/Blue-wingedteal/TX/22-041132-007-original/2022_H5N1__segment4hemagglutinin_HA_genecompletecds|2022-09-20|4|H5N1|USA|Spatuladiscors|USA_TX|Alphainfluenzavirus|Alphainfluenzavirus influenz"/>
NNN
</sequence>
<sequence>
<taxon idref="PQ706801.1|InfluenzaAvirus_A/CacklingGoose/AK/22-039438-003-original/2022_H5N1__segment4hemagglutinin_HA_genecompletecds|2022-09-05|4|H5N1|USA|Brantahutchinsii|USA_AK|Alphainfluenzavirus|Alphainfluenzavirusinfluenz"/>
NNN
</sequence>
<sequence>
<taxon idref="PQ706809.1|InfluenzaAvirus_A/CacklingGoose/AK/22-039438-004-original/2022_H5N1__segment4hemagglutinin_HA_genecompletecds|2022-09-05|4|H5N1|USA|Brantahutchinsii|USA_AK|Alphainfluenzavirus|Alphainfluenzavirusinfluenz"/>
NNN
</sequence>
<sequence>
<taxon idref="PQ706817.1|InfluenzaAvirus_A/CacklingGoose/AK/22-039438-012-original/2022_H5N1__segment4hemagglutinin_HA_genecompletecds|2022-09-09|4|H5N1|USA|Brantahutchinsii|USA_AK|Alphainfluenzavirus|Alphainfluenzavirusinfluenz"/>
NNN
</sequence>
<sequence>
<taxon idref="PQ706825.1|InfluenzaAvirus_A/CacklingGoose/AK/23-002693-023-original/2022_H5N1__segment4hemagglutinin_HA_genecompletecds|2022-09-30|4|H5N1|USA|Brantahutchinsii|USA_AK|Alphainfluenzavirus|Alphainfluenzavirusinfluenz"/>
NNN
</sequence>
<sequence>
<taxon idref="PQ706905.1|InfluenzaAvirus_A/CacklingGoose/OR/22-036878-001-original-repeat2/2022_H5N1__segment4hemagglutinin_HA_genecompletecds|2022-10-02|4|H5N1|USA|Brantahutchinsii|USA_OR|Alphainfluenzavirus|Alphainfluenzavirusinfluenz"/>
NNN
</sequence>
<sequence>
<taxon idref="PQ706913.1|InfluenzaAvirus_A/CacklingGoose/OR/22-037259-001-original-repeat2/2022_H5N1__segment4hemagglutinin_HA_genecompletecds|2022-11-14|4|H5N1|USA|Brantahutchinsii|USA_OR|Alphainfluenzavirus|Alphainfluenzavirusinfluenz"/>
NNN
</sequence>
<sequence>

<taxon idref="PQ706929.1|InfluenzaAvirus_A/CacklingGoose/OR/22-037263-001-original/2022_H5N1__segment4hemagglutinin_HA_genecompletecds|2022-11-10|4|H5N1|USA|Brantahutchinsii|USA_OR|Alphainfluenzavirus|Alphainfluenzavirusinfluenz"/>
NNN
</sequence>
<sequence>
<taxon idref="PQ706945.1|InfluenzaAvirus_A/CacklingGoose/WA/22-037657-001-original/2022_H5N1__segment4hemagglutinin_HA_genecompletecds|2022-11-16|4|H5N1|USA|Brantahutchinsii|USA_WA|Alphainfluenzavirus|Alphainfluenzavirusinfluenz"/>
NNN
</sequence>
<sequence>
<taxon idref="PQ707057.1|InfluenzaAvirus_A/Canadagoose/AK/22-014435-001-original/2022_H5N1__segment4hemagglutinin_HA_genecompletecds|2022-05-05|4|H5N1|USA|Brantacanadensis|USA_AK|Alphainfluenzavirus|Alphainfluenzavirusinfluenz"/>
NNN
</sequence>
<sequence>
<taxon idref="PQ707089.1|InfluenzaAvirus_A/Canadagoose/AZ/22-034128-001-original-repeat/2022_H5N1__segment4hemagglutinin_HA_genecompletecds|2022-10-17|4|H5N1|USA|Brantacanadensis|USA_AZ|Alphainfluenzavirus|Alphainfluenzavirusinfluenz"/>
NNN
</sequence>
<sequence>
<taxon idref="PQ707529.1|InfluenzaAvirus_A/Canadagoose/CO/22-033695-001-original/2022_H5N1__segment4hemagglutinin_HA_genecompletecds|2022-10-18|4|H5N1|USA|Brantacanadensis|USA_CO|Alphainfluenzavirus|Alphainfluenzavirusinfluenz"/>
NNN
</sequence>
<sequence>
<taxon idref="PQ707545.1|InfluenzaAvirus_A/Canadagoose/CO/22-038655-001-original/2022_H5N1__segment4hemagglutinin_HA_genecompletecds|2022-11-28|4|H5N1|USA|Brantacanadensis|USA_CO|Alphainfluenzavirus|Alphainfluenzavirusinfluenz"/>
NNN
</sequence>
<sequence>
<taxon idref="PQ707801.1|InfluenzaAvirus_A/Canadagoose/ID/22-014968-006-original/2022_H5N1__segment4hemagglutinin_HA_genecompletecds|2022-05-10|4|H5N1|USA|Brantacanadensis|USA_ID|Alphainfluenzavirus|Alphainfluenzavirusinfluenz"/>
NNN
</sequence>
<sequence>
<taxon idref="PQ707833.1|InfluenzaAvirus_A/Canadagoose/ID/22-017360-002-original/2022_H5N1__segment4hemagglutinin_HA_genecompletecds|2022-05-31|4|H5N1|USA|Brantacanadensis|USA_ID|Alphainfluenzavirus|Alphainfluenzavirusinfluenz"/>
NNN
</sequence>
<sequence>
<taxon idref="PQ707841.1|InfluenzaAvirus_A/Canadagoose/ID/22-018980-001-original/2022_H5N1__segment4hemagglutinin_HA_genecompletecds|2022-06-04|4|H5N1|USA|Brantacanadensis|USA_ID|Alphainfluenzavirus|Alphainfluenzavirusinfluenz"/>

NNN
</sequence>
<sequence>
<taxon idref="PQ708265.1|InfluenzaAvirus_A/Canadagoose/MT/22-033859-002-original/2022_H5N1__segment4hemagglutinin_HA_genecompletecds|2022-10-20|4|H5N1|USA|Brantacanadensis|USA_MT|Alphainfluenzavirus|Alphainfluenzavirusinfluenz"/>
NNN
</sequence>
<sequence>
<taxon idref="PQ708281.1|InfluenzaAvirus_A/Canadagoose/NC/22-036623-001-original-repeat2/2022_H5N1__segment4hemagglutinin_HA_genecompletecds|2022-10-30|4|H5N1|USA|Brantacanadensis|USA_NC|Alphainfluenzavirus|Alphainfluenzavirusinfluenz"/>
NNN
</sequence>
<sequence>
<taxon idref="PQ708313.1|InfluenzaAvirus_A/Canadagoose/ND/23-002238-004-original/2022_H5N1__segment4hemagglutinin_HA_genecompletecds|2022-11-29|4|H5N1|USA|Brantacanadensis|USA_ND|Alphainfluenzavirus|Alphainfluenzavirusinfluenz"/>
NNN
</sequence>
<sequence>
<taxon idref="PQ708353.1|InfluenzaAvirus_A/Canadagoose/NV/22-033403-003-original-repeat/2022_H5N1__segment4hemagglutinin_HA_genecompletecds|2022-09-23|4|H5N1|USA|Brantacanadensis|USA_NV|Alphainfluenzavirus|Alphainfluenzavirusinfluenz"/>
NNN
</sequence>
<sequence>
<taxon idref="PQ708697.1|InfluenzaAvirus_A/Canadagoose/OR/22-037260-001-original-repeat/2022_H5N1__segment4hemagglutinin_HA_genecompletecds|2022-11-02|4|H5N1|USA|Brantacanadensis|USA_OR|Alphainfluenzavirus|Alphainfluenzavirusinfluenz"/>
NNN
</sequence>
<sequence>
<taxon idref="PQ708705.1|InfluenzaAvirus_A/Canadagoose/OR/22-037722-001-original/2022_H5N1__segment4hemagglutinin_HA_genecompletecds|2022-11-17|4|H5N1|USA|Brantacanadensis|USA_OR|Alphainfluenzavirus|Alphainfluenzavirusinfluenz"/>
NNN
</sequence>
<sequence>
<taxon idref="PQ708929.1|InfluenzaAvirus_A/Canadagoose/WA/22-015526-002-original/2022_H5N1__segment4hemagglutinin_HA_genecompletecds|2022-05-11|4|H5N1|USA|Brantacanadensis|USA_WA|Alphainfluenzavirus|Alphainfluenzavirusinfluenz"/>
NNN
</sequence>
<sequence>
<taxon idref="PQ708937.1|InfluenzaAvirus_A/Canadagoose/WA/22-015526-003-original/2022_H5N1__segment4hemagglutinin_HA_genecompletecds|2022-05-11|4|H5N1|USA|Brantacanadensis|USA_WA|Alphainfluenzavirus|Alphainfluenzavirusinfluenz"/>
NNN
</sequence>

<sequence>
<taxon idref="PQ708945.1|InfluenzaAvirus_A/Canadagoose/WA/22-015526-005-original/2022_H5N1__segment4hemagglutinin_HA_genecompletecds|2022-05-09|4|H5N1|USA|Brantacanadensis|USA_WA|Alphainfluenzavirus|Alphainfluenzavirusinfluenz"/>
NNN
</sequence>
<sequence>
<taxon idref="PQ709145.1|InfluenzaAvirus_A/Canadagoose/WY/22-033366-001-original/2022_H5N1__segment4hemagglutinin_HA_genecompletecds|2022-10-14|4|H5N1|USA|Brantacanadensis|USA_WY|Alphainfluenzavirus|Alphainfluenzavirusinfluenz"/>
NNN
</sequence>
<sequence>
<taxon idref="PQ709153.1|InfluenzaAvirus_A/Canadagoose/WY/22-036111-001-original/2022_H5N1__segment4hemagglutinin_HA_genecompletecds|2022-11-03|4|H5N1|USA|Brantacanadensis|USA_WY|Alphainfluenzavirus|Alphainfluenzavirusinfluenz"/>
NNN
</sequence>
<sequence>
<taxon idref="PQ709201.1|InfluenzaAvirus_A/Canadagoose/WY/23-000336-002-original/2022_H5N1__segment4hemagglutinin_HA_genecompletecds|2022-10-30|4|H5N1|USA|Brantacanadensis|USA_WY|Alphainfluenzavirus|Alphainfluenzavirusinfluenz"/>
NNN
</sequence>
<sequence>
<taxon idref="PQ709657.1|InfluenzaAvirus_A/Cormorant/CA/22-037516-001-original/2022_H5N1__segment4hemagglutinin_HA_genecompletecds|2022-11-06|4|H5N1|USA|Phalacrocoracidae|USA_CA|Alphainfluenzavirus|Alphainfluenzavirusinfluenz"/>
NNN
</sequence>
<sequence>
<taxon idref="PQ709665.1|InfluenzaAvirus_A/Cormorant/FL/22-032961-001-original/2022_H5N1__segment4hemagglutinin_HA_genecompletecds|2022-10-12|4|H5N1|USA|Phalacrocoracidae|USA_FL|Alphainfluenzavirus|Alphainfluenzavirusinfluenz"/>
NNN
</sequence>
<sequence>
<taxon idref="PQ709697.1|InfluenzaAvirus_A/Double-crestedCormorant/FL/22-036552-001-original/2022_H5N1__segment4hemagglutinin_HA_genecompletecds|2022-11-03|4|H5N1|USA|Phalacrocoraxauritus|USA_FL|Alphainfluenzavirus|Alphainfluenzavirusinfluenz"/>
NNN
</sequence>
<sequence>
<taxon idref="PQ709841.1|InfluenzaAvirus_A/Duck/ID/22-014968-005-original/2022_H5N1__segment4hemagglutinin_HA_genecompletecds|2022-05-09|4|H5N1|USA|Anatidae|USA_ID|Alphainfluenzavirus|Alphainfluenzavirusinfluenz"/>
NNN
</sequence>
<sequence>
<taxon idref="PQ709897.1|InfluenzaAvirus_A/Duck/UT/22-031728-004-original-repeat/2022_H5N1__segment4hemagglutinin_HA_genecompletecds|2022-10-

04|4|H5N1|USA|Anatidae|USA_UT|Alphainfluenzavirus|Alphainfluenzavirusinflue
nz"/>
NNN
</sequence>
<sequence>
<taxon idref="PQ709905.1|InfluenzaAvirus_A/Duck/UT/22-031728-005-
original/2022_H5N1__segment4hemagglutinin_HA_genecompletecds|2022-10-
04|4|H5N1|USA|Anatidae|USA_UT|Alphainfluenzavirus|Alphainfluenzavirusinflue
nz"/>
NNN
</sequence>
<sequence>
<taxon idref="PQ709921.1|InfluenzaAvirus_A/Dunlin/WA/22-039678-002-
original/2022_H5N1__segment4hemagglutinin_HA_genecompletecds|2022-11-
21|4|H5N1|USA|Calidrisalpina|USA_WA|Alphainfluenzavirus|Alphainfluenzavirus
influenz"/>
NNN
</sequence>
<sequence>
<taxon idref="PQ710001.1|InfluenzaAvirus_A/Earedgrebe/UT/22-030915-001-
original/2022_H5N1__segment4hemagglutinin_HA_genecompletecds|2022-09-
27|4|H5N1|USA||USA_UT|Alphainfluenzavirus|Alphainfluenzavirusinfluenz"/>
NNN
</sequence>
<sequence>
<taxon idref="PQ710009.1|InfluenzaAvirus_A/Earedgrebe/UT/22-030915-002-
original/2022_H5N1__segment4hemagglutinin_HA_genecompletecds|2022-09-
27|4|H5N1|USA||USA_UT|Alphainfluenzavirus|Alphainfluenzavirusinfluenz"/>
NNN
</sequence>
<sequence>
<taxon idref="PQ710017.1|InfluenzaAvirus_A/Earedgrebe/UT/22-030915-003-
original/2022_H5N1__segment4hemagglutinin_HA_genecompletecds|2022-09-
27|4|H5N1|USA||USA_UT|Alphainfluenzavirus|Alphainfluenzavirusinfluenz"/>
NNN
</sequence>
<sequence>
<taxon idref="PQ710081.1|InfluenzaAvirus_A/Gadwall/AK/23-002693-002-
original/2022_H5N1__segment4hemagglutinin_HA_genecompletecds|2022-09-
20|4|H5N1|USA|Marecastrepera|USA_AK|Alphainfluenzavirus|Alphainfluenzavirus
influenz"/>
NNN
</sequence>
<sequence>
<taxon idref="PQ710089.1|InfluenzaAvirus_A/Gadwall/AK/23-002693-003-
original/2022_H5N1__segment4hemagglutinin_HA_genecompletecds|2022-09-
20|4|H5N1|USA|Marecastrepera|USA_AK|Alphainfluenzavirus|Alphainfluenzavirus
influenz"/>
NNN
</sequence>
<sequence>
<taxon idref="PQ710209.1|InfluenzaAvirus_A/Gadwall/LA/22-037240-002-
original-repeat/2022_H5N1__segment4hemagglutinin_HA_genecompletecds|2022-
11-
13|4|H5N1|USA|Marecastrepera|USA_LA|Alphainfluenzavirus|Alphainfluenzavirus
influenz"/>
NNN
</sequence>
<sequence>
<taxon idref="PQ710337.1|InfluenzaAvirus_A/Gadwall/TX/22-036753-004-
original-repeat/2022_H5N1__segment4hemagglutinin_HA_genecompletecds|2022-

11-
05|4|H5N1|USA|Marecastrepera|USA_TX|Alphainfluenzavirus|Alphainfluenzavirus influenz"/>
NNN
</sequence>
<sequence>
<taxon idref="PQ710345.1|InfluenzaAvirus_A/Gadwall/TX/22-036753-021-original-repeat/2022_H5N1__segment4hemagglutinin_HA_genecompletecds|2022-11-
05|4|H5N1|USA|Marecastrepera|USA_TX|Alphainfluenzavirus|Alphainfluenzavirus influenz"/>
NNN
</sequence>
<sequence>
<taxon idref="PQ710353.1|InfluenzaAvirus_A/Gadwall/TX/22-036753-040-original-repeat/2022_H5N1__segment4hemagglutinin_HA_genecompletecds|2022-11-
05|4|H5N1|USA|Marecastrepera|USA_TX|Alphainfluenzavirus|Alphainfluenzavirus influenz"/>
NNN
</sequence>
<sequence>
<taxon idref="PQ710361.1|InfluenzaAvirus_A/Gadwall/TX/22-036753-054-original-repeat/2022_H5N1__segment4hemagglutinin_HA_genecompletecds|2022-11-
05|4|H5N1|USA|Marecastrepera|USA_TX|Alphainfluenzavirus|Alphainfluenzavirus influenz"/>
NNN
</sequence>
<sequence>
<taxon idref="PQ710369.1|InfluenzaAvirus_A/Gadwall/TX/22-036753-074-original/2022_H5N1__segment4hemagglutinin_HA_genecompletecds|2022-11-
05|4|H5N1|USA|Marecastrepera|USA_TX|Alphainfluenzavirus|Alphainfluenzavirus influenz"/>
NNN
</sequence>
<sequence>
<taxon idref="PQ710377.1|InfluenzaAvirus_A/Gadwall/TX/22-036753-075-original-repeat/2022_H5N1__segment4hemagglutinin_HA_genecompletecds|2022-11-
05|4|H5N1|USA|Marecastrepera|USA_TX|Alphainfluenzavirus|Alphainfluenzavirus influenz"/>
NNN
</sequence>
<sequence>
<taxon idref="PQ710385.1|InfluenzaAvirus_A/Gadwall/TX/22-036753-082-original-repeat/2022_H5N1__segment4hemagglutinin_HA_genecompletecds|2022-11-
05|4|H5N1|USA|Marecastrepera|USA_TX|Alphainfluenzavirus|Alphainfluenzavirus influenz"/>
NNN
</sequence>
<sequence>
<taxon idref="PQ710393.1|InfluenzaAvirus_A/Gadwall/TX/22-036754-036-original/2022_H5N1__segment4hemagglutinin_HA_genecompletecds|2022-11-
06|4|H5N1|USA|Marecastrepera|USA_TX|Alphainfluenzavirus|Alphainfluenzavirus influenz"/>
NNN
</sequence>
<sequence>

<taxon idref="PQ710401.1|InfluenzaAvirus_A/Gadwall/TX/22-036754-046-original/2022_H5N1__segment4hemagglutinin_HA_genecompletecds|2022-11-06|4|H5N1|USA|Marecastrepera|USA_TX|Alphainfluenzavirus|Alphainfluenzavirus influenz"/>
NNN
</sequence>
<sequence>
<taxon idref="PQ710409.1|InfluenzaAvirus_A/Gadwall/TX/22-036754-057-original/2022_H5N1__segment4hemagglutinin_HA_genecompletecds|2022-11-06|4|H5N1|USA|Marecastrepera|USA_TX|Alphainfluenzavirus|Alphainfluenzavirus influenz"/>
NNN
</sequence>
<sequence>
<taxon idref="PQ710417.1|InfluenzaAvirus_A/Gadwall/TX/22-036754-066-original/2022_H5N1__segment4hemagglutinin_HA_genecompletecds|2022-11-06|4|H5N1|USA|Marecastrepera|USA_TX|Alphainfluenzavirus|Alphainfluenzavirus influenz"/>
NNN
</sequence>
<sequence>
<taxon idref="PQ710465.1|InfluenzaAvirus_A/GlaucousGull/AK/22-017953-003-original/2022_H5N1__segment4hemagglutinin_HA_genecompletecds|2022-06-06|4|H5N1|USA|Larushyperboreus|USA_AK|Alphainfluenzavirus|Alphainfluenzavirusinfluenz"/>
NNN
</sequence>
<sequence>
<taxon idref="PQ710473.1|InfluenzaAvirus_A/GlaucousGull/AK/22-017953-004-original/2022_H5N1__segment4hemagglutinin_HA_genecompletecds|2022-06-06|4|H5N1|USA|Larushyperboreus|USA_AK|Alphainfluenzavirus|Alphainfluenzavirusinfluenz"/>
NNN
</sequence>
<sequence>
<taxon idref="PQ710481.1|InfluenzaAvirus_A/GlaucousGull/AK/22-017953-009-original/2022_H5N1__segment4hemagglutinin_HA_genecompletecds|2022-06-06|4|H5N1|USA|Larushyperboreus|USA_AK|Alphainfluenzavirus|Alphainfluenzavirusinfluenz"/>
NNN
</sequence>
<sequence>
<taxon idref="PQ710489.1|InfluenzaAvirus_A/GlaucousGull/AK/22-017953-010-original/2022_H5N1__segment4hemagglutinin_HA_genecompletecds|2022-06-06|4|H5N1|USA|Larushyperboreus|USA_AK|Alphainfluenzavirus|Alphainfluenzavirusinfluenz"/>
NNN
</sequence>
<sequence>
<taxon idref="PQ710497.1|InfluenzaAvirus_A/GlaucousGull/AK/22-018101-002-original/2022_H5N1__segment4hemagglutinin_HA_genecompletecds|2022-06-06|4|H5N1|USA|Larushyperboreus|USA_AK|Alphainfluenzavirus|Alphainfluenzavirusinfluenz"/>
NNN
</sequence>
<sequence>
<taxon idref="PQ710505.1|InfluenzaAvirus_A/GlaucousGull/AK/22-021855-001-original/2022_H5N1__segment4hemagglutinin_HA_genecompletecds|2022-05-31|4|H5N1|USA|Larushyperboreus|USA_AK|Alphainfluenzavirus|Alphainfluenzavirusinfluenz"/>
NNN

</sequence>
<sequence>
<taxon idref="PQ710529.1|InfluenzaAvirus_A/GlaucousGull/AK/22-024502-001-original/2022_H5N1__segment4hemagglutinin_HA_genecompletecds|2022-07-01|4|H5N1|USA|Larushyperboreus|USA_AK|Alphainfluenzavirus|Alphainfluenzavirusinfluenz"/>
NNN
</sequence>
<sequence>
<taxon idref="PQ710801.1|InfluenzaAvirus_A/Greathornedowl/CA/22-035595-002-original/2022_H5N1__segment4hemagglutinin_HA_genecompletecds|2022-10-27|4|H5N1|USA|Bubovirginianus|USA_CA|Alphainfluenzavirus|Alphainfluenzavirusinfluenz"/>
NNN
</sequence>
<sequence>
<taxon idref="PQ710897.1|InfluenzaAvirus_A/Greathornedowl/CO/22-033694-001-original/2022_H5N1__segment4hemagglutinin_HA_genecompletecds|2022-10-18|4|H5N1|USA|Bubovirginianus|USA_CO|Alphainfluenzavirus|Alphainfluenzavirusinfluenz"/>
NNN
</sequence>
<sequence>
<taxon idref="PQ710913.1|InfluenzaAvirus_A/Greathornedowl/CO/22-038652-003-original/2022_H5N1__segment4hemagglutinin_HA_genecompletecds|2022-11-22|4|H5N1|USA|Bubovirginianus|USA_CO|Alphainfluenzavirus|Alphainfluenzavirusinfluenz"/>
NNN
</sequence>
<sequence>
<taxon idref="PQ711169.1|InfluenzaAvirus_A/Greathornedowl/KS/22-040466-001-original/2022_H5N1__segment4hemagglutinin_HA_genecompletecds|2022-11-29|4|H5N1|USA|Bubovirginianus|USA_KS|Alphainfluenzavirus|Alphainfluenzavirusinfluenz"/>
NNN
</sequence>
<sequence>
<taxon idref="PQ711673.1|InfluenzaAvirus_A/Greathornedowl/ND/22-032264-001-original/2022_H5N1__segment4hemagglutinin_HA_genecompletecds|2022-09-28|4|H5N1|USA|Bubovirginianus|USA_ND|Alphainfluenzavirus|Alphainfluenzavirusinfluenz"/>
NNN
</sequence>
<sequence>
<taxon idref="PQ711681.1|InfluenzaAvirus_A/Greathornedowl/NE/22-038800-001-original/2022_H5N1__segment4hemagglutinin_HA_genecompletecds|2022-11-30|4|H5N1|USA|Bubovirginianus|USA_NE|Alphainfluenzavirus|Alphainfluenzavirusinfluenz"/>
NNN
</sequence>
<sequence>
<taxon idref="PQ711745.1|InfluenzaAvirus_A/Greathornedowl/OR/22-033001-001-original/2022_H5N1__segment4hemagglutinin_HA_genecompletecds|2022-10-11|4|H5N1|USA|Bubovirginianus|USA_OR|Alphainfluenzavirus|Alphainfluenzavirusinfluenz"/>
NNN
</sequence>
<sequence>
<taxon idref="PQ711849.1|InfluenzaAvirus_A/Greathornedowl/UT/22-032952-008-original/2022_H5N1__segment4hemagglutinin_HA_genecompletecds|2022-10-

13|4|H5N1|USA|Bubovirginianus|USA_UT|Alphainfluenzavirus|Alphainfluenzaviru
sinfluenz"/>
NNN
</sequence>
<sequence>
<taxon idref="PQ711857.1|InfluenzaAvirus_A/Greathornedowl/UT/22-035956-007-
original/2022_H5N1__segment4hemagglutinin_HA_genecompletecds|2022-10-
25|4|H5N1|USA|Bubovirginianus|USA_UT|Alphainfluenzavirus|Alphainfluenzaviru
sinfluenz"/>
NNN
</sequence>
<sequence>
<taxon idref="PQ711873.1|InfluenzaAvirus_A/Greathornedowl/WA/22-015526-004-
original/2022_H5N1__segment4hemagglutinin_HA_genecompletecds|2022-05-
09|4|H5N1|USA|Bubovirginianus|USA_WA|Alphainfluenzavirus|Alphainfluenzaviru
sinfluenz"/>
NNN
</sequence>
<sequence>
<taxon idref="PQ711889.1|InfluenzaAvirus_A/Greathornedowl/WA/22-038418-001-
original/2022_H5N1__segment4hemagglutinin_HA_genecompletecds|2022-11-
15|4|H5N1|USA|Bubovirginianus|USA_WA|Alphainfluenzavirus|Alphainfluenzaviru
sinfluenz"/>
NNN
</sequence>
<sequence>
<taxon idref="PQ712073.1|InfluenzaAvirus_A/Greathornedowl/WY/22-029194-001-
original/2022_H5N1__segment4hemagglutinin_HA_genecompletecds|2022-09-
11|4|H5N1|USA|Bubovirginianus|USA_WY|Alphainfluenzavirus|Alphainfluenzaviru
sinfluenz"/>
NNN
</sequence>
<sequence>
<taxon idref="PQ712081.1|InfluenzaAvirus_A/Greathornedowl/WY/22-030747-001-
original/2022_H5N1__segment4hemagglutinin_HA_genecompletecds|2022-09-
16|4|H5N1|USA|Bubovirginianus|USA_WY|Alphainfluenzavirus|Alphainfluenzaviru
sinfluenz"/>
NNN
</sequence>
<sequence>
<taxon idref="PQ712097.1|InfluenzaAvirus_A/Greathornedowl/WY/22-031607-001-
original/2022_H5N1__segment4hemagglutinin_HA_genecompletecds|2022-09-
29|4|H5N1|USA|Bubovirginianus|USA_WY|Alphainfluenzavirus|Alphainfluenzaviru
sinfluenz"/>
NNN
</sequence>
<sequence>
<taxon idref="PQ712105.1|InfluenzaAvirus_A/Greathornedowl/WY/22-032604-001-
original/2022_H5N1__segment4hemagglutinin_HA_genecompletecds|2022-10-
11|4|H5N1|USA|Bubovirginianus|USA_WY|Alphainfluenzavirus|Alphainfluenzaviru
sinfluenz"/>
NNN
</sequence>
<sequence>
<taxon idref="PQ712113.1|InfluenzaAvirus_A/Greathornedowl/WY/22-033035-001-
original/2022_H5N1__segment4hemagglutinin_HA_genecompletecds|2022-10-
12|4|H5N1|USA|Bubovirginianus|USA_WY|Alphainfluenzavirus|Alphainfluenzaviru
sinfluenz"/>
NNN
</sequence>
<sequence>

<taxon idref="PQ712121.1|InfluenzaAvirus_A/Greathornedowl/WY/22-033036-002-original/2022_H5N1__segment4hemagglutinin_HA_genecompletecds|2022-10-12|4|H5N1|USA|Bubovirginianus|USA_WY|Alphainfluenzavirus|Alphainfluenzavirusinfluenz"/>
NNN
</sequence>
<sequence>
<taxon idref="PQ712249.1|InfluenzaAvirus_A/Gull/AK/22-017953-006-original/2022_H5N1__segment4hemagglutinin_HA_genecompletecds|2022-06-06|4|H5N1|USA|Laridae|USA_AK|Alphainfluenzavirus|Alphainfluenzavirusinfluenz"/>
NNN
</sequence>
<sequence>
<taxon idref="PQ712257.1|InfluenzaAvirus_A/Gull/AK/22-017953-007-original/2022_H5N1__segment4hemagglutinin_HA_genecompletecds|2022-06-06|4|H5N1|USA|Laridae|USA_AK|Alphainfluenzavirus|Alphainfluenzavirusinfluenz"/>
NNN
</sequence>
<sequence>
<taxon idref="PQ712361.1|InfluenzaAvirus_A/Gull/UT/22-031729-002-original-repeat/2022_H5N1__segment4hemagglutinin_HA_genecompletecds|2022-09-29|4|H5N1|USA|Laridae|USA_UT|Alphainfluenzavirus|Alphainfluenzavirusinfluenz"/>
NNN
</sequence>
<sequence>
<taxon idref="PQ712369.1|InfluenzaAvirus_A/Gull/UT/22-031729-007-original/2022_H5N1__segment4hemagglutinin_HA_genecompletecds|2022-09-29|4|H5N1|USA|Laridae|USA_UT|Alphainfluenzavirus|Alphainfluenzavirusinfluenz"/>
NNN
</sequence>
<sequence>
<taxon idref="PQ712393.1|InfluenzaAvirus_A/Hawk/ID/22-017864-001-original/2022_H5N1__segment4hemagglutinin_HA_genecompletecds|2022-06-06|4|H5N1|USA|Accipitridae|USA_ID|Alphainfluenzavirus|Alphainfluenzavirusinfluenz"/>
NNN
</sequence>
<sequence>
<taxon idref="PQ712537.1|InfluenzaAvirus_A/Hawk/WY/23-000336-001-original/2022_H5N1__segment4hemagglutinin_HA_genecompletecds|2022-10-30|4|H5N1|USA|Accipitridae|USA_WY|Alphainfluenzavirus|Alphainfluenzavirusinfluenz"/>
NNN
</sequence>
<sequence>
<taxon idref="PQ712929.1|InfluenzaAvirus_A/Mallard/AK/22-029101-001-original/2022_H5N1__segment4hemagglutinin_HA_genecompletecds|2022-09-05|4|H5N1|USA|Anasplatyrhynchos|USA_AK|Alphainfluenzavirus|Alphainfluenzavirusinfluenz"/>
NNN
</sequence>
<sequence>
<taxon idref="PQ712937.1|InfluenzaAvirus_A/Mallard/AK/22-031400-001-original-repeat/2022_H5N1__segment4hemagglutinin_HA_genecompletecds|2022-09-03|4|H5N1|USA|Anasplatyrhynchos|USA_AK|Alphainfluenzavirus|Alphainfluenzavirusinfluenz"/>

NNN
</sequence>
<sequence>
<taxon idref="PQ712945.1|InfluenzaAvirus_A/Mallard/AK/22-036139-015-original/2022_H5N1__segment4hemagglutinin_HA_genecompletecds|2022-11-02|4|H5N1|USA|Anasplatyrhynchos|USA_AK|Alphainfluenzavirus|Alphainfluenzavirusinfluenz"/>
NNN
</sequence>
<sequence>
<taxon idref="PQ712953.1|InfluenzaAvirus_A/Mallard/AK/22-036139-029-original/2022_H5N1__segment4hemagglutinin_HA_genecompletecds|2022-11-02|4|H5N1|USA|Anasplatyrhynchos|USA_AK|Alphainfluenzavirus|Alphainfluenzavirusinfluenz"/>
NNN
</sequence>
<sequence>
<taxon idref="PQ712969.1|InfluenzaAvirus_A/Mallard/AK/22-036139-032-original/2022_H5N1__segment4hemagglutinin_HA_genecompletecds|2022-11-02|4|H5N1|USA|Anasplatyrhynchos|USA_AK|Alphainfluenzavirus|Alphainfluenzavirusinfluenz"/>
NNN
</sequence>
<sequence>
<taxon idref="PQ712977.1|InfluenzaAvirus_A/Mallard/AK/22-036139-037-original/2022_H5N1__segment4hemagglutinin_HA_genecompletecds|2022-11-02|4|H5N1|USA|Anasplatyrhynchos|USA_AK|Alphainfluenzavirus|Alphainfluenzavirusinfluenz"/>
NNN
</sequence>
<sequence>
<taxon idref="PQ712985.1|InfluenzaAvirus_A/Mallard/AK/22-036139-058-original/2022_H5N1__segment4hemagglutinin_HA_genecompletecds|2022-11-02|4|H5N1|USA|Anasplatyrhynchos|USA_AK|Alphainfluenzavirus|Alphainfluenzavirusinfluenz"/>
NNN
</sequence>
<sequence>
<taxon idref="PQ713001.1|InfluenzaAvirus_A/Mallard/AK/22-036139-062-original/2022_H5N1__segment4hemagglutinin_HA_genecompletecds|2022-11-02|4|H5N1|USA|Anasplatyrhynchos|USA_AK|Alphainfluenzavirus|Alphainfluenzavirusinfluenz"/>
NNN
</sequence>
<sequence>
<taxon idref="PQ713009.1|InfluenzaAvirus_A/Mallard/AK/22-036139-063-original/2022_H5N1__segment4hemagglutinin_HA_genecompletecds|2022-11-02|4|H5N1|USA|Anasplatyrhynchos|USA_AK|Alphainfluenzavirus|Alphainfluenzavirusinfluenz"/>
NNN
</sequence>
<sequence>
<taxon idref="PQ713017.1|InfluenzaAvirus_A/Mallard/AK/22-036139-081-original/2022_H5N1__segment4hemagglutinin_HA_genecompletecds|2022-11-02|4|H5N1|USA|Anasplatyrhynchos|USA_AK|Alphainfluenzavirus|Alphainfluenzavirusinfluenz"/>
NNN
</sequence>
<sequence>
<taxon idref="PQ713025.1|InfluenzaAvirus_A/Mallard/AK/22-036139-092-original/2022_H5N1__segment4hemagglutinin_HA_genecompletecds|2022-11-

02|4|H5N1|USA|Anasplatyrhynchos|USA_AK|Alphainfluenzavirus|Alphainfluenzavirusinfluenz"/>
NNN
</sequence>
<sequence>
<taxon idref="PQ713033.1|InfluenzaAvirus_A/Mallard/AK/23-002693-020-original/2022_H5N1__segment4hemagglutinin_HA_genecompletecds|2022-09-30|4|H5N1|USA|Anasplatyrhynchos|USA_AK|Alphainfluenzavirus|Alphainfluenzavirusinfluenz"/>
NNN
</sequence>
<sequence>
<taxon idref="PQ713041.1|InfluenzaAvirus_A/Mallard/AK/23-002693-024-original/2022_H5N1__segment4hemagglutinin_HA_genecompletecds|2022-09-30|4|H5N1|USA|Anasplatyrhynchos|USA_AK|Alphainfluenzavirus|Alphainfluenzavirusinfluenz"/>
NNN
</sequence>
<sequence>
<taxon idref="PQ713049.1|InfluenzaAvirus_A/Mallard/AK/23-003143-001-original/2022_H5N1__segment4hemagglutinin_HA_genecompletecds|2022-09-30|4|H5N1|USA|Anasplatyrhynchos|USA_AK|Alphainfluenzavirus|Alphainfluenzavirusinfluenz"/>
NNN
</sequence>
<sequence>
<taxon idref="PQ713057.1|InfluenzaAvirus_A/Mallard/AK/23-003641-001-original/2022_H5N1__segment4hemagglutinin_HA_genecompletecds|2022-10-09|4|H5N1|USA|Anasplatyrhynchos|USA_AK|Alphainfluenzavirus|Alphainfluenzavirusinfluenz"/>
NNN
</sequence>
<sequence>
<taxon idref="PQ713065.1|InfluenzaAvirus_A/Mallard/AK/23-003641-008-original/2022_H5N1__segment4hemagglutinin_HA_genecompletecds|2022-10-14|4|H5N1|USA|Anasplatyrhynchos|USA_AK|Alphainfluenzavirus|Alphainfluenzavirusinfluenz"/>
NNN
</sequence>
<sequence>
<taxon idref="PQ713129.1|InfluenzaAvirus_A/Mallard/CO/22-033430-002-original/2022_H5N1__segment4hemagglutinin_HA_genecompletecds|2022-10-09|4|H5N1|USA|Anasplatyrhynchos|USA_CO|Alphainfluenzavirus|Alphainfluenzavirusinfluenz"/>
NNN
</sequence>
<sequence>
<taxon idref="PQ713273.1|InfluenzaAvirus_A/Mallard/IN/22-034829-007-original-repeat2/2022_H5N1__segment4hemagglutinin_HA_genecompletecds|2022-10-28|4|H5N1|USA|Anasplatyrhynchos|USA_IN|Alphainfluenzavirus|Alphainfluenzavirusinfluenz"/>
NNN
</sequence>
<sequence>
<taxon idref="PQ713505.1|InfluenzaAvirus_A/Mallard/MN/22-033478-004-original/2022_H5N1__segment4hemagglutinin_HA_genecompletecds|2022-09-13|4|H5N1|USA|Anasplatyrhynchos|USA_MN|Alphainfluenzavirus|Alphainfluenzavirusinfluenz"/>
NNN
</sequence>

<sequence>
<taxon idref="PQ713929.1|InfluenzaAvirus_A/Mallard/OR/22-036880-048-original/2022_H5N1__segment4hemagglutinin_HA_genecompletecds|2022-11-07|4|H5N1|USA|Anasplatyrhynchos|USA_OR|Alphainfluenzavirus|Alphainfluenzavirusinfluenz"/>
NNN
</sequence>
<sequence>
<taxon idref="PQ714161.1|InfluenzaAvirus_A/Mallard/WA/22-039095-002-original/2022_H5N1__segment4hemagglutinin_HA_genecompletecds|2022-11-25|4|H5N1|USA|Anasplatyrhynchos|USA_WA|Alphainfluenzavirus|Alphainfluenzavirusinfluenz"/>
NNN
</sequence>
<sequence>
<taxon idref="PQ714241.1|InfluenzaAvirus_A/MuscovyDuck/CA/22-034331-001-original-repeat/2022_H5N1__segment4hemagglutinin_HA_genecompletecds|2022-10-20|4|H5N1|USA|Cairinamoschata|USA_CA|Alphainfluenzavirus|Alphainfluenzavirusinfluenz"/>
NNN
</sequence>
<sequence>
<taxon idref="PQ714249.1|InfluenzaAvirus_A/MuscovyDuck/CA/22-034331-003-original-repeat/2022_H5N1__segment4hemagglutinin_HA_genecompletecds|2022-10-20|4|H5N1|USA|Cairinamoschata|USA_CA|Alphainfluenzavirus|Alphainfluenzavirusinfluenz"/>
NNN
</sequence>
<sequence>
<taxon idref="PQ714257.1|InfluenzaAvirus_A/MuscovyDuck/CA/22-034331-004-original-repeat/2022_H5N1__segment4hemagglutinin_HA_genecompletecds|2022-10-20|4|H5N1|USA|Cairinamoschata|USA_CA|Alphainfluenzavirus|Alphainfluenzavirusinfluenz"/>
NNN
</sequence>
<sequence>
<taxon idref="PQ714313.1|InfluenzaAvirus_A/MuscovyDuck/FL/22-036966-002-original/2022_H5N1__segment4hemagglutinin_HA_genecompletecds|2022-11-04|4|H5N1|USA|Cairinamoschata|USA_FL|Alphainfluenzavirus|Alphainfluenzavirusinfluenz"/>
NNN
</sequence>
<sequence>
<taxon idref="PQ714457.1|InfluenzaAvirus_A/Northernpintail/AK/22-029100-001-original/2022_H5N1__segment4hemagglutinin_HA_genecompletecds|2022-09-04|4|H5N1|USA|Anasacuta|USA_AK|Alphainfluenzavirus|Alphainfluenzavirusinfluenz"/>
NNN
</sequence>
<sequence>
<taxon idref="PQ714481.1|InfluenzaAvirus_A/Northernpintail/AK/22-039438-001-original/2022_H5N1__segment4hemagglutinin_HA_genecompletecds|2022-09-02|4|H5N1|USA|Anasacuta|USA_AK|Alphainfluenzavirus|Alphainfluenzavirusinfluenz"/>
NNN
</sequence>
<sequence>

<taxon idref="PQ714489.1|InfluenzaAvirus_A/Northernpintail/AK/22-039438-005-original/2022_H5N1__segment4hemagglutinin_HA_genecompletecds|2022-09-07|4|H5N1|USA|Anasacuta|USA_AK|Alphainfluenzavirus|Alphainfluenzavirusinfluenz"/>
NNN
</sequence>
<sequence>
<taxon idref="PQ714497.1|InfluenzaAvirus_A/Northernpintail/AK/22-039438-008-original/2022_H5N1__segment4hemagglutinin_HA_genecompletecds|2022-09-09|4|H5N1|USA|Anasacuta|USA_AK|Alphainfluenzavirus|Alphainfluenzavirusinfluenz"/>
NNN
</sequence>
<sequence>
<taxon idref="PQ714505.1|InfluenzaAvirus_A/Northernpintail/AK/22-039438-009-original/2022_H5N1__segment4hemagglutinin_HA_genecompletecds|2022-09-09|4|H5N1|USA|Anasacuta|USA_AK|Alphainfluenzavirus|Alphainfluenzavirusinfluenz"/>
NNN
</sequence>
<sequence>
<taxon idref="PQ714513.1|InfluenzaAvirus_A/Northernpintail/AK/22-039438-010-original/2022_H5N1__segment4hemagglutinin_HA_genecompletecds|2022-09-09|4|H5N1|USA|Anasacuta|USA_AK|Alphainfluenzavirus|Alphainfluenzavirusinfluenz"/>
NNN
</sequence>
<sequence>
<taxon idref="PQ714521.1|InfluenzaAvirus_A/Northernpintail/AK/22-039438-011-original/2022_H5N1__segment4hemagglutinin_HA_genecompletecds|2022-09-09|4|H5N1|USA|Anasacuta|USA_AK|Alphainfluenzavirus|Alphainfluenzavirusinfluenz"/>
NNN
</sequence>
<sequence>
<taxon idref="PQ714529.1|InfluenzaAvirus_A/Northernpintail/AK/22-039438-013-original/2022_H5N1__segment4hemagglutinin_HA_genecompletecds|2022-09-12|4|H5N1|USA|Anasacuta|USA_AK|Alphainfluenzavirus|Alphainfluenzavirusinfluenz"/>
NNN
</sequence>
<sequence>
<taxon idref="PQ714537.1|InfluenzaAvirus_A/Northernpintail/AK/22-039438-014-original/2022_H5N1__segment4hemagglutinin_HA_genecompletecds|2022-09-12|4|H5N1|USA|Anasacuta|USA_AK|Alphainfluenzavirus|Alphainfluenzavirusinfluenz"/>
NNN
</sequence>
<sequence>
<taxon idref="PQ714545.1|InfluenzaAvirus_A/Northernpintail/AK/22-039438-017-original/2022_H5N1__segment4hemagglutinin_HA_genecompletecds|2022-09-14|4|H5N1|USA|Anasacuta|USA_AK|Alphainfluenzavirus|Alphainfluenzavirusinfluenz"/>
NNN
</sequence>
<sequence>
<taxon idref="PQ714553.1|InfluenzaAvirus_A/Northernpintail/AK/22-039438-018-original/2022_H5N1__segment4hemagglutinin_HA_genecompletecds|2022-09-14|4|H5N1|USA|Anasacuta|USA_AK|Alphainfluenzavirus|Alphainfluenzavirusinfluenz"/>
NNN

</sequence>
<sequence>
<taxon idref="PQ714577.1|InfluenzaAvirus_A/Northernpintail/AK/23-002693-006-original/2022_H5N1__segment4hemagglutinin_HA_genecompletecds|2022-09-28|4|H5N1|USA|Anasacuta|USA_AK|Alphainfluenzavirus|Alphainfluenzavirusinfluenz"/>
NNN
</sequence>
<sequence>
<taxon idref="PQ714585.1|InfluenzaAvirus_A/Northernpintail/AK/23-002693-007-original/2022_H5N1__segment4hemagglutinin_HA_genecompletecds|2022-09-28|4|H5N1|USA|Anasacuta|USA_AK|Alphainfluenzavirus|Alphainfluenzavirusinfluenz"/>
NNN
</sequence>
<sequence>
<taxon idref="PQ714601.1|InfluenzaAvirus_A/Northernpintail/AK/23-002693-012-original/2022_H5N1__segment4hemagglutinin_HA_genecompletecds|2022-09-28|4|H5N1|USA|Anasacuta|USA_AK|Alphainfluenzavirus|Alphainfluenzavirusinfluenz"/>
NNN
</sequence>
<sequence>
<taxon idref="PQ714617.1|InfluenzaAvirus_A/Northernpintail/AK/23-002693-014-original/2022_H5N1__segment4hemagglutinin_HA_genecompletecds|2022-09-30|4|H5N1|USA|Anasacuta|USA_AK|Alphainfluenzavirus|Alphainfluenzavirusinfluenz"/>
NNN
</sequence>
<sequence>
<taxon idref="PQ714625.1|InfluenzaAvirus_A/Northernpintail/AK/23-002693-015-original/2022_H5N1__segment4hemagglutinin_HA_genecompletecds|2022-09-30|4|H5N1|USA|Anasacuta|USA_AK|Alphainfluenzavirus|Alphainfluenzavirusinfluenz"/>
NNN
</sequence>
<sequence>
<taxon idref="PQ714633.1|InfluenzaAvirus_A/Northernpintail/AK/23-002693-016-original/2022_H5N1__segment4hemagglutinin_HA_genecompletecds|2022-09-30|4|H5N1|USA|Anasacuta|USA_AK|Alphainfluenzavirus|Alphainfluenzavirusinfluenz"/>
NNN
</sequence>
<sequence>
<taxon idref="PQ714641.1|InfluenzaAvirus_A/Northernpintail/AK/23-002693-017-original/2022_H5N1__segment4hemagglutinin_HA_genecompletecds|2022-09-30|4|H5N1|USA|Anasacuta|USA_AK|Alphainfluenzavirus|Alphainfluenzavirusinfluenz"/>
NNN
</sequence>
<sequence>
<taxon idref="PQ714649.1|InfluenzaAvirus_A/Northernpintail/AK/23-002693-018-original/2022_H5N1__segment4hemagglutinin_HA_genecompletecds|2022-09-30|4|H5N1|USA|Anasacuta|USA_AK|Alphainfluenzavirus|Alphainfluenzavirusinfluenz"/>
NNN
</sequence>
<sequence>
<taxon idref="PQ714657.1|InfluenzaAvirus_A/Northernpintail/AK/23-002693-021-original/2022_H5N1__segment4hemagglutinin_HA_genecompletecds|2022-09-

30|4|H5N1|USA|Anasacuta|USA_AK|Alphainfluenzavirus|Alphainfluenzavirusinflu
enz"/>
NNN
</sequence>
<sequence>
<taxon idref="PQ714697.1|InfluenzaAvirus_A/Northernpintail/AK/23-002693-
030-original/2022_H5N1__segment4hemagglutinin_HA_genecompletecds|2022-10-
02|4|H5N1|USA|Anasacuta|USA_AK|Alphainfluenzavirus|Alphainfluenzavirusinflu
enz"/>
NNN
</sequence>
<sequence>
<taxon idref="PQ714705.1|InfluenzaAvirus_A/Northernpintail/LA/22-039392-
003-original-
repeat/2022_H5N1__segment4hemagglutinin_HA_genecompletecds|2022-11-
28|4|H5N1|USA|Anasacuta|USA_LA|Alphainfluenzavirus|Alphainfluenzavirusinflu
enz"/>
NNN
</sequence>
<sequence>
<taxon idref="PQ714713.1|InfluenzaAvirus_A/Northernpintail/OR/22-034677-
048-original-
repeat2/2022_H5N1__segment4hemagglutinin_HA_genecompletecds|2022-10-
08|4|H5N1|USA|Anasacuta|USA_OR|Alphainfluenzavirus|Alphainfluenzavirusinflu
enz"/>
NNN
</sequence>
<sequence>
<taxon idref="PQ714737.1|InfluenzaAvirus_A/Northernshoveler/CA/23-005876-
020-original/2022_H5N1__segment4hemagglutinin_HA_genecompletecds|2022-10-
23|4|H5N1|USA|Spatulaclypeata|USA_CA|Alphainfluenzavirus|Alphainfluenzaviru
sinfluenz"/>
NNN
</sequence>
<sequence>
<taxon idref="PQ714745.1|InfluenzaAvirus_A/Northernshoveler/CA/23-005876-
026-original/2022_H5N1__segment4hemagglutinin_HA_genecompletecds|2022-10-
23|4|H5N1|USA|Spatulaclypeata|USA_CA|Alphainfluenzavirus|Alphainfluenzaviru
sinfluenz"/>
NNN
</sequence>
<sequence>
<taxon idref="PQ714761.1|InfluenzaAvirus_A/Northernshoveler/LA/22-037236-
004-original/2022_H5N1__segment4hemagglutinin_HA_genecompletecds|2022-11-
12|4|H5N1|USA|Spatulaclypeata|USA_LA|Alphainfluenzavirus|Alphainfluenzaviru
sinfluenz"/>
NNN
</sequence>
<sequence>
<taxon idref="PQ714777.1|InfluenzaAvirus_A/Northernshoveler/NV/22-037517-
003-original/2022_H5N1__segment4hemagglutinin_HA_genecompletecds|2022-11-
05|4|H5N1|USA|Spatulaclypeata|USA_NV|Alphainfluenzavirus|Alphainfluenzaviru
sinfluenz"/>
NNN
</sequence>
<sequence>
<taxon idref="PQ714785.1|InfluenzaAvirus_A/Northernshoveler/OR/22-036880-
083-original/2022_H5N1__segment4hemagglutinin_HA_genecompletecds|2022-11-
07|4|H5N1|USA|Spatulaclypeata|USA_OR|Alphainfluenzavirus|Alphainfluenzaviru
sinfluenz"/>
NNN

</sequence>
<sequence>
<taxon idref="PQ714937.1|InfluenzaAvirus_A/Parasiticjaeger/AK/22-020952-001-original/2022_H5N1__segment4hemagglutinin_HA_genecompletecds|2022-07-22|4|H5N1|USA||USA_AK|Alphainfluenzavirus|Alphainfluenzavirusinfluenz"/>
NNN
</sequence>
<sequence>
<taxon idref="PQ714953.1|InfluenzaAvirus_A/Pelican/AZ/22-038234-001-original/2022_H5N1__segment4hemagglutinin_HA_genecompletecds|2022-10-15|4|H5N1|USA|Pelecanidae|USA_AZ|Alphainfluenzavirus|Alphainfluenzavirusinfluenz"/>
NNN
</sequence>
<sequence>
<taxon idref="PQ714961.1|InfluenzaAvirus_A/Pelican/AZ/22-038234-002-original/2022_H5N1__segment4hemagglutinin_HA_genecompletecds|2022-10-15|4|H5N1|USA|Pelecanidae|USA_AZ|Alphainfluenzavirus|Alphainfluenzavirusinfluenz"/>
NNN
</sequence>
<sequence>
<taxon idref="PQ714969.1|InfluenzaAvirus_A/Pelican/AZ/22-038234-003-original/2022_H5N1__segment4hemagglutinin_HA_genecompletecds|2022-10-15|4|H5N1|USA|Pelecanidae|USA_AZ|Alphainfluenzavirus|Alphainfluenzavirusinfluenz"/>
NNN
</sequence>
<sequence>
<taxon idref="PQ714977.1|InfluenzaAvirus_A/Pelican/AZ/22-038234-004-original/2022_H5N1__segment4hemagglutinin_HA_genecompletecds|2022-10-15|4|H5N1|USA|Pelecanidae|USA_AZ|Alphainfluenzavirus|Alphainfluenzavirusinfluenz"/>
NNN
</sequence>
<sequence>
<taxon idref="PQ714985.1|InfluenzaAvirus_A/Pelican/AZ/22-038234-005-original/2022_H5N1__segment4hemagglutinin_HA_genecompletecds|2022-10-15|4|H5N1|USA|Pelecanidae|USA_AZ|Alphainfluenzavirus|Alphainfluenzavirusinfluenz"/>
NNN
</sequence>
<sequence>
<taxon idref="PQ714993.1|InfluenzaAvirus_A/Pelican/CA/22-037519-001-original/2022_H5N1__segment4hemagglutinin_HA_genecompletecds|2022-11-04|4|H5N1|USA|Pelecanidae|USA_CA|Alphainfluenzavirus|Alphainfluenzavirusinfluenz"/>
NNN
</sequence>
<sequence>
<taxon idref="PQ715033.1|InfluenzaAvirus_A/Pelican/KS/22-036624-001-original-repeat/2022_H5N1__segment4hemagglutinin_HA_genecompletecds|2022-10-17|4|H5N1|USA|Pelecanidae|USA_KS|Alphainfluenzavirus|Alphainfluenzavirusinfluenz"/>
NNN
</sequence>
<sequence>
<taxon idref="PQ715065.1|InfluenzaAvirus_A/Pelican/NE/22-032908-001-original/2022_H5N1__segment4hemagglutinin_HA_genecompletecds|2022-10-

13|4|H5N1|USA|Pelecanidae|USA_NE|Alphainfluenzavirus|Alphainfluenzavirusinf
luenz"/>
NNN
</sequence>
<sequence>
<taxon idref="PQ715089.1|InfluenzaAvirus_A/Peregrinefalcon/CA/22-032573-
001-original/2022_H5N1__segment4hemagglutinin_HA_genecompletecds|2022-10-
03|4|H5N1|USA|Falcoperegrinus|USA_CA|Alphainfluenzavirus|Alphainfluenzaviru
sinfluenz"/>
NNN
</sequence>
<sequence>
<taxon idref="PQ715105.1|InfluenzaAvirus_A/Peregrinefalcon/CA/22-039721-
003-original-3/2022_H5N1__segment4hemagglutinin_HA_genecompletecds|2022-11-
16|4|H5N1|USA|Falcoperegrinus|USA_CA|Alphainfluenzavirus|Alphainfluenzaviru
sinfluenz"/>
NNN
</sequence>
<sequence>
<taxon idref="PQ715121.1|InfluenzaAvirus_A/Peregrinefalcon/FL/22-035514-
001-original/2022_H5N1__segment4hemagglutinin_HA_genecompletecds|2022-10-
29|4|H5N1|USA|Falcoperegrinus|USA_FL|Alphainfluenzavirus|Alphainfluenzaviru
sinfluenz"/>
NNN
</sequence>
<sequence>
<taxon idref="PQ715129.1|InfluenzaAvirus_A/Peregrinefalcon/FL/22-035514-
002-original/2022_H5N1__segment4hemagglutinin_HA_genecompletecds|2022-10-
29|4|H5N1|USA|Falcoperegrinus|USA_FL|Alphainfluenzavirus|Alphainfluenzaviru
sinfluenz"/>
NNN
</sequence>
<sequence>
<taxon idref="PQ715137.1|InfluenzaAvirus_A/Peregrinefalcon/IN/22-032710-
001-original/2022_H5N1__segment4hemagglutinin_HA_genecompletecds|2022-10-
07|4|H5N1|USA|Falcoperegrinus|USA_IN|Alphainfluenzavirus|Alphainfluenzaviru
sinfluenz"/>
NNN
</sequence>
<sequence>
<taxon idref="PQ715233.1|InfluenzaAvirus_A/Peregrinefalcon/MN/22-031157-
003-original-
repeat/2022_H5N1__segment4hemagglutinin_HA_genecompletecds|2022-09-
29|4|H5N1|USA|Falcoperegrinus|USA_MN|Alphainfluenzavirus|Alphainfluenzaviru
sinfluenz"/>
NNN
</sequence>
<sequence>
<taxon idref="PQ715241.1|InfluenzaAvirus_A/Peregrinefalcon/MN/22-032849-
001-original/2022_H5N1__segment4hemagglutinin_HA_genecompletecds|2022-10-
13|4|H5N1|USA|Falcoperegrinus|USA_MN|Alphainfluenzavirus|Alphainfluenzaviru
sinfluenz"/>
NNN
</sequence>
<sequence>
<taxon idref="PQ715249.1|InfluenzaAvirus_A/Peregrinefalcon/MN/22-032849-
002-original/2022_H5N1__segment4hemagglutinin_HA_genecompletecds|2022-10-
13|4|H5N1|USA|Falcoperegrinus|USA_MN|Alphainfluenzavirus|Alphainfluenzaviru
sinfluenz"/>
NNN
</sequence>

<sequence>
<taxon idref="PQ715385.1|InfluenzaAvirus_A/Raven/AK/22-020951-001-original/2022_H5N1__segment4hemagglutinin_HA_genecompletecds|2022-06-26|4|H5N1|USA|Corvus|USA_AK|Alphainfluenzavirus|Alphainfluenzavirusinfluenz"/>
NNN
</sequence>
<sequence>
<taxon idref="PQ715393.1|InfluenzaAvirus_A/Raven/AK/22-020951-002-original/2022_H5N1__segment4hemagglutinin_HA_genecompletecds|2022-06-26|4|H5N1|USA|Corvus|USA_AK|Alphainfluenzavirus|Alphainfluenzavirusinfluenz"/>
NNN
</sequence>
<sequence>
<taxon idref="PQ715417.1|InfluenzaAvirus_A/Raven/AK/22-031265-001-original/2022_H5N1__segment4hemagglutinin_HA_genecompletecds|2022-09-20|4|H5N1|USA|Corvus|USA_AK|Alphainfluenzavirus|Alphainfluenzavirusinfluenz"/>
NNN
</sequence>
<sequence>
<taxon idref="PQ715537.1|InfluenzaAvirus_A/RedFox/AK/22-020792-002-original/2022_H5N1__segment4hemagglutinin_HA_genecompletecds|2022-06-13|4|H5N1|USA|Vulpesvulpes|USA_AK|Alphainfluenzavirus|Alphainfluenzavirusinfluenz"/>
NNN
</sequence>
<sequence>
<taxon idref="PQ715649.1|InfluenzaAvirus_A/Red-tailedhawk/CA/22-033187-001-original/2022_H5N1__segment4hemagglutinin_HA_genecompletecds|2022-10-12|4|H5N1|USA|Buteojamaicensis|USA_CA|Alphainfluenzavirus|Alphainfluenzavirusinfluenz"/>
NNN
</sequence>
<sequence>
<taxon idref="PQ715737.1|InfluenzaAvirus_A/Red-tailedhawk/CO/22-029818-001-original-repeat/2022_H5N1__segment4hemagglutinin_HA_genecompletecds|2022-09-19|4|H5N1|USA|Buteojamaicensis|USA_CO|Alphainfluenzavirus|Alphainfluenzavirusinfluenz"/>
NNN
</sequence>
<sequence>
<taxon idref="PQ715745.1|InfluenzaAvirus_A/Red-tailedhawk/CO/22-031741-001-original/2022_H5N1__segment4hemagglutinin_HA_genecompletecds|2022-10-04|4|H5N1|USA|Buteojamaicensis|USA_CO|Alphainfluenzavirus|Alphainfluenzavirusinfluenz"/>
NNN
</sequence>
<sequence>
<taxon idref="PQ715753.1|InfluenzaAvirus_A/Red-tailedhawk/CO/22-037213-001-original/2022_H5N1__segment4hemagglutinin_HA_genecompletecds|2022-11-14|4|H5N1|USA|Buteojamaicensis|USA_CO|Alphainfluenzavirus|Alphainfluenzavirusinfluenz"/>
NNN
</sequence>
<sequence>
<taxon idref="PQ715761.1|InfluenzaAvirus_A/Red-tailedhawk/CO/22-037824-001-original/2022_H5N1__segment4hemagglutinin_HA_genecompletecds|2022-11-

18|4|H5N1|USA|Buteojamaicensis|USA_CO|Alphainfluenzavirus|Alphainfluenzavir
usinfluenz"/>
NNN
</sequence>
<sequence>
<taxon idref="PQ716385.1|InfluenzaAvirus_A/Red-tailedhawk/MT/22-036316-001-
original/2022_H5N1__segment4hemagglutinin_HA_genecompletecds|2022-11-
09|4|H5N1|USA|Buteojamaicensis|USA_MT|Alphainfluenzavirus|Alphainfluenzavir
usinfluenz"/>
NNN
</sequence>
<sequence>
<taxon idref="PQ716737.1|InfluenzaAvirus_A/Red-tailedhawk/WA/22-037658-001-
original/2022_H5N1__segment4hemagglutinin_HA_genecompletecds|2022-11-
16|4|H5N1|USA|Buteojamaicensis|USA_WA|Alphainfluenzavirus|Alphainfluenzavir
usinfluenz"/>
NNN
</sequence>
<sequence>
<taxon idref="PQ716913.1|InfluenzaAvirus_A/Ring-billedgull/CO/22-030941-
001-original/2022_H5N1__segment4hemagglutinin_HA_genecompletecds|2022-09-
28|4|H5N1|USA|Larusdelawarensis|USA_CO|Alphainfluenzavirus|Alphainfluenzavi
rusinfluenz"/>
NNN
</sequence>
<sequence>
<taxon idref="PQ717001.1|InfluenzaAvirus_A/Roseatespoonbill/TX/22-035763-
001-original/2022_H5N1__segment4hemagglutinin_HA_genecompletecds|2022-10-
11|4|H5N1|USA||USA_TX|Alphainfluenzavirus|Alphainfluenzavirusinfluenz"/>
NNN
</sequence>
<sequence>
<taxon idref="PQ717049.1|InfluenzaAvirus_A/Ross_sgoose/KS/22-038592-005-
original/2022_H5N1__segment4hemagglutinin_HA_genecompletecds|2022-11-
21|4|H5N1|USA||USA_KS|Alphainfluenzavirus|Alphainfluenzavirusinfluenz"/>
NNN
</sequence>
<sequence>
<taxon idref="PQ717073.1|InfluenzaAvirus_A/Ross_sgoose/KS/22-041130-002-
original/2022_H5N1__segment4hemagglutinin_HA_genecompletecds|2022-11-
29|4|H5N1|USA||USA_KS|Alphainfluenzavirus|Alphainfluenzavirusinfluenz"/>
NNN
</sequence>
<sequence>
<taxon idref="PQ717225.1|InfluenzaAvirus_A/Sandhillcrane/AK/22-019268-001-
original/2022_H5N1__segment4hemagglutinin_HA_genecompletecds|2022-06-
14|4|H5N1|USA|Antigonecanadensis|USA_AK|Alphainfluenzavirus|Alphainfluenzav
irusinfluenz"/>
NNN
</sequence>
<sequence>
<taxon idref="PQ717329.1|InfluenzaAvirus_A/Short-earedOwl/WA/22-039678-004-
original/2022_H5N1__segment4hemagglutinin_HA_genecompletecds|2022-11-
21|4|H5N1|USA|Asioflammeus|USA_WA|Alphainfluenzavirus|Alphainfluenzavirusin
fluenz"/>
NNN
</sequence>
<sequence>
<taxon idref="PQ717337.1|InfluenzaAvirus_A/Snowgoose/AK/22-017364-001-
original/2022_H5N1__segment4hemagglutinin_HA_genecompletecds|2022-05-

27|4|H5N1|USA|Ansercaerulescens|USA_AK|Alphainfluenzavirus|Alphainfluenzavi
rusinfluenz"/>
NNN
</sequence>
<sequence>
<taxon idref="PQ717353.1|InfluenzaAvirus_A/Snowgoose/AR/22-038202-001-
original/2022_H5N1__segment4hemagglutinin_HA_genecompletecds|2022-11-
25|4|H5N1|USA|Ansercaerulescens|USA_AR|Alphainfluenzavirus|Alphainfluenzavi
rusinfluenz"/>
NNN
</sequence>
<sequence>
<taxon idref="PQ717361.1|InfluenzaAvirus_A/Snowgoose/AR/22-039436-005-
original/2022_H5N1__segment4hemagglutinin_HA_genecompletecds|2022-11-
29|4|H5N1|USA|Ansercaerulescens|USA_AR|Alphainfluenzavirus|Alphainfluenzavi
rusinfluenz"/>
NNN
</sequence>
<sequence>
<taxon idref="PQ717441.1|InfluenzaAvirus_A/Snowgoose/CA/22-041406-001-
original/2022_H5N1__segment4hemagglutinin_HA_genecompletecds|2022-11-
28|4|H5N1|USA|Ansercaerulescens|USA_CA|Alphainfluenzavirus|Alphainfluenzavi
rusinfluenz"/>
NNN
</sequence>
<sequence>
<taxon idref="PQ717513.1|InfluenzaAvirus_A/Snowgoose/CA/23-001856-001-
original/2022_H5N1__segment4hemagglutinin_HA_genecompletecds|2022-11-
28|4|H5N1|USA|Ansercaerulescens|USA_CA|Alphainfluenzavirus|Alphainfluenzavi
rusinfluenz"/>
NNN
</sequence>
<sequence>
<taxon idref="PQ717537.1|InfluenzaAvirus_A/Snowgoose/CO/22-036625-001-
original-repeat/2022_H5N1__segment4hemagglutinin_HA_genecompletecds|2022-
11-
08|4|H5N1|USA|Ansercaerulescens|USA_CO|Alphainfluenzavirus|Alphainfluenzavi
rusinfluenz"/>
NNN
</sequence>
<sequence>
<taxon idref="PQ717545.1|InfluenzaAvirus_A/Snowgoose/CO/22-037670-001-
original/2022_H5N1__segment4hemagglutinin_HA_genecompletecds|2022-11-
16|4|H5N1|USA|Ansercaerulescens|USA_CO|Alphainfluenzavirus|Alphainfluenzavi
rusinfluenz"/>
NNN
</sequence>
<sequence>
<taxon idref="PQ717569.1|InfluenzaAvirus_A/Snowgoose/ID/22-038611-001-
original/2022_H5N1__segment4hemagglutinin_HA_genecompletecds|2022-11-
18|4|H5N1|USA|Ansercaerulescens|USA_ID|Alphainfluenzavirus|Alphainfluenzavi
rusinfluenz"/>
NNN
</sequence>
<sequence>
<taxon idref="PQ717577.1|InfluenzaAvirus_A/Snowgoose/ID/22-038611-002-
original/2022_H5N1__segment4hemagglutinin_HA_genecompletecds|2022-11-
18|4|H5N1|USA|Ansercaerulescens|USA_ID|Alphainfluenzavirus|Alphainfluenzavi
rusinfluenz"/>
NNN
</sequence>

<sequence>
<taxon idref="PQ717593.1|InfluenzaAvirus_A/Snowgoose/IN/22-038827-002-original-repeat/2022_H5N1__segment4hemagglutinin_HA_genecompletecds|2022-11-23|4|H5N1|USA|Ansercaerulescens|USA_IN|Alphainfluenzavirus|Alphainfluenzavirusinfluenz"/>
NNN
</sequence>
<sequence>
<taxon idref="PQ717601.1|InfluenzaAvirus_A/Snowgoose/IN/22-038827-003-original-repeat/2022_H5N1__segment4hemagglutinin_HA_genecompletecds|2022-11-23|4|H5N1|USA|Ansercaerulescens|USA_IN|Alphainfluenzavirus|Alphainfluenzavirusinfluenz"/>
NNN
</sequence>
<sequence>
<taxon idref="PQ717633.1|InfluenzaAvirus_A/Snowgoose/IN/22-038827-008-original-repeat/2022_H5N1__segment4hemagglutinin_HA_genecompletecds|2022-11-23|4|H5N1|USA|Ansercaerulescens|USA_IN|Alphainfluenzavirus|Alphainfluenzavirusinfluenz"/>
NNN
</sequence>
<sequence>
<taxon idref="PQ717657.1|InfluenzaAvirus_A/Snowgoose/KS/22-039690-001-original/2022_H5N1__segment4hemagglutinin_HA_genecompletecds|2022-11-21|4|H5N1|USA|Ansercaerulescens|USA_KS|Alphainfluenzavirus|Alphainfluenzavirusinfluenz"/>
NNN
</sequence>
<sequence>
<taxon idref="PQ717665.1|InfluenzaAvirus_A/Snowgoose/KS/22-039690-002-original/2022_H5N1__segment4hemagglutinin_HA_genecompletecds|2022-11-23|4|H5N1|USA|Ansercaerulescens|USA_KS|Alphainfluenzavirus|Alphainfluenzavirusinfluenz"/>
NNN
</sequence>
<sequence>
<taxon idref="PQ717689.1|InfluenzaAvirus_A/Snowgoose/KS/22-039690-005-original/2022_H5N1__segment4hemagglutinin_HA_genecompletecds|2022-11-23|4|H5N1|USA|Ansercaerulescens|USA_KS|Alphainfluenzavirus|Alphainfluenzavirusinfluenz"/>
NNN
</sequence>
<sequence>
<taxon idref="PQ717697.1|InfluenzaAvirus_A/Snowgoose/KS/22-039690-006-original/2022_H5N1__segment4hemagglutinin_HA_genecompletecds|2022-11-28|4|H5N1|USA|Ansercaerulescens|USA_KS|Alphainfluenzavirus|Alphainfluenzavirusinfluenz"/>
NNN
</sequence>
<sequence>
<taxon idref="PQ717705.1|InfluenzaAvirus_A/Snowgoose/KS/22-039690-007-original/2022_H5N1__segment4hemagglutinin_HA_genecompletecds|2022-11-27|4|H5N1|USA|Ansercaerulescens|USA_KS|Alphainfluenzavirus|Alphainfluenzavirusinfluenz"/>
NNN
</sequence>
<sequence>

<taxon idref="PQ717769.1|InfluenzaAvirus_A/Snowgoose/LA/22-039688-006-original/2022_H5N1__segment4hemagglutinin_HA_genecompletecds|2022-11-28|4|H5N1|USA|Ansercaerulescens|USA_LA|Alphainfluenzavirus|Alphainfluenzavirusinfluenz"/>
NNN
</sequence>
<sequence>
<taxon idref="PQ717777.1|InfluenzaAvirus_A/Snowgoose/LA/22-039688-009-original/2022_H5N1__segment4hemagglutinin_HA_genecompletecds|2022-11-30|4|H5N1|USA|Ansercaerulescens|USA_LA|Alphainfluenzavirus|Alphainfluenzavirusinfluenz"/>
NNN
</sequence>
<sequence>
<taxon idref="PQ717793.1|InfluenzaAvirus_A/Snowgoose/LA/23-000409-003-original/2022_H5N1__segment4hemagglutinin_HA_genecompletecds|2022-11-28|4|H5N1|USA|Ansercaerulescens|USA_LA|Alphainfluenzavirus|Alphainfluenzavirusinfluenz"/>
NNN
</sequence>
<sequence>
<taxon idref="PQ717841.1|InfluenzaAvirus_A/Snowgoose/MN/22-037439-001-original/2022_H5N1__segment4hemagglutinin_HA_genecompletecds|2022-11-17|4|H5N1|USA|Ansercaerulescens|USA_MN|Alphainfluenzavirus|Alphainfluenzavirusinfluenz"/>
NNN
</sequence>
<sequence>
<taxon idref="PQ717881.1|InfluenzaAvirus_A/Snowgoose/MO/22-037787-001-original/2022_H5N1__segment4hemagglutinin_HA_genecompletecds|2022-11-13|4|H5N1|USA|Ansercaerulescens|USA_MO|Alphainfluenzavirus|Alphainfluenzavirusinfluenz"/>
NNN
</sequence>
<sequence>
<taxon idref="PQ717897.1|InfluenzaAvirus_A/Snowgoose/MO/22-038564-002-original/2022_H5N1__segment4hemagglutinin_HA_genecompletecds|2022-11-23|4|H5N1|USA|Ansercaerulescens|USA_MO|Alphainfluenzavirus|Alphainfluenzavirusinfluenz"/>
NNN
</sequence>
<sequence>
<taxon idref="PQ717945.1|InfluenzaAvirus_A/Snowgoose/MS/22-038820-002-original-repeat/2022_H5N1__segment4hemagglutinin_HA_genecompletecds|2022-11-27|4|H5N1|USA|Ansercaerulescens|USA_MS|Alphainfluenzavirus|Alphainfluenzavirusinfluenz"/>
NNN
</sequence>
<sequence>
<taxon idref="PQ717969.1|InfluenzaAvirus_A/Snowgoose/MS/22-038820-006-original-repeat/2022_H5N1__segment4hemagglutinin_HA_genecompletecds|2022-11-27|4|H5N1|USA|Ansercaerulescens|USA_MS|Alphainfluenzavirus|Alphainfluenzavirusinfluenz"/>
NNN
</sequence>
<sequence>
<taxon idref="PQ717985.1|InfluenzaAvirus_A/Snowgoose/MS/22-038821-004-original/2022_H5N1__segment4hemagglutinin_HA_genecompletecds|2022-11-

23|4|H5N1|USA|Ansercaerulescens|USA_MS|Alphainfluenzavirus|Alphainfluenzavirusinfluenz"/>
NNN
</sequence>
<sequence>
<taxon idref="PQ718009.1|InfluenzaAvirus_A/Snowgoose/MS/22-038821-008-original/2022_H5N1__segment4hemagglutinin_HA_genecompletecds|2022-11-23|4|H5N1|USA|Ansercaerulescens|USA_MS|Alphainfluenzavirus|Alphainfluenzavirusinfluenz"/>
NNN
</sequence>
<sequence>
<taxon idref="PQ718017.1|InfluenzaAvirus_A/Snowgoose/MS/22-038821-010-original/2022_H5N1__segment4hemagglutinin_HA_genecompletecds|2022-11-23|4|H5N1|USA|Ansercaerulescens|USA_MS|Alphainfluenzavirus|Alphainfluenzavirusinfluenz"/>
NNN
</sequence>
<sequence>
<taxon idref="PQ718025.1|InfluenzaAvirus_A/Snowgoose/MS/22-038821-011-original/2022_H5N1__segment4hemagglutinin_HA_genecompletecds|2022-11-23|4|H5N1|USA|Ansercaerulescens|USA_MS|Alphainfluenzavirus|Alphainfluenzavirusinfluenz"/>
NNN
</sequence>
<sequence>
<taxon idref="PQ718033.1|InfluenzaAvirus_A/Snowgoose/MS/22-038821-012-original/2022_H5N1__segment4hemagglutinin_HA_genecompletecds|2022-11-23|4|H5N1|USA|Ansercaerulescens|USA_MS|Alphainfluenzavirus|Alphainfluenzavirusinfluenz"/>
NNN
</sequence>
<sequence>
<taxon idref="PQ718049.1|InfluenzaAvirus_A/Snowgoose/MS/22-038821-019-original/2022_H5N1__segment4hemagglutinin_HA_genecompletecds|2022-11-23|4|H5N1|USA|Ansercaerulescens|USA_MS|Alphainfluenzavirus|Alphainfluenzavirusinfluenz"/>
NNN
</sequence>
<sequence>
<taxon idref="PQ718073.1|InfluenzaAvirus_A/Snowgoose/MS/22-038821-023-original/2022_H5N1__segment4hemagglutinin_HA_genecompletecds|2022-11-23|4|H5N1|USA|Ansercaerulescens|USA_MS|Alphainfluenzavirus|Alphainfluenzavirusinfluenz"/>
NNN
</sequence>
<sequence>
<taxon idref="PQ718089.1|InfluenzaAvirus_A/Snowgoose/MS/22-038821-025-original/2022_H5N1__segment4hemagglutinin_HA_genecompletecds|2022-11-23|4|H5N1|USA|Ansercaerulescens|USA_MS|Alphainfluenzavirus|Alphainfluenzavirusinfluenz"/>
NNN
</sequence>
<sequence>
<taxon idref="PQ718097.1|InfluenzaAvirus_A/Snowgoose/MS/22-038821-026-original/2022_H5N1__segment4hemagglutinin_HA_genecompletecds|2022-11-23|4|H5N1|USA|Ansercaerulescens|USA_MS|Alphainfluenzavirus|Alphainfluenzavirusinfluenz"/>
NNN
</sequence>
<sequence>

<taxon idref="PQ718121.1|InfluenzaAvirus_A/Snowgoose/MS/22-038821-030-original/2022_H5N1__segment4hemagglutinin_HA_genecompletecds|2022-11-23|4|H5N1|USA|Ansercaerulescens|USA_MS|Alphainfluenzavirus|Alphainfluenzavirusinfluenz"/>
NNN
</sequence>
<sequence>
<taxon idref="PQ718153.1|InfluenzaAvirus_A/Snowgoose/MS/22-038821-039-original/2022_H5N1__segment4hemagglutinin_HA_genecompletecds|2022-11-23|4|H5N1|USA|Ansercaerulescens|USA_MS|Alphainfluenzavirus|Alphainfluenzavirusinfluenz"/>
NNN
</sequence>
<sequence>
<taxon idref="PQ718169.1|InfluenzaAvirus_A/Snowgoose/MS/22-038821-044-original/2022_H5N1__segment4hemagglutinin_HA_genecompletecds|2022-11-23|4|H5N1|USA|Ansercaerulescens|USA_MS|Alphainfluenzavirus|Alphainfluenzavirusinfluenz"/>
NNN
</sequence>
<sequence>
<taxon idref="PQ718281.1|InfluenzaAvirus_A/Snowgoose/ND/23-002238-001-original/2022_H5N1__segment4hemagglutinin_HA_genecompletecds|2022-11-29|4|H5N1|USA|Ansercaerulescens|USA_ND|Alphainfluenzavirus|Alphainfluenzavirusinfluenz"/>
NNN
</sequence>
<sequence>
<taxon idref="PQ718385.1|InfluenzaAvirus_A/Snowgoose/OR/22-038527-001-original/2022_H5N1__segment4hemagglutinin_HA_genecompletecds|2022-11-17|4|H5N1|USA|Ansercaerulescens|USA_OR|Alphainfluenzavirus|Alphainfluenzavirusinfluenz"/>
NNN
</sequence>
<sequence>
<taxon idref="PQ718393.1|InfluenzaAvirus_A/Snowgoose/OR/23-002231-001-original/2022_H5N1__segment4hemagglutinin_HA_genecompletecds|2022-11-19|4|H5N1|USA|Ansercaerulescens|USA_OR|Alphainfluenzavirus|Alphainfluenzavirusinfluenz"/>
NNN
</sequence>
<sequence>
<taxon idref="PQ718409.1|InfluenzaAvirus_A/Snowgoose/SD/22-038546-001-original/2022_H5N1__segment4hemagglutinin_HA_genecompletecds|2022-11-21|4|H5N1|USA|Ansercaerulescens|USA_SD|Alphainfluenzavirus|Alphainfluenzavirusinfluenz"/>
NNN
</sequence>
<sequence>
<taxon idref="PQ718417.1|InfluenzaAvirus_A/Snowgoose/SD/22-038788-002-original/2022_H5N1__segment4hemagglutinin_HA_genecompletecds|2022-11-21|4|H5N1|USA|Ansercaerulescens|USA_SD|Alphainfluenzavirus|Alphainfluenzavirusinfluenz"/>
NNN
</sequence>
<sequence>
<taxon idref="PQ718441.1|InfluenzaAvirus_A/Snowgoose/WA/22-037636-001-original/2022_H5N1__segment4hemagglutinin_HA_genecompletecds|2022-11-15|4|H5N1|USA|Ansercaerulescens|USA_WA|Alphainfluenzavirus|Alphainfluenzavirusinfluenz"/>
NNN

</sequence>
<sequence>
<taxon idref="PQ718449.1|InfluenzaAvirus_A/Snowgoose/WA/22-039678-001-original/2022_H5N1__segment4hemagglutinin_HA_genecompletecds|2022-11-21|4|H5N1|USA|Ansercaerulescens|USA_WA|Alphainfluenzavirus|Alphainfluenzavirusinfluenz"/>
NNN
</sequence>
<sequence>
<taxon idref="PQ718473.1|InfluenzaAvirus_A/Snowyowl/AK/22-022807-001-original/2022_H5N1__segment4hemagglutinin_HA_genecompletecds|2022-07-19|4|H5N1|USA|Buboscandiacus|USA_AK|Alphainfluenzavirus|Alphainfluenzavirusinfluenz"/>
NNN
</sequence>
<sequence>
<taxon idref="PQ718537.1|InfluenzaAvirus_A/Sparrow/NE/22-033885-001-original/2022_H5N1__segment4hemagglutinin_HA_genecompletecds|2022-10-19|4|H5N1|USA|Passeridae|USA_NE|Alphainfluenzavirus|Alphainfluenzavirusinfluenz"/>
NNN
</sequence>
<sequence>
<taxon idref="PQ718577.1|InfluenzaAvirus_A/Swainson_sHawk/OR/22-034146-001-original-repeat/2022_H5N1__segment4hemagglutinin_HA_genecompletecds|2022-10-10|4|H5N1|USA|Buteoswainsoni|USA_OR|Alphainfluenzavirus|Alphainfluenzavirusinfluenz"/>
NNN
</sequence>
<sequence>
<taxon idref="PQ718761.1|InfluenzaAvirus_A/TrumpeterSwan/MN/22-037438-001-original/2022_H5N1__segment4hemagglutinin_HA_genecompletecds|2022-11-17|4|H5N1|USA||USA_MN|Alphainfluenzavirus|Alphainfluenzavirusinfluenz"/>
NNN
</sequence>
<sequence>
<taxon idref="PQ718777.1|InfluenzaAvirus_A/TrumpeterSwan/MT/22-030263-001-original/2022_H5N1__segment4hemagglutinin_HA_genecompletecds|2022-09-16|4|H5N1|USA||USA_MT|Alphainfluenzavirus|Alphainfluenzavirusinfluenz"/>
NNN
</sequence>
<sequence>
<taxon idref="PQ718785.1|InfluenzaAvirus_A/TrumpeterSwan/WY/22-030049-002-original/2022_H5N1__segment4hemagglutinin_HA_genecompletecds|2022-09-14|4|H5N1|USA||USA_WY|Alphainfluenzavirus|Alphainfluenzavirusinfluenz"/>
NNN
</sequence>
<sequence>
<taxon idref="PQ718793.1|InfluenzaAvirus_A/TrumpeterSwan/WY/22-030049-003-original/2022_H5N1__segment4hemagglutinin_HA_genecompletecds|2022-09-14|4|H5N1|USA||USA_WY|Alphainfluenzavirus|Alphainfluenzavirusinfluenz"/>
NNN
</sequence>
<sequence>
<taxon idref="PQ718817.1|InfluenzaAvirus_A/TundraSwan/WI/22-038828-001-original-repeat/2022_H5N1__segment4hemagglutinin_HA_genecompletecds|2022-11-22|4|H5N1|USA|Cygnuscolumbianus|USA_WI|Alphainfluenzavirus|Alphainfluenzavirusinfluenz"/>
NNN

</sequence>
<sequence>
<taxon idref="PQ719113.1|InfluenzaAvirus_A/TurkeyVulture/MN/22-035505-002-original/2022_H5N1__segment4hemagglutinin_HA_genecompletecds|2022-10-29|4|H5N1|USA|Cathartesaura|USA_MN|Alphainfluenzavirus|Alphainfluenzavirusinfluenz"/>
NNN
</sequence>
<sequence>
<taxon idref="PQ719425.1|InfluenzaAvirus_A/TurkeyVulture/WY/22-031606-001-original-repeat/2022_H5N1__segment4hemagglutinin_HA_genecompletecds|2022-10-03|4|H5N1|USA|Cathartesaura|USA_WY|Alphainfluenzavirus|Alphainfluenzavirusinfluenz"/>
NNN
</sequence>
<sequence>
<taxon idref="PQ719473.1|InfluenzaAvirus_A/WesternGull/CA/22-035882-001-original/2022_H5N1__segment4hemagglutinin_HA_genecompletecds|2022-11-03|4|H5N1|USA|Larusoccidentalis|USA_CA|Alphainfluenzavirus|Alphainfluenzavirusinfluenz"/>
NNN
</sequence>
<sequence>
<taxon idref="PQ719481.1|InfluenzaAvirus_A/WesternGull/CA/22-037512-001-original/2022_H5N1__segment4hemagglutinin_HA_genecompletecds|2022-11-08|4|H5N1|USA|Larusoccidentalis|USA_CA|Alphainfluenzavirus|Alphainfluenzavirusinfluenz"/>
NNN
</sequence>
<sequence>
<taxon idref="PQ719521.1|InfluenzaAvirus_A/Westernscreechowl/WA/22-015526-006-original/2022_H5N1__segment4hemagglutinin_HA_genecompletecds|2022-05-11|4|H5N1|USA||USA_WA|Alphainfluenzavirus|Alphainfluenzavirusinfluenz"/>
NNN
</sequence>
<sequence>
<taxon idref="PQ719529.1|InfluenzaAvirus_A/Westernsnowyplover/OR/22-038785-001-original-repeat/2022_H5N1__segment4hemagglutinin_HA_genecompletecds|2022-10-25|4|H5N1|USA||USA_OR|Alphainfluenzavirus|Alphainfluenzavirusinfluenz"/>
NNN
</sequence>
<sequence>
<taxon idref="PQ719905.1|InfluenzaAvirus_A/blackskimmer/FL/22-036966-001-original/2022_H5N1__segment4hemagglutinin_HA_genecompletecds|2022-11-04|4|H5N1|USA|Rynchopsniger|USA_FL|Alphainfluenzavirus|Alphainfluenzavirusinfluenz"/>
NNN
</sequence>
<sequence>
<taxon idref="PQ719945.1|InfluenzaAvirus_A/goose/AR/22-036798-001-original/2022_H5N1__segment4hemagglutinin_HA_genecompletecds|2022-11-14|4|H5N1|USA|Anatidae|USA_AR|Alphainfluenzavirus|Alphainfluenzavirusinfluenz"/>
NNN
</sequence>
<sequence>
<taxon idref="PQ720105.1|InfluenzaAvirus_A/goose/TX/22-038236-001-original/2022_H5N1__segment4hemagglutinin_HA_genecompletecds|2022-11-

23|4|H5N1|USA|Anatidae|USA_TX|Alphainfluenzavirus|Alphainfluenzavirusinflue
nz"/>
NNN
</sequence>
<sequence>
<taxon idref="PQ720289.1|InfluenzaAvirus_A/lesserscaup/LA/22-037240-003-
original/2022_H5N1__segment4hemagglutinin_HA_genecompletecds|2022-11-
13|4|H5N1|USA|Aythyaaffinis|USA_LA|Alphainfluenzavirus|Alphainfluenzavirusi
nfluenz"/>
NNN
</sequence>
<sequence>
<taxon idref="PQ720297.1|InfluenzaAvirus_A/lessersnowgoose/OR/22-037719-
001-original/2022_H5N1__segment4hemagglutinin_HA_genecompletecds|2022-11-
08|4|H5N1|USA|Ansercaerulescenscaerulescens|USA_OR|Alphainfluenzavirus|Alph
ainfluenzavirusinfluenz"/>
NNN
</sequence>
<sequence>
<taxon idref="A/Brown_skua/Fildes_Peninsula/B1/2024(H5N1)|2024-12-
10|2.3.4.4b||PB2|EPI_ISL_19847535"/>
NNN
</sequence>
<sequence>
<taxon idref="A/Brown_skua/Fildes_Peninsula/B3/2024(H5N1)|2024-12-
26|2.3.4.4b||PB2|EPI_ISL_19847538"/>
NNN
</sequence>
<sequence>
<taxon idref="A/Brown_skua/Fildes_Peninsula/F4/2024(H5N1)|2024-12-
26|2.3.4.4b||PB2|EPI_ISL_19847539"/>
NNN
</sequence>
<sequence>
<taxon idref="A/Brown_skua/Fildes_Peninsula/B2/2024(H5N1)|2024-12-
25|2.3.4.4b||PB2|EPI_ISL_19847536"/>
NNN
</sequence>
<sequence>
<taxon idref="A/Brown_Skua/Torgersen_Island/o81-b82/2024|2024-12-
17|2.3.4.4b||PB2|EPI_ISL_19645365"/>
NNN
</sequence>
<sequence>
<taxon idref="A/Brown_skua/Torgersen_Island/o8182/2024|2024-12-
01|2.3.4.4b||PB2|EPI_ISL_19745586"/>
NNN
</sequence>
</alignment>
<!-- The unique patterns from 1 to end
-->
<!-- npatterns=7065
-->
<patterns id="patterns" from="1" strip="false">
<alignment idref="alignment"/>
</patterns>
<!-- This is a simple constant population size coalescent model
-->
<!-- that is used to generate an initial tree for the chain.
-->
<constantSize id="initialDemo" units="years">

```
<populationSize>
<parameter id="initialDemo.popSize" value="10.0"/>
</populationSize>
</constantSize>
<!--  Generate a random starting tree under the coalescent process
-->
<coalescentSimulator id="startingTree">
<taxa idref="taxa"/>
<constantSize idref="initialDemo"/>
</coalescentSimulator>
<!--  Generate a tree model
-->
<treeModel id="treeModel">
<coalescentTree idref="startingTree"/>
<rootHeight>
<parameter id="treeModel.rootHeight"/>
</rootHeight>
<nodeHeights internalNodes="true">
<parameter id="treeModel.internalNodeHeights"/>
</nodeHeights>
<nodeHeights internalNodes="true" rootNode="true">
<parameter id="treeModel.allInternalNodeHeights"/>
</nodeHeights>
</treeModel>
<!--  Statistic for height of the root of the tree
-->
<treeHeightStatistic id="rootHeight">
<treeModel idref="treeModel"/>
</treeHeightStatistic>
<!--  Statistic for sum of the branch lengths of the tree (tree length)
-->
<treeLengthStatistic id="treeLength">
<treeModel idref="treeModel"/>
</treeLengthStatistic>
<!--  Statistic for time of most recent common ancestor of tree
-->
<tmrcaStatistic id="age(root)" absolute="true">
<treeModel idref="treeModel"/>
</tmrcaStatistic>
<!--  Generate a gmrfSkyGridLikelihood for the Bayesian SkyGrid process
-->
<gmrfSkyGridLikelihood id="skygrid">
<populationSizes>
<!--  skygrid.logPopSize is in log units unlike other popSize
-->
<parameter id="skygrid.logPopSize" dimension="60" value="1.0"/>
</populationSizes>
<precisionParameter>
<parameter id="skygrid.precision" value="0.1" lower="0.0"/>
</precisionParameter>
<numGridPoints>
<parameter id="skygrid.numGridPoints" value="59.0"/>
</numGridPoints>
<cutOff>
<parameter id="skygrid.cutOff" value="5.0"/>
</cutOff>
<populationTree>
<treeModel idref="treeModel"/>
</populationTree>
</gmrfSkyGridLikelihood>
```

```
<gammaPrior id="skygrid.precision.prior" shape="0.001" scale="1000.0" offse
t="0.0">
<parameter idref="skygrid.precision"/>
</gammaPrior>
<gmrfSkyrideGradient id="gmrfGradientPop" wrtParameter="logPopulationSizes"
>
<gmrfSkyGridLikelihood idref="skygrid"/>
</gmrfSkyrideGradient>
<compoundParameter id="skygrid.parameters">
<parameter idref="skygrid.precision"/>
<parameter idref="skygrid.logPopSize"/>
</compoundParameter>
<gmrfSkyrideGradient id="gmrfGradientPrec" wrtParameter="precision">
<gmrfSkyGridLikelihood idref="skygrid"/>
</gmrfSkyrideGradient>
<jointGradient id="joint.skygrid.precision">
<gmrfSkyrideGradient idref="gmrfGradientPrec"/>
<gradient>
<gammaPrior idref="skygrid.precision.prior"/>
<parameter idref="skygrid.precision"/>
</gradient>
</jointGradient>
<compoundGradient id="full.skygrid.gradient">
<jointGradient idref="joint.skygrid.precision"/>
<gmrfSkyrideGradient idref="gmrfGradientPop"/>
</compoundGradient>
<!--  The Hamiltonian Monte Carlo relaxed clock
-->
<arbitraryBranchRates id="branchRates" centerAtOne="false">
<treeModel idref="treeModel"/>
<rates>
<parameter id="branchRatesHMC.rates" value="0.001" lower="0.0"/>
</rates>
<location>
<parameter id="branchRatesHMC.rate" value="0.001" lower="0.0"/>
</location>
<scale>
<parameter id="branchRatesHMC.scale" value="1.0" lower="0.0"/>
</scale>
</arbitraryBranchRates>
<distributionLikelihood id="ratesPrior">
<data>
<parameter idref="branchRatesHMC.rates"/>
</data>
<distribution>
<logNormalDistributionModel meanInRealSpace="true">
<mean>
<parameter value="1.0" lower="0.0"/>
</mean>
<stdev>
<parameter value="0.1" lower="0.0"/>
</stdev>
</logNormalDistributionModel>
</distribution>
</distributionLikelihood>
<rateStatistic id="meanRate" name="meanRate" mode="mean" internal="true" ex
ternal="true">
<treeModel idref="treeModel"/>
<arbitraryBranchRates idref="branchRates"/>
</rateStatistic>
```

```
<rateStatistic id="coefficientOfVariation" name="coefficientOfVariation" mo
de="coefficientOfVariation" internal="true" external="true">
<treeModel idref="treeModel"/>
<arbitraryBranchRates idref="branchRates"/>
</rateStatistic>
<rateCovarianceStatistic id="covariance" name="covariance">
<treeModel idref="treeModel"/>
<arbitraryBranchRates idref="branchRates"/>
</rateCovarianceStatistic>
<distributionLikelihood id="scalePrior">
<data>
<parameter idref="branchRatesHMC.scale"/>
</data>
<distribution>
<exponentialDistributionModel>
<mean>
<parameter value="1.0" lower="0.0"/>
</mean>
</exponentialDistributionModel>
</distribution>
</distributionLikelihood>
<compoundParameter id="locationScale">
<parameter idref="branchRatesHMC.rate"/>
<parameter idref="branchRatesHMC.scale"/>
</compoundParameter>
<ctmcScalePrior id="locationPrior">
<ctmcScale>
<parameter idref="branchRatesHMC.rate"/>
</ctmcScale>
<treeModel idref="treeModel"/>
</ctmcScalePrior>
<!--  The general time reversible (GTR) substitution model
-->
<gtrModel id="gtr">
<frequencies>
<frequencyModel dataType="nucleotide">
<frequencies>
<parameter id="frequencies" value="0.25 0.25 0.25 0.25"/>
</frequencies>
</frequencyModel>
</frequencies>
<rates>
<parameter id="gtr.rates" dimension="6" value="1.0" lower="0.0"/>
</rates>
</gtrModel>
<!--  site model
-->
<siteModel id="siteModel">
<substitutionModel>
<gtrModel idref="gtr"/>
</substitutionModel>
<gammaShape gammaCategories="4">
<parameter id="alpha" value="0.5" lower="0.0"/>
</gammaShape>
</siteModel>
<!--
-->
<statistic id="mu" name="mu">
<siteModel idref="siteModel"/>
</statistic>
```

```xml
<!--  Likelihood for tree given sequence data
-->
<treeDataLikelihood id="treeLikelihood" useAmbiguities="false" usePreOrder=
"true">
<partition>
<patterns idref="patterns"/>
<siteModel idref="siteModel"/>
</partition>
<treeModel idref="treeModel"/>
<branchRates idref="branchRates"/>
</treeDataLikelihood>
<!--  Define operators
-->
<operators id="operators" optimizationSchedule="log">
<deltaExchange delta="0.01" weight="1">
<parameter idref="gtr.rates"/>
</deltaExchange>
<deltaExchange delta="0.01" weight="1">
<parameter idref="frequencies"/>
</deltaExchange>
<scaleOperator scaleFactor="0.75" weight="1">
<parameter idref="alpha"/>
</scaleOperator>
<scaleOperator scaleFactor="0.75" weight="3">
<parameter idref="branchRatesHMC.rate"/>
</scaleOperator>
<hamiltonianMonteCarloOperator weight="1" nSteps="4" stepSize="0.01" autoOp
timize="true" mode="vanilla" gradientCheckCount="0" preconditioning="diagon
al" preconditioningUpdateFrequency="10">
<jointGradient>
<hessian>
<distributionLikelihood idref="ratesPrior"/>
<parameter idref="branchRatesHMC.rates"/>
</hessian>
<branchRateGradient traitName="Sequence">
<treeDataLikelihood idref="treeLikelihood"/>
</branchRateGradient>
</jointGradient>
<parameter idref="branchRatesHMC.rates"/>
<signTransform>
<parameter idref="branchRatesHMC.rates"/>
</signTransform>
</hamiltonianMonteCarloOperator>
<scaleOperator scaleFactor="0.75" weight="3">
<parameter idref="branchRatesHMC.scale"/>
</scaleOperator>
<upDownOperator scaleFactor="0.75" weight="3">
<up>
<parameter idref="treeModel.allInternalNodeHeights"/>
</up>
<down>
<parameter idref="branchRatesHMC.rate"/>
</down>
</upDownOperator>
<subtreeSlide size="1.0" gaussian="true" weight="30">
<treeModel idref="treeModel"/>
</subtreeSlide>
<narrowExchange weight="30">
<treeModel idref="treeModel"/>
</narrowExchange>
<wideExchange weight="3">
```

```
<treeModel idref="treeModel"/>
</wideExchange>
<wilsonBalding weight="3">
<treeModel idref="treeModel"/>
</wilsonBalding>
<scaleOperator scaleFactor="0.75" weight="3">
<parameter idref="treeModel.rootHeight"/>
</scaleOperator>
<uniformOperator weight="30">
<parameter idref="treeModel.internalNodeHeights"/>
</uniformOperator>
<hamiltonianMonteCarloOperator weight="2" nSteps="50" stepSize="0.01" mode=
"vanilla" autoOptimize="true" gradientCheckCount="0" gradientCheckTolerance
="0.1" preconditioning="none" preconditioningUpdateFrequency="100">
<compoundGradient idref="full.skygrid.gradient"/>
<compoundParameter idref="skygrid.parameters"/>
<signTransform start="1" end="1">
<compoundParameter idref="skygrid.parameters"/>
</signTransform>
</hamiltonianMonteCarloOperator>
</operators>
<!--  Define MCMC
-->
<mcmc id="mcmc" chainLength="500000000" autoOptimize="true" operatorAnalysi
s="revision_alignment.ops">
<joint id="joint">
<prior id="prior">
<dirichletPrior alpha="1.0" sumsTo="6.0">
<parameter idref="gtr.rates"/>
</dirichletPrior>
<dirichletPrior alpha="1.0" sumsTo="1.0">
<parameter idref="frequencies"/>
</dirichletPrior>
<exponentialPrior mean="0.5" offset="0.0">
<parameter idref="alpha"/>
</exponentialPrior>
<ctmcScalePrior idref="locationPrior"/>
<distributionLikelihood idref="ratesPrior"/>
<distributionLikelihood idref="scalePrior"/>
<gammaPrior idref="skygrid.precision.prior"/>
<gmrfSkyGridLikelihood idref="skygrid"/>
<branchRates idref="branchRates"/>
</prior>
<likelihood id="likelihood">
<treeDataLikelihood idref="treeLikelihood"/>
</likelihood>
</joint>
<operators idref="operators"/>
<!--  write log to screen
-->
<log id="screenLog" logEvery="10000">
<column label="Joint" dp="4" width="12">
<joint idref="joint"/>
</column>
<column label="Prior" dp="4" width="12">
<prior idref="prior"/>
</column>
<column label="Likelihood" dp="4" width="12">
<likelihood idref="likelihood"/>
</column>
<column label="age(root)" sf="6" width="12">
```

```
<tmrcaStatistic idref="age(root)"/>
</column>
<column label="branchRatesHMC.rate" sf="6" width="12">
<parameter idref="branchRatesHMC.rate"/>
</column>
</log>
<!--  write log to file
-->
<log id="fileLog" logEvery="10000" fileName="revision_alignment.log" overwr
ite="false">
<joint idref="joint"/>
<prior idref="prior"/>
<likelihood idref="likelihood"/>
<treeHeightStatistic idref="rootHeight"/>
<tmrcaStatistic idref="age(root)"/>
<treeLengthStatistic idref="treeLength"/>
<parameter idref="skygrid.precision"/>
<parameter idref="skygrid.logPopSize"/>
<parameter idref="skygrid.cutOff"/>
<parameter idref="gtr.rates"/>
<parameter idref="frequencies"/>
<parameter idref="alpha"/>
<parameter idref="branchRatesHMC.rate"/>
<parameter idref="branchRatesHMC.scale"/>
<rateStatistic idref="meanRate"/>
<rateStatistic idref="coefficientOfVariation"/>
<rateCovarianceStatistic idref="covariance"/>
<treeDataLikelihood idref="treeLikelihood"/>
<branchRates idref="branchRates"/>
<gmrfSkyGridLikelihood idref="skygrid"/>
</log>
<!--  write tree log to file
-->
<logTree id="treeFileLog" logEvery="50000" nexusFormat="true" fileName="rev
ision_alignment.trees" sortTranslationTable="true">
<treeModel idref="treeModel"/>
<trait name="rate" tag="rate">
<arbitraryBranchRates idref="branchRates"/>
</trait>
<joint idref="joint"/>
</logTree>
<!--  write state of Markov chain to checkpoint file
-->
<logCheckpoint id="checkpointFileLog" checkpointEvery="1000000" checkpointF
inal="500000000" fileName="revision_alignment.chkpt" overwrite="false"/>
</mcmc>
<report>
<property name="timer">
<mcmc idref="mcmc"/>
</property>
</report>
</beast>
```
