# Supplementary material for: Circumpolar spread of avian influenza H5N1 to southern Indian Ocean islands
Source: Nat Commun. 2025 Sep 29;16:8463. doi: 10.1038/s41467-025-64297-y (PMC12479825; doi:10.1038/s41467-025-64297-y)
Supplement: Supplementary file 8 — Supplementary Data 5 [file 41467_2025_64297_MOESM8_ESM.pdf]

SUPPLEMENTAL TABLE

Data Availability

GISAID Identifier:	EPI_SET_250304qg
doi:	10.55876/gis8.250304qg

All genome sequences and associated metadata in this dataset are published in GISAID's EpiFlu database. To view the contributors of each individual sequence with details such as accession number, Virus name, Collection date, Originating Lab and Submitting Lab and the list of Authors, visit 10.55876/gis8.250304qg

Data Snapshot

- EPI_SET_250304qg is composed of 8466 individual viruses;
- The collection dates range from 2022-12-01 to 2024-12-07;
- Data were collected in 75 countries and territories.
