# Supplementary material for: Circumpolar spread of avian influenza H5N1 to southern Indian Ocean islands
Source: Nat Commun. 2025 Sep 29;16:8463. doi: 10.1038/s41467-025-64297-y (PMC12479825; doi:10.1038/s41467-025-64297-y)
Supplement: Supplementary file 13 — Reporting Summary [file 41467_2025_64297_MOESM13_ESM.pdf]

nature portfolio

Corresponding author(s): Thierry Boulinier

Last updated by author(s): 2025/08/12

Reporting Summary

Nature Portfolio wishes to improve the reproducibility of the work that we publish. This form provides structure for consistency and transparency in reporting. For further information on Nature Portfolio policies, see our Editorial Policies and the Editorial Policy Checklist.

Statistics

For all statistical analyses, confirm that the following items are present in the figure legend, table legend, main text, or Methods section.

n/a	Confirmed	
☐	☒	The exact sample size (*n*) for each experimental group/condition, given as a discrete number and unit of measurement
☐	☒	A statement on whether measurements were taken from distinct samples or whether the same sample was measured repeatedly
☒	☐	The statistical test(s) used AND whether they are one- or two-sided<br>*Only common tests should be described solely by name; describe more complex techniques in the Methods section.*
☒	☐	A description of all covariates tested
☒	☐	A description of any assumptions or corrections, such as tests of normality and adjustment for multiple comparisons
☒	☐	A full description of the statistical parameters including central tendency (e.g. means) or other basic estimates (e.g. regression coefficient) AND variation (e.g. standard deviation) or associated estimates of uncertainty (e.g. confidence intervals)
☒	☐	For null hypothesis testing, the test statistic (e.g. *F*, *t*, *r*) with confidence intervals, effect sizes, degrees of freedom and *P* value noted<br>*Give P values as exact values whenever suitable.*
☐	☒	For Bayesian analysis, information on the choice of priors and Markov chain Monte Carlo settings
☒	☐	For hierarchical and complex designs, identification of the appropriate level for tests and full reporting of outcomes
☒	☐	Estimates of effect sizes (e.g. Cohen's *d*, Pearson's *r*), indicating how they were calculated

Our web collection on statistics for biologists contains articles on many of the points above.

Software and code

Policy information about availability of computer code

Data collection	On December 26th, 2024, we downloaded all available H5N1 HA sequences as well as full genomes from GenBank 55 and GISAID 49,50. We first removed duplicate entries and complemented the resulting data set with our 25 newly generated H5N1 2.3.4.4b sequences from the Crozet and Kerguelen archipelagos (Extended Data Table 1). We aligned the resulting HA data set using MAFFT v7.490 56, followed by manual / visual inspection. This yielded an initial data set containing 15072 sequences. We then performed maximum-likelihood (ML) phylogenetic inference using IQ-TREE v2.2.2.6 57 with automated model selection using ModelFinder 58, which selected the GTR+F+I+R6 model according to the Bayesian Information Criterion. We performed 1000 bootstrap replicates using the ultrafast bootstrap approximation approach (UFBoot) 59,60. From this large ML phylogeny, we extracted a large, well-supported (bootstrap support 94%) cluster that contained our 25 novel HPAI H5N1 2.3.4.4b sequences from the Crozet and Kerguelen archipelagos, to be used in subsequent analyses. Within this cluster, our 25 sequences were included within an 83-sequences clade that had 100% bootstrap support and contained sequences from South Georgia and the South Sandwich islands, as well as Antarctica.
Data analysis	Details are provided as the phylogenetic analyses are central to the study. We proceeded to construct a full-genome data set for this cluster to improve phylogenetic resolution in a subsequent Bayesian phylogenetic analysis. We followed the approach in Uhart et al. 17 in that such a workflow limits the time for H5N1 to accrue mutations and diversify, thereby limiting genetic diversity, but did not restrict ourselves to only complete genomes in order to reduce both geographic and genetic biases 61. We removed reassortant sequences while constructing ML trees to assess temporal signal in this complete genome data set and remove outliers using TempEst 62. This resulted in a final data set consisting of 1221 genomic sequences which exhibited a clear temporal signal.<br>We continued to perform a Bayesian time-calibrated phylogenetic analysis on this data set using Markov chain Monte Carlo as implemented in BEAST v1.10.5 63, employing the BEAGLE high-performance computational library for computational efficiency to run on a powerful graphics processing unit 64. We employed a non-parametric coalescent model 65,66 as the tree prior, a general time-reversible substitution model 67 with a discretized gamma distribution to accommodate among-site rate heterogeneity 68, and an uncorrelated relaxed

clock with an underlying lognormal distribution 69. We used the default priors in BEAST v1.10.5 63, including a conditional reference prior on the mean evolutionary rate 70. We ran this analysis until all relevant effective sample sizes reached at least 200, as assessed in Tracer v1.7.3 71, and checked if independent replicate analyses converged to the same posterior distribution. Based on the sampled posterior phylogenetic trees from this analysis, we performed a Bayesian discrete phylogeographic analysis using an asymmetric continuous-time Markov chain model with Bayesian stochastic search variable selection 72. The following locations were present in our 1221-taxa data set and used as discrete states: Antarctica, Argentina, Bolivia, Brasil, Canada, Chile, Colombia, Costa Rica, Crozet islands, Ecuador, Falkland islands, Guatemala, Honduras, Kerguelen islands, Panama, Peru, South Georgia islands, United States, and Uruguay. We used the highest independent posterior subtree reconstruction (HIPSTR) approach in TreeAnnotator X 29 to construct a time-calibrated consensus phylogeny, after removing 10% of the chain as burn-in.

For manuscripts utilizing custom algorithms or software that are central to the research but not yet described in published literature, software must be made available to editors and reviewers. We strongly encourage code deposition in a community repository (e.g. GitHub). See the Nature Portfolio guidelines for submitting code & software for further information.

Data

Policy information about availability of data

All manuscripts must include a data availability statement. This statement should provide the following information, where applicable:

- Accession codes, unique identifiers, or web links for publicly available datasets
- A description of any restrictions on data availability
- For clinical datasets or third party data, please ensure that the statement adheres to our policy

A detailed data availability statement is provided in the manuscript with all the necessary and required information.

Research involving human participants, their data, or biological material

Policy information about studies with human participants or human data. See also policy information about sex, gender (identity/presentation), and sexual orientation and race, ethnicity and racism.

Reporting on sex and gender	*Use the terms sex (biological attribute) and gender (shaped by social and cultural circumstances) carefully in order to avoid confusing both terms. Indicate if findings apply to only one sex or gender; describe whether sex and gender were considered in study design; whether sex and/or gender was determined based on self-reporting or assigned and methods used.*<br>*Provide in the source data disaggregated sex and gender data, where this information has been collected, and if consent has been obtained for sharing of individual-level data; provide overall numbers in this Reporting Summary. Please state if this information has not been collected.*<br>*Report sex- and gender-based analyses where performed, justify reasons for lack of sex- and gender-based analysis.*
Reporting on race, ethnicity, or other socially relevant groupings	*Please specify the socially constructed or socially relevant categorization variable(s) used in your manuscript and explain why they were used. Please note that such variables should not be used as proxies for other socially constructed/relevant variables (for example, race or ethnicity should not be used as a proxy for socioeconomic status).*<br>*Provide clear definitions of the relevant terms used, how they were provided (by the participants/respondents, the researchers, or third parties), and the method(s) used to classify people into the different categories (e.g. self-report, census or administrative data, social media data, etc.)*<br>*Please provide details about how you controlled for confounding variables in your analyses.*
Population characteristics	*Describe the covariate-relevant population characteristics of the human research participants (e.g. age, genotypic information, past and current diagnosis and treatment categories). If you filled out the behavioural & social sciences study design questions and have nothing to add here, write "See above."*
Recruitment	*Describe how participants were recruited. Outline any potential self-selection bias or other biases that may be present and how these are likely to impact results.*
Ethics oversight	*Identify the organization(s) that approved the study protocol.*

Note that full information on the approval of the study protocol must also be provided in the manuscript.

Field-specific reporting

Please select the one below that is the best fit for your research. If you are not sure, read the appropriate sections before making your selection.

☐ Life sciences ☐ Behavioural & social sciences ☒ Ecological, evolutionary & environmental sciences

For a reference copy of the document with all sections, see nature.com/documents/nr-reporting-summary-flat.pdf

Life sciences study design

All studies must disclose on these points even when the disclosure is negative.

Sample size	*Describe how sample size was determined, detailing any statistical methods used to predetermine sample size OR if no sample-size calculation was performed, describe how sample sizes were chosen and provide a rationale for why these sample sizes are sufficient.*

Data exclusions	*Describe any data exclusions. If no data were excluded from the analyses, state so OR if data were excluded, describe the exclusions and the rationale behind them, indicating whether exclusion criteria were pre-established.*
Replication	*Describe the measures taken to verify the reproducibility of the experimental findings. If all attempts at replication were successful, confirm this OR if there are any findings that were not replicated or cannot be reproduced, note this and describe why.*
Randomization	*Describe how samples/organisms/participants were allocated into experimental groups. If allocation was not random, describe how covariates were controlled OR if this is not relevant to your study, explain why.*
Blinding	*Describe whether the investigators were blinded to group allocation during data collection and/or analysis. If blinding was not possible, describe why OR explain why blinding was not relevant to your study.*

Behavioural & social sciences study design

All studies must disclose on these points even when the disclosure is negative.

Study description	*Briefly describe the study type including whether data are quantitative, qualitative, or mixed-methods (e.g. qualitative cross-sectional, quantitative experimental, mixed-methods case study).*
Research sample	*State the research sample (e.g. Harvard university undergraduates, villagers in rural India) and provide relevant demographic information (e.g. age, sex) and indicate whether the sample is representative. Provide a rationale for the study sample chosen. For studies involving existing datasets, please describe the dataset and source.*
Sampling strategy	*Describe the sampling procedure (e.g. random, snowball, stratified, convenience). Describe the statistical methods that were used to predetermine sample size OR if no sample-size calculation was performed, describe how sample sizes were chosen and provide a rationale for why these sample sizes are sufficient. For qualitative data, please indicate whether data saturation was considered, and what criteria were used to decide that no further sampling was needed.*
Data collection	*Provide details about the data collection procedure, including the instruments or devices used to record the data (e.g. pen and paper, computer, eye tracker, video or audio equipment) whether anyone was present besides the participant(s) and the researcher, and whether the researcher was blind to experimental condition and/or the study hypothesis during data collection.*
Timing	*Indicate the start and stop dates of data collection. If there is a gap between collection periods, state the dates for each sample cohort.*
Data exclusions	*If no data were excluded from the analyses, state so OR if data were excluded, provide the exact number of exclusions and the rationale behind them, indicating whether exclusion criteria were pre-established.*
Non-participation	*State how many participants dropped out/declined participation and the reason(s) given OR provide response rate OR state that no participants dropped out/declined participation.*
Randomization	*If participants were not allocated into experimental groups, state so OR describe how participants were allocated to groups, and if allocation was not random, describe how covariates were controlled.*

Ecological, evolutionary & environmental sciences study design

All studies must disclose on these points even when the disclosure is negative.

Study description	Provided in the manuscript. Extensive multi-host species field sampling of carcasses from die-offs suspected to be due to high pathogenic avian influenza, (HPAI) followed by qRT-PCR, sequencing and phylogenetic analyses; in addition, field blood sampling to determine antibody levelds against avian influenza virus was conducted in elephant seals, prior and during the HPAI outbreak, followed by ELISA analyses in the lab.
Research sample	Described in the manuscript: samples from carcasses sampled at locations where abnormal die-offs were recorded; plasma samples from sampling of live southernn elephant seals in Kerguelen.
Sampling strategy	Outlined in the manuscript: extensive sampling of carcasses of all species at field sites. To insure efficient detection and sequencing of the HPAI, brain swab samples were collected. For plasma sampling for serology, random sampling of live individuals.
Data collection	Describded in the manuscript. Sampling of brain swabs followed by storage at -80°C or in RNA-shield. Co-Authors (JT, ML, CDP) contributed to most of the field sampling of carcasses.
Timing and spatial scale	Field sampling of carcasses between October until December 2024; on Crozet and Kerguelen (maps provided for sampling locations)
Data exclusions	No data exclusions
Reproducibility	Use of reproductible laboratory methods. Repeated implementation oof analyses on subsamples for the serology analyses.

Randomization	Field sampling is described in the manuscript: attempt to sample all fresh carcasses; random sampling of available individuals for the serology study
Blinding	Blinding with regards to qRtPCR sampling as laboeratory analyses were conducted independrandtly of field sampling (although most samples that were submitted to analyses were expected to be positive)..

Did the study involve field work? ☒ Yes ☐ No

Field work, collection and transport

Field conditions	Sub-antarctic field conditions facilitated by support from the French Polar Institute (IPEV) and TAAF (French Southern Lands Authorities), notably for access to the remote sites considered in the study.
Location	Crozet (46°24′ S, 51°45′ E) and Kerguelen (49°15′ S, 69°10′ E) archipelagos, Southern Indian Ocean, with detgails provided in the manuscript.
Access & import/export	Permits from the TAAF authority (arrêtés préfectoraux), and Ministry of research: Permits to handle animals were obtained from the French Ministry of Research and the Animal experimental research authority (APAFIS #31773-2019112519421390 v4). Sampling was conducted under permits from Préfecture des TAAF (A-2018-126, A-2023-82, A-2023-159) after evaluation by the Conseil de l'Environnement Polaire and Conseil National de la Protection de la Nature. After the suspicion of HPAI, permits were granted by the Préfecture des TAAF to pursue eco-epidemiological fieldwork (A-2024-92, A-2024-93, A-2024-95, A-2024-144).. Import permits from French authorities (DDPP) and CITES.for the marine mammals.
Disturbance	Limited disturbance of the wild populations within the National Nature of the French Southern Lands, with applications for access made to the TAAF authorities, examinded by the Comité de l'Environnement Polaire and CNPN comittee..

Reporting for specific materials, systems and methods

We require information from authors about some types of materials, experimental systems and methods used in many studies. Here, indicate whether each material, system or method listed is relevant to your study. If you are not sure if a list item applies to your research, read the appropriate section before selecting a response.

Materials & experimental systems

n/a	Involved in the study	
☒	☐	Antibodies
☒	☐	Eukaryotic cell lines
☒	☐	Palaeontology and archaeology
☐	☒	Animals and other organisms
☒	☐	Clinical data
☒	☐	Dual use research of concern
☒	☐	Plants

Methods

n/a	Involved in the study	
☒	☐	ChIP-seq
☒	☐	Flow cytometry
☒	☐	MRI-based neuroimaging

Eukaryotic cell lines

Policy information about cell lines and Sex and Gender in Research

Cell line source(s)	*State the source of each cell line used and the sex of all primary cell lines and cells derived from human participants or vertebrate models.*
Authentication	*Describe the authentication procedures for each cell line used OR declare that none of the cell lines used were authenticated.*
Mycoplasma contamination	*Confirm that all cell lines tested negative for mycoplasma contamination OR describe the results of the testing for mycoplasma contamination OR declare that the cell lines were not tested for mycoplasma contamination.*
Commonly misidentified lines (See ICLAC register)	*Name any commonly misidentified cell lines used in the study and provide a rationale for their use.*

Palaeontology and Archaeology

Specimen provenance	*Provide provenance information for specimens and describe permits that were obtained for the work (including the name of the issuing authority, the date of issue, and any identifying information). Permits should encompass collection and, where applicable, export.*

Specimen deposition	*Indicate where the specimens have been deposited to permit free access by other researchers.*
Dating methods	*If new dates are provided, describe how they were obtained (e.g. collection, storage, sample pretreatment and measurement), where they were obtained (i.e. lab name), the calibration program and the protocol for quality assurance OR state that no new dates are provided.*

☐ Tick this box to confirm that the raw and calibrated dates are available in the paper or in Supplementary Information.

Ethics oversight	*Identify the organization(s) that approved or provided guidance on the study protocol, OR state that no ethical approval or guidance was required and explain why not.*

Note that full information on the approval of the study protocol must also be provided in the manuscript.

Animals and other research organisms

Policy information about studies involving animals; ARRIVE guidelines recommended for reporting animal research, and Sex and Gender in Research

Laboratory animals	No
Wild animals	Live animals were adult females of elephant seals in 2018, 2022 and 2023 and 26 pups of elephants seals captured for sampling in Kerguelen. in 2024 All animals were released on site after sampling.
Reporting on sex	Only adult females sampled for serology of adult elephant seals. Otherwises, no information available on sex.
Field-collected samples	Yes. Sampling of dead and alive individuals of protected wild marine species on islands of the southern Indian Ocean.
Ethics oversight	Assessed via the Occitanie Regional Animal Experimentation Committee and Miniqtry of Research (APAFIS). Permits to handle animals were obtained from the French Ministry of Research and the Animal experimental research authority (APAFIS #31773-2019112519421390 v4). Sampling was conducted under permits from Préfecture des TAAF (A-2018-126, A-2023-82, A-2023-159) after evaluation by the Conseil de l'Environnement Polaire and Conseil National de la Protection de la Nature.

Note that full information on the approval of the study protocol must also be provided in the manuscript.

Clinical data

Policy information about clinical studies

All manuscripts should comply with the ICMJEguidelines for publication of clinical research and a completedCONSORT checklist must be included with all submissions.

Clinical trial registration	*Provide the trial registration number from ClinicalTrials.gov or an equivalent agency.*
Study protocol	*Note where the full trial protocol can be accessed OR if not available, explain why.*
Data collection	*Describe the settings and locales of data collection, noting the time periods of recruitment and data collection.*
Outcomes	*Describe how you pre-defined primary and secondary outcome measures and how you assessed these measures.*

Dual use research of concern

Policy information about dual use research of concern

Hazards

Could the accidental, deliberate or reckless misuse of agents or technologies generated in the work, or the application of information presented in the manuscript, pose a threat to:

No	Yes	
☒	☐	Public health
☒	☐	National security
☒	☐	Crops and/or livestock
☒	☐	Ecosystems
☒	☐	Any other significant area

Experiments of concern

Does the work involve any of these experiments of concern:

No	Yes	
☒	☐	Demonstrate how to render a vaccine ineffective
☒	☐	Confer resistance to therapeutically useful antibiotics or antiviral agents
☒	☐	Enhance the virulence of a pathogen or render a nonpathogen virulent
☒	☐	Increase transmissibility of a pathogen
☒	☐	Alter the host range of a pathogen
☒	☐	Enable evasion of diagnostic/detection modalities
☒	☐	Enable the weaponization of a biological agent or toxin
☒	☐	Any other potentially harmful combination of experiments and agents

Plants

Seed stocks

Report on the source of all seed stocks or other plant material used. If applicable, state the seed stock centre and catalogue number. If plant specimens were collected from the field, describe the collection location, date and sampling procedures.

Novel plant genotypes

Describe the methods by which all novel plant genotypes were produced. This includes those generated by transgenic approaches, gene editing, chemical/radiation-based mutagenesis and hybridization. For transgenic lines, describe the transformation method, the number of independent lines analyzed and the generation upon which experiments were performed. For gene-edited lines, describe the editor used, the endogenous sequence targeted for editing, the targeting guide RNA sequence (if applicable) and how the editor was applied.

Authentication

Describe any authentication procedures for each seed stock used or novel genotype generated. Describe any experiments used to assess the effect of a mutation and, where applicable, how potential secondary effects (e.g. second site T-DNA insertions, mosiacism, off-target gene editing) were examined.

ChIP-seq

Data deposition

☐ Confirm that both raw and final processed data have been deposited in a public database such as GEO.

☐ Confirm that you have deposited or provided access to graph files (e.g. BED files) for the called peaks.

Data access links
May remain private before publication.

For "Initial submission" or "Revised version" documents, provide reviewer access links. For your "Final submission" document, provide a link to the deposited data.

Files in database submission

Provide a list of all files available in the database submission.

Genome browser session
(e.g. UCSC)

Provide a link to an anonymized genome browser session for "Initial submission" and "Revised version" documents only, to enable peer review. Write "no longer applicable" for "Final submission" documents.

Methodology

Replicates

Describe the experimental replicates, specifying number, type and replicate agreement.

Sequencing depth

Describe the sequencing depth for each experiment, providing the total number of reads, uniquely mapped reads, length of reads and whether they were paired- or single-end.

Antibodies

Describe the antibodies used for the ChIP-seq experiments; as applicable, provide supplier name, catalog number, clone name, and lot number.

Peak calling parameters

Specify the command line program and parameters used for read mapping and peak calling, including the ChIP, control and index files used.

Data quality

Describe the methods used to ensure data quality in full detail, including how many peaks are at FDR 5% and above 5-fold enrichment.

Software

Describe the software used to collect and analyze the ChIP-seq data. For custom code that has been deposited into a community repository, provide accession details.

Flow Cytometry

Plots

Confirm that:

- [ ] The axis labels state the marker and fluorochrome used (e.g. CD4-FITC).
- [ ] The axis scales are clearly visible. Include numbers along axes only for bottom left plot of group (a 'group' is an analysis of identical markers).
- [ ] All plots are contour plots with outliers or pseudocolor plots.
- [ ] A numerical value for number of cells or percentage (with statistics) is provided.

Methodology

Sample preparation	*Describe the sample preparation, detailing the biological source of the cells and any tissue processing steps used.*
Instrument	*Identify the instrument used for data collection, specifying make and model number.*
Software	*Describe the software used to collect and analyze the flow cytometry data. For custom code that has been deposited into a community repository, provide accession details.*
Cell population abundance	*Describe the abundance of the relevant cell populations within post-sort fractions, providing details on the purity of the samples and how it was determined.*
Gating strategy	*Describe the gating strategy used for all relevant experiments, specifying the preliminary FSC/SSC gates of the starting cell population, indicating where boundaries between "positive" and "negative" staining cell populations are defined.*

- [ ] Tick this box to confirm that a figure exemplifying the gating strategy is provided in the Supplementary Information.

Magnetic resonance imaging

Experimental design

Design type	*Indicate task or resting state; event-related or block design.*
Design specifications	*Specify the number of blocks, trials or experimental units per session and/or subject, and specify the length of each trial or block (if trials are blocked) and interval between trials.*
Behavioral performance measures	*State number and/or type of variables recorded (e.g. correct button press, response time) and what statistics were used to establish that the subjects were performing the task as expected (e.g. mean, range, and/or standard deviation across subjects).*

Acquisition

Imaging type(s)	*Specify: functional, structural, diffusion, perfusion.*
Field strength	*Specify in Tesla*
Sequence & imaging parameters	*Specify the pulse sequence type (gradient echo, spin echo, etc.), imaging type (EPI, spiral, etc.), field of view, matrix size, slice thickness, orientation and TE/TR/flip angle.*
Area of acquisition	*State whether a whole brain scan was used OR define the area of acquisition, describing how the region was determined.*

Diffusion MRI ☐ Used ☐ Not used

Preprocessing

Preprocessing software	*Provide detail on software version and revision number and on specific parameters (model/functions, brain extraction, segmentation, smoothing kernel size, etc.).*
Normalization	*If data were normalized/standardized, describe the approach(es): specify linear or non-linear and define image types used for transformation OR indicate that data were not normalized and explain rationale for lack of normalization.*
Normalization template	*Describe the template used for normalization/transformation, specifying subject space or group standardized space (e.g. original Talairach, MNI305, ICBM152) OR indicate that the data were not normalized.*
Noise and artifact removal	*Describe your procedure(s) for artifact and structured noise removal, specifying motion parameters, tissue signals and physiological signals (heart rate, respiration).*

Volume censoring | *Define your software and/or method and criteria for volume censoring, and state the extent of such censoring.*

Statistical modeling & inference

Model type and settings | *Specify type (mass univariate, multivariate, RSA, predictive, etc.) and describe essential details of the model at the first and second levels (e.g. fixed, random or mixed effects; drift or auto-correlation).*

Effect(s) tested | *Define precise effect in terms of the task or stimulus conditions instead of psychological concepts and indicate whether ANOVA or factorial designs were used.*

Specify type of analysis: ☐ Whole brain ☐ ROI-based ☐ Both

Statistic type for inference | *Specify voxel-wise or cluster-wise and report all relevant parameters for cluster-wise methods.*

(See Eklund et al. 2016)

Correction | *Describe the type of correction and how it is obtained for multiple comparisons (e.g. FWE, FDR, permutation or Monte Carlo).*

Models & analysis

n/a	Involved in the study
☐	☐ Functional and/or effective connectivity
☐	☐ Graph analysis
☐	☐ Multivariate modeling or predictive analysis

Functional and/or effective connectivity | *Report the measures of dependence used and the model details (e.g. Pearson correlation, partial correlation, mutual information).*

Graph analysis | *Report the dependent variable and connectivity measure, specifying weighted graph or binarized graph, subject- or group-level, and the global and/or node summaries used (e.g. clustering coefficient, efficiency, etc.).*

Multivariate modeling and predictive analysis | *Specify independent variables, features extraction and dimension reduction, model, training and evaluation metrics.*
